# Structurally divergent enantioselective synthesis of benzofuran fused azocine derivatives and spiro-cyclopentanone benzofurans enabled by sequential catalysis†

**DOI:** 10.1039/d3sc03239f

**Published:** 2023-09-07

**Authors:** Rupkumar Khuntia, Sanat Kumar Mahapatra, Lisa Roy, Subhas Chandra Pan

**Affiliations:** a Department of Chemistry, Indian Institute of Technology Guwahati Assam 781039 India span@iitg.ac.in https://www.iitg.ac.in/span/; b Institute of Chemical Technology Mumbai IOC Odisha Campus Bhubaneswar Bhubaneswar 751013 India

## Abstract

An important objective in organic synthesis and medicinal chemistry is the capacity to access structurally varied and complex molecules rapidly and affordably from easily available starting materials. Herein, a protocol for the structurally divergent synthesis of benzofuran fused azocine derivatives and spiro-cyclopentanone benzofurans has been developed *via* chiral bifunctional urea catalyzed reaction between aurone-derived α,β-unsaturated imine and ynone followed by switchable divergent annulation reactions by Lewis base catalysts (DBU and PPh_3_) with concomitant epimerization. The skeletally diversified products were formed in high yields with high diastereo- and enantioselectivities. Computational analysis with DFT and accurate DLPNO-CCSD(T) has been employed to gain deeper insights into mechanistic intricacies and investigate the role of chiral and Lewis base catalysts in skeletal diversity.

## Abstract 2

We have developed a divergent approach for the catalytic asymmetric synthesis of skeletally different benzofuran fused azocine derivatives and spiro-cyclopentanone benzofurans. The methodology involves sequential catalysis: chiral H-bonding catalysis and Lewis base catalysis.

## Graphical abstract

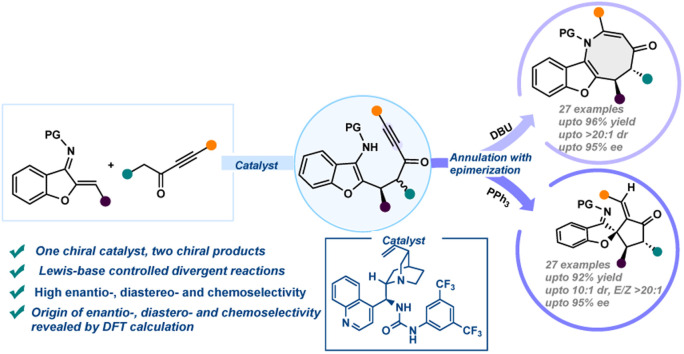

## Introduction

**Scheme 1:**
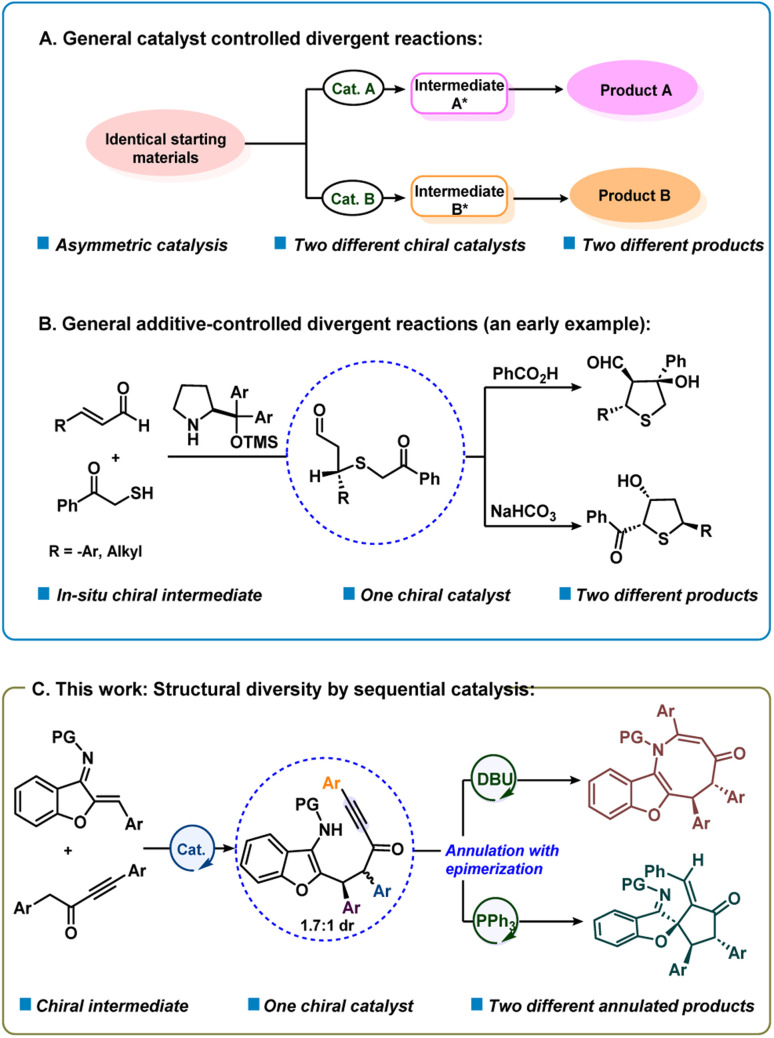
Catalytic asymmetric divergent reactions.

In recent years, significant attention has been given to the development of concise, green and efficient methodologies for the preparation of target molecules.^1^ Divergent synthesis is a fascinating and efficient strategy that has attracted much attention from chemists for the synthesis of diverse isomers with different chemo-, regio- or diastereoselectivities from identical starting materials.^2–4^ In fact, synthesis of two or more structurally and stereogenically different types of chiral products *via* a divergent strategy is quite attractive.^2*c*^ The principal strategy that has been used for the development of enantioselective divergent reactions is to employ different chiral catalysts to obtain product selectivity *via* different catalytic intermediates (catalyst controlled selectivity) (Scheme 1a).^3*d*–*s*^ On the other hand, it is quite challenging to obtain structurally different compounds using the same chiral catalyst and with different additives or reagents or varying reaction conditions (additive or reagent controlled).^3*a*–*c*,*t*–*x*^ In an early study, Jørgensen and co-workers reported an additive-controlled divergent synthesis of tetrahydrothiophenes using a diaryl prolinol TMS ether catalyst (Scheme 1b).^3*a*^ Interestingly, the authors observed different product formations using different additives, *i.e.*, PhCOOH and NaHCO_3_. Recently, the groups of Wang and Xu reported solvent dependent organocatalytic divergent reactions.^3*u*,*v*^ In the current work, we describe a unique divergent process by sequential catalysis^4^ where the first step uses a bifunctional catalyst and in the second step, the chiral intermediate participates in Lewis base controlled divergent annulation reactions (Scheme 1c). This method presents organocatalytic cycle-varying cascade reactions utilizing aurone-derived α,β-unsaturated imine.

Previously the groups of Huang, Cheng and Zhai reported achiral divergent reactions with aurone-derived α,β-unsaturated imine.^5^ To the best of our knowledge, the catalytic asymmetric divergent reaction is still not known using an aurone-derived. α,β-unsaturated imine. Also, benzofuran or indole-fused eight-membered rings containing a nitrogen atom (azocines) are important structural motifs that are prevalent in many natural products and biologically active molecules such as PKD inhibitor kb-NB96-53, balasubramide, grandilodine A, *etc.*^6^ The efficient construction of eight-membered rings in such molecular structures is a fundamental synthetic challenge.^7^ The inherent strains of the targeted azocines, as well as competing pathways leading to either the highly favoured six- or five-membered rings are the key hindrances for the formation of eight-membered rings. We envisioned that a benzofuran/indole-derived nitrogen-containing electrophilic partner such as aurone-derived α,β-unsaturated imine^8^ could be coupled with alkynyl ketones^9^ to deliver azocine. Similarly, spiro-cyclopentane benzofuran motifs are present in many bioactive natural products such as laurentristich-4-ol, spiroapplanatumine K and involucratustone B.^10^ However, only a few catalytic asymmetric syntheses of spiro-cyclopentanone benzofurans are known.^11^ Thus, an efficient route for the preparation of such a motif in enantioselective fashion is highly desirable. We speculated that such a motif can be generated by reacting only the C=C of aurone-derived α,β-unsaturated imine. Herein, we would like to disclose our extensive explorations on these issues.

## Results and discussion

The initial experiment involved performing the reaction be-tween *N*-sulfonyl 1-azadiene 1a and ynone 2a with bifunctional urea catalyst I in toluene at room temperature (Table 1, entry 1). A smooth conversion was observed in 12 hours to provide intermediate A with 1.7 : 1 dr, which after short column chromatography was treated with DBU. This resulted in an annulation reaction with concomitant epimerization^12^ for the formation of thermodynamically stable *trans* benzofuran fused azocine derivative 3a with 88% yield, >20 : 1 dr and 84% ee. Interestingly, Takemoto catalyst II with a thiourea motif afforded 3a with much less enantioselectivity (Table 1, entry 2). A good enantioselectivity of 80% was detected with *t*-leucine derived bifunctional urea catalyst III but thiourea derivative IV was not effective (Table 1, entries 3–4). Then we screened *t*-leucine derived bifunctional squaramide catalyst V in the reaction and moderate enantioselectivity was obtained (Table 1, entry 5). Then we turned our attention to employ cinchona alkaloid derived bifunctional urea and thiourea catalysts and this proved to be effective.^13^ Cinchonidine derived bifunctional urea catalyst VI promoted the reaction with 90% yield and 88% ee was observed (Table 1, entry 6). Thiourea derivative VII was not effective (Table 1, entry 7). Similar trends of enantioselectivities were observed with quinine derived bifunctional urea and thiourea catalysts VIII and IX (Table 1, entries 8–9). Bifunctional squaramide catalyst X also was not suitable for the reaction (Table 1, entry 10). Then we focused on the solvent optimization with catalyst VI (Table 1, entries 11–12). Slightly lower enantioselectivities were found in mesitylene and *o*-xylene solvents. Other solvents also could not improve the enantioselectivity (see the ESI† for details). Then the reaction was run in toluene at lower temperatures (Table 1, entries 13–14). Though the enantioselectivity did not change at 0 °C, the enantioselectivity improved to 92% ee after running the reaction at −10 °C for 2 days. Under these conditions, the enantioselectivities of the major and minor diastereomers of intermediate A were found to be 92% and 88% ees, respectively. A one-pot reaction was also performed but the product 3a was obtained in lower enantioselectivity (90% yield and 83% ee).

**Table 1:** Catalyst and solvent optimization for azocine

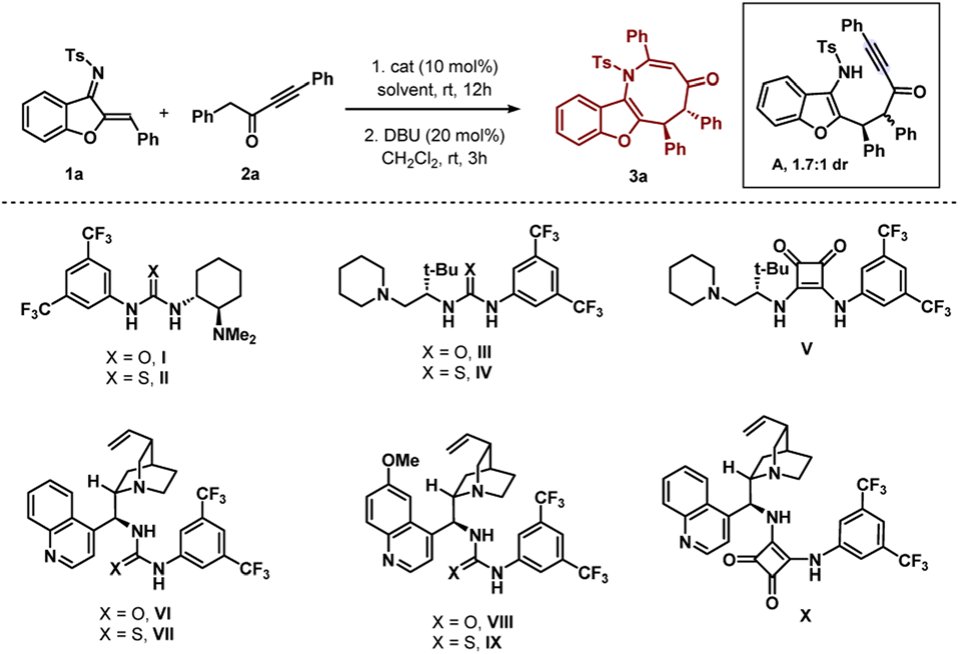					
Entry^a^	Catalyst	Solvent	Yield^b^	d.r^c^	ee^d^
1	I	Toluene	88	>20 : 1	84
2	II	Toluene	57	>20 : 1	33
3	III	Toluene	89	>20 : 1	80
4	IV	Toluene	90	>20 : 1	5
5	V	Toluene	90	>20 : 1	67
6	VI	Toluene	90	>20 : 1	88
7	VII	Toluene	30	>20 : 1	12
8	VIII	Toluene	92	>20 : 1	77
9	IX	Toluene	55	>20 : 1	47
10	X	Toluene	<5	n.d	n.d
11	VI	Mesitylene	91	>20 : 1	82
12	VI	*o*-Xylene	77	>20 : 1	82
13^e^	VI	Toluene	88	>20 : 1	88
14^f^	VI	Toluene	91	>20 : 1	92

a Unless otherwise mentioned, reactions were carried out with 0.1 mmol of 1a and 0.11 mmol of 2a in 1 ml solvent at rt.

b Isolated yield after silica gel column chromatography.

c Determined by ^1^H NMR.

d Determined by chiral HPLC.

e Reaction was run at 0 °C.

f Reaction was run at −10 °C and for 2d.

**Scheme 2:**
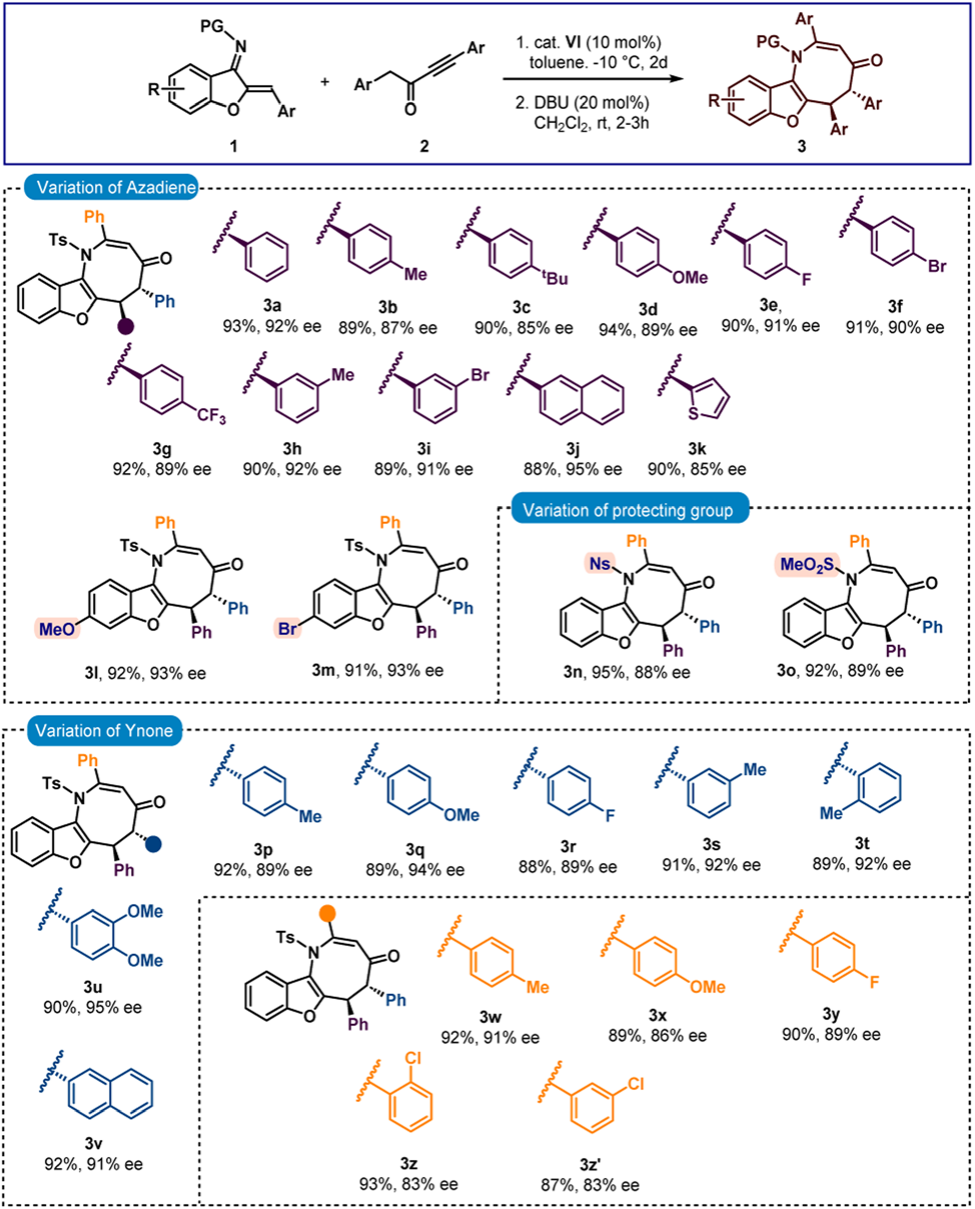
Scope of benzofuran fused azocines^*a*,*b*^. ^a^All reactions were carried out with 0.1 mmol of 1 and 0.11 mmol of 2 in 1 ml toluene at −10 °C for 2 days. Then, the isolated adduct was treated with DBU (20 mol%) in CH_2_Cl_2_ (1 ml) at rt for 2 h. ^b^Yields correspond to the isolated yields after silica gel column chromatography, dr was determined by ^1^H NMR and in all cases >20 : 1; ee was determined by HPLC.

After finalizing the optimal reaction conditions, we set out to determine the substrate range for this new [4 + 4] annulation. Initially, the scope of azadiene 1 was checked and gratifyingly good results were obtained. As shown in Scheme 2, high to excellent enantioselectivities and good to high yields were obtained for a variety of azadienes with substitutions in the *ortho*, *meta*, and *para* positions of the phenyl group. 4-Methyl and 4-methoxy substituted azadienes 1b and 1d provided products 3b and 3d in 87% and 89% ees, respectively. Slightly lower enantioselectivity was detected for product 3c having 4-^*t*^butyl substitution. Halo substitutions were also tolerated and high enantioselectivities were observed for products 3e and 3f having 4-fluoro and 4-bromo substitutions, respectively. A smooth conversion was also detected for compound 2g having 4-CF_3_ substitution and the desired product 3g was isolated in 92% yield with 89% ee. The reaction outcome did not change with meta-substituted aryl group containing azadienes 1h and 1i, and high enantioselectivities were attained for products 3h and 3i. 2-Naphthyl containing azadiene 1j also participated in the reaction to deliver product 3j in 95% ee. Finally, 2-thienyl containing azadiene 1k was engaged in the reaction and a good result was obtained for product 3k. Then substitutions in the benzofuran motif were examined, and pleasingly, methoxy- and bromo-substituted azadienes 1l and 1m reacted smoothly to provide products 3l and 3m, respectively, in 93% ee. Other groups, such as N-Ns and N–SO_2_Me groups, could be used in place of the N-Ts group; and high enantioselectivities were found for 3n and 3o. Then the scope of ynone 2 was examined and encouragingly positive outcomes were found (Scheme 2).

Initially the aryl group close to the carbonyl group was varied. Here also, different substitutions at the *ortho*-, *meta*- and *para*-positions were tolerated and excellent enantioselectivities were detected for the products 3p–3t. High enantioselectivity was detected for compound 3u having a 3,4-disubstituted aryl group. The reaction outcome did not change with 2-naphthyl substitution and product 3v was isolated in 92% yield with 91% ee. Then the substitutions on the aryl group attached to the triple bond in 2 were checked. To our delight, good results were observed for products 3w–3y having different *para*-substituted aryl groups. Slightly lower enantioselectivities were detected for products 3z and 3z′ having *ortho*- and *meta*-substitutions, respectively.

**Scheme 3:**
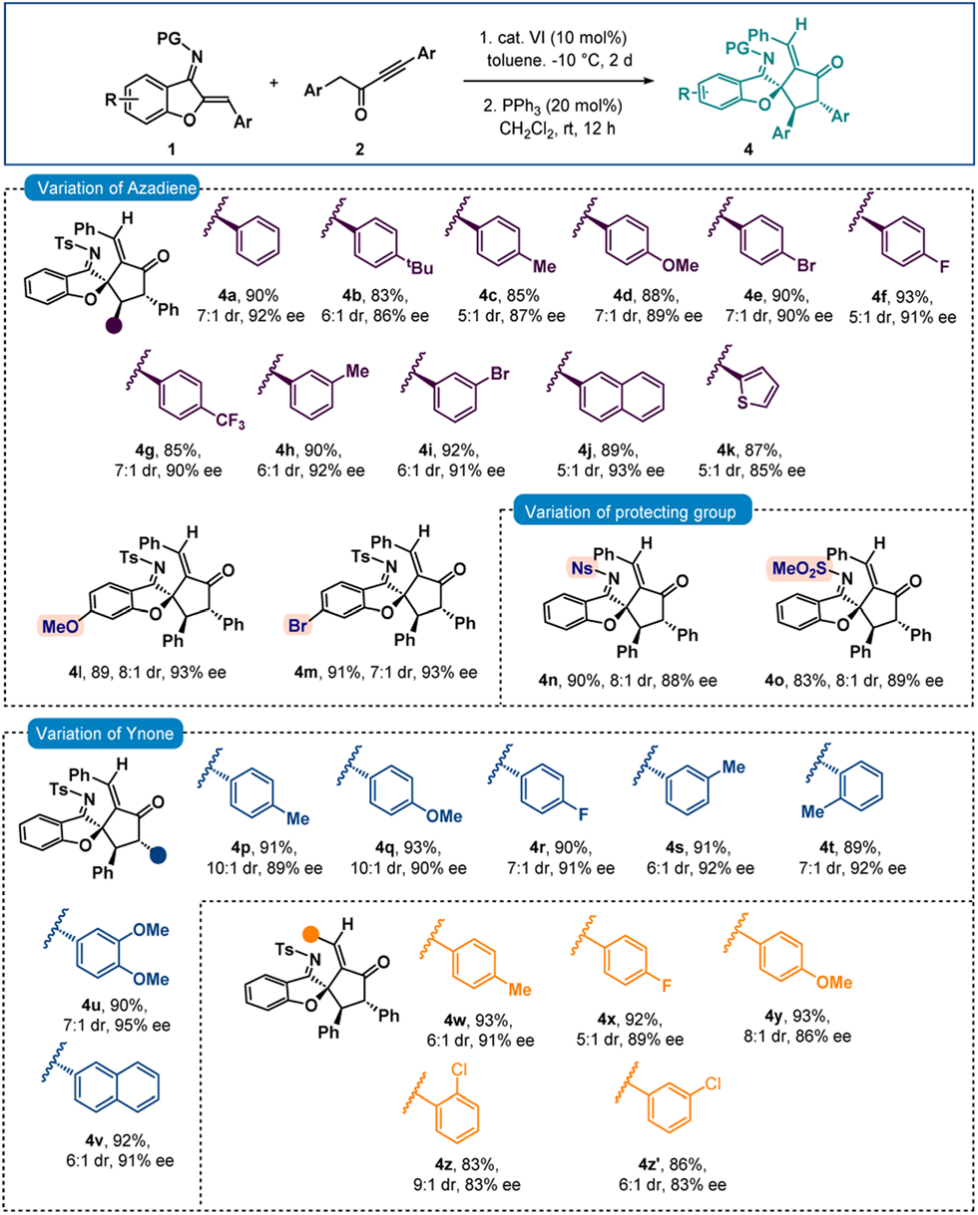
Scope of spiro-cyclopentanone benzofurans^*a*,*b*^. ^a^All reactions were carried out with 0.1 mmol of 1 and 0.11 mmol of 2 in 1 ml toluene at −10 °C for 2 days. Then, the isolated adduct was treated with PPh_3_ (20 mol%) in CH_2_Cl_2_ (1 ml) at rt for 12 h. ^b^Yields correspond to the isolated yields after silica gel column chromatography, dr was determined by ^1^H NMR, in all cases *E*/*Z* ratio >20 : 1 and ee was determined by HPLC.

Next, the chiral intermediate A (dr = 1.7 : 1) formed from the reaction of 1a and 2a was treated with 20 mol% triphenylphosphine in CH_2_Cl_2_. To our delight, after stirring for 12 hours, spiro-cyclopentanone benzofuran 4a with a stereogenic quaternary centre was isolated in 90% yield with 7 : 1 dr and 92% ee (Scheme 3). Here also epimerization was observed. A one-pot reaction was also performed but spiro-benzofuran 4a was isolated in 83% yield with 77% ee. The minor diastereomer originates from the creation of a stereogenic quaternary centre (see the ESI† for details). Then a variety of azadienes 1 were examined under sequential conditions, and encouragingly positive results were found. When several *para*-substitutions were first tested, the results for products 4b–4g were pleasingly high in terms of diastereoselectivities and excellent in terms of enantioselectivities (Scheme 3). The outcome did not change with *meta*-substitutions and the products 4h and 4i were obtained in 92% and 91% ees, respectively. Naphthyl and thienyl substitutions were also tolerated and good results were detected for products 4j and 4k. Then, the benzofuran motif's substitutions were looked at, and it was pleasing to see that the methoxy- and bromo-substituted azadienes 1l and 1m reacted smoothly to produce products 4l and 4m in 93% ee (Scheme 3). Next, other imine protective groups were examined, and it was discovered that N-Ns and N–SO_2_Me groups containing azadienes produced positive results for the products 4n–4o. Then the scope of ynone 2 was checked and here also excellent results were found with different aryl group variations incorporated at the α-position of the carbonyl group of 2. In fact, 89–92% ees were observed for products 4p–4v. Then different substitutions on the aryl group attached to the triple bond in 2 were checked and excellent results were achieved for products 4w–4y having different *para*-substitutions. The enantioselectivities slightly dropped with *ortho*- and *meta*-substitutions (both 4z and 4z′ in 83% ee).

**Scheme 4:**
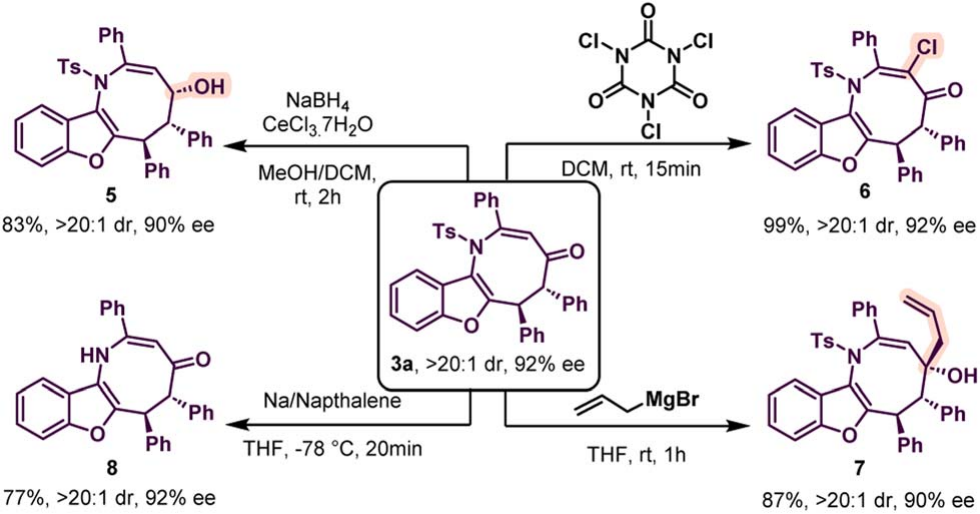
Synthetic transformations of azocine 3a.

The absolute configuration of compound 3f was determined to be (5*S*, 6*S*) by X-ray crystallography.^14^ Thus other azocine derivatives 3 are expected to have the same configuration. Similarly, the absolute configuration of 4k was found to be (4′*S*, 5′*S*) from X-ray crystallography.^15^ Thus, other benzofurans 4 will have the same configuration. Then, azocine 3a was subjected to a variety of organic transformations to further demonstrate the usefulness of our technique (Scheme 4). Initially, 3a was treated with sodium borohydride and cerous chloride to provide 5 with an alcohol group in high diastereoselectivity and the enantioselectivity was almost retained. The relative configuration of 5 was determined by 2D NMR spectroscopy. Then a chlorination reaction was performed with trichloroisocyanuric acid (TCCA). This resulted in the formation of 6 in 99% yield and both diastereo- and enantioselectivity were unchanged. Then, allylation of 3a was carried out with allyl magnesium bromide in THF. To our delight, the reaction proceeded smoothly to provide compound 7 as a single diastereomer with high enantioselectivity. Finally, deprotection of the *N*-tosyl group was performed and product 8 was formed without erosion in enantioselectivity.

**Scheme 5:**
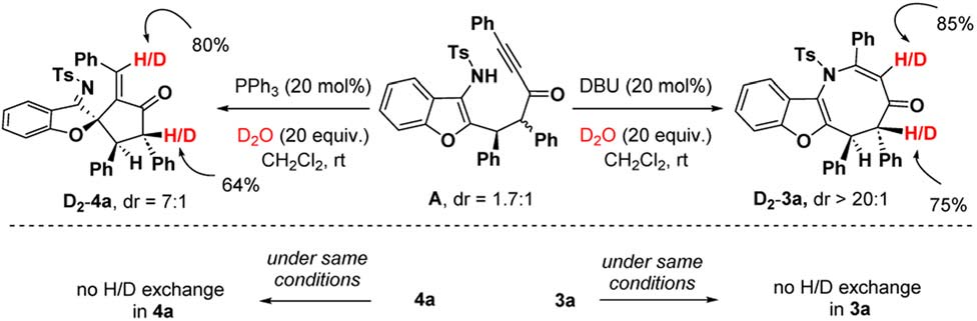
Deuterium exchange experiment.

To get insight into the mechanism, we carried out deuteration experiments with intermediate A (Scheme 5). At first, A (1.7 : 1 dr) was treated with DBU (20 mol%) and D_2_O (20 equiv.) in CH_2_Cl_2_ at room temperature. ^1^H nuclear magnetic resonance (NMR) analysis of 3a revealed 85% deuterium incorporation at the C3-position and 75% deuterium incorporation at the C5-position. When 3a was stirred under similar conditions, no H/D exchange was detected. Similarly, the deuteration reaction was performed with intermediate A, PPh_3_ (20 mol%) and D_2_O (20 equiv.), and in fact, 80% deuterium incorporation was found at the olefin carbon and 64% deuterium incorporation was detected at the C4′-position of 4a. No H/D exchange was noticed when 4a was agitated in the same manner. This suggests that in both cases, the C2-center of A underwent an epimerization to deliver the stereoisomers in favour of the thermodynamically more stable *trans*-isomer.

**Fig. 1:**
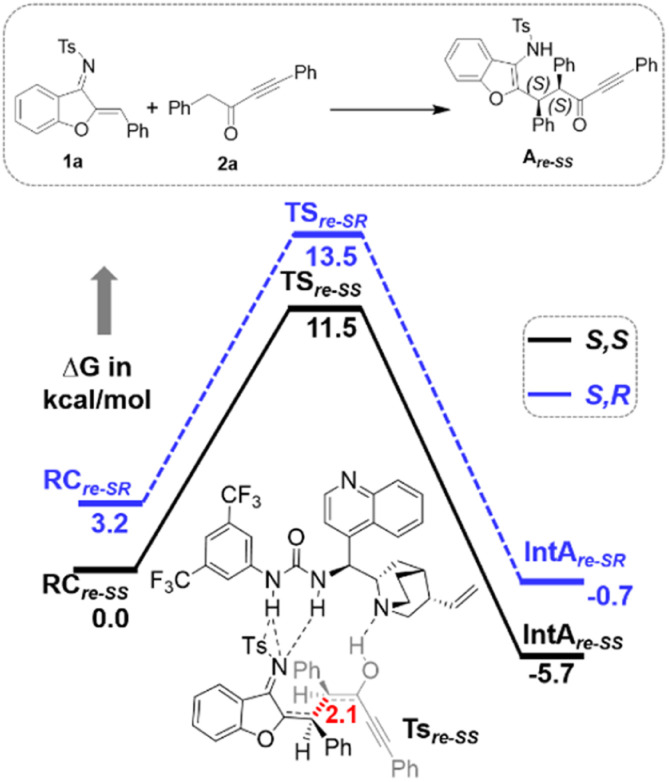
Gibbs free energy (kcal mol^−1^) profile at B3LYP-D3(BJ)/CPCM(toluene)/def2-TZVP for bifunctional urea catalyst VI mediated 1a and 2a coupling. Distances shown are in units of Å.

**Fig. 2:**
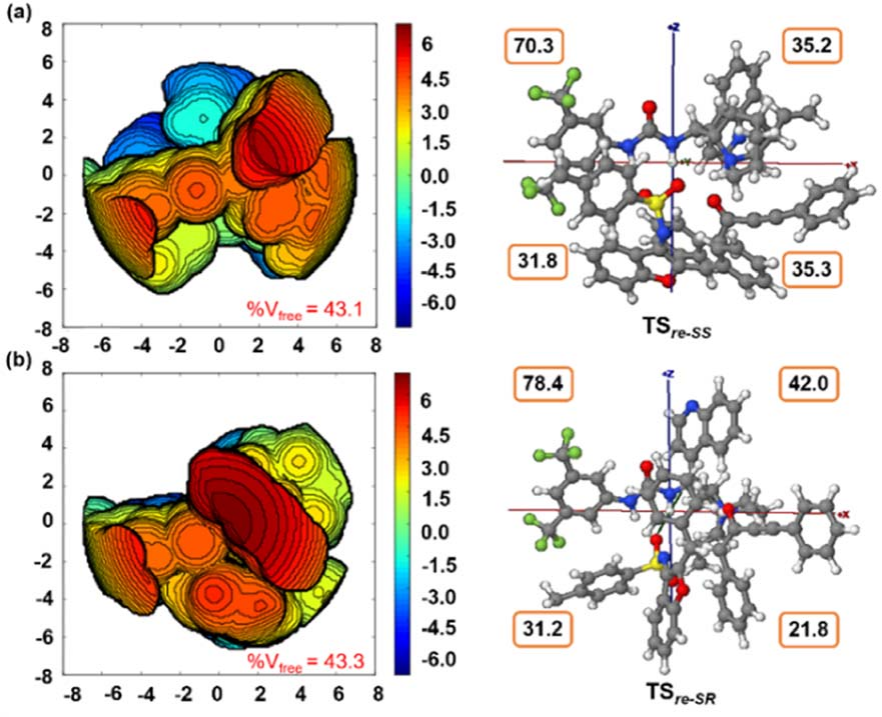
Steric map (within a range of ± 6.0 kcal mol^−1^) and 3D model with *xyz* axes (between ± 8 Å) for (a) TS_re-SS_ and (b) TS_re-SR_. Total % *V*_free_ and those in each quadrant along the *xyz* axes are shown. Contour color – blue: strong attractive; green: weak attractive; red: strong repulsive.

**Fig. 3:**
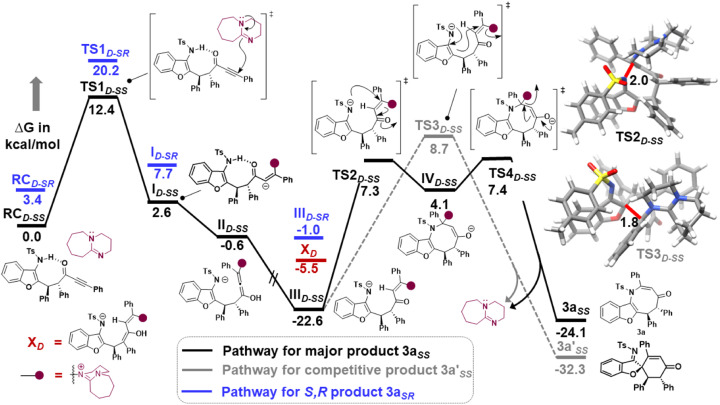
Gibbs free energy (kcal mol^−1^) profile at B3LYP-D3(BJ)/CPCM(CH_2_Cl_2_)/def2-TZVP for the DBU assisted intramolecular cyclization of intermediate A_re-SS_ and A_re-SR_. Distances shown are in units of Å. Color code: C(grey), H(white), N(blue), O(red), and S(yellow). D sub-script denotes DBU assisted.

**Fig. 4:**
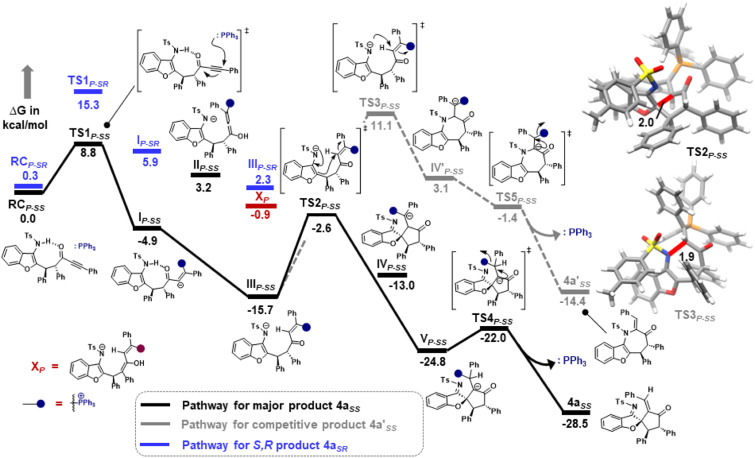
Gibbs free energy (kcal mol^−1^) profile at B3LYP-D3(BJ)/CPCM(CH_2_Cl_2_)/def2-TZVP for the PPh_3_ assisted intramolecular cyclization of intermediates A_re-SS_ and A_re-SR_. Distances shown are in units of Å. Color code: C(grey), H(white), N(blue), O(red), and S(yellow). P subscript denotes PPh_3_ assisted.

Furthermore, computational studies with the density functional theory (DFT) at B3LYP-D3(BJ)/def2-TZVP/C-PCM//PBE-D3(BJ)/def2-SVP^16^ were conducted in order to understand the origin of the enantioselectivity and divergence of the asymmetric product on switching of Lewis base catalysts. To ponder about the enantioselectivity of the azocine and spiro derivatives and probe the plausible catalytic activation modes, we chose the reaction between *N*-sulfonyl 1-azadiene, 1a, and ynone, 2a, in the presence of the cinchonidine derived bifunctional urea catalyst VI (Table 1, entry 6), and conducted an extensive computational study with both Re- and Si-face activation of the substrates (see the ESI†). The chiral reactant complex consisting of the substrates and the weakly bound catalyst in the Re-face (RC_re-SS_), resulting in the *S*,*S* configuration of the aurone-derived α,β-unsaturated imine (A_re-SS_, Fig. 1 inset), is significantly lower in energy as compared to the other adducts (see Fig. S2†). Indeed, RC_re-SS_ is highly exergonic (Δ*G* = −10.4 kcal mol^−1^) as compared to the separated reactants and the catalyst. Hence it has been chosen as the reference for the Gibbs free energy profile as shown in Fig. 1. The nucleophilic attack of azadiene 1a to the enol form of 2a along the Re-face through the two transition states, TS_re-SS_ and TS_re-SR_, resulting in major (*S*,*S*) and minor (*S*,*R*) enantiomers of the α,β-unsaturated imine intermediate A, is predicted at a Gibbs free energy barrier of 11.5 and 13.5 kcal mol^−1^ (Fig. 1), consistent with the experimental findings. Further refinement of energetics at the DLPNO-CCSD(T)/def2-TZVP/C-PCM//PBE-D3(BJ)/def2-SVP level of theory predicts the relative Δ*G*^‡^ at 9.2 and 13.3 kcal mol^−1^, respectively, which is in accordance with the stereochemical outcome. The lower activation energy requirement of TS_re-SS_ as compared to TS_re-SR_ is presumably due to extensive attractive non-covalent interactions such as π–π and H-bond interactions (see Fig. S6†).^17^ Furthermore, plots of the steric maps of TS_re-SS_ and TS_re-SR_ in Fig. 2 show that % *V*_free_, which accounts for the percentage of total free volume within the catalyst pocket, has similar values.^18^ However, a careful investigation reveals that % *V*_free_ in the south-east quadrant, where the C–C coupling between the two substrates takes place is significantly lower for TS_re-SR_ (21.8%) as compared to that for TS_re-SS_ (35.3%). A smaller value of % *V*_free_ indicates greater confinement and thereby more steric encumbrance. Hence the “open” structure within the catalytic pocket, with lowered steric congestion, along with the complimentary attractive non-covalent interactions results in TS_re-SS_, predicted to be always more stabilized than TS_re-SR_ (see the ESI†).^19^ Incidentally, in the absence of VI, the reaction between the enol form of 2a and 1a is estimated at high energetic expenses (see Fig. S1†), further emphasizing the crucial role of the H-bond networks offered by the chiral catalyst. The catalytic *Si*-face activation of the substrates is predicted at predominantly high barriers (see Fig. S2†) and rationalizes the significance of the Re-face approach for a favourable attack. Next, we explored the intramolecular cyclization of α,β-unsaturated imine intermediate A in the presence of the Lewis base catalyst, DBU (Fig. 3). Starting from the reactant complex RC_D-SS_ consisting of the major enantiomer A_re-SS_ together with DBU, we envisage a 1,4-addition of DBU towards the electrophilic alkyne carbon centre of A_re-SS_ via TS1_D-SS_ at an energetic expense of 12.4 kcal mol^−1^. Expectedly, a similar attack by DBU on A_re-SR_ via TS1_D-SR_ requires more than 7.0 kcal mol^−1^ free energy of activation, primarily due to the absence of an intramolecular H-bond unlike RC_D-SS_ and TS1_D-SS_, and the subsequent reaction, leading to the (*S*,*R*) product, 4_SR_ as shown in the ESI (see Fig. S3†). The resultant chiral α,β-unsaturated zwitterion, I_D-SS_, quickly reorganizes to the allene II_D-SS_ on N–H-shifting with a barrier-less rotation followed by an energetically favourable keto–enol tautomerism to the α,β-unsaturated keto intermediate, III_D-SS_, crucial for the emergence of the azocine and the spiro compounds. The trajectory for the formation of the eight membered cyclized azocine derivative 3a_SS_ proceeds through a stepwise intramolecular nucleophilic attack of the N-centre on the electrophilic β-sp^2^-C centre *via*TS2_D-SS_ and DBU release *via*TS4_D-SS_ (Fig. 3). In a similar way, the other diastereomer RC_D-SR_ reacts through I_D-SR_, II_D-SR_ and III_D-SR_ (see the ESI†) intermediates. The intermediate III_D-SR_ undergoes a rapid epimerization to generate the thermodynamically stable III_D-SS_ through intermediate XD, which facilitates generation of 3a with high diastereoselectivity. Alternatively, a six membered cyclized plausible product, 3a′_SS_, is hypothesized through a concerted transition state, TS3_D-SS_. The chemoselectivity for the observed 8 membered azocine product over the six-membered analogue is evident from ΔΔ*G* = 1.4 kcalmol^−1^. Indeed, the higher selectivity of the H-bond assisted C–N cyclization step over C–C cyclization is also supported at the DLPNO-CCSD(T)/def2-TZVP level of theory (ΔΔ*G*^‡^ = 4.1 kcal mol^−1^), nullifying the possibility of the C–C coupling analogue. In contrast, A_re-SS_ delivers a highly stable five membered spiro compound 4a_SS_ in the presence of PPh_3_ (Fig. 4).

The chiral reactant complexes, RC_P-SS_ and RC_P-SR_, consisting of the two diastereomers, A_re-SS_ and A_re-SR_, together with PPh_3_ are predicted to be equi-energetic (Fig. 4). Evidently, the higher stability of TS1_P-SS_ (Δ*G*^‡^ = 8.8 kcal mol^−1^) than TS1_P-SR_ (Δ*G*^‡^ = 15.3 kcal mol^−1^), during nucleophilic attack of PPh_3_ on the α, β-unsaturated imine, drives the reaction towards the S,S enantiomer preferentially. This results in stable I_P-SS_ reorganizing to an allene rotamer II_P-SS_, with a proton shift from the N-centre to the carbonyl O-atom along with the breakage of H-bonding. Subsequently, a free energetically and sterically stable keto–enol tautomer intermediate III_P-SS_ is formed which controls the selectivity in the reaction. The delocalisation of the anionic electrons present over the N-atom to the C1 atom leads to the development of an intrinsic nucleophilic centre adjacent to furan O. This leads to an intramolecular nucleophilic umpolung attack on the α-sp^2^ C-centre at an energetic expense of 13.1 kcal mol^−1^ (TS2_P-SS_), to furnish a chiral spiro intermediate IV_P-SS_. With a 1,2 H-shift, a stable V_P-SS_ is formed which subsequently releases PPh_3_ at an energy barrier of 2.8 kcal mol^−1^ (TS4_P-SS_) to generate the five membered chiral spiro compound, 4a_SS_. On the other hand, the minor diastereomer RC_P-SR_ delivers 4a with high diastereoselectivity *via* epimerization through intermediate X_P_. The study correlates an enol-tautomerization of III_P-SR_ to give energetically stable X_P_, which subsequently epimerized to the thermally stable III_P-SS_ to produce the targeted product 4a. The alternative pathway of intramolecular nucleophilic attack of N on the α-sp^2^ C-centre through TS3_P-SS_ is ruled out due to the large energy drift. Hence, the formation of the seven membered 4a′_SS_ is not feasible. This is supported by DLPNO-CCSD(T)/def2-TZVP energy barriers (ΔΔ*G*^‡^ = 12.5 kcal mol^−1^). Notably, DBU is found to be highly basic with a p*K*_a_ of 16.8 as compared to PPh_3_ having p*K*_a_ = 2.8.^20^ One may fathom an alternative pathway consisting of a deprotonative activation of the TsNH moiety by Brønsted basic action of DBU, that leads to intramolecular cyclization through C–N attack. Indeed, formation of the eight-membered ring containing product, 3a_SS_, takes place at a barrier of 19.4 kcal mol^−1^, while the six-membered ring containing C–C coupling TS leading to 3a′_SS_ has to overcome a kinetic expense of 23.5 kcal mol^−1^, further emphasizing the predominance of 3a_SS_ as the product. To confirm the Brønsted or Lewis base pathway, we have recorded the ESI-MS spectrum of the reaction mixture, and a *m*/*z* of 748.3209 corresponding to [M + H+] of intermediates I_D-ss_, II_D-ss_, and III_D-ss_ was found. This ruled out the alternate path where DBU functions as a Brønsted base (see the ESI† for details).

**Fig. 5:**
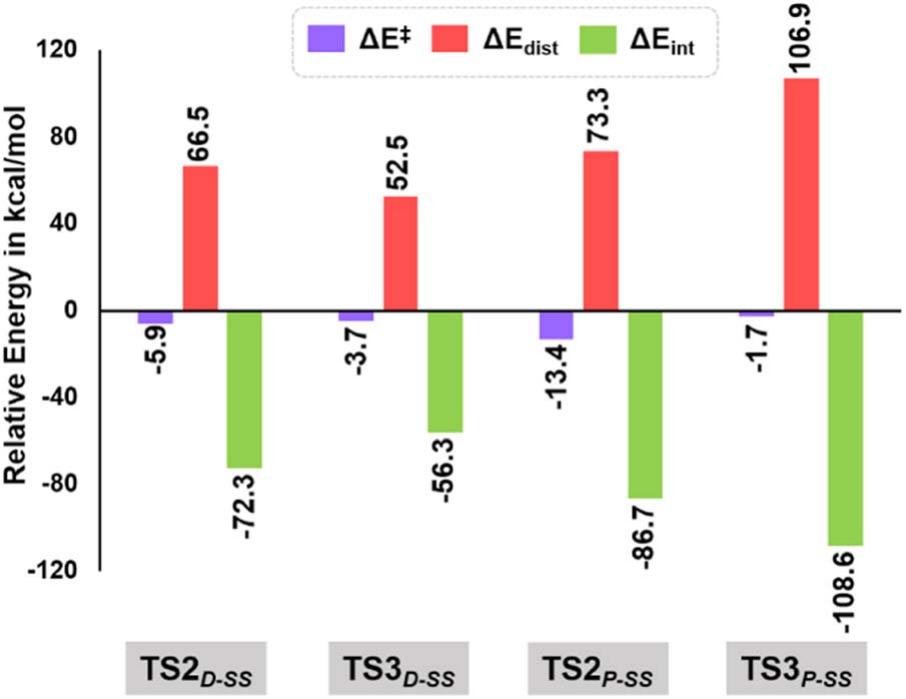
Energy decomposition analysis at B3LYP-D3(BJ)/CPCM(CH_2_Cl_2_)/def2-TZVP in units of kcal mol^−1^ for TS2_D-SS_, TS3_D-SS_, TS2_P-SS_ and TS3_P-SS_.

As evident from our computational studies,^21^ the cyclization processes in the presence of two different Lewis base catalysts turns out to be the rate determining steps (RDSs) and are responsible for the product selectivity, *i.e.*, predominance of 3a_SS_ over 3a′_SS_ and 4a_SS_ over 4a′_SS_. A careful investigation shows that TS2_D-SS_ leading to the eight-membered cyclic core in 3a_SS_ suffers from greater geometric distortion (ΔΔ*E*_dist_ = 13.9 kcal mol^−1^) as compared to TS3_D-SS_ leading to the six-membered moiety in 4′_SS_ (Fig. 5, see the ESI† for computational details). In contrast, the relative interaction energy (ΔΔ*E*_int_ = −16.0 kcal mol^−1^) suggests a better orbital overlap and favourable electronic interactions for TS2_D-SS_ and stabilizes its energetic requirement by 2.1 kcal mol^−1^. Interestingly, formation of the five-membered core in TS2_P-SS_ features greater π–π interactions coupled to lower steric encumbrance from the bulkier PPh_3_ additive that is reflected in their lower geometric distortion as compared to that of TS3_P-SS_ (ΔΔ*E*_dist_ = −33.6 kcal mol^−1^). This easily over shoots the unfavourable interaction energy (ΔΔ*E*_int_ = 21.9 kcal mol^−1^) and rationalises the observance of the 5-membered spiro compound, 4a_SS_.

In summary, we have developed a divergent pathway for the catalytic asymmetric synthesis of skeletally different benzofuran fused azocine derivatives and spiro-cyclopentanone benzofurans. The methodology involves sequential catalysis of a chiral bifunctional squaramide catalysed reaction between aurone-derived α,β-unsaturated imine and ynone followed by Lewis base catalyzed divergent annulation reactions. In both cases, epimerization leads to high diastereoselctivity. Few synthetic transformations have also been performed. Additionally, computational analysis has been performed to investigate the reaction mechanism and comprehend the chiral and Lewis base catalysts' control on the observed skeletal selectivity. Interestingly, London dispersive forces such as π–π and H-bond interactions are found to be crucial to overthrow the steric encumbrance for the stereochemical selectivity and achieve skeletal diversity. Given the significant medicinal value of azocines and spiro-cyclopentanone benzofurans, the pharmaceutical sector may find our procedure valuable.

## Data availability

All the experimental and computational data are available in ESI.†

## Author contributions

RK and SCP designed the experiment. RK performed the experiments. SKM and LR done the DFT study of the reactions.

## Conflicts of interest

There are no conflicts to declare.

## Supplementary Material

SC-014-D3SC03239F-s001

SC-014-D3SC03239F-s002

## References

[cit1] Doerksen R. S., Meyer C. C., Krische M. J. (2019). Angew. Chem., Int. Ed..

[cit2] Miller L. C., Sarpong R. (2011). Chem. Soc. Rev..

[cit3] Brandau S., Maerten E., Jørgensen K. A. (2006). J. Am. Chem. Soc..

[cit4] Masson-Makdissi J., Prieto L., Abel-Snape X., Lautens M. (2021). Angew. Chem., Int. Ed..

[cit5] Chen J., Jia P., Huang Y. (2018). Org. Lett..

[cit6] Nakamura I., Sato Y., Takeda K., Terada M. (2014). Chem.–Eur. J..

[cit7] Zheng W., Yao W., Ullah N., Lu Y. (2017). Angew. Chem., Int. Ed..

[cit8] Rong Z.-Q., Wang M., E Chow C. H., Zhao Y. (2016). Chem.–Eur. J..

[cit9] Wang L., Zhu H., Peng T., Yang D. (2021). Org. Biomol. Chem..

[cit10] Chen P., Wang J., Liu K., Li C. (2008). J. Org. Chem..

[cit11] Liu X., Wang K., Liu Y., Li C. (2021). Chem.–Eur. J..

[cit12] Carder H. M., Wang Y., Wendlandt A. E. (2022). J. Am. Chem. Soc..

[cit13] Connon S. J. (2008). Chem. Commun..

[cit14] CCDC 2235079 contains the crystallographic data for compound 3f

[cit15] CCDC 2235267 contains the crystallographic data for compound 4k

[cit16] Gatzenmeier T., Turberg M., Yepes D., Xie Y., Neese F., Bistoni G., List B. (2018). J. Am. Chem. Soc..

[cit17] Arun V., Roy L., De Sarkar S. (2020). Chem.–Eur. J..

[cit18] Harden I., Neese F., Bistoni G. (2022). Chem. Sci..

[cit19] Huffman B. J., Chu T., Hanaki Y., Wong J. J., Chen S., Houk K. N., Shenvi R. A. (2022). Angew. Chem., Int. Ed..

[cit20] Roy L. (2020). Int. J. Quantum Chem..

[cit21] Baidya M., Maiti D., Roy L., De Sarkar S. (2022). Angew. Chem., Int. Ed..

