# Correction: Structurally divergent enantioselective synthesis of benzofuran fused azocine derivatives and spiro-cyclopentanone benzofurans enabled by sequential catalysis

**DOI:** 10.1039/d3sc90206d

**Published:** 2023-10-20

**Authors:** Rupkumar Khuntia, Sanat Kumar Mahapatra, Lisa Roy, Subhas Chandra Pan

**Affiliations:** a Department of Chemistry, Indian Institute of Technology Guwahati Assam 781039 India span@iitg.ac.in https://www.iitg.ac.in/span/; b Institute of Chemical Technology Mumbai IOC Odisha Campus Bhubaneswar Bhubaneswar 751013 India

## Abstract

Correction for ‘Structurally divergent enantioselective synthesis of benzofuran fused azocine derivatives and spiro-cyclopentanone benzofurans enabled by sequential catalysis’ by Rupkumar Khuntia *et al.*, *Chem. Sci.*, 2023, **14**, 10768–10776, https://doi.org/10.1039/D3SC03239F.

The authors regret that some relevant citations to previous work on annulation reactions with enynes were not included in the original reference list of the published article. Ref. 12 in the article should be corrected as shown in ref. 1 below.

The Royal Society of Chemistry apologises for these errors and any consequent inconvenience to authors and readers.

## Supplementary Material

## References

[cit1] Ramachary D. B., Venkaiah C., Krishna P. M. (2012). Chem. Commun..

