# Supplementary material for: Structurally divergent enantioselective synthesis of benzofuran fused azocine derivatives and spiro-cyclopentanone benzofurans enabled by sequential catalysis
Source: Chem Sci. 2023 Sep 7;14(39):10768–76. doi: 10.1039/d3sc03239f (PMC10566461; doi:10.1039/d3sc03239f)
Supplement: SC-014-D3SC03239F-s001 [file SC-014-D3SC03239F-s001.pdf]

Electronic Supplementary Material (ESI) for Chemical Science.
This journal is © The Royal Society of Chemistry 2023

Supporting Information

Structurally Divergent Enantioselective Synthesis of Benzofuran Fused Azocine Derivatives and Spiro-Cyclopentanone Benzofurans Enabled by Sequential Catalysis

Rupkumar Khuntia[a] Sanat Kumar Mahapatra[b] Lisa Roy[b] and Subhas Chandra Pan[*a]*

[a]Indian Institute of Technology Guwahati Assam, 781039, India,

[b]Institute of Chemical Technology Mumbai IOC Odisha Campus Bhubaneswar Bhubaneswar 751013, India

E-mail: span@iitg.ac.in

Table of Contents

1. General Information………………………………………………………… *S1*
2. Preparation of Starting materials and Catalysts…………………………….. *S1-S5*
3. Optimization Study………………………………………………………… *S5-S7*
4. General Procedure for Synthesis of chiral azocines derivatives……………. *S7*
5. Characterization data of azocines derivatives………………………………. *S8-S21*
6. General Procedure for Synthesis of chiral Spiro-Cyclopentane Benzofurans... *S21*
7. Characterization of Spiro-Cyclopentane Benzofuran derivatives………….. *S22-S36*
8. Synthetic transformation of **3a**………………………………………………. *S36-S39*
9. Single crystal X-ray diffraction analysis…………………………………….. *S39-S41*
10. Mechanistic Study………………………………………………………… *S42-S45*
11. Computational details……………………………………………………... *S46-S178*
12. References………………………………………………………………. *S179*
13. NMR Spectra & HPLC Chromatogram……………………………………… *S180-S304*

1. General Information:

All dry solvents were dried using activated 4Å molecular sieves and stored under argon. For thin layer chromatography (TLC), silica gel plates with fluorescence indicator 254 nm were used and compounds were visualized by irradiation with UV light and/or by I_2. Celite® 512 medium was used for filtrations. Flash column chromatography was performed using 100-200 or 230-400 mesh silica gel. Petroleum ether and ethyl acetate for flash chromatography were acquired from commercial sources and were used without purification. NMR spectra were acquired on a Bruker 400 MHz, 500 MHz and 600 MHz spectrometer. Chemical shifts (δ) are reported in ppm relative to residual solvent signals ($CDCl_3$, 7.26 ppm for ^1H NMR and 77.23 ppm for ^{13}C NMR respectively. ^{13}C spectra were acquired on a broad band decoupled mode. For ^1H-NMR, data are reported as follows: chemical shift, multiplicity (s = singlet, d = doublet, dd = double doublet, ddd = doublet of doublet of doublets, t = triplet, q = quartet, dt = doublet of triplets, m = multiplet), coupling constants (Hz) and integration. Using ESI mode HRMS spectra were recorded. Enantiomeric ratios were determined by HPLC analysis performed on Chiral Columns using a Daicel Chiralpak IA, ID and IE Column.

2. Preparation of Starting materials and Catalysts:

General Procedure for 1-Azadiene Synthesis:

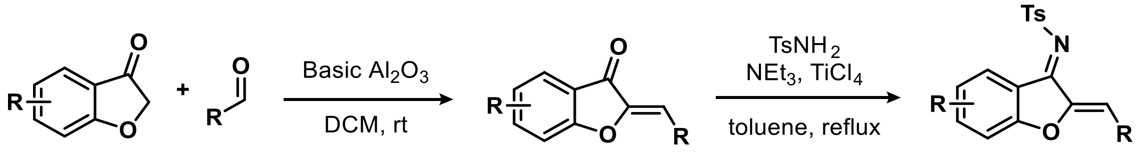

In a round bottom flask, charged with corresponding benzofuran-3(2*H*)-one (7 mmol, 1.0 equiv.) was dissolved in CH_2Cl_2 (30 mL). Then, the corresponding aldehyde (8.4 mmol, 1.2 equiv.) and Al_2O_3 (activated basic, 10 equiv.) were sequentially added and the reaction mixture was stirred for 5 hours at room temperature. After that, the reaction mixture was filtered through Celite® and solvents were removed in vacuo. Then purified by flash chromatography (petroleum ether : ethyl acetate = 98:2) to give aurones (**S1**).[1]

In a two-neck round bottom flask, corresponding aurones (3 mmol) and *p*-toluenesulfonamide (4.5 mmol) were taken and purged with argon 3 times. Then, toluene (30 mL) was added and cooled to 0 °C. Triethylamine (0.9 mL, 6 mmol, 2.0 equiv.) and $TiCl_4$ (1.0M in toluene, 3.0 mL, 3 mmol, 1.0 equiv.) were added dropwise sequentially at the same temperature. The reaction mixture was stirred overnight at reflux for overnight. After cooled to room temperature, the reaction mixture was quenched by 100ml water. Then diluted with DCM, washed with brine (3 x 30 mL), dried over Na_2SO_4, filtered and the solvent was evaporated in

vacuo. The mixture was purified by flash column chromatography (petroleum ether : ethyl acetate = 98:2) to afford 1-azadiene (**1a-o**). Na_2SO_4, filtered and the solvent was evaporated in vacuo. The mixture was purified by flash column chromatography (petroleum ether : ethyl acetate = 98:2) to afford 1-azadiene (**1a-o**).[2]

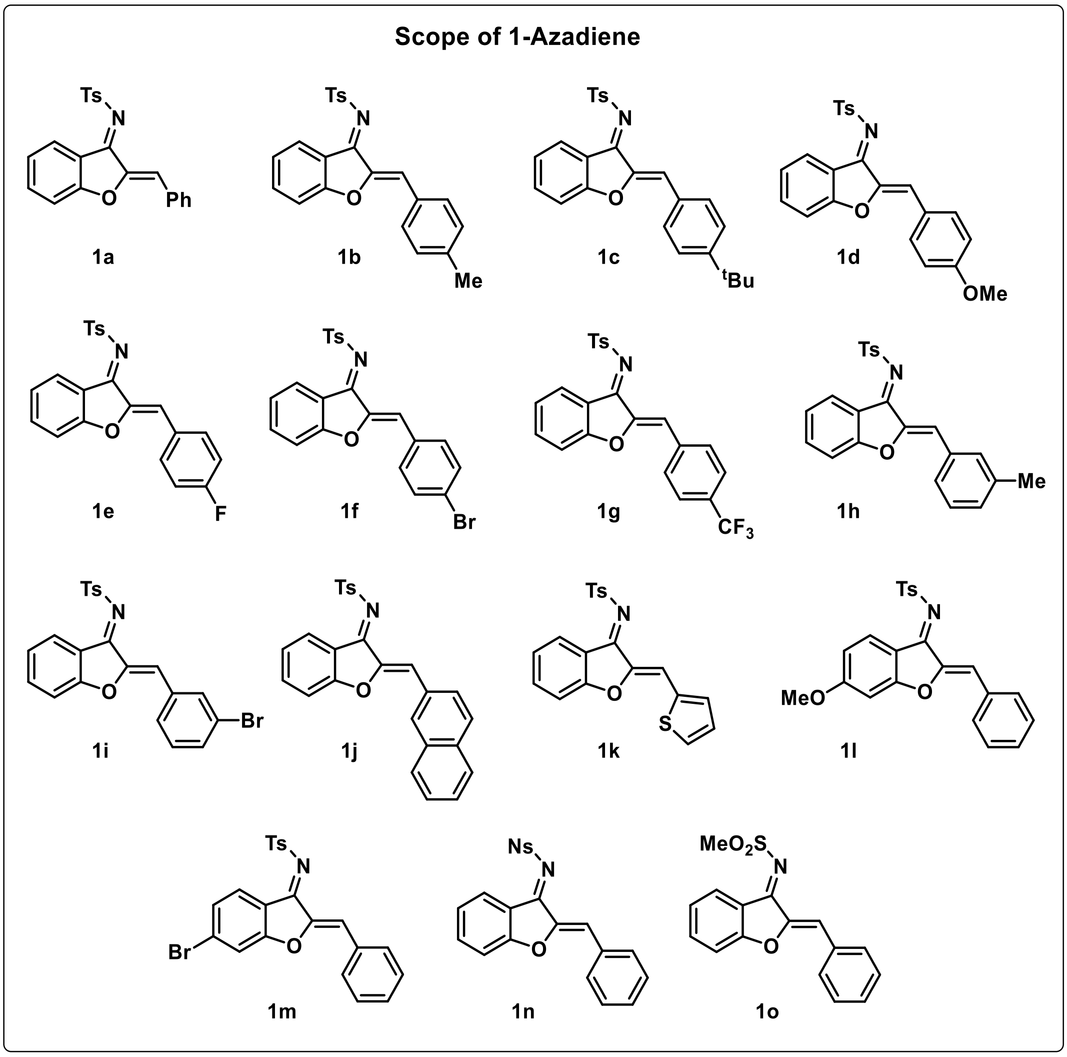

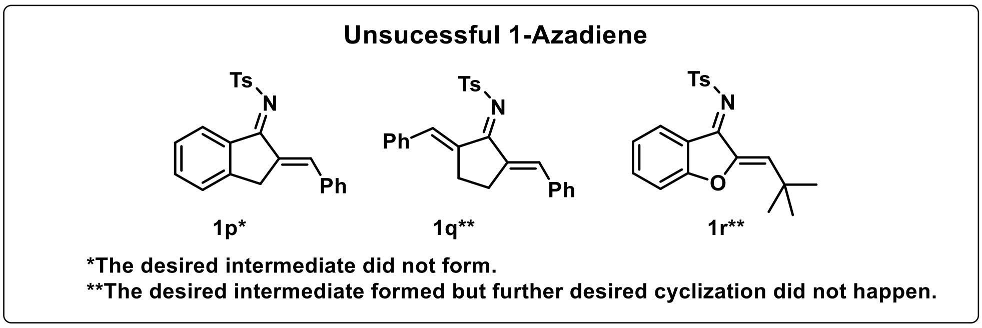

General Procedure for Ynones Synthesis:

Mathod A:

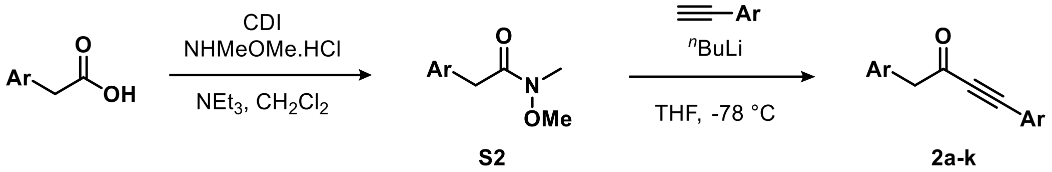

A 50-mL round-bottom flask was charged with aryl acetic acid (1.5mmol, 1.0 equiv) and 20 ml DCM, then carbonyldiimidazole (CDI) (1.8 mmol, 1.2 equiv) was added portion wise at 0℃. After the solution was stirred at room temperature for 1 h. The mixture again cooled to 0℃ and *N*,*O*-dimethylhydroxylamine (1.5 equiv) was added portion wise. Then the solution was stirred for another 12 h at room temperature. After completion 20 ml Water was added to the resulting solution and the aqueous layer was extracted with DCM (3 × 10ml). The combined organic layers were dried over Na_2SO_4. After concentrated under vacuum, the resulting Weinreb amide (**S2**) was used without further purification.[3]

Under argon atmosphere, an oven dried 100 ml round-bottom flask was charged with acetylene derivatives (3 mmol, 2.0 equiv.) in tetrahydrofuran (30 ml). To this solution at -78 ºC was added *n*-butyllithium (2 M in cyclohexane, 1.5ml, 3 mmol, 2 equiv.) dropwise. The resulting mixture was stirred at -78 ºC for 2 h. After that, a solution of weinreb amide in tetrahydrofuran (5 ml) was added over 30 minutes. The solution was stirred for overnight. After completion, aq. NH_4Cl was added. The mixture was diluted with diethyl ether was added and layers were separated. The aqueous layer was extracted with diethyl ether (3 × 15 ml) and the combined organic layers were washed with brine. After drying over Na_2SO_4 and concentrated in vacuo. The crude residue was purified by flash chromatography (petroleum ether : ethyl acetate = 98:2) to give the product (**2a-2k**).[4]

Mathod B:

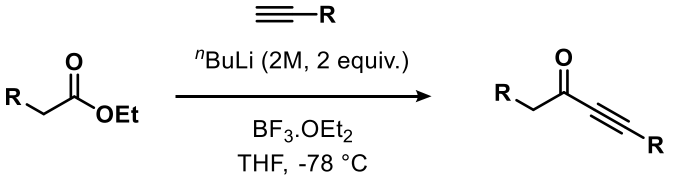

Ethynylbenzene (8.0 mmol) was dissolved into THF (8 mL), and the solution was cooled to -78 ºC. To the solution, n-Buli (8mmol, 2M cyclohexane) was added. After being stirred for 1h at -78 ºC, ethyl propionate (4.0 mmol) and $BF_3.OEt_2$ (9.6 mmol) were added successively. Then

stirred the mixture for 12-24h at -78 °C. The reaction was quenched by sat. aq. NH_4Cl, and extracted three times with EtOAc. The combined organic layer was dried over Na_2SO_4, and the solvent was removed under a reduced pressure. The residue was purified by flash column chromatography (petroleum ether : ethyl acetate = 98:2) to give the products (**2l-2m**).[5]

Scope of Ynone

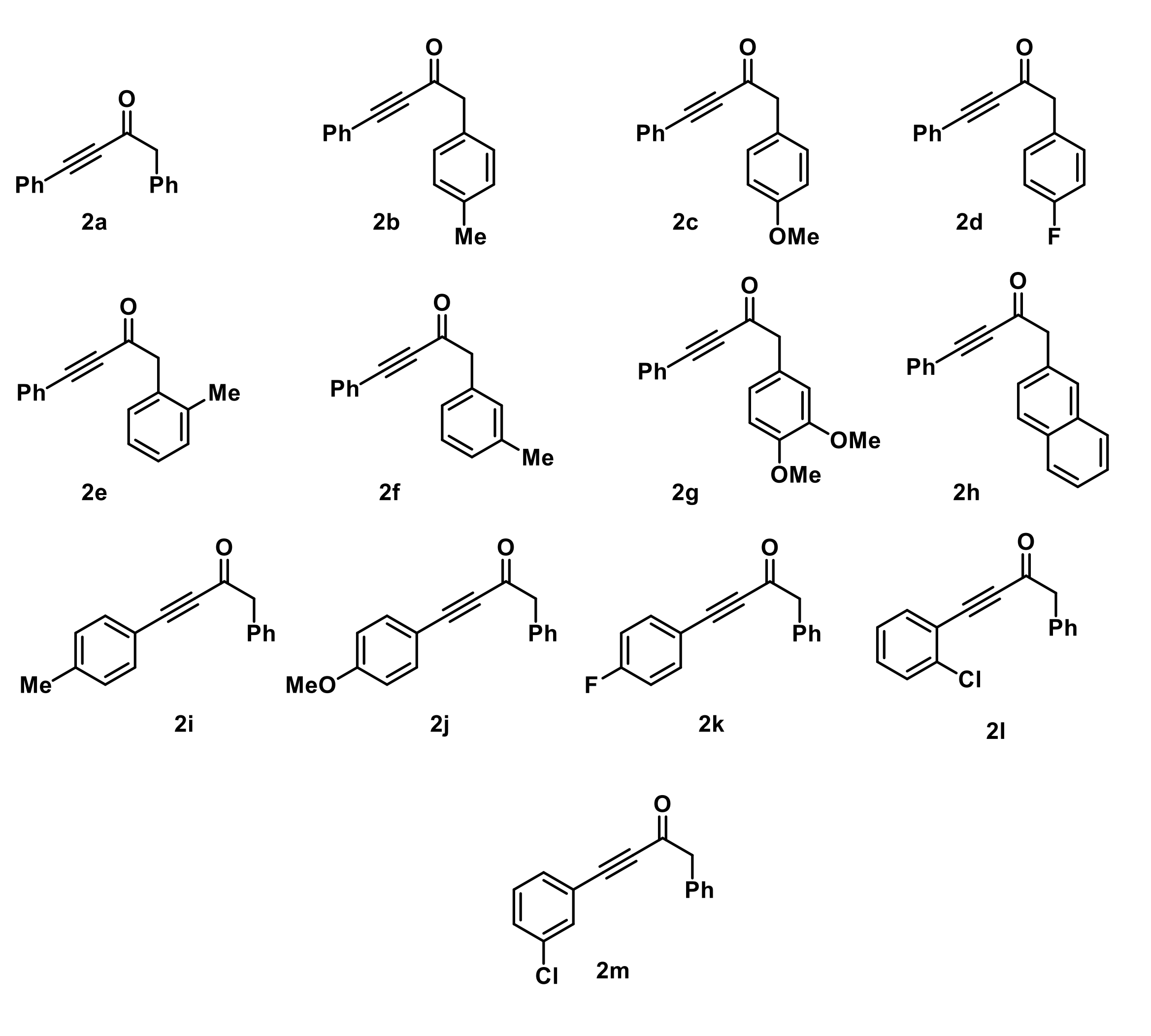

Unsucessfull Ynones

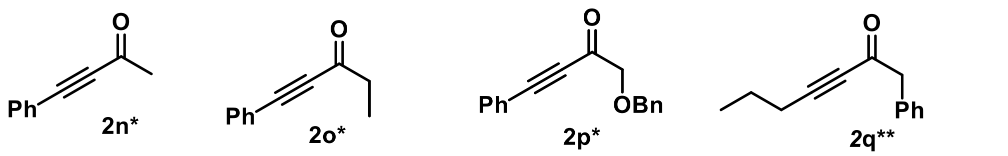

*The desired intermediate did not form.

**The desired intermediate formed but further desired cyclization did not happen.

Catalyst Preparation:

Catalyst **I**, **II**, **III**, **IV**, **V**, **VI**, **VII**, **VIII**, **IX** and **X** were prepared from available literature.[6,7]

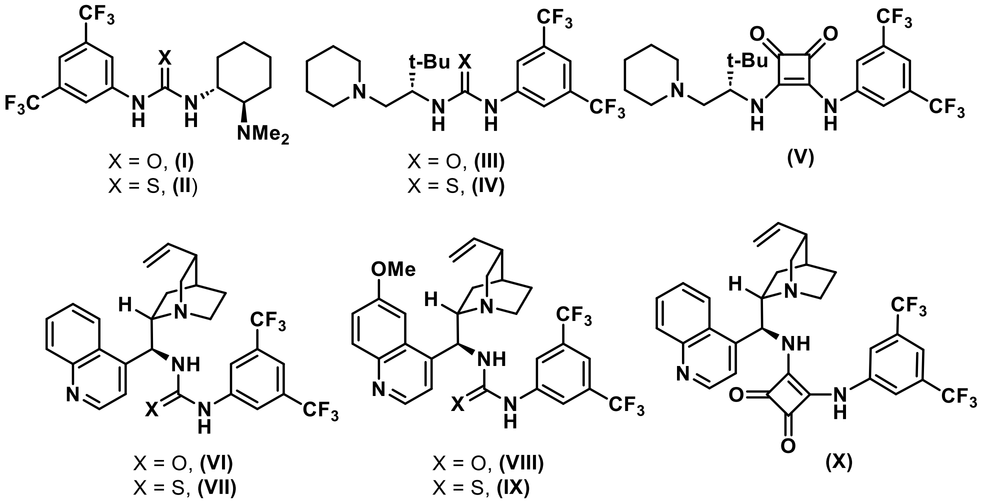

3. Optimization Study:

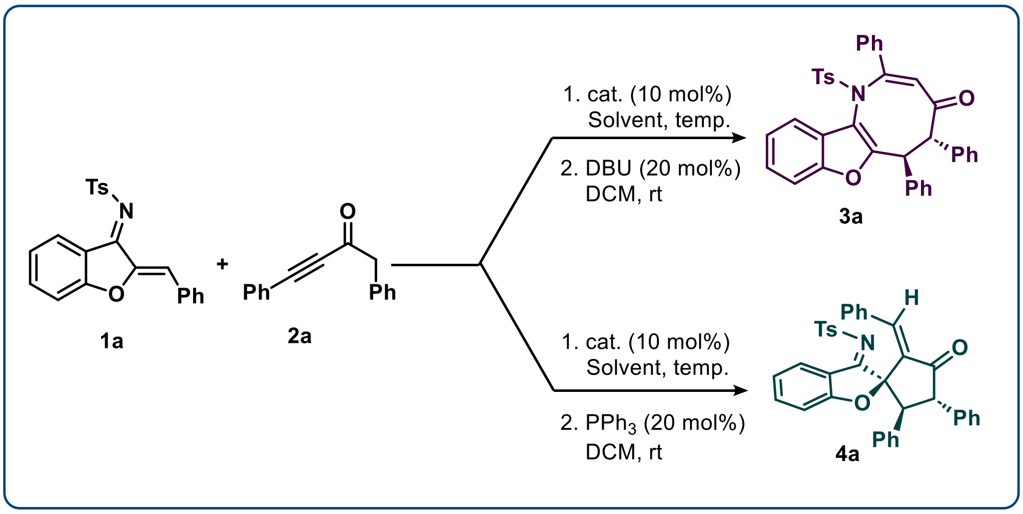

Table S1. ***Optimization of catalysts and Solvents for chiral azocine:***

Entry[a]	Catalyst	Solvent	Yield(3a)[b]	d.r(3a)[c]	*ee*(3a)[d]
1	**I**	toluene	88	>20:1	84
2	**II**	toluene	57	>20:1	33
3	**III**	toluene	89	>20:1	80
4	**IV**	toluene	90	>20:1	5
5	**V**	toluene	90	>20:1	67
6	**VI**	toluene	90	>20:1	88

7	**VII**	toluene	30	>20:1	12
8	**VIII**	toluene	92	>20:1	77
9	**IX**	toluene	55	>20:1	47
10	**X**	toluene	<5	N.D	N.D
11	**VI**	mesitylene	91	>20:1	82
12	**VI**	*o*-xylene	77	>20:1	82
13	**VI**	DCM	89	>20:1	79
14	**VI**	DCE	88	>20:1	83
15	**VI**	MTBE	89	>20:1	76
16[e]	**VI**	toluene	88	>20:1	88
17[f]	**VI**	toluene	91	>20:1	92
18[g]	**VI**	toluene	<5	N.D	N.D

[a]Reactions were carried out with 0.1 mmol of **1a** with 0.11 mmol of **2a** in 1 ml solvent at rt. Then, the isolated intermediate **A** was treated with DBU (0.02 mmol) in DCM (1 mL) at rt for 3 h. [b] Isolated yield after silica gel column chromatography. [c] Determined by ^{1}H NMR. [d]Determined by chiral HPLC. [e]Reaction was run at 0 °C. [f]Reaction was run at -10 °C and for 2d. [g] Reaction was run at -20 °C for 72 hr.

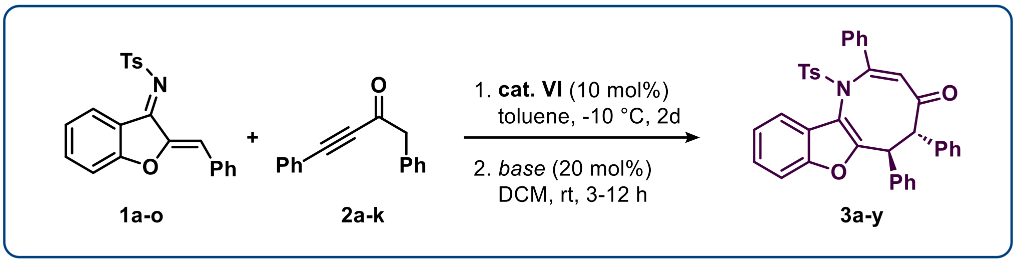

Table S2. ***Optimization of bases for azocines synthesis:***

Entry[a]	Base	Yield(3a)[b]	d.r(3a)[c]	*ee*(3a)[d]
1	DBU	91	>20:1	92
2	DABCO	N.D.	-	-
3	DMAP	N.D.	-	-
4[e]	DBU	95	>20:1	83%

[a]Reactions were carried out with 0.1 mmol of **1a** with 0.11 mmol of **2a** in 1 ml solvent at rt. Then, the isolated intermediate **A** was treated with *base* (0.02 mmol) in DCM (1 mL) at rt for 3-12 h. [b]Isolated yield after silica gel column chromatography. [c]Determined by ^{1}H NMR. [d]Determined by chiral HPLC. [e]Reactions were carried out with 0.1 mmol of **1a** with 0.11 mmol of **2a** in 1 ml solvent at rt. After consumption of starting material DBU (0.02 mmol) was added to the solution and stirred for 3 hr.

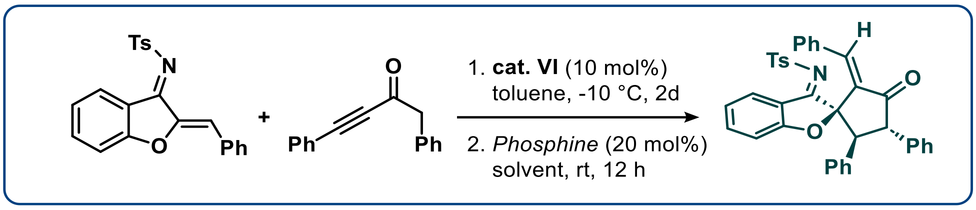

Table S3. ***Optimization of Phosphines for Spiro-Cyclopentane Benzofurans synthesis*:**

Entry[a]	Phosphine	Yield(4a)[b]	d.r(4a)[c]	*ee*(4a)[d]
1	PPh_3	90	7:1	92
2	PCy_3	53	5:1	92
3	$EtPPh_2$	77	7:1	92
4[e]	PPh_3	mixture	N.D.	N.D.
4[f]	PPh_3	83%	7:1	77%

[a]Reactions were carried out with 0.1 mmol of **1a** with 0.11 mmol of **2a** in 1 ml solvent at rt. Then, the isolated intermediate **A** was treated with phosphines (0.02 mmol) in DCM (1 mL) at rt for 12 h. [b]Isolated yield after silica gel column chromatography. [c]Determined by ^{1}H NMR. [d] Determined by chiral HPLC. [e]the isolated intermediate **A** was treated with PPh_3 (0.02 mmol), AcOH (0.04 mmol) in toluene (1 mL) at 75 °C for 8 h. [f]Reactions were carried out with 0.1 mmol of **1a** with 0.11 mmol of **2a** in 1 ml solvent at rt. After consumption of starting material DBU (0.02 mmol) was added to the solution and stirred for 12 hr.

4. General Procedure for the Synthesis of chiral azocines derivatives:

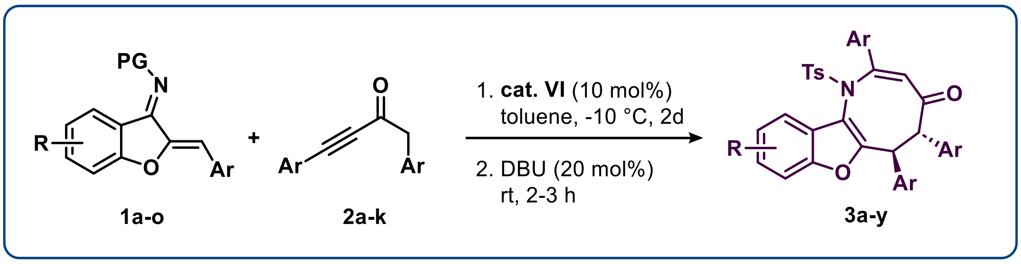

To a stirred solution of 1-azadienes **1a** (0.1 mmol) and ynones **2a** (0.11 mmol) in dry toluene (1 mL) at -10 °C, were added catalyst **VI** 10 mol%, and the reaction was allowed to run at the same temperature for 2 days. After full consumption of starting materials, solvents were evaporated and the reaction mixture was subjected to a short column chromatography (petroleum ether : ethyl acetate = 95:5) to afford intermediate **A.** Then the intermediate **A** dissolved in 1ml DCM, and DBU (20 mol%) was added subsequently. The reaction mixture was stirred at room temperature until the complete conversion of intermediate **A** was detected. The solvents were removed under reduced pressure and purified by flash column chromatography (petroleum ether : ethyl acetate = 95:5) to give azocines (**3a-z'**).

5. Charecterization data of azocines derivatives:

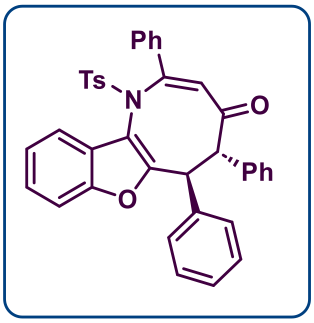

(5*S*,6*S*,*Z*)-2,5,6-triphenyl-1-tosyl-5,6-dihydrobenzofuro[3,2-b]azocin-4(1*H*)-one(3a): White solid, 55.4 mg, 93% yield, >20:1 *dr*, 92% *ee*; R_f = 0.5 (petroleum ether/ethyl acetate = 95:5)

^{1}H NMR (400 MHz, Chloroform-d) δ 7.58 (d, J = 7.3 Hz, 2H), 7.55 – 7.52 (m, 3H), 7.50 (s, 1H), 7.48 – 7.43 (m, 1H), 7.38 (d, J = 8.2 Hz, 1H), 7.33 (t, J = 7.6 Hz, 2H), 7.29 – 7.23 (m, 2H), 7.21 (d, J = 7.8 Hz, 2H), 7.17 – 7.13 (m, 5H), 7.09 (d, J = 7.3 Hz, 1H), 7.07 – 7.03 (m, 3H), 6.05 (d, J = 13.1 Hz, 1H), 5.92 (s, 1H), 4.88 (d, J = 13.1 Hz, 1H), 2.42 (s, 3H).**^{13}C NMR** (126 MHz, Chloroform-d) δ 201.13, 158.44, 154.88, 152.64, 144.85, 137.64, 136.98, 136.57, 136.21, 130.36, 130.22, 130.04, 129.95, 129.35, 129.11, 128.53, 128.35, 128.26, 127.51, 127.34, 126.88, 126.26, 125.31, 123.23, 120.54, 119.29, 112.24, 54.23, 49.51, 21.85.

HRMS (ESI$^+$) *m/z*: $[M+H]^+$ calculated for $C_{38}H_{29}NO_4S$: 596.1890, found: 596.1893;

HPLC: The enantiomeric excess was determined using CHIRALPAK ID column (*n*-Hexane/ *i*-PrOH=90:10, flow rate=1.0 mL/min, λ= 220 nm, τ_{major} = 22.6 min, τ_{minor} = 36.0 min).

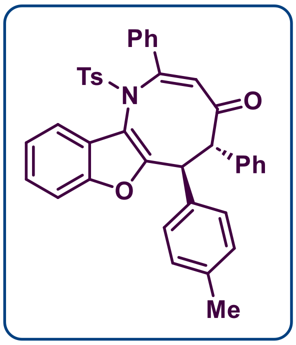

(5*S*,6*S*,*Z*)-2,5-diphenyl-6-(*p*-tolyl)-1-tosyl-5,6-dihydrobenzofuro [3,2-b]azocin-4(1*H*)-one (3b): White solid, 54.2 mg, 89% yield, >20:1 *dr*, 87% *ee*; R_f = 0.5 (petroleum ether/ethyl acetate = 95:5).

^{1}H NMR (500 MHz, Chloroform-d) δ 7.54 (t, J = 8.0 Hz, 4H), 7.49 – 7.44 (m, 3H), 7.38 (d, J = 8.3 Hz, 1H), 7.34 (t, J = 7.6 Hz, 2H), 7.28 – 7.23 (m, 4H), 7.18 – 7.13 (m, 4H), 7.06 – 7.04 (m, 3H), 6.99 (d, J = 7.8 Hz, 2H), 6.07 (d, J = 13.1 Hz, 1H), 5.92 (s, 1H), 4.88 (d, J = 13.1 Hz, 1H), 2.42 (s, 3H), 2.20 (s, 3H). **13C NMR** (126 MHz, Chloroform-d) δ 201.24, 158.69, 154.89, 152.59, 144.80, 136.98, 136.83, 136.53, 136.33, 134.49, 130.33, 130.18, 129.93, 129.84, 129.32, 129.10, 129.07, 128.51, 128.26, 127.46, 126.79, 126.27, 125.21, 123.18, 120.34, 119.21, 112.23, 54.22, 48.93, 21.84, 21.26.

HRMS (ESI$^+$) *m/z*: $[M+Na]^+$ calculated for $C_{38}H_{29}NO_4S$: 632.1866, found: 632.1853;

HPLC: The enantiomeric excess was determined using CHIRALPAK ID column (*n*-Hexane/ *i*-PrOH =90:10, flow rate=1.0 mL/min, λ= 254 nm, τ_{major} = 23.0 min, τ_{minor} = 29.4 min).

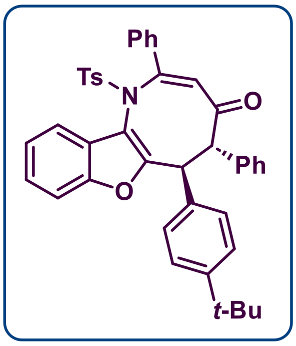

(5*S*,6*S*,*Z*)-6-(4-(*tert*-butyl)phenyl)-2,5-diphenyl-1-tosyl-5,6-dihydrobenzofuro[3,2-b]azocin-4(1*H*)-one: White solid, 60.9 mg, 90% yield, >20:1 *dr*, 85% *ee*; R_f = 0.5 (petroleum ether/ethyl acetate = 95:5)

^{1}H NMR (500 MHz, Chloroform-d) *δ* 7.54 – 7.49 (m, 4H), 7.47 – 7.43 (m, 3H), 7.39 (d, *J* = 8.2 Hz, 1H), 7.33 (t, *J* = 7.6 Hz, 2H), 7.25 – 7.24 (m, 2H), 7.22 (t, *J* = 7.5 Hz, 2H), 7.18 – 7.13 (m, 6H), 7.05 (t, *J* = 9.2 Hz, 3H), 6.02 (d, *J* = 13.1 Hz, 1H), 5.90 (s, 1H), 4.86 (d, *J* = 13.2 Hz, 1H), 2.42 (s, 3H), 1.19 (s, 9H). **^{13}C NMR** (126 MHz, Chloroform-d) *δ* 201.37, 158.83, 154.79, 152.62, 149.86, 144.78, 137.07, 134.43, 130.31, 130.19, 129.97, 129.52, 129.34, 129.10, 128.51, 128.22, 127.38, 126.83, 126.34, 125.25, 123.20, 120.45, 119.25, 112.26, 54.40, 48.99, 34.53, 31.44, 21.86.

HRMS (ESI$^+$) *m/z*: $[M+H]^+$ calculated for $C_{42}H_{37}NO_4S$: 652.2517, found: 652.2517;

HPLC: The enantiomeric excess was determined using CHIRALPAK ID column (*n*-Hexane/ *i*-PrOH =90:10, flow rate=1.0 mL/min, λ= 254 nm, τ_{major} = 13.6 min, τ_{minor} = 19.7 min).

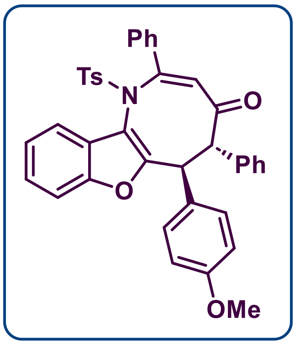

(5*S*,6*S*,*Z*)-6-(4-methoxyphenyl)-2,5-diphenyl-1-tosyl-5,6-dihy*dro* benzofuro[3,2-b]azocin-4(1*H*)-one (3d): White solid, 58.7 mg, 94% yield, >20:1 *dr*, 90% *ee*; R_f = 0.5 (petroleum ether/ethyl acetate = 95:5);

1H NMR (500 MHz, Chloroform-d) δ 7.51 (q, J = 8.0 Hz, 6H), 7.44 (t, J = 7.4 Hz, 1H), 7.37 (d, J = 8.2 Hz, 1H), 7.32 (t, J = 7.6 Hz, 2H), 7.24 – 7.21 (m, 3H), 7.16 – 7.12 (m, 4H), 7.03 (t, J = 7.3 Hz, 3H), 6.70 (d, J = 8.6 Hz, 2H), 6.02 (d, J = 13.1 Hz, 1H), 5.90 (s, 1H), 4.84 (d, J = 13.1 Hz, 1H), 3.66 (s, 3H), 2.40 (s, 3H). **13C NMR** (126 MHz, Chloroform-d) δ 201.21, 158.76, 158.69, 154.81, 152.59, 144.82, 136.98, 136.53, 136.35, 131.03, 130.33, 130.19, 129.92, 129.74, 129.33, 129.07, 128.51, 128.27, 127.47, 126.83, 126.27, 125.23, 123.20, 120.33, 119.23, 113.76, 112.20, 55.20, 54.46, 48.70, 21.82.

HRMS (ESI$^+$) *m/z*: $[M+H]^+$ calculated for $C_{39}H_{31}NO_5S$: 626.1996, found: 626.1996;

HPLC: The enantiomeric excess was determined using CHIRALPAK ID column (*n*-Hexane/ *i*-PrOH =90:10, flow rate=1.0 mL/min, λ= 254 nm, τ_{major} = 13.6 min, τ_{minor} = 19.7 min).

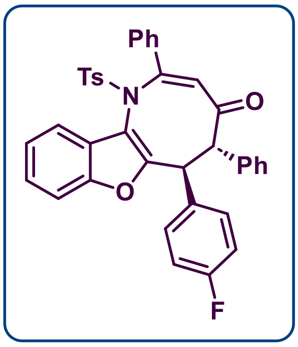

(5*S*,6*S*,*Z*)-6-(4-fluorophenyl)-2,5-diphenyl-1-tosyl-5,6-dihydro benzofuro[3,2-b]azocin-4(1*H*)-one (3e): White solid, 55.1 mg, 90% yield, >20:1 *dr*, 91% *ee*; R_f = 0.5 (petroleum ether/ethyl acetate = 95:5)

^{1}H NMR (500 MHz, Chloroform-d) δ 7.58 – 7.51 (m, 6H), 7.47 (t, *J* = 7.4 Hz, 1H), 7.40 (d, *J* = 8.2 Hz, 1H), 7.34 (t, *J* = 7.5 Hz, 2H), 7.30 – 7.23 (m, 4H), 7.20 – 7.17 (m, 2H), 7.13 (d, *J* = 8.1 Hz, 2H), 7.05 (d, *J* = 8.0 Hz, 3H), 6.87 (t, *J* = 8.7 Hz, 2H), 6.03 (d, *J* = 13.1 Hz, 1H), 5.93 (s, 1H), 4.88 (d, *J* = 13.1 Hz, 1H), 2.43 (s, 3H). **^{13}C NMR** (126 MHz, Chloroform-d) δ 200.95, 163.03, 161.07, 158.13, 154.88, 152.63, 144.94, 136.86, 136.42, 136.04, 133.43, 133.41, 131.63, 131.57, 130.41, 130.16, 129.93, 129.37, 129.07, 128.56, 128.37, 127.64, 126.80, 126.14, 125.44, 123.32, 120.58, 119.32, 115.35, 115.18, 112.22, 54.28, 48.77, 21.86.

HRMS (ESI$^+$) *m/z*: $[M+K]^+$ calculated for $C_{38}H_{28}FNO_4S$: 652.1355, found: 652.1358;

HPLC: The enantiomeric excess was determined using CHIRALPAK ID column (*n*-Hexane/ *i*-PrOH =90:10, flow rate=1.0 mL/min, λ= 254 nm, τ_{major} = 17.5 min, τ_{minor} = 27.0 min).

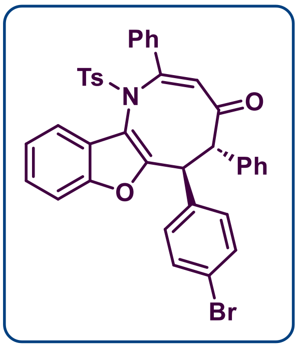

(5*S*,6*S*,*Z*)-6-(4-bromophenyl)-2,5-diphenyl-1-tosyl-5,6-dihy*dro* benzofuro[3,2-b]azocin-4(1*H*)-one (3f): White solid, 61.3 mg, 91% yield, >20:1 *dr*, 90% *ee*; R_f = 0.5 (petroleum ether/ethyl acetate = 95:5)

^{1}H NMR (400 MHz, Chloroform-d) δ 7.54 – 7.51 (m, 3H), 7.50 – 7.47 (m, 2H), 7.46 – 7.44 (m, 2H), 7.39 (d, *J* = 8.2 Hz, 1H), 7.34 (d, *J* = 7.6 Hz, 2H), 7.32 – 7.28 (m, 3H), 7.24 – 7.23 (m, 2H), 7.20 – 7.15 (m, 2H), 7.14 – 7.10 (m, 2H), 7.05 – 7.02 (m, 3H), 6.03 (d, *J* = 13.2 Hz, 1H), 5.92 (s, 1H), 4.85 (d, *J* = 13.2 Hz, 1H), 2.42 (s, 3H). **^{13}C NMR** (101 MHz, Chloroform-d) δ 200.79, 157.78, 154.92, 152.65, 144.96, 136.83, 136.67, 136.41, 135.88, 131.73, 131.53, 130.44, 130.11, 129.93, 129.38, 129.07, 128.57, 128.47, 127.76, 126.75, 126.09, 125.50, 123.36, 121.53, 120.66, 119.31, 112.24, 53.93, 48.82, 21.88.

HRMS (ESI$^+$) *m/z*: $[M+H]^+$ calculated for $C_{38}H_{28}BrNO_4S$: 674.0995, found: 674.1024;

HPLC: The enantiomeric excess was determined using CHIRALPAK ID column (*n*-Hexane/ *i*-PrOH =90:10, flow rate=1.0 mL/min, λ= 254 nm, τ_{major} = 19.8 min, τ_{minor} = 29.0 min).

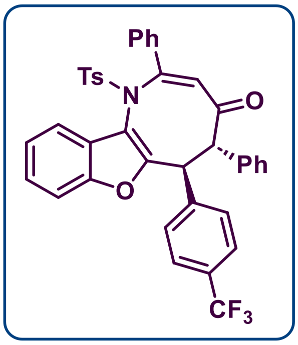

(5*S*,6*S*,*Z*)-2,5-diphenyl-1-tosyl-6-(4-(trifluoromethyl)phenyl)-5,6-dihydrobenzofuro[3,2-b]azocin-4(1*H*)-one (3g): White solid, 60.9 mg, 92% yield, >20:1 *dr*, 89% *ee*; R_f = 0.5 (petroleum ether/ethyl acetate = 95:5)

^{1}H NMR (500 MHz, Chloroform-d) δ 7.72 (d, J = 8.1 Hz, 2H), 7.54 – 7.51 (m, 4H), 7.46 - 7.43 (m, 3H), 7.39 (d, J = 8.2 Hz, 1H), 7.34 (t, J = 7.7 Hz, 2H), 7.31 – 7.27 (m, 1H), 7.25 – 7.22 (m, 2H), 7.17 (q, J = 7.5 Hz, 2H), 7.12 (d, J = 8.3 Hz, 2H), 7.06 – 7.03 (m, 3H), 6.08 (d, J = 13.2 Hz, 1H), 5.94 (s, 1H), 4.95 (d, J = 13.1 Hz, 1H), 2.43 (s, 3H). **^{13}C NMR** (126 MHz, Chloroform-d) δ 200.41, 157.26, 154.75, 141.43, 135.51, 130.26, 130.18, 129.87, 129.73, 129.19, 128.87, 128.38, 128.30, 127.62, 126.54, 125.41, 125.15, 125.12, 123.22, 120.71, 119.16, 112.06, 53.63, 48.94, 21.66.

HRMS (ESI$^+$) *m/z*: $[M+H]^+$ calculated for $C_{39}H_{28}F_3NO_4S$: 664.1764, found: 664.1797;

HPLC: The enantiomeric excess was determined using CHIRALPAK IA column (*n*-Hexane/ *i*-PrOH =90:10, flow rate=1.0 mL/min, λ= 220 nm, τ_{major} = 10.73 min, τ_{minor} = 15.5 min).

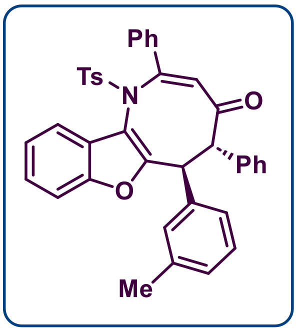

(5*S*,6*S*,*Z*)-2,5-diphenyl-6-(*m*-tolyl)-1-tosyl-5,6-dihydro benzofuro[3,2-b]azocin-4(1*H*)-one (3h): White solid, 54.8 mg, 90% yield, >20:1 *dr*, 92% *ee*; R_f = 0.5 (petroleum ether/ethyl acetate = 95:5)

^{1}H NMR (500 MHz, Chloroform-d) δ 7.54 (d, J = 6.9 Hz, 2H), 7.50 (d, J = 7.0 Hz, 2H), 7.47 – 7.43 (m, 1H), 7.41 – 7.36 (m, 3H), 7.33 (t, J = 7.6 Hz, 2H), 7.28 – 7.27 (m, 1H), 7.26 – 7.25 (m, 1H), 7.23 – 7.20 (m, 2H), 7.17 – 7.14 (m, 4H), 7.08 (s, 1H), 7.05 (dd, J = 8.1, 6.4 Hz, 3H), 6.89 (d, J = 7.5 Hz, 1H), 6.02 (d, J = 13.1 Hz, 1H), 5.90 (s, 1H), 4.83 (d, J = 13.1 Hz, 1H), 2.42 (s, 3H), 2.23 (s, 3H). **^{13}C NMR** (126 MHz, Chloroform-d) δ 201.21, 158.61, 154.82, 152.65, 144.79, 137.83, 137.48, 137.10, 136.62, 136.31, 130.88, 130.31, 130.24, 129.97, 129.34, 129.13, 128.51, 128.20, 128.17, 128.11, 127.47, 127.00, 126.91, 126.37, 125.25, 123.21, 120.57, 119.27, 112.27, 54.35, 49.47, 21.84, 21.56.

HRMS (ESI$^+$) *m/z*: $[M+H]^+$ calculated for $C_{39}H_{31}NO_4S$: 610.2047, found: 610.2050;

HPLC: The enantiomeric excess was determined using CHIRALPAK ID column (*n*-Hexane/ *i*-PrOH=90:10, flow rate=1.0 mL/min, λ= 254 nm, τ_{major} = 23.0 min, τ_{minor} = 28.1 min).

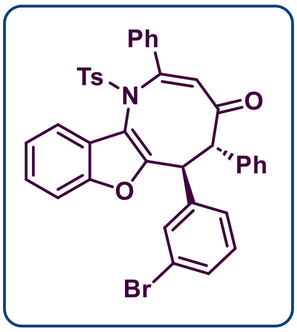

(5*S*,6*S*,*Z*)-6-(3-bromophenyl)-2,5-diphenyl-1-tosyl-5,6-dihydro benzofuro[3,2-b]azocin-4(1*H*)-one (3i): White solid, 59.9 mg, 89% yield, >20:1 *dr*, 91% *ee*; R_f = 0.5 (petroleum ether/ethyl acetate = 95:5)

^{1}H NMR (400 MHz, Chloroform-d) δ 7.77 – 7.76 (m, 1H), 7.53 – 7.46 (m, 6H), 7.43 (d, *J* = 7.6 Hz, 1H), 7.39 (d, *J* = 8.2 Hz, 1H), 7.33 (d, *J* = 7.6 Hz, 2H), 7.31 – 7.26 (m, 1H), 7.26-7.21 (m, 3H), 7.20 – 7.18 (m, 1H), 7.17 – 7.11 (m, 4H), 7.04 – 6.99 (m, 4H), 5.98 (d, *J* = 13.2 Hz, 1H), 5.90 (s, 1H), 4.81 (d, *J* = 13.1 Hz, 1H), 2.41 (s, 3H). **^{13}C NMR** (126 MHz, Chloroform-d) δ 200.66, 157.59, 154.89, 152.71, 144.93, 139.93, 136.89, 136.52, 135.82, 133.11, 130.53, 130.42, 130.17, 129.94, 129.87, 129.38, 129.13, 128.60, 128.56, 128.43, 127.75, 126.83, 126.17, 125.54, 123.38, 122.36, 120.92, 119.37, 112.30, 54.07, 49.16, 21.85.

HRMS (ESI$^+$) *m/z*: $[M+H]^+$ calculated for $C_{38}H_{28}BrNO_4S$: 674.0995, found: 674.1001;

HPLC: The enantiomeric excess was determined using CHIRALPAK ID column (*n*-Hexane/ *i*-PrOH =90:10, flow rate=1.0 mL/min, λ= 254 nm, τ_{major} = 22.7 min, τ_{minor} = 33.8 min).

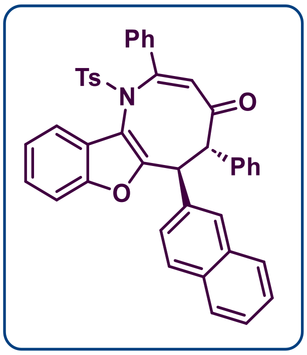

(5*S*,6*S*,*Z*)-6-(naphthalen-2-yl)-2,5-diphenyl-1-tosyl-5,6-dihydro benzofuro[3,2-b]azocin-4(1*H*)-one (3j): White solid, 56.7 mg, 88% yield, >20:1 *dr*, 95% *ee*; R_f = 0.5 (petroleum ether/ethyl acetate = 95:5)

^{1}H NMR (500 MHz, Chloroform-d) δ 8.10 (s, 1H), 7.78 – 7.77 (m, 1H), 7.74 – 7.72 (m, 1H), 7.69 – 7.67 (m, 1H), 7.64 (d, *J* = 8.6 Hz, 1H), 7.57 – 7.54 (m, 4H), 7.48 – 7.45 (m, 1H), 7.39 – 7.33 (m, 5H), 7.26 – 7.23 (m, 1H), 7.19 – 7.13 (m, 5H), 7.10 – 7.03 (m, 4H), 6.16 (d, *J* = 13.1 Hz, 1H), 5.94 (s, 1H), 5.05 (d, *J* = 13.1 Hz, 1H), 2.42 (s, 3H). **^{13}C NMR** (126 MHz, Chloroform-d) δ 201.08, 158.39, 154.92, 152.68, 144.87, 137.01, 136.61, 136.14, 135.27, 133.51, 132.73, 130.38, 130.19, 129.99, 129.53, 129.37, 129.16, 128.55, 128.30, 127.95, 127.67, 127.64, 127.56, 126.92, 126.30, 125.88, 125.34, 123.26, 119.30, 112.24, 54.32, 49.57, 21.85.

HRMS (ESI$^+$) *m/z*: $[M+K]^+$ calculated for $C_{42}H_{31}NO_4S$: 684.1606, found: 684.1611;

HPLC: The enantiomeric excess was determined using CHIRALPAK ID column (*n*-Hexane/ *i*-PrOH=90:10, flow rate=1.0 mL/min, λ= 254 nm, τ_{major} = 33.4 min, τ_{minor} = 40.5 min).

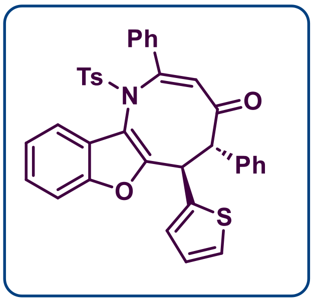

(5*S*,6*R*,*Z*)-2,5-diphenyl-6-(thiophen-2-yl)-1-tosyl-5,6-dihydro benzofuro[3,2-b]azocin-4(1*H*)-one (3k): White solid, 54.0 mg, 90% yield, >20:1 *dr*, 85% *ee*; R_f = 0.5 (petroleum ether/ethyl acetate = 95:5)

1H NMR (400 MHz, Chloroform-d) *δ* 7.57 – 7.42 (m, 4H), 7.46 – 7.42 (m, 2H), 7.35 (d, *J* = 7.5 Hz, 2H), 7.33 – 7.28 (m, 4H), 7.25 (d, *J* = 5.9 Hz, 1H), 7.19 – 7.14 (m, 4H), 7.11 (d, *J* = 7.8 Hz, 1H), 7.05 (d, *J* = 7.7 Hz, 2H), 6.81 - 6.79 (m, 1H), 5.90 (d, *J* = 12.9 Hz, 1H), 5.88 (s, 1H), 5.15 (d, *J* = 13.0 Hz, 1H), 2.42 (s, 3H). **^{13}C NMR** (101 MHz, Chloroform-d) *δ* 200.55, 157.62, 144.89, 139.85, 136.83, 136.55, 136.17, 130.43, 130.18, 129.84, 129.41, 129.05, 128.53, 128.44, 127.93, 127.90, 126.71, 126.45, 125.54, 125.19, 123.43, 119.31, 112.33, 55.42, 44.20, 21.85.

HRMS (ESI$^+$) *m/z*: $[M+H]^+$ calculated for $C_{36}H_{27}NO_4S_2$: 602.1450, found: 602.1450;

HPLC: The enantiomeric excess was determined using CHIRALPAK ID column (*n*-Hexane/ *i*-PrOH =90:10, flow rate=1.0 mL/min, λ= 254 nm, τ_{major} = 32.4 min, τ_{minor} = 45.8 min).

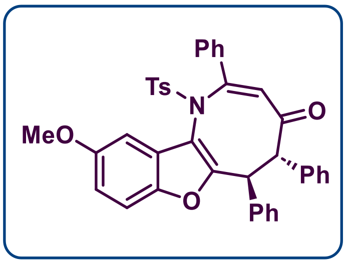

(5*S*,6*S*,*Z*)-10-methoxy-2,5,6-triphenyl-1-tosyl-5,6-dihy*dro* benzofuro[3,2-b]azocin-4(1*H*)-one (3l): White solid, 57.5 mg, 92% yield, >20:1 *dr*, 93% *ee*; R_f = 0.5 (petroleum ether/ethyl acetate = 95:5)

^{1}H NMR (500 MHz, Chloroform-d) *δ* 7.64 – 7.61 (m, 4H), 7.56 – 7.55 (m, 2H), 7.51 (t, *J* = 7.4 Hz, 1H), 7.39 (t, *J* = 7.7 Hz, 2H), 7.31 – 7.24 (m, 4H), 7.22 – 7.17 (m, 5H), 7.14 (d, *J* = 7.4 Hz, 1H), 7.10 (d, *J* = 8.1 Hz, 2H), 6.88 (dd, *J* = 8.9, 2.5 Hz, 1H), 6.42 (d, *J* = 2.5 Hz, 1H), 6.11 (d, *J* = 13.1 Hz, 1H), 5.98 (s, 1H), 4.90 (d, *J* = 13.1 Hz, 1H), 3.67 (s, 3H), 2.44 (s, 3H). **^{13}C NMR** (126 MHz, Chloroform-d) *δ* 201.10, 159.09, 156.17, 154.85, 147.56, 144.82, 137.70, 137.08, 136.76, 136.19, 130.29, 130.23, 130.03, 129.90, 129.36, 129.23, 128.49, 128.34, 128.24, 127.49, 127.31, 127.24, 126.62, 120.54, 114.00, 112.77, 101.88, 55.61, 54.23, 49.69, 21.77.

HRMS (ESI$^+$) *m/z*: $[M+H]^+$ calculated for $C_{39}H_{31}NO_5S$: 626.1996, found: 626.2004;

HPLC: The enantiomeric excess was determined using CHIRALPAK ID column (*n*-Hexane/ *i*-PrOH =90:10, flow rate=1.0 mL/min, λ= 254 nm, τ_{major} = 26.8 min, τ_{minor} = 30.3 min).

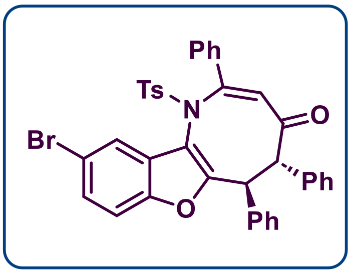

(5*S*,6*S*,*Z*)-10-bromo-2,5,6-triphenyl-1-tosyl-5,6-dihy*dro* benzofuro[3,2-b]azocin-4(1*H*)-one (3m): White solid, 61.3 mg, 91% yield, >20:1 *dr*, 93% *ee*; R_f = 0.5 (petroleum ether/ethyl acetate = 95:5)

^{1}H NMR (500 MHz, Chloroform-d) *δ* 7.55 (d, *J* = 8.7 Hz, 3H), 7.49 (d, *J* = 7.2 Hz, 4H), 7.46 (d, *J* = 7.3 Hz, 1H), 7.34 (t, *J* = 7.5 Hz, 2H), 7.28 – 7.26 (m, 2H), 7.21 (q, *J* = 6.6, 5.8 Hz, 3H), 7.17 – 7.15 (m, 2H), 7.11 (d, *J* = 7.8 Hz, 3H), 7.05 (d, *J* = 8.0 Hz, 2H), 6.86 (d, *J* = 8.3 Hz, 1H), 6.01 (d, *J* = 13.0 Hz, 1H), 5.93 (s, 1H), 4.84 (d, *J* = 13.0 Hz, 1H), 2.43 (s, 3H). **^{13}C NMR** (126 MHz, Chloroform-d) *δ* 200.97, 159.14, 154.59, 152.87, 145.10, 137.25, 136.74, 136.45, 135.97, 130.46, 130.18, 129.98, 129.83, 129.45, 129.05, 128.61, 128.41, 128.29, 127.58, 127.46, 126.99, 126.72, 125.35, 120.43, 120.22, 118.59, 115.69, 54.11, 49.45, 21.88.

HRMS (ESI$^+$) *m/z*: $[M+H]^+$ calculated for $C_{38}H_{28}BrNO_4S$: 674.0995, found: 674.0961;

HPLC: The enantiomeric excess was determined using CHIRALPAK ID column (*n*-Hexane/ *i*-PrOH =90:10, flow rate=1.0 mL/min, λ= 254 nm, τ_{major} = 22.7 min, τ_{minor} = 33.8 min).

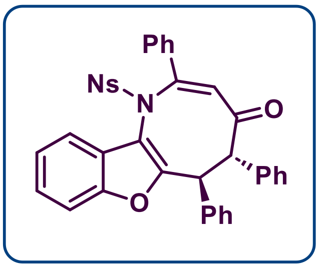

(5*S*,6*S*,*Z*)-1-((4-nitrophenyl)sulfonyl)-2,5,6-triphenyl-5,6-di hydrobenzofuro[3,2-b]azocin -4(1*H*)-one (3n): Yellow solid, 34.1 mg, 95% yield, >20:1 *dr*, >99% *ee*; R_f = 0.5 (petroleum ether/ethyl acetate = 90:10);

1H NMR (600 MHz, Chloroform-d) δ 8.06 (d, J = 8.8 Hz, 2H), 7.58 (d, J = 7.3 Hz, 2H), 7.53 - 7.50 (m, 5H), 7.44 (d, J = 8.2 Hz, 1H), 7.41 – 7.38 (m, 2H), 7.35 (t, J = 7.6 Hz, 2H), 7.33 – 7.30 (m, 1H), 7.24 (t, J = 7.5 Hz, 2H), 7.20 – 7.16 (m, 4H), 7.13 – 7.10 (m, 1H), 6.95 (d, J = 7.8 Hz, 1H), 6.04 (d, J = 13.2 Hz, 1H), 6.00 (s, 1H), 4.93 (d, J = 13.2 Hz, 1H). **^{13}C NMR** (151 MHz, Chloroform-d) δ 200.88, 158.93, 153.46, 152.77, 150.53, 145.51, 137.14, 136.13, 135.82, 130.83, 130.28, 130.10, 130.00, 129.96, 128.84, 128.45, 128.42, 127.74, 127.73, 127.54, 125.76, 125.67, 123.74, 123.62, 119.62, 118.45, 112.72, 54.41, 49.50.

HRMS (ESI$^+$) *m/z*: $[M+H]^+$ calculated for $C_{37}H_{26}N_2O_6S$: 649.1404, found: 649.1409;

HPLC: The enantiomeric excess was determined using CHIRALPAK ID column (*n*-Hexane/ *i*-PrOH =90:10, flow rate=1.0 mL/min, λ= 254 nm, τ_{major} = 16.4 min, τ_{minor} = 25.7 min)

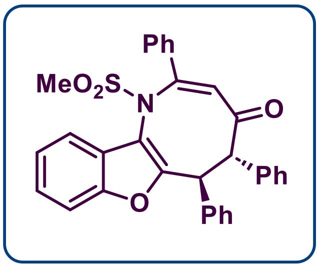

(5*S*,6*S*,*Z*)-1-(methylsulfonyl)-2,5,6-triphenyl-5,6-dihydro benzofuro[3,2-b]azocin-4(1*H*)-one(3o): White solid, 47.8 mg, 92% yield, >20:1 *dr*, >89% *ee*; R_f = 0.5 (petroleum ether/ethyl acetate = 95:5)

^{1}H NMR (500 MHz, Chloroform-d) *δ* 7.82 – 7.80 (m, 2H), 7.77 (d, *J* = 7.6 Hz, 1H), 7.60 – 7.54 (m, 3H), 7.50 - 7.47 (m, 4H), 7.43 (t, *J* = 7.3 Hz, 1H), 7.39 (d, *J* = 7.5 Hz, 1H), 7.36 – 7.33 (m, 1H), 7.21 (t, *J* = 7.4 Hz, 2H), 7.14 (t, *J* = 7.8 Hz, 3H), 7.07 (t, *J* = 7.3 Hz, 1H), 5.97 (s, 1H), 5.90 (d, *J* = 13.1 Hz, 1H), 4.88 (d, *J* = 13.1 Hz, 1H), 3.01 (s, 3H). **^{13}C NMR** (126 MHz, Chloroform-d) *δ* 201.14, 158.54, 153.91, 137.31, 136.92, 135.95, 130.80, 130.13, 129.92, 129.53, 129.09, 128.34, 128.31, 127.59, 127.39, 127.06, 126.21, 125.62, 124.04, 119.61, 117.91, 112.74, 54.46, 49.44, 44.02.

HRMS (ESI$^+$) *m/z*: $[M+H]^+$ calculated for $C_{32}H_{25}NO_4S$: 520.1578, found: 520.1578;

HPLC: The enantiomeric excess was determined using CHIRALPAK ID column (*n*-Hexane/ *i*-PrOH =90:10, flow rate=1.0 mL/min, λ= 254 nm, τ_{major} = 19.9 min, τ_{minor} = 34.4 min).

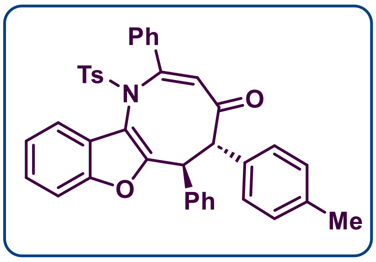

(5*S*,6*S*,*Z*)-2,6-diphenyl-5-(*p*-tolyl)-1-tosyl-5,6-dihydro benzofuro[3,2-b]azocin-4(1*H*)-one(3p): White solid, 56.0 mg, 92% yield, >20:1 *dr*, 89% *ee*; R_f = 0.5 (petroleum ether/ethyl acetate = 95:5)

^{1}H NMR (500 MHz, Chloroform-d) *δ* 7.60 (d, *J* = 7.8 Hz, 2H), 7.54 (d, *J* = 7.6 Hz, 2H), 7.45 (t, *J* = 7.4 Hz, 1H), 7.40 - 7.37 (m, 3H), 7.34 (t, *J* = 7.5 Hz, 2H), 7.28 – 7.25 (m, 2H), 7.19 (t, *J* = 7.5 Hz, 2H), 7.17 – 7.13 (m, 3H), 7.11 (t, *J* = 7.4 Hz, 1H), 7.05 (q, *J* = 8.8, 7.3 Hz, 5H), 6.03 (d, *J* = 13.1 Hz, 1H), 5.91 (s, 1H), 4.88 (d, *J* = 13.1 Hz, 1H), 2.42 (s, 3H), 2.25 (s, 3H). **^{13}C NMR** (126 MHz, Chloroform-d) *δ* 201.37, 158.59, 154.74, 152.65, 144.81, 137.74, 137.08, 136.62, 133.15, 130.31, 130.08, 129.98, 129.94, 129.34, 129.11, 129.03, 128.51, 128.34, 127.29, 126.88, 126.30, 125.26, 123.21, 120.49, 119.28, 112.22, 53.87, 49.34, 21.83, 21.26.

HRMS (ESI$^+$) *m/z*: $[M+H]^+$ calculated for $C_{39}H_{31}NO_4S$: 610.2047, found: 610.2056;

HPLC: The enantiomeric excess was determined using CHIRALPAK ID column (*n*-Hexane/ *i*-PrOH =90:10, flow rate=1.0 mL/min, λ= 254 nm, τ_{major} = 24.3 min, τ_{minor} = 39.0 min).

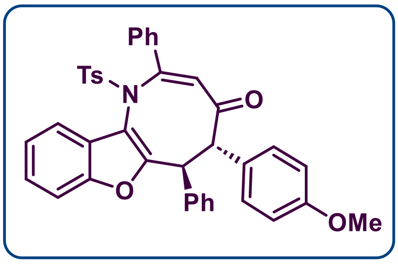

(5*S*,6*S*,*Z*)-5-(4-methoxyphenyl)-2,6-diphenyl-1-tosyl-5,6-dihydrobenzofuro[3,2-b]azocin-4(1*H*)-one(3q): White solid, 55.6 mg, 89% yield, >20:1 *dr*, 94% *ee*; R_f = 0.5 (petroleum ether/ethyl acetate = 95:5)

^{1}H NMR (500 MHz, Chloroform-d) δ 7.58 (d, J = 7.6 Hz, 2H), 7.54 (d, J = 7.6 Hz, 2H), 7.47 - 7.71 (m, 3H), 7.38 (d, J = 8.2 Hz, 1H), 7.33 (t, J = 7.6 Hz, 2H), 7.28 - 7.25 (m, 1H), 7.19 (t, J = 7.6 Hz, 2H), 7.16 – 7.13 (m, 3H), 7.10 (t, J = 7.3 Hz, 1H), 7.06 - 7.03 (m, 3H), 6.76 (d, J = 8.3 Hz, 2H), 5.99 (d, J = 13.1 Hz, 1H), 5.92 (s, 1H), 4.84 (d, J = 13.1 Hz, 1H), 3.73 (s, 3H), 2.42 (s, 3H). **^{13}C NMR** (126 MHz, Chloroform-d) δ 201.57, 159.02, 158.55, 154.77, 152.65, 144.82, 137.77, 137.04, 136.63, 131.15, 130.32, 130.08, 129.94, 129.34, 129.12, 128.52, 128.37, 127.30, 126.92, 126.29, 125.28, 123.21, 120.54, 119.28, 113.75, 112.23, 55.35, 53.43, 49.58, 21.83.

HRMS (ESI$^+$) *m/z*: [M+Na]$^+$ calculated for $C_{39}H_{31}NO_5S$: 648.1816, found: 648.1820;

HPLC: The enantiomeric excess was determined using CHIRALPAK ID column (*n*-Hexane/ *i*-PrOH =90:10, flow rate=1.0 mL/min, λ= 254 nm, τ_{major} = 23.6 min, τ_{minor} = 43.7 min).

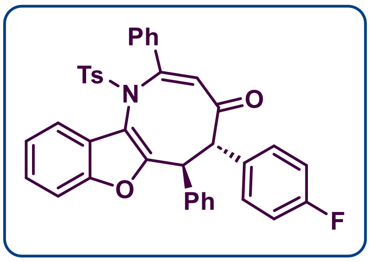

(5*S*,6*S*,*Z*)-5-(4-fluorophenyl)-2,6-diphenyl-1-tosyl-5,6-dihydrobenzofuro[3,2-b]azocin-4(1*H*)-one(3r): White solid, 53.9 mg, 88% yield, >20:1 *dr*, 89% *ee*; R_f = 0.5 (petroleum ether/ethyl acetate = 95:5)

^{1}H NMR (500 MHz, Chloroform-d) δ 7.53 (t, J = 8.3 Hz, 4H), 7.47 - 7.42 (m, 3H), 7.36 (d, J = 8.2 Hz, 1H), 7.32 (t, J = 7.6 Hz, 2H), 7.24 - 7.23 (m, 1H), 7.17 (t, J = 7.5 Hz, 2H), 7.14 – 7.11 (m, 3H), 7.09 (t, J = 7.4 Hz, 1H), 7.03 – 7.01 (m, 3H), 6.88 (t, J = 8.5 Hz, 2H), 6.04 (d, J = 13.1 Hz, 1H), 5.91 (s, 1H), 4.78 (d, J = 13.1 Hz, 1H), 2.40 (s, 3H). **^{13}C NMR** (126 MHz, Chloroform-d) δ 200.91, 163.32, 161.37, 158.22, 155.18, 152.67, 144.93, 137.57, 136.95, 136.55, 132.09, 132.06, 131.76, 131.70, 130.44, 130.02, 129.95, 129.38, 129.14, 128.56, 128.47, 127.45, 126.83, 126.22, 125.37, 123.28, 120.63, 119.29, 115.21, 115.04, 112.27, 53.39, 49.83, 21.84.

HRMS (ESI$^+$) *m/z*: [M+Na]$^+$ calculated for $C_{38}H_{28}FNO_4S$: 636.1616, found: 636.1605;

HPLC: The enantiomeric excess was determined using CHIRALPAK ID column (*n*-Hexane/ *i*-PrOH =90:10, flow rate=1.0 mL/min, λ= 254 nm, τ_{major} = 17.9 min, τ_{minor} = 28.0 min).

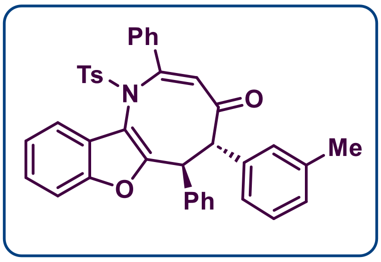

(5*S*,6*S*,*Z*)-2,6-diphenyl-5-(*m*-tolyl)-1-tosyl-5,6-dihydro benzofuro[3,2-b]azocin-4(1*H*)-one(3s): White solid, 55.4 mg, 91% yield, >20:1 *dr*, 92% *ee*; R_f = 0.5 (petroleum ether/ethyl acetate = 95:5)

^{1}H NMR (500 MHz, Chloroform-d) δ 7.58 (d, J = 7.3 Hz, 2H), 7.54 (d, J = 6.9 Hz, 2H), 7.45 (t, J = 7.4 Hz, 1H), 7.37 (d, J = 8.2 Hz, 1H), 7.33 (t, J = 7.8 Hz, 3H), 7.28 (d, J = 7.4 Hz, 1H), 7.25 – 7.24 (m, 1H), 7.19 – 7.13 (m, 5H), 7.11 – 7.07 (m, 2H), 7.04 (t, J = 8.5 Hz, 3H), 6.95 (d, J = 7.6 Hz, 1H), 6.00 (d, J = 13.2 Hz, 1H), 5.91 (s, 1H), 4.88 (d, J = 13.1 Hz, 1H), 2.41 (s, 3H), 2.28 (s, 3H). **^{13}C NMR** (126 MHz, Chloroform-d) δ 201.24, 158.59, 154.71, 152.66, 144.82, 137.73, 137.69, 137.05, 136.63, 136.06, 130.92, 130.32, 130.05, 129.96, 129.35, 129.12, 128.52, 128.31, 128.28, 128.12, 127.30, 127.20, 126.93, 126.31, 125.28, 123.22, 120.51, 119.29, 112.23, 54.18, 49.39, 21.83, 21.60.

HRMS (ESI$^+$) *m/z*: $[M+H]^+$ calculated for $C_{39}H_{31}NO_4S$: 610.2047, found: 610.2053;

HPLC: The enantiomeric excess was determined using CHIRALPAK ID column (*n*-Hexane/ *i*-PrOH =90:10, flow rate=1.0 mL/min, λ= 254 nm, τ_{major} = 18.5 min, τ_{minor} = 30.3 min).

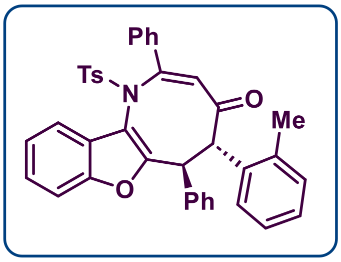

(5*S*,6*S*,*Z*)-2,6-diphenyl-5-(*o*-tolyl)-1-tosyl-5,6-dihydro benzofuro[3,2-b]azocin-4(1*H*)-one(3t): White solid, 54.2 mg, 89% yield, >20:1 *dr*, 92% *ee*; R_f = 0.5 (petroleum ether/ethyl acetate = 95:5)

^{1}H NMR (500 MHz, Chloroform-d) δ 7.71 (d, J = 7.9 Hz, 1H), 7.63 (d, J = 7.6 Hz, 2H), 7.52 (d, J = 7.5 Hz, 2H), 7.48 (t, J = 7.5 Hz, 1H), 7.40 – 7.34 (m, 3H), 7.24 (d, J = 7.6 Hz, 2H), 7.19 (t, J = 7.6 Hz, 2H), 7.15 – 7.09 (m, 3H), 7.07 – 7.00 (m, 5H), 6.85 (d, J = 7.9 Hz, 1H), 6.50 (d, J = 13.1 Hz, 1H), 5.92 (s, 1H), 4.98 (d, J = 13.1 Hz, 1H), 2.59 (s, 3H), 2.41 (s, 3H). **^{13}C NMR** (126 MHz, Chloroform-d) δ 201.12, 158.74, 154.80, 152.66, 144.85, 137.63, 137.43, 137.04, 136.61, 134.55, 130.30, 130.27, 129.99, 129.89, 129.55, 129.27, 129.21, 128.59, 128.35, 127.39, 127.11, 127.01, 126.02, 126.00, 125.26, 123.09, 120.19, 119.29, 112.23, 48.94, 48.89, 21.86, 21.14.

HRMS (ESI$^+$) *m/z*: $[M+H]^+$ calculated for $C_{39}H_{31}NO_4S$: 610.2047, found: 610.2047;

HPLC: The enantiomeric excess was determined using CHIRALPAK ID column (*n*-Hexane/ *i*-PrOH =90:10, flow rate=1.0 mL/min, λ= 254 nm, τ_{major} = 11.6 min, τ_{minor} = 18.8 min).

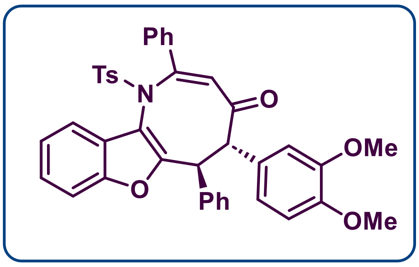

(5*S*,6*S*,*Z*)-5-(3,4-dimethoxyphenyl)-2,6-diphenyl-1-tosyl-5,6-dihydrobenzofuro[3,2-b]azocin-4(1*H*)-one(3u): White solid, 58.9 mg, 90% yield, >20:1 *dr*, 95% *ee*; R_f = 0.5 (petroleum ether/ethyl acetate = 95:5)

^{1}H NMR (400 MHz, Chloroform-d) *δ* 7.57 - 7.53 (m, 4H), 7.45 (t, *J* = 7.4 Hz, 1H), 7.38 – 7.31 (m, 3H), 7.26 (t, *J* = 7.7 Hz, 1H), 7.20 – 7.13 (m, 5H), 7.10 – 7.09 (m, 2H), 7.04 (d, *J* = 8.0 Hz, 3H), 7.01 - 6.99 (m, 1H), 6.69 (d, *J* = 8.3 Hz, 1H), 5.97 (d, *J* = 13.0 Hz, 1H), 5.92 (s, 1H), 4.82 (d, *J* = 13.0 Hz, 1H), 3.87 (s, 3H), 3.80 (s, 3H), 2.42 (s, 3H). **^{13}C NMR** (101 MHz, Chloroform-d) *δ* 201.52, 158.50, 154.71, 152.64, 148.78, 148.52, 144.85, 137.79, 137.00, 136.60, 130.34, 130.00, 129.93, 129.35, 129.11, 128.75, 128.53, 128.39, 127.35, 126.92, 126.26, 125.31, 123.23, 122.83, 120.57, 119.28, 113.19, 112.23, 110.79, 56.19, 55.95, 53.65, 49.88, 21.84.

HRMS (ESI$^+$) *m/z*: $[M+Na]^+$ calculated for $C_{40}H_{28}NO_6S$: 678.1921, found: 678.1928;

HPLC: The enantiomeric excess was determined using CHIRALPAK ID column (*n*-Hexane/ *i*-PrOH =90:10, flow rate=1.0 mL/min, λ= 254 nm, τ_{major} = 51.4 min, τ_{minor} = 84.6 min).

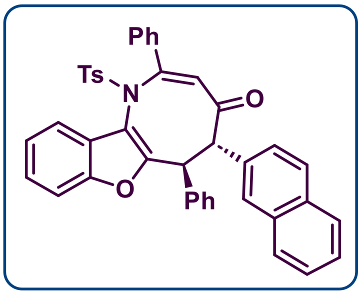

(5*S*,6*S*,*Z*)-5-(naphthalen-2-yl)-2,6-diphenyl-1-tosyl-5,6-dihydrobenzofuro[3,2-b]azocin-4(1*H*)-one(3v): White solid, 59.3 mg, 92% yield, >20:1 *dr*, 91% *ee*; R_f = 0.5 (petroleum ether/ethyl acetate = 95:5)

^{1}H NMR (400 MHz, Chloroform-d) *δ* 7.92 (s, 1H), 7.82 – 7.80 (m, 1H), 7.75 – 7.70 (m, 3H), 7.64 (d, *J* = 7.3 Hz, 2H), 7.57 (d, *J* = 7.0 Hz, 2H), 7.47 (t, *J* = 7.6 Hz, 1H), 7.43 – 7.39 (m, 3H), 7.35 (t, *J* = 7.5 Hz, 2H), 7.29 (d, *J* = 7.4 Hz, 1H), 7.19 – 7.11 (m, 5H), 7.06 (d, *J* = 8.0 Hz, 3H), 7.02 (t, *J* = 7.4 Hz, 1H), 6.25 (d, *J* = 13.1 Hz, 1H), 5.94 (s, 1H), 5.03 (d, *J* = 13.1 Hz, 1H), 2.44 (s, 3H). **^{13}C NMR** (101 MHz, Chloroform-d) *δ* 201.07, 158.52, 155.00, 152.70, 144.89, 137.52, 137.03, 136.61, 133.86, 133.36, 132.93, 130.39, 130.03, 129.98, 129.52, 129.39, 129.16, 128.56, 128.41, 128.16, 127.86, 127.72, 127.68, 127.39, 126.88, 126.29, 125.98, 125.94, 125.33, 123.26, 120.54, 119.31, 112.28, 54.35, 49.38, 21.87.

HRMS (ESI$^+$) *m/z*: $[M+K]^+$ calculated for $C_{42}H_{33}NO_4S$: 684.1606, found: 684.1607;

HPLC: The enantiomeric excess was determined using CHIRALPAK ID column (*n*-Hexane/ *i*-PrOH =90:10, flow rate=1.0 mL/min, λ= 254 nm, τ_{major} = 33.2 min, τ_{minor} = 52.7 min).

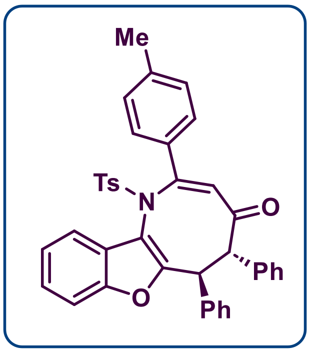

(5*S*,6*S*,*Z*)-5,6-diphenyl-2-(*p*-tolyl)-1-tosyl-5,6-dihydro benzofuro[3,2-b]azocin-4(1*H*)-one(3w): White solid, 56 mg, 92% yield, >20:1 *dr*, 91% *ee*; R_f = 0.5 (petroleum ether/ethyl acetate = 95:5)

^{1}H NMR (600 MHz, Chloroform-d) δ 7.58 (d, *J* = 7.6 Hz, 2H), 7.50 (d, *J* = 7.3 Hz, 2H), 7.42 (d, *J* = 7.8 Hz, 2H), 7.38 (d, *J* = 8.2 Hz, 1H), 7.28 – 7.25 (m, 2H), 7.21 (t, *J* = 7.5 Hz, 2H), 7.18 (d, *J* = 8.0 Hz, 3H), 7.17 – 7.15 (m, 2H), 7.13 (d, *J* = 7.9 Hz, 2H), 7.08 (d, *J* = 7.4 Hz, 2H), 7.06 (d, *J* = 8.0 Hz, 2H), 6.04 (d, *J* = 13.1 Hz, 1H), 5.90 (s, 1H), 4.87 (d, *J* = 13.1 Hz, 1H), 2.43 (s, 6H). **^{13}C NMR** (151 MHz, Chloroform-d) δ 201.14, 158.35, 155.02, 152.54, 144.80, 140.80, 137.64, 136.94, 136.21, 133.68, 130.18, 129.98, 129.82, 129.26, 129.16, 129.09, 128.34, 128.22, 127.46, 127.32, 126.28, 126.20, 125.27, 123.22, 120.48, 119.23, 112.20, 54.05, 49.35, 21.89, 21.69.

HRMS (ESI$^+$) *m/z*: $[M+H]^+$ calculated for $C_{39}H_{31}NO_4S$: 610.2047, found: 610.2054;

HPLC: The enantiomeric excess was determined using CHIRALPAK ID column (*n*-Hexane/ *i*-PrOH =90:10, flow rate=1.0 mL/min, λ= 254 nm, τ_{major} = 19.9 min, τ_{minor} = 34.4 min).

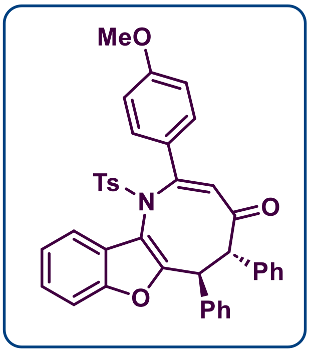

(5*S*,6*S*,*Z*)-2-(4-methoxyphenyl)-5,6-diphenyl-1-tosyl-5,6-dihydrobenzofuro[3,2-b]azocin-4(1*H*)-one(3x): White solid, 55.6 mg, 89% yield, >20:1 *dr*, 86% *ee*; R_f = 0.5 (petroleum ether/ethyl acetate = 95:5)

^{1}H NMR (400 MHz, Chloroform-d) δ 7.56 (d, *J* = 7.5 Hz, 2H), 7.49 (d, *J* = 7.0 Hz, 2H), 7.46 (d, *J* = 8.7 Hz, 2H), 7.37 (d, *J* = 8.2 Hz, 1H), 7.28 – 7.22 (m, 4H), 7.19 (d, *J* = 8.2 Hz, 2H), 7.16 – 7.11 (m, 4H), 7.08 (d, *J* = 7.3 Hz, 3H), 6.82 (d, *J* = 8.7 Hz, 2H), 6.03 (d, *J* = 13.1 Hz, 1H), 5.86 (s, 1H), 4.86 (d, *J* = 13.1 Hz, 1H), 3.87 (s, 3H), 2.42 (s, 3H). **^{13}C NMR** (101 MHz, Chloroform-d) δ 200.92, 161.65, 158.51, 154.74, 152.66, 144.75, 137.77, 137.27, 136.38, 131.47, 130.26, 130.04, 129.35, 129.16, 129.04, 128.33, 128.20, 127.43, 127.29, 126.44, 125.70, 125.27, 123.25, 120.58, 119.22, 113.94, 112.25, 55.71, 54.07, 49.50, 21.85.

HRMS (ESI$^+$) *m/z*: $[M+H]^+$ calculated for $C_{39}H_{31}NO_5S$: 626.1996, found: 626.1996;

HPLC: The enantiomeric excess was determined using CHIRALPAK ID column (*n*-Hexane/ *i*-PrOH =90:10, flow rate=1.0 mL/min, λ= 254 nm, τ_{major} = 15.6 min, τ_{minor} = 25.2 min).

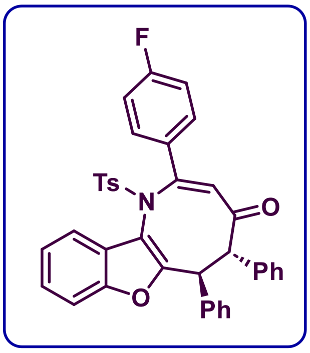

(5*S*,6*S*,*Z*)-2-(4-fluorophenyl)-5,6-diphenyl-1-tosyl-5,6-dihydrobenzofuro[3,2-b]azocin-4(1*H*)-one(3y): White solid, 55.1 mg, 90% yield, >20:1 *dr*, 89% *ee*; R_f = 0.5 (petroleum ether/ethyl acetate = 95:5)

^{1}H NMR (500 MHz, Chloroform-d) *δ* 7.57 – 7.49 (m, 6H), 7.39 (d, *J* = 8.2 Hz, 1H), 7.28 (d, *J* = 7.5 Hz, 3H), 7.21 (q, *J* = 6.9, 6.4 Hz, 4H), 7.17 – 7.13 (m, 4H), 7.11 - 7.07 (m, 3H), 7.05 – 7.02 (m, 3H), 6.02 (d, *J* = 13.1 Hz, 1H), 5.89 (s, 1H), 4.87 (d, *J* = 13.1 Hz, 1H), 2.44 (s, 3H). **^{13}C NMR** (126 MHz, Chloroform-d) *δ* 200.94, 165.16, 163.16, 158.56, 153.65, 152.67, 145.15, 137.57, 137.04, 136.14, 132.87, 132.85, 131.94, 131.87, 130.21, 130.03, 129.47, 129.06, 128.37, 128.29, 127.56, 127.38, 126.90, 126.27, 125.39, 123.33, 120.42, 119.06, 115.74, 115.57, 112.35, 54.24, 49.57, 21.86.

HRMS (ESI$^+$) *m/z*: $[M+K]^+$ calculated for $C_{38}H_{28}FNO_4S$: 652.1355, found: 652.1355;

HPLC: The enantiomeric excess was determined using CHIRALPAK ID column (*n*-Hexane/ *i*-PrOH =90:10, flow rate=1.0 mL/min, λ= 254 nm, τ_{major} = 17.2 min, τ_{minor} = 27.2 min).

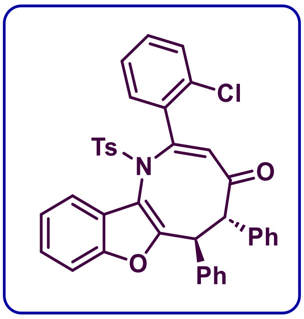

(5*S*,6*S*,*Z*)-2-(2-chlorophenyl)-5,6-diphenyl-1-tosyl-5,6-dihydrobenzofuro[3,2-b]azocin-4(1*H*)-one(3z): White solid, 58.5 mg, 93% yield, >20:1 *dr*, 83% *ee*; R_f = 0.5 (petroleum ether/ethyl acetate = 95:5)

^{1}H NMR (600 MHz, Chloroform-d) δ 7.58 (d, *J* = 7.2 Hz, 2H), 7.55 (d, *J* = 8.1 Hz, 1H), 7.53 – 7.50 (m, 3H), 7.39 (t, *J* = 8.0 Hz, 2H), 7.24 (t, *J* = 7.8 Hz, 3H), 7.20 - 7.16 (m, 4H), 7.16 – 7.13 (m, 2H), 7.12 – 7.08 (m, 2H), 7.04 (d, *J* = 8.0 Hz, 2H), 6.94 (d, *J* = 7.7 Hz, 1H), 6.06 (d, *J* = 13.2 Hz, 1H), 5.95 (s, 1H), 4.92 (d, *J* = 13.2 Hz, 1H), 2.41 (s, 3H).**^{13}C NMR** (151 MHz, Chloroform-d) δ 201.27, 158.64, 152.61, 149.38, 144.97, 137.39, 136.70, 136.08, 135.01, 134.71, 132.34, 131.01, 130.95, 130.01, 129.95, 129.43, 129.01, 128.93, 128.40, 127.58, 127.40, 126.37, 125.89, 125.29, 123.02, 119.85, 112.19, 54.52, 48.98, 21.85.

HRMS (ESI$^+$) *m/z*: $[M+H]^+$ calcd for $C_{38}H_{29}ClNO_4S$: 630.1501, found: 630.1501;

HPLC: The enantiomeric excess was determined using CHIRALPAK ID column (*n*-Hexane/*i*-PrOH =90:10, flow rate=1.0 mL/min, λ= 254 nm, τ_{major} = 24.2 min, τ_{minor} = 45.3 min).

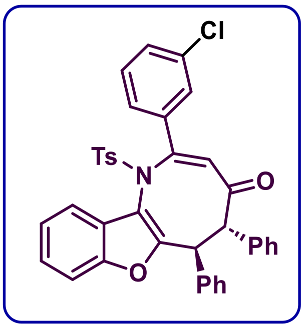

(5*S*,6*S*,*Z*)-2-(3-chlorophenyl)-5,6-diphenyl-1-tosyl-5,6-dihydrobenzofuro[3,2-b]azocin-4(1*H*)-one(3z'): White solid, 54.8 mg, 87% yield, >20:1 *dr*, 83% *ee*; R_f = 0.5 (petroleum ether/ethyl acetate = 95:5)

^{1}H NMR (500 MHz, Chloroform-d) δ 7.58 (d, *J* = 7.6 Hz, 2H), 7.51 (d, *J* = 7.6 Hz, 2H), 7.47 (d, *J* = 5.9 Hz, 2H), 7.43 - 7.38 (m, 2H), 7.33 (t, *J* = 7.9 Hz, 1H), 7.28 (d, *J* = 7.6 Hz, 1H), 7.24 - 7.21 (m, 4H), 7.21 - 7.15 (m, 4H), 7.14 - 7.08 (m, 3H), 7.04 (d, *J* = 7.8 Hz, 1H), 6.05 (d, *J* = 13.1 Hz, 1H), 5.95 (s, 1H), 4.89 (d, *J* = 13.1 Hz, 1H), 2.45 (s, 3H). **^{13}C NMR** (126 MHz, Chloroform-d) δ 200.99, 158.59, 153.20, 152.65, 145.38, 138.21, 137.49, 136.67, 136.03, 134.50, 130.36, 130.18, 130.03, 129.82, 129.56, 128.96, 128.37, 128.31, 128.06, 127.58, 127.39, 127.34, 126.12, 125.40, 123.40, 120.27, 119.04, 112.32, 54.25, 49.56, 21.85.

HRMS (ESI$^+$) *m/z*: $[M+H]^+$ calcd for $C_{38}H_{29}ClNO_4S$: 630.1501, found: 630.1498;

HPLC: The enantiomeric excess was determined using CHIRALPAK ID column (*n*-Hexane/*i*-PrOH =90:10, flow rate=1.0 mL/min, λ= 254 nm, τ_{major} = 15.5 min, τ_{minor} = 22.0 min).

6. General Procedure for the Synthesis of chiral Spiro-Cyclopentane Benzofurans:

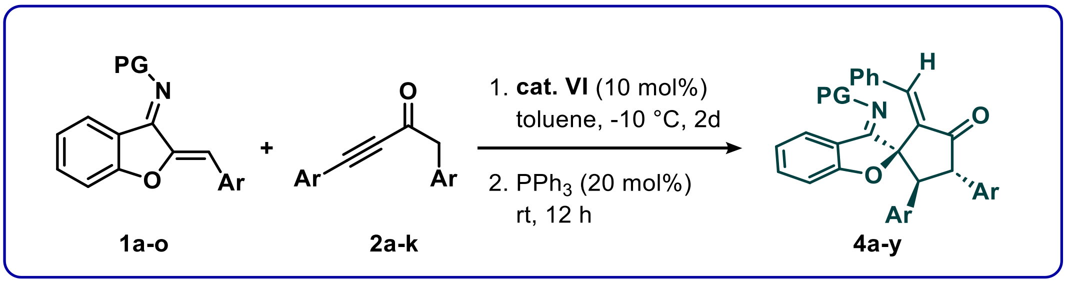

To a stirred solution of 1-azadienes **1a** (0.1 mmol) and freshly prepared ynones **2a** (0.11 mmol) in toluene solvent (1 mL) at -10 °C, were added 10 mol% catalyst **VI**. The reaction was allowed to run in the same temperature for 2 days. After full consumption of starting materials, solvents were evaporated and the reaction mixture was subjected to a short column chromatography (petroleum ether : ethyl acetate = 90:10) to afford intermediate **A.** Then the intermediate **A**

dissolved in 1ml DCM, and PPh_3 (20 mol%) was added subsequently. The reaction mixture was stirred at room temperature until the complete conversion of intermediate was detected. The solvents were removed under reduced pressure and purified by flash column chromatography (petroleum ether : ethyl acetate = 95:5 to 90:10) to give Spiro-Cyclopentane Benzofurans.

7. Charecterization of Spiro-Cyclopentane Benzofurans derivatives:

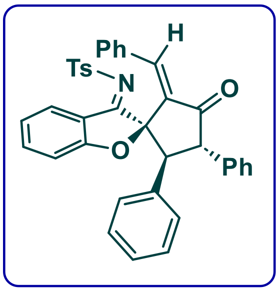

***N*-((2*R*,4'*S*,5'S,*E*)-2'-((*E*)-benzylidene)-3'-oxo-4',5'-diphenyl-3*H*-spiro[benzofuran-2,1'-cyclopentan]-3-ylidene)-4-methyl benzenesulfonamide (4a):** Pale yellow Solid, 53.5 mg, 90% yield, 92% *ee*, the measured *dr* is 7:1 from ^{1}H NMR: 4.65-4.62(*minor*), 4.54-4.52(*major*), E/Z = >20:1; R_f = 0.4 (petroleum ether/ethyl acetate = 90:10)

^{1}H NMR (500 MHz, $CDCl_3$): δ 8.32 – 8.17 (m, 1H), 8.01 (s, 1H), 7.52 (d, J = 7.9 Hz, 2H), 7.47-7.43 (m, 1H), 7.29 – 7.23 (m, 5H), 7.19 (t, J = 7.4 Hz, 5H), 7.08 (t, J = 7.7 Hz, 2H), 7.02 (t, J = 7.6 Hz, 3H), 6.99 – 6.93 (m, 3H), 6.91 (d, J = 7.7 Hz, 1H), 6.85 (d, J = 8.4 Hz, 1H), 4.53 (d, J = 13.9 Hz, 1H), 3.95 (d, J = 13.6 Hz, 1H), 2.45 (s, 3H). **^{13}C NMR (126 MHz, $CDCl_3$)** (*major*+*minor*): δ 201.28, 200.70, 181.73, 179.61, 171.22, 167.91, 144.06, 143.58, 140.81, 139.01, 138.95, 138.72, 138.51, 135.79, 135.77, 135.07, 134.61, 133.77, 133.14, 132.43, 132.35, 132.29, 132.13, 130.92, 130.80, 130.02, 129.92, 129.76, 129.47, 129.35, 129.22, 129.08, 129.05, 128.87, 128.81, 128.78, 128.64, 128.38, 128.35, 128.31, 128.17, 128.15, 128.08, 127.61, 127.19, 127.07, 122.65, 122.27, 121.05, 117.60, 112.69, 111.90, 95.20, 94.11, 61.67, 57.86, 56.62, 54.49, 22.86, 21.81.

HRMS (ESI$^+$) (*m/z*): $[M+Na]^+$ calcd for $C_{38}H_{29}NO_4S$: 618.1710, found: 618.1715;

HPLC: The enantiomeric excess was determined using CHIRALPAK IA column (*n*-Hexane/ *i*-PrOH = 90:30, flow rate = 1.0 mL/min, λ =254 nm, τ_{major} = 23.5 min, τ_{minor} = 49.7 min).

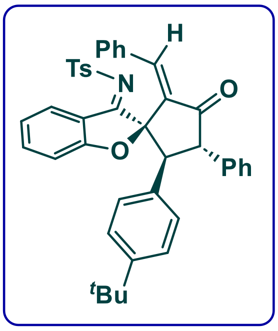

***N*-((2*R*,4'*S*,5'*S*,*E*)-2'-((*E*)-benzylidene)-5'-(4-(tert-butyl)phenyl)-3'-oxo-4'-phenyl-3*H*-spiro[benzofuran-2,1'-cyclopentan]-3-ylidene)-4-methylbenzenesulfonamide(4b):** Yellow solid, 54 mg, 83% yield, 6:1 *dr* and 85% *ee*, the measured *dr* is 6:1 from ^{1}H NMR: 4.65-4.62(*minor*), 4.54-4.51(*major*), E/Z = >20:1; R_f = 0.5 (petroleum ether/ethyl acetate = 90:10);

^{1}H NMR (500 MHz, Chloroform-d) δ 8.23 (s, 1H), 8.00 (s, 1H), 7.50 (d, J = 7.9 Hz, 2H), 7.39 (t, J = 7.8 Hz, 1H), 7.28 – 7.25 (m, 6H), 7.22 – 7.20 (m, 3H), 7.08 (d, J = 8.0 Hz, 3H), 7.01 (s, 1H), 6.99 – 6.96 (m, 3H), 6.89 (t, J = 7.7 Hz, 1H), 6.78 (d, J = 8.5 Hz, 1H), 4.53 (d, J = 13.8 Hz, 1H), 3.96 (s, 1H), 2.45 (s, 3H), 1.08 (s, 9H). **^{13}C NMR** (126 MHz, Chloroform-d) δ 201.55, 179.92, 167.89, 151.06, 143.52, 138.59, 136.01, 134.24, 133.85, 130.84, 129.91, 129.44, 129.15, 129.09, 128.81, 128.79, 128.70, 127.59, 127.20, 124.89, 122.04, 112.79, 94.31, 61.53, 56.27, 31.28, 21.81.

HRMS (ESI^{+}) (*m/z*): $[M+H]^{+}$ calcd for $C_{42}H_{37}NO_4S$: 652.2517, found: 652.2517;

HPLC: The enantiomeric excess was determined using CHIRALPAK IA column (*n*-Hexane/ *i*-PrOH = 90:30, flow rate = 1.0 mL/min, λ =254 nm, τ_{major} = 12.8 min, τ_{minor} = 21.0 min).

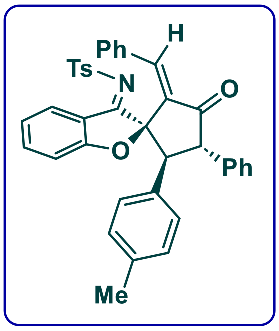

***N*-((2*R*,4'*S*,5'*S*,*E*)-2'-((*E*)-benzylidene)-3'-oxo-4'-phenyl-5'-(*p*-tolyl)-3*H*-spiro [benzofuran-2,1'-cyclopentan]-3-ylidene)-4-methylbenzenesulfonamide (4c):**Pale yellow solid, 51.7 mg, 85% yield, 87% *ee*, the measured *dr* is 5:1 from ^{1}H NMR: 4.63-4.59(*minor*), 4.51-4.48(*major*), E/Z = >20:1; R_f = 0.4 (petroleum ether/ethyl acetate = 90:10)

^{1}H NMR (400 MHz, Chloroform-d) δ 8.23 (s, 1H), 8.00 (s, 1H), 7.53 (d, J = 7.9 Hz, 2H), 7.50 – 7.44 (m, 1H), 7.27 (d, J = 7.1 Hz, 3H), 7.25 -7.22(m, 2H), 7.20-7.16 (m, 3H), 7.06 (d, J = 7.8 Hz, 4H), 6.93 (dd, J = 7.8, 5.2 Hz, 3H), 6.86 (d, J = 8.4 Hz, 1H), 6.82 (d, J = 7.8 Hz, 2H), 4.49 (d, J = 14.0 Hz, 1H), 3.94 (s, 1H), 2.45 (s, 3H), 2.08 (s, 3H). **^{13}C NMR** (101 MHz, Chloroform-d) δ 201.39, 179.75, 168.04, 150.00, 143.54, 143.43, 138.95, 138.65, 137.69, 135.95, 134.89, 133.84, 130.72, 129.74, 129.48, 129.08, 128.89, 128.79, 128.62, 127.56, 127.21, 122.25, 112.73, 94.23, 61.34, 56.82, 21.81, 21.13.

HRMS (ESI^{+}) (*m/z*): $[M+H]^{+}$ calcd for $C_{39}H_{31}NO_4S$: 610.2047, found: 610.2036;

HPLC: The enantiomeric excess was determined using CHIRALPAK IA column (*n*-Hexane/ *i*-PrOH=90:30, flow rate=1.0 mL/min, λ= 254 nm, τ_{major} = 28.5 min, τ_{minor} = 42.3 min).

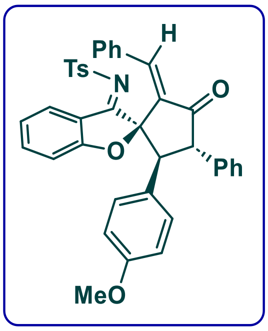

***N*-((2*R*,4'*S*,5'*S*,*E*)-2'-((*E*)-benzylidene)-5'-(4-methoxyphenyl)-3'-oxo-4'-phenyl-3*H*-spiro[benzofuran-2,1'-cyclopentan]-3-ylidene)-4-methylbenzenesulfonamide(4d):** Pale yellow solid**,** 55mg, 88% yield, 90% *ee*. The measured *dr* is 7:1 from ^{1}H NMR: 4.61-4.58 (*minor*), 4.48-4.44(*major*), E/Z = >20:1; R_f = 0.4 (petroleum ether/ethyl acetate = 90:10)

^{1}H NMR (400 MHz, CDCl$_3$): δ 8.25 (s, 1H), 8.00 (s, 1H), 7.57 – 7.44 (m, 3H), 7.25 (t, J = 9.3 Hz, 5H), 7.21 – 7.14 (m, 3H), 7.13 – 7.05 (m, 4H), 6.98 – 6.91 (m, 3H), 6.88 (d, J = 8.4 Hz, 1H), 6.57 (dd, J = 7.3, 4.9 Hz, 2H), 4.46 (d, J = 14.0 Hz, 1H), 3.91(s, 1H), 3.59 (s, 3H), 2.45 (s, 3H). **^{13}C NMR (101 MHz, CDCl$_3$)**: δ 201.35, 179.79, 168.01, 159.26, 143.55, 139.10, 138.56, 135.88, 134.75, 133.80, 130.75, 130.27, 129.74, 129.47, 129.06, 128.78, 128.62, 127.57, 127.17, 124.33, 122.29, 113.64, 112.71, 94.24, 61.15, 56.90, 55.24, 21.80.

HRMS (ESI$^+$) (*m/z*): [M+H]$^+$ calcd for $C_{39}H_{31}NO_5S$: 626.1996, found: 618.1997;

HPLC: The enantiomeric excess was determined using CHIRALPAK IA column (*n*-Hexane/ ^{i}PrOH=90:30, flow rate=1.0 mL/min, λ = 254 nm, τ_{major} = 32.0 min, τ_{minor} = 50.7 min).

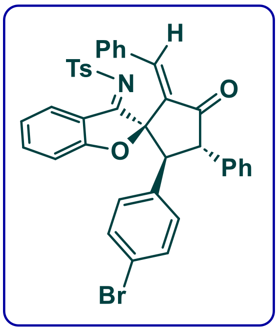

***N*-((2*R*,4'*S*,5'*S*,*E*)-2'-((*E*)-benzylidene)-5'-(4-bromophenyl)-3'-oxo-4'-phenyl-3*H*-spiro[benzofuran-2,1'-cyclopentan]-3-ylidene)-4-methylbenzenesulfonamide(4e):** Pale yellow solid, 60.6 mg, 90% yield, 90% *ee*, the measured *dr* is 7:1 from ^{1}H NMR: 4.59-4.56(*minor*), 4.47-4.44(*major*), E/Z = >20:1; R_f = 0.4 (petroleum ether/ethyl acetate = 90:10)

^{1}H NMR (500 MHz, Chloroform-d): δ 8.26 (s, 1H), 8.01 (s, 1H), 7.61 – 7.47 (m, 3H), 7.29 – 7.23 (m, 4H), 7.21 (m, 1H), 7.19 – 7.11 (m, 4H), 7.10 – 7.05 (m, 4H), 7.02 – 6.96 (m, 2H), 6.93 (d, J = 7.3Hz, 2H), 6.89 (d, J = 8.4 Hz, 1H), 4.45 (d, J = 13.9 Hz, 1H), 3.89 (d, J = 13.5 Hz, 1H), 2.45 (s, 3H). **^{13}C NMR** (126 MHz, Chloroform-d) (*major*+*minor*): δ 200.76, 179.21, 171.14, 167.79, 144.21, 143.91, 143.70, 141.11, 139.40, 139.28, 138.55, 138.35, 135.42, 134.83, 134.42, 133.66, 133.02, 132.34, 132.18, 131.61, 131.41, 131.10, 130.90, 130.85, 130.41, 129.72, 129.51, 129.30, 128.97, 128.92, 128.67, 128.19, 127.80, 127.19, 127.05, 122.97, 122.64, 122.43, 122.22, 120.75, 117.46, 112.70, 111.90, 94.90, 93.83, 61.15, 57.42, 56.74, 54.69, 21.83.

HRMS (ESI$^+$) (*m/z*): [M+H]$^+$ calcd for $C_{38}H_{28}BrNO_4S$: 674.0995, found: 674.0956;

HPLC: The enantiomeric excess was determined using CHIRALPAK IA column (*n*-Hexane/ ^{i}PrOH=90:30, flow rate=1.0 mL/min, λ= 274 nm, τ_{major} = 44.7 min, τ_{minor} = 80.2 min)

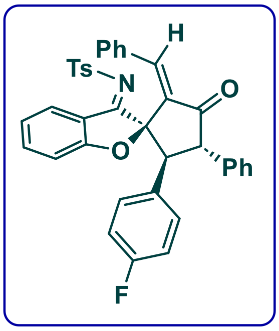

***N*-((2*R*,4'*S*,5'*S*,*E*)-2'-((*E*)-benzylidene)-5'-(4-fluorophenyl)-3'-oxo-4'-phenyl-3*H*-spiro [benzofuran-2,1'-cyclopentan]-3-ylidene)-4-methylbenzenesulfonamide(4f):** Pale yellow solid, 57 mg, 93% yield, 91% *ee*, the measured *dr* is 5:1 from ^{1}H NMR: 4.60-4.57(*minor*), 4.20-4.17(*major*), E/Z = >20:1; R_f = 0.4 (petroleum ether/ethyl acetate = 90:10)

^{1}H NMR (500 MHz, Chloroform-d) δ 8.26 (s, 1H), 8.01 (s, 1H), 7.53-7.49 (m, 3H), 7.31 – 7.27 (m, 4H), 7.25 (d, *J* = 5.3 Hz, 2H), 7.19-7.15 (m, 4H), 7.11 – 7.06 (m, 2H), 6.95 (t, *J* = 7.5 Hz, 3H), 6.88 (d, *J* = 8.4 Hz, 1H), 6.73 (t, *J* = 8.5 Hz, 2H), 4.45 (d, *J* = 14.0 Hz, 1H), 3.94 (s, 1H), 2.46 (s, 3H). **^{13}C NMR** (126 MHz, Chloroform-d) δ 200.88, 179.37, 167.83, 163.47, 161.50, 143.76, 143.68, 139.27, 138.45, 135.58, 134.47, 133.74, 130.87, 130.81, 129.97, 129.76, 129.51, 129.36, 129.02, 128.94, 128.89, 128.68, 128.26, 128.20, 127.75, 127.20, 127.05, 122.51, 115.32, 115.15, 112.66, 94.00, 61.01, 56.92, 21.82.

HRMS (ESI$^+$) (*m/z*): $[M+K]^+$ calcd for $C_{38}H_{28}FNO_4S$: 652.1355, found: 652.1352;

HPLC: The enantiomeric excess was determined using CHIRALPAK IA column (*n*-Hexane/ ^{i}PrOH=90:30, flow rate=1.0 mL/min, λ_{max}= 254 nm, τ_{major} = 32.6 min, τ_{minor} = 72.7 min).

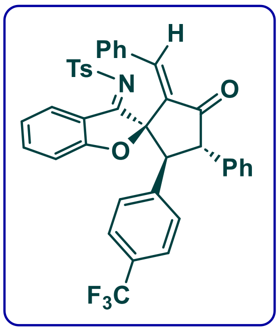

***N*-((2*R*,4'*S*,5'*S*,*E*)-2'-((*E*)-benzylidene)-3'-oxo-4'-phenyl-5'-(4-(trifluoromethyl)phenyl)-3*H*-spiro[benzofuran-2,1'-cyclopentan]-3-ylidene)-4-methylbenzenesulfonamide(4g):** Pale yellow solid, 56.3 mg, 85% yield, 92% *ee*, the measured *dr* is 7:1 from ^{1}H NMR: 4.65-4.62(*minor*), 4.54-4.51(*major*), E/Z = >20:1; R_f = 0.4 (petroleum ether/ethyl acetate = 90:10)

^{1}H NMR (500 MHz, Chloroform-d) δ 8.25 (s, 1H), 8.03 (s, 1H), 7.52 (d, *J* = 7.9 Hz, 2H), 7.47 (t, *J* = 7.8 Hz, 1H), 7.33 (d, *J* = 8.2 Hz, 2H), 7.28 (dd, *J* = 7.9 Hz, 7H), 7.22 (d, *J* = 7.1 Hz, 1H), 7.16 (d, *J* = 7.5 Hz, 2H), 7.08 (t, *J* = 7.6 Hz, 2H), 6.94 (d, *J* = 7.6 Hz, 3H), 6.84 (d, *J* = 8.4 Hz, 1H), 4.53 (d, *J* = 13.9 Hz, 1H), 4.01 (s, 1H), 2.45 (s, 3H). **^{13}C NMR** (126 MHz, Chloroform-d) δ 200.48, 178.99, 167.66, 144.08, 143.76, 139.30, 138.36, 135.32, 134.20, 133.65, 130.95, 130.45, 130.19, 129.76, 129.65, 129.53, 129.33, 129.24, 128.97, 128.70, 128.40, 128.22, 127.88, 127.21, 127.06, 125.12 , 122.88, 122.66, 112.64, 93.85, 61.35, 56.63, 21.81.

HRMS (ESI$^+$) (*m/z*): $[M+K]^+$ calcd for $C_{39}H_{28}F_3NO_4S$: 702.1323, found: 702.1259;

HPLC: The enantiomeric excess was determined using CHIRALPAK IA column (*n*-Hexane/ ^{i}PrOH=90:30, flow rate=1.0 mL/min, λ= 254 nm, τ_{major} = 39.4 min, τ_{minor} = 64.3 min).

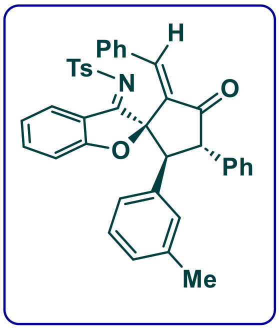

***N*-((2*R*,4'*S*,5'*S*,*E*)-2'-((*E*)-benzylidene)-3'-oxo-4'-phenyl-5'-(*m*-tolyl)-3*H*-spiro[benzofuran-2,1'-cyclopentan]-3-ylidene)-4-methylbenzenesulfonamide(4h):** Pale yellow solid, 54.8 mg, 90% yield, 92% *ee*, the measured *dr* is 6:1 from ^{1}H NMR: 4.62-4.58(*minor*), 4.51-4.48(*major*), E/Z = >20:1; R_f = 0.4 (petroleum ether/ethyl acetate = 90:10)

^{1}H NMR (400 MHz, Chloroform-d) δ 8.25 (d, J = 8.2 Hz, 1H), 8.02 (s, 1H), 7.54 (d, J = 8.1 Hz, 2H), 7.46 (d, J = 8.5, 1H), 7.28 (d, J = 8.2 Hz, 2H), 7.22 – 7.18 (m, 2H), 7.14 (t, J = 7.8 Hz, 2H), 7.11 – 7.06 (m, 3H), 7.05 – 6.98 (m, 6H), 6.97 – 6.94 (m, 3H), 6.85 (d, J = 8.4 Hz, 1H), 4.50 (d, J = 13.9 Hz, 1H), 3.97 (d, J = 13.7 Hz, 1H), 2.46 (s, 3H), 2.27 (s, 3H). **^{13}C NMR** (126 MHz, Chloroform-d) (*major+minor*) δ 201.37, 200.8, 181.83, 179.68, 171.21, 167.94, 143.98, 143.52, 140.71, 138.89, 138.83, 138.55, 137.94, 137.68, 135.86, 135.00, 134.52, 133.78, 133.15, 132.14, 130.85, 130.77, 130.21, 130.02, 129.88, 129.76, 129.44, 129.39, 129.04, 128.95, 128.83, 128.77, 128.71, 128.63, 128.13, 127.96, 127.55, 127.14, 127.01, 126.02, 125.97, 122.57, 122.16, 121.08, 117.63, 112.58, 111.80, 95.23, 94.17, 61.62, 57.78, 56.37, 54.34, 21.82, 21.78, 21.37, 21.30.

HRMS (ESI$^+$) (*m/z*): $[M+H]^+$ calcd for $C_{39}H_{31}NO_4S$: 610.2047, found: 610.2035;

HPLC: The enantiomeric excess was determined using CHIRALPAK IA column (*n*-Hexane/ ^{i}PrOH=90:30, flow rate=1.0 mL/min, λ = 254 nm, τ_{major} = 17.3 min, τ_{minor} = 23.9 min).

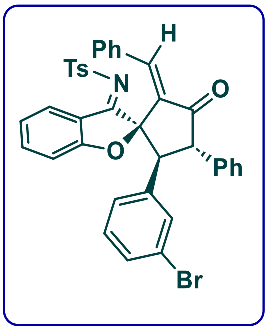

***N*-((2*R*,4'*S*,5'*S*,*E*)-2'-((*E*)-benzylidene)-5'-(3-bromophenyl)-3'-oxo-4'-phenyl-3*H*-spiro [benzofuran-2,1'-cyclopentan]-3-ylidene)-4-methylbenzenesulfonamide(4i):** pale yellow solid, 62 mg, 92% yield, 91% *ee*, the measured *dr* is 6:1 from ^{1}H NMR: 4.60-4.57(*minor*), 4.51-4.48(*major*), E/Z = >20:1; R_f = 0.4 (petroleum ether/ethyl acetate = 90:10)

^{1}H NMR (400 MHz, Chloroform-d) δ 8.33 (s, 1H), 8.06 (s, 1H), 7.58 – 7.54 (m, 2H), 7.50-7.49 (m, 1H), 7.36 – 7.29 (m, 5H), 7.21 (d, J = 7.5 Hz, 3H), 7.14 (dd, J = 9.6, 7.2 Hz, 3H), 7.11 – 7.05 (m, 2H), 7.00 (d, J = 7.8 Hz, 3H), 6.97 (d, J = 8.3 Hz, 1H), 6.92 (t, J = 7.8 Hz, 1H), 4.50 (d, J = 13.9 Hz, 1H), 3.94 (s, 1H), 2.50 (s, 3H). **^{13}C NMR** (101 MHz, Chloroform-d) (*major+minor*) δ 200.62, 200.1, 181.19, 179.04, 171.11, 167.75, 144.15, 144.10, 143.89, 143.68, 141.27, 139.26, 139.22, 138.61, 138.40, 135.42, 134.98, 134.95, 134.69, 134.16, 133.69, 132.34, 132.08, 131.84, 131.43, 131.22, 131.04, 130.92, 130.12, 129.98, 129.93, 129.90, 129.79, 129.51, 129.34, 129.02, 129.00, 128.93, 128.88, 128.70, 128.43, 128.38,

128.20, 127.81, 127.21, 127.18, 122.94, 122.56, 122.28, 117.51, 112.67, 111.91, 94.88, 93.86, 61.34, 57.41, 56.62, 54.33, 21.86, 21.82.

HRMS (ESI$^+$) (*m/z*): $[M+H]^+$ calcd for $C_{38}H_{28}BrNO_4S$: 674.0995, found: 674.1002;

HPLC: The enantiomeric excess was determined using Churalpak IA column (*n*-Hexane/ ^{i}PrOH=90:30, flow rate=1.0 mL/min, λ = 254 nm, τ_{major} = 29.4 min, τ_{minor} = 31.8 min).

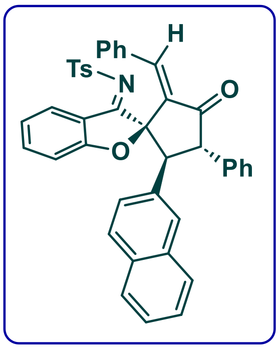

***N*-((2*R*,4'*S*,5'*S*,*E*)-2'-((*E*)-benzylidene)-5'-(naphthalen-2-yl)-3'-oxo-4'-phenyl-3*H*-spiro [benzofuran-2,1'-cyclopentan]-3-ylidene)-4-methylbenzenesulfonamide(4j):** Yellow solid, 34.1 mg, 89% yield, 93% *ee*, the measured *dr* is 5:1 from ^{1}H NMR: 4.84-4.81(*minor*), 4.73-4.70(*major*), E/Z = >20:1; R_f = 0.4 (petroleum ether/ethyl acetate = 90:10)

^{1}H NMR (500 MHz, Chloroform-d) δ 8.23 (s, 1H), 8.09 (s, 1H), 7.70 (d, *J* = 10.3 Hz, 2H), 7.63 (t, *J* = 7.7 Hz, 3H), 7.57 (d, *J* = 8.4 Hz, 1H), 7.41 (d, *J* = 8.7 Hz, 2H), 7.42-7.33 (m, 5H), 7.32 – 7.27 (m, 3H), 7.23 – 7.16 (m, 2H), 7.12 (t, *J* = 7.8 Hz, 2H), 7.00 (d, *J* = 7.7 Hz, 2H), 6.82 (t, *J* = 8.8 Hz, 2H), 4.71 (d, *J* = 13.9 Hz, 1H), 4.29 – 4.09 (m, 1H), 2.51 (s, 3H). **^{13}C NMR** (101 MHz, Chloroform-d) (*major*+minor) δ 201.21, 200.66, 179.66, 167.84, 144.08, 143.61, 140.84, 138.97, 138.60, 135.78, 134.76, 133.79, 133.00, 132.94, 132.33, 130.79, 130.36, 130.03, 129.96, 129.89, 129.75, 129.52, 129.35, 129.04, 129.02, 128.88, 128.83, 128.68, 128.64, 128.17, 128.11, 127.82, 127.63, 127.49, 127.23, 127.10, 126.55, 126.19, 126.11, 126.05, 122.68, 122.29, 112.48, 111.85, 95.36, 94.36, 62.80, 58.22, 56.78, 54.97, 22.90, 21.83.

HRMS (ESI$^+$) (*m/z*): $[M+Na]^+$ calcd for $C_{42}H_{31}NO_4S$: 668.1866, found: 668.1849;

HPLC: The enantiomeric excess was determined using CHIRALPAK IA column (*n*-Hexane/ ^{i}PrOH=90:30, flow rate=1.0 mL/min, λ = 254 nm, τ_{major} = 33.0 min, τ_{minor} = 61.1 min).

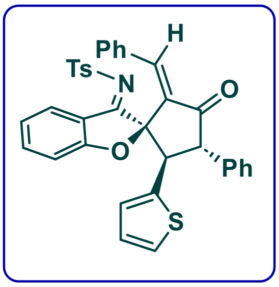

***N*-((2*R*,4'*S*,5'*R*,*E*)-2'-((*E*)-benzylidene)-3'-oxo-4'-phenyl-5'-(thiophen-2-yl)-3*H*-spiro [benzofuran-2,1'-cyclopentan]-3-ylidene)-4-methylbenzenesulfonamide(4k):** yellow solid, 52.2 mg, 87% yield, 85% *ee* (98% *ee* after crystallization), the measured *dr* is 5:1 from ^{1}H NMR: 4.53-4.49(*minor*), 4.37-4.33(*major*), E/Z = >20:1; R_f = 0.4 (petroleum ether/ethyl acetate = 90:10)

^{1}H NMR (400 MHz, Chloroform-d) δ 8.30 (s, 1H), 8.01 (s, 1H), 7.59 – 7.48 (m, 3H), 7.31 – 7.26 (m, 4H), 7.26 – 7.18 (m, 4H), 7.10 (q, *J* = 7.6, 6.6 Hz, 2H), 7.04 – 6.93 (m, 5H), 6.69 (d,

J = 3.4 Hz, 1H), 6.63 – 6.55 (m, 1H), 4.35 (d, *J* = 13.6 Hz, 1H), 4.18 (s, 1H), 2.45 (s, 3H). **^{13}C NMR** (101 MHz, Chloroform-d) δ 200.46, 179.34, 168.09, 153.64, 143.78, 143.61, 139.07, 138.54, 135.69, 135.30, 134.54, 133.73, 130.84, 129.75, 129.49, 129.12, 128.85, 128.66, 128.40, 128.15, 127.80, 127.22, 126.60, 125.62, 122.49, 113.02, 93.68, 59.19, 57.48, 21.81.

HRMS (ESI$^+$) (*m/z*): $[M+H]^+$ calcd for $C_{36}H_{27}NO_4S_2$: 602.1455, found: 602.1460;

HPLC: The enantiomeric excess was determined using CHIRALPAK IA column (*n*-Hexane/ ^{i}PrOH=90:30, flow rate=1.0 mL/min, λ = 254 nm, τ_{major} = 23.4 min, τ_{minor} = 64.6 min).

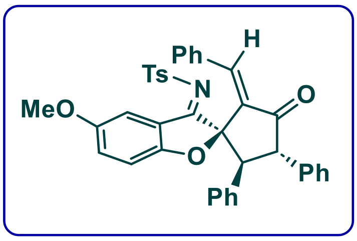

***N*-((2*R*,4'*S*,5'*S*,*E*)-2'-((*E*)-benzylidene)-6-methoxy-3'-oxo-4',5'-diphenyl-3*H*-spiro [benzofuran-2,1'-cyclopentan]-3-ylidene)-4-methylbenzenesulfonamide(4l):** Pale yellow solid, 55.6 mg, 89% yield, 93% *ee*, the measured *dr* is 8:1 from ^{1}H NMR: 4.66-4.63(*minor*), 4.53-4.49(*major*), E/Z = >20:1; R_f = 0.4 (petroleum ether/ethyl acetate = 90:10)

^{1}H NMR (400 MHz, Chloroform-d) (*major*) δ 8.00 (s, 1H), 7.68 (s, 1H), 7.53 (d, *J* = 7.9 Hz, 2H), 7.26 (dd, *J* = 9.3, 7.5 Hz, 5H), 7.22 – 7.16 (m, 5H), 7.10 (ddd, *J* = 7.9, 4.9, 2.0 Hz, 3H), 7.07 – 7.02 (m, 2H), 7.02 – 6.99 (m, 1H), 6.98 – 6.94 (m, 2H), 6.76 (d, *J* = 9.1 Hz, 1H), 4.51 (d, *J* = 13.9 Hz, 1H), 3.95 (s, 1H), 3.74 (s, 3H), 2.45 (s, 3H). **^{13}C NMR** (101 MHz, Chloroform-d) δ 201.32, 179.84, 163.48, 154.62, 143.51, 138.66, 135.93, 134.69, 133.77, 132.52, 132.29, 130.72, 129.82, 129.66, 129.48, 129.23, 129.08, 128.80, 128.61, 128.38, 128.17, 128.10, 127.59, 127.14, 113.42, 94.72, 61.70, 56.80, 56.11, 21.80.

HRMS (ESI$^+$) *m/z*: $[M+H]^+$ calcd for $C_{39}H_{31}NO_5S$: 626.1996, found: 626.2002;

HPLC: The enantiomeric excess was determined using CHIRALPAK IA column (*n*-Hexane/ ^{i}PrOH=90:30, flow rate=1.0 mL/min, λ = 254 nm, τ_{major} = 41.0 min, τ_{minor} = 53.8 min).

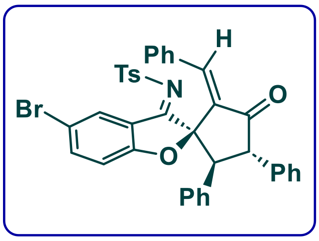

***N*-((2*R*,4'*S*,5'*S*,*E*)-2'-((*E*)-benzylidene)-6-bromo-3'-oxo-4',5'-diphenyl-3*H*-spiro [benzofuran-2,1'-cyclopentan]-3-ylidene)-4-methylbenzenesulfonamide(4m):** yellow solid, 61.3 mg, 91% yield, 93% *ee*, the measured *dr* is 7:1 from ^{1}H NMR: 4.61-4.58(*minor*), 4.52-4.49(*major*), E/Z = >20:1; R_f = 0.4 (petroleum ether/ethyl acetate = 90:10)

^{1}H NMR (500 MHz, Chloroform-d) δ 8.11 (s, 1H), 8.02 (s, 1H), 7.51 (d, *J* = 7.9 Hz, 2H), 7.25-7.28 (d, *J* = 8.3 Hz, 3H), 7.26-7.24 (m, 2H), 7.19 (dd, *J* = 13.1, 7.0 Hz, 5H), 7.15 – 7.10 (m, 3H), 7.05 (q, *J* = 6.3 Hz, 4H), 6.93 (d, *J* = 7.6 Hz, 2H), 4.51 (d, *J* = 13.9 Hz, 1H), 3.94 (d, *J* =

14.1 Hz, 1H), 2.46 (s, 3H). **^{13}C NMR** (126 MHz, Chloroform-d) δ 200.98, 178.17, 167.83, 144.01, 143.83, 138.12, 135.58, 134.31, 133.88, 133.73, 132.06, 130.93, 129.65, 129.54, 129.16, 129.02, 128.84, 128.75, 128.38, 128.34, 127.68, 127.23, 126.03, 116.08, 95.02, 62.00 – 61.24 (m), 56.46, 21.84.

HRMS (ESI$^+$) *m/z*: $[M+H]^+$ calcd for $C_{38}H_{28}BrNO_4S$: 674.0995, found: 674.0978;

HPLC: The enantiomeric excess was determined using CHIRALPAK IE column (*n*-Hexane/ ^{i}PrOH=90:30, flow rate=1.0 mL/min, λ = 254 nm, τ_{major} = 16.1 min, τ_{minor} = 60.2 min).

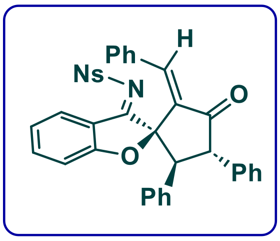

***N*-((2*R*,4'*S*,5'*S*,*E*)-2'-((*E*)-benzylidene)-3'-oxo-4',5'-diphenyl-3*H*-spiro[benzofuran-2,1'-cyclopentan]-3-ylidene)-4-nitrobenzenesulfonamide(4n):** Pale yellow solid, 56.3 mg, 90% yield, 88% *ee*, the measured *dr* is 8:1 from ^{1}H NMR: 4.29-4.26(*minor*), 3.94(*major*), E/Z = >20:1; R_f = 0.4 (petroleum ether/ethyl acetate = 90:10)

^{1}H NMR (500 MHz, Chloroform-d) δ 8.32 (d, *J* = 8.5 Hz, 2H), 8.18 (d, *J* = 8.0 Hz, 1H), 8.02 (s, 1H), 7.77 (d, *J* = 8.5 Hz, 2H), 7.53 (t, *J* = 7.8 Hz, 1H), 7.34 (t, *J* = 7.7 Hz, 1H), 7.25 (t, *J* = 7.4 Hz, 3H), 7.21 – 7.19 (m, 2H), 7.17 (d, *J* = 7.7 Hz, 2H), 7.12 (t, *J* = 7.6 Hz, 2H), 7.05 (t, *J* = 7.5 Hz, 2H), 7.00 (d, *J* = 7.6 Hz, 1H), 6.98 – 6.95 (m, 2H), 6.91 (d, *J* = 8.4 Hz, 1H), 4.55 (d, *J* = 14.0 Hz, 1H), 3.94 (s, 1H). **^{13}C NMR** (126 MHz, Chloroform-d) δ 200.82, 181.58, 168.40, 150.31, 146.86, 143.49, 139.97, 135.59, 134.07, 133.67, 132.32, 132.08, 131.11, 129.87, 129.18, 128.97, 128.87, 128.83, 128.53, 128.49, 128.47, 128.32, 128.30, 127.75, 124.20, 122.66, 113.06, 94.48, 62.00, 56.56.

HRMS (ESI$^+$) *m/z*: $[M+Na]^+$ calcd for $C_{37}H_{26}N_2O_6S$: 649.1404, found: 649.1410;

HPLC: The enantiomeric excess was determined using CHIRALPAK IA column (*n*-Hexane/ ^{i}PrOH=90:30, flow rate=1.0 mL/min, λ = 274 nm, τ_{major} = 14.8 min, τ_{minor} = 21.8 min).

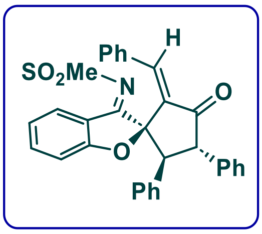

***N*-((2*R*,4'*S*,5'*S*,*E*)-2'-((*E*)-benzylidene)-3'-oxo-4',5'-diphenyl-3*H*-spiro[benzofuran-2,1'-cyclopentan]-3-ylidene)methanesulfonamide(4o):** Pale yellow solid, 43 mg, 83% yield, 89% *ee*, the measured *dr* is 7:1 from ^{1}H NMR: 5.11-5.08(*minor*), 4.62-4.60(*major*), E/Z = >20:1; R_f = 0.4 (petroleum ether/ethyl acetate = 90:10)

^{1}H NMR (500 MHz, Chloroform-d) δ 8.17 (s, 1H), 8.08 (d, *J* = 8.2 Hz, 1H), 7.51-7.47 (m, 1H), 7.33 (d, *J* = 7.5 Hz, 2H), 7.31 – 7.26 (m, 4H), 7.26 – 7.22 (m, 2H), 7.11 (t, *J* = 7.6 Hz,

2H), 7.08 – 7.03 (m, 4H), 7.03 – 6.99 (m, 1H), 6.93 (t, J = 7.7 Hz, 1H), 6.89 (d, J = 8.4 Hz, 1H), 4.61 (d, J = 14.0 Hz, 1H), 4.06 (d, J = 14.0 Hz, 1H), 2.93 (s, 3H). **^{13}C NMR** (126 MHz, Chloroform-d) δ 201.21, 180.28, 167.90, 143.80, 139.15, 135.76, 134.98, 134.00, 133.37, 132.27, 131.18, 130.80, 130.05, 129.49, 129.45, 129.18, 129.09, 129.07, 129.04, 128.88, 128.78, 128.74, 128.64, 128.20, 128.13, 127.70, 127.45, 124.91, 123.78, 122.27, 120.84, 120.12, 112.73, 94.02, 61.80, 56.65, 42.56.

HRMS (ESI$^+$) *m/z*: [M+H]$^+$ calcd for $C_{32}H_{25}NO_4S$: 520.1578, found: 520.1579;

HPLC: The enantiomeric excess was determined using CHIRALPAK IE column (*n*-Hexane/ ^{i}PrOH=90:30, flow rate=1.0 mL/min, λ = 274 nm, τ_{major} = 13.8 min, τ_{minor} = 16.4 min).

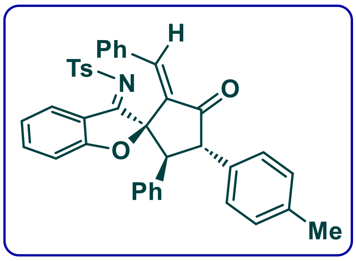

***N*-((2*R*,4'*S*,5'*S*,*E*)-2'-((*E*)-benzylidene)-3'-oxo-5'-phenyl-4'-(p-tolyl)-3*H*-spiro [benzofuran-2,1'-cyclopentan]-3-ylidene)-4-methylbenzenesulfonamide(4p):** Pale yellow solid, 55.4 mg, 91% yield, 89% *ee*, the measured *dr* is 10:1 from ^{1}H NMR: 4.57-4.55(*minor*), 4.46-4.44(*major*), E/Z = >20:1; R_f = 0.4 (petroleum ether/ethyl acetate = 90:10)

^{1}H NMR (500 MHz, Chloroform-d) δ 8.19 (s, 1H), 7.96 (s, 1H), 7.49 (d, J = 7.9 Hz, 2H), 7.41 (t, J = 7.8 Hz, 1H), 7.24-7.22 (m, 4H), 7.15 (d, J = 7.5 Hz, 2H), 7.05-7.02 (m, 5H), 6.98 (t, J = 7.5 Hz, 2H), 66.94-6.86 (m, 4H), 6.80 (d, J = 8.4 Hz, 1H), 4.45 (d, J = 14.0 Hz, 1H), 3.90 (s, 1H), 2.42 (s, 3H), 2.21 (s, 3H). **^{13}C NMR** (126 MHz, Chloroform-d) δ 201.50, 179.69, 167.98, 143.56, 143.47, 138.96, 138.63, 137.25, 134.74, 133.86, 132.79, 132.49, 130.74, 129.91, 129.76, 129.58, 129.48, 129.34, 129.28, 128.91, 128.64, 128.17, 128.05, 127.22, 127.11, 122.25, 112.69, 94.17, 61.74, 56.40, 21.81, 21.27.

HRMS (ESI$^+$) *m/z*: [M+H]$^+$ calcd for $C_{39}H_{31}NO_4S$: 610.2047, found: 610.2056;

HPLC: The enantiomeric excess was determined using CHIRALPAK IA column (*n*-Hexane/ ^{i}PrOH=90:30, flow rate=1.0 mL/min, λ = 254 nm, τ_{major} = 23.3 min, τ_{minor} = 38.3 min).

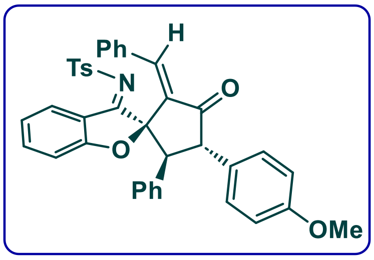

***N*-((2*R*,4'*S*,5'*S*,*E*)-2'-((*E*)-benzylidene)-4'-(4-methoxy phenyl)-3'-oxo-5'-phenyl-3*H*-spiro[benzofuran-2,1'-cyclopentan]-3-ylidene)-4 methylbenzenesulfonamide(4q):** Pale yellow solid, 58.1 mg, 93% yield, 90% *ee*, the measured *dr* is 10:1 from ^{1}H NMR: 4.61-4.59(*minor*), 4.50-4.47(*major*), E/Z = >20:1; R_f = 0.4 (petroleum ether/ethyl acetate = 90:10).

^{1}**H NMR** (500 MHz, Chloroform-d) *δ* 8.25 (s, 1H), 8.02 (s, 1H), 7.54 (d, *J* = 7.9 Hz, 2H), 7.46 (t, *J* = 7.8 Hz, 1H), 7.28 (d, *J* = 8.1 Hz, 3H), 7.20 (d, *J* = 7.5 Hz, 2H), 7.10 (t, *J* = 8.4 Hz, 3H), 7.03 (t, *J* = 7.5 Hz, 3H), 6.99-6.91 (m, 4H), 6.85 (d, *J* = 8.4 Hz, 1H), 6.79 (d, *J* = 8.2 Hz, 2H), 4.49 (d, *J* = 14.0 Hz, 1H), 3.89 (d, *J* = 27.8 Hz, 1H), 3.72 (s, 3H), 2.46 (s, 3H). ^{13}**C NMR** (126 MHz, Chloroform-d) *δ* 201.65, 179.67, 167.93, 159.03, 143.56, 138.98, 138.55, 134.62, 133.80, 132.46, 130.75, 130.05, 129.75, 129.46, 129.25, 128.62, 128.16, 128.05, 127.82, 127.18, 114.34, 112.68, 94.08, 61.80, 55.95, 55.36, 21.79.

HRMS (ESI$^+$) *m/z*: [M+H]$^+$ calcd for $C_{39}H_{31}NO_5S$: 626.1996, found: 626.1996;

HPLC: The enantiomeric excess was determined using CHIRALPAK IA column (*n*-Hexane/ ^{i}PrOH=90:30, flow rate=1.0 mL/min, λ = 254 nm, τ_{major} = 37.8 min, τ_{minor} = 68.8 min).

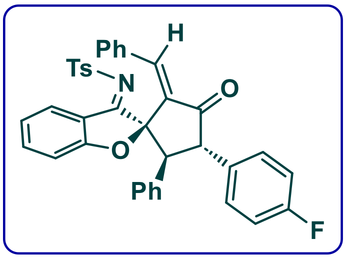

***N*-((2*R*,4'*S*,5'*S*,*E*)-2'-((*E*)-benzylidene)-4'-(4-fluorophenyl)-3'-oxo-5'-phenyl-3*H*-spiro [benzofuran-2,1'-cyclopentan]-3-ylidene)-4-methylbenzenesulfonamide(4r):** Pale yellow solid, 55.1 mg, 90% yield, 91% *ee*, the measured *dr* is 7:1 from ^{1}H NMR: 4.64-4.62(*minor*), 4.53-4.51(*major*), E/Z = >20:1; R_f = 0.4 (petroleum ether/ethyl acetate = 90:10)

^{1}**H NMR** (600 MHz, Chloroform-d) *δ* 8.25 (d, *J* = 7.3 Hz, 1H), 8.02 (s, 1H), 7.52 (d, *J* = 7.9 Hz, 2H), 7.48 – 7.45 (m, 1H), 7.32 – 7.26 (m, 3H), 7.18 (d, *J* = 7.6 Hz, 2H), 7.15 (t, *J* = 6.2 Hz, 2H), 7.11 – 7.07 (m, 2H), 7.05 – 7.01 (m, 3H), 6.99 (d, *J* = 7.3 Hz, 1H), 6.96 – 6.93 (m, 4H), 6.86 (d, *J* = 8.4 Hz, 1H), 4.52 (d, *J* = 13.9 Hz, 1H), 3.89 (s, 1H), 2.45 (s, 3H). ^{13}**C NMR** (151 MHz, Chloroform-d) δ 201.05, 167.90, 163.10, 161.47, 143.64, 139.06, 138.47, 134.38, 133.69, 132.17, 131.43, 130.89, 130.64, 130.58, 129.79, 129.48, 129.18, 128.67, 128.26, 128.21, 127.20, 122.34, 115.86, 115.71, 112.68, 93.97, 61.75, 55.85, 21.81.

HRMS (ESI$^+$) (*m/z*): [M+Na]$^+$ calcd for $C_{38}H_{28}FNO_4S$: 636.1616, found: 636.1602;

HPLC: The enantiomeric excess was determined using CHIRALPAK IA column (*n*-Hexane/ ^{i}PrOH=90:30, flow rate=1.0 mL/min, λ = 254 nm, τ_{major} = 28.9 min, τ_{minor} = 51.3 min).

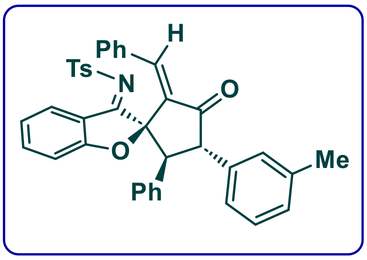

***N*-((2*R*,4'*S*,5'*S*,*E*)-2'-((*E*)-benzylidene)-3'-oxo-5'-phenyl-4'-(m-tolyl)-3*H*-spiro [benzofuran-2,1'-cyclopentan]-3-ylidene)-4-methylbenzenesulfonamide(4s):** Pale yellow solid, 55.4 mg, 91% yield, 92% *ee*, the measured *dr* is 6:1 from ^{1}H NMR: 4.62-4.58(*minor*), 4.51-4.48(*major*), E/Z = >20:1; R_f = 0.4 (petroleum ether/ethyl acetate = 90:10)

^{1}H NMR (400 MHz, Chloroform-d) δ 8.25 (d, J = 8.2 Hz, 1H), 8.02 (s, 1H), 7.54 (d, J = 8.1 Hz, 2H), 7.48-7.43 (m, 1H), 7.43 – 7.39 (m, 1H), 7.28 (d, J = 8.2 Hz, 3H), 7.20 (d, J = 7.0 Hz, 2H), 7.14 (t, J = 7.8 Hz, 2H), 7.11 – 7.06 (m, 3H), 7.05-6.99 (m, 6H), 6.98 – 6.94 (m, 4H), 6.92 (d, J = 7.7 Hz, 1H), 6.85 (d, J = 8.4 Hz, 1H), 4.50 (d, J = 13.9 Hz, 1H), 3.97 (d, J = 13.7 Hz, 1H), 2.46 (s, 3H), 2.27 (s, 3H). **^{13}C NMR** (101 MHz, Chloroform-d) (*major+minor*) δ 201.51, 200.96, 181.73, 179.69, 171.23, 167.94, 144.02, 143.57, 140.72, 138.99, 138.92, 138.74, 138.54, 138.44, 138.37, 138.32, 135.76, 135.10, 134.63, 133.79, 133.18, 132.50, 132.41, 132.31, 132.14, 130.92, 130.77, 129.99, 129.91, 129.85, 129.76, 129.62, 129.46, 129.35, 129.24, 129.00, 128.78, 128.74, 128.68, 128.63, 128.50, 128.33, 128.26, 128.14, 128.03, 127.18, 127.08, 126.15, 126.02, 122.62, 122.25, 112.69, 111.90, 95.24, 94.15, 61.71, 57.94, 56.70, 54.55, 21.84, 21.80, 21.72, 21.64.

HRMS (ESI$^+$) *m/z*: [M+H]$^+$ calcd for $C_{39}H_{31}NO_4S$: 610.2047, found: 610.2054;

HPLC: The enantiomeric excess was determined using CHIRALPAK IA column (*n*-Hexane/ ^{i}PrOH=90:30, flow rate=1.0 mL/min, λ = 254 nm, τ_{major} = 21.2 min, τ_{minor} = 39.5 min).

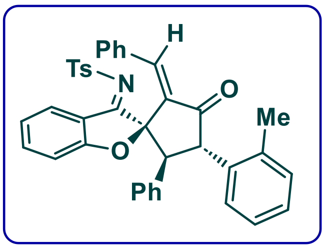

***N*-((2*R*,4'*S*,5'*S*,*E*)-2'-((*E*)-benzylidene)-3'-oxo-5'-phenyl-4'-(o-tolyl)-3*H*-spiro [benzofuran-2,1'-cyclopentan]-3-ylidene)-4-methylbenzenesulfonamide(4t):** yellow solid, 54.2 mg, 89% yield, 92% *ee*, the measured *dr* is 7:1 from ^{1}H NMR: 5.02-4.99(*minor*), 4.80-4.77(*major*), E/Z = >20:1; R_f = 0.4 (petroleum ether/ethyl acetate = 90:10)

^{1}H NMR (500 MHz, Chloroform-d) (*major*) δ 8.24 (s, 1H), 7.99 (s, 1H), 7.55 (d, J = 7.9 Hz, 2H), 7.46 (t, J = 7.8 Hz, 1H), 7.28 (d, J = 8.0 Hz, 3H), 7.18 (d, J = 7.5 Hz, 2H), 7.14 (d, J = 7.4 Hz, 1H), 7.10 – 7.05 (m, 4H), 7.01 (t, J = 7.1 Hz, 3H), 6.95 (dd, J = 11.8, 7.6 Hz, 4H), 6.88 (d, J = 8.4 Hz, 1H), 4.79 (d, J = 14.1 Hz, 1H), 4.09 (d, J = 14.3 Hz, 1H), 2.46 (s, 3H), 2.40 (s, 3H). **^{13}C NMR** (126 MHz, Chloroform-d) (*major+minor*) δ 201.00, 199.97, 181.45, 179.69, 171.28, 167.98, 144.06, 143.58, 140.46, 139.04, 138.84, 138.53, 134.95, 134.74, 134.38, 133.80, 133.66, 133.19, 132.45, 132.32, 130.96, 130.82, 130.75, 129.87, 129.84, 129.69, 129.48, 129.26, 129.22, 128.75, 128.63, 128.33, 128.14, 128.10, 127.56, 127.20, 127.16, 126.44, 122.71, 122.30, 117.53, 112.68, 111.95, 95.23, 94.24, 60.89, 56.22, 21.83, 20.46.

HRMS (ESI+) *m/z*: [M+H]+ calcd for C39H31NO4S: 610.2047, found: 610.2054;

HPLC: The enantiomeric excess was determined using CHIRALPAK IA column (*n*-Hexane/ ^{i}PrOH=90:30, flow rate=1.0 mL/min, λ = 254 nm, τ_{major} = 20.1 min, τ_{minor} = 32.5 min).

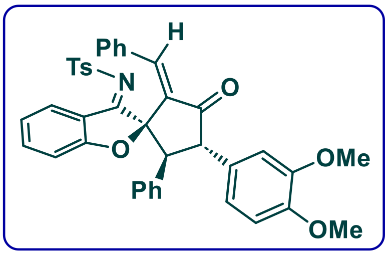

***N*-((2*R*,4'*S*,5'*S*,*E*)-2'-((*E*)-benzylidene)-4'-(3,4-dimethoxy phenyl)-3'-oxo-5'-phenyl-3*H*-spiro[benzofuran-2,1'-cyclopentan]-3-ylidene)-4-methylbenzenesulfonamide (4u):** Yellow solid, 58.9 mg, 90% yield, 95% *ee*, the measured *dr* is 7:1 from ^{1}H NMR: 4.61-4.57(*minor*), 4.49-4.45(*major*), E/Z = >20:1; R_f = 0.4 (petroleum ether/ethyl acetate = 90:10)

^{1}H NMR (400 MHz, Chloroform-d) δ 8.25 (d, J = 8.2 Hz, 1H), 8.02 (s, 1H), 7.53 (d, J = 8.0 Hz, 2H), 7.48 – 7.42 (m, 1H), 7.29 – 7.23 (m, 3H), 7.22 – 7.17 (m, 2H), 7.10 – 7.00 (m, 5H), 6.99-6.64 (m, 3H), 6.91 (t, J = 7.6 Hz, 1H), 6.84 (d, J = 8.4 Hz, 1H), 6.75 – 6.70 (m, 2H), 4.47 (d, J = 13.9 Hz, 1H), 3.92 (s, 1H), 3.78 (s, 3H), 3.76 (s, 3H), 2.44 (s, 3H). **^{13}C NMR** (101 MHz, Chloroform-d) δ 201.42, 179.68, 167.92, 149.12, 148.54, 143.59, 139.00, 138.53, 134.61, 133.74, 132.56, 132.27, 132.23, 130.74, 129.73, 129.45, 129.24, 128.80, 128.76, 128.65, 128.59, 128.35, 128.33, 128.17, 128.07, 127.14, 122.26, 121.19, 112.66, 112.38, 111.49, 93.99, 62.82, 56.20, 56.02, 55.96, 21.76.

HRMS (ESI$^+$) *m/z*: [M+Na]$^+$ calcd for $C_{40}H_{33}NO_6S$: 678.1921, found: 678.1928;

HPLC: The enantiomeric excess was determined using CHIRALPAK IA column (*n*-Hexane/ ^{i}PrOH=90:30, flow rate=1.0 mL/min, λ = 254 nm, τ_{major} = 36.4 min, τ_{minor} = 63.1 min).

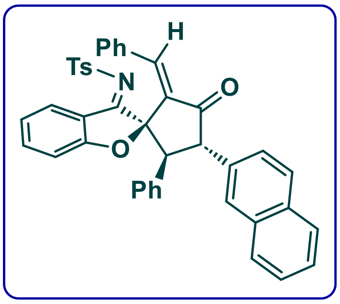

***N*-((2*R*,4'*S*,5'*S*,*E*)-2'-((*E*)-benzylidene)-4'-(naphthalen-2-yl)-3'-oxo-5'-phenyl-3*H*-spiro [benzofuran-2,1'-cyclopentan]-3-ylidene)-4-methylbenzenesulfonamide(4v):** Yellow solid, 59.3 mg, 92% yield, 91% *ee*, the measured *dr* is 6:1 from ^{1}H NMR: 4.85-4.81(*minor*), 4.74-4.71(*major*), E/Z = >20:1; R_f = 0.4 (petroleum ether/ethyl acetate = 90:10)

^{1}H NMR (400 MHz, Chloroform-d) δ 8.29 (d, J = 8.2 Hz, 1H), 8.07 (s, 1H), 7.78-7.73 (m, 3H), 7.65 (s, 1H), 7.57 (d, J = 8.1 Hz, 2H), 7.52 – 7.46 (m, 1H), 7.45 – 7.40 (m, 2H), 7.34-7.28 (m, 4H), 7.25 (d, J = 7.2 Hz, 2H), 7.11 (t, J = 7.6 Hz, 2H), 7.06-6.97 (m, 5H), 6.97-6.93 (m, 1H), 6.90 (d, J = 8.4 Hz, 1H), 4.72 (d, J = 13.9 Hz, 1H), 4.11 (s, 1H), 2.48 (s, 3H). **^{13}C NMR** (101 MHz, Chloroform-d) δ 201.36, 179.64, 167.98, 143.72, 143.62, 140.96, 139.03, 138.57, 134.67, 133.79, 133.57, 133.38, 132.92, 132.34, 130.84, 129.82, 129.51, 129.26, 128.67, 128.61, 128.54, 128.21, 128.13, 128.00, 127.80, 127.24, 126.57, 126.22, 126.09, 122.31, 112.72, 94.21, 61.73, 56.99, 21.82.

HRMS (ESI$^+$) (*m/z*): [M+H]$^+$ calcd for $C_{42}H_{31}NO_4S$: 646.2047, found: 646.2046;

HPLC: The enantiomeric excess was determined using CHIRALPAK IA column (*n*-Hexane/ ^{i}PrOH=90:30, flow rate=1.0 mL/min, λ = 254 nm, τ_{major} = 40.9 min, τ_{minor} = 66.4 min).

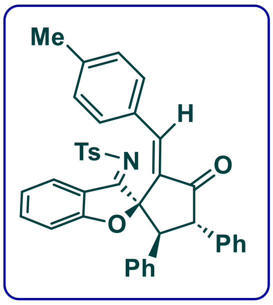

4-methyl-*N*-((2*R*,4'*S*,5'*S*,*E*)-2'-((*E*)-4-methylbenzylidene)-3'-oxo-4',5'-diphenyl-3*H*-spiro [benzofuran-2,1'-cyclopentan]-3-ylidene)benzenesulfonamide(4w): Pale yellow solid, 56.6 mg, 93% yield, 91% *ee*, the measured *dr* is 6:1 from ^{1}H NMR: 4.64-4.61(*minor*), 4.52-4.49(*major*), E/Z = >20:1; R_f = 0.4 (petroleum ether/ethyl acetate = 90:10)

^{1}**H NMR** (500 MHz, Chloroform-d) δ 8.26 (s, 1H), 7.97 (s, 1H), 7.50 (d, *J* = 7.9 Hz, 2H), 7.44 (t, *J* = 7.9 Hz, 1H), 7.24 (t, *J* = 7.3 Hz, 4H), 7.19 (d, *J* = 8.2 Hz, 5H), 7.02 (t, *J* = 7.5 Hz, 2H), 6..97-6.63 (m, 2H), 6.89-6.86 (m, 3H), 6.85 – 6.81 (m, 1H), 4.51 (d, *J* = 13.9 Hz, 1H), 3.97 (s, 1H), 2.44 (s, 3H), 2.28 (s, 3H). ^{13}**C NMR** (126 MHz, Chloroform-d) (*major*+*minor*) δ 201.34, 179.79, 168.05, 143.79, 143.50, 141.65, 138.93, 138.60, 135.97, 133.16, 132.50, 130.85, 130.18, 129.43, 129.29, 129.11, 129.08, 128.84, 128.79, 128.15, 128.05, 127.56, 127.16, 122.23, 112.71, 94.41, 61.89, 56.64, 21.80, 21.74.

HRMS (ESI^{+}) *m/z*: $[M+H]^{+}$ calcd for $C_{39}H_{31}NO_4S$: 610.2047, found: 610.2055;

HPLC: The enantiomeric excess was determined using CHIRALPAK IE column (*n*-Hexane/ ^{i}PrOH=90:30, flow rate=1.0 mL/min, λ = 254 nm, τ_{major} = 21.0 min, τ_{minor} = 59.3 min).

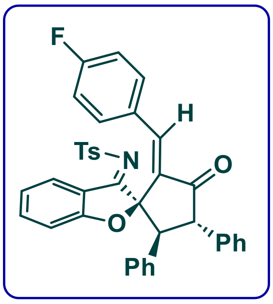

***N*-((2*R*,4'*S*,5'*S*,*E*)-2'-((*E*)-4-fluorobenzylidene)-3'-oxo-4',5'-diphenyl-3*H*-spiro [benzofuran-2,1'-cyclopentan]-3-ylidene)-4-methylbenzenesulfonamide(4x):** Pale yellow solid, 56.3 mg, 92% yield, 89% *ee*, the measured *dr* is 5:1 from ^{1}H NMR: 4.64-4.60 (*minor*), 4.53-4.50(*major*), E/Z = >20:1; R_f = 0.4 (petroleum ether/ethyl acetate = 90:10)

^{1}**H NMR (400 MHz,** Chloroform-d) δ 8.26 (s, 1H), 7.95 (s, 1H), 7.59 (d, *J* = 7.9 Hz, 2H), 7.49 – 7.43 (m, 1H), 7.31 – 7.26 (m, 3H), 7.26 – 7.23 (m, 2H), 7.20-7.17 (m, 4H), 7.06 – 6.98 (m, 3H), 6.97 – 6.89 (m, 3H), 6.84 (d, *J* = 8.4 Hz, 1H), 6.74 (t, *J* = 8.7 Hz, 2H), 4.52 (d, *J* = 14.0 Hz, 1H), 3.98 (s, 1H), 2.46 (s, 3H). ^{13}**C NMR** (101 MHz, Chloroform-d) (*major*) δ 201.13, 179.54, 167.96, 143.72, 142.28, 139.16, 138.51, 135.76, 134.62, 132.31, 131.94, 131.86, 131.61, 129.98, 129.95, 129.56, 129.24, 129.06, 128.90, 128.85, 128.41, 128.23, 128.15, 127.66, 127.21, 127.09, 122.45, 116.03, 115.82, 112.69, 94.09, 61.62, 56.69, 21.82.

HRMS (ESI^{+}) (*m/z*): $[M+K]^{+}$ calcd for $C_{38}H_{28}FNO_4S$: 618.1710, found: 618.1715;

HPLC: The enantiomeric excess was determined using CHIRALPAK IA column (*n*-Hexane/ ^{i}PrOH=90:30, flow rate=1.0 mL/min, λ = 254 nm, τ_{minor} = 22.7 min, τ_{major} = 56.9 min).

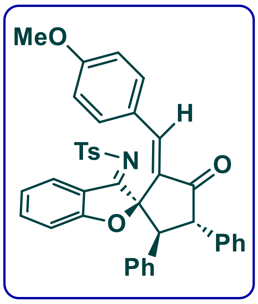

***N*-((2*R*,4'*S*,5'*S*,*E*)-2'-((*E*)-4-methoxybenzylidene)-3'-oxo-4',5'-diphenyl-3*H*-spiro [benzofuran-2,1'-cyclopentan]-3-ylidene)-4-methylbenzenesulfonamide(4y):** Pale yellow solid, 58.1 mg, 93% yield, 86% *ee*, the measured *dr* is 8:1 from ^{1}H NMR: 4.62-4.60(*minor*), 4.52-4.49(*major*), E/Z = >20:1; R_f = 0.4 (petroleum ether/ethyl acetate = 90:10)

^{1}H NMR (500 MHz, Chloroform-d) δ 8.31 (s, 1H), 7.94 (d, *J* = 2.6 Hz, 1H), 7.50 (d, *J* = 8.0 Hz, 2H), 7.44 (t, *J* = 7.8 Hz, 1H), 7.24 (dd, *J* = 7.7, 4.9 Hz, 4H), 7.21 – 7.15 (m, 5H), 7.03-7.00 (m, 3H), 6.97-6.92 (m, 4H), 6.83 (d, *J* = 8.5 Hz, 1H), 6.60 (d, *J* = 8.3 Hz, 1H), 4.50 (d, *J* = 14.0 Hz, 1H), 3.91 (d, *J* = 36.8 Hz, 1H), 3.72 (d, *J* = 15.7 Hz, 3H), 2.43 (s, 3H).**^{13}C NMR** (126 MHz, Chloroform-d) (*major*+*minor*) δ 201.34, 200.73, 200.08, 181.71, 180.02, 171.13, 168.07, 162.16, 161.65, 143.98, 143.89, 143.49, 140.86, 138.98, 138.73, 138.57, 136.09, 135.22, 132.55, 132.38, 131.46, 130.99, 129.93, 129.88, 129.83, 129.81, 129.61, 129.43, 129.29, 129.12, 129.08, 128.85, 128.83, 128.76, 128.32, 128.26, 128.13, 128.02, 127.52, 127.26, 127.12, 127.03, 126.77, 126.16, 125.51, 122.65, 122.21, 114.34, 113.88, 113.81, 112.79, 112.11, 95.75, 94.71, 62.13, 58.41, 56.59, 55.66, 55.51, 54.35, 21.82, 21.78.

HRMS (ESI^{+}) *m/z*: $[M+H]^{+}$ calcd for $C_{39}H_{31}NO_5S$: 626.1996, found: 626.1990;

HPLC: The enantiomeric excess was determined using CHIRALPAK IA column (*n*-Hexane/ ^{i}PrOH=90:30, flow rate=1.0 mL/min, λ_{max}= 254 nm, τ_{minor} = 22.7 min, τ_{major} = 56.9 min).

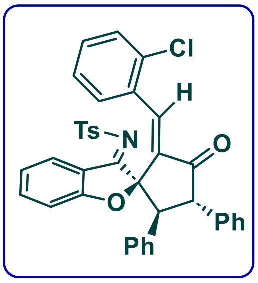

***N*-((2*R*,4'*S*,5'*S*,*E*)-2'-((*E*)-2-chlorobenzylidene)-3'-oxo-4',5'-diphenyl-3H-spiro[benzofuran-2,1'-cyclopentan]-3-ylidene)-4-methylbenzene sulfonamide(4z):** Pale yellow solid, 52.2 mg, 83% yield, 83% *ee*, the measured *dr* is 9:1 from ^{1}H NMR: 4.65-4.63(*minor*), 4.56-4.54(*major*), E/Z = >20:1; R_f = 0.4 (petroleum ether/ethyl acetate = 90:10)

^{1}H NMR (600 MHz, Chloroform-d) δ 8.14 (d, *J* = 8.3 Hz, 1H), 8.12 (s, 1H), 7.78 – 7.73 (m, 2H), 7.47 - 7.45 (m, 1H), 7.36 (t, *J* = 7.7 Hz, 3H), 7.27-7.26 (m, 1H), 7.26 - 7.25 (m, 2H), 7.21 - 7.19 (m, 6H), 7.04 (t, *J* = 7.7 Hz, 2H), 7.00 – 6.97 (m, 1H), 6.91 – 6.86 (m, 2H), 6.61 – 6.55 (m, 2H), 4.55 (d, *J* = 14.0 Hz, 1H), 3.99 (d, *J* = 13.7 Hz, 1H), 2.48 (s, 3H).**^{13}C NMR** (151 MHz, Chloroform-d) (*major*+*minor*) δ 200.67, 179.31, 167.73, 143.74, 140.28, 139.51, 139.07,

138.44, 137.60, 137.33, 135.42, 135.38, 134.78, 132.72, 132.17, 131.70, 130.31, 130.10, 129.96, 129.77, 129.64, 129.12, 129.03, 128.82, 128.26, 128.15, 127.67, 127.32, 127.10, 126.46, 125.23, 122.62, 122.28, 120.72, 114.28, 112.59, 93.73, 60.85, 56.55, 53.64, 22.91, 21.87.

HRMS (ESI$^+$) *m/z*: $[M+H]^+$ calcd for $C_{38}H_{29}ClNO_4S$: 630.1501, found: 630.1501;

HPLC: The enantiomeric excess was determined using CHIRALPAK IA column (*n*-Hexane/ ^{i}PrOH=90:30, flow rate=1.0 mL/min, λ_{max}= 254 nm, τ_{major} = 24.7 min, τ_{minor} = 52.1min).

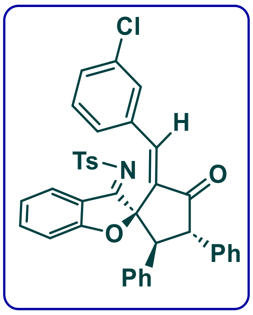

***N*-((2*R*,4'*S*,5'*S*,*E*)-2'-((*E*)-3-chlorobenzylidene)-3'-oxo-4',5'-diphenyl-3*H*-spiro[benzofuran-2,1'-cyclopentan]-3-ylidene)-4-methylbenzenesulfonamide(4z'):** Pale yellow solid, 54.1 mg, 86% yield, 83% *ee*, the measured *dr* is 6:1 from ^{1}H NMR: 4.65-4.63(*minor*), 4.56-4.54(*major*), E/Z = >20:1; R_f = 0.4 (petroleum ether/ethyl acetate = 90:10)

^{1}H NMR (500 MHz, CDCl3) δ 8.277 (s, 1H), 7.92 (s, 1H), 7.59 (d, J = 7.8 Hz, 2H), 7.45 (t, J = 7.8 Hz, 1H), 7.28 (d, J = 8.0 Hz, 2H), 7.24 (d, J = 6.8 Hz, 2H), 7.19 (d, J = 7.3 Hz, 4H), 7.04 - 7.00 (m, 4H), 6.85 - 6.82 (m, 3H), 6.83 (t, J = 6.9 Hz, 3H), 4.51 (d, J = 13.9 Hz, 1H), 3.98 (s, 1H), 2.44 (s, 3H). **^{13}C NMR** (126 MHz, CDCl3) (*major*+*minor*) δ 200.86, 200.26, 181.22, 179.16, 170.88, 167.78, 143.95, 143.55, 141.70, 139.02, 138.98, 138.93, 138.48, 138.21, 136.73, 135.91, 135.72, 135.53, 134.99, 133.45, 133.29, 132.14, 132.05, 131.94, 130.79, 130.74, 130.43, 130.16, 129.74, 129.68, 129.36, 129.04, 128.86, 128.71, 128.65, 128.57, 128.44, 128.23, 128.19, 128.04, 127.97, 127.48, 127.03, 126.89, 122.64, 122.30, 120.59, 117.39, 114.08, 112.45, 111.71, 94.88, 93.77, 61.35, 57.52, 56.50, 54.39, 21.65, 21.62.

HRMS (ESI$^+$) *m/z*: $[M+H]^+$ calcd for $C_{38}H_{29}ClNO_4S$: 630.1501, found: 630.1498;

HPLC: The enantiomeric excess was determined using CHIRALPAK IA column (*n*-Hexane/ ^{i}PrOH=90:30, flow rate=1.0 mL/min, λ_{max}= 254 nm, τ_{minor} = 17.9 min, τ_{major} = 20.9 min)

8. Synthetic transformation of 3a:

Reduction of 3a:

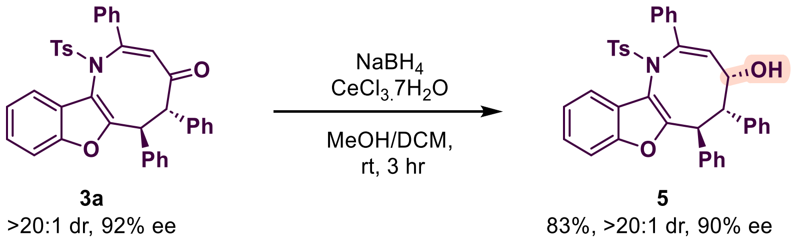

In an oven *dr*ied 10 mL round-bottom flask, **3a** (59.5 mg, 0.1 mmol, 1.0 equiv) was taken along with 1.5 mL of MeOH and 1.5 mL of DCM. The resulting solution was cooled to 0 °C using an ice-bath, followed by portion-wise addition of $CeCl_3.7H_2O$ (73.9mg, 0.3 mmol, 3 equiv) and $NaBH_4$ (18.9 mg, 0.5 mmol, 5 equiv) separately. The resulting mixture was stirred at 0 °C. After complete consumption of **3a**, H_2O (5 mL) was added dropwise. The resulting mixture was diluted with 5 mL of DCM. The organic layer was separated, and the aqueous layer was extracted with DCM (3 × 5 mL). Combined organic layer was washed with brine (3 × 5 mL), dried over anhydrous Na_2SO_4 and concentrated under reduced pressure. The residue was purified by flash column chromatography (petroleum ether : ethyl acetate = 95:5) to give product **5** as white solid (49.5 mg, 83% yield, >20:1 *dr*, 90% *ee*). **^{1}H NMR** (500 MHz, Chloroform-d) δ 7.50 (d, J = 7.7 Hz, 2H), 7.41 – 7.20 (m, 17H), 7.15 (t, J = 7.3 Hz, 2H), 7.05 (d, J = 8.0 Hz, 2H), 5.30 (s, 1H), 5.14 (s, 1H), 4.60 (s, 1H), 4.30 (s, 1H), 2.38 (s, 3H). **^{13}C NMR** (126 MHz, Chloroform-d) δ 153.52, 144.09, 138.73, 137.42, 129.44, 129.28, 129.11, 128.80, 128.60, 128.53, 128.45, 128.18, 127.29, 127.06, 126.71, 124.75, 123.43, 119.72, 112.22, 71.63, 46.39, 31.79, 21.77. **HRMS (ESI$^+$) (*m/z*):** $[M+H]^+$ calcd for $C_{38}H_{31}NO_4S$: 598.2047, found: 598.2053; **HPLC:** HPLC: The enantiomeric excess was determined using CHIRALPAK ID column (*n*-Hexane/ *i*-PrOH=90:10, flow rate=1.0 mL/min, λ_{max}= 254 nm, τ_{major} = 13.4 min, τ_{minor} = 20.9 min).

Chlorination of 3a:

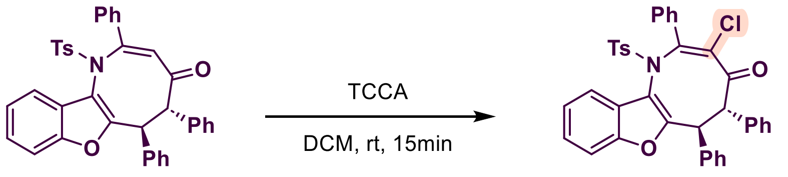

3a
>20:1 dr, 92% ee

6
99%, >20:1 dr, 92% ee

In an oven dried 5ml vial, **3a** (59.5 mg, 0.1 mmol) was taken in 0.5 ml DCM. In that resulting solution, trichloroisocyanuric acid (TCCA) (23.2 mg, 0.1 mmol) was added and stirred at room temperature until **3a** fully consumed. At the end of the reaction, the solvent was removed in vacuo. The residue was purified by flash column chromatography (petroleum ether : ethyl acetate = 95:5) to give product **6** as white solid (57.96 mg, 92% yield, >20:1 *dr*, 92% *ee*). **^{1}H NMR** (500 MHz, Chloroform-d) δ 7.60 (d, J = 7.6 Hz, 2H), 7.57 (d, J = 7.5 Hz, 4H), 7.49 (t, J = 7.6 Hz, 1H), 7.41 (t, J = 9.1 Hz, 3H), 7.28 (d, J = 6.9 Hz, 1H), 7.24 (d, J = 8.4 Hz, 2H), 7.18 (d, J = 7.4 Hz, 3H), 7.11 (t, J = 7.8 Hz, 2H), 6.94 (q, J = 8.1 Hz, 4H), 6.79 (d, J = 7.8 Hz, 1H), 6.25 (d, J = 13.1 Hz, 1H), 5.00 (d, J = 13.1 Hz, 1H), 2.39 (s, 3H). **^{13}C NMR** (126 MHz, Chloroform-d) δ 197.27, 158.89, 152.76, 150.93, 144.79, 136.83, 136.69, 135.84, 134.77, 131.43 – 131.04 (m), 130.67, 130.19, 129.29, 128.90, 128.61, 128.52, 128.37, 127.88, 127.52, 125.62, 125.53, 123.30, 120.80, 119.37, 112.36, 54.65, 49.79, 21.80. **HRMS (ESI$^+$) (*m/z*):**

[M+Na]$^+$ calcd for $C_{38}H_{28}ClNO_4S$: 652.1320, found: 652.1320; **HPLC:** HPLC: The enantiomeric excess was determined using CHIRALPAK ID column (*n*-Hexane/ *i*-PrOH=90:10, flow rate=1.0 mL/min, λ_{max}= 254 nm, τ_{major} = 13.4 min, τ_{minor} = 20.9 min).

Allylation of 3a:

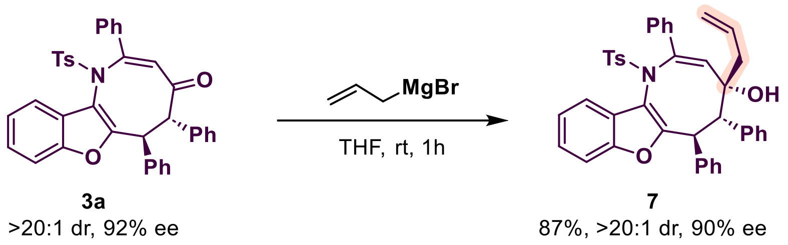

In an oven dried 10 mL round-bottom flask, **3a** (59.5 mg, 0.1 mmol, 1.0 equiv) was taken in 1.5 mL dry THF under argon atmosphere. The resulting solution was cooled to 0 °C using ice-bath, followed by dropwise addition of allyl magnesium bromide (1 M in Et_2O; 0.60 mL, 0.60 mmol, 4.0 equiv) over 5 minutes. The resulting mixture was allowed to attain ambient temperature. After complete consumption of **3a**, sat. aqueous NH_4Cl (3 mL) was added. The resulting mixture was dilute with EtOAc (5 mL). The organic layer was separated, and the aqueous layer was extracted with EtOAc (3 × 5 mL). Combined organic layer was washed with brine (2 × 5 mL), dried over anhydrous Na_2SO_4 and concentrated under reduced pressure. Purification by flash column chromatography (petroleum ether : ethyl acetate = 95:5) afforded **7** as a white solid (52.87mg, 87% yield, >20:1 *dr*, 90% *ee*). **^{1}H NMR** (400 MHz, Chloroform-d) δ 7.46 (t, J = 6.9 Hz, 3H), 7.34 (t, J = 7.4 Hz, 3H), 7.29 – 7.25 (m, 3H), 7.22 – 7.20 (m, 3H), 7.15 - 7.08 (m, 4H), 7.01 – 6.96 (m, 5H), 6.93 – 6.90 (m, 1H), 6.60 (d, J = 7.9 Hz, 1H), 6.02 – 5.92 (m, 1H), 5.78 (s, 1H), 5.20 (d, J = 10.0 Hz, 1H), 5.09 (s, 2H), 5.02 (d, J = 17.0 Hz, 1H), 2.54 - 2.49 (m, 1H), 2.43 (s, 3H), 2.13 - 2.08 (m, 1H). **^{13}C NMR** (126 MHz, Chloroform-d) δ 158.84, 153.30, 144.29, 141.83, 140.63, 140.38, 139.67, 137.82, 133.53, 133.19, 130.37, 129.64, 129.49, 129.17, 128.81, 128.73, 128.33, 127.81, 127.68, 127.66, 126.34, 126.13, 126.04, 124.33, 122.75, 120.70, 120.06, 119.63, 111.91, 50.41, 49.87, 47.67, 21.82. **HRMS (ESI$^+$) (*m/z*):** [M+K]$^+$ calcd for $C_{41}H_{35}NO_4S$: 676.1919, found: 676.1922; **HPLC:** The enantiomeric excess was determined using CHIRALPAK ID column (*n*-Hexane/ ^{i}PrOH=90:10, flow rate=1.0 mL/min, λ_{max}= 254 nm, τ_{minor} = 23.2 min, τ_{major} = 25.9 min).

Tosyl deprotection of 3a:

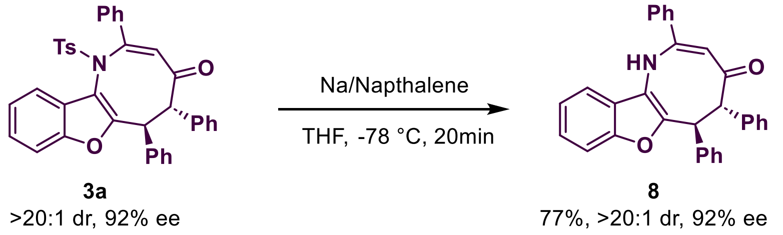

To a solution of Napthalene(76.8mg, 0.6mmol, 6eqiv.) in 2ml THF was added sodium(13.6mg, 06mmol, 6equiv.) at room temperature under argon atmosphere. The colourless solution was stirred until becomes dark-green solution. After that, this solution was added over a solution of **3a** (59.5mg, 0.1mmol, 1equiv.) in 1ml THF at -78 °C for 10 min. The reaction mixture was stirred at the same temperature for 20 min. After, consumption of **3a**, sat. aq. NH_4Cl (3ml) was added. The mixture was diluted with EtOAc (5ml). The organic layer was separated, and the aqueous layer was extracted with EtOAc (3 × 5 mL). Combined organic layer was washed with brine (2 × 5 mL), dried over anhydrous Na_2SO_4 and concentrated under reduced pressure. Purification by flash column chromatography (petroleum ether : ethyl acetate = 90:15) afforded **8** as a white solid (33.9mg, 77% yield, >20:1 dr, 92% ee). **^{1}H NMR** (600 MHz, DMSO-d_6) δ 9.23 (s, 1H), 8.01 – 7.98 (m, 2H), 7.93 (d, J = 7.5 Hz, 1H), 7.67 – 7.61 (m, 3H), 7.47 (d, J = 8.0 Hz, 1H), 7.44 – 7.41 (m, 2H), 7.40 - 7.38 (m, 1H), 7.36 - 7.33 (m, 1H), 7.30 – 7.27 (m, 2H), 7.18 (t, J = 7.6 Hz, 2H), 7.15 – 7.09 (m, 3H), 7.06 – 7.02 (m, 1H), 5.22 (s, 1H), 5.19 (d, J = 12.9 Hz, 1H), 4.77 (d, J = 12.9 Hz, 1H). **^{13}C NMR** (151 MHz, DMSO-d_6) δ 198.21, 158.31, 153.62, 150.98, 140.34, 137.76, 136.77, 131.29, 129.55, 129.08, 128.92, 128.28, 128.09, 127.61, 126.87, 126.64, 126.22, 125.37, 123.11, 121.83, 119.49, 111.18, 103.82, 55.68, 44.99. **HRMS (ESI$^+$) (*m/z*):** $[M+H]^+$ calcd for $C_{31}H_{23}NO_2$: 442.1802, found: 442.1802; **HPLC:** The enantiomeric excess was determined using CHIRALPAK ID column (*n*-Hexane/ ^{i}PrOH=90:30, flow rate=1.0 mL/min, λ_{max}= 254 nm, τ_{major} = 8.9 min, τ_{minor} = 19.0 min).[Note: this product **8** is unstable in silica gel)

9. Single crystal X-ray diffraction analysis:

Single crystal X-ray diffraction analysis of 3f: The compound **3f** was dissolved in minimum amount of hot *n*-hexane/ethyl acetate (3:1) and kept the solution at room temperature for 4 days to give block like crystal. The crystallographic refinement parameters are given below:

**CCDC**	2235079		
**Bond precision:**	C-C = 0.0068 Å		**Wavelength** = 0.71073
**Cell:**	**a** = 8.8558(7) Å	**b** = 17.2619(11) Å	**c** = 10.4540(8) Å
	**α** = 90°	**β** = 91.877°	**γ** = 90°
**Temperature:**	296 K		

**Volume (Å^3)**	1597.2(2)
**Space group**	*P*21
**Crystal system**	*Monoclinic*
**Moiety formula**	$C_{38}H_{28}BrNO_4S$
**Formula Weight**	674.58
**Density (g cm^{-3})**	1.403
**Z**	2

**Absorption coefficient, μ (mm^{-1})**	1.394
**F000**	692.0
**Index ranges**	$-10 \leq h \leq 10$,$-20 \leq k$ 20,$-12 \leq l \leq 12$
**Independent reflections**	5612
**T_{min},T_{max}**	0.647,0.696
**$T_{min'}$**	0.634
**Data completeness**	1.93/1.00
**θ range for data collection**	1.949 to 24.996°
**Final R indexes [I ≥ 2σ (I)]**	R_1 = 0.0321, ωR_2 = 0.0658
**Final R indexes [all data]**	R_1 = 0.0444, ωR_2 = 0.0710
**Goodness-of-fit on F^2**	1.041
**Data/restraints/parameters**	5612/1/407
**Flack parameter**	0.023

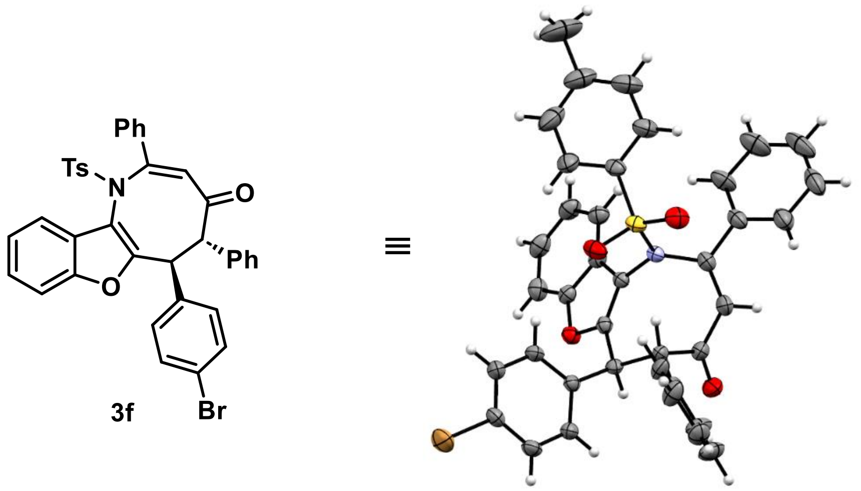

ORTEP of the X-ray structure of **3f** (ellipsoids with 30% probability)

Single crystal X-ray diffraction analysis of 4k: The compound **4k** was dissolved in minimum amount of hexane/chloroform (3:1) and kept the solution at room temperature for 7 days to give block like crystal. The crystallographic refinement parameters are given below:

**CCDC**	2235267		
**Bond precision:**	C-C = 0.0093 Å		**Wavelength** = 0.71073
**Cell:**	**a** = 10.022(4) Å	**b** = 11.611(5) Å	**c** = 30.123(13) Å
	**α** = 90°	**β** = 90°	**γ** = 90°
**Temperature:**	296 K		

**Volume (Å^3)**	3505(3)
**Space group**	*P*212121
**Crystal system**	*Orthorhombic*
**Moiety formula**	$C_{36}H_{27}NO_4S_2$
**Formula Weight**	721.07
**Density (g cm^{-3})**	1.366
**Z**	4
**Absorption coefficient, μ (mm^{-1})**	0.421
**F000**	1488.0
**Index ranges**	-11 ≤ h ≤ 11 ,-13 ≤ k 13,-35 ≤ l ≤ 35
**Independent reflections**	6128
**T_{min},T_{max}**	0.922, 0.959
**$T_{min'}$**	0.912
**Data completeness**	1.76/0.99
**θ range for data collection**	1.35 to 25.00°
**Final *R* indexes [I ≥ 2σ (I)]**	R_1 = 0.0770, ωR_2 = 0.2148
**Final *R* indexes [all data]**	R_1 = 0.0935, ωR_2 = 0.2402
**Goodness-of-fit on F^2**	1.008
**Data/restraints/parameters**	6128/0/425
**Flack parameter**	0.05

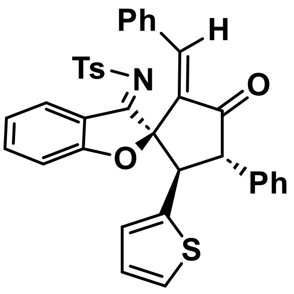

4k

≡

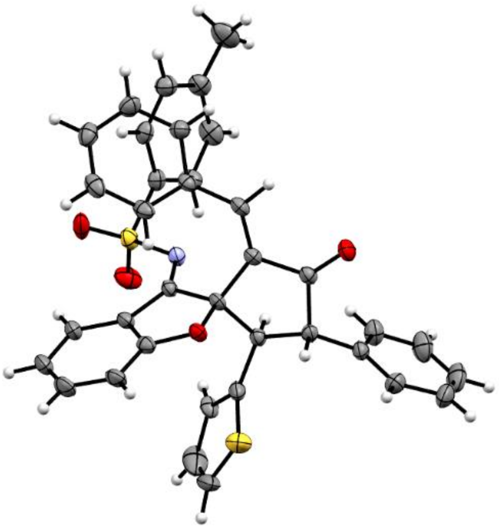

ORTEP of the X-ray structure of **4k** (ellipsoids with 30% probability)

10. Mechanistic Study:

Procedure for synthesis of Intermediate A:

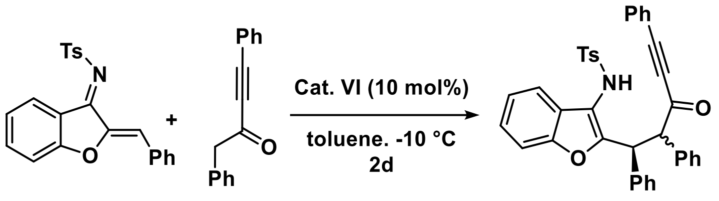

To a stirred solution of 1-azadienes **1a** (37.5 mg, 0.1 mmol) and freshly prepared ynones **2a** (22mg, 0.11 mmol) in toluene solvent (1 mL) at -10 °C, were added 10 mol% catalyst **VI**. The reaction was allowed to run in the same temperature for 2 days. After full consumption of starting materials, solvents were evaporated and the reaction mixture was purified by flash column chromatography (petroleum ether : ethyl acetate = 95:5 to 90:15) to afford intermediate **A** as a white solid (58.9 mg, 0.099 mmol, 99% yield, 92%ee(*major isomer*), 88%ee(*minor isomer*)). the measured *dr* is 7:1 from ^{1}H NMR: 4.62-4.60(*minor*), 4.52-4.49(*major*). **^{1}H NMR** (500 MHz, Chloroform-d) δ 7.69 (d, J = 8.0 Hz, 1H), 7.52 (t, J = 8.7 Hz, 4H), 7.48 – 7.45 (m, 1H), 7.41 (q, J = 9.5, 8.2 Hz, 5H), 7.34 (q, J = 8.3 Hz, 4H), 7.30 – 7.25 (m, 4H), 7.24 – 7.15 (m, 7H), 7.12 (d, J = 7.8 Hz, 3H), 7.10 - 7.07 (m, 2H), 7.02 - 6.99 (dd, J = 5.1, 1.9 Hz, 2H), 6.94 (t, J = 7.5 Hz, 1H), 6.83 – 6.80 (m, 2H), 6.70 (s, 1H), 5.53 (d, J = 4.5 Hz, 1H), 5.15 (d, J = 11.5 Hz, 1H), 4.87 (d, J = 11.5 Hz, 1H), 4.78 (d, J = 11.6 Hz, 1H), 4.53 (d, J = 11.7 Hz, 1H), 2.39 (s, 2H), 2.36 (s, 3H). **^{13}C NMR** (126 MHz, Chloroform-d) δ 185.96, 184.97, 154.37, 153.62, 153.32, 153.27, 144.10, 143.86, 138.19, 137.05, 137.03, 136.74, 135.56, 134.61, 133.40, 133.34, 131.15, 131.13, 130.00, 129.91, 129.43, 129.18, 129.15, 129.05, 129.00, 128.94, 128.83, 128.76, 128.69, 128.41, 128.17, 128.07, 127.74, 127.72, 127.54, 127.05, 126.40, 125.58, 124.62, 124.44, 123.42, 123.06, 120.53, 119.96, 119.77, 119.12, 113.99, 113.76, 111.45, 111.37, 93.94, 93.51, 88.01, 87.90, 64.49, 64.46, 45.01, 44.15, 21.79, 21.75. **HRMS (ESI+) (*m/z*)**: [M+H]+ calcd for $C_{38}H_{29}NO_4S$: 596.1891, found: 596.1891; **HPLC**: The enantiomeric excess was determined using CHIRALPAK ID column (n-Hexane/ i-PrOH = 90:10, flow rate = 1.0 mL/min, λ =254 nm, τ_{major}(*major isomer*) = 15.7 min, τ_{minor}(*major isomer*) = 17.7 min, τ_{major}(*major isomer*) = 23.5 min, τ_{minor}(*major isomer*) = 25.2 min).

H/D exchange experiment of intermediate A with DBU:

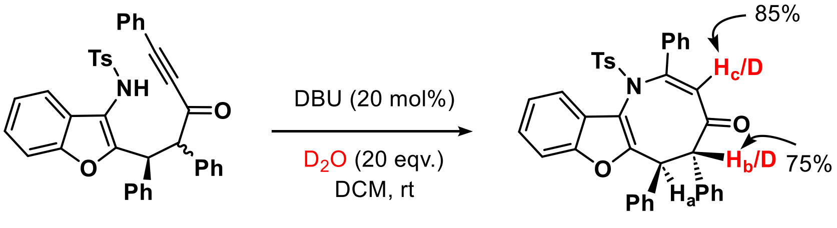

A, d.r.= 1.7:1 **D$_2$-3a**, d.r. > 20:1

The isolated intermediate **A** (59.5mg, 0.1mmol) was taken in an oven dried vial and DCM were added. After that, DBU (20 mol%) and D_2O (20 equiv.) were added sequentially. The reaction mixture was stirred for 3 hr at room temperature. Then, the solvent was evaporated and residue was purified by flash chromatography (petroleum ether : ethyl acetate = 95:5) to give **D_2-3a**.

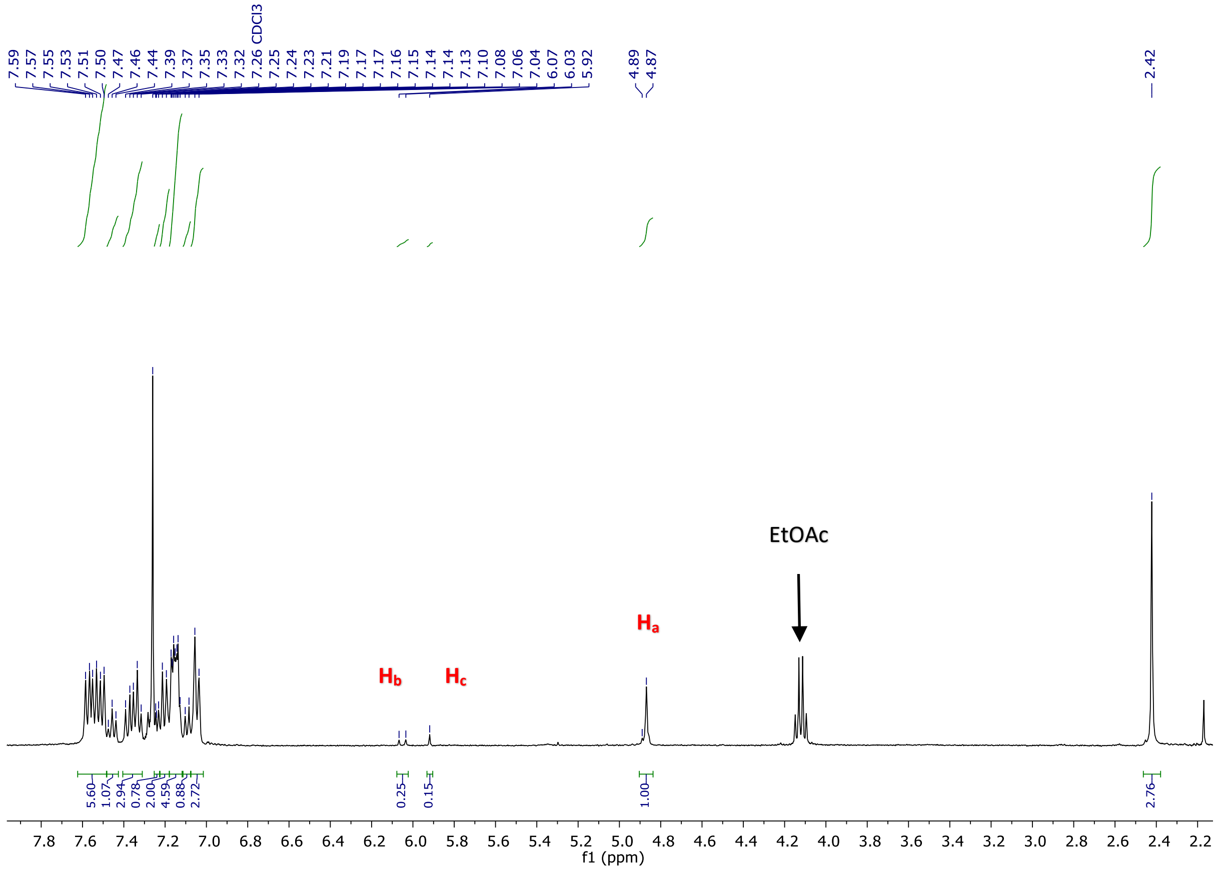

H/D exchange experiment of intermediate A with PPh_3:

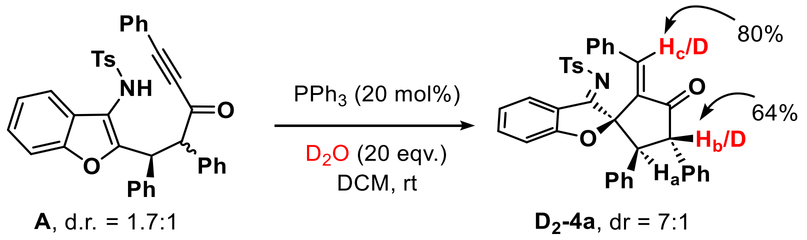

The isolated intermediate **A** (59.5 mg, 0.1 mmol) was taken in an oven dried vial and DCM were added. After that, PPh_3 (20 mol%) and D_2O (20 equiv.) were added. The reaction mixture was stirred for 12 hr at room temperature. Then, the solvent was evaporated and purified by flash chromatography (petroleum ether : ethyl acetate = 90:10) to afford **D_2-4a**.

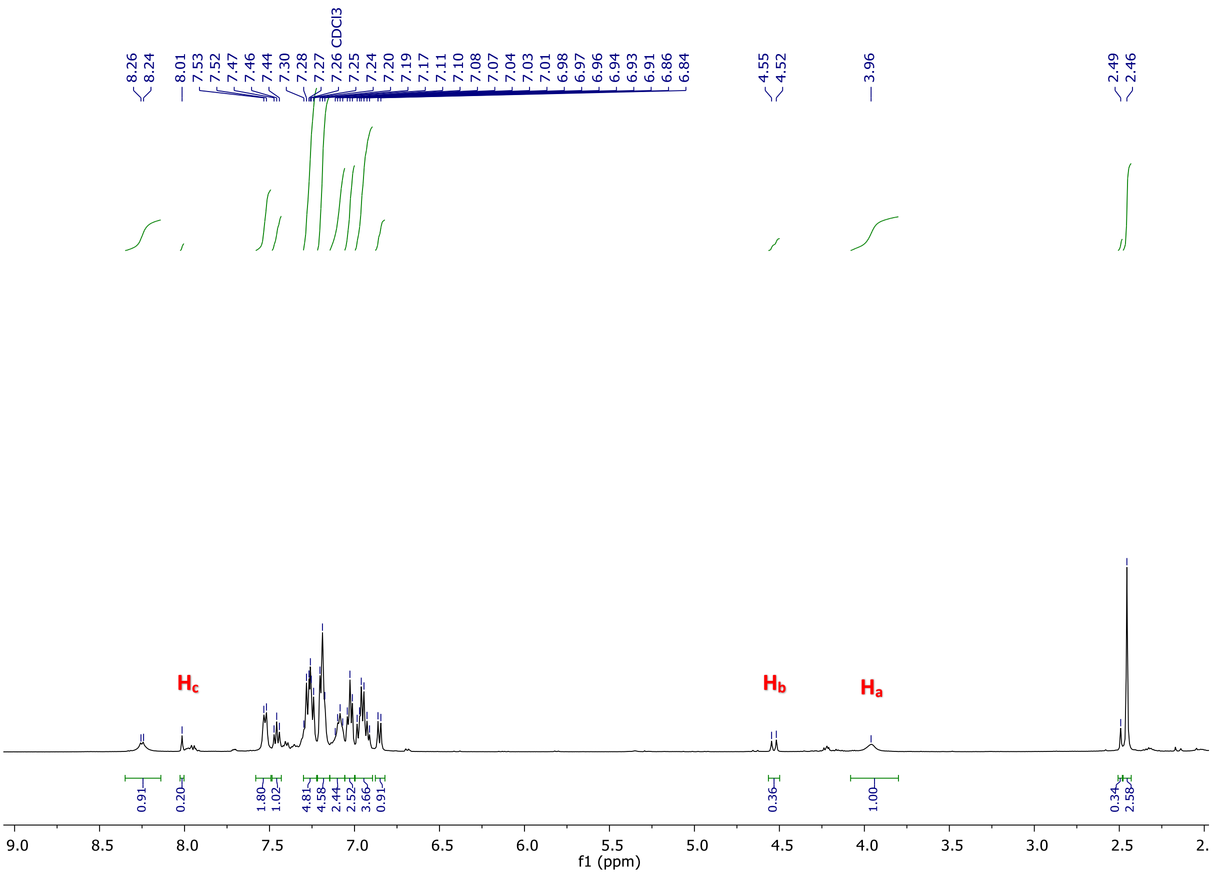
8.26
8.24
8.01
7.53
7.52
7.47
7.46
7.44
7.30
7.28
7.27
7.26 CDCl3
7.25
7.24
7.20
7.19
7.17
7.11
7.10
7.08
7.07
7.04
7.03
7.01
6.98
6.97
6.96
6.94
6.93
6.91
6.86
6.84
4.55
4.52
3.96
2.49
2.46
Hc
Hb
Ha
0.91
0.20
1.80
1.02
4.81
4.58
2.44
2.52
3.66
0.91
0.36
1.00
0.34
2.58
f1 (ppm)

ESI-MS mass spectroscopy:

Intermediate **A**(0.05mmol) was taken in an oven-dried mass vial and 0.5ml CH_3CN was added. Then, 20mol% DBU was added. After stirring 15min, mixture was diluted with CH_3CN and ESI-MS data was measured. A *m/z* of 748.3209, [M+H+] correspond to intermediate I_D, II_D, and III_D.

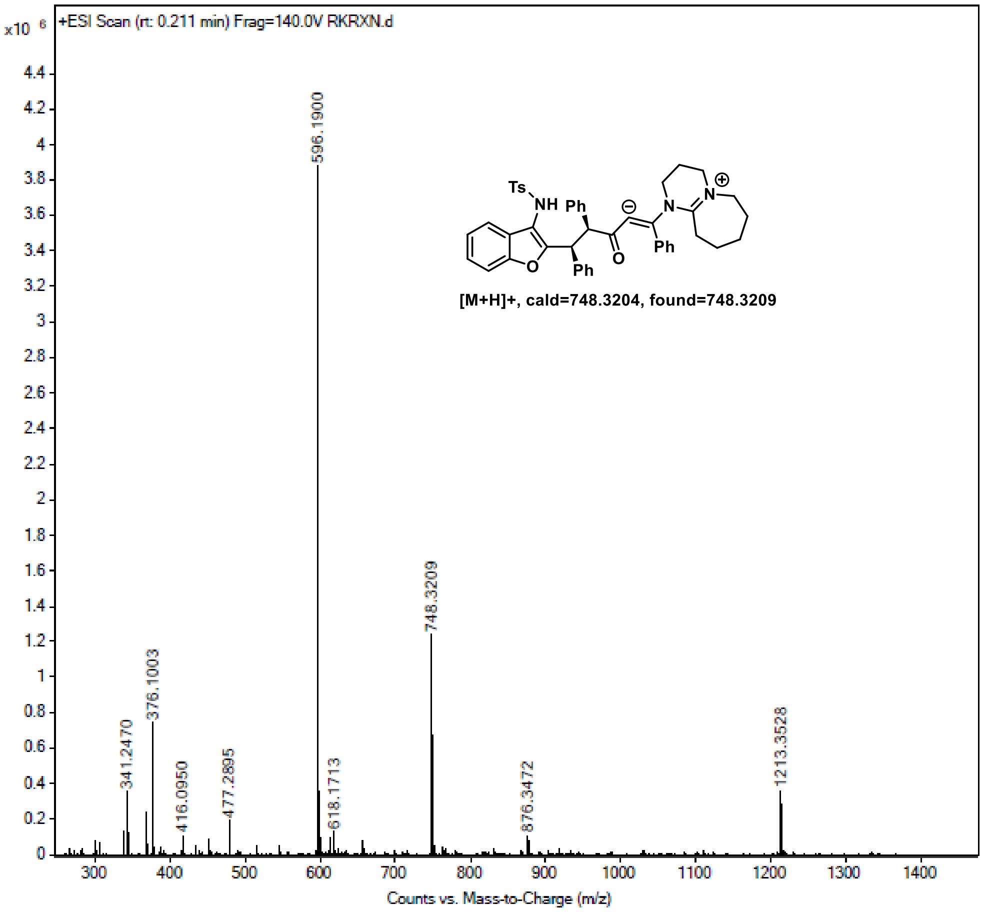

11. Computational Details:

All calculations were carried out with ORCA 5.0.3 suite of programs.[8] Geometries were optimized without any constraints in gas phase using the PBE functional,[9] in conjugation with the double ζ quality split-valence def2-SVP atom-centred basis set on all atoms. Grimme's empirical dispersion correction with Becke-Johnson damping function[10] has been utilized in order to correctly account for the various non-covalent interactions such as π-π, C-H---π and H-bond interactions. The density fitting resolution of identity (RI) approximation was used to accelerate the calculations in conjunction to the decontracted auxiliary Coulomb-fitting basis set def2/J.[11] The following thresholds were used for optimizations: energy change tolerance of 5×10^{-6} Hartree, maximum gradient of 3×10^{-4} Hartree, root mean square gradient of 1×10^{-4} Hartree/Bohr, maximum displacement of 4×10^{-3} Bohr and root mean square displacement of 2×10^{-3} Bohr. The DFT fine integration grid in built within ORCA 5.0.3 version has been used all through. Tight convergence criteria (energy tolerance = 1×10^{-8} Hartree) for self-consistent field (SCF) calculations were employed throughout all calculations. To verify the nature of all stationary points (minima and transition states) and to evaluate thermochemical corrections, analytic frequency calculations were performed at 298.18 K by using the rigid-rotor harmonic oscillator (RRHO) approximation. Further, the energies of optimized geometries were refined by single point calculations with the empirical dispersion corrected and triple ζ quality split-valence B3LYP-D3(BJ)/def2-TZVP in conductor like polarizable continuum model (C-PCM) [12] solvent model using dielectric parameters of toluene (ε = 2.374, refractive index = 1.497) or dichloromethane (ε = 8.9, refractive index = 1.42), as and when required. The electronic energies in the solvent phase were also refined using single-point calculations at DLPNO-CCSD(T)/def2-TZVP [13] level of theory to verify the reliability of the relative energies predicted with DFT. Zero-point energy, enthalpy and entropy corrections were always extracted from PBE/def2-SVP calculations. Unless and otherwise mentioned, the following protocol has been utilized to predict the relative Gibbs free energies in the main text and supporting information: B3LYP-D3(BJ)/CPCM(Solvent)/def2-TZVP//PBE-D3(BJ)/def2-SVP. This approach has been recently successful in predicting the structural ensembles in silylium imidodiphos-phorimidate (IDPi) Lewis acid catalyzed Diels Alder reaction of α,β-unsaturated methyl esters, aminomethylation via asymmetric counter-anion directed catalysis, asymmetric intramolecular hydroalkoxylation of terminal olefins catalyzed by bulky Brønsted acids etc.[14]

The SambVca 2.1 web tool has been utilized to plot the steric maps and check for the confinement effect, as shown in Figure 2 (main text) and Figure S5 (supporting information).[15] One of the N-H protons of catalyst **VI** which is almost centrally placed within the frameworks of **TS*re-SS*** and **TS*re-SR*** has been chosen as the centre of the sphere with a radius of 7 Å. In case of **TS*si-SS*** and **TS*si-SR***, the above N centre is the chosen origin. The noncovalent interaction (NCI) plots were calculated with the Multiwfn 3.8 program.[16] B3LYP-

D3(BJ)/CPCM(Solvent)/def2-TZVP electron densities were utilized to calculate the reduced density matrices.

Intramolecular cyclization to five-, six-, seven- or eight membered heterocyclic or carbocyclic rings for the Lewis base (DBU and PPh_3) assisted transformation of $\mathbf{A}_{\boldsymbol{re\text{-}SS}}$ to the desired products (**3a** or **4a**) involve significant geometrical rearrangements from the equilibrium geometry of the reactant and the Lewis base to confined and sterically encumbered scaffolds. This principle leads to the cyclization process deemed as the rate-determining step for both type of reactions. Following the activation strain model proposed by Bickelhaupt and others,[17] we hypothesize that geometrical distortion in the relevant transition state ($\mathbf{TS2}_{\boldsymbol{D\text{-}SS}}$, $\mathbf{TS3}_{\boldsymbol{D\text{-}SS}}$, $\mathbf{TS2}_{\boldsymbol{P\text{-}SS}}$ and $\mathbf{TS3}_{\boldsymbol{P\text{-}SS}}$) is associated with the destabilizing strain energy coupled to a stabilizing effect of the interaction energy ensuing due to a favourable overlap of the molecular orbitals of the approaching entities. Thus, the zero-point uncorrected activation energy of a transition state ($\Delta E^{\ddagger}$) is a combination of the distortion energy (ΔE_{dist}) and the interaction energy (ΔE_{int}) according to the following relation: $\Delta E^{\ddagger} = \Delta E_{\mathrm{dist}} + \Delta E_{\mathrm{int}}$ (1). Hence, we take the separated reactants as reference points (unbound $\mathbf{A}_{\boldsymbol{re\text{-}SS}}$ and DBU/PPh_3) for each of the C-N or C-C bond forming transition states. The decomposition of the activation energy into the corresponding distortion energy and interaction energy is shown in Figure 5 (main text) which holds meaningful explanation on the observed selectivity. The eight-membered moiety is preferred over the six-membered moiety in DBU assisted pathway due to lower interaction energy for the former ($\Delta\Delta E_{\mathrm{int}}$ = -16.0 kcal/mol). However, the five-membered moiety predominates over the seven-membered ring in the PPh_3 assisted pathway due to significantly lowered geometrical distortion or rearrangement ($\Delta\Delta E_{\mathrm{dist}}$ = -33.6 kcal/mol).

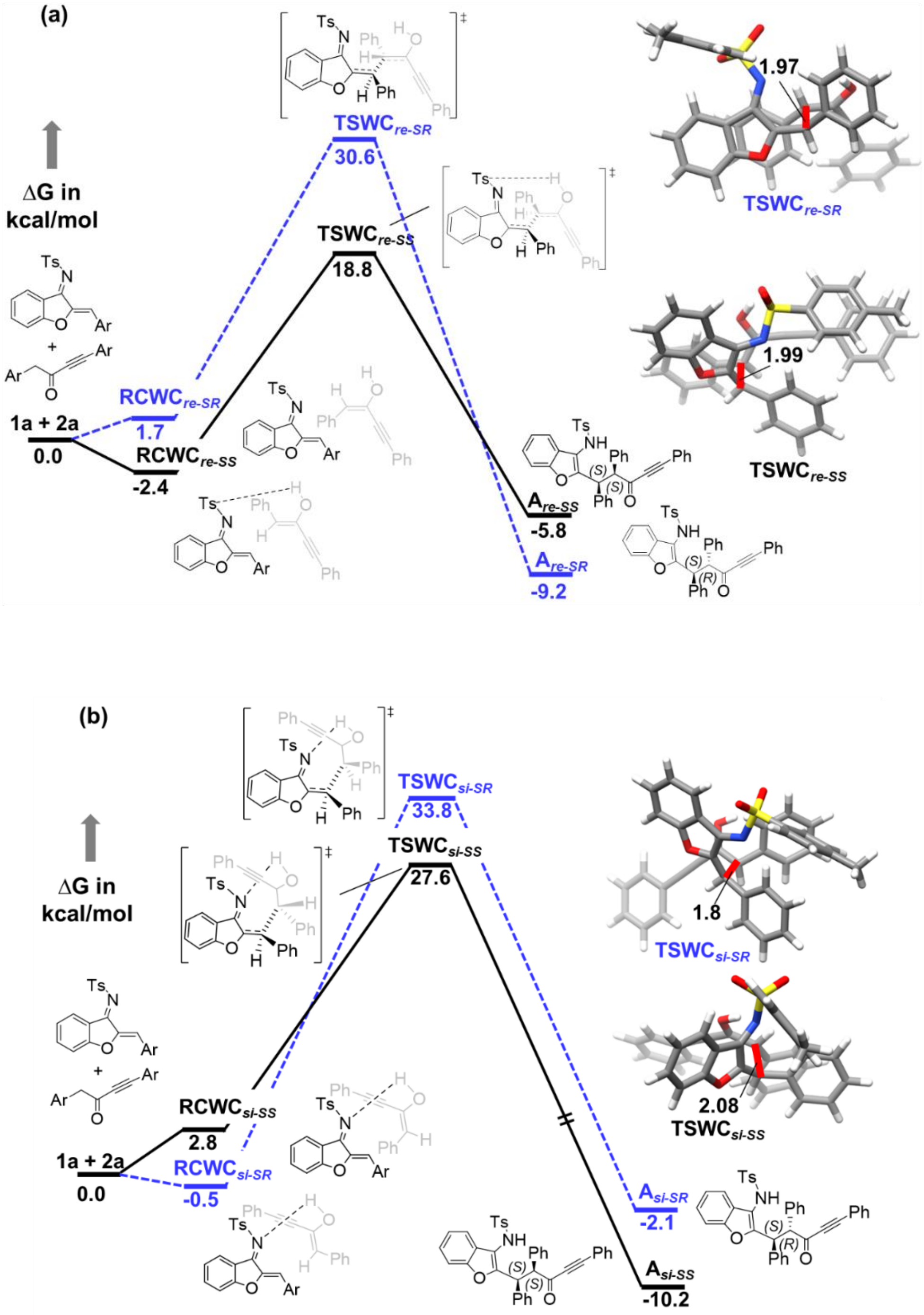

Figure S1. Gibbs free energy (kcal/mol) profile at B3LYP-D3(BJ)/CPCM(Toluene)/def2-TZVP for the uncatalyzed coupling between **1a** and **2a** in **(a)** *re*-face and **(b)** *si*-face. Distances shown are in units of Å. Color Code: C(grey), H(white), N(blue), O(red), S(yellow). WC = without catalyst.

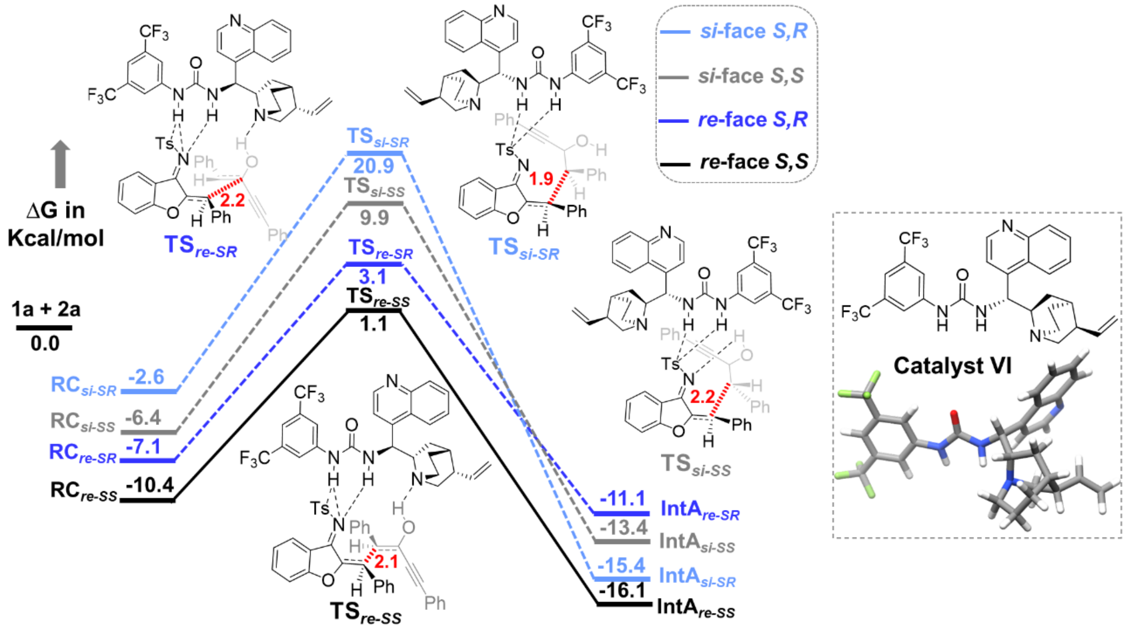

Figure S2. Gibbs free energy (kcal/mol) profile at B3LYP-D3(BJ)/CPCM(Toluene)/def2-TZVP for the for the bifunctional urea catalyst **VI** mediated **1a** and **2a** coupling in both *re*- and *si*-faces. Distances shown are in units of Å. Color Code: C(grey), H(white), N(blue), O(red), S(yellow). WC = without catalyst.

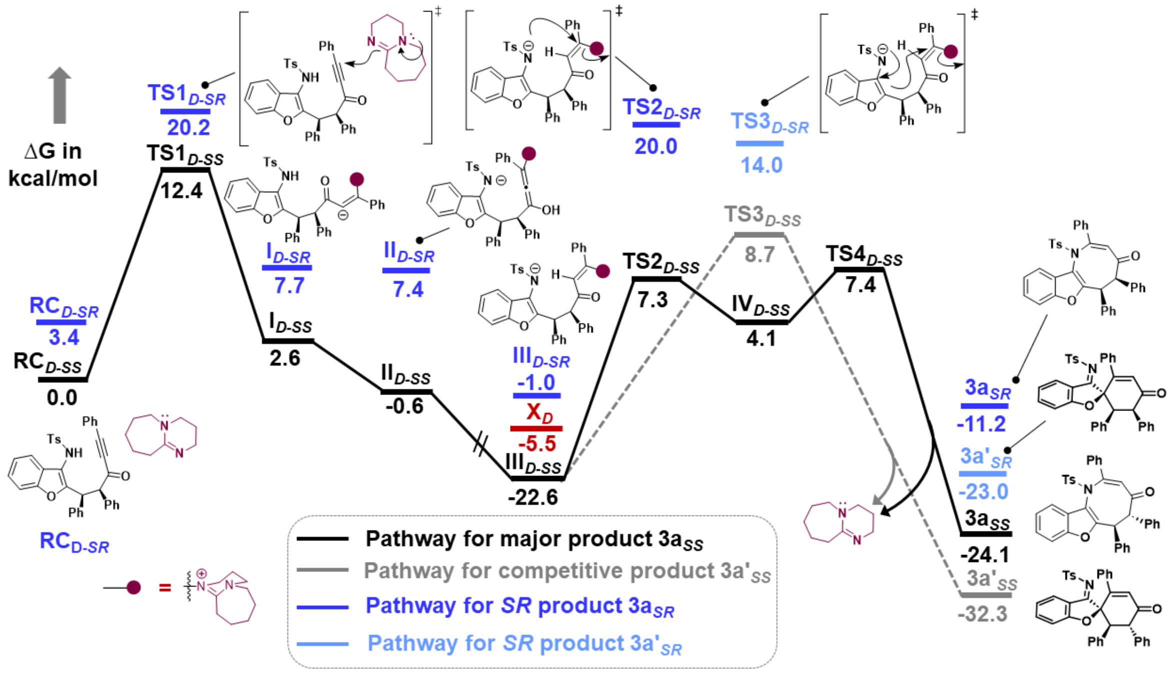

Figure S3. Gibbs free energy (kcal/mol) profile at B3LYP-D3(BJ)/CPCM(CH_2Cl_2)/def2-TZVP for the DBU assisted intramolecular cyclization of intermediates $\mathbf{A}_{re\text{-}SS}$ and $\mathbf{A}_{re\text{-}SR}$. D subscript denotes DBU assisted.

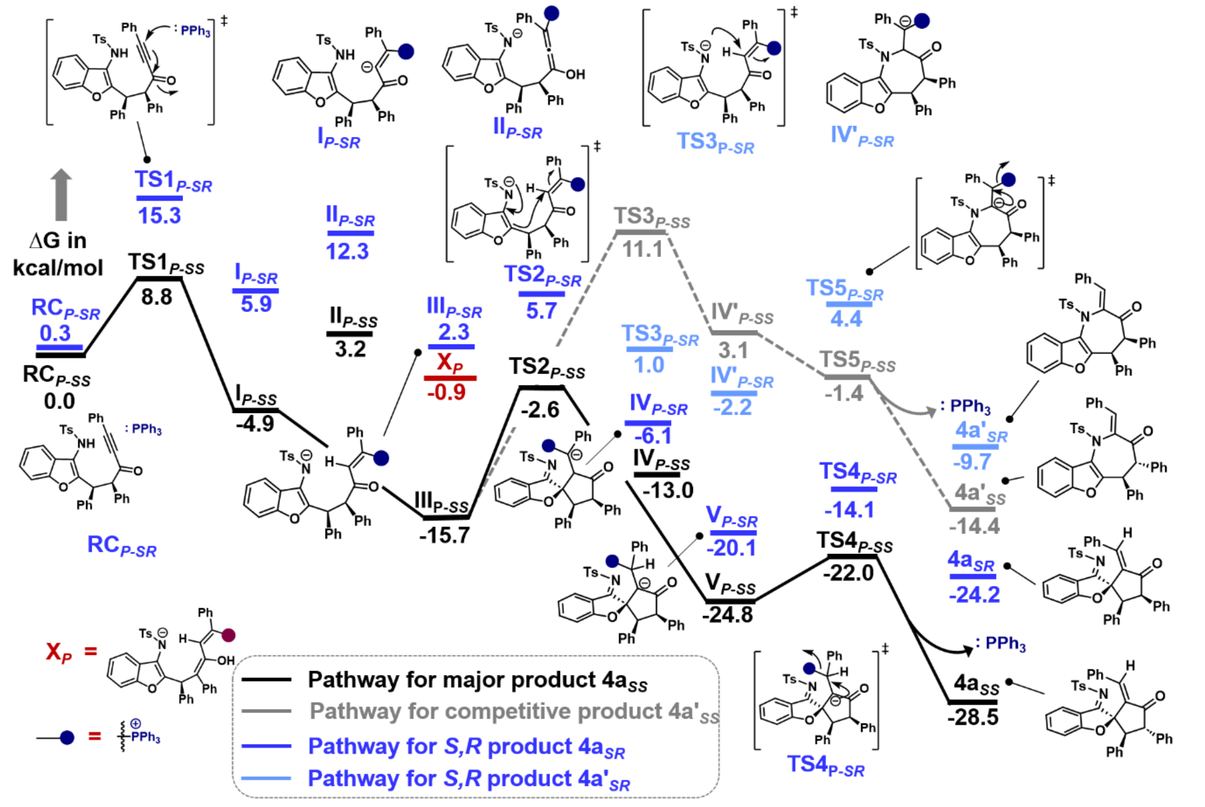

Figure S4. Gibbs free energy (kcal/mol) profile at B3LYP-D3(BJ)/CPCM(CH_2Cl_2)/def2-TZVP for the PPh_3 assisted intramolecular cyclization of intermediate $\mathbf{A}_{re\text{-}SS}$ and $\mathbf{A}_{re\text{-}SR}$. P subscript denotes PPh_3 assisted.

Unlike the DBU assisted pathway, the formation of the allene intermediate ($\mathbf{II}_{D\text{-}SS}$, Figure S3) is not a necessity for the PPh_3 assisted mechanism ($\mathbf{II}_{P\text{-}SS}$, Figure S4). As evident from Figure S4, there can be direct protonation of the α-carbanion from –NH group with a barrier less rotation starting from $\mathbf{I}_{P\text{-}SS}$ to generate the energetically favourable α,β-unsaturated keto intermediate and $\mathbf{III}_{P\text{-}SS}$. In case of the DBU assisted pathway (Figure S3), abstraction of H-bonded N-H proton by carbonyl O generate an enol in allene fashion ($\mathbf{II}_{D\text{-}SS}$) with significant stabilization as compared to $\mathbf{I}_{D\text{-}SS}$, followed by a quick keto-enol tautomerism to $\mathbf{III}_{D\text{-}SS}$. Similarly, from $\mathbf{TS2}_{P\text{-}SS}$, $\mathbf{V}_{P\text{-}SS}$ can be formed directly with the 1,2 α-proton shift to the electronically rich anionic β-C centre rather going through a local intermediate $\mathbf{IV}_{P\text{-}SS}$ (Figure S4).

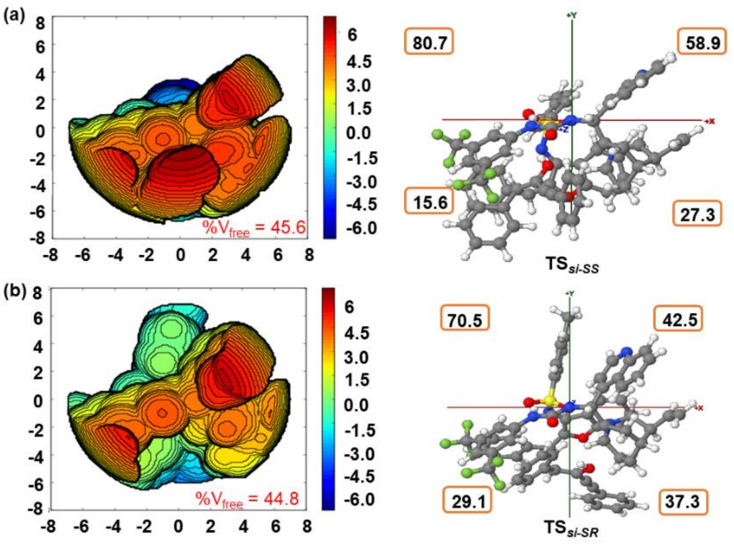

Figure S5. Steric map (within a range of ± 6.0 kcal/mol) and 3D model with xyz axes (between ± 8 Å) for **(a) TS$_{si\text{-}SS}$** and **(b) TS$_{si\text{-}SR}$**. Total %V$_{free}$ and those in each quadrant along the xyz axes are shown. Contour colour - blue: strong attractive; green: weak attractive; red: strong repulsive.

The percentage of free volume within the sphere denoted by %V$_{free}$ is used as a qualitative measure of "free" volume or "unconfined" space in the catalyst pocket that is accessible to both the reacting substrates.[13c] The greater %V$_{free}$ of **TS$_{si\text{-}SS}$** than **TS$_{si\text{-}SR}$** indicate selectivity towards formation of the major intermediate, **IntA$_{si\text{-}SS}$** instead of the minor intermediate, **IntA$_{si\text{-}SR}$**. The steric contour maps show greater attractive potentials (deep blue contour, Figure S5, top-left) presumably due to involvement of attractive dispersive effects in **TS$_{si\text{-}SS}$** as compared to **TS$_{si\text{-}SR}$** (more pronounced weakly attractive greenish contour, Figure S5, down-left).

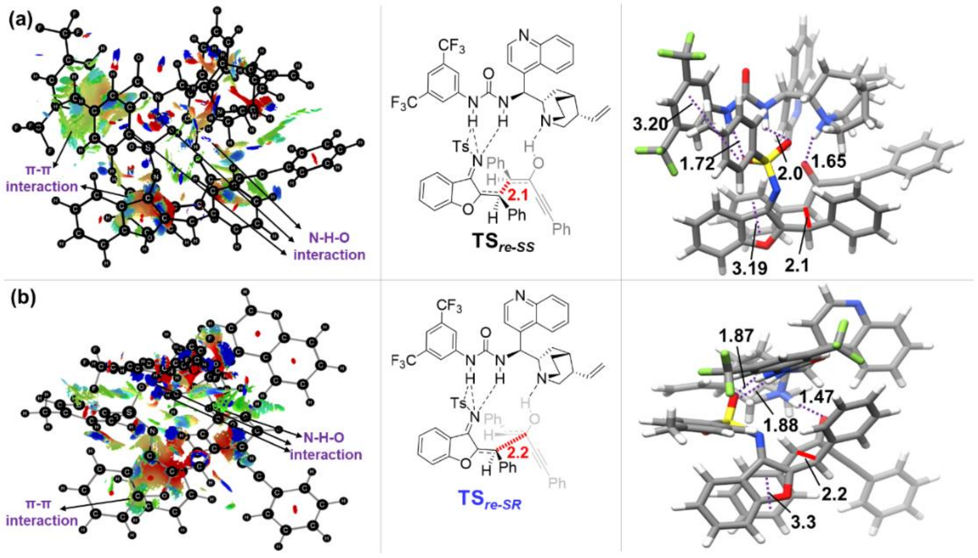

Figure S6. NCI plots with optimized geometry of (a) **TS$_{re\text{-}SS}$** and (b) **TS$_{re\text{-}SR}$**. Color Code: C(grey), H(white), N(blue), O(red), S(yellow). Blue: strong attractive; green: weak attractive; red: strong repulsive at contour value 0.52 a.u. Distances shown are in units of Å.

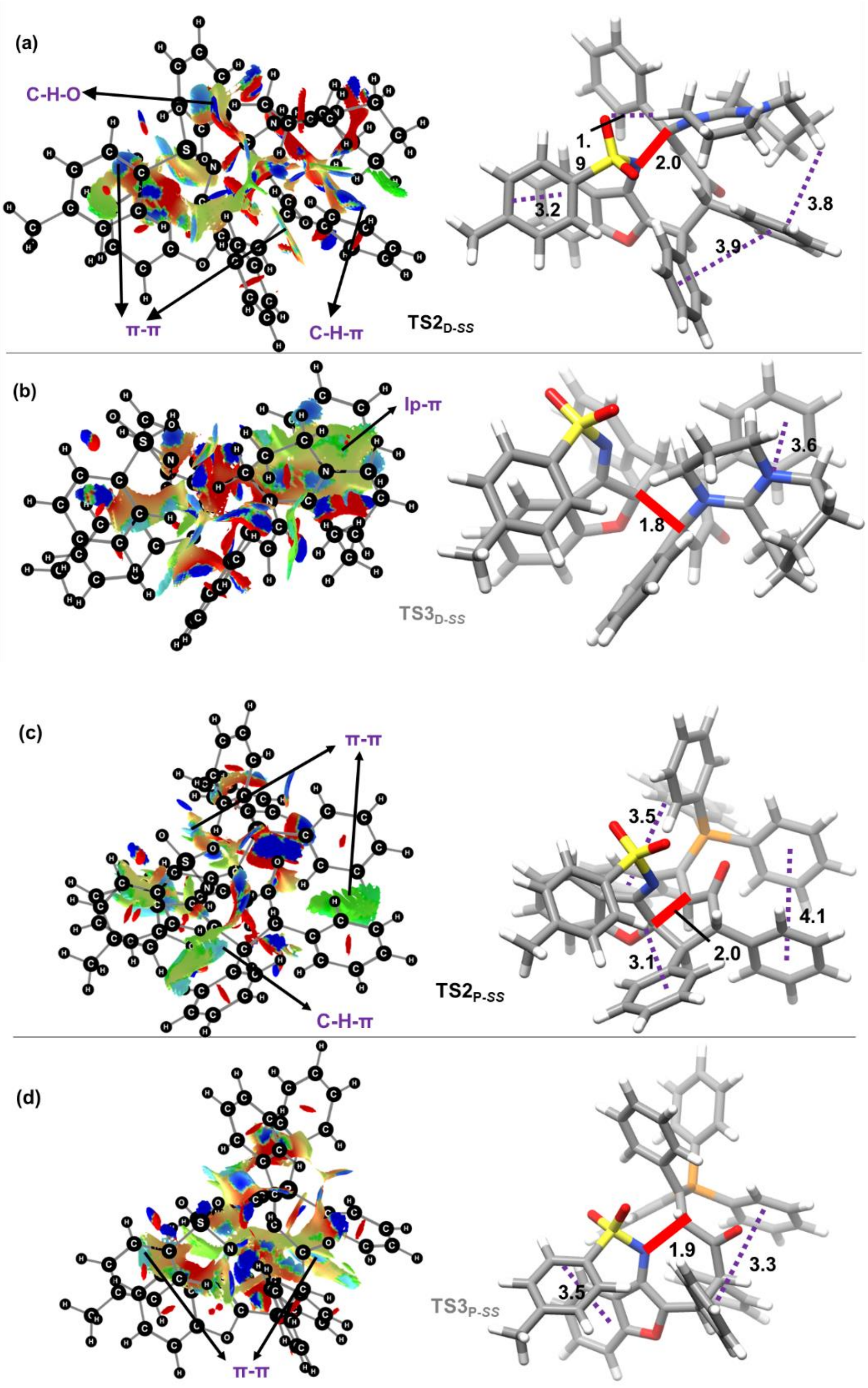

Figure S7. NCI plots with optimized geometry of (a) **TS2**$_{\textbf{\textit{D-SS}}}$ and (b) **TS3**$_{\textbf{\textit{D-SR}}}$, (c) **TS2**$_{\textbf{\textit{P-SS}}}$ (d) **TS3**$_{\textbf{\textit{P-SR}}}$. Color Code: C(grey), H(white), N(blue), O(red), S(yellow). Blue: strong attractive; green: weak attractive; red: strong repulsive at contour value 0.52 a.u. Distances shown are in units of Å.

Table S4: Relative Gibbs free energy (kcal/mol) at different levels of DFT to account for the effect of dispersion interaction.

**Stationary State**	**B3LYP-D3(BJ)/CPCM(Solvent)/def2-TZVP**	**B3LYP/CPCM(Solvent)/def2-TZVP**
**TS*re-SS***	**11.5**[a]	**19.7**[a]
**TS*re-SR***	**13.5**[a]	**13.5**[a]
**TS*si-SS***	**16.3**[b]	**22.3**[b]
**TS*si-SR***	**27.2**[b]	**24.2**[b]
**TS1*D-SS***	**12.4**[c]	**14.4**[c]
**TS2*D-SS***	**30.0**[d]	**36.2**[d]
**TS3*D-SS***	**31.3**[d]	**37.1**[d]
**TS4*D-SS***	**30.1**[d]	**33.6**[d]
**TS1*D-SR***	**20.2**[c]	**22.0**[c]
**TS2*D-SR***	**42.6**[d]	**50.4**[d]
**TS3*D-SR***	**36.5**[d]	**45.4**[d]
**TS1*P-SS***	**8.8**[e]	**11.8**[e]
**TS2*P-SS***	**13.1**[f]	**15.8**[f]
**TS3*P-SS***	**26.8**[f]	**40.7**[f]
**TS4*P-SS***	**2.8**[g]	**0.6**[g]
**TS5*P-SS***	**23.4**[g]	**31.1**[g]
**TS1*P-SR***	**15.3**[e]	**22.1**[f]
**TS2*P-SR***	**21.4**[f]	**29.5**[f]
**TS3*P-SR***	**16.7**[f]	**30.4**[f]
**TS4*P-SR***	**10.8**[g]	**13.7**[g]
**TS5*P-SR***	**29.3**[g]	**35.5**[g]

It can be observed from Table S4 that London dispersion has a large impact on the overall relative Gibbs free energy of activation for the chemoselective bifunctional urea catalyst **VI** mediated **1a** and **2a** coupling. The $\Delta G^{\ddagger}$ for **TS*re-SS*** is lowered by 8.2 kcal/mol, amounting to ~ 42 % decrease, while it is unchanged for **TS*re-SR*** on addition of empirical dispersion corrections. Further, the gain in stabilization of the transition states **TS2*D-SS*** and **TS3*D-SS*** are almost identical (~ 6 kcal/mol) suggesting that London dispersion play key role to stabilize the transition states for the observed and unobserved intramolecular cyclization in presence of DBU. Additionally, for the PPh_3 assisted cyclization, **TS3*P-SS*** leading towards the hypothetical product **4a'** is significantly lowered on addition of D3BJ, over **TS2*P-SS*** for the observed product **4a**. Hence, in both the Lewis base assisted mechanisms, non-covalent dispersive interactions presumably lead to decreased energetic requirement during geometric preparation.

Table S5: Relative Gibbs free energy (kcal/mol) at different levels of theory to account for the bifunctional urea catalyst **VI** mediated **1a** and **2a** coupling in both *re*-face and *si*-face.

Stationary State	B3LYP-D3(BJ)/CPCM(Toluene)/def2-TZVP	PBE-D3(BJ)/CPCM(Toluene)/def2-TZVP	DLPNO-CCSD(T)/CPCM(Toluene)/def2-TZVP
$TS_{re\text{-}SS}$	11.5[a]	8.8[a]	9.2[a]
$TS_{re\text{-}SR}$	13.5[a]	8.6[a]	13.3[a]
$TS_{si\text{-}SS}$	16.3[b]	10.8[b]	19.8[a]
$TS_{si\text{-}SR}$	27.2[b]	19.4[b]	31.1[a]

Table S6: Relative Gibbs free energy (kcal/mol) at different levels of theory to account for the DBU assisted intramolecular cyclization of intermediate $\mathbf{A}_{re\text{-}SS}$ and $\mathbf{A}_{re\text{-}SR}$.

Stationary State	B3LYP-D3(BJ)/CPCM(DCM)/def2-TZVP	PBE-D3(BJ)/CPCM(DCM)/def2-TZVP	DLPNO-CCSD(T)/CPCM(DCM)/def2-TZVP
$TS1_{D\text{-}SS}$	12.4[c]	7.1[c]	-
$TS2_{D\text{-}SS}$	30.0[d]	23.9[d]	25.7[d]
$TS3_{D\text{-}SS}$	31.3[d]	23.7[d]	29.8[d]
$TS4_{D\text{-}SS}$	30.1[d]	24.3[d]	-
$TS1_{D\text{-}SR}$	20.2[c]	10.7[c]	-
$TS2_{D\text{-}SR}$	42.6[d]	33.2[d]	-
$TS3_{D\text{-}SR}$	36.5[d]	26.4[d]	-
$TS1_{D.d\text{-}SS}$	-0.3[h]	-	
$TS2_{D.d\text{-}SS}$	19.4[i]	-	20.4[i]
$TS3_{D.d\text{-}SS}$	23.7[i]	-	23.5[i]
$TS1_{D.d\text{-}SR}$	5.9[i]	-	-
$TS2_{D.d\text{-}SR}$	23.5[i]	-	-
$TS3_{D.d\text{-}SR}$	28.5[i]	-	-

Table S7: Relative Gibbs free energy (kcal/mol) at different levels of DFT to account for the effect of implicit solvent model for DBU assisted intramolecular cyclization of $\mathbf{A}_{re\text{-}SS}$.

Stationary State	B3LYP-D3(BJ)/SMD(DCM)/def2-TZVP	PBE-D3(BJ)/SMD(DCM)/def2-TZVP
$TS2_{D\text{-}SS}$	30.9[d]	24.8[d]
$TS3_{D\text{-}SS}$	33.1[d]	25.4[d]

Table S8: Relative Gibbs free energy (kcal/mol) at different levels of theory to account for the PPh_3 assisted intramolecular cyclization of intermediate $\mathbf{A}_{re\text{-}SS}$ and $\mathbf{A}_{re\text{-}SR}$

Stationary State	B3LYP-D3(BJ)/CPCM(Toluene)/def2-TZVP	PBE-D3(BJ)/CPCM(Toluene)/def2-TZVP	DLPNO-CCSD(T)/CPCM(DCM)/def2-TZVP
$TS1_{P\text{-}SS}$	8.8[e]	4.3[e]	-
$TS2_{P\text{-}SS}$	13.1[f]	10.7[f]	11.2[f]

**$TS3_{P\text{-}SS}$**	**26.8^{f}**	**27.5^{f}**	**22.5^{f}**
**$TS4_{P\text{-}SS}$**	**2.8^{g}**	**3.3^{g}**	**-**
**$TS5_{P\text{-}SS}$**	**23.4^{g}**	**21.5^{g}**	**-**
**$TS1_{P\text{-}SR}$**	**15.3^{e}**	**10.0^{e}**	**-**
**$TS2_{P\text{-}SR}$**	**21.4^{f}**	**19.1^{f}**	**-**
**$TS3_{P\text{-}SR}$**	**16.7^{f}**	**17.6^{f}**	**-**
**$TS4_{P\text{-}SR}$**	**10.8^{g}**	**10.6^{g}**	**-**
**$TS5_{P\text{-}SR}$**	**29.3^{g}**	**27.3^{g}**	**-**

^{a}w.r.t. **$RC_{re\text{-}SS}$**, ^{b}w.r.t. **$RC_{si\text{-}SS}$**, ^{c}w.r.t. **$RC_{D\text{-}SS}$**, ^{d}w.r.t. **$III_{D\text{-}SS}$**, ^{e}w.r.t. **$RC_{P\text{-}SS}$**, ^{f}w.r.t. **$III_{P\text{-}SS}$**, ^{g}w.r.t. **$V_{P\text{-}SS}$**, ^{h}w.r.t. **$RC_{D.d\text{-}SS}$**, ^{i}w.r.t. **$I_{D.d\text{-}SS}$**

From **Tables S5** and **S6**, it is found that the computationally less demanding PBE functional falters to properly evaluate the $\Delta\Delta G^{\ddagger}$ in the solvent phase for **$TS_{re\text{-}SS}$/$TS_{re\text{-}SR}$** and **$TS2_{D\text{-}SS}$/$TS3_{D\text{-}SS}$** pairs. This is presumably because of the lack of proper accounting of the electronic exchange enegies unlike B3LYP-D3(BJ) and DLPNO-CCSD(T), indicating less credibility of the PBE functional for energetic evaluations, although it provides reasonably reliable geometries in the gas phase.[13] Further, from **Tables S6** and **S7**, it is evident that the implicit solvent model (CPCM versus SMD) has little role to play on the transition state energies.

pK_a Calculation:

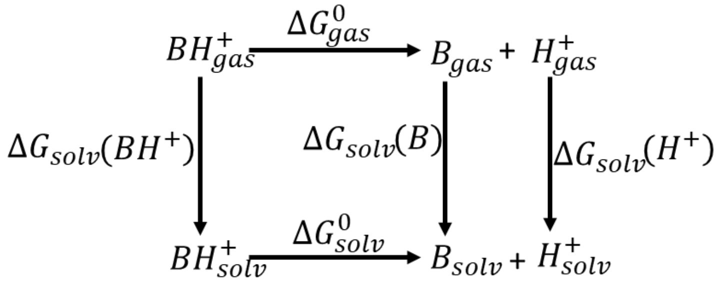

Scheme S1. Thermochemical cycle for the calculation of pK_a of DBU and PPh_3 in DCM solvent.

Here we have employed a standard protocol for computational estimation of pK_a of DBU and PPh_3 adopted by Carter and others[18] using the above Born-Haber thermochemical cycle as follows.

With the help of the above thermochemical cycle in Scheme S1, solvation Gibbs free energy (ΔG^{0}_{solv}) can be predicted as:

$$\Delta G^{0}_{solv} = \Delta G^{0}_{gas} + \Delta G_{solv}(B) + \Delta G_{solv}(H^{+}) - \Delta G_{solv}(BH^{+}) \qquad (1)$$

Hence, from equation (1) the pK_a of the target conjugate acid (BH^+) is given by:

$$\text{direct p}K_a = -\log K_a = \Delta G^{0}_{solv} / 2.303\,RT \qquad (2)$$

In equation (1) we employed an absolute proton solvation energy, ΔG_{solv} (H^+) = –264.0 kcal/mol. This was corrected by 1.9 kcal/mol for transferring a proton from 1 atm gas phase to a standard state of 1 M in solution phase which makes ΔG_{solv} (H^+) = -262.1 kcal/mol. To be noted that the absolute free energy of proton in gas phase at standard temperature and pressure is G^0_{gas} (H^+) = - 6.3 kcal/mol which can be evaluated with the Sackur-Tetrode equation.

Species	Calculated pK_a
$DBUH^+$	16.8
PPh_3H^+	2.8

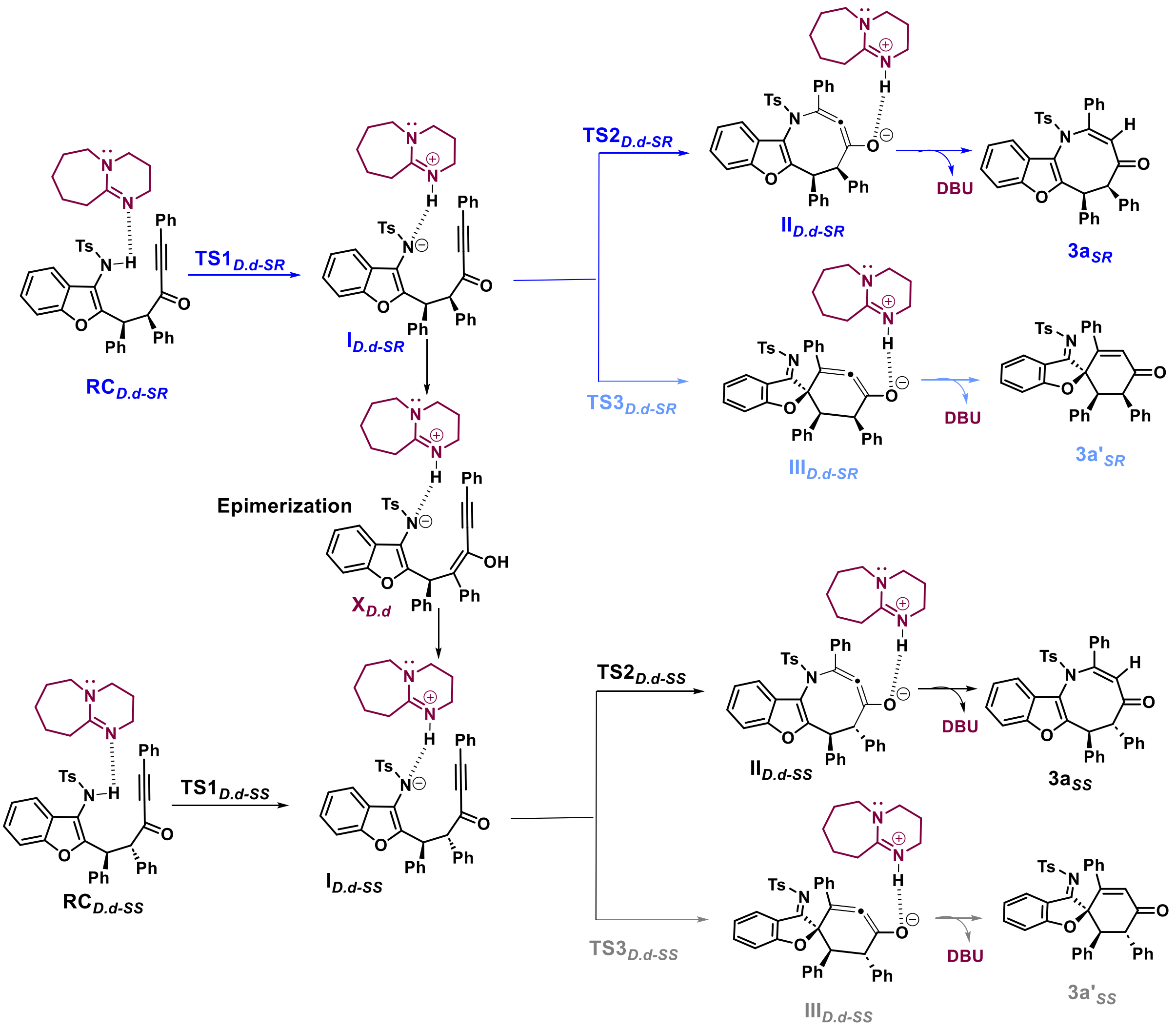

Scheme S2. Alternative reaction pathway for DBU acting as a Brønsted base.

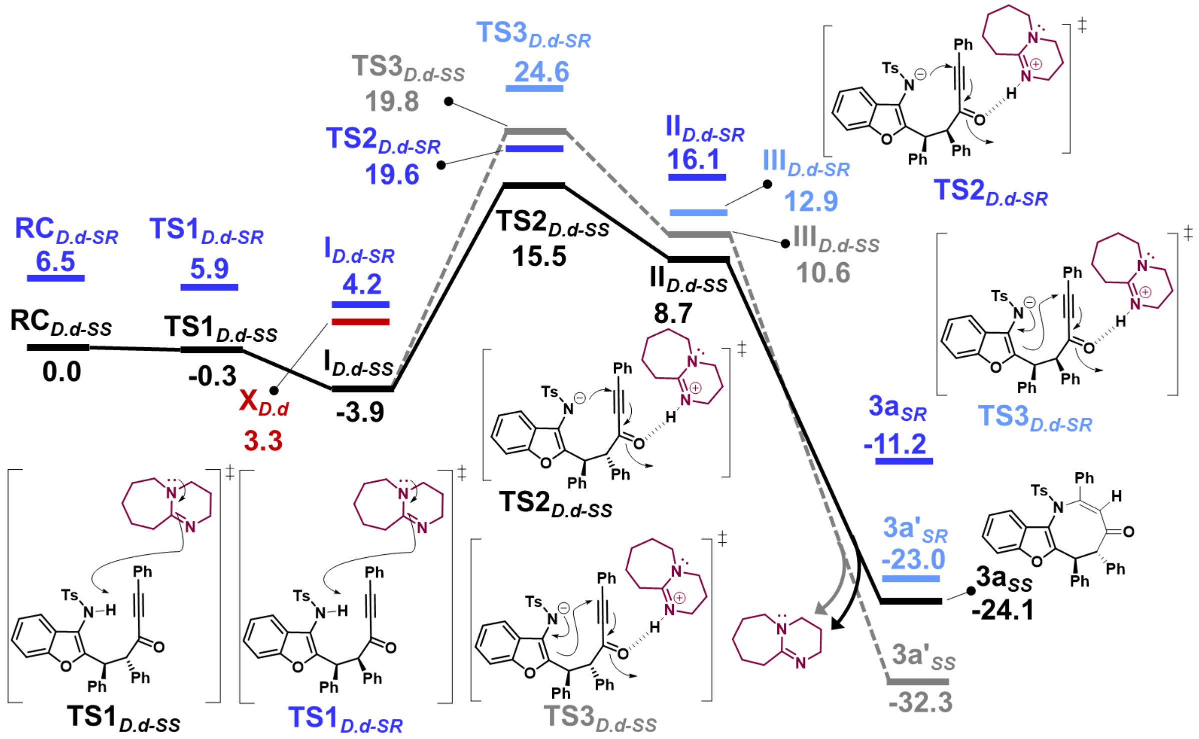

Figure S8. Gibbs free energy (kcal/mol) profile at B3LYP-D3(BJ)/CPCM(CH_2Cl_2)/def2-TZVP for the alternative DBU assisted deprotonative activation of TsNH moiety and intramolecular cyclization of intermediates $\mathbf{A}_{re\text{-}SS}$ and $\mathbf{A}_{re\text{-}SR}$. D.d subscript denotes DBU assisted deprotonation pathway for Reaction Scheme S1. Black pathway for major product $\mathbf{3a}_{SS}$, Grey pathway for product $\mathbf{3a'}_{SS}$, deep blue pathway for SR product $\mathbf{3a}_{SR}$ and faint blue pathway for SR product $\mathbf{3a'}_{SR}$.

Our calculations predict that DBU is fairly basic (pK_a=16.8) as compared to PPh_3 (pK_a=2.8) as shown earlier. We hypothesize an alternative pathway where DBU deprotonates the TsNH proton ($\mathbf{TS1}_{D.d\text{-}SS}$, Figure S8) to generate an ion salt pair $\mathbf{I}_{D.d\text{-}SS}$ which can further undergo a direct Michael addition ($\mathbf{TS2}_{D.d\text{-}SS}$). This leads to a stable allene-type eight membered ring intermediate $\mathbf{II}_{D.d\text{-}SS}$ at an energetic expense of 19.4 kcal/mol. From here a quick proton exchange occurs from $DBUH^+$ to the anionic α-centre of $\mathbf{II}_{D.d\text{-}SS}$ leading to our desired product, $\mathbf{3a}_{SS}$ (Figure S4). Similarly, we have analysed the pathway leading to the *SR* product, $\mathbf{3a}_{SR}$.as shown in Figure S4. The energetic requirements are in agreement to the experimental observations. It may be plausible that the enolised intermediate $\mathbf{X}_{D.d}$ undergoes epimerisation to convert to the highly stable, $\mathbf{I}_{D.d\text{-}S}$ to avoid formation of *SR* diastereomeric product. Moreover, after repeated attempts, the initial deprotonation pathway could not be traced with PPh_3 due to its low basicity and hence not explored any further.

XYZ Cartesian coordinates of computed species

1a

Electronic energy = -1526.993747 Hartree

C	0.690571000000	4.102800000000	-2.937591000000
C	1.026334000000	3.046862000000	-2.081467000000
C	-0.656322000000	4.470499000000	-3.147201000000
H	2.072312000000	2.760630000000	-1.917173000000
H	-0.889479000000	5.305087000000	-3.825852000000
C	-0.016958000000	2.356743000000	-1.430959000000
C	-1.710443000000	3.796568000000	-2.509400000000
H	-2.762616000000	4.072368000000	-2.663421000000
C	-1.356795000000	2.747451000000	-1.660307000000
H	1.489049000000	4.654428000000	-3.454500000000
C	-1.532376000000	1.051590000000	-0.233167000000
C	-0.082605000000	1.233171000000	-0.488850000000
O	-2.255711000000	1.982847000000	-0.958184000000
C	-2.095213000000	0.117499000000	0.584824000000
H	-1.339368000000	-0.524316000000	1.067019000000
C	-3.487763000000	-0.143633000000	0.898358000000
C	-3.782170000000	-1.201675000000	1.799133000000
C	-4.573276000000	0.596498000000	0.355784000000
H	-2.949830000000	-1.782720000000	2.225744000000
H	-4.368290000000	1.417540000000	-0.342603000000
C	-5.101188000000	-1.510297000000	2.145405000000
C	-5.890065000000	0.282708000000	0.706315000000
H	-5.305631000000	-2.334629000000	2.845198000000
H	-6.718958000000	0.865769000000	0.276853000000
C	-6.162254000000	-0.768442000000	1.599785000000
H	-7.201596000000	-1.008923000000	1.870192000000
N	0.789912000000	0.454932000000	0.096438000000
S	2.448628000000	0.663597000000	-0.204336000000
O	2.900468000000	1.964540000000	0.349187000000
O	2.750174000000	0.315498000000	-1.615282000000
C	3.091121000000	-0.641038000000	0.842541000000
C	3.400850000000	-0.355606000000	2.179732000000
C	3.287571000000	-1.917766000000	0.301469000000
H	3.251419000000	0.663622000000	2.563997000000
H	3.050847000000	-2.099291000000	-0.756735000000
C	3.904412000000	-1.380510000000	2.989004000000
C	3.793140000000	-2.930447000000	1.127524000000
H	4.150043000000	-1.167698000000	4.041493000000
H	3.951019000000	-3.938634000000	0.713297000000
C	4.108270000000	-2.681905000000	2.479776000000
C	4.678447000000	-3.766838000000	3.357140000000
H	5.775111000000	-3.638468000000	3.481958000000
H	4.234722000000	-3.743545000000	4.372687000000
H	4.509954000000	-4.773005000000	2.927189000000

2a

Electronic energy = -691.9844152 Hartree

C	-0.742756000000	-0.735743000000	0.164700000000
O	-1.211697000000	-1.828758000000	-0.130168000000
C	0.683703000000	-0.477187000000	0.112311000000
C	-1.595369000000	0.446486000000	0.653650000000
H	-1.145366000000	1.389367000000	0.281038000000
H	-1.452693000000	0.468867000000	1.759372000000
C	-3.057737000000	0.356932000000	0.302674000000
C	-3.860113000000	-0.687953000000	0.805608000000
C	-3.645647000000	1.307822000000	-0.553842000000
H	-3.411828000000	-1.446058000000	1.464005000000
H	-3.027587000000	2.125685000000	-0.957944000000
C	-5.217095000000	-0.772441000000	0.463584000000
C	-5.004435000000	1.226550000000	-0.895111000000
H	-5.829799000000	-1.594725000000	0.863872000000
H	-5.446999000000	1.981493000000	-1.562901000000
C	-5.794451000000	0.184532000000	-0.386652000000
H	-6.860426000000	0.117397000000	-0.652649000000
C	1.895947000000	-0.257545000000	0.064008000000
C	3.300466000000	-0.030778000000	0.001006000000
C	3.844504000000	1.234304000000	0.343173000000
C	4.175722000000	-1.071115000000	-0.406261000000
H	3.166357000000	2.040369000000	0.658454000000
H	3.752634000000	-2.050668000000	-0.671257000000
C	5.225641000000	1.447464000000	0.278955000000
C	5.555028000000	-0.846114000000	-0.467685000000
H	5.638836000000	2.431596000000	0.546541000000
H	6.226249000000	-1.658265000000	-0.785017000000

C	6.083259000000	0.410496000000	-0.125896000000
H	7.169095000000	0.582711000000	-0.175457000000

RCWC*si-SR*

Electronic energy = -2218.994508 Hartree

C	0.461426000000	0.471839000000	2.341653000000
O	1.643633000000	0.454274000000	3.009359000000
C	-0.194912000000	-0.793800000000	2.286470000000
C	-0.117264000000	1.564797000000	1.730271000000
H	2.315036000000	1.017078000000	2.527248000000
H	-1.023927000000	1.344367000000	1.146607000000
C	0.287034000000	2.962486000000	1.760744000000
C	1.214650000000	3.502871000000	2.693413000000
C	-0.317532000000	3.861208000000	0.840982000000
H	1.684429000000	2.842562000000	3.433673000000
H	-1.041664000000	3.467244000000	0.111718000000
C	1.515405000000	4.869785000000	2.696781000000
C	-0.002997000000	5.224497000000	0.838925000000
H	2.232825000000	5.261072000000	3.434127000000
H	-0.482143000000	5.889847000000	0.103969000000
C	0.917639000000	5.740280000000	1.767616000000
H	1.164739000000	6.812808000000	1.772189000000
C	-0.717510000000	-1.908493000000	2.222211000000
C	-1.251020000000	-3.217810000000	2.080321000000
C	-2.171529000000	-3.515345000000	1.041450000000
C	-0.826232000000	-4.267868000000	2.936575000000
H	-2.495692000000	-2.706602000000	0.371203000000
H	-0.108020000000	-4.041513000000	3.737574000000
C	-2.639206000000	-4.821949000000	0.862235000000
C	-1.303782000000	-5.569511000000	2.750723000000
H	-3.347700000000	-5.039157000000	0.048344000000
H	-0.965028000000	-6.373641000000	3.421792000000
C	-2.208456000000	-5.854581000000	1.712965000000
H	-2.579067000000	-6.880602000000	1.568557000000
C	3.605559000000	-3.284271000000	1.550348000000
C	3.524471000000	-1.980955000000	1.051329000000
C	2.624194000000	-4.250883000000	1.229822000000
H	4.294247000000	-1.235087000000	1.284652000000
H	2.713652000000	-5.267472000000	1.642232000000
C	2.430769000000	-1.653331000000	0.221099000000
C	1.528702000000	-3.947251000000	0.409131000000
H	0.745886000000	-4.680670000000	0.174137000000
C	1.456889000000	-2.637903000000	-0.070610000000
H	4.445825000000	-3.562714000000	2.202495000000
C	0.691891000000	-0.801018000000	-1.076462000000
C	1.989947000000	-0.440285000000	-0.458915000000
O	0.444394000000	-2.152998000000	-0.843039000000
C	-0.321789000000	-0.115345000000	-1.694409000000
H	-1.192253000000	-0.769191000000	-1.877779000000
C	-0.519748000000	1.262903000000	-2.109382000000
C	0.496893000000	2.248386000000	-2.223894000000
C	-1.852309000000	1.639765000000	-2.441862000000
H	1.525389000000	1.979360000000	-1.955481000000
H	-2.652911000000	0.886593000000	-2.372868000000
C	0.185648000000	3.546845000000	-2.640440000000
C	-2.159419000000	2.943651000000	-2.839547000000
H	0.994057000000	4.290221000000	-2.711184000000
H	-3.199497000000	3.212582000000	-3.077936000000
C	-1.138180000000	3.905393000000	-2.942855000000
H	-1.377225000000	4.931725000000	-3.260967000000
N	2.484351000000	0.769019000000	-0.588406000000
S	3.888625000000	1.301425000000	0.151941000000
O	5.068141000000	0.451207000000	-0.140882000000
O	3.580963000000	1.630040000000	1.581494000000
C	4.075727000000	2.849086000000	-0.724575000000
C	5.004390000000	2.925385000000	-1.772154000000
C	3.267864000000	3.939692000000	-0.376142000000
H	5.624854000000	2.049866000000	-2.011543000000
H	2.552748000000	3.862467000000	0.453283000000
C	5.114422000000	4.123930000000	-2.487878000000
C	3.393703000000	5.128122000000	-1.106062000000
H	5.839302000000	4.193911000000	-3.314157000000
H	2.757936000000	5.985268000000	-0.833922000000
C	4.312855000000	5.242487000000	-2.170764000000
C	4.460827000000	6.534170000000	-2.932784000000
H	3.568261000000	7.179780000000	-2.821395000000
H	5.334448000000	7.112279000000	-2.561990000000
H	4.629180000000	6.353751000000	-4.013212000000

TSWC$_{si\text{-}SR}$

Electronic energy = -2218.939682 Hartree

C	-1.545215000000	-0.244094000000	1.443286000000
O	-0.727329000000	-0.744692000000	2.379662000000
C	-2.679068000000	-0.985137000000	1.109508000000
C	-1.229682000000	0.990678000000	0.742667000000
H	-0.046534000000	-0.056213000000	2.590407000000
H	-2.160143000000	1.470087000000	0.395515000000
C	-0.342274000000	1.963774000000	1.455486000000
C	0.930943000000	1.606934000000	1.959920000000
C	-0.789587000000	3.289144000000	1.646414000000
H	1.381837000000	0.628358000000	1.730063000000
H	-1.765416000000	3.589606000000	1.235000000000
C	1.714041000000	2.536282000000	2.664774000000
C	-0.006074000000	4.218110000000	2.341828000000
H	2.714005000000	2.229000000000	3.002664000000
H	-0.373946000000	5.246622000000	2.476615000000
C	1.243521000000	3.841618000000	2.864369000000
H	1.857578000000	4.572929000000	3.411446000000
C	-3.691510000000	-1.605206000000	0.751497000000
C	-4.841811000000	-2.327794000000	0.360062000000
C	-5.687814000000	-1.842761000000	-0.676479000000
C	-5.172720000000	-3.556444000000	0.997834000000
H	-5.435404000000	-0.894529000000	-1.172721000000
H	-4.521007000000	-3.934062000000	1.798672000000
C	-6.821349000000	-2.566057000000	-1.056435000000
C	-6.310750000000	-4.267199000000	0.608015000000
H	-7.467126000000	-2.181659000000	-1.860271000000
H	-6.557018000000	-5.215693000000	1.108681000000
C	-7.138727000000	-3.778175000000	-0.418078000000
H	-8.032842000000	-4.343004000000	-0.721557000000
C	3.470484000000	-3.634005000000	0.613273000000
C	3.532802000000	-2.281496000000	0.249726000000
C	2.250964000000	-4.345752000000	0.598860000000
H	4.484740000000	-1.734291000000	0.243375000000
H	2.238332000000	-5.406377000000	0.892397000000
C	2.335621000000	-1.638429000000	-0.127253000000
C	1.050051000000	-3.726028000000	0.215958000000
H	0.089044000000	-4.258311000000	0.197823000000
C	1.133606000000	-2.379420000000	-0.136754000000
H	4.393260000000	-4.152546000000	0.912689000000
C	0.553472000000	-0.320238000000	-0.767018000000
C	1.972683000000	-0.269568000000	-0.533919000000
O	0.076871000000	-1.602260000000	-0.529062000000
C	-0.543569000000	0.608587000000	-0.911714000000
H	-1.410921000000	0.030356000000	-1.281546000000
C	-0.494748000000	1.934316000000	-1.609236000000
C	0.612023000000	2.808402000000	-1.582578000000
C	-1.676782000000	2.362739000000	-2.257487000000
H	1.540855000000	2.468931000000	-1.101688000000
H	-2.552332000000	1.692689000000	-2.283174000000
C	0.537991000000	4.065667000000	-2.199657000000
C	-1.749345000000	3.619128000000	-2.872396000000
H	1.420346000000	4.724004000000	-2.171694000000
H	-2.675966000000	3.927481000000	-3.380218000000
C	-0.638060000000	4.478583000000	-2.843531000000
H	-0.691546000000	5.466576000000	-3.326160000000
N	2.688969000000	0.848113000000	-0.666473000000
S	4.239970000000	0.996745000000	-0.103751000000
O	5.227364000000	0.128267000000	-0.802959000000
O	4.224217000000	0.975392000000	1.388037000000
C	4.540647000000	2.685384000000	-0.648692000000
C	5.476039000000	2.911951000000	-1.664222000000
C	3.835772000000	3.747766000000	-0.061475000000
H	6.008690000000	2.054132000000	-2.099415000000
H	3.106097000000	3.552675000000	0.736481000000
C	5.701268000000	4.225410000000	-2.102924000000
C	4.066911000000	5.050149000000	-0.516789000000
H	6.433914000000	4.409699000000	-2.904462000000
H	3.509164000000	5.885757000000	-0.064132000000
C	5.002518000000	5.313306000000	-1.543011000000
C	5.257156000000	6.726008000000	-2.003571000000
H	4.309728000000	7.283869000000	-2.148755000000
H	5.848526000000	7.289053000000	-1.250041000000
H	5.821773000000	6.751338000000	-2.955510000000

A$_{si\text{-}SR}$

Electronic energy = -2219.003013 Hartree

C	-1.626330000000	-0.521433000000	1.696439000000
O	-1.111990000000	-1.137857000000	2.623837000000
C	-2.903792000000	-0.908763000000	1.128172000000
C	-0.992067000000	0.674234000000	0.968057000000
H	2.667947000000	1.349516000000	1.433684000000
H	-1.834674000000	1.289230000000	0.592789000000
C	-0.073829000000	1.562596000000	1.772694000000
C	0.775055000000	1.071093000000	2.789632000000
C	-0.003856000000	2.932457000000	1.438504000000
H	0.716553000000	0.011217000000	3.065897000000
H	-0.665965000000	3.331149000000	0.655345000000
C	1.683671000000	1.929260000000	3.432156000000
C	0.913787000000	3.784244000000	2.069705000000
H	2.340016000000	1.529976000000	4.220304000000
H	0.966400000000	4.843351000000	1.775611000000
C	1.766223000000	3.284329000000	3.066561000000
H	2.488459000000	3.948819000000	3.565211000000
C	-3.973556000000	-1.196910000000	0.587416000000
C	-5.204822000000	-1.521524000000	-0.047967000000
C	-5.689745000000	-0.715439000000	-1.111105000000
C	-5.960061000000	-2.649593000000	0.364203000000
H	-5.103632000000	0.159860000000	-1.427874000000
H	-5.582618000000	-3.273189000000	1.187213000000
C	-6.896607000000	-1.034027000000	-1.741889000000
C	-7.166385000000	-2.957625000000	-0.273531000000
H	-7.265302000000	-0.404439000000	-2.565513000000
H	-7.746393000000	-3.834156000000	0.051984000000
C	-7.637149000000	-2.153579000000	-1.325626000000
H	-8.586609000000	-2.401079000000	-1.823936000000
C	4.485350000000	-3.333144000000	1.110649000000
C	4.218246000000	-1.966135000000	0.974975000000
C	3.502883000000	-4.312263000000	0.826826000000
H	4.982047000000	-1.206578000000	1.194203000000
H	3.752791000000	-5.377568000000	0.942762000000
C	2.929743000000	-1.586858000000	0.544448000000
C	2.214557000000	-3.953400000000	0.404572000000
H	1.436404000000	-4.698117000000	0.187250000000
C	1.964750000000	-2.584337000000	0.280541000000
H	5.482317000000	-3.656693000000	1.445344000000
C	0.959581000000	-0.636514000000	-0.095468000000
C	2.257400000000	-0.326081000000	0.290747000000
O	0.780855000000	-2.006537000000	-0.093015000000
C	-0.317885000000	0.100390000000	-0.368018000000
H	-1.008654000000	-0.697907000000	-0.708756000000
C	-0.344749000000	1.151529000000	-1.466394000000
C	0.651995000000	2.130124000000	-1.642183000000
C	-1.482369000000	1.188558000000	-2.301877000000
H	1.544222000000	2.108736000000	-1.002110000000
H	-2.265809000000	0.422367000000	-2.178229000000
C	0.504304000000	3.124261000000	-2.622863000000
C	-1.628175000000	2.179079000000	-3.282597000000
H	1.295031000000	3.880330000000	-2.738175000000
H	-2.520126000000	2.184307000000	-3.927808000000
C	-0.632996000000	3.156531000000	-3.443135000000
H	-0.742557000000	3.938100000000	-4.210461000000
N	2.785440000000	0.972675000000	0.477704000000
S	4.374204000000	1.324797000000	-0.079414000000
O	4.627656000000	0.457546000000	-1.244956000000
O	5.282360000000	1.364249000000	1.091369000000
C	4.182068000000	3.015814000000	-0.644633000000
C	4.057973000000	3.259498000000	-2.017927000000
C	4.175234000000	4.065610000000	0.287831000000
H	4.082057000000	2.414758000000	-2.721200000000
H	4.308526000000	3.848565000000	1.357158000000
C	3.908930000000	4.582328000000	-2.459367000000
C	4.007796000000	5.375957000000	-0.171411000000
H	3.818637000000	4.783861000000	-3.538419000000
H	3.993685000000	6.204891000000	0.553999000000
C	3.872197000000	5.658548000000	-1.549485000000
C	3.717100000000	7.079672000000	-2.025989000000
H	2.920235000000	7.609432000000	-1.465121000000
H	4.654437000000	7.654922000000	-1.871048000000
H	3.469619000000	7.126508000000	-3.103737000000

RCWC$_{si\text{-}SS}$

Electronic energy = -2218.989263 Hartree

C	-1.250276000000	-0.603836000000	2.085639000000
O	-0.293435000000	-1.421692000000	2.585544000000

C	-2.324077000000	-1.314372000000	1.480286000000
C	-1.149236000000	0.773942000000	2.160181000000
H	0.567664000000	-0.918653000000	2.644822000000
H	-0.248774000000	1.133671000000	2.684861000000
C	-2.020584000000	1.824923000000	1.664525000000
C	-1.717261000000	3.160844000000	2.045739000000
C	-3.135746000000	1.631069000000	0.803158000000
H	-0.840804000000	3.339460000000	2.687697000000
H	-3.380793000000	0.618755000000	0.458335000000
C	-2.495099000000	4.239742000000	1.618754000000
C	-3.912961000000	2.714966000000	0.378049000000
H	-2.224576000000	5.261369000000	1.924993000000
H	-4.771005000000	2.533983000000	-0.288454000000
C	-3.603367000000	4.024365000000	0.781854000000
H	-4.213720000000	4.872935000000	0.438258000000
C	-3.186657000000	-1.986887000000	0.912108000000
C	-4.162645000000	-2.736301000000	0.202018000000
C	-5.357098000000	-2.122056000000	-0.258397000000
C	-3.946571000000	-4.111239000000	-0.081366000000
H	-5.530424000000	-1.059094000000	-0.034859000000
H	-3.021795000000	-4.586524000000	0.276126000000
C	-6.298069000000	-2.861546000000	-0.982804000000
C	-4.895613000000	-4.840294000000	-0.805937000000
H	-7.219900000000	-2.373508000000	-1.334009000000
H	-4.717510000000	-5.905547000000	-1.018359000000
C	-6.072679000000	-4.220800000000	-1.260338000000
H	-6.816633000000	-4.798670000000	-1.829100000000
C	0.860105000000	-3.541447000000	-0.284109000000
C	1.274419000000	-2.249978000000	0.051540000000
C	-0.248646000000	-3.753877000000	-1.135700000000
H	2.118347000000	-2.092834000000	0.731270000000
H	-0.557055000000	-4.783199000000	-1.374821000000
C	0.568539000000	-1.152612000000	-0.490379000000
C	-0.974957000000	-2.684336000000	-1.675738000000
H	-1.850671000000	-2.833076000000	-2.321549000000
C	-0.544157000000	-1.399775000000	-1.333344000000
H	1.396417000000	-4.406353000000	0.131947000000
C	-0.415914000000	0.822161000000	-1.224107000000
C	0.686918000000	0.299385000000	-0.399845000000

O	-1.128259000000	-0.252515000000	-1.764834000000
C	-0.931225000000	2.060249000000	-1.527521000000
H	-1.830320000000	1.962593000000	-2.159267000000
C	-0.588856000000	3.434336000000	-1.221990000000
C	0.503319000000	3.868263000000	-0.421040000000
C	-1.453368000000	4.425473000000	-1.768173000000
H	1.169606000000	3.114468000000	0.019847000000
H	-2.308872000000	4.103890000000	-2.381571000000
C	0.706032000000	5.230999000000	-0.185217000000
C	-1.239629000000	5.785500000000	-1.532216000000
H	1.553303000000	5.543739000000	0.443810000000
H	-1.923163000000	6.531451000000	-1.964988000000
C	-0.156965000000	6.194759000000	-0.735899000000
H	0.014013000000	7.265422000000	-0.544884000000
N	1.540067000000	1.128561000000	0.174249000000
S	2.636154000000	0.753186000000	1.362091000000
O	2.097595000000	-0.242401000000	2.354735000000
O	3.099721000000	2.052169000000	1.882814000000
C	4.010968000000	-0.045322000000	0.526186000000
C	4.621340000000	-1.163088000000	1.110771000000
C	4.501975000000	0.509581000000	-0.667149000000
H	4.223388000000	-1.567082000000	2.053238000000
H	4.016748000000	1.395011000000	-1.103481000000
C	5.729947000000	-1.741539000000	0.477284000000
C	5.604311000000	-0.087006000000	-1.287944000000
H	6.214639000000	-2.619251000000	0.932995000000
H	5.989010000000	0.339444000000	-2.227831000000
C	6.236588000000	-1.221030000000	-0.730627000000
C	7.410418000000	-1.861372000000	-1.425309000000
H	7.082109000000	-2.406964000000	-2.335622000000
H	8.148676000000	-1.102621000000	-1.754908000000
H	7.928672000000	-2.586783000000	-0.769421000000

TSWC$_{si\text{-}SS}$

Electronic energy = -2218.948813 Hartree

C	-1.880950000000	-0.418850000000	0.873529000000
O	-0.909802000000	-1.242793000000	1.218500000000
C	-3.173113000000	-0.972298000000	0.765326000000
C	-1.578055000000	0.946795000000	0.578249000000

H	-0.003746000000	-0.754713000000	1.347772000000
H	-0.740194000000	1.305406000000	1.200188000000
C	-2.596350000000	1.974784000000	0.275234000000
C	-2.462932000000	3.258185000000	0.851405000000
C	-3.652058000000	1.757209000000	-0.641438000000
H	-1.623568000000	3.451532000000	1.536804000000
H	-3.749796000000	0.781747000000	-1.139283000000
C	-3.374207000000	4.280133000000	0.553676000000
C	-4.563169000000	2.779739000000	-0.937371000000
H	-3.251467000000	5.270717000000	1.016924000000
H	-5.376745000000	2.591840000000	-1.655140000000
C	-4.432406000000	4.043702000000	-0.337213000000
H	-5.147104000000	4.846065000000	-0.575047000000
C	-4.330950000000	-1.394429000000	0.682261000000
C	-5.678419000000	-1.814403000000	0.553810000000
C	-6.686680000000	-0.857248000000	0.254929000000
C	-6.042208000000	-3.178028000000	0.713871000000
H	-6.400907000000	0.198854000000	0.139672000000
H	-5.261344000000	-3.916314000000	0.945949000000
C	-8.017509000000	-1.261918000000	0.116503000000
C	-7.377992000000	-3.567023000000	0.575702000000
H	-8.792699000000	-0.515851000000	-0.114455000000
H	-7.652817000000	-4.624988000000	0.702074000000
C	-8.367577000000	-2.614253000000	0.276446000000
H	-9.417160000000	-2.927105000000	0.169365000000
C	2.806229000000	-4.202640000000	-1.382499000000
C	2.706565000000	-3.108005000000	-0.517129000000
C	2.030462000000	-4.280542000000	-2.562778000000
H	3.274184000000	-3.061005000000	0.420404000000
H	2.133373000000	-5.156869000000	-3.220654000000
C	1.817033000000	-2.065609000000	-0.857574000000
C	1.118943000000	-3.271393000000	-2.907094000000
H	0.495177000000	-3.325116000000	-3.810082000000
C	1.029713000000	-2.185273000000	-2.033385000000
H	3.494112000000	-5.024743000000	-1.135588000000
C	0.443337000000	-0.247799000000	-1.141553000000
C	1.395469000000	-0.813809000000	-0.241616000000
O	0.180249000000	-1.124007000000	-2.184295000000
C	-0.334299000000	0.947555000000	-1.093287000000
H	-1.097319000000	0.926760000000	-1.891840000000
C	0.224860000000	2.317723000000	-0.911851000000
C	1.142976000000	2.675247000000	0.104059000000
C	-0.237154000000	3.326701000000	-1.787601000000
H	1.465517000000	1.906794000000	0.824288000000
H	-0.978045000000	3.069538000000	-2.560220000000
C	1.612102000000	3.991957000000	0.202284000000
C	0.231206000000	4.643553000000	-1.682402000000
H	2.327639000000	4.248210000000	0.998662000000
H	-0.142971000000	5.411140000000	-2.376646000000
C	1.163485000000	4.980883000000	-0.689930000000
H	1.528414000000	6.015426000000	-0.600731000000
N	1.537846000000	-0.242340000000	0.978637000000
S	2.789357000000	-0.604558000000	2.032111000000
O	2.782460000000	-2.029627000000	2.460596000000
O	2.719638000000	0.454941000000	3.060454000000
C	4.286358000000	-0.353496000000	1.047373000000
C	5.351104000000	-1.247535000000	1.223964000000
C	4.407672000000	0.748268000000	0.185564000000
H	5.228696000000	-2.098756000000	1.909811000000
H	3.570750000000	1.449717000000	0.056813000000
C	6.546101000000	-1.037806000000	0.521944000000
C	5.604499000000	0.937038000000	-0.514669000000
H	7.385194000000	-1.737742000000	0.660765000000
H	5.698327000000	1.796183000000	-1.197753000000
C	6.692980000000	0.050333000000	-0.361519000000
C	7.965753000000	0.256572000000	-1.142095000000
H	7.830770000000	-0.035118000000	-2.205753000000
H	8.276518000000	1.320775000000	-1.138233000000
H	8.798661000000	-0.348427000000	-0.734901000000

A*si-SS*

Electronic energy = -2219.012903 Hartree

C	-1.517747000000	-0.485562000000	1.518472000000
O	-1.115317000000	-1.001062000000	2.566498000000
C	-2.870850000000	-0.655003000000	1.059959000000
C	-0.611114000000	0.389254000000	0.627689000000
H	0.611612000000	-1.419367000000	2.315673000000
H	0.329994000000	0.513272000000	1.196479000000

C	-1.254274000000	1.758875000000	0.478846000000
C	-1.017318000000	2.727267000000	1.474603000000
C	-2.122905000000	2.081868000000	-0.582617000000
H	-0.344549000000	2.483133000000	2.311101000000
H	-2.318450000000	1.346777000000	-1.375849000000
C	-1.624066000000	3.989997000000	1.410440000000
C	-2.728585000000	3.344435000000	-0.650312000000
H	-1.423782000000	4.733223000000	2.197114000000
H	-3.394356000000	3.584247000000	-1.493583000000
C	-2.482533000000	4.303154000000	0.345488000000
H	-2.957630000000	5.294199000000	0.289375000000
C	-4.021195000000	-0.766035000000	0.631113000000
C	-5.344629000000	-0.873838000000	0.124337000000
C	-5.795023000000	0.026027000000	-0.877086000000
C	-6.228842000000	-1.871479000000	0.610228000000
H	-5.108698000000	0.803732000000	-1.242456000000
H	-5.877505000000	-2.565398000000	1.387214000000
C	-7.097243000000	-0.075311000000	-1.376249000000
C	-7.527246000000	-1.965463000000	0.098973000000
H	-7.441576000000	0.627123000000	-2.149967000000
H	-8.207268000000	-2.743110000000	0.477610000000
C	-7.964238000000	-1.070302000000	-0.892470000000
H	-8.987509000000	-1.147826000000	-1.289911000000
C	3.060555000000	-5.412764000000	-0.542390000000
C	2.745026000000	-4.373334000000	0.340691000000
C	2.537723000000	-5.452035000000	-1.857120000000
H	3.167178000000	-4.320478000000	1.352720000000
H	2.813132000000	-6.282536000000	-2.524507000000
C	1.883219000000	-3.356390000000	-0.117308000000
C	1.674724000000	-4.451708000000	-2.328905000000
H	1.255562000000	-4.465809000000	-3.344624000000
C	1.370304000000	-3.424389000000	-1.432152000000
H	3.734342000000	-6.216760000000	-0.210148000000
C	0.511080000000	-1.587307000000	-0.530552000000
C	1.311186000000	-2.148872000000	0.459517000000
O	0.532084000000	-2.366843000000	-1.668928000000
C	-0.313046000000	-0.337815000000	-0.706442000000
H	-1.285441000000	-0.666117000000	-1.137844000000
C	0.352094000000	0.556699000000	-1.753268000000
C	1.396825000000	1.431352000000	-1.405989000000
C	-0.053154000000	0.478000000000	-3.098344000000
H	1.732466000000	1.499303000000	-0.362305000000
H	-0.861036000000	-0.216580000000	-3.377753000000
C	2.015194000000	2.221368000000	-2.384983000000
C	0.565879000000	1.266191000000	-4.079916000000
H	2.830141000000	2.902432000000	-2.097669000000
H	0.236731000000	1.193932000000	-5.127761000000
C	1.601548000000	2.143770000000	-3.724472000000
H	2.087689000000	2.766069000000	-4.491057000000
N	1.453746000000	-1.740447000000	1.793806000000
S	2.899830000000	-1.240689000000	2.509869000000
O	3.983082000000	-2.122292000000	2.030549000000
O	2.562567000000	-1.081902000000	3.936449000000
C	3.245804000000	0.400251000000	1.854123000000
C	4.167982000000	0.554545000000	0.810897000000
C	2.583214000000	1.509372000000	2.407925000000
H	4.684264000000	-0.330536000000	0.412936000000
H	1.895727000000	1.360574000000	3.254351000000
C	4.421579000000	1.841646000000	0.313546000000
C	2.837778000000	2.783310000000	1.888521000000
H	5.147613000000	1.970030000000	-0.504337000000
H	2.317551000000	3.656282000000	2.313344000000
C	3.758609000000	2.971968000000	0.832545000000
C	4.000169000000	4.349509000000	0.271773000000
H	3.082846000000	4.742834000000	-0.215521000000
H	4.273351000000	5.070183000000	1.069643000000
H	4.810625000000	4.348629000000	-0.482017000000

RCWC*re-SR*

Electronic energy = -2218.991121 Hartree

C	1.024341000000	-1.135457000000	-0.504965000000
O	-0.052903000000	-0.300261000000	-0.636600000000
C	2.075717000000	-0.546239000000	0.244644000000
C	1.018191000000	-2.419101000000	-1.003266000000
H	-0.782050000000	-0.793323000000	-1.070488000000
H	0.084822000000	-2.720680000000	-1.507665000000
C	2.003138000000	-3.487133000000	-0.899317000000
C	1.600382000000	-4.782016000000	-1.325526000000

C	3.315922000000	-3.347843000000	-0.375187000000
H	0.581978000000	-4.914865000000	-1.722343000000
H	3.670951000000	-2.358607000000	-0.061140000000
C	2.452153000000	-5.886393000000	-1.205898000000
C	4.161848000000	-4.455832000000	-0.256938000000
H	2.099073000000	-6.879033000000	-1.525139000000
H	5.173973000000	-4.319721000000	0.155647000000
C	3.736768000000	-5.734024000000	-0.661309000000
H	4.405511000000	-6.601959000000	-0.557808000000
C	2.934862000000	-0.033725000000	0.964264000000
C	3.913565000000	0.500664000000	1.844298000000
C	4.967375000000	-0.318192000000	2.328635000000
C	3.828777000000	1.843608000000	2.294214000000
H	5.028406000000	-1.363289000000	1.991204000000
H	3.011366000000	2.478118000000	1.922759000000
C	5.898853000000	0.193319000000	3.237916000000
C	4.765943000000	2.344306000000	3.204896000000
H	6.709265000000	-0.452935000000	3.608169000000
H	4.687614000000	3.387244000000	3.547972000000
C	5.802213000000	1.523755000000	3.682190000000
H	6.536047000000	1.921169000000	4.399432000000
C	-0.659425000000	-8.655877000000	-0.030300000000
C	-1.040785000000	-7.317611000000	0.113599000000
C	0.500889000000	-9.156183000000	0.604802000000
H	-1.943851000000	-6.934555000000	-0.376916000000
H	0.775947000000	-10.213427000000	0.469931000000
C	-0.232739000000	-6.474658000000	0.905954000000
C	1.309069000000	-8.338918000000	1.408025000000
H	2.212205000000	-8.712403000000	1.909339000000
C	0.913421000000	-7.005125000000	1.541967000000
H	-1.273367000000	-9.330077000000	-0.644760000000
C	0.889857000000	-4.859885000000	2.160937000000
C	-0.268958000000	-5.056657000000	1.262701000000
O	1.575652000000	-6.073361000000	2.275203000000
C	1.437044000000	-3.825874000000	2.878467000000
H	2.347058000000	-4.156753000000	3.408027000000
C	1.099802000000	-2.436120000000	3.114385000000
C	-0.002598000000	-1.743819000000	2.546020000000
C	1.960672000000	-1.715052000000	3.989471000000
H	-0.650309000000	-2.281661000000	1.841875000000
H	2.829620000000	-2.230428000000	4.427400000000
C	-0.226808000000	-0.398162000000	2.848609000000
C	1.728701000000	-0.371309000000	4.291000000000
H	-1.069530000000	0.122484000000	2.369917000000
H	2.420364000000	0.168071000000	4.955095000000
C	0.629710000000	0.293717000000	3.721777000000
H	0.452663000000	1.356561000000	3.946591000000
N	-1.057989000000	-4.062685000000	0.919030000000
S	-2.309281000000	-4.257704000000	-0.188612000000
O	-3.430895000000	-5.010759000000	0.420407000000
O	-1.797608000000	-4.672102000000	-1.526079000000
C	-2.787269000000	-2.529982000000	-0.326072000000
C	-3.208559000000	-1.822902000000	0.809667000000
C	-2.765265000000	-1.923447000000	-1.590367000000
H	-3.244891000000	-2.323053000000	1.788129000000
H	-2.458384000000	-2.517720000000	-2.463805000000
C	-3.558445000000	-0.474045000000	0.677489000000
C	-3.135075000000	-0.573922000000	-1.706238000000
H	-3.872307000000	0.091114000000	1.569113000000
H	-3.116671000000	-0.091444000000	-2.695997000000
C	-3.514328000000	0.178489000000	-0.574380000000
C	-3.827443000000	1.646219000000	-0.692429000000
H	-2.901960000000	2.247205000000	-0.560094000000
H	-4.241653000000	1.898826000000	-1.687900000000
H	-4.547228000000	1.976295000000	0.081680000000

TSWC$_{re\text{-}SR}$

Electronic energy = -2218.944101 Hartree

C	2.999072000000	-1.165925000000	1.251425000000
O	2.873620000000	0.169993000000	1.316329000000
C	4.271690000000	-1.668292000000	1.567445000000
C	1.885778000000	-1.997032000000	0.909434000000
H	1.923268000000	0.414880000000	1.195605000000
H	1.183902000000	-1.508923000000	0.196269000000
C	2.069930000000	-3.448580000000	0.627820000000
C	1.500976000000	-3.994973000000	-0.543241000000
C	2.705438000000	-4.320471000000	1.540763000000
H	0.957322000000	-3.351176000000	-1.250690000000

H	3.116812000000	-3.923692000000	2.480217000000
C	1.584614000000	-5.371129000000	-0.803297000000
C	2.793303000000	-5.692946000000	1.275308000000
H	1.117913000000	-5.772211000000	-1.715183000000
H	3.284335000000	-6.357785000000	2.002843000000
C	2.234138000000	-6.224015000000	0.099812000000
H	2.291425000000	-7.304745000000	-0.100469000000
C	5.383851000000	-2.160915000000	1.790376000000
C	6.619539000000	-2.806753000000	2.035501000000
C	6.723616000000	-4.213517000000	1.849054000000
C	7.761462000000	-2.079011000000	2.466064000000
H	5.839473000000	-4.771714000000	1.507149000000
H	7.680717000000	-0.991784000000	2.608057000000
C	7.936205000000	-4.864160000000	2.093390000000
C	8.968048000000	-2.744021000000	2.703012000000
H	8.009132000000	-5.952236000000	1.945955000000
H	9.848262000000	-2.173073000000	3.034752000000
C	9.060516000000	-4.134981000000	2.519534000000
H	10.013356000000	-4.652434000000	2.707372000000
C	-2.758231000000	-6.236261000000	-0.129480000000
C	-2.460309000000	-4.892326000000	-0.381862000000
C	-2.299670000000	-6.882954000000	1.041359000000
H	-2.791775000000	-4.404310000000	-1.304971000000
H	-2.546758000000	-7.942634000000	1.206841000000
C	-1.690687000000	-4.184828000000	0.565644000000
C	-1.524360000000	-6.204406000000	1.992992000000
H	-1.145689000000	-6.692605000000	2.901501000000
C	-1.233044000000	-4.865875000000	1.722333000000
H	-3.354230000000	-6.803067000000	-0.859917000000
C	-0.427784000000	-2.812594000000	1.910005000000
C	-1.151841000000	-2.828931000000	0.671989000000
O	-0.469420000000	-4.056088000000	2.516430000000
C	0.575216000000	-1.903021000000	2.378081000000
H	1.131682000000	-2.371614000000	3.209712000000
C	0.363711000000	-0.446748000000	2.637631000000
C	-0.229716000000	0.450512000000	1.712345000000
C	0.849654000000	0.073188000000	3.860363000000
H	-0.606746000000	0.049769000000	0.756060000000
H	1.332331000000	-0.607302000000	4.579209000000

C	-0.336118000000	1.816043000000	2.023298000000
C	0.728590000000	1.434625000000	4.167581000000
H	-0.795855000000	2.496942000000	1.290627000000
H	1.111639000000	1.812303000000	5.127714000000
C	0.136528000000	2.314532000000	3.248049000000
H	0.051377000000	3.386396000000	3.482159000000
N	-1.107514000000	-1.747315000000	-0.114977000000
S	-1.580994000000	-1.704039000000	-1.706827000000
O	-1.166581000000	-2.924915000000	-2.460508000000
O	-1.138824000000	-0.377952000000	-2.189185000000
C	-3.384614000000	-1.694281000000	-1.660846000000
C	-4.093817000000	-2.418962000000	-2.628838000000
C	-4.058447000000	-0.910510000000	-0.710872000000
H	-3.537970000000	-3.017607000000	-3.365791000000
H	-3.486068000000	-0.343515000000	0.038175000000
C	-5.494398000000	-2.365039000000	-2.634160000000
C	-5.457419000000	-0.874906000000	-0.725364000000
H	-6.054614000000	-2.931053000000	-3.395264000000
H	-5.990007000000	-0.266741000000	0.023033000000
C	-6.200050000000	-1.600096000000	-1.683142000000
C	-7.707021000000	-1.574644000000	-1.667374000000
H	-8.107051000000	-2.269852000000	-0.898045000000
H	-8.094979000000	-0.565681000000	-1.422510000000
H	-8.132070000000	-1.882351000000	-2.642421000000

A*$_{re\text{-}SR}$*

Electronic energy = -2218.94729 Hartree

C	1.743795000000	-1.233636000000	1.376096000000
O	1.203527000000	-0.018757000000	1.510364000000
C	3.126193000000	-1.296651000000	1.228393000000
C	0.893443000000	-2.440089000000	1.369555000000
H	0.225510000000	-0.109732000000	1.663272000000
H	0.095325000000	-2.322399000000	0.584426000000
C	1.636521000000	-3.733770000000	1.100641000000
C	1.344368000000	-4.484923000000	-0.053784000000
C	2.581396000000	-4.234474000000	2.019968000000
H	0.574087000000	-4.141400000000	-0.761565000000
H	2.814584000000	-3.666351000000	2.933873000000
C	1.996125000000	-5.705689000000	-0.289454000000

C	3.235203000000	-5.451222000000	1.781596000000
H	1.741939000000	-6.284153000000	-1.190253000000
H	3.968393000000	-5.829022000000	2.510769000000
C	2.945547000000	-6.190366000000	0.621746000000
H	3.450816000000	-7.150954000000	0.438155000000
C	4.345561000000	-1.447329000000	1.053713000000
C	5.715212000000	-1.712016000000	0.844420000000
C	6.145187000000	-3.057872000000	0.656033000000
C	6.678954000000	-0.665350000000	0.815628000000
H	5.395761000000	-3.863112000000	0.676336000000
H	6.346163000000	0.372401000000	0.960808000000
C	7.498387000000	-3.335386000000	0.449311000000
C	8.027779000000	-0.961550000000	0.604532000000
H	7.823207000000	-4.376698000000	0.303984000000
H	8.767227000000	-0.146933000000	0.582167000000
C	8.443050000000	-2.293167000000	0.421917000000
H	9.507174000000	-2.519206000000	0.256355000000
C	-2.514930000000	-7.927544000000	1.282867000000
C	-2.507851000000	-6.617651000000	0.786259000000
C	-1.875757000000	-8.258414000000	2.499445000000
H	-3.020220000000	-6.350282000000	-0.147881000000
H	-1.901981000000	-9.297885000000	2.860070000000
C	-1.833646000000	-5.627951000000	1.531292000000
C	-1.205136000000	-7.287138000000	3.260078000000
H	-0.701468000000	-7.525858000000	4.207089000000
C	-1.197123000000	-5.990614000000	2.744985000000
H	-3.033824000000	-8.715323000000	0.716716000000
C	-0.823313000000	-3.828183000000	2.513067000000
C	-1.579026000000	-4.197064000000	1.372041000000
O	-0.582015000000	-4.908236000000	3.327924000000
C	-0.025452000000	-2.602527000000	2.718400000000
H	0.665735000000	-2.801092000000	3.561118000000
C	-0.810299000000	-1.338502000000	3.041533000000
C	-1.716532000000	-0.758448000000	2.119975000000
C	-0.601481000000	-0.694912000000	4.279459000000
H	-1.918229000000	-1.284336000000	1.171431000000
H	0.107574000000	-1.130645000000	5.000820000000
C	-2.384180000000	0.436374000000	2.438920000000
C	-1.277336000000	0.492211000000	4.595869000000
H	-3.084197000000	0.869252000000	1.707645000000
H	-1.098713000000	0.978277000000	5.567057000000
C	-2.167772000000	1.066442000000	3.674090000000
H	-2.690673000000	2.003010000000	3.919689000000
N	-1.815591000000	-3.280536000000	0.407165000000
S	-2.439643000000	-3.686993000000	-1.073931000000
O	-3.788552000000	-4.307297000000	-0.969390000000
O	-1.417265000000	-4.362005000000	-1.924967000000
C	-2.656525000000	-2.009458000000	-1.690452000000
C	-3.737463000000	-1.248132000000	-1.224972000000
C	-1.735406000000	-1.480011000000	-2.603186000000
H	-4.459342000000	-1.698498000000	-0.528078000000
H	-0.912491000000	-2.115083000000	-2.962685000000
C	-3.877014000000	0.074396000000	-1.667312000000
C	-1.892480000000	-0.157442000000	-3.038412000000
H	-4.724934000000	0.679163000000	-1.308040000000
H	-1.171640000000	0.264458000000	-3.756914000000
C	-2.958010000000	0.643621000000	-2.574703000000
C	-3.104769000000	2.068419000000	-3.045455000000
H	-2.238544000000	2.686518000000	-2.728404000000
H	-3.148062000000	2.124111000000	-4.152844000000
H	-4.022155000000	2.540629000000	-2.644611000000

RCWC*re-SS*

Electronic energy = -2218.998319 Hartree

C	1.989650000000	2.292432000000	1.252428000000
O	0.980589000000	1.978692000000	2.103557000000
C	2.239454000000	1.425038000000	0.148825000000
C	2.792106000000	3.396979000000	1.440482000000
H	0.313524000000	1.392983000000	1.645361000000
H	3.577520000000	3.530672000000	0.682565000000
C	2.714519000000	4.418532000000	2.471157000000
C	1.849453000000	4.357272000000	3.597548000000
C	3.540925000000	5.568133000000	2.337623000000
H	1.208316000000	3.477152000000	3.728958000000
H	4.225235000000	5.629376000000	1.476164000000
C	1.810585000000	5.405111000000	4.525328000000
C	3.490393000000	6.615093000000	3.263470000000
H	1.129643000000	5.331794000000	5.387630000000

H	4.138586000000	7.495250000000	3.130334000000
C	2.620455000000	6.541549000000	4.365564000000
H	2.581184000000	7.362313000000	5.097880000000
C	2.487787000000	0.677421000000	-0.800079000000
C	2.773697000000	-0.181443000000	-1.896722000000
C	2.598630000000	-1.585973000000	-1.783639000000
C	3.214019000000	0.351850000000	-3.136624000000
H	2.266851000000	-2.005917000000	-0.822868000000
H	3.353538000000	1.438605000000	-3.225354000000
C	2.840308000000	-2.421351000000	-2.880740000000
C	3.453951000000	-0.492960000000	-4.226508000000
H	2.698044000000	-3.507874000000	-2.777894000000
H	3.794931000000	-0.064438000000	-5.181625000000
C	3.264766000000	-1.881064000000	-4.107137000000
H	3.453909000000	-2.541547000000	-4.966718000000
C	-3.061303000000	3.529698000000	4.008963000000
C	-2.572914000000	3.174033000000	2.748456000000
C	-2.682816000000	4.747718000000	4.621829000000
H	-2.878678000000	2.237345000000	2.265908000000
H	-3.084896000000	4.997808000000	5.615513000000
C	-1.682550000000	4.060674000000	2.103781000000
C	-1.806814000000	5.645728000000	3.996304000000
H	-1.497463000000	6.591817000000	4.460259000000
C	-1.323112000000	5.273150000000	2.738751000000
H	-3.752972000000	2.853716000000	4.532039000000
C	-0.161045000000	5.288093000000	0.838106000000
C	-0.967103000000	4.050065000000	0.831770000000
O	-0.455497000000	6.008984000000	1.996616000000
C	0.841923000000	5.818748000000	0.067133000000
H	1.245830000000	6.737057000000	0.526402000000
C	1.525206000000	5.420766000000	-1.148277000000
C	1.160335000000	4.336882000000	-1.990888000000
C	2.669127000000	6.189907000000	-1.507330000000
H	0.288979000000	3.727602000000	-1.720578000000
H	2.965423000000	7.034327000000	-0.865527000000
C	1.910031000000	4.043078000000	-3.134310000000
C	3.421673000000	5.881795000000	-2.643157000000
H	1.598117000000	3.202882000000	-3.773939000000
H	4.306358000000	6.485857000000	-2.894867000000
C	3.043176000000	4.805199000000	-3.465432000000
H	3.630270000000	4.566404000000	-4.365610000000
N	-0.925123000000	3.214321000000	-0.182974000000
S	-1.719761000000	1.741408000000	-0.187347000000
O	-3.189930000000	1.889468000000	-0.077025000000
O	-1.036239000000	0.797902000000	0.762594000000
C	-1.314345000000	1.232173000000	-1.853107000000
C	-0.582937000000	0.056296000000	-2.055120000000
C	-1.738209000000	2.025506000000	-2.930256000000
H	-0.259630000000	-0.535580000000	-1.187880000000
H	-2.321973000000	2.939635000000	-2.749894000000
C	-0.252053000000	-0.318110000000	-3.363174000000
C	-1.383975000000	1.642395000000	-4.229682000000
H	0.343140000000	-1.229488000000	-3.526198000000
H	-1.695318000000	2.268320000000	-5.080568000000
C	-0.631930000000	0.471395000000	-4.467747000000
C	-0.229346000000	0.065993000000	-5.861930000000
H	-0.706559000000	-0.893407000000	-6.152484000000
H	-0.514573000000	0.827960000000	-6.612330000000
H	0.867436000000	-0.092444000000	-5.923125000000

TSWC*re-SS*

Electronic energy = -2218.963632 Hartree

C	1.298268000000	2.187900000000	1.067425000000
O	0.310138000000	1.858973000000	1.859124000000
C	1.680442000000	1.380720000000	-0.027138000000
C	1.979175000000	3.441787000000	1.280648000000
H	-0.379434000000	1.170307000000	1.406230000000
H	2.915328000000	3.502000000000	0.704644000000
C	2.078993000000	4.091542000000	2.614555000000
C	1.058800000000	4.057192000000	3.593785000000
C	3.248049000000	4.840096000000	2.890344000000
H	0.141969000000	3.487849000000	3.403360000000
H	4.050958000000	4.877619000000	2.136239000000
C	1.216831000000	4.742853000000	4.805709000000
C	3.402436000000	5.523023000000	4.103051000000
H	0.410184000000	4.703457000000	5.553705000000
H	4.324430000000	6.092337000000	4.296185000000
C	2.383733000000	5.477370000000	5.068326000000

H	2.499789000000	6.013895000000	6.022149000000
C	2.123551000000	0.707884000000	-0.964512000000
C	2.650788000000	-0.032257000000	-2.052219000000
C	2.568106000000	-1.449358000000	-2.081056000000
C	3.281751000000	0.644828000000	-3.130423000000
H	2.081139000000	-1.972477000000	-1.246518000000
H	3.339269000000	1.742201000000	-3.109742000000
C	3.108832000000	-2.163598000000	-3.154530000000
C	3.806088000000	-0.081816000000	-4.204126000000
H	3.046906000000	-3.262331000000	-3.163188000000
H	4.288225000000	0.452617000000	-5.036708000000
C	3.726501000000	-1.485408000000	-4.220154000000
H	4.150129000000	-2.052708000000	-5.062714000000
C	-4.506583000000	3.983648000000	2.652530000000
C	-3.760533000000	3.385595000000	1.631101000000
C	-4.032257000000	5.124526000000	3.342949000000
H	-4.123318000000	2.500894000000	1.089252000000
H	-4.645481000000	5.566336000000	4.143058000000
C	-2.506435000000	3.949146000000	1.305189000000
C	-2.794691000000	5.704951000000	3.030317000000
H	-2.407559000000	6.589595000000	3.554708000000
C	-2.058206000000	5.092870000000	2.011983000000
H	-5.484423000000	3.559192000000	2.924470000000
C	-0.443760000000	4.624742000000	0.563277000000
C	-1.452769000000	3.642705000000	0.342389000000
O	-0.834590000000	5.499273000000	1.572849000000
C	0.938300000000	4.737797000000	0.188050000000
H	1.367682000000	5.612862000000	0.705665000000
C	1.506764000000	4.613668000000	-1.183802000000
C	0.940929000000	3.835607000000	-2.216809000000
C	2.703184000000	5.322328000000	-1.454857000000
H	0.026922000000	3.264596000000	-1.999160000000
H	3.155573000000	5.937286000000	-0.659670000000
C	1.547173000000	3.783056000000	-3.480023000000
C	3.311351000000	5.261858000000	-2.714417000000
H	1.087461000000	3.168755000000	-4.269695000000
H	4.236744000000	5.827080000000	-2.902900000000
C	2.732129000000	4.491030000000	-3.736931000000
H	3.203677000000	4.446616000000	-4.730782000000
N	-1.314788000000	2.669773000000	-0.595854000000
S	-2.011799000000	1.217099000000	-0.450499000000
O	-3.492115000000	1.143425000000	-0.528370000000
O	-1.425251000000	0.479014000000	0.762309000000
C	-1.349513000000	0.395276000000	-1.896256000000
C	-0.997665000000	-0.956688000000	-1.789138000000
C	-1.291458000000	1.061567000000	-3.129660000000
H	-1.055860000000	-1.452911000000	-0.809756000000
H	-1.594856000000	2.115837000000	-3.198393000000
C	-0.562938000000	-1.639407000000	-2.932323000000
C	-0.834666000000	0.367622000000	-4.256404000000
H	-0.286483000000	-2.702238000000	-2.853178000000
H	-0.774123000000	0.891097000000	-5.223800000000
C	-0.457738000000	-0.990380000000	-4.179585000000
C	0.081322000000	-1.713499000000	-5.385038000000
H	-0.134635000000	-2.799127000000	-5.341872000000
H	-0.337460000000	-1.310299000000	-6.327934000000
H	1.186114000000	-1.601304000000	-5.435407000000

$A_{re\text{-}SS}$

Electronic energy = -2218.982677 Hartree

C	1.532369000000	2.224721000000	1.081340000000
O	0.800215000000	1.680354000000	1.931610000000
C	1.971013000000	1.518637000000	-0.082380000000
C	1.999567000000	3.663791000000	1.239196000000
H	-0.289383000000	0.701112000000	1.386570000000
H	3.022124000000	3.709636000000	0.816276000000
C	2.064928000000	4.142977000000	2.674932000000
C	0.964886000000	4.068718000000	3.555336000000
C	3.259048000000	4.734229000000	3.134320000000
H	0.031572000000	3.598385000000	3.223004000000
H	4.126450000000	4.796484000000	2.456730000000
C	1.065530000000	4.577513000000	4.857990000000
C	3.359639000000	5.243000000000	4.437560000000
H	0.196956000000	4.511871000000	5.530793000000
H	4.302358000000	5.699266000000	4.776118000000
C	2.259025000000	5.166371000000	5.304323000000
H	2.331948000000	5.564266000000	6.328021000000
C	2.358895000000	0.883235000000	-1.065452000000

C	2.794674000000	0.157914000000	-2.204564000000
C	2.723907000000	-1.258682000000	-2.225629000000
C	3.289874000000	0.847781000000	-3.341695000000
H	2.324504000000	-1.787490000000	-1.348902000000
H	3.320931000000	1.946677000000	-3.324400000000
C	3.154242000000	-1.964047000000	-3.353757000000
C	3.705733000000	0.130111000000	-4.467607000000
H	3.100136000000	-3.063027000000	-3.362664000000
H	4.084293000000	0.670347000000	-5.348507000000
C	3.645199000000	-1.274562000000	-4.475700000000
H	3.979301000000	-1.834868000000	-5.362127000000
C	-4.596868000000	4.227975000000	2.347652000000
C	-3.740247000000	3.493181000000	1.519420000000
C	-4.199028000000	5.459346000000	2.920983000000
H	-4.043999000000	2.534036000000	1.077158000000
H	-4.899681000000	6.006409000000	3.569731000000
C	-2.451729000000	4.013123000000	1.266427000000
C	-2.926120000000	5.995806000000	2.680576000000
H	-2.595221000000	6.948640000000	3.116932000000
C	-2.080157000000	5.250705000000	1.854169000000
H	-5.603941000000	3.838473000000	2.559708000000
C	-0.323974000000	4.577606000000	0.695340000000
C	-1.284987000000	3.588934000000	0.503368000000
O	-0.805542000000	5.593292000000	1.501829000000
C	1.121578000000	4.656716000000	0.337371000000
H	1.453476000000	5.659619000000	0.680375000000
C	1.453556000000	4.574299000000	-1.154842000000
C	0.448633000000	4.525322000000	-2.140004000000
C	2.796752000000	4.634035000000	-1.582896000000
H	-0.604225000000	4.502484000000	-1.829648000000
H	3.608248000000	4.707273000000	-0.842203000000
C	0.775595000000	4.502922000000	-3.505406000000
C	3.127909000000	4.617949000000	-2.945268000000
H	-0.030692000000	4.460663000000	-4.253878000000
H	4.184635000000	4.674014000000	-3.248940000000
C	2.115941000000	4.541632000000	-3.916370000000
H	2.371437000000	4.527570000000	-4.986876000000
N	-1.056480000000	2.510190000000	-0.342705000000
S	-1.717165000000	1.102429000000	-0.235446000000
O	-3.180249000000	0.877178000000	-0.203961000000
O	-1.140056000000	0.245731000000	1.030919000000
C	-1.055164000000	0.247165000000	-1.655930000000
C	-1.204486000000	-1.148807000000	-1.695821000000
C	-0.520645000000	0.950769000000	-2.743252000000
H	-1.619681000000	-1.683046000000	-0.829230000000
H	-0.388780000000	2.038310000000	-2.671463000000
C	-0.806807000000	-1.839226000000	-2.846119000000
C	-0.138463000000	0.240481000000	-3.888225000000
H	-0.911532000000	-2.935127000000	-2.878894000000
H	0.294908000000	0.790791000000	-4.737675000000
C	-0.279314000000	-1.159547000000	-3.966008000000
C	0.099704000000	-1.907470000000	-5.217302000000
H	0.433538000000	-2.939121000000	-4.991038000000
H	-0.768753000000	-1.986755000000	-5.906371000000
H	0.913137000000	-1.393653000000	-5.765113000000

Catalyst VI

Electronic energy = -2015.128212 Hartree

C	0.700165000000	0.234536000000	-0.143505000000
O	0.900467000000	1.217195000000	-0.854327000000
N	1.717455000000	-0.591252000000	0.353136000000
N	-0.551457000000	-0.170344000000	0.273755000000
H	-0.748439000000	-1.154390000000	0.527818000000
H	1.434422000000	-1.321469000000	1.007989000000
C	3.088841000000	-0.510556000000	0.105936000000
C	3.924472000000	-1.480523000000	0.707157000000
C	3.672654000000	0.479212000000	-0.714341000000
H	3.489211000000	-2.265375000000	1.344062000000
H	3.025929000000	1.235125000000	-1.176071000000
C	5.306267000000	-1.460968000000	0.494199000000
C	5.060686000000	0.473793000000	-0.922267000000
C	5.892019000000	-0.485105000000	-0.329197000000
H	6.974529000000	-0.475424000000	-0.507678000000
C	5.641488000000	1.531432000000	-1.835796000000
F	5.329014000000	2.778236000000	-1.409959000000
F	6.990752000000	1.453643000000	-1.914007000000
F	5.154964000000	1.410860000000	-3.095447000000
C	6.187636000000	-2.464858000000	1.200586000000

F	5.504125000000	-3.584421000000	1.545530000000
F	7.232265000000	-2.843619000000	0.428329000000
F	6.705232000000	-1.948322000000	2.342970000000
C	-1.744016000000	0.459138000000	-0.253452000000
H	-1.410879000000	1.155439000000	-1.049414000000
C	-2.489012000000	1.266615000000	0.801356000000
C	-3.318787000000	2.384327000000	0.445564000000
C	-4.058533000000	3.036280000000	1.504568000000
N	-3.978297000000	2.655623000000	2.814322000000
C	-3.159818000000	1.658822000000	3.109935000000
C	-2.397416000000	0.942841000000	2.150362000000
H	-3.087616000000	1.376819000000	4.176862000000
H	-1.730758000000	0.131144000000	2.475552000000
C	-4.274783000000	3.970731000000	-1.157534000000
C	-3.446211000000	2.893271000000	-0.882227000000
C	-5.021213000000	4.591093000000	-0.120011000000
H	-2.874642000000	2.433124000000	-1.700192000000
H	-5.678090000000	5.442458000000	-0.354299000000
C	-4.911572000000	4.134020000000	1.182911000000
H	-5.461188000000	4.596652000000	2.015279000000
H	-4.349990000000	4.352006000000	-2.187073000000
C	-3.667908000000	-2.049022000000	0.788138000000
C	-5.075468000000	-2.078686000000	0.089047000000
C	-4.907534000000	-1.582201000000	-1.375936000000
C	-4.381756000000	-2.705176000000	-2.314105000000
C	-3.460020000000	-3.739188000000	-1.648599000000
C	-2.578025000000	-0.693035000000	-0.943766000000
C	-4.014034000000	-0.330867000000	-1.366987000000
H	-3.636140000000	-1.189281000000	1.485499000000
H	-5.457845000000	-3.120024000000	0.062296000000
H	-5.907736000000	-1.276807000000	-1.745838000000
H	-5.250165000000	-3.252279000000	-2.736772000000
H	-3.872450000000	-2.237152000000	-3.185330000000
H	-3.094611000000	-4.464650000000	-2.406313000000
H	-1.991188000000	-0.923682000000	-1.855343000000
H	-4.006379000000	0.156582000000	-2.363218000000
H	-4.457028000000	0.411144000000	-0.674873000000
C	-2.252726000000	-3.140617000000	-0.902910000000
H	-1.442131000000	-2.889247000000	-1.618583000000
H	-4.057613000000	-4.342584000000	-0.933258000000
N	-2.530290000000	-1.924541000000	-0.127702000000
C	-6.065673000000	-1.256802000000	0.869034000000
C	-7.179321000000	-1.734317000000	1.451067000000
H	-7.450219000000	-2.801641000000	1.380505000000
H	-7.860066000000	-1.080834000000	2.018269000000
H	-5.828987000000	-0.180776000000	0.977621000000
H	-1.833298000000	-3.909148000000	-0.216169000000
H	-3.526139000000	-2.956362000000	1.409597000000

RC$_{si\text{-}SS}$

Electronic energy = -4234.149601 Hartree

C	-0.728140000000	0.476156000000	1.804187000000
O	0.530456000000	-0.041845000000	1.633965000000
C	-1.520094000000	-0.318355000000	2.676706000000
C	-1.129966000000	1.628370000000	1.172020000000
H	1.104580000000	0.587102000000	1.140760000000
H	-0.385064000000	2.066526000000	0.487547000000
C	-2.371104000000	2.381972000000	1.282622000000
C	-2.582246000000	3.444160000000	0.364328000000
C	-3.361621000000	2.162842000000	2.274733000000
H	-1.817960000000	3.642314000000	-0.401570000000
H	-3.215714000000	1.378200000000	3.026109000000
C	-3.729375000000	4.242137000000	0.426985000000
C	-4.502240000000	2.969520000000	2.340774000000
H	-3.864226000000	5.060396000000	-0.297326000000
H	-5.236567000000	2.792175000000	3.140842000000
C	-4.698427000000	4.010840000000	1.417137000000
H	-5.596085000000	4.644883000000	1.476400000000
C	-2.176409000000	-1.033045000000	3.436491000000
C	-2.967932000000	-1.853174000000	4.290632000000
C	-4.233909000000	-2.321167000000	3.852243000000
C	-2.494335000000	-2.229092000000	5.574671000000
H	-4.599227000000	-2.023375000000	2.858696000000
H	-1.518049000000	-1.848013000000	5.913693000000
C	-4.997603000000	-3.157659000000	4.673619000000
C	-3.271039000000	-3.067525000000	6.383624000000
H	-5.976391000000	-3.518981000000	4.323318000000

H	-2.897218000000	-3.354563000000	7.378393000000
C	-4.517854000000	-3.538032000000	5.938475000000
H	-5.120547000000	-4.198576000000	6.580160000000
C	4.029018000000	-4.275249000000	-0.049071000000
C	3.941489000000	-2.923642000000	0.290573000000
C	3.278291000000	-4.809970000000	-1.122511000000
H	4.495952000000	-2.521557000000	1.143801000000
H	3.360471000000	-5.882178000000	-1.356662000000
C	3.089221000000	-2.089171000000	-0.470952000000
C	2.427396000000	-4.007060000000	-1.894514000000
H	1.836095000000	-4.406423000000	-2.729394000000
C	2.357015000000	-2.655096000000	-1.547716000000
H	4.679797000000	-4.937400000000	0.539789000000
C	1.781527000000	-0.504471000000	-1.566835000000
C	2.714103000000	-0.681598000000	-0.438708000000
O	1.586142000000	-1.738967000000	-2.187963000000
C	1.083750000000	0.540456000000	-2.129193000000
H	0.461704000000	0.181260000000	-2.967382000000
C	0.974973000000	1.968002000000	-1.904768000000
C	1.693439000000	2.729161000000	-0.938598000000
C	0.046365000000	2.659370000000	-2.736726000000
H	2.399351000000	2.215864000000	-0.273536000000
H	-0.519818000000	2.090561000000	-3.490386000000
C	1.477891000000	4.104484000000	-0.812591000000
C	-0.165292000000	4.032999000000	-2.602383000000
H	2.019974000000	4.664165000000	-0.037926000000
H	-0.897832000000	4.538663000000	-3.248962000000
C	0.547784000000	4.762587000000	-1.634533000000
H	0.367142000000	5.840328000000	-1.507794000000
N	3.007872000000	0.353070000000	0.332051000000
S	3.948138000000	0.332803000000	1.710280000000
O	3.852652000000	-0.928954000000	2.503510000000
O	3.583371000000	1.588155000000	2.412982000000
C	5.621666000000	0.470178000000	1.096652000000
C	6.600785000000	-0.406778000000	1.582267000000
C	5.938978000000	1.488326000000	0.180369000000
H	6.327860000000	-1.180564000000	2.314111000000
H	5.158516000000	2.171938000000	-0.184285000000
C	7.920122000000	-0.264678000000	1.129745000000
C	7.259208000000	1.607830000000	-0.261458000000
H	8.695759000000	-0.948260000000	1.508043000000
H	7.516161000000	2.398903000000	-0.983139000000
C	8.272070000000	0.737328000000	0.203685000000
C	9.689393000000	0.891731000000	-0.281277000000
H	10.093153000000	1.890854000000	-0.015554000000
H	10.358936000000	0.126656000000	0.155095000000
H	9.745582000000	0.808112000000	-1.386382000000
H	2.452581000000	-1.448067000000	3.799986000000
N	1.975242000000	-1.578357000000	4.700944000000
C	1.164228000000	-0.573245000000	5.168342000000
O	0.303380000000	-0.746626000000	6.041913000000
N	1.417286000000	0.671123000000	4.584497000000
H	2.235583000000	0.751611000000	3.971481000000
C	1.745383000000	-2.939551000000	5.159085000000
C	3.036792000000	-3.706762000000	5.350759000000
C	4.106370000000	-3.523810000000	4.477234000000
H	4.063329000000	-2.756278000000	3.695409000000
C	5.246121000000	-4.361335000000	4.569928000000
H	6.079693000000	-4.216494000000	3.855247000000
N	5.387890000000	-5.341662000000	5.448460000000
C	4.381432000000	-5.516708000000	6.356260000000
C	3.182238000000	-4.706674000000	6.372974000000
C	2.222467000000	-4.943101000000	7.403130000000
H	1.322427000000	-4.315175000000	7.462716000000
C	2.411604000000	-5.947138000000	8.340969000000
H	1.660068000000	-6.106269000000	9.129055000000
C	3.571198000000	-6.766609000000	8.297193000000
H	3.704960000000	-7.564356000000	9.043684000000
C	4.537018000000	-6.549597000000	7.328498000000
H	5.457068000000	-7.149274000000	7.272876000000
H	1.218454000000	-2.831897000000	6.126370000000
C	0.561804000000	1.772739000000	4.563386000000
C	-0.676672000000	1.811189000000	5.240001000000
H	-0.966142000000	0.958549000000	5.863846000000
C	-1.518654000000	2.923625000000	5.090194000000
C	-1.155253000000	4.022595000000	4.302908000000
C	0.092500000000	3.990579000000	3.656400000000
C	0.944313000000	2.888747000000	3.779326000000

H	1.904760000000	2.869492000000	3.246407000000
C	0.477167000000	5.147974000000	2.771329000000
F	1.788595000000	5.113512000000	2.409337000000
F	0.257574000000	6.341120000000	3.371910000000
F	-0.244031000000	5.160858000000	1.613948000000
C	-2.846058000000	2.900119000000	5.810779000000
F	-2.687037000000	2.932949000000	7.156261000000
F	-3.545774000000	1.764775000000	5.532555000000
F	-3.636169000000	3.947544000000	5.472293000000
H	-1.833682000000	4.876927000000	4.186116000000
C	0.735234000000	-3.663743000000	4.197250000000
C	0.051407000000	-4.902946000000	4.802725000000
N	1.317814000000	-3.943336000000	2.876433000000
C	0.472984000000	-3.501331000000	1.765950000000
H	0.158794000000	-2.464934000000	1.991443000000
H	1.102612000000	-3.433355000000	0.853807000000
C	-0.751533000000	-4.395233000000	1.466703000000
H	-0.441287000000	-5.214971000000	0.783199000000
H	-1.495044000000	-3.800407000000	0.893744000000
C	-1.433868000000	-5.013220000000	2.700515000000
H	-2.221731000000	-5.705090000000	2.334916000000
H	-1.973286000000	-4.229707000000	3.273693000000
C	1.807195000000	-5.309642000000	2.743648000000
H	2.295725000000	-5.413295000000	1.753373000000
H	2.598647000000	-5.468348000000	3.501706000000
C	0.705505000000	-6.423710000000	2.939113000000
H	0.373965000000	-6.765981000000	1.933691000000
C	-0.497411000000	-5.791146000000	3.671125000000
H	-1.100025000000	-6.602336000000	4.128950000000
C	1.333233000000	-7.617887000000	3.611305000000
H	2.215420000000	-8.020146000000	3.076582000000
C	0.986366000000	-8.203538000000	4.771587000000
H	0.129068000000	-7.862644000000	5.373310000000
H	1.558234000000	-9.056241000000	5.168701000000
H	-0.759978000000	-4.581562000000	5.487750000000
H	0.765713000000	-5.497528000000	5.405442000000
H	-0.046215000000	-2.894355000000	4.039652000000

TS$_{si\text{-}SS}$

Electronic energy = -4234.1235 Hartree

C	-0.615338000000	0.167336000000	2.048897000000
O	0.570288000000	-0.370292000000	2.267631000000
C	-1.677366000000	-0.560821000000	2.626007000000
C	-0.729663000000	1.388236000000	1.326637000000
H	1.377411000000	0.199409000000	1.952193000000
H	0.101499000000	2.071174000000	1.560940000000
C	-2.007158000000	2.034440000000	1.003828000000
C	-2.086335000000	3.446804000000	1.005337000000
C	-3.154786000000	1.294180000000	0.633289000000
H	-1.196777000000	4.035550000000	1.267934000000
H	-3.103207000000	0.196507000000	0.587128000000
C	-3.287370000000	4.096124000000	0.691498000000
C	-4.352956000000	1.946233000000	0.321386000000
H	-3.330978000000	5.195600000000	0.710017000000
H	-5.238767000000	1.354764000000	0.043941000000
C	-4.427154000000	3.349532000000	0.355798000000
H	-5.371868000000	3.859100000000	0.113019000000
C	-2.450017000000	-1.199315000000	3.346507000000
C	-3.268693000000	-1.931836000000	4.241514000000
C	-4.634639000000	-2.190606000000	3.960567000000
C	-2.690823000000	-2.415548000000	5.447953000000
H	-5.076549000000	-1.802391000000	3.031751000000
H	-1.638384000000	-2.175880000000	5.671261000000
C	-5.400076000000	-2.939338000000	4.859938000000
C	-3.475610000000	-3.154860000000	6.338769000000
H	-6.457642000000	-3.144647000000	4.636631000000
H	-3.028837000000	-3.523163000000	7.274588000000
C	-4.823955000000	-3.424816000000	6.047019000000
H	-5.432501000000	-4.011836000000	6.751482000000
C	3.724207000000	-3.864003000000	-0.667107000000
C	3.761429000000	-2.607980000000	-0.055334000000
C	2.655560000000	-4.241615000000	-1.515449000000
H	4.582524000000	-2.338946000000	0.617105000000
H	2.655907000000	-5.241874000000	-1.973862000000
C	2.700988000000	-1.704866000000	-0.302081000000
C	1.592577000000	-3.367875000000	-1.778289000000
H	0.749721000000	-3.642397000000	-2.427075000000
C	1.642689000000	-2.116668000000	-1.157322000000

H	4.539109000000	-4.577882000000	-0.477974000000
C	1.130208000000	-0.047537000000	-0.555100000000
C	2.335458000000	-0.367007000000	0.139551000000
O	0.692703000000	-1.150594000000	-1.280430000000
C	0.263779000000	1.081288000000	-0.601685000000
H	-0.655341000000	0.822872000000	-1.154312000000
C	0.661134000000	2.490327000000	-0.803173000000
C	1.806786000000	3.092055000000	-0.226623000000
C	-0.195293000000	3.293466000000	-1.594455000000
H	2.444053000000	2.494230000000	0.441969000000
H	-1.103090000000	2.845800000000	-2.026620000000
C	2.093362000000	4.440148000000	-0.466767000000
C	0.099656000000	4.642215000000	-1.834988000000
H	2.974240000000	4.895477000000	0.008636000000
H	-0.579947000000	5.245274000000	-2.455642000000
C	1.247045000000	5.220384000000	-1.273822000000
H	1.474080000000	6.282970000000	-1.447799000000
N	2.763453000000	0.470054000000	1.135472000000
S	4.052180000000	0.139140000000	2.106778000000
O	4.054698000000	-1.260127000000	2.647139000000
O	4.062565000000	1.215697000000	3.136713000000
C	5.511897000000	0.303540000000	1.077447000000
C	6.638368000000	-0.471390000000	1.392674000000
C	5.537686000000	1.229302000000	0.023021000000
H	6.591203000000	-1.189578000000	2.224296000000
H	4.646044000000	1.828401000000	-0.212282000000
C	7.803410000000	-0.315737000000	0.631572000000
C	6.710768000000	1.365450000000	-0.728542000000
H	8.691130000000	-0.920066000000	0.875292000000
H	6.734736000000	2.086743000000	-1.560319000000
C	7.860641000000	0.598305000000	-0.441540000000
C	9.104640000000	0.734306000000	-1.280097000000
H	9.279009000000	1.785946000000	-1.582341000000
H	10.002463000000	0.372076000000	-0.743156000000
H	9.015735000000	0.139446000000	-2.214624000000
H	2.889987000000	-1.645245000000	4.189322000000
N	2.233225000000	-1.799284000000	4.968798000000
C	1.333063000000	-0.816718000000	5.283884000000
O	0.280555000000	-1.031558000000	5.902612000000
N	1.726406000000	0.463710000000	4.868835000000
H	2.675209000000	0.577142000000	4.482204000000
C	1.944612000000	-3.167209000000	5.381346000000
C	3.214307000000	-3.964051000000	5.584957000000
C	4.272297000000	-3.846740000000	4.685681000000
H	4.230203000000	-3.136839000000	3.849097000000
C	5.402304000000	-4.694107000000	4.812706000000
H	6.229186000000	-4.597742000000	4.083650000000
N	5.540342000000	-5.627636000000	5.741245000000
C	4.535888000000	-5.750010000000	6.659451000000
C	3.347422000000	-4.924289000000	6.646107000000
C	2.383973000000	-5.114330000000	7.682383000000
H	1.488255000000	-4.478291000000	7.716442000000
C	2.561088000000	-6.085396000000	8.656833000000
H	1.804913000000	-6.209531000000	9.446845000000
C	3.713610000000	-6.915577000000	8.647657000000
H	3.838786000000	-7.685740000000	9.424010000000
C	4.681851000000	-6.744565000000	7.672185000000
H	5.595404000000	-7.355736000000	7.638863000000
H	1.393634000000	-3.071473000000	6.336406000000
C	0.892756000000	1.562556000000	4.681524000000
C	-0.480826000000	1.565745000000	5.017450000000
H	-0.909871000000	0.684260000000	5.505568000000
C	-1.271742000000	2.684343000000	4.717619000000
C	-0.728349000000	3.833480000000	4.128355000000
C	0.642771000000	3.833807000000	3.814748000000
C	1.446923000000	2.719756000000	4.077313000000
H	2.506179000000	2.720563000000	3.785636000000
C	1.250738000000	5.073809000000	3.206318000000
F	2.493671000000	4.855034000000	2.714270000000
F	1.345864000000	6.081969000000	4.105451000000
F	0.489534000000	5.548032000000	2.176442000000
C	-2.760611000000	2.597916000000	4.962878000000
F	-3.053800000000	1.880373000000	6.071825000000
F	-3.391857000000	1.987919000000	3.922489000000
F	-3.325302000000	3.821529000000	5.102899000000
H	-1.361781000000	4.703257000000	3.912436000000
C	0.936421000000	-3.857028000000	4.392318000000
C	0.168560000000	-5.037414000000	5.017478000000

N 1.540125000000 -4.210069000000 3.103512000000

C 0.753319000000 -3.768822000000 1.952740000000

H 0.475981000000 -2.713912000000 2.136938000000

H 1.414568000000 -3.762662000000 1.062742000000

C -0.502777000000 -4.613220000000 1.640247000000

H -0.210861000000 -5.472518000000 0.998840000000

H -1.196301000000 -4.007502000000 1.017215000000

C -1.254722000000 -5.149871000000 2.871726000000

H -2.065849000000 -5.814675000000 2.506465000000

H -1.770294000000 -4.319338000000 3.400341000000

C 1.965077000000 -5.601851000000 3.022517000000

H 2.469470000000 -5.758443000000 2.046353000000

H 2.732335000000 -5.778688000000 3.801105000000

C 0.803046000000 -6.653515000000 3.226155000000

H 0.481667000000 -7.016566000000 2.224788000000

C -0.388058000000 -5.937428000000 3.898836000000

H -1.044149000000 -6.702425000000 4.362797000000

C 1.345444000000 -7.853561000000 3.959744000000

H 2.226076000000 -8.315730000000 3.473075000000

C 0.924529000000 -8.384965000000 5.121825000000

H 0.063196000000 -7.982488000000 5.677944000000

H 1.436438000000 -9.252086000000 5.566628000000

H -0.647759000000 -4.652073000000 5.663382000000

H 0.832607000000 -5.644199000000 5.665333000000

H 0.203798000000 -3.052768000000 4.187352000000

IntA$_{si\text{-}SS}$

Electronic energy = -4234.162974 Hartree

C -0.595727000000 -0.351628000000 2.249423000000

O 0.456434000000 -0.962065000000 2.519490000000

C -1.780903000000 -0.661868000000 2.975971000000

C -0.569671000000 0.818869000000 1.266506000000

H 1.955738000000 -0.230949000000 2.160425000000

H 0.112711000000 1.526886000000 1.784755000000

C -1.907029000000 1.511141000000 1.083527000000

C -2.044854000000 2.885531000000 1.345841000000

C -3.029138000000 0.790569000000 0.626426000000

H -1.171170000000 3.463415000000 1.676788000000

H -2.938312000000 -0.284638000000 0.403893000000

C -3.283759000000 3.523240000000 1.185206000000

C -4.269375000000 1.424692000000 0.468332000000

H -3.374571000000 4.598425000000 1.401604000000

H -5.138578000000 0.846112000000 0.120011000000

C -4.400905000000 2.793799000000 0.753283000000

H -5.374605000000 3.292265000000 0.631837000000

C -2.640629000000 -0.862594000000 3.838691000000

C -3.563803000000 -1.076336000000 4.889290000000

C -4.897393000000 -0.599945000000 4.802354000000

C -3.118464000000 -1.732115000000 6.068937000000

H -5.218015000000 -0.069228000000 3.894735000000

H -2.065972000000 -2.047990000000 6.131451000000

C -5.773783000000 -0.797738000000 5.872468000000

C -4.007085000000 -1.914054000000 7.132895000000

H -6.808688000000 -0.429904000000 5.807765000000

H -3.662474000000 -2.412252000000 8.051335000000

C -5.332167000000 -1.454520000000 7.035212000000

H -6.026269000000 -1.601099000000 7.876819000000

C 4.510719000000 -3.402920000000 -1.522066000000

C 4.269190000000 -2.458651000000 -0.518327000000

C 3.566224000000 -3.662870000000 -2.542470000000

H 4.996307000000 -2.300838000000 0.286313000000

H 3.791232000000 -4.415180000000 -3.312958000000

C 3.047142000000 -1.747401000000 -0.541496000000

C 2.343131000000 -2.980434000000 -2.582389000000

H 1.587059000000 -3.170167000000 -3.356840000000

C 2.119567000000 -2.035995000000 -1.577723000000

H 5.456373000000 -3.965116000000 -1.510115000000

C 1.155112000000 -0.491322000000 -0.317158000000

C 2.390109000000 -0.733883000000 0.262838000000

O 0.982802000000 -1.293428000000 -1.430009000000

C 0.055753000000 0.522749000000 -0.145615000000

H -0.769373000000 0.122608000000 -0.767812000000

C 0.512902000000 1.835717000000 -0.790621000000

C 1.339391000000 2.741652000000 -0.096083000000

C 0.146575000000 2.135533000000 -2.115113000000

H 1.641904000000 2.532208000000 0.940603000000

H -0.500660000000 1.436570000000 -2.667283000000

C 1.782711000000 3.923322000000 -0.707056000000

C	0.593006000000	3.314660000000	-2.731763000000
H	2.406557000000	4.623964000000	-0.132687000000
H	0.289702000000	3.536553000000	-3.766279000000
C	1.411918000000	4.212593000000	-2.030136000000
H	1.752311000000	5.142618000000	-2.510058000000
N	2.745998000000	-0.098015000000	1.474101000000
S	4.222074000000	-0.245614000000	2.258505000000
O	4.648667000000	-1.646778000000	2.514854000000
O	4.074863000000	0.622506000000	3.464102000000
C	5.347211000000	0.498816000000	1.093571000000
C	6.650567000000	-0.012692000000	1.013370000000
C	4.942654000000	1.604031000000	0.327604000000
H	6.936503000000	-0.881824000000	1.623232000000
H	3.915551000000	1.987257000000	0.397668000000
C	7.560683000000	0.597129000000	0.141271000000
C	5.865774000000	2.189810000000	-0.543673000000
H	8.586290000000	0.203061000000	0.071410000000
H	5.549052000000	3.048087000000	-1.156646000000
C	7.186271000000	1.700670000000	-0.653624000000
C	8.156634000000	2.330326000000	-1.617682000000
H	8.106906000000	3.436871000000	-1.576688000000
H	9.199136000000	2.019917000000	-1.413733000000
H	7.917676000000	2.036173000000	-2.661980000000
H	3.046544000000	-1.821286000000	4.610994000000
N	2.247334000000	-2.011512000000	5.221377000000
C	1.303914000000	-1.035766000000	5.436645000000
O	0.199140000000	-1.263649000000	5.941683000000
N	1.731963000000	0.239112000000	5.048228000000
H	2.681239000000	0.316539000000	4.648888000000
C	1.877205000000	-3.406875000000	5.432211000000
C	3.068989000000	-4.229010000000	5.862807000000
C	4.288144000000	-4.089953000000	5.202563000000
H	4.417060000000	-3.367376000000	4.383604000000
C	5.373143000000	-4.938289000000	5.543956000000
H	6.333306000000	-4.823369000000	5.006907000000
N	5.320171000000	-5.889209000000	6.463643000000
C	4.142628000000	-6.045975000000	7.138861000000
C	2.973468000000	-5.231176000000	6.886764000000
C	1.799074000000	-5.478898000000	7.659811000000
H	0.903383000000	-4.862191000000	7.500370000000
C	1.769305000000	-6.483604000000	8.615645000000
H	0.851807000000	-6.654357000000	9.198995000000
C	2.914903000000	-7.289423000000	8.851819000000
H	2.876925000000	-8.083978000000	9.612589000000
C	4.076968000000	-7.069628000000	8.130552000000
H	4.986863000000	-7.666887000000	8.287399000000
H	1.112972000000	-3.376130000000	6.229884000000
C	0.937168000000	1.362190000000	4.856199000000
C	-0.405084000000	1.459267000000	5.293344000000
H	-0.846416000000	0.615315000000	5.834052000000
C	-1.144894000000	2.617377000000	5.019541000000
C	-0.581636000000	3.709517000000	4.342668000000
C	0.752958000000	3.610361000000	3.912874000000
C	1.507457000000	2.458150000000	4.159576000000
H	2.543618000000	2.385187000000	3.799602000000
C	1.377707000000	4.777245000000	3.187910000000
F	2.530215000000	4.430907000000	2.552179000000
F	1.674832000000	5.798233000000	4.022556000000
F	0.539051000000	5.278936000000	2.238369000000
C	-2.611182000000	2.657929000000	5.386510000000
F	-2.910086000000	1.829055000000	6.412241000000
F	-3.388546000000	2.281138000000	4.332965000000
F	-3.008496000000	3.905721000000	5.736002000000
H	-1.174892000000	4.612098000000	4.145733000000
C	1.163251000000	-3.983995000000	4.154018000000
C	0.077840000000	-5.031056000000	4.465400000000
N	2.108425000000	-4.459700000000	3.139419000000
C	1.860742000000	-3.929475000000	1.802886000000
H	1.729002000000	-2.837724000000	1.909155000000
H	2.775213000000	-4.072953000000	1.190630000000
C	0.646453000000	-4.539728000000	1.063663000000
H	0.975495000000	-5.444959000000	0.510470000000
H	0.306280000000	-3.829498000000	0.278932000000
C	-0.543613000000	-4.917004000000	1.967248000000
H	-1.297603000000	-5.428331000000	1.332500000000
H	-1.044296000000	-3.998101000000	2.343357000000
C	2.317780000000	-5.898881000000	3.184994000000
H	3.116605000000	-6.169426000000	2.464121000000

H	2.700977000000	-6.162613000000	4.191558000000
C	1.011376000000	-6.743345000000	2.912814000000
H	1.007018000000	-7.037902000000	1.840332000000
C	-0.210141000000	-5.836519000000	3.183625000000
H	-1.096770000000	-6.478715000000	3.362738000000
C	1.069829000000	-8.023827000000	3.704236000000
H	1.980749000000	-8.624286000000	3.516197000000
C	0.191838000000	-8.488086000000	4.611585000000
H	-0.735977000000	-7.951134000000	4.865325000000
H	0.372840000000	-9.436266000000	5.140927000000
H	-0.834146000000	-4.518144000000	4.836944000000
H	0.403999000000	-5.729471000000	5.263907000000
H	0.653860000000	-3.099855000000	3.727357000000

RC$_{si\text{-}SR}$

Electronic energy = -4234.143979 Hartree

C	-1.668158000000	-0.407831000000	0.810595000000
O	-1.742057000000	-0.413092000000	2.168236000000
C	-1.879979000000	-1.682709000000	0.223691000000
C	-1.442282000000	0.720490000000	0.049516000000
H	-1.640953000000	0.513224000000	2.480258000000
H	-1.543216000000	0.603326000000	-1.039045000000
C	-1.213107000000	2.060234000000	0.579694000000
C	-0.579468000000	2.288844000000	1.832916000000
C	-1.630425000000	3.194783000000	-0.162934000000
H	-0.113209000000	1.454329000000	2.379323000000
H	-2.101237000000	3.041228000000	-1.146339000000
C	-0.427891000000	3.585722000000	2.337546000000
C	-1.466879000000	4.489650000000	0.341047000000
H	0.068213000000	3.738950000000	3.305256000000
H	-1.795587000000	5.352745000000	-0.257325000000
C	-0.877424000000	4.691776000000	1.600779000000
H	-0.739251000000	5.706314000000	2.001770000000
C	-2.038511000000	-2.810117000000	-0.248472000000
C	-2.171702000000	-4.120736000000	-0.779058000000
C	-2.368757000000	-4.332044000000	-2.168850000000
C	-2.079983000000	-5.249751000000	0.077143000000
H	-2.446658000000	-3.460886000000	-2.834600000000
H	-1.938867000000	-5.090782000000	1.155017000000
C	-2.458235000000	-5.632095000000	-2.679616000000
C	-2.163110000000	-6.544656000000	-0.445041000000
H	-2.614165000000	-5.782682000000	-3.758791000000
H	-2.078753000000	-7.407510000000	0.232996000000
C	-2.350179000000	-6.742119000000	-1.824244000000
H	-2.416268000000	-7.761933000000	-2.232056000000
C	1.241186000000	-3.344868000000	-1.302255000000
C	1.559911000000	-2.201837000000	-0.566495000000
C	0.727413000000	-3.246783000000	-2.616319000000
H	1.927454000000	-2.279698000000	0.462333000000
H	0.481613000000	-4.166353000000	-3.166985000000
C	1.369184000000	-0.934489000000	-1.166328000000
C	0.508870000000	-2.003457000000	-3.225802000000
H	0.106694000000	-1.909580000000	-4.243813000000
C	0.837759000000	-0.868451000000	-2.478254000000
H	1.371970000000	-4.336305000000	-0.845121000000
C	1.197251000000	1.263068000000	-1.924432000000
C	1.646810000000	0.448175000000	-0.783680000000
O	0.713146000000	0.411200000000	-2.924172000000
C	1.118480000000	2.606218000000	-2.209387000000
H	0.676156000000	2.764051000000	-3.208897000000
C	1.445599000000	3.842771000000	-1.526285000000
C	2.010863000000	3.964871000000	-0.227071000000
C	1.132922000000	5.040897000000	-2.233564000000
H	2.251606000000	3.054797000000	0.335154000000
H	0.692123000000	4.966381000000	-3.240157000000
C	2.232441000000	5.227035000000	0.333071000000
C	1.367030000000	6.296774000000	-1.671742000000
H	2.640317000000	5.298183000000	1.352500000000
H	1.117227000000	7.206409000000	-2.238370000000
C	1.918058000000	6.394693000000	-0.381285000000
H	2.097053000000	7.383102000000	0.068819000000
N	2.235302000000	1.030976000000	0.242895000000
S	2.553794000000	0.335142000000	1.701538000000
O	1.562545000000	-0.726918000000	2.076034000000
O	2.655058000000	1.465490000000	2.671502000000
C	4.187549000000	-0.390256000000	1.574781000000
C	4.929312000000	-0.498997000000	2.762821000000
C	4.653578000000	-0.951121000000	0.375647000000

H	4.559311000000	-0.038725000000	3.688782000000
H	4.087497000000	-0.835683000000	-0.558657000000
C	6.122912000000	-1.228566000000	2.752081000000
C	5.855687000000	-1.668829000000	0.386717000000
H	6.690850000000	-1.340181000000	3.688373000000
H	6.220469000000	-2.119844000000	-0.549132000000
C	6.592459000000	-1.851512000000	1.576959000000
C	7.796207000000	-2.751704000000	1.606421000000
H	8.343364000000	-2.744381000000	0.643521000000
H	8.497718000000	-2.477156000000	2.417663000000
H	7.456964000000	-3.795145000000	1.794209000000
H	1.801242000000	-1.306831000000	4.107432000000
N	2.379547000000	-1.472504000000	4.947030000000
C	2.357467000000	-0.505876000000	5.940433000000
O	2.518405000000	-0.745407000000	7.138803000000
N	2.177414000000	0.774716000000	5.411491000000
H	2.262977000000	0.879783000000	4.387587000000
C	2.481229000000	-2.872051000000	5.321579000000
C	3.431658000000	-3.624141000000	4.403658000000
C	3.424359000000	-3.403487000000	3.030170000000
H	2.756241000000	-2.647822000000	2.595536000000
C	4.296237000000	-4.133893000000	2.184315000000
H	4.259066000000	-3.957439000000	1.094176000000
N	5.184232000000	-5.019036000000	2.612072000000
C	5.233369000000	-5.254997000000	3.957917000000
C	4.364515000000	-4.593347000000	4.908441000000
C	4.492791000000	-4.931751000000	6.289530000000
H	3.843748000000	-4.445241000000	7.030837000000
C	5.427111000000	-5.864671000000	6.714566000000
H	5.508603000000	-6.106414000000	7.785072000000
C	6.282142000000	-6.506822000000	5.779587000000
H	7.019510000000	-7.244675000000	6.130802000000
C	6.186147000000	-6.205650000000	4.430724000000
H	6.826719000000	-6.687476000000	3.677695000000
H	2.890785000000	-2.870136000000	6.349182000000
C	2.071628000000	1.980605000000	6.096266000000
C	1.954913000000	2.085986000000	7.501797000000
H	1.987740000000	1.173763000000	8.109237000000
C	1.830003000000	3.352133000000	8.091543000000
C	1.825454000000	4.529470000000	7.325679000000
C	1.952876000000	4.417369000000	5.932205000000
C	2.080097000000	3.165193000000	5.320496000000
H	2.210993000000	3.090356000000	4.231660000000
C	1.916887000000	5.648155000000	5.059535000000
F	0.679117000000	5.837248000000	4.511631000000
F	2.784067000000	5.556853000000	4.015787000000
F	2.216757000000	6.774281000000	5.741206000000
C	1.642403000000	3.444169000000	9.589095000000
F	2.270175000000	2.440438000000	10.242962000000
F	0.328053000000	3.376564000000	9.925622000000
F	2.115160000000	4.614773000000	10.084585000000
H	1.744082000000	5.512274000000	7.806737000000
C	1.037429000000	-3.484496000000	5.424017000000
C	0.985014000000	-5.007548000000	5.643400000000
N	0.188060000000	-3.057936000000	4.302841000000
C	-1.117632000000	-2.540345000000	4.721991000000
H	-0.936285000000	-1.858531000000	5.578927000000
H	-1.524507000000	-1.916040000000	3.901192000000
C	-2.152208000000	-3.619605000000	5.109025000000
H	-2.652600000000	-3.981458000000	4.185521000000
H	-2.961548000000	-3.151372000000	5.709150000000
C	-1.580977000000	-4.831764000000	5.866750000000
H	-2.404921000000	-5.564119000000	6.000884000000
H	-1.278257000000	-4.535568000000	6.895144000000
C	0.111856000000	-4.025983000000	3.215635000000
H	-0.485277000000	-3.582896000000	2.392177000000
H	1.131467000000	-4.176787000000	2.814557000000
C	-0.453190000000	-5.438184000000	3.640396000000
H	-1.523313000000	-5.494602000000	3.337554000000
C	-0.381832000000	-5.550099000000	5.179832000000
H	-0.438862000000	-6.622322000000	5.458668000000
C	0.262034000000	-6.516814000000	2.867050000000
H	0.244001000000	-6.367302000000	1.769711000000
C	0.925688000000	-7.587085000000	3.340151000000
H	1.000952000000	-7.807591000000	4.416703000000
H	1.426930000000	-8.290160000000	2.657240000000
H	1.167882000000	-5.249838000000	6.710795000000
H	1.782778000000	-5.515125000000	5.063383000000

H	0.621869000000	-2.986441000000	6.322806000000

TS$_{si\text{-}SR}$

Electronic energy = -4234.105691 Hartree

C	-2.198727000000	0.121997000000	-0.156695000000
O	-1.938920000000	-0.877006000000	0.688320000000
C	-3.023986000000	-0.161856000000	-1.250380000000
C	-1.600356000000	1.427104000000	0.012851000000
H	-1.443776000000	-0.504283000000	1.467001000000
H	-2.203672000000	2.204604000000	-0.482315000000
C	-1.177720000000	1.810459000000	1.389926000000
C	-0.311001000000	1.005463000000	2.167477000000
C	-1.672683000000	3.005071000000	1.955154000000
H	0.213830000000	0.140434000000	1.730667000000
H	-2.316678000000	3.660129000000	1.348445000000
C	0.001038000000	1.360967000000	3.489023000000
C	-1.349492000000	3.361534000000	3.270100000000
H	0.699885000000	0.732188000000	4.057509000000
H	-1.733584000000	4.302178000000	3.691229000000
C	-0.530768000000	2.529678000000	4.051384000000
H	-0.292182000000	2.805984000000	5.088475000000
C	-3.735097000000	-0.364767000000	-2.243072000000
C	-4.550766000000	-0.617989000000	-3.371283000000
C	-4.804845000000	0.405355000000	-4.325510000000
C	-5.127009000000	-1.903760000000	-3.565670000000
H	-4.360119000000	1.399708000000	-4.176161000000
H	-4.931227000000	-2.694439000000	-2.827324000000
C	-5.609422000000	0.143244000000	-5.437522000000
C	-5.931526000000	-2.149946000000	-4.681323000000
H	-5.800078000000	0.940188000000	-6.171724000000
H	-6.374650000000	-3.146911000000	-4.823559000000
C	-6.174912000000	-1.131210000000	-5.619466000000
H	-6.808391000000	-1.331276000000	-6.496590000000
C	2.241857000000	-3.956302000000	-0.406097000000
C	2.584984000000	-2.656975000000	-0.012646000000
C	1.128824000000	-4.202617000000	-1.241710000000
H	3.439252000000	-2.490416000000	0.652358000000
H	0.880937000000	-5.236874000000	-1.524011000000
C	1.791976000000	-1.581529000000	-0.470721000000
C	0.332077000000	-3.150138000000	-1.718814000000
H	-0.538101000000	-3.315107000000	-2.368963000000
C	0.694284000000	-1.864140000000	-1.316303000000
H	2.848395000000	-4.801841000000	-0.049732000000
C	0.672051000000	0.346250000000	-1.038457000000
C	1.787843000000	-0.122829000000	-0.281868000000
O	0.040450000000	-0.718961000000	-1.671933000000
C	-0.106738000000	1.553030000000	-1.167763000000
H	-0.705655000000	1.468392000000	-2.092803000000
C	0.349312000000	2.956727000000	-0.966890000000
C	1.253608000000	3.372410000000	0.036484000000
C	-0.267656000000	3.944506000000	-1.772873000000
H	1.756574000000	2.619228000000	0.656663000000
H	-0.983686000000	3.633785000000	-2.551721000000
C	1.533704000000	4.734540000000	0.215153000000
C	0.019157000000	5.302794000000	-1.597031000000
H	2.234632000000	5.039435000000	1.004823000000
H	-0.463382000000	6.053325000000	-2.241346000000
C	0.920336000000	5.701877000000	-0.595301000000
H	1.141774000000	6.769502000000	-0.444932000000
N	2.617195000000	0.705246000000	0.390471000000
S	3.420595000000	0.235043000000	1.710587000000
O	2.702783000000	-0.878006000000	2.434284000000
O	3.708646000000	1.436818000000	2.550193000000
C	5.020320000000	-0.415599000000	1.210143000000
C	5.885919000000	-0.869393000000	2.219926000000
C	5.374847000000	-0.510748000000	-0.142004000000
H	5.599610000000	-0.788350000000	3.278928000000
H	4.685340000000	-0.140086000000	-0.914256000000
C	7.108238000000	-1.446711000000	1.858741000000
C	6.606538000000	-1.086218000000	-0.483984000000
H	7.779991000000	-1.819563000000	2.647551000000
H	6.885673000000	-1.168904000000	-1.546149000000
C	7.490304000000	-1.567106000000	0.504331000000
C	8.819950000000	-2.167609000000	0.127720000000
H	8.769182000000	-2.693551000000	-0.845883000000
H	9.595721000000	-1.377524000000	0.032380000000
H	9.175967000000	-2.884027000000	0.893428000000
H	2.892562000000	-1.212437000000	4.171132000000

N	2.978691000000	-1.355814000000	5.194095000000
C	2.733512000000	-0.281176000000	6.019623000000
O	2.396445000000	-0.383691000000	7.202167000000
N	2.918159000000	0.949556000000	5.367997000000
H	3.297851000000	0.943564000000	4.407727000000
C	2.709829000000	-2.702217000000	5.674189000000
C	3.792630000000	-3.665145000000	5.229272000000
C	4.369980000000	-3.532324000000	3.968030000000
H	4.068592000000	-2.715415000000	3.299156000000
C	5.314949000000	-4.487007000000	3.515483000000
H	5.760215000000	-4.364427000000	2.509454000000
N	5.703892000000	-5.543569000000	4.213648000000
C	5.175108000000	-5.695755000000	5.464670000000
C	4.222049000000	-4.770046000000	6.039547000000
C	3.767551000000	-5.007441000000	7.372155000000
H	3.068504000000	-4.300724000000	7.840745000000
C	4.199995000000	-6.112996000000	8.089119000000
H	3.837866000000	-6.273333000000	9.115938000000
C	5.113049000000	-7.035548000000	7.511317000000
H	5.444921000000	-7.910799000000	8.090462000000
C	5.593545000000	-6.825665000000	6.229257000000
H	6.312045000000	-7.508545000000	5.753113000000
H	2.682648000000	-2.625388000000	6.778709000000
C	2.457639000000	2.194113000000	5.786325000000
C	1.798001000000	2.411723000000	7.018363000000
H	1.691761000000	1.574281000000	7.718035000000
C	1.280293000000	3.682377000000	7.311242000000
C	1.418632000000	4.760625000000	6.424443000000
C	2.093381000000	4.539797000000	5.211777000000
C	2.614383000000	3.283024000000	4.892022000000
H	3.112249000000	3.117095000000	3.926658000000
C	2.148471000000	5.648740000000	4.191528000000
F	0.991673000000	5.712561000000	3.471636000000
F	3.153763000000	5.480089000000	3.294530000000
F	2.314338000000	6.863530000000	4.765504000000
C	0.459001000000	3.860085000000	8.565901000000
F	0.866664000000	3.052202000000	9.569006000000
F	-0.850254000000	3.567955000000	8.327315000000
F	0.497347000000	5.135447000000	9.023653000000
H	1.013718000000	5.749858000000	6.674275000000
C	1.269217000000	-3.136672000000	5.221001000000
C	0.681597000000	-4.348322000000	5.968445000000
N	1.171825000000	-3.294040000000	3.764283000000
C	0.043347000000	-2.590971000000	3.163537000000
H	0.005749000000	-1.584571000000	3.626916000000
H	0.261611000000	-2.429041000000	2.084576000000
C	-1.319667000000	-3.298493000000	3.318662000000
H	-1.424010000000	-4.058856000000	2.515756000000
H	-2.138112000000	-2.570085000000	3.128156000000
C	-1.535771000000	-3.979305000000	4.682566000000
H	-2.508198000000	-4.513540000000	4.640000000000
H	-1.654100000000	-3.212335000000	5.479390000000
C	1.314160000000	-4.673109000000	3.313305000000
H	1.300312000000	-4.682276000000	2.204238000000
H	2.315276000000	-5.034084000000	3.616539000000
C	0.235161000000	-5.667203000000	3.898644000000
H	-0.539560000000	-5.843349000000	3.119371000000
C	-0.431467000000	-4.990826000000	5.115596000000
H	-0.926788000000	-5.771753000000	5.728277000000
C	0.880172000000	-7.004058000000	4.161517000000
H	1.405411000000	-7.426610000000	3.283206000000
C	0.923179000000	-7.702808000000	5.310089000000
H	0.436179000000	-7.353368000000	6.234211000000
H	1.458441000000	-8.662907000000	5.368905000000
H	0.296922000000	-4.029391000000	6.959468000000
H	1.459829000000	-5.113831000000	6.160332000000
H	0.656302000000	-2.251785000000	5.486202000000

IntA$_{si\text{-}SR}$

Electronic energy = -4234.16611 Hartree

C	-2.980744000000	0.554164000000	0.137797000000
O	-3.507007000000	-0.121165000000	1.010318000000
C	-3.403797000000	0.481602000000	-1.249234000000
C	-1.858928000000	1.592516000000	0.421084000000
H	2.427276000000	1.379924000000	-0.735227000000
H	-2.281756000000	2.552008000000	0.048243000000
C	-1.630093000000	1.740594000000	1.908889000000
C	-1.049310000000	0.724255000000	2.694570000000

C	-2.093801000000	2.906001000000	2.552928000000
H	-0.694938000000	-0.203160000000	2.224993000000
H	-2.545544000000	3.709344000000	1.949124000000
C	-0.939385000000	0.881410000000	4.084129000000
C	-1.981146000000	3.063030000000	3.941123000000
H	-0.486943000000	0.081984000000	4.689802000000
H	-2.337053000000	3.987405000000	4.419672000000
C	-1.408175000000	2.044331000000	4.715009000000
H	-1.317171000000	2.162442000000	5.804646000000
C	-3.760075000000	0.436466000000	-2.428511000000
C	-4.164732000000	0.378548000000	-3.792253000000
C	-3.558215000000	1.225262000000	-4.756224000000
C	-5.175474000000	-0.526454000000	-4.207496000000
H	-2.776925000000	1.929017000000	-4.433565000000
H	-5.645660000000	-1.181213000000	-3.459862000000
C	-3.954042000000	1.163776000000	-6.096392000000
C	-5.563109000000	-0.579322000000	-5.550575000000
H	-3.478723000000	1.824732000000	-6.836634000000
H	-6.347840000000	-1.284194000000	-5.863784000000
C	-4.955486000000	0.263054000000	-6.497108000000
H	-5.264468000000	0.217975000000	-7.552336000000
C	1.987550000000	-4.140756000000	-0.149147000000
C	2.406015000000	-2.803884000000	-0.153587000000
C	0.616269000000	-4.485927000000	-0.183519000000
H	3.472988000000	-2.542337000000	-0.129191000000
H	0.325967000000	-5.547074000000	-0.183017000000
C	1.419695000000	-1.797066000000	-0.196752000000
C	-0.382501000000	-3.500783000000	-0.224763000000
H	-1.453358000000	-3.744803000000	-0.256680000000
C	0.056743000000	-2.175891000000	-0.234552000000
H	2.740186000000	-4.942539000000	-0.117983000000
C	0.082750000000	0.044703000000	-0.297403000000
C	1.413428000000	-0.347613000000	-0.242317000000
O	-0.737511000000	-1.062906000000	-0.313742000000
C	-0.632803000000	1.344910000000	-0.522210000000
H	-1.102800000000	1.221726000000	-1.526670000000
C	0.330734000000	2.515184000000	-0.669390000000
C	0.708713000000	3.353720000000	0.397049000000
C	0.908964000000	2.736165000000	-1.940691000000
H	0.286862000000	3.197566000000	1.398337000000
H	0.618826000000	2.086770000000	-2.783025000000
C	1.634609000000	4.389716000000	0.193605000000
C	1.847257000000	3.760489000000	-2.139212000000
H	1.919293000000	5.030011000000	1.039448000000
H	2.288732000000	3.912775000000	-3.135918000000
C	2.210736000000	4.592471000000	-1.068063000000
H	2.946697000000	5.396665000000	-1.216386000000
N	2.568075000000	0.468817000000	-0.269893000000
S	3.332494000000	0.809855000000	1.230825000000
O	2.419744000000	0.354757000000	2.325875000000
O	3.776058000000	2.217201000000	1.187399000000
C	4.753949000000	-0.270855000000	1.271637000000
C	5.265594000000	-0.608286000000	2.536634000000
C	5.375566000000	-0.711081000000	0.093949000000
H	4.764434000000	-0.272548000000	3.455321000000
H	4.960972000000	-0.427338000000	-0.883753000000
C	6.396661000000	-1.425493000000	2.611341000000
C	6.498545000000	-1.544090000000	0.194622000000
H	6.780981000000	-1.716565000000	3.600995000000
H	6.974780000000	-1.917608000000	-0.725159000000
C	7.020063000000	-1.928278000000	1.448623000000
C	8.155635000000	-2.906357000000	1.563077000000
H	8.678619000000	-3.052161000000	0.598700000000
H	8.895681000000	-2.582343000000	2.321935000000
H	7.753774000000	-3.888998000000	1.899100000000
H	2.476220000000	-1.169395000000	3.815284000000
N	2.645781000000	-1.324188000000	4.814824000000
C	2.382768000000	-0.287706000000	5.692662000000
O	2.229940000000	-0.451047000000	6.905354000000
N	2.352948000000	0.950370000000	5.049556000000
H	2.465774000000	0.918400000000	4.024928000000
C	2.666329000000	-2.693909000000	5.307474000000
C	3.716906000000	-3.493324000000	4.559580000000
C	3.732668000000	-3.490391000000	3.167011000000
H	2.958505000000	-2.952899000000	2.601060000000
C	4.736051000000	-4.205256000000	2.467635000000
H	4.739939000000	-4.185606000000	1.362605000000
N	5.708993000000	-4.890022000000	3.049747000000

C	5.720584000000	-4.928417000000	4.416715000000
C	4.734544000000	-4.250737000000	5.232732000000
C	4.838971000000	-4.365141000000	6.651771000000
H	4.101930000000	-3.862337000000	7.293434000000
C	5.857065000000	-5.103373000000	7.237020000000
H	5.916119000000	-5.176855000000	8.333374000000
C	6.825662000000	-5.761506000000	6.433230000000
H	7.628724000000	-6.342471000000	6.911637000000
C	6.757671000000	-5.673287000000	5.052270000000
H	7.486476000000	-6.174587000000	4.398838000000
H	2.940867000000	-2.604195000000	6.374965000000
C	1.872199000000	2.155876000000	5.544325000000
C	1.388095000000	2.325438000000	6.860987000000
H	1.435556000000	1.479134000000	7.557035000000
C	0.829420000000	3.554570000000	7.237020000000
C	0.769498000000	4.641342000000	6.351191000000
C	1.288193000000	4.474982000000	5.057686000000
C	1.837331000000	3.253581000000	4.652715000000
H	2.233706000000	3.139004000000	3.632273000000
C	1.202475000000	5.597195000000	4.054115000000
F	0.205764000000	5.383440000000	3.147477000000
F	2.350777000000	5.717963000000	3.334170000000
F	0.958334000000	6.792477000000	4.633273000000
C	0.173641000000	3.660285000000	8.592992000000
F	0.834341000000	2.957416000000	9.539369000000
F	-1.096834000000	3.166693000000	8.548081000000
F	0.081628000000	4.941792000000	9.020624000000
H	0.335468000000	5.598855000000	6.664505000000
C	1.233198000000	-3.309091000000	5.294833000000
C	1.077553000000	-4.552631000000	6.187365000000
N	0.712379000000	-3.529092000000	3.936211000000
C	-0.623270000000	-2.964313000000	3.732802000000
H	-0.603404000000	-1.933683000000	4.136893000000
H	-0.804749000000	-2.861092000000	2.640991000000
C	-1.782173000000	-3.760281000000	4.372226000000
H	-2.102355000000	-4.558237000000	3.669278000000
H	-2.664766000000	-3.093773000000	4.474116000000
C	-1.458858000000	-4.407043000000	5.732933000000
H	-2.338904000000	-5.015483000000	6.028980000000
H	-1.362762000000	-3.626448000000	6.519072000000
C	0.834510000000	-4.916200000000	3.492446000000
H	0.495025000000	-4.976877000000	2.439671000000
H	1.909121000000	-5.181983000000	3.486856000000
C	0.076306000000	-5.965798000000	4.391952000000
H	-0.898680000000	-6.199259000000	3.910068000000
C	-0.193136000000	-5.316914000000	5.765895000000
H	-0.382974000000	-6.118043000000	6.509178000000
C	0.851449000000	-7.257989000000	4.412803000000
H	1.072029000000	-7.663302000000	3.406212000000
C	1.314793000000	-7.938352000000	5.476536000000
H	1.144078000000	-7.603195000000	6.511954000000
H	1.886842000000	-8.870152000000	5.348352000000
H	1.028033000000	-4.242439000000	7.251699000000
H	1.956100000000	-5.225687000000	6.090956000000
H	0.620402000000	-2.503281000000	5.746079000000

RC$_{re\text{-}SS}$

Electronic energy = -4234.156901 Hartree

C	2.633614000000	1.571854000000	0.782976000000
O	1.968303000000	0.422640000000	1.028622000000
C	3.791528000000	1.554661000000	-0.047378000000
C	2.268322000000	2.789962000000	1.326215000000
H	2.288324000000	-0.403089000000	0.479547000000
H	2.909771000000	3.628811000000	1.018947000000
C	1.193634000000	3.124828000000	2.240065000000
C	0.310291000000	2.176704000000	2.822798000000
C	1.015781000000	4.492758000000	2.589235000000
H	0.419921000000	1.118071000000	2.566577000000
H	1.691666000000	5.243083000000	2.149705000000
C	-0.703007000000	2.586855000000	3.694892000000
C	0.001675000000	4.896303000000	3.462004000000
H	-1.382652000000	1.833267000000	4.121086000000
H	-0.116982000000	5.962484000000	3.707815000000
C	-0.870036000000	3.943810000000	4.017859000000
H	-1.677839000000	4.259323000000	4.694930000000
C	4.806654000000	1.593271000000	-0.749463000000
C	5.951313000000	1.652823000000	-1.587879000000
C	6.539266000000	0.473264000000	-2.117192000000

C	6.524335000000	2.907222000000	-1.928909000000
H	6.117181000000	-0.504451000000	-1.846248000000
H	6.073766000000	3.823697000000	-1.523360000000
C	7.648008000000	0.550702000000	-2.966748000000
C	7.635393000000	2.972595000000	-2.776144000000
H	8.089059000000	-0.374357000000	-3.368297000000
H	8.066701000000	3.952909000000	-3.030530000000
C	8.200453000000	1.798390000000	-3.303219000000
H	9.073315000000	1.855104000000	-3.970695000000
C	-3.841970000000	4.511562000000	1.941886000000
C	-3.025498000000	3.662593000000	1.191283000000
C	-3.542078000000	5.887592000000	2.067136000000
H	-3.248296000000	2.593942000000	1.118689000000
H	-4.200849000000	6.532573000000	2.668292000000
C	-1.895629000000	4.205102000000	0.535959000000
C	-2.420290000000	6.449372000000	1.443959000000
H	-2.168741000000	7.515035000000	1.531668000000
C	-1.621027000000	5.586928000000	0.687315000000
H	-4.729130000000	4.095974000000	2.440911000000
C	-0.015570000000	4.859851000000	-0.674851000000
C	-0.873298000000	3.696064000000	-0.378549000000
O	-0.516851000000	5.976487000000	-0.001385000000
C	1.125861000000	5.092271000000	-1.404196000000
H	1.439942000000	6.145655000000	-1.297767000000
C	1.995686000000	4.322776000000	-2.271484000000
C	1.846738000000	2.950206000000	-2.612615000000
C	3.094899000000	5.035772000000	-2.835385000000
H	1.012779000000	2.384945000000	-2.176476000000
H	3.224834000000	6.099553000000	-2.581346000000
C	2.757042000000	2.338525000000	-3.480763000000
C	3.998125000000	4.414714000000	-3.698801000000
H	2.626935000000	1.275171000000	-3.731473000000
H	4.841226000000	4.984771000000	-4.116793000000
C	3.833128000000	3.057410000000	-4.026162000000
H	4.551055000000	2.560436000000	-4.695627000000
N	-0.629528000000	2.542514000000	-0.969596000000
S	-1.267881000000	1.072893000000	-0.558784000000
O	-1.693144000000	1.026556000000	0.882984000000
O	-0.275030000000	0.050362000000	-0.972354000000
C	-2.725749000000	0.823283000000	-1.573859000000
C	-2.912650000000	-0.469803000000	-2.086729000000
C	-3.730785000000	1.797491000000	-1.686460000000
H	-2.108571000000	-1.212508000000	-1.991311000000
H	-3.583670000000	2.812789000000	-1.293470000000
C	-4.137309000000	-0.799734000000	-2.678037000000
C	-4.944072000000	1.451269000000	-2.291000000000
H	-4.305524000000	-1.826466000000	-3.035505000000
H	-5.744092000000	2.204343000000	-2.356020000000
C	-5.180211000000	0.143959000000	-2.770489000000
C	-1.269514000000	-2.689591000000	1.055006000000
O	-1.513439000000	-3.879789000000	0.837853000000
N	-2.252796000000	-1.711596000000	1.175803000000
N	0.012210000000	-2.195765000000	1.242917000000
H	0.189835000000	-1.201307000000	1.061740000000
H	-1.939976000000	-0.747303000000	1.372104000000
C	-3.592011000000	-1.806117000000	0.812555000000
C	-4.396363000000	-0.659753000000	1.020614000000
C	-4.173289000000	-2.943180000000	0.205783000000
H	-3.950178000000	0.226331000000	1.489329000000
H	-3.561063000000	-3.840743000000	0.059424000000
C	-5.727702000000	-0.634025000000	0.598660000000
C	-5.509576000000	-2.890972000000	-0.216979000000
C	-6.304971000000	-1.750239000000	-0.027998000000
H	-7.346615000000	-1.728600000000	-0.373657000000
C	-6.078352000000	-4.045451000000	-1.005250000000
F	-5.445281000000	-5.209673000000	-0.757565000000
F	-7.397365000000	-4.229109000000	-0.763555000000
F	-5.962566000000	-3.811893000000	-2.352241000000
C	-6.560425000000	0.599716000000	0.845993000000
F	-7.442134000000	0.823312000000	-0.164991000000
F	-7.288295000000	0.503313000000	1.983728000000
F	-5.796099000000	1.720091000000	0.962475000000
C	1.118689000000	-3.132567000000	1.150596000000
H	0.607929000000	-4.112143000000	1.066493000000
C	1.985840000000	-3.152785000000	2.403443000000
C	2.458918000000	-4.389448000000	2.967517000000
C	3.365420000000	-4.310829000000	4.093916000000
N	3.747540000000	-3.130297000000	4.664546000000

C	3.233242000000	-2.019329000000	4.161568000000
C	2.356240000000	-1.975572000000	3.046478000000
H	3.531709000000	-1.070793000000	4.645309000000
H	2.009558000000	-0.998749000000	2.683242000000
C	2.592754000000	-6.836145000000	3.081139000000
C	2.079104000000	-5.685604000000	2.501224000000
C	3.515388000000	-6.752678000000	4.157962000000
H	1.354226000000	-5.779994000000	1.680923000000
H	3.920036000000	-7.673903000000	4.604031000000
C	3.887219000000	-5.514835000000	4.655389000000
H	4.580965000000	-5.406271000000	5.501579000000
H	2.275383000000	-7.821749000000	2.708391000000
C	4.104967000000	-1.965611000000	-0.000655000000
C	4.868862000000	-3.051079000000	-0.826322000000
C	3.834219000000	-3.981423000000	-1.518617000000
C	3.308461000000	-3.383508000000	-2.850898000000
C	3.266259000000	-1.850073000000	-2.888753000000
C	1.885525000000	-3.005110000000	-0.203564000000
C	2.713569000000	-4.269077000000	-0.512547000000
H	4.111391000000	-2.252742000000	1.068563000000
H	5.481445000000	-2.561075000000	-1.611728000000
H	4.338761000000	-4.941646000000	-1.751245000000
H	3.956122000000	-3.728511000000	-3.683893000000
H	2.300744000000	-3.804851000000	-3.059593000000
H	2.808638000000	-1.512005000000	-3.843235000000
H	1.069888000000	-2.935141000000	-0.949407000000
H	2.034812000000	-5.062702000000	-0.887129000000
H	3.169839000000	-4.669902000000	0.414403000000
C	2.510580000000	-1.171990000000	-1.736329000000
H	1.421093000000	-1.155795000000	-1.922175000000
H	4.305994000000	-1.463183000000	-2.918805000000
N	2.690143000000	-1.756541000000	-0.380274000000
C	5.818406000000	-3.800672000000	0.068505000000
C	7.158614000000	-3.758216000000	-0.023798000000
H	7.660330000000	-3.155011000000	-0.799957000000
H	7.804173000000	-4.316869000000	0.671259000000
H	5.355700000000	-4.404571000000	0.872672000000
H	2.820690000000	-0.109443000000	-1.684675000000
H	4.626663000000	-0.991709000000	-0.057586000000
C	-6.514860000000	-0.236494000000	-3.353319000000
H	-6.652174000000	-1.334614000000	-3.350726000000
H	-7.340753000000	0.221629000000	-2.774613000000
H	-6.610224000000	0.116101000000	-4.402584000000

TS$_{re\text{-}SS}$

Electronic energy = -4234.140315 Hartree

C	2.745693000000	2.022358000000	0.545141000000
O	1.966512000000	1.021841000000	0.567278000000
C	4.002994000000	1.889567000000	-0.162020000000
C	2.480592000000	3.298327000000	1.173178000000
H	2.168871000000	-0.496372000000	-0.056481000000
H	3.377366000000	3.938407000000	1.214130000000
C	1.567770000000	3.442496000000	2.314837000000
C	0.406745000000	2.644926000000	2.467909000000
C	1.808709000000	4.467472000000	3.263202000000
H	0.179729000000	1.861732000000	1.737261000000
H	2.703024000000	5.101525000000	3.151785000000
C	-0.477903000000	2.874674000000	3.530474000000
C	0.926764000000	4.687102000000	4.326125000000
H	-1.386698000000	2.259252000000	3.609195000000
H	1.133575000000	5.489165000000	5.051276000000
C	-0.226618000000	3.893057000000	4.461140000000
H	-0.930630000000	4.076414000000	5.286980000000
C	5.041116000000	1.592281000000	-0.759115000000
C	6.199717000000	1.122955000000	-1.446227000000
C	7.098472000000	0.234712000000	-0.798818000000
C	6.435126000000	1.476415000000	-2.799128000000
H	6.921974000000	-0.042307000000	0.250869000000
H	5.732751000000	2.160175000000	-3.295447000000
C	8.195684000000	-0.287628000000	-1.494032000000
C	7.536618000000	0.946738000000	-3.481580000000
H	8.885293000000	-0.974282000000	-0.979795000000
H	7.708450000000	1.225943000000	-4.532192000000
C	8.418527000000	0.063601000000	-2.836102000000
H	9.282201000000	-0.349345000000	-3.378527000000
C	-4.077294000000	4.254250000000	1.751060000000
C	-3.318001000000	3.520846000000	0.835258000000
C	-3.551669000000	5.410863000000	2.378962000000

H	-3.732542000000	2.619921000000	0.368152000000
H	-4.173818000000	5.963799000000	3.099011000000
C	-2.007358000000	3.958161000000	0.542518000000
C	-2.251999000000	5.860412000000	2.110604000000
H	-1.821094000000	6.744345000000	2.600465000000
C	-1.505485000000	5.109607000000	1.195995000000
H	-5.099310000000	3.922123000000	1.986527000000
C	0.151784000000	4.377423000000	-0.095045000000
C	-0.931019000000	3.481164000000	-0.312238000000
O	-0.227402000000	5.368646000000	0.816246000000
C	1.547079000000	4.414970000000	-0.401173000000
H	1.978155000000	5.341557000000	0.012092000000
C	2.210411000000	4.017564000000	-1.662317000000
C	1.757092000000	2.978181000000	-2.509129000000
C	3.376755000000	4.728405000000	-2.037679000000
H	0.849281000000	2.424622000000	-2.221865000000
H	3.749186000000	5.533908000000	-1.384842000000
C	2.437283000000	2.689435000000	-3.699946000000
C	4.050820000000	4.438419000000	-3.230050000000
H	2.056600000000	1.889927000000	-4.355211000000
H	4.945050000000	5.017611000000	-3.506720000000
C	3.578403000000	3.418687000000	-4.073404000000
H	4.092384000000	3.200269000000	-5.022516000000
N	-0.916551000000	2.445463000000	-1.202376000000
S	-1.331149000000	0.963046000000	-0.811152000000
O	-1.682029000000	0.833611000000	0.667661000000
O	-0.296155000000	-0.032279000000	-1.251364000000
C	-2.797712000000	0.512020000000	-1.754474000000
C	-2.989107000000	-0.841501000000	-2.073878000000
C	-3.781486000000	1.463309000000	-2.061582000000
H	-2.198925000000	-1.570793000000	-1.849641000000
H	-3.598220000000	2.527061000000	-1.853620000000
C	-4.196646000000	-1.248199000000	-2.653028000000
C	-4.986559000000	1.040252000000	-2.636347000000
H	-4.363521000000	-2.315201000000	-2.864676000000
H	-5.770342000000	1.782963000000	-2.851751000000
C	-5.224593000000	-0.321786000000	-2.922478000000
C	-1.535822000000	-2.577427000000	0.977575000000
O	-1.648243000000	-3.792732000000	0.792169000000

N	-2.590769000000	-1.690932000000	1.143784000000
N	-0.309703000000	-1.931131000000	1.072630000000
H	-0.292148000000	-0.914825000000	0.928980000000
H	-2.304992000000	-0.691189000000	1.193599000000
C	-3.922250000000	-1.867021000000	0.788709000000
C	-4.740696000000	-0.711816000000	0.828608000000
C	-4.476275000000	-3.086149000000	0.341849000000
H	-4.303656000000	0.240909000000	1.158191000000
H	-3.842813000000	-3.980995000000	0.310272000000
C	-6.068966000000	-0.766685000000	0.403626000000
C	-5.809788000000	-3.112736000000	-0.095325000000
C	-6.623119000000	-1.969927000000	-0.065796000000
H	-7.661517000000	-2.012526000000	-0.419095000000
C	-6.346210000000	-4.370561000000	-0.733052000000
F	-5.740136000000	-5.486115000000	-0.274341000000
F	-7.678540000000	-4.515099000000	-0.542376000000
F	-6.148924000000	-4.344141000000	-2.088489000000
C	-6.926315000000	0.473804000000	0.469760000000
F	-7.763448000000	0.564040000000	-0.598550000000
F	-7.707025000000	0.489168000000	1.576852000000
F	-6.184405000000	1.612045000000	0.492458000000
C	0.903158000000	-2.696427000000	0.935466000000
H	0.541001000000	-3.745970000000	0.926824000000
C	1.838768000000	-2.491814000000	2.123278000000
C	2.618170000000	-3.563484000000	2.680262000000
C	3.630941000000	-3.215008000000	3.654372000000
N	3.812671000000	-1.943652000000	4.119638000000
C	2.989162000000	-1.007873000000	3.672786000000
C	1.995451000000	-1.226923000000	2.682718000000
H	3.116969000000	0.009900000000	4.084794000000
H	1.402175000000	-0.369641000000	2.337210000000
C	3.283903000000	-5.914919000000	2.881232000000
C	2.451704000000	-4.942078000000	2.345813000000
C	4.325733000000	-5.558356000000	3.779079000000
H	1.630867000000	-5.245708000000	1.680620000000
H	4.986426000000	-6.338535000000	4.186342000000
C	4.485603000000	-4.237486000000	4.165270000000
H	5.255410000000	-3.925709000000	4.885731000000
H	3.127234000000	-6.971493000000	2.617103000000

C	3.904900000000	-1.616803000000	-0.175424000000
C	4.612110000000	-2.792364000000	-0.901795000000
C	3.583852000000	-3.597769000000	-1.744791000000
C	3.310021000000	-2.919912000000	-3.110199000000
C	3.367611000000	-1.390584000000	-3.061786000000
C	1.582670000000	-2.543845000000	-0.476224000000
C	2.325673000000	-3.828495000000	-0.897706000000
H	3.831559000000	-1.818274000000	0.906777000000
H	5.379530000000	-2.378418000000	-1.586864000000
H	4.025158000000	-4.595369000000	-1.943924000000
H	4.062157000000	-3.271588000000	-3.846209000000
H	2.322724000000	-3.258186000000	-3.493070000000
H	3.095127000000	-0.964852000000	-4.049809000000
H	0.743205000000	-2.337126000000	-1.163762000000
H	1.607817000000	-4.483989000000	-1.430230000000
H	2.634391000000	-4.386163000000	0.005824000000
C	2.448343000000	-0.741400000000	-2.040876000000
H	1.383531000000	-0.766435000000	-2.330709000000
H	4.412354000000	-1.059863000000	-2.884870000000
N	2.500037000000	-1.338107000000	-0.642519000000
C	5.337731000000	-3.646232000000	0.108460000000
C	6.671058000000	-3.810099000000	0.142645000000
H	7.328068000000	-3.314887000000	-0.592726000000
H	7.151115000000	-4.442092000000	0.905470000000
H	4.714781000000	-4.146580000000	0.872618000000
H	2.706596000000	0.326849000000	-1.914755000000
H	4.459559000000	-0.671795000000	-0.289424000000
C	-6.539860000000	-0.773005000000	-3.501664000000
H	-6.663580000000	-1.868944000000	-3.409153000000
H	-7.385423000000	-0.282861000000	-2.980632000000
H	-6.617140000000	-0.509857000000	-4.578169000000

IntA$_{re\text{-}SS}$

Electronic energy = -4234.169503 Hartree

C	3.006492000000	2.203182000000	0.722382000000
O	3.154272000000	1.230344000000	1.466500000000
C	3.136524000000	1.987083000000	-0.705121000000
C	2.854226000000	3.620469000000	1.290054000000
H	1.325614000000	-1.572650000000	-0.227377000000
H	3.895891000000	4.007993000000	1.199649000000
C	2.500118000000	3.605496000000	2.763259000000
C	1.403395000000	2.860772000000	3.248594000000
C	3.233458000000	4.393631000000	3.668951000000
H	0.822367000000	2.228377000000	2.563848000000
H	4.093912000000	4.977142000000	3.301966000000
C	1.055183000000	2.911926000000	4.605448000000
C	2.886562000000	4.445764000000	5.028240000000
H	0.196573000000	2.325055000000	4.965529000000
H	3.475222000000	5.066848000000	5.720555000000
C	1.793310000000	3.704412000000	5.499735000000
H	1.517324000000	3.741094000000	6.564577000000
C	3.115487000000	1.519407000000	-1.844379000000
C	2.887863000000	0.921325000000	-3.113799000000
C	3.908192000000	0.254585000000	-3.837045000000
C	1.562245000000	0.918353000000	-3.624160000000
H	4.936423000000	0.262748000000	-3.446263000000
H	0.775606000000	1.429796000000	-3.053761000000
C	3.603141000000	-0.407160000000	-5.034025000000
C	1.268925000000	0.241712000000	-4.812806000000
H	4.402315000000	-0.919962000000	-5.590826000000
H	0.231995000000	0.229870000000	-5.182077000000
C	2.285126000000	-0.423943000000	-5.521742000000
H	2.050481000000	-0.952550000000	-6.457993000000
C	-3.892802000000	3.843476000000	1.862266000000
C	-2.850078000000	3.116845000000	1.275645000000
C	-3.706432000000	5.174843000000	2.302422000000
H	-2.996003000000	2.072996000000	0.971268000000
H	-4.545986000000	5.717349000000	2.762434000000
C	-1.593948000000	3.743626000000	1.111493000000
C	-2.465011000000	5.814162000000	2.169162000000
H	-2.295452000000	6.844680000000	2.511736000000
C	-1.435205000000	5.078441000000	1.576372000000
H	-4.876435000000	3.365194000000	1.983797000000
C	0.517164000000	4.489740000000	0.738042000000
C	-0.297173000000	3.382813000000	0.552924000000
O	-0.163570000000	5.523246000000	1.357097000000
C	1.977268000000	4.676673000000	0.497483000000
H	2.223490000000	5.641447000000	0.989025000000

C	2.395720000000	4.803401000000	-0.968056000000
C	1.520791000000	4.501297000000	-2.028327000000
C	3.704586000000	5.226646000000	-1.277349000000
H	0.497653000000	4.174196000000	-1.799261000000
H	4.398230000000	5.495356000000	-0.463713000000
C	1.956519000000	4.577943000000	-3.358735000000
C	4.143193000000	5.304533000000	-2.606233000000
H	1.262585000000	4.318737000000	-4.173073000000
H	5.171347000000	5.631541000000	-2.824946000000
C	3.271675000000	4.968245000000	-3.654178000000
H	3.614471000000	5.019323000000	-4.698810000000
N	0.220287000000	2.256591000000	-0.065077000000
S	-0.407744000000	0.830844000000	-0.134023000000
O	-1.210314000000	0.390698000000	1.089576000000
O	0.695862000000	-0.141165000000	-0.486976000000
C	-1.518083000000	0.719908000000	-1.564162000000
C	-1.505952000000	-0.444695000000	-2.347712000000
C	-2.385089000000	1.777988000000	-1.885804000000
H	-0.815138000000	-1.259984000000	-2.100369000000
H	-2.384617000000	2.703536000000	-1.293399000000
C	-2.387542000000	-0.566076000000	-3.428412000000
C	-3.265200000000	1.639839000000	-2.966869000000
H	-2.401443000000	-1.499061000000	-4.012248000000
H	-3.962992000000	2.459918000000	-3.196146000000
C	-3.293898000000	0.464746000000	-3.749209000000
C	-1.343156000000	-2.920787000000	0.469226000000
O	-1.289560000000	-4.020056000000	-0.098524000000
N	-2.401478000000	-2.025241000000	0.395890000000
N	-0.326090000000	-2.438243000000	1.268912000000
H	-0.402292000000	-1.477284000000	1.627119000000
H	-2.200274000000	-1.097498000000	0.820055000000
C	-3.480036000000	-2.032199000000	-0.481444000000
C	-4.326771000000	-0.899655000000	-0.446011000000
C	-3.739552000000	-3.055214000000	-1.417805000000
H	-4.120636000000	-0.093546000000	0.271736000000
H	-3.084213000000	-3.933993000000	-1.445648000000
C	-5.387641000000	-0.776667000000	-1.344709000000
C	-4.802878000000	-2.903363000000	-2.321350000000
C	-5.640579000000	-1.778632000000	-2.296785000000
H	-6.462690000000	-1.676590000000	-3.017129000000
C	-4.965913000000	-3.913224000000	-3.430381000000
F	-4.536318000000	-5.144005000000	-3.078248000000
F	-6.249533000000	-4.023489000000	-3.841680000000
F	-4.235702000000	-3.541469000000	-4.528917000000
C	-6.285528000000	0.435855000000	-1.273408000000
F	-6.653103000000	0.859242000000	-2.513428000000
F	-7.429910000000	0.171786000000	-0.598324000000
F	-5.685282000000	1.480103000000	-0.653042000000
C	0.822547000000	-3.244986000000	1.625670000000
H	0.450809000000	-4.243041000000	1.939667000000
C	1.581383000000	-2.609295000000	2.780583000000
C	2.126967000000	-3.409270000000	3.843909000000
C	2.892247000000	-2.724102000000	4.863880000000
N	3.103779000000	-1.374365000000	4.861527000000
C	2.567151000000	-0.667851000000	3.880356000000
C	1.799049000000	-1.236482000000	2.825499000000
H	2.747592000000	0.420534000000	3.871815000000
H	1.418876000000	-0.559285000000	2.045834000000
C	2.531589000000	-5.526161000000	5.012614000000
C	1.965716000000	-4.823223000000	3.958677000000
C	3.290367000000	-4.851863000000	6.005503000000
H	1.380458000000	-5.372205000000	3.207095000000
H	3.734613000000	-5.422409000000	6.835065000000
C	3.463399000000	-3.479513000000	5.930845000000
H	4.037524000000	-2.917582000000	6.681485000000
H	2.386941000000	-6.614905000000	5.081061000000
C	3.374262000000	-1.743440000000	-0.160727000000
C	4.557806000000	-2.737033000000	-0.296559000000
C	4.026988000000	-4.194317000000	-0.413898000000
C	3.529328000000	-4.515654000000	-1.850766000000
C	2.945223000000	-3.317556000000	-2.613631000000
C	1.695841000000	-3.582807000000	0.373016000000
C	2.951818000000	-4.409365000000	0.661067000000
H	3.315536000000	-1.325682000000	0.859616000000
H	5.126808000000	-2.505319000000	-1.220381000000
H	4.871894000000	-4.874002000000	-0.184466000000
H	4.380223000000	-4.906653000000	-2.445377000000
H	2.788483000000	-5.342924000000	-1.801669000000

H	2.579116000000	-3.639981000000	-3.609774000000
H	1.003668000000	-4.160079000000	-0.267331000000
H	2.665599000000	-5.478412000000	0.721243000000
H	3.374042000000	-4.145584000000	1.650532000000
C	1.798960000000	-2.586217000000	-1.925532000000
H	0.843888000000	-3.143695000000	-1.991935000000
H	3.748708000000	-2.584416000000	-2.829152000000
N	2.027134000000	-2.352396000000	-0.450784000000
C	5.503151000000	-2.559856000000	0.865704000000
C	6.801896000000	-2.238306000000	0.749646000000
H	7.266240000000	-2.073860000000	-0.237516000000
H	7.447067000000	-2.121392000000	1.633647000000
H	5.070720000000	-2.694521000000	1.874964000000
H	1.642225000000	-1.586404000000	-2.374287000000
H	3.473969000000	-0.868697000000	-0.827893000000
C	-4.262093000000	0.313952000000	-4.893273000000
H	-4.388986000000	-0.750095000000	-5.171249000000
H	-5.254877000000	0.725334000000	-4.626870000000
H	-3.908522000000	0.860219000000	-5.793935000000

RC*re-SR*

Electronic energy = -4234.150835 Hartree

C	3.180457000000	-1.605241000000	-0.140016000000
O	3.212882000000	-0.294851000000	-0.472954000000
C	3.933115000000	-1.883005000000	1.036159000000
C	2.461745000000	-2.529252000000	-0.873209000000
H	2.816515000000	-0.114234000000	-1.418884000000
H	1.879715000000	-2.099669000000	-1.702455000000
C	2.304923000000	-3.966201000000	-0.721599000000
C	1.467742000000	-4.634079000000	-1.658484000000
C	2.911694000000	-4.757483000000	0.291539000000
H	0.955086000000	-4.038401000000	-2.429683000000
H	3.565540000000	-4.277791000000	1.031082000000
C	1.251101000000	-6.013884000000	-1.591631000000
C	2.684962000000	-6.135627000000	0.358767000000
H	0.587763000000	-6.495776000000	-2.325517000000
H	3.163911000000	-6.722406000000	1.158145000000
C	1.854985000000	-6.776227000000	-0.578855000000
H	1.677847000000	-7.860452000000	-0.516840000000
C	4.558114000000	-1.994292000000	2.092972000000
C	5.225799000000	-2.104681000000	3.342216000000
C	5.326455000000	-3.351889000000	4.013019000000
C	5.778719000000	-0.950402000000	3.958854000000
H	4.899282000000	-4.249052000000	3.541084000000
H	5.695396000000	0.017250000000	3.443070000000
C	5.958315000000	-3.436159000000	5.258777000000
C	6.407644000000	-1.047177000000	5.204516000000
H	6.029025000000	-4.409391000000	5.767792000000
H	6.830962000000	-0.144848000000	5.671463000000
C	6.500551000000	-2.287393000000	5.859830000000
H	6.995927000000	-2.358509000000	6.839770000000
C	-2.329882000000	-6.282666000000	-0.734113000000
C	-2.007230000000	-4.927218000000	-0.818183000000
C	-1.863730000000	-7.083199000000	0.336002000000
H	-2.337861000000	-4.333505000000	-1.675874000000
H	-2.132045000000	-8.150013000000	0.370338000000
C	-1.207812000000	-4.354022000000	0.199538000000
C	-1.054695000000	-6.549393000000	1.344411000000
H	-0.666879000000	-7.154393000000	2.174778000000
C	-0.744854000000	-5.188182000000	1.248971000000
H	-2.949531000000	-6.738551000000	-1.519920000000
C	0.089457000000	-3.180093000000	1.729828000000
C	-0.671511000000	-3.024347000000	0.482135000000
O	0.020235000000	-4.517213000000	2.139726000000
C	0.838271000000	-2.386798000000	2.568260000000
H	1.275911000000	-3.002468000000	3.373346000000
C	1.175927000000	-0.988930000000	2.683852000000
C	0.715030000000	0.068085000000	1.850041000000
C	2.050496000000	-0.666203000000	3.763556000000
H	0.058262000000	-0.172267000000	1.001754000000
H	2.416775000000	-1.477405000000	4.411036000000
C	1.107756000000	1.384941000000	2.112153000000
C	2.458967000000	0.645690000000	3.999697000000
H	0.732431000000	2.212628000000	1.493686000000
H	3.147596000000	0.863953000000	4.829536000000
C	1.984336000000	1.677250000000	3.172462000000
H	2.296298000000	2.718139000000	3.346069000000
N	-0.756724000000	-1.842884000000	-0.120366000000

S	-1.569393000000	-1.629911000000	-1.552688000000
O	-1.172275000000	-2.570187000000	-2.633331000000
O	-1.405631000000	-0.173772000000	-1.875889000000
C	-3.307497000000	-1.852455000000	-1.169170000000
C	-4.158462000000	-2.325854000000	-2.177943000000
C	-3.803358000000	-1.477242000000	0.090023000000
H	-3.741561000000	-2.605728000000	-3.156902000000
H	-3.123334000000	-1.091031000000	0.863271000000
C	-5.529173000000	-2.434457000000	-1.908880000000
C	-5.173796000000	-1.601329000000	0.339256000000
H	-6.205908000000	-2.801172000000	-2.696235000000
H	-5.572779000000	-1.295385000000	1.317632000000
C	-6.059876000000	-2.080708000000	-0.650946000000
C	-0.654658000000	2.769899000000	-0.577427000000
O	-0.307039000000	3.634023000000	0.231153000000
N	-1.956420000000	2.270061000000	-0.663131000000
N	0.188370000000	2.207259000000	-1.522572000000
H	-0.150468000000	1.324757000000	-1.926069000000
H	-2.073434000000	1.420168000000	-1.234344000000
C	-3.077266000000	2.700518000000	0.038255000000
C	-4.307778000000	2.049607000000	-0.231795000000
C	-3.058978000000	3.756255000000	0.977905000000
H	-4.344624000000	1.234567000000	-0.968839000000
H	-2.109326000000	4.265039000000	1.182011000000
C	-5.479545000000	2.451226000000	0.417001000000
C	-4.247337000000	4.137079000000	1.619730000000
C	-5.465919000000	3.500983000000	1.352398000000
H	-6.387971000000	3.826549000000	1.850275000000
C	-4.177206000000	5.263230000000	2.627567000000
F	-3.589808000000	6.363367000000	2.100271000000
F	-5.404615000000	5.630232000000	3.069211000000
F	-3.447886000000	4.907870000000	3.713728000000
C	-6.784334000000	1.749565000000	0.135977000000
F	-7.113197000000	0.884623000000	1.144478000000
F	-7.813326000000	2.620450000000	0.032210000000
F	-6.748876000000	1.019221000000	-1.004873000000
C	1.642607000000	2.338131000000	-1.451783000000
H	2.023385000000	1.661633000000	-0.661880000000
C	2.112949000000	3.750985000000	-1.140402000000
C	3.262953000000	3.959627000000	-0.305807000000
C	3.709383000000	5.323885000000	-0.120361000000
N	3.102951000000	6.396356000000	-0.710287000000
C	2.053217000000	6.155453000000	-1.480398000000
C	1.524453000000	4.863762000000	-1.727893000000
H	1.570972000000	7.033918000000	-1.948371000000
H	0.631337000000	4.750095000000	-2.358406000000
C	5.078527000000	3.200004000000	1.151016000000
C	3.982155000000	2.911993000000	0.349483000000
C	5.510023000000	4.539833000000	1.339935000000
H	3.684063000000	1.860469000000	0.228590000000
H	6.379688000000	4.750600000000	1.981176000000
C	4.837963000000	5.578088000000	0.715022000000
H	5.140722000000	6.628645000000	0.832971000000
H	5.615820000000	2.374852000000	1.644134000000
C	1.457780000000	-0.263426000000	-3.619839000000
C	1.111993000000	0.293723000000	-5.043732000000
C	1.869771000000	1.631314000000	-5.275126000000
C	3.359298000000	1.396644000000	-5.647488000000
C	4.003059000000	0.163556000000	-4.998958000000
C	2.344365000000	1.909591000000	-2.787211000000
C	1.697453000000	2.530902000000	-4.044141000000
H	0.555148000000	-0.242178000000	-2.982067000000
H	1.454983000000	-0.429904000000	-5.812397000000
H	1.386762000000	2.144239000000	-6.132016000000
H	3.441195000000	1.281638000000	-6.748539000000
H	3.939378000000	2.312012000000	-5.396983000000
H	5.079216000000	0.111674000000	-5.268388000000
H	3.367619000000	2.316433000000	-2.679353000000
H	2.136260000000	3.530502000000	-4.230926000000
H	0.616475000000	2.700238000000	-3.863997000000
C	3.883475000000	0.093428000000	-3.471681000000
H	4.615884000000	0.767157000000	-2.983081000000
H	3.551453000000	-0.751379000000	-5.437699000000
N	2.549894000000	0.436338000000	-2.917470000000
C	-0.378800000000	0.434221000000	-5.201715000000
C	-1.118239000000	-0.203116000000	-6.124913000000
H	-0.657690000000	-0.892365000000	-6.852871000000
H	-2.209028000000	-0.065948000000	-6.184945000000

H -0.889472000000 1.095413000000 -4.477506000000
H 4.133267000000 -0.934623000000 -3.130029000000
H 1.731108000000 -1.334320000000 -3.691122000000
C -7.530010000000 -2.209185000000 -0.356044000000
H -7.722104000000 -3.069062000000 0.320359000000
H -7.907787000000 -1.302193000000 0.154376000000
H -8.121575000000 -2.367579000000 -1.277780000000

TS*re-SR*

Electronic energy = -4234.135358 Hartree

C 3.231571000000 -1.368987000000 0.616129000000
O 3.203927000000 -0.112564000000 0.318276000000
C 4.386783000000 -1.860411000000 1.308226000000
C 2.120684000000 -2.211479000000 0.305863000000
H 2.706841000000 0.086153000000 -1.057563000000
H 1.360197000000 -1.686926000000 -0.297578000000
C 2.126246000000 -3.670062000000 0.124878000000
C 1.294847000000 -4.206936000000 -0.891038000000
C 2.841614000000 -4.574826000000 0.948087000000
H 0.697472000000 -3.532218000000 -1.523977000000
H 3.457932000000 -4.188546000000 1.771246000000
C 1.194151000000 -5.591195000000 -1.085838000000
C 2.740103000000 -5.956023000000 0.747765000000
H 0.524676000000 -5.978108000000 -1.868656000000
H 3.294659000000 -6.641929000000 1.407011000000
C 1.916567000000 -6.472423000000 -0.268757000000
H 1.828516000000 -7.560170000000 -0.411185000000
C 5.413207000000 -2.242860000000 1.876566000000
C 6.575080000000 -2.734746000000 2.532338000000
C 6.811622000000 -4.133010000000 2.611156000000
C 7.512915000000 -1.844593000000 3.117987000000
H 6.089621000000 -4.823105000000 2.150347000000
H 7.330811000000 -0.761900000000 3.057288000000
C 7.951697000000 -4.619233000000 3.259076000000
C 8.647913000000 -2.343358000000 3.765775000000
H 8.126179000000 -5.704410000000 3.313153000000
H 9.367277000000 -1.644731000000 4.219006000000
C 8.871821000000 -3.728863000000 3.838798000000
H 9.766874000000 -4.116049000000 4.348559000000
C -2.914321000000 -5.979035000000 -0.329420000000
C -2.592569000000 -4.632618000000 -0.524547000000
C -2.362805000000 -6.721776000000 0.742012000000
H -3.008222000000 -4.082879000000 -1.375997000000
H -2.636273000000 -7.780724000000 0.864443000000
C -1.698901000000 -4.011740000000 0.378764000000
C -1.462706000000 -6.137069000000 1.641669000000
H -1.004634000000 -6.698152000000 2.467717000000
C -1.144916000000 -4.792591000000 1.428539000000
H -3.607768000000 -6.472434000000 -1.026446000000
C -0.232647000000 -2.787293000000 1.645708000000
C -1.092446000000 -2.690390000000 0.512299000000
O -0.262157000000 -4.075337000000 2.171330000000
C 0.828329000000 -1.955810000000 2.096738000000
H 1.527900000000 -2.493951000000 2.759018000000
C 0.739152000000 -0.514168000000 2.355485000000
C -0.467811000000 0.212792000000 2.195040000000
C 1.862170000000 0.164915000000 2.891918000000
H -1.352853000000 -0.298655000000 1.797878000000
H 2.796696000000 -0.389636000000 3.062258000000
C -0.540226000000 1.560027000000 2.554384000000
C 1.795715000000 1.525851000000 3.214525000000
H -1.489278000000 2.103725000000 2.448311000000
H 2.686833000000 2.039559000000 3.605003000000
C 0.596367000000 2.231265000000 3.038621000000
H 0.540196000000 3.303642000000 3.274894000000
N -1.139816000000 -1.545142000000 -0.225860000000
S -1.799354000000 -1.476195000000 -1.695737000000
O -1.369139000000 -2.537091000000 -2.653494000000
O -1.545878000000 -0.059514000000 -2.186224000000
C -3.587385000000 -1.587847000000 -1.520114000000
C -4.348261000000 -1.988797000000 -2.629410000000
C -4.199250000000 -1.270235000000 -0.299072000000
H -3.840798000000 -2.255096000000 -3.568269000000
H -3.585787000000 -0.964466000000 0.560581000000
C -5.740562000000 -2.065985000000 -2.504473000000
C -5.592416000000 -1.363349000000 -0.190971000000
H -6.343724000000 -2.381653000000 -3.370291000000
H -6.076406000000 -1.111450000000 0.764121000000

C	-6.387145000000	-1.756100000000	-1.288275000000
C	-0.836597000000	2.722770000000	-0.425869000000
O	-0.542389000000	3.643539000000	0.335855000000
N	-2.128912000000	2.222698000000	-0.599951000000
N	0.073175000000	2.085845000000	-1.255909000000
H	-0.246755000000	1.177259000000	-1.630410000000
H	-2.211732000000	1.409726000000	-1.234750000000
C	-3.298742000000	2.708882000000	-0.029851000000
C	-4.530889000000	2.177526000000	-0.487763000000
C	-3.321167000000	3.703358000000	0.974992000000
H	-4.535973000000	1.419797000000	-1.283766000000
H	-2.370977000000	4.139004000000	1.306506000000
C	-5.740021000000	2.623208000000	0.055375000000
C	-4.546391000000	4.124395000000	1.513563000000
C	-5.765233000000	3.598684000000	1.068172000000
H	-6.714852000000	3.949801000000	1.491166000000
C	-4.513995000000	5.185848000000	2.589835000000
F	-4.048075000000	6.364717000000	2.110979000000
F	-5.740125000000	5.419229000000	3.115933000000
F	-3.696655000000	4.825283000000	3.610662000000
C	-7.048245000000	2.044820000000	-0.424232000000
F	-7.541333000000	1.121667000000	0.451832000000
F	-8.001359000000	2.996677000000	-0.553382000000
F	-6.930231000000	1.421209000000	-1.622956000000
C	1.500662000000	2.299016000000	-1.101392000000
H	1.862583000000	1.777642000000	-0.190396000000
C	1.887302000000	3.769602000000	-0.990809000000
C	3.017464000000	4.155224000000	-0.195088000000
C	3.396540000000	5.551386000000	-0.217534000000
N	2.741846000000	6.492108000000	-0.960970000000
C	1.712028000000	6.087940000000	-1.688091000000
C	1.251197000000	4.747838000000	-1.743344000000
H	1.191362000000	6.861921000000	-2.282129000000
H	0.375283000000	4.491566000000	-2.356901000000
C	4.866009000000	3.706881000000	1.344917000000
C	3.784147000000	3.250673000000	0.603588000000
C	5.231497000000	5.079162000000	1.330402000000
H	3.533707000000	2.180314000000	0.646633000000
H	6.088161000000	5.423967000000	1.929550000000
C	4.509925000000	5.980686000000	0.564509000000
H	4.760591000000	7.050647000000	0.525758000000
H	5.440476000000	2.993058000000	1.955273000000
C	1.785399000000	-0.658654000000	-2.865626000000
C	1.699598000000	-0.418631000000	-4.398292000000
C	2.218308000000	1.011856000000	-4.734892000000
C	3.767579000000	1.051716000000	-4.851254000000
C	4.499731000000	0.026911000000	-3.972459000000
C	2.315413000000	1.750467000000	-2.316413000000
C	1.678547000000	1.995458000000	-3.688515000000
H	0.787887000000	-0.535460000000	-2.403357000000
H	2.353501000000	-1.146583000000	-4.919374000000
H	1.792003000000	1.293946000000	-5.717652000000
H	4.058127000000	0.853358000000	-5.903208000000
H	4.123783000000	2.081291000000	-4.630402000000
H	5.596743000000	0.164271000000	-4.065868000000
H	3.281997000000	2.283524000000	-2.266214000000
H	1.880765000000	3.043233000000	-3.984169000000
H	0.577014000000	1.899105000000	-3.617266000000
C	4.162682000000	0.057674000000	-2.482513000000
H	4.713795000000	0.852400000000	-1.943528000000
H	4.299375000000	-0.991115000000	-4.362957000000
N	2.709943000000	0.286941000000	-2.158336000000
C	0.294384000000	-0.660868000000	-4.893097000000
C	-0.000851000000	-1.433657000000	-5.950949000000
H	0.783414000000	-1.962504000000	-6.519553000000
H	-1.041032000000	-1.578409000000	-6.280132000000
H	-0.522137000000	-0.186227000000	-4.321319000000
H	4.438912000000	-0.900001000000	-1.999240000000
H	2.117569000000	-1.687067000000	-2.629329000000
C	-7.887246000000	-1.805542000000	-1.174553000000
H	-8.210065000000	-2.090049000000	-0.154208000000
H	-8.315940000000	-0.802890000000	-1.381360000000
H	-8.330816000000	-2.517836000000	-1.897037000000

IntA$_{re\text{-}SR}$

Electronic energy = -4234.1614 Hartree

C	3.051725000000	-1.174593000000	0.597928000000
O	3.536817000000	-0.457748000000	-0.309153000000

C	3.584666000000	-1.134533000000	1.918073000000
C	1.907632000000	-2.117816000000	0.268161000000
H	2.810805000000	-0.347512000000	-1.823132000000
H	1.183007000000	-1.524038000000	-0.341403000000
C	2.447614000000	-3.241321000000	-0.618918000000
C	1.684006000000	-3.722684000000	-1.703912000000
C	3.688501000000	-3.850656000000	-0.340902000000
H	0.709379000000	-3.268757000000	-1.932140000000
H	4.290530000000	-3.503648000000	0.514064000000
C	2.160359000000	-4.780873000000	-2.494667000000
C	4.166857000000	-4.903059000000	-1.137146000000
H	1.543013000000	-5.147338000000	-3.329056000000
H	5.138763000000	-5.364422000000	-0.904393000000
C	3.403481000000	-5.371255000000	-2.218183000000
H	3.773485000000	-6.201978000000	-2.838252000000
C	3.931565000000	-1.044775000000	3.099229000000
C	4.237926000000	-0.939957000000	4.479691000000
C	3.223226000000	-1.224032000000	5.432805000000
C	5.522992000000	-0.531256000000	4.921535000000
H	2.223689000000	-1.518522000000	5.079883000000
H	6.300601000000	-0.305090000000	4.177977000000
C	3.499481000000	-1.097136000000	6.797266000000
C	5.787062000000	-0.423725000000	6.290140000000
H	2.707934000000	-1.305547000000	7.532396000000
H	6.785606000000	-0.112168000000	6.631119000000
C	4.778110000000	-0.703662000000	7.228583000000
H	4.989810000000	-0.609791000000	8.304504000000
C	-3.436016000000	-5.878637000000	-1.100705000000
C	-2.868591000000	-4.604903000000	-1.223623000000
C	-2.980547000000	-6.803902000000	-0.133019000000
H	-3.213325000000	-3.915828000000	-2.003299000000
H	-3.451421000000	-7.796096000000	-0.062985000000
C	-1.818349000000	-4.240153000000	-0.349731000000
C	-1.925837000000	-6.478438000000	0.731409000000
H	-1.540612000000	-7.183498000000	1.481648000000
C	-1.367962000000	-5.205009000000	0.593509000000
H	-4.252807000000	-6.167893000000	-1.779209000000
C	-0.082612000000	-3.438912000000	0.862439000000
C	-0.974313000000	-3.063123000000	-0.134560000000
O	-0.317315000000	-4.721671000000	1.321353000000
C	1.074426000000	-2.690227000000	1.450051000000
H	1.698028000000	-3.430406000000	1.994974000000
C	0.554056000000	-1.671693000000	2.466406000000
C	0.024407000000	-2.159133000000	3.681083000000
C	0.532603000000	-0.285491000000	2.237030000000
H	0.012917000000	-3.245915000000	3.861602000000
H	0.918719000000	0.137134000000	1.299514000000
C	-0.504866000000	-1.282804000000	4.639976000000
C	0.027443000000	0.595717000000	3.205938000000
H	-0.922918000000	-1.686323000000	5.575401000000
H	0.049581000000	1.674180000000	3.005027000000
C	-0.499903000000	0.103763000000	4.407931000000
H	-0.913353000000	0.803428000000	5.149258000000
N	-0.911324000000	-1.791107000000	-0.708589000000
S	-1.791766000000	-1.398643000000	-1.973927000000
O	-1.722158000000	-2.296776000000	-3.168839000000
O	-1.444636000000	0.057220000000	-2.304335000000
C	-3.522024000000	-1.346706000000	-1.463197000000
C	-4.526497000000	-1.360658000000	-2.442501000000
C	-3.843269000000	-1.315781000000	-0.097945000000
H	-4.247388000000	-1.407519000000	-3.505281000000
H	-3.038211000000	-1.306219000000	0.651426000000
C	-5.868323000000	-1.335466000000	-2.041286000000
C	-5.190008000000	-1.308990000000	0.284143000000
H	-6.662507000000	-1.338287000000	-2.804633000000
H	-5.446361000000	-1.277946000000	1.353507000000
C	-6.223942000000	-1.316004000000	-0.675750000000
C	-0.047110000000	2.181509000000	-0.199292000000
O	0.585116000000	2.799189000000	0.663370000000
N	-1.410810000000	1.910656000000	-0.160817000000
N	0.549559000000	1.720246000000	-1.360721000000
H	0.018430000000	1.017984000000	-1.910873000000
H	-1.749590000000	1.280420000000	-0.912267000000
C	-2.325442000000	2.344732000000	0.791736000000
C	-3.693610000000	2.028711000000	0.599606000000
C	-1.960602000000	3.105098000000	1.925300000000
H	-4.005883000000	1.469116000000	-0.293000000000
H	-0.912493000000	3.402727000000	2.045151000000

C	-4.651817000000	2.437691000000	1.533655000000
C	-2.932809000000	3.471124000000	2.866753000000
C	-4.283503000000	3.148476000000	2.689819000000
H	-5.036531000000	3.461419000000	3.423919000000
C	-2.469405000000	4.203598000000	4.103216000000
F	-1.732968000000	5.296787000000	3.794609000000
F	-3.499833000000	4.610142000000	4.880206000000
F	-1.676076000000	3.408121000000	4.876937000000
C	-6.109454000000	2.106342000000	1.325504000000
F	-6.495026000000	1.036102000000	2.082137000000
F	-6.911623000000	3.135970000000	1.687611000000
F	-6.393666000000	1.800627000000	0.038400000000
C	1.956096000000	1.984752000000	-1.560254000000
H	2.546832000000	1.524619000000	-0.740199000000
C	2.294645000000	3.474387000000	-1.556539000000
C	3.614744000000	3.911767000000	-1.202999000000
C	3.907877000000	5.319800000000	-1.351855000000
N	2.987086000000	6.231771000000	-1.784923000000
C	1.774001000000	5.787719000000	-2.072299000000
C	1.381139000000	4.426106000000	-1.984964000000
H	1.038157000000	6.540876000000	-2.409973000000
H	0.356561000000	4.125353000000	-2.249531000000
C	5.905397000000	3.545263000000	-0.419784000000
C	4.644273000000	3.046759000000	-0.718187000000
C	6.197278000000	4.925228000000	-0.584778000000
H	4.441969000000	1.978609000000	-0.545015000000
H	7.201573000000	5.304165000000	-0.341503000000
C	5.215817000000	5.793470000000	-1.035435000000
H	5.398289000000	6.870484000000	-1.161153000000
H	6.681430000000	2.865131000000	-0.036789000000
C	1.657396000000	-0.893066000000	-3.516948000000
C	1.425901000000	-0.538979000000	-5.007869000000
C	2.036705000000	0.859719000000	-5.311486000000
C	3.564197000000	0.772641000000	-5.590409000000
C	4.289521000000	-0.375890000000	-4.868117000000
C	2.496303000000	1.426209000000	-2.899666000000
C	1.695119000000	1.800198000000	-4.148041000000
H	0.737377000000	-0.716749000000	-2.928473000000
H	1.942755000000	-1.281990000000	-5.648074000000
H	1.541305000000	1.247416000000	-6.222924000000
H	3.721487000000	0.622824000000	-6.677889000000
H	4.039996000000	1.748884000000	-5.352533000000
H	5.376996000000	-0.325458000000	-5.081719000000
H	3.510281000000	1.856486000000	-2.989977000000
H	1.923624000000	2.854047000000	-4.399082000000
H	0.606687000000	1.754969000000	-3.942558000000
C	4.127912000000	-0.433463000000	-3.349639000000
H	4.811654000000	0.263940000000	-2.828069000000
H	3.952831000000	-1.340169000000	-5.298438000000
N	2.745508000000	-0.084625000000	-2.858559000000
C	-0.045409000000	-0.616810000000	-5.341024000000
C	-0.527920000000	-1.233305000000	-6.431702000000
H	0.134093000000	-1.747194000000	-7.150041000000
H	-1.608744000000	-1.264247000000	-6.635662000000
H	-0.743512000000	-0.157273000000	-4.619715000000
H	4.342589000000	-1.449587000000	-2.963264000000
H	1.922666000000	-1.955694000000	-3.376030000000
C	-7.666351000000	-1.315959000000	-0.241807000000
H	-7.963464000000	-2.315137000000	0.142404000000
H	-7.831886000000	-0.589035000000	0.576960000000
H	-8.346039000000	-1.063452000000	-1.078351000000

RC$_{D\text{-}SS}$

Electronic energy = -2681.034979 Hartree

C	-0.976231000000	-0.898914000000	-0.368026000000
O	-0.952044000000	-1.337710000000	0.792509000000
C	-1.700661000000	0.294463000000	-0.674739000000
C	-0.266573000000	-1.630469000000	-1.521126000000
H	0.791279000000	-1.848485000000	1.133317000000
H	-0.927820000000	-1.461223000000	-2.399148000000
C	-0.224617000000	-3.134823000000	-1.279169000000
C	0.951775000000	-3.905261000000	-1.315971000000
C	-1.449007000000	-3.798007000000	-1.043129000000
H	1.923988000000	-3.429560000000	-1.501249000000
H	-2.383337000000	-3.216170000000	-1.025621000000
C	0.906683000000	-5.294183000000	-1.104781000000
C	-1.497057000000	-5.180064000000	-0.830655000000
H	1.841542000000	-5.873989000000	-1.132486000000

H	-2.465605000000	-5.669353000000	-0.645108000000
C	-0.313159000000	-5.936365000000	-0.856120000000
H	-0.344282000000	-7.023008000000	-0.685387000000
C	-2.328418000000	1.346201000000	-0.825092000000
C	-3.016967000000	2.580879000000	-0.951320000000
C	-2.830453000000	3.391318000000	-2.102559000000
C	-3.866565000000	3.023313000000	0.100612000000
H	-2.162753000000	3.039125000000	-2.901318000000
H	-4.009347000000	2.376287000000	0.994794000000
C	-3.473867000000	4.629749000000	-2.192257000000
C	-4.507110000000	4.262678000000	-0.015275000000
H	-3.321989000000	5.260542000000	-3.081122000000
H	-5.167103000000	4.605954000000	0.796105000000
C	-4.312184000000	5.067649000000	-1.151114000000
H	-4.817252000000	6.042870000000	-1.228004000000
C	6.393554000000	-1.544118000000	0.967708000000
C	5.042395000000	-1.582494000000	1.331650000000
C	6.797782000000	-1.270761000000	-0.360578000000
H	4.726927000000	-1.774356000000	2.364442000000
H	7.870012000000	-1.242342000000	-0.606551000000
C	4.076843000000	-1.338097000000	0.331351000000
C	5.855120000000	-1.042843000000	-1.372847000000
H	6.142592000000	-0.842769000000	-2.414394000000
C	4.511294000000	-1.093625000000	-0.991380000000
H	7.161065000000	-1.729709000000	1.734040000000
C	2.286766000000	-1.073937000000	-1.057900000000
C	2.619434000000	-1.310788000000	0.272958000000
O	3.438618000000	-0.955710000000	-1.823630000000
C	1.080178000000	-0.975012000000	-1.958556000000
H	1.377217000000	-1.570421000000	-2.850380000000
C	0.882322000000	0.452258000000	-2.478116000000
C	1.209953000000	1.576131000000	-1.699977000000
C	0.361169000000	0.649006000000	-3.771562000000
H	1.620919000000	1.436879000000	-0.689175000000
H	0.114016000000	-0.225224000000	-4.396242000000
C	1.025467000000	2.870388000000	-2.207559000000
C	0.171001000000	1.941716000000	-4.280403000000
H	1.293997000000	3.737101000000	-1.586593000000
H	-0.227201000000	2.076647000000	-5.297769000000
C	0.508307000000	3.059061000000	-3.498504000000
H	0.373478000000	4.075831000000	-3.898112000000
N	1.772774000000	-1.592864000000	1.356088000000
S	1.738387000000	-0.582885000000	2.749696000000
O	3.107155000000	-0.509905000000	3.296787000000
O	0.613278000000	-1.108204000000	3.545481000000
C	1.323955000000	1.069418000000	2.175350000000
C	2.352626000000	1.981728000000	1.893056000000
C	-0.024854000000	1.424311000000	2.022717000000
H	3.400383000000	1.682394000000	2.038944000000
H	-0.812327000000	0.686793000000	2.227384000000
C	2.012425000000	3.267442000000	1.445804000000
C	-0.342411000000	2.714151000000	1.587905000000
H	2.813700000000	3.987878000000	1.217503000000
H	-1.399953000000	2.993861000000	1.469380000000
C	0.665203000000	3.655658000000	1.288250000000
C	-4.174411000000	-0.329083000000	2.206574000000
C	-3.588866000000	-2.786895000000	2.423124000000
H	-2.752852000000	-2.912307000000	1.694184000000
H	-3.301192000000	-3.389031000000	3.308172000000
C	-4.895819000000	-3.354665000000	1.857907000000
H	-5.731984000000	-2.958184000000	2.471706000000
H	-4.898707000000	-4.453239000000	2.015328000000
C	-5.122767000000	-3.052163000000	0.369554000000
H	-6.200453000000	-3.180597000000	0.130749000000
H	-4.585893000000	-3.800169000000	-0.254044000000
C	-4.667300000000	-1.641541000000	-0.018830000000
H	-5.081661000000	-1.378065000000	-1.013949000000
H	-3.566263000000	-1.616061000000	-0.140307000000
C	-5.071567000000	-0.562832000000	0.994437000000
H	-6.101831000000	-0.767395000000	1.364357000000
H	-5.129530000000	0.424232000000	0.500807000000
C	-2.748430000000	-1.114723000000	4.023227000000
H	-2.761665000000	-1.992075000000	4.703070000000
H	-1.698327000000	-1.012270000000	3.666667000000
C	-3.199317000000	0.138461000000	4.762630000000
H	-2.476643000000	0.384305000000	5.566872000000
H	-4.181720000000	-0.053591000000	5.244951000000
C	-3.330094000000	1.285940000000	3.761169000000

H	-2.319243000000	1.680337000000	3.507056000000
H	-3.862558000000	2.145356000000	4.227585000000
N	-3.654482000000	-1.404721000000	2.906110000000
N	-4.031921000000	0.924830000000	2.542720000000
C	0.289234000000	5.035506000000	0.813650000000
H	1.167375000000	5.597773000000	0.441124000000
H	-0.466525000000	4.984231000000	0.002918000000
H	-0.164524000000	5.628195000000	1.636044000000

TS1*D-SS*

Electronic energy = -2681.015603 Hartree

C	-0.627851000000	-0.544603000000	-0.021068000000
O	-0.333842000000	-0.889059000000	1.152809000000
C	-1.428181000000	0.583447000000	-0.272033000000
C	-0.096537000000	-1.326064000000	-1.235999000000
H	1.329680000000	-1.371454000000	1.226871000000
H	-0.884704000000	-1.202708000000	-2.012925000000
C	0.040515000000	-2.822032000000	-0.969482000000
C	1.081598000000	-3.601730000000	-1.511436000000
C	-0.944671000000	-3.480077000000	-0.201324000000
H	1.876758000000	-3.136969000000	-2.109814000000
H	-1.758981000000	-2.892680000000	0.243284000000
C	1.134603000000	-4.988512000000	-1.294161000000
C	-0.894368000000	-4.863104000000	0.016671000000
H	1.963841000000	-5.570061000000	-1.724620000000
H	-1.670006000000	-5.347832000000	0.630174000000
C	0.149510000000	-5.626779000000	-0.528979000000
H	0.197815000000	-6.711818000000	-0.352049000000
C	-2.440922000000	1.336004000000	-0.159346000000
C	-3.005702000000	2.644135000000	-0.398272000000
C	-2.161945000000	3.606288000000	-1.014526000000
C	-4.325439000000	3.016115000000	-0.057852000000
H	-1.141544000000	3.314301000000	-1.297183000000
H	-4.971981000000	2.254779000000	0.399948000000
C	-2.628995000000	4.901171000000	-1.260479000000
C	-4.784340000000	4.315892000000	-0.311030000000
H	-1.961074000000	5.632465000000	-1.741862000000
H	-5.815492000000	4.589130000000	-0.039443000000
C	-3.940819000000	5.265400000000	-0.908659000000
H	-4.305897000000	6.284512000000	-1.107049000000
C	6.834527000000	-1.658986000000	0.123603000000
C	5.570059000000	-1.531227000000	0.710468000000
C	7.024979000000	-1.507017000000	-1.270495000000
H	5.421471000000	-1.625415000000	1.793241000000
H	8.035588000000	-1.607791000000	-1.694421000000
C	4.472840000000	-1.243051000000	-0.129063000000
C	5.946317000000	-1.235732000000	-2.124128000000
H	6.066375000000	-1.125719000000	-3.210997000000
C	4.690769000000	-1.119905000000	-1.520262000000
H	7.703041000000	-1.880698000000	0.761996000000
C	2.500544000000	-0.866530000000	-1.210731000000
C	3.037206000000	-1.066498000000	0.058141000000
O	3.507603000000	-0.912462000000	-2.168427000000
C	1.166229000000	-0.671684000000	-1.886082000000
H	1.290482000000	-1.229621000000	-2.840296000000
C	0.940074000000	0.777095000000	-2.329289000000
C	1.581724000000	1.858141000000	-1.701394000000
C	0.110649000000	1.036483000000	-3.439424000000
H	2.226624000000	1.674659000000	-0.829589000000
H	-0.392652000000	0.198266000000	-3.949120000000
C	1.420047000000	3.164770000000	-2.188325000000
C	-0.065686000000	2.341433000000	-3.918825000000
H	1.946896000000	3.992625000000	-1.691343000000
H	-0.713862000000	2.522460000000	-4.789758000000
C	0.600972000000	3.412346000000	-3.300252000000
H	0.479992000000	4.436348000000	-3.685589000000
N	2.363178000000	-1.213326000000	1.278054000000
S	2.639433000000	-0.170516000000	2.611310000000
O	4.095237000000	-0.109475000000	2.858444000000
O	1.690457000000	-0.638131000000	3.637033000000
C	2.136889000000	1.477609000000	2.088206000000
C	3.108007000000	2.388780000000	1.649261000000
C	0.777435000000	1.826916000000	2.130163000000
H	4.166290000000	2.090617000000	1.645236000000
H	0.044063000000	1.084545000000	2.473511000000
C	2.701102000000	3.669323000000	1.244403000000
C	0.390588000000	3.103578000000	1.711449000000
H	3.458135000000	4.393295000000	0.903257000000

H	-0.676431000000	3.376134000000	1.725165000000
C	1.342793000000	4.047296000000	1.266245000000
C	-4.186591000000	-0.882416000000	0.447409000000
C	-4.624640000000	-3.362575000000	0.853555000000
H	-3.817272000000	-4.070753000000	0.551395000000
H	-5.023579000000	-3.747339000000	1.816932000000
C	-5.747700000000	-3.396189000000	-0.179692000000
H	-6.483264000000	-2.601466000000	0.067874000000
H	-6.289921000000	-4.354844000000	-0.044761000000
C	-5.277860000000	-3.272264000000	-1.633334000000
H	-6.165628000000	-3.113231000000	-2.282055000000
H	-4.819934000000	-4.228631000000	-1.963447000000
C	-4.274339000000	-2.126956000000	-1.826241000000
H	-4.219668000000	-1.853614000000	-2.899468000000
H	-3.253152000000	-2.465991000000	-1.551133000000
C	-4.628449000000	-0.881749000000	-1.007089000000
H	-5.721474000000	-0.680242000000	-1.052888000000
H	-4.151698000000	0.017445000000	-1.441397000000
C	-3.302524000000	-1.968182000000	2.430258000000
H	-3.483274000000	-2.905879000000	2.988813000000
H	-2.208353000000	-1.889508000000	2.232377000000
C	-3.770905000000	-0.742402000000	3.209078000000
H	-3.232408000000	-0.661784000000	4.174260000000
H	-4.854495000000	-0.838565000000	3.435283000000
C	-3.492346000000	0.478608000000	2.337778000000
H	-2.406641000000	0.713675000000	2.368037000000
H	-4.018979000000	1.378768000000	2.722883000000
N	-4.029530000000	-2.051519000000	1.152968000000
N	-3.872919000000	0.292040000000	0.944596000000
C	0.903188000000	5.423210000000	0.837057000000
H	1.733642000000	5.999259000000	0.385480000000
H	0.074114000000	5.367102000000	0.102795000000
H	0.520483000000	6.005559000000	1.701363000000

$I_{D\text{-}SS}$

Electronic energy = -2681.03265 Hartree

C	-0.725406000000	0.986499000000	-0.506114000000
O	-0.123509000000	1.644451000000	-1.419934000000
C	-1.101339000000	-0.341699000000	-0.639130000000
C	-0.922356000000	1.637153000000	0.885653000000
H	1.328809000000	0.838929000000	-1.175313000000
H	-1.944912000000	1.371222000000	1.222675000000
C	-0.866510000000	3.143119000000	0.803911000000
C	0.316732000000	3.834313000000	0.468220000000
C	-2.037283000000	3.893220000000	1.036572000000
H	1.234190000000	3.267336000000	0.263343000000
H	-2.968385000000	3.361615000000	1.297165000000
C	0.323740000000	5.234026000000	0.385397000000
C	-2.035113000000	5.294374000000	0.948111000000
H	1.256838000000	5.756908000000	0.125061000000
H	-2.960344000000	5.859688000000	1.140577000000
C	-0.849128000000	5.969809000000	0.622534000000
H	-0.837841000000	7.068465000000	0.554343000000
C	-2.192180000000	-0.980290000000	-1.068894000000
C	-2.419533000000	-2.421769000000	-1.244106000000
C	-1.344355000000	-3.319977000000	-1.036503000000
C	-3.678093000000	-2.949962000000	-1.612382000000
H	-0.352026000000	-2.911999000000	-0.795821000000
H	-4.519915000000	-2.265773000000	-1.803340000000
C	-1.539177000000	-4.698190000000	-1.168046000000
C	-3.866498000000	-4.334136000000	-1.747053000000
H	-0.689525000000	-5.379432000000	-1.006066000000
H	-4.854850000000	-4.725041000000	-2.034970000000
C	-2.800185000000	-5.216025000000	-1.517885000000
H	-2.946239000000	-6.301965000000	-1.622016000000
C	6.085926000000	0.993523000000	1.536116000000
C	5.009737000000	0.751918000000	0.673346000000
C	5.883770000000	1.303462000000	2.902181000000
H	5.175687000000	0.526776000000	-0.388385000000
H	6.753696000000	1.483631000000	3.551649000000
C	3.703811000000	0.816497000000	1.202954000000
C	4.592489000000	1.398338000000	3.442065000000
H	4.411282000000	1.655911000000	4.494968000000
C	3.532888000000	1.155325000000	2.563326000000
H	7.112602000000	0.944759000000	1.142647000000
C	1.483285000000	0.929118000000	1.713576000000
C	2.362329000000	0.650267000000	0.664783000000
O	2.203850000000	1.240019000000	2.857921000000

C	0.002903000000	1.020188000000	1.996160000000
H	-0.048561000000	1.746044000000	2.834871000000
C	-0.620706000000	-0.266916000000	2.534293000000
C	-0.292584000000	-1.538269000000	2.030012000000
C	-1.636241000000	-0.160531000000	3.507564000000
H	0.510725000000	-1.648892000000	1.288872000000
H	-1.887373000000	0.831330000000	3.919094000000
C	-0.992242000000	-2.674576000000	2.471068000000
C	-2.328908000000	-1.296186000000	3.953530000000
H	-0.733454000000	-3.658880000000	2.052910000000
H	-3.108480000000	-1.195948000000	4.725316000000
C	-2.015559000000	-2.559806000000	3.423859000000
H	-2.559127000000	-3.454115000000	3.765514000000
N	2.116997000000	0.333043000000	-0.686322000000
S	2.191845000000	-1.271455000000	-1.237408000000
O	1.428344000000	-1.292369000000	-2.501697000000
O	1.918084000000	-2.244144000000	-0.144712000000
C	3.931068000000	-1.476882000000	-1.639758000000
C	4.743234000000	-2.272750000000	-0.824802000000
C	4.454962000000	-0.799994000000	-2.753132000000
H	4.297016000000	-2.787644000000	0.037426000000
H	3.789784000000	-0.192743000000	-3.384116000000
C	6.111053000000	-2.373357000000	-1.119912000000
C	5.821291000000	-0.907543000000	-3.028671000000
H	6.758596000000	-2.990041000000	-0.477117000000
H	6.242094000000	-0.374115000000	-3.895936000000
C	6.674187000000	-1.689685000000	-2.214938000000
C	-4.190164000000	0.403679000000	-0.628614000000
C	-6.071316000000	2.053315000000	-0.274101000000
H	-5.859333000000	2.897365000000	0.422458000000
H	-6.657686000000	2.486617000000	-1.111836000000
C	-6.922271000000	0.989778000000	0.415086000000
H	-6.973245000000	0.098916000000	-0.247372000000
H	-7.960224000000	1.377122000000	0.472238000000
C	-6.429701000000	0.602505000000	1.813860000000
H	-6.933337000000	-0.336517000000	2.126602000000
H	-6.734167000000	1.375990000000	2.550391000000
C	-4.909915000000	0.421669000000	1.855999000000
H	-4.606592000000	-0.104506000000	2.781908000000
H	-4.401894000000	1.409011000000	1.903099000000
C	-4.359680000000	-0.362078000000	0.665004000000
H	-4.958688000000	-1.278741000000	0.471945000000
H	-3.345371000000	-0.744996000000	0.906595000000
C	-4.262531000000	2.435848000000	-1.947653000000
H	-4.974190000000	3.263165000000	-2.122629000000
H	-3.301238000000	2.883032000000	-1.609968000000
C	-4.048305000000	1.584050000000	-3.194931000000
H	-3.657613000000	2.194948000000	-4.031999000000
H	-5.019202000000	1.152383000000	-3.517867000000
C	-3.039988000000	0.505232000000	-2.834910000000
H	-2.007029000000	0.915988000000	-2.810934000000
H	-3.041912000000	-0.336355000000	-3.557782000000
N	-4.807846000000	1.589787000000	-0.873117000000
N	-3.297099000000	-0.073230000000	-1.502835000000
C	8.146326000000	-1.783919000000	-2.524552000000
H	8.317823000000	-2.206933000000	-3.536461000000
H	8.623868000000	-0.781902000000	-2.512695000000
H	8.678298000000	-2.422978000000	-1.794058000000

II*D-SS*

Electronic energy = -2681.039623 Hartree

C	2.107959000000	3.712938000000	2.869891000000
O	1.525311000000	4.046313000000	4.072607000000
C	3.319428000000	4.113979000000	2.507674000000
C	1.219390000000	2.896976000000	1.953680000000
H	2.217783000000	4.427390000000	4.646103000000
H	1.822883000000	2.701541000000	1.049446000000
C	0.028089000000	3.733301000000	1.508198000000
C	-0.949388000000	4.217316000000	2.401015000000
C	-0.082921000000	4.051801000000	0.137575000000
H	-0.884633000000	3.962380000000	3.467943000000
H	0.644712000000	3.608371000000	-0.563159000000
C	-1.995371000000	5.030114000000	1.937904000000
C	-1.127962000000	4.867993000000	-0.323220000000
H	-2.753281000000	5.399323000000	2.646270000000
H	-1.207264000000	5.098219000000	-1.397358000000
C	-2.082287000000	5.368794000000	0.577240000000
H	-2.905933000000	6.004688000000	0.217313000000

C	4.478744000000	4.504653000000	1.989838000000
C	5.803065000000	3.896167000000	2.228167000000
C	5.895074000000	2.600243000000	2.782949000000
C	6.988719000000	4.591450000000	1.906416000000
H	4.974077000000	2.050073000000	3.016603000000
H	6.928051000000	5.604495000000	1.479751000000
C	7.145590000000	2.013579000000	2.998715000000
C	8.241456000000	4.002205000000	2.130657000000
H	7.196616000000	0.992570000000	3.406041000000
H	9.158633000000	4.554529000000	1.875563000000
C	8.322931000000	2.710415000000	2.672898000000
H	9.305605000000	2.242869000000	2.837906000000
C	5.623770000000	-2.313523000000	2.388870000000
C	5.224315000000	-1.127367000000	1.758059000000
C	4.815137000000	-2.926374000000	3.376946000000
H	5.835422000000	-0.639857000000	0.984600000000
H	5.155187000000	-3.859599000000	3.851473000000
C	3.990675000000	-0.562014000000	2.129882000000
C	3.585883000000	-2.368649000000	3.766799000000
H	2.949833000000	-2.832173000000	4.534472000000
C	3.205439000000	-1.186679000000	3.124238000000
H	6.580213000000	-2.782872000000	2.111443000000
C	2.102283000000	0.632197000000	2.489077000000
C	3.256242000000	0.626424000000	1.701365000000
O	2.067158000000	-0.463470000000	3.346792000000
C	0.887968000000	1.511559000000	2.579864000000
H	0.659734000000	1.655127000000	3.658176000000
C	-0.329552000000	0.836403000000	1.954270000000
C	-0.399317000000	0.616015000000	0.563308000000
C	-1.392735000000	0.407127000000	2.768079000000
H	0.424418000000	0.958230000000	-0.086595000000
H	-1.340622000000	0.576311000000	3.855645000000
C	-1.517253000000	-0.021644000000	0.006786000000
C	-2.509780000000	-0.235605000000	2.210316000000
H	-1.560644000000	-0.182835000000	-1.081616000000
H	-3.332411000000	-0.568459000000	2.862173000000
C	-2.574963000000	-0.451979000000	0.825918000000
H	-3.449648000000	-0.954645000000	0.384915000000
N	3.772014000000	1.559278000000	0.805743000000

S	3.452768000000	1.496332000000	-0.758632000000
O	4.494195000000	2.294652000000	-1.482476000000
O	2.029811000000	1.866354000000	-1.121484000000
C	3.662438000000	-0.222682000000	-1.269443000000
C	2.695632000000	-1.184550000000	-0.933743000000
C	4.877054000000	-0.607000000000	-1.856495000000
H	1.740041000000	-0.878136000000	-0.484748000000
H	5.608870000000	0.173761000000	-2.110977000000
C	2.967543000000	-2.538497000000	-1.166680000000
C	5.128004000000	-1.964606000000	-2.094271000000
H	2.214268000000	-3.293710000000	-0.890925000000
H	6.079950000000	-2.267301000000	-2.560023000000
C	4.188345000000	-2.954440000000	-1.737796000000
C	3.735224000000	6.669733000000	1.105302000000
C	2.539354000000	8.593007000000	0.201373000000
H	3.144799000000	9.238318000000	0.866443000000
H	2.529624000000	9.093458000000	-0.786638000000
C	1.111507000000	8.419426000000	0.757943000000
H	0.404686000000	8.205674000000	-0.070719000000
H	0.798534000000	9.393111000000	1.191035000000
C	0.998896000000	7.294461000000	1.793246000000
H	1.067592000000	6.314112000000	1.275690000000
H	-0.019510000000	7.300191000000	2.229065000000
C	2.032989000000	7.323427000000	2.926116000000
H	1.832337000000	6.444417000000	3.568923000000
H	1.892338000000	8.223180000000	3.560966000000
C	3.517689000000	7.272612000000	2.467742000000
H	4.138904000000	6.717970000000	3.193430000000
H	3.938406000000	8.299731000000	2.423910000000
C	3.104945000000	6.677929000000	-1.298140000000
H	2.047472000000	6.802247000000	-1.613175000000
H	3.735063000000	7.249728000000	-2.014374000000
C	3.495192000000	5.212576000000	-1.257240000000
H	3.673161000000	4.806726000000	-2.269830000000
H	2.690998000000	4.588637000000	-0.817691000000
C	4.746115000000	5.057058000000	-0.406969000000
H	5.596198000000	5.648643000000	-0.810036000000
H	5.031042000000	3.988048000000	-0.363389000000
N	3.238022000000	7.313347000000	0.029898000000

N	4.440449000000	5.540765000000	0.957902000000
C	4.491878000000	-4.419232000000	-1.926560000000
H	3.582255000000	-4.996465000000	-2.187145000000
H	5.246885000000	-4.583789000000	-2.720547000000
H	4.897117000000	-4.859285000000	-0.989451000000

III*D-SS*

Electronic energy = -2681.07459 Hartree

C	2.104486000000	3.835961000000	2.956740000000
O	1.551470000000	4.388361000000	3.906910000000
C	3.582603000000	3.630489000000	3.011164000000
C	1.327258000000	3.175342000000	1.814296000000
H	3.933976000000	3.096339000000	3.907393000000
H	2.009834000000	3.020755000000	0.954608000000
C	0.108039000000	3.941685000000	1.356232000000
C	-0.888360000000	4.394778000000	2.246158000000
C	-0.041745000000	4.203503000000	-0.022384000000
H	-0.791032000000	4.180195000000	3.318208000000
H	0.682756000000	3.766188000000	-0.728452000000
C	-1.977524000000	5.140801000000	1.771340000000
C	-1.131273000000	4.951669000000	-0.494845000000
H	-2.744769000000	5.492902000000	2.478262000000
H	-1.236503000000	5.139510000000	-1.574787000000
C	-2.096232000000	5.435630000000	0.403360000000
H	-2.953220000000	6.020579000000	0.035246000000
C	4.525697000000	4.110818000000	2.158546000000
C	5.961711000000	3.776669000000	2.277137000000
C	6.352376000000	2.467150000000	2.633519000000
C	6.959325000000	4.748560000000	2.041877000000
H	5.584307000000	1.691500000000	2.760724000000
H	6.667501000000	5.773778000000	1.764107000000
C	7.707980000000	2.144176000000	2.762211000000
C	8.315886000000	4.423057000000	2.174540000000
H	7.992913000000	1.113098000000	3.020443000000
H	9.083436000000	5.191093000000	1.995016000000
C	8.693826000000	3.119679000000	2.535809000000
H	9.759478000000	2.861491000000	2.630488000000
C	5.777431000000	-2.052088000000	2.183018000000
C	5.341824000000	-0.904535000000	1.507378000000
C	5.018887000000	-2.612911000000	3.239430000000
H	5.914926000000	-0.457319000000	0.682535000000
H	5.386470000000	-3.517108000000	3.748122000000
C	4.122468000000	-0.324214000000	1.903405000000
C	3.805840000000	-2.039138000000	3.654954000000
H	3.208240000000	-2.461404000000	4.475305000000
C	3.389220000000	-0.896588000000	2.965448000000
H	6.722735000000	-2.532291000000	1.887117000000
C	2.236569000000	0.873249000000	2.279046000000
C	3.356337000000	0.833451000000	1.440423000000
O	2.254633000000	-0.169231000000	3.198785000000
C	1.011341000000	1.732875000000	2.375874000000
H	0.780692000000	1.838589000000	3.458091000000
C	-0.217472000000	1.069896000000	1.760421000000
C	-0.307114000000	0.821981000000	0.376704000000
C	-1.283470000000	0.686142000000	2.594822000000
H	0.512996000000	1.132400000000	-0.292941000000
H	-1.218668000000	0.881931000000	3.677219000000
C	-1.444808000000	0.195746000000	-0.152958000000
C	-2.419982000000	0.056311000000	2.063836000000
H	-1.501890000000	0.011512000000	-1.236995000000
H	-3.243528000000	-0.240625000000	2.731484000000
C	-2.503327000000	-0.191613000000	0.685576000000
H	-3.393712000000	-0.683906000000	0.264811000000
N	3.794443000000	1.707453000000	0.455003000000
S	3.438219000000	1.456711000000	-1.097726000000
O	4.480370000000	2.137653000000	-1.926607000000
O	2.017338000000	1.821718000000	-1.452974000000
C	3.597186000000	-0.317201000000	-1.393618000000
C	2.625849000000	-1.210965000000	-0.914340000000
C	4.779696000000	-0.792146000000	-1.978309000000
H	1.692034000000	-0.833650000000	-0.473735000000
H	5.513252000000	-0.061661000000	-2.350064000000
C	2.866993000000	-2.587900000000	-0.992520000000
C	4.998803000000	-2.173601000000	-2.061697000000
H	2.112462000000	-3.289033000000	-0.601779000000
H	5.926247000000	-2.549499000000	-2.523168000000
C	4.059299000000	-3.093568000000	-1.552116000000
C	3.574875000000	6.205024000000	1.270106000000

C	2.699660000000	8.294918000000	0.353888000000
H	3.372467000000	8.826127000000	1.056054000000
H	2.820070000000	8.792216000000	-0.628333000000
C	1.247932000000	8.367877000000	0.843036000000
H	0.550282000000	8.154571000000	0.006444000000
H	1.056880000000	9.416825000000	1.155258000000
C	0.976807000000	7.389479000000	1.989440000000
H	0.849030000000	6.369589000000	1.573867000000
H	-0.002395000000	7.622602000000	2.452326000000
C	2.053803000000	7.352885000000	3.078552000000
H	1.701581000000	6.676125000000	3.878788000000
H	2.172149000000	8.358172000000	3.534677000000
C	3.460307000000	6.851110000000	2.629767000000
H	3.869739000000	6.151065000000	3.377126000000
H	4.174351000000	7.704215000000	2.589901000000
C	3.114743000000	6.376158000000	-1.177127000000
H	2.071299000000	6.518494000000	-1.530932000000
H	3.770380000000	7.011577000000	-1.811926000000
C	3.524379000000	4.917232000000	-1.242114000000
H	3.842098000000	4.628577000000	-2.260528000000
H	2.685443000000	4.238973000000	-0.996108000000
C	4.660500000000	4.670891000000	-0.266835000000
H	5.545654000000	5.299165000000	-0.508378000000
H	4.948011000000	3.605487000000	-0.269119000000
N	3.186880000000	6.916839000000	0.194783000000
N	4.197986000000	5.021140000000	1.096004000000
C	4.332350000000	-4.576027000000	-1.569520000000
H	3.413046000000	-5.160631000000	-1.774192000000
H	5.091565000000	-4.845551000000	-2.329773000000
H	4.717137000000	-4.913152000000	-0.582525000000

TS2$_{D\text{-}SS}$

Electronic energy = -2681.027222 Hartree

C	2.010552000000	3.629363000000	2.878923000000
O	1.519721000000	3.818118000000	4.016191000000
C	3.429100000000	3.568272000000	2.680908000000
C	1.048756000000	3.266659000000	1.709658000000
H	3.988761000000	3.459090000000	3.619849000000
H	1.596989000000	3.293133000000	0.750413000000
C	-0.204396000000	4.112195000000	1.577208000000
C	-0.943181000000	4.554924000000	2.696932000000
C	-0.701603000000	4.416923000000	0.291702000000
H	-0.558173000000	4.306912000000	3.695962000000
H	-0.179904000000	4.019576000000	-0.591911000000
C	-2.104416000000	5.324538000000	2.527844000000
C	-1.867909000000	5.178474000000	0.121204000000
H	-2.657156000000	5.671821000000	3.414889000000
H	-2.236573000000	5.393391000000	-0.893897000000
C	-2.570122000000	5.646614000000	1.242825000000
H	-3.486442000000	6.243453000000	1.115444000000
C	4.179845000000	3.513627000000	1.452638000000
C	5.673989000000	3.365021000000	1.614543000000
C	6.184426000000	2.528143000000	2.631632000000
C	6.594843000000	4.045089000000	0.792374000000
H	5.486779000000	1.980365000000	3.278867000000
H	6.238736000000	4.710462000000	-0.004074000000
C	7.563550000000	2.371132000000	2.812327000000
C	7.977145000000	3.888080000000	0.971716000000
H	7.930989000000	1.707066000000	3.609585000000
H	8.673138000000	4.427257000000	0.311185000000
C	8.470293000000	3.048428000000	1.980217000000
H	9.554804000000	2.923060000000	2.119250000000
C	5.584443000000	-1.714954000000	2.839557000000
C	5.254032000000	-0.571951000000	2.101495000000
C	4.624053000000	-2.381990000000	3.636197000000
H	6.005618000000	-0.052119000000	1.493236000000
H	4.917060000000	-3.278256000000	4.203442000000
C	3.930509000000	-0.091702000000	2.161294000000
C	3.304745000000	-1.912424000000	3.724757000000
H	2.543641000000	-2.406182000000	4.344971000000
C	3.000795000000	-0.769655000000	2.981243000000
H	6.613519000000	-2.103212000000	2.800584000000
C	1.900702000000	0.926483000000	2.077392000000
C	3.183930000000	1.001247000000	1.539487000000
O	1.782957000000	-0.139131000000	2.935816000000
C	0.668932000000	1.770166000000	1.962929000000
H	0.191804000000	1.743889000000	2.965385000000
C	-0.333678000000	1.229103000000	0.952887000000

C	0.027996000000	1.037001000000	-0.395731000000
C	-1.657752000000	0.963422000000	1.346761000000
H	1.049965000000	1.263002000000	-0.739984000000
H	-1.949390000000	1.124896000000	2.396323000000
C	-0.918236000000	0.580401000000	-1.325601000000
C	-2.604938000000	0.508891000000	0.416139000000
H	-0.616698000000	0.437078000000	-2.374834000000
H	-3.637419000000	0.308303000000	0.741327000000
C	-2.237385000000	0.313250000000	-0.923924000000
H	-2.978921000000	-0.044123000000	-1.654933000000
N	3.667600000000	1.969618000000	0.642917000000
S	4.046371000000	1.412388000000	-0.913441000000
O	5.364620000000	1.989918000000	-1.309564000000
O	2.888528000000	1.693389000000	-1.816154000000
C	4.236293000000	-0.376099000000	-0.894397000000
C	3.123129000000	-1.211039000000	-0.708571000000
C	5.517778000000	-0.904693000000	-1.091458000000
H	2.118984000000	-0.782698000000	-0.579297000000
H	6.360364000000	-0.218203000000	-1.257829000000
C	3.318800000000	-2.593892000000	-0.665771000000
C	5.689786000000	-2.295469000000	-1.064250000000
H	2.453173000000	-3.253844000000	-0.498740000000
H	6.695222000000	-2.718674000000	-1.216040000000
C	4.603427000000	-3.160230000000	-0.827451000000
C	3.416597000000	5.810248000000	1.064377000000
C	2.425902000000	8.025271000000	0.731758000000
H	3.390037000000	8.482790000000	1.030032000000
H	2.079249000000	8.583404000000	-0.159017000000
C	1.403846000000	8.134395000000	1.877397000000
H	0.373283000000	8.100352000000	1.465661000000
H	1.529541000000	9.141424000000	2.329793000000
C	1.540845000000	7.031007000000	2.931705000000
H	1.133922000000	6.093510000000	2.506769000000
H	0.873265000000	7.257143000000	3.787172000000
C	2.952225000000	6.743200000000	3.448820000000
H	2.872659000000	5.883915000000	4.143666000000
H	3.350216000000	7.604876000000	4.025127000000
C	3.976414000000	6.377156000000	2.346746000000
H	4.738974000000	5.683354000000	2.733948000000
H	4.524672000000	7.293319000000	2.023381000000
C	2.003856000000	6.236352000000	-0.953205000000
H	0.911622000000	6.232210000000	-0.748733000000
H	2.200774000000	7.042592000000	-1.690415000000
C	2.480103000000	4.884944000000	-1.487113000000
H	2.493079000000	4.889298000000	-2.594116000000
H	1.823374000000	4.048635000000	-1.185482000000
C	3.869746000000	4.625775000000	-0.933998000000
H	4.555439000000	5.451245000000	-1.233562000000
H	4.327767000000	3.697325000000	-1.300074000000
N	2.690725000000	6.645574000000	0.290805000000
N	3.796158000000	4.618774000000	0.538086000000
C	4.801783000000	-4.649079000000	-0.715001000000
H	3.985413000000	-5.209804000000	-1.212790000000
H	5.765306000000	-4.970616000000	-1.155547000000
H	4.802836000000	-4.958559000000	0.352464000000

IV$_{D\text{-}SS}$

Electronic energy = -2681.033863 Hartree

C	1.745508000000	3.381248000000	2.902281000000
O	1.338398000000	3.532461000000	4.084598000000
C	3.119133000000	3.245943000000	2.585620000000
C	0.682330000000	3.214305000000	1.772798000000
H	3.774970000000	3.162440000000	3.462504000000
H	1.167779000000	3.283260000000	0.785343000000
C	-0.467384000000	4.213586000000	1.780103000000
C	-0.893778000000	4.886109000000	2.946371000000
C	-1.168870000000	4.467457000000	0.577774000000
H	-0.360797000000	4.658879000000	3.881182000000
H	-0.898762000000	3.911261000000	-0.332821000000
C	-1.948321000000	5.811743000000	2.895139000000
C	-2.227239000000	5.386601000000	0.528290000000
H	-2.254314000000	6.332234000000	3.816498000000
H	-2.756412000000	5.556840000000	-0.422199000000
C	-2.617783000000	6.071957000000	1.689206000000
H	-3.448435000000	6.794040000000	1.655922000000
C	3.738097000000	3.099804000000	1.264321000000
C	5.270042000000	3.025501000000	1.317235000000
C	5.874675000000	2.231906000000	2.315578000000

C	6.107167000000	3.673098000000	0.386902000000
H	5.241534000000	1.717097000000	3.050327000000
H	5.667776000000	4.282837000000	-0.412417000000
C	7.267160000000	2.081855000000	2.374760000000
C	7.501298000000	3.524938000000	0.445687000000
H	7.709042000000	1.452356000000	3.162158000000
H	8.130834000000	4.036140000000	-0.298623000000
C	8.090231000000	2.725563000000	1.437276000000
H	9.183634000000	2.607897000000	1.481020000000
C	4.807086000000	-2.003830000000	3.009501000000
C	4.596146000000	-0.859199000000	2.232100000000
C	3.744794000000	-2.653504000000	3.678569000000
H	5.440099000000	-0.377193000000	1.727941000000
H	3.944422000000	-3.554467000000	4.277687000000
C	3.286649000000	-0.343499000000	2.109786000000
C	2.440357000000	-2.146492000000	3.604664000000
H	1.595611000000	-2.606096000000	4.136394000000
C	2.259518000000	-0.994814000000	2.835177000000
H	5.828312000000	-2.404652000000	3.100468000000
C	1.314642000000	0.773239000000	1.899360000000
C	2.632726000000	0.787310000000	1.447754000000
O	1.084525000000	-0.309275000000	2.708157000000
C	0.164787000000	1.735595000000	1.868380000000
H	-0.304657000000	1.650421000000	2.871092000000
C	-0.883920000000	1.391468000000	0.823662000000
C	-0.514476000000	1.029688000000	-0.487039000000
C	-2.250998000000	1.509953000000	1.139049000000
H	0.548291000000	0.919094000000	-0.753376000000
H	-2.543409000000	1.802016000000	2.159607000000
C	-1.497467000000	0.804410000000	-1.463046000000
C	-3.233156000000	1.283387000000	0.163761000000
H	-1.191790000000	0.521762000000	-2.482411000000
H	-4.297391000000	1.385185000000	0.426318000000
C	-2.859161000000	0.933290000000	-1.143215000000
H	-3.628702000000	0.755659000000	-1.910273000000
N	3.230783000000	1.770123000000	0.601691000000
S	3.734399000000	1.183933000000	-0.963202000000
O	4.594707000000	2.239911000000	-1.551454000000
O	2.510089000000	0.743167000000	-1.679929000000

C	4.782030000000	-0.255741000000	-0.796245000000
C	4.214636000000	-1.536782000000	-0.845715000000
C	6.163808000000	-0.066936000000	-0.640037000000
H	3.130767000000	-1.646084000000	-0.989771000000
H	6.583166000000	0.949406000000	-0.625651000000
C	5.050587000000	-2.648492000000	-0.701767000000
C	6.981113000000	-1.194761000000	-0.493739000000
H	4.613471000000	-3.658654000000	-0.729714000000
H	8.066094000000	-1.059026000000	-0.362704000000
C	6.441021000000	-2.498594000000	-0.508224000000
C	3.530598000000	5.551745000000	0.786489000000
C	3.325052000000	7.996300000000	0.619455000000
H	4.352479000000	8.105688000000	1.013915000000
H	3.248919000000	8.702042000000	-0.230314000000
C	2.287211000000	8.320891000000	1.710081000000
H	1.328475000000	8.627077000000	1.240015000000
H	2.656743000000	9.206223000000	2.270430000000
C	2.029789000000	7.141693000000	2.657888000000
H	1.394677000000	6.387594000000	2.146287000000
H	1.413961000000	7.485185000000	3.513248000000
C	3.287855000000	6.443249000000	3.184693000000
H	2.959730000000	5.603213000000	3.828687000000
H	3.880426000000	7.139509000000	3.815491000000
C	4.230298000000	5.864756000000	2.091586000000
H	4.731121000000	4.968958000000	2.478046000000
H	5.036435000000	6.586640000000	1.843502000000
C	2.358779000000	6.565880000000	-1.161886000000
H	1.641756000000	7.411010000000	-1.151773000000
H	3.025569000000	6.705146000000	-2.042214000000
C	1.631608000000	5.241784000000	-1.212476000000
H	1.160342000000	5.092041000000	-2.202841000000
H	0.824267000000	5.233146000000	-0.455189000000
C	2.619907000000	4.121615000000	-0.949817000000
H	3.372878000000	4.047079000000	-1.756998000000
H	2.100723000000	3.147717000000	-0.917807000000
N	3.158092000000	6.643712000000	0.066426000000
N	3.305753000000	4.305768000000	0.342276000000
C	7.317204000000	-3.704272000000	-0.290360000000
H	7.251718000000	-4.044393000000	0.765913000000

H	7.002640000000	-4.557987000000	-0.922859000000
H	8.381398000000	-3.484603000000	-0.502452000000

TS4$_{D\text{-}SS}$

Electronic energy = -2681.027545 Hartree

C	1.752474000000	3.360728000000	2.895756000000
O	1.337939000000	3.556056000000	4.064608000000
C	3.141067000000	3.268142000000	2.597677000000
C	0.704741000000	3.171612000000	1.755619000000
H	3.794413000000	3.431735000000	3.465458000000
H	1.211555000000	3.230032000000	0.777308000000
C	-0.435841000000	4.182690000000	1.729197000000
C	-0.881015000000	4.881696000000	2.871859000000
C	-1.114787000000	4.411080000000	0.509292000000
H	-0.367135000000	4.679246000000	3.822117000000
H	-0.823151000000	3.840315000000	-0.385016000000
C	-1.935024000000	5.805560000000	2.782262000000
C	-2.173090000000	5.327045000000	0.421366000000
H	-2.255650000000	6.347148000000	3.686528000000
H	-2.684462000000	5.477542000000	-0.541936000000
C	-2.584459000000	6.037328000000	1.560088000000
H	-3.414651000000	6.758097000000	1.496620000000
C	3.780803000000	3.034089000000	1.330955000000
C	5.306799000000	3.009056000000	1.380948000000
C	5.912390000000	2.273040000000	2.424203000000
C	6.146023000000	3.625867000000	0.431025000000
H	5.274626000000	1.790342000000	3.177261000000
H	5.701500000000	4.197816000000	-0.391174000000
C	7.306455000000	2.143193000000	2.504150000000
C	7.540884000000	3.498492000000	0.512525000000
H	7.748235000000	1.559351000000	3.326147000000
H	8.172162000000	3.987766000000	-0.245114000000
C	8.130046000000	2.751928000000	1.544837000000
H	9.224534000000	2.651801000000	1.604933000000
C	4.845094000000	-1.984386000000	2.986467000000
C	4.622996000000	-0.818210000000	2.245299000000
C	3.783357000000	-2.684399000000	3.604204000000
H	5.464286000000	-0.295375000000	1.778244000000
H	3.991935000000	-3.601068000000	4.175843000000
C	3.303498000000	-0.334657000000	2.108331000000
C	2.467653000000	-2.209281000000	3.515608000000
H	1.623248000000	-2.710623000000	4.008807000000
C	2.274566000000	-1.035261000000	2.783647000000
H	5.873811000000	-2.362415000000	3.089996000000
C	1.309661000000	0.738890000000	1.881114000000
C	2.635766000000	0.798718000000	1.467848000000
O	1.084653000000	-0.376139000000	2.646724000000
C	0.164478000000	1.702524000000	1.867252000000
H	-0.286412000000	1.628310000000	2.880088000000
C	-0.911601000000	1.354302000000	0.851882000000
C	-0.578759000000	0.963090000000	-0.459970000000
C	-2.268914000000	1.487600000000	1.200932000000
H	0.477021000000	0.853040000000	-0.751728000000
H	-2.531384000000	1.804967000000	2.221978000000
C	-1.588764000000	0.719070000000	-1.403309000000
C	-3.278148000000	1.244009000000	0.258181000000
H	-1.313825000000	0.412935000000	-2.424806000000
H	-4.334552000000	1.358005000000	0.546536000000
C	-2.940810000000	0.860612000000	-1.049410000000
H	-3.731350000000	0.668246000000	-1.791059000000
N	3.237305000000	1.814652000000	0.656969000000
S	3.721561000000	1.259605000000	-0.939741000000
O	4.590619000000	2.304378000000	-1.526852000000
O	2.476412000000	0.840973000000	-1.627596000000
C	4.740864000000	-0.200075000000	-0.781358000000
C	4.146480000000	-1.467850000000	-0.842628000000
C	6.127516000000	-0.040775000000	-0.635100000000
H	3.059508000000	-1.553731000000	-0.979209000000
H	6.568585000000	0.966390000000	-0.609920000000
C	4.960887000000	-2.598079000000	-0.718105000000
C	6.922995000000	-1.186835000000	-0.510489000000
H	4.502584000000	-3.598550000000	-0.753855000000
H	8.011904000000	-1.074545000000	-0.388233000000
C	6.355592000000	-2.478786000000	-0.534990000000
C	3.548782000000	5.656998000000	0.683897000000
C	3.286923000000	8.106541000000	0.574559000000
H	4.308859000000	8.227837000000	0.981998000000
H	3.205167000000	8.835467000000	-0.255862000000

C	2.238081000000	8.385128000000	1.666722000000
H	1.269582000000	8.666961000000	1.200982000000
H	2.577331000000	9.272683000000	2.243229000000
C	2.022216000000	7.183080000000	2.595239000000
H	1.409400000000	6.416793000000	2.075502000000
H	1.403313000000	7.492631000000	3.461519000000
C	3.307602000000	6.518437000000	3.101935000000
H	3.008168000000	5.682899000000	3.765210000000
H	3.890975000000	7.234631000000	3.719808000000
C	4.245361000000	5.951465000000	1.997083000000
H	4.748757000000	5.048616000000	2.364297000000
H	5.057362000000	6.671108000000	1.764212000000
C	2.353668000000	6.681313000000	-1.236285000000
H	1.632867000000	7.523193000000	-1.234002000000
H	3.014806000000	6.813639000000	-2.123003000000
C	1.627326000000	5.353101000000	-1.278489000000
H	1.137212000000	5.205139000000	-2.260538000000
H	0.834588000000	5.343971000000	-0.505542000000
C	2.626888000000	4.234085000000	-1.026131000000
H	3.349905000000	4.151945000000	-1.860953000000
H	2.107180000000	3.258255000000	-0.981042000000
N	3.152567000000	6.768212000000	-0.010246000000
N	3.351022000000	4.426945000000	0.230678000000
C	7.208380000000	-3.703783000000	-0.336056000000
H	7.198551000000	-4.010319000000	0.732633000000
H	6.829930000000	-4.564520000000	-0.921375000000
H	8.263590000000	-3.523043000000	-0.618285000000

3a*SS*

Electronic energy = -2219.059655 Hartree

C	1.867213000000	4.000796000000	2.383506000000
O	1.509906000000	4.589386000000	3.404782000000
C	3.288856000000	4.020763000000	1.990644000000
C	0.839810000000	3.271316000000	1.497312000000
H	3.856929000000	4.848394000000	2.442509000000
H	1.320559000000	2.939704000000	0.561308000000
C	-0.341200000000	4.137356000000	1.097470000000
C	-1.083300000000	4.897759000000	2.023625000000
C	-0.747319000000	4.123468000000	-0.251934000000
H	-0.760529000000	4.923783000000	3.073297000000
H	-0.177731000000	3.519408000000	-0.975947000000
C	-2.202924000000	5.630442000000	1.601870000000
C	-1.870485000000	4.850953000000	-0.670846000000
H	-2.770590000000	6.224382000000	2.334869000000
H	-2.175045000000	4.824052000000	-1.728285000000
C	-2.603072000000	5.607710000000	0.256498000000
H	-3.485708000000	6.179425000000	-0.069014000000
C	3.983760000000	3.115019000000	1.237450000000
C	5.393822000000	3.373240000000	0.853334000000
C	6.379939000000	2.381578000000	1.039807000000
C	5.777373000000	4.630318000000	0.338713000000
H	6.088028000000	1.409329000000	1.459421000000
H	5.003796000000	5.392344000000	0.163177000000
C	7.720501000000	2.641941000000	0.724548000000
C	7.115243000000	4.886230000000	0.015125000000
H	8.480957000000	1.863297000000	0.888956000000
H	7.399026000000	5.864695000000	-0.400897000000
C	8.091471000000	3.893351000000	0.207018000000
H	9.142669000000	4.096488000000	-0.047984000000
C	4.876399000000	-2.113256000000	2.821781000000
C	4.659749000000	-0.920185000000	2.121205000000
C	3.892982000000	-2.653202000000	3.682931000000
H	5.422787000000	-0.530456000000	1.433232000000
H	4.096595000000	-3.593372000000	4.216701000000
C	3.428286000000	-0.254885000000	2.293705000000
C	2.659338000000	-2.011426000000	3.867503000000
H	1.879000000000	-2.412119000000	4.529188000000
C	2.465516000000	-0.822564000000	3.160797000000
H	5.831316000000	-2.645464000000	2.696184000000
C	1.543453000000	1.026790000000	2.358475000000
C	2.805883000000	0.957246000000	1.779191000000
O	1.342551000000	-0.038204000000	3.197838000000
C	0.400102000000	1.987730000000	2.273845000000
H	0.168361000000	2.312057000000	3.312535000000
C	-0.858760000000	1.336450000000	1.714331000000
C	-0.821494000000	0.569969000000	0.534185000000
C	-2.093316000000	1.544620000000	2.356142000000
H	0.135541000000	0.422703000000	0.013113000000

H	-2.129852000000	2.149104000000	3.275684000000
C	-2.001133000000	0.022873000000	0.008725000000
C	-3.273217000000	0.999490000000	1.829155000000
H	-1.958758000000	-0.571061000000	-0.917248000000
H	-4.231860000000	1.174716000000	2.340881000000
C	-3.230351000000	0.235453000000	0.652915000000
H	-4.155474000000	-0.192520000000	0.237359000000
N	3.436228000000	1.851887000000	0.878261000000
S	3.303546000000	1.511557000000	-0.847178000000
O	3.843445000000	2.703003000000	-1.525147000000
O	1.916833000000	1.048141000000	-1.073551000000
C	4.380039000000	0.116929000000	-1.130788000000
C	3.860791000000	-1.182470000000	-1.019406000000
C	5.728052000000	0.347380000000	-1.440761000000
H	2.795687000000	-1.328619000000	-0.793451000000
H	6.097846000000	1.375989000000	-1.550460000000
C	4.724965000000	-2.268358000000	-1.191075000000
C	6.575794000000	-0.755751000000	-1.604796000000
H	4.328519000000	-3.291077000000	-1.096215000000
H	7.636678000000	-0.587471000000	-1.847124000000
C	6.095489000000	-2.076347000000	-1.472490000000
C	7.016672000000	-3.259152000000	-1.618039000000
H	6.590731000000	-4.021601000000	-2.301230000000
H	8.009972000000	-2.962770000000	-2.005531000000
H	7.171853000000	-3.759183000000	-0.638366000000

TS3*D-SS*

Electronic energy = -2681.023796 Hartree

C	1.213440000000	3.262910000000	-1.101673000000
O	0.690035000000	2.966795000000	-2.196604000000
C	2.601014000000	3.538014000000	-0.926779000000
C	0.303586000000	3.291894000000	0.159886000000
H	3.260603000000	3.220082000000	-1.747261000000
H	-0.515802000000	2.611055000000	-0.149206000000
C	-0.385996000000	4.649442000000	0.323240000000
C	-0.825947000000	5.148002000000	1.567472000000
C	-0.690056000000	5.411305000000	-0.830288000000
H	-0.668201000000	4.564122000000	2.486318000000
H	-0.407104000000	5.003956000000	-1.813010000000
C	-1.503104000000	6.376060000000	1.663842000000
C	-1.363734000000	6.637094000000	-0.735864000000
H	-1.842982000000	6.729619000000	2.650044000000
H	-1.591743000000	7.202094000000	-1.653770000000
C	-1.765548000000	7.135993000000	0.515009000000
H	-2.302413000000	8.094067000000	0.589902000000
C	3.191491000000	3.926466000000	0.324771000000
C	4.698985000000	3.853614000000	0.377666000000
C	5.365621000000	2.720388000000	-0.143260000000
C	5.498473000000	4.903503000000	0.885207000000
H	4.773491000000	1.914644000000	-0.592065000000
H	5.029929000000	5.827330000000	1.249145000000
C	6.761851000000	2.619399000000	-0.107077000000
C	6.898557000000	4.810310000000	0.908619000000
H	7.246276000000	1.716194000000	-0.507765000000
H	7.489947000000	5.649225000000	1.306369000000
C	7.539057000000	3.660137000000	0.425204000000
H	8.636624000000	3.582069000000	0.447520000000
C	5.186971000000	-1.008256000000	3.004065000000
C	4.625403000000	0.171557000000	3.506210000000
C	4.976274000000	-1.398948000000	1.663047000000
H	4.760131000000	0.450642000000	4.557161000000
H	5.426263000000	-2.333480000000	1.294491000000
C	3.863138000000	0.981034000000	2.638954000000
C	4.179584000000	-0.635275000000	0.796074000000
H	3.969298000000	-0.945446000000	-0.236928000000
C	3.628793000000	0.540397000000	1.315518000000
H	5.788594000000	-1.646316000000	3.667710000000
C	2.439862000000	2.457579000000	1.460885000000
C	3.045197000000	2.178760000000	2.801184000000
O	2.795428000000	1.362461000000	0.631082000000
C	0.923025000000	2.703410000000	1.463960000000
H	0.789211000000	3.412301000000	2.299935000000
C	0.246949000000	1.405339000000	1.902315000000
C	-0.031058000000	0.379910000000	0.974788000000
C	-0.049757000000	1.187137000000	3.263104000000
H	0.210653000000	0.525926000000	-0.089138000000
H	0.205920000000	1.960994000000	4.002728000000
C	-0.604448000000	-0.827987000000	1.397715000000

C	-0.624972000000	-0.021138000000	3.685172000000
H	-0.815574000000	-1.617576000000	0.660130000000
H	-0.848693000000	-0.172074000000	4.752271000000
C	-0.906883000000	-1.031892000000	2.753765000000
H	-1.358611000000	-1.979821000000	3.084072000000
N	2.683327000000	2.902314000000	3.836223000000
S	3.355697000000	3.152505000000	5.322767000000
O	2.743732000000	2.246055000000	6.321886000000
O	3.248810000000	4.623442000000	5.518624000000
C	5.125819000000	2.796116000000	5.258992000000
C	5.922748000000	3.248737000000	4.197171000000
C	5.686938000000	2.069161000000	6.318961000000
H	5.489908000000	3.806595000000	3.354052000000
H	5.031758000000	1.716400000000	7.129139000000
C	7.288204000000	2.941883000000	4.188707000000
C	7.059064000000	1.785023000000	6.303166000000
H	7.901007000000	3.271974000000	3.335534000000
H	7.500936000000	1.209903000000	7.132346000000
C	7.881763000000	2.209230000000	5.238774000000
C	2.363084000000	6.300148000000	0.205498000000
C	1.527478000000	8.594292000000	0.008995000000
H	0.937524000000	8.263692000000	-0.864131000000
H	0.828565000000	9.193587000000	0.622933000000
C	2.758427000000	9.422038000000	-0.416218000000
H	3.006135000000	10.166250000000	0.369791000000
H	2.484896000000	10.007024000000	-1.319610000000
C	3.995733000000	8.555861000000	-0.685779000000
H	4.378138000000	8.171551000000	0.285030000000
H	4.810941000000	9.192963000000	-1.086129000000
C	3.769963000000	7.369161000000	-1.632529000000
H	4.705152000000	6.772566000000	-1.667301000000
H	3.598148000000	7.736168000000	-2.665730000000
C	2.596617000000	6.417638000000	-1.279329000000
H	2.778868000000	5.405426000000	-1.679623000000
H	1.651695000000	6.754042000000	-1.744347000000
C	1.851106000000	7.588090000000	2.266535000000
H	1.995796000000	8.666143000000	2.476538000000
H	0.848954000000	7.298649000000	2.647091000000
C	2.943147000000	6.742461000000	2.885510000000
H	2.884675000000	6.722822000000	3.990351000000
H	3.939592000000	7.149418000000	2.607838000000
C	2.776520000000	5.321483000000	2.395768000000
H	1.884745000000	4.866441000000	2.860173000000
H	3.621979000000	4.701814000000	2.725370000000
N	1.880693000000	7.413740000000	0.807292000000
N	2.697406000000	5.210157000000	0.929291000000
C	9.358851000000	1.910054000000	5.229359000000
H	9.731277000000	1.730350000000	4.201302000000
H	9.938380000000	2.765187000000	5.639661000000
H	9.601631000000	1.023593000000	5.847218000000

3a'$_{SS}$

Electronic energy = -2219.072942 Hartree

C	1.919798000000	4.762603000000	-0.322444000000
O	1.604468000000	5.823576000000	-0.849526000000
C	3.339703000000	4.395105000000	-0.144787000000
C	0.880026000000	3.739243000000	0.163394000000
H	4.056950000000	5.112093000000	-0.574281000000
H	0.801138000000	3.002353000000	-0.667128000000
C	-0.478722000000	4.366439000000	0.382278000000
C	-0.687056000000	5.277825000000	1.435826000000
C	-1.560203000000	4.051961000000	-0.459201000000
H	0.148221000000	5.545853000000	2.101536000000
H	-1.408068000000	3.344518000000	-1.289964000000
C	-1.947174000000	5.853098000000	1.646133000000
C	-2.823709000000	4.626489000000	-0.254197000000
H	-2.092213000000	6.562554000000	2.475030000000
H	-3.658293000000	4.366527000000	-0.923081000000
C	-3.021429000000	5.527696000000	0.802167000000
H	-4.011805000000	5.978094000000	0.968273000000
C	3.780110000000	3.270152000000	0.482011000000
C	5.225228000000	2.998503000000	0.657636000000
C	5.766604000000	1.708464000000	0.456436000000
C	6.092557000000	4.040849000000	1.056688000000
H	5.115466000000	0.885811000000	0.130735000000
H	5.674253000000	5.036990000000	1.262340000000
C	7.136423000000	1.474964000000	0.637897000000
C	7.459451000000	3.802838000000	1.243524000000
H	7.541666000000	0.466307000000	0.466588000000

H	8.113249000000	4.622622000000	1.576443000000
C	7.987289000000	2.519213000000	1.032949000000
H	9.060627000000	2.330047000000	1.185772000000
C	3.002571000000	-2.244285000000	2.317869000000
C	3.272073000000	-0.998713000000	2.893346000000
C	2.574558000000	-2.344895000000	0.975250000000
H	3.611066000000	-0.898616000000	3.932350000000
H	2.368633000000	-3.337837000000	0.546670000000
C	3.108734000000	0.159626000000	2.102408000000
C	2.394318000000	-1.208878000000	0.174264000000
H	2.049556000000	-1.272119000000	-0.866812000000
C	2.659932000000	0.034207000000	0.760594000000
H	3.121651000000	-3.157178000000	2.919148000000
C	2.767614000000	2.287389000000	1.063846000000
C	3.278670000000	1.587225000000	2.336661000000
O	2.525352000000	1.214949000000	0.104251000000
C	1.414948000000	2.984643000000	1.403506000000
H	1.695336000000	3.725432000000	2.181048000000
C	0.431211000000	2.010259000000	2.025327000000
C	-0.360265000000	1.154585000000	1.234167000000
C	0.346614000000	1.903576000000	3.429204000000
H	-0.312419000000	1.223499000000	0.137875000000
H	0.975901000000	2.544228000000	4.066283000000
C	-1.205962000000	0.207444000000	1.828707000000
C	-0.498273000000	0.956239000000	4.024615000000
H	-1.815881000000	-0.453290000000	1.194003000000
H	-0.542217000000	0.884195000000	5.121832000000
C	-1.276666000000	0.103790000000	3.226511000000
H	-1.940574000000	-0.639687000000	3.693282000000
N	3.679729000000	2.387720000000	3.270592000000
S	4.219381000000	1.963764000000	4.820033000000
O	4.710313000000	0.567250000000	4.954191000000
O	3.188111000000	2.482832000000	5.745593000000
C	5.654998000000	3.038680000000	4.856574000000
C	5.475621000000	4.417234000000	5.041838000000
C	6.926176000000	2.483977000000	4.663105000000
H	4.464268000000	4.818816000000	5.198795000000
H	7.025167000000	1.399203000000	4.516215000000
C	6.599219000000	5.251233000000	5.019123000000
C	8.038407000000	3.334595000000	4.650511000000
H	6.470419000000	6.336462000000	5.156394000000
H	9.041506000000	2.908740000000	4.491079000000
C	7.896099000000	4.727191000000	4.822720000000
C	9.101789000000	5.631314000000	4.820010000000
H	8.843000000000	6.658188000000	4.495552000000
H	9.538436000000	5.711004000000	5.838933000000
H	9.900021000000	5.245361000000	4.155437000000

RC$_{D\text{-}SR}$

Electronic energy = -2681.034771 Hartree

C	2.402669000000	1.384581000000	0.148929000000
O	2.847157000000	2.208412000000	-0.651092000000
C	3.130179000000	0.986046000000	1.328310000000
C	1.027320000000	0.736427000000	-0.074193000000
H	-2.430567000000	2.335395000000	1.265929000000
H	0.480805000000	1.429071000000	-0.740649000000
C	1.150356000000	-0.587536000000	-0.812176000000
C	0.395815000000	-0.788638000000	-1.985292000000
C	1.950511000000	-1.641459000000	-0.326969000000
H	-0.230454000000	0.035627000000	-2.359405000000
H	2.545670000000	-1.499614000000	0.587924000000
C	0.431992000000	-2.021889000000	-2.652850000000
C	1.992333000000	-2.870958000000	-0.999666000000
H	-0.167450000000	-2.166363000000	-3.564947000000
H	2.624662000000	-3.683981000000	-0.611067000000
C	1.229681000000	-3.067360000000	-2.162161000000
H	1.259821000000	-4.034590000000	-2.686712000000
C	3.681092000000	0.697989000000	2.393718000000
C	4.335292000000	0.365802000000	3.612955000000
C	4.169621000000	-0.922019000000	4.186092000000
C	5.151414000000	1.329597000000	4.262525000000
H	3.531605000000	-1.660543000000	3.678009000000
H	5.231287000000	2.337709000000	3.802939000000
C	4.822617000000	-1.244356000000	5.380229000000
C	5.788768000000	0.990250000000	5.461837000000
H	4.697959000000	-2.246193000000	5.818275000000
H	6.417622000000	1.737703000000	5.969621000000
C	5.632882000000	-0.290107000000	6.020324000000

H	6.142435000000	-0.547063000000	6.961788000000
C	-4.925074000000	-2.444804000000	0.126883000000
C	-4.516860000000	-1.108544000000	0.219251000000
C	-4.021652000000	-3.511124000000	0.352507000000
H	-5.218090000000	-0.281679000000	0.036719000000
H	-4.377259000000	-4.548703000000	0.264982000000
C	-3.169690000000	-0.848422000000	0.541937000000
C	-2.679647000000	-3.272617000000	0.686309000000
H	-1.962081000000	-4.085565000000	0.863995000000
C	-2.292040000000	-1.933804000000	0.774640000000
H	-5.970617000000	-2.673343000000	-0.129424000000
C	-1.078742000000	-0.096159000000	1.030992000000
C	-2.356553000000	0.339963000000	0.714102000000
O	-1.042308000000	-1.470392000000	1.095201000000
C	0.234547000000	0.591315000000	1.263128000000
H	0.817568000000	-0.091860000000	1.915844000000
C	0.068316000000	1.910433000000	2.010317000000
C	0.063401000000	1.908954000000	3.419838000000
C	-0.120644000000	3.135839000000	1.337519000000
H	0.228413000000	0.961632000000	3.956760000000
H	-0.114925000000	3.173452000000	0.238905000000
C	-0.141244000000	3.093961000000	4.140492000000
C	-0.343780000000	4.320696000000	2.058093000000
H	-0.132817000000	3.072193000000	5.240712000000
H	-0.514509000000	5.258670000000	1.509131000000
C	-0.354848000000	4.304475000000	3.460668000000
H	-0.522855000000	5.234324000000	4.024651000000
N	-2.836390000000	1.663154000000	0.597622000000
S	-2.728640000000	2.439413000000	-0.947517000000
O	-1.508022000000	2.017355000000	-1.683002000000
O	-2.980098000000	3.864972000000	-0.654866000000
C	-4.130805000000	1.718310000000	-1.791041000000
C	-3.935860000000	0.627162000000	-2.647895000000
C	-5.411857000000	2.221344000000	-1.519349000000
H	-2.919563000000	0.252808000000	-2.833163000000
H	-5.527594000000	3.083019000000	-0.846246000000
C	-5.055514000000	0.020827000000	-3.231195000000
C	-6.516781000000	1.602079000000	-2.113856000000
H	-4.914033000000	-0.847153000000	-3.893523000000
H	-7.528136000000	1.984870000000	-1.905294000000
C	-6.359653000000	0.491810000000	-2.974019000000
C	-7.555946000000	-0.154389000000	-3.622764000000
H	-8.456892000000	-0.082830000000	-2.982443000000
H	-7.799161000000	0.346757000000	-4.584487000000
H	-7.370514000000	-1.222506000000	-3.848727000000
C	5.056572000000	4.168742000000	1.364004000000
C	4.641081000000	4.942755000000	-1.029658000000
H	4.060993000000	4.114403000000	-1.498440000000
H	4.190479000000	5.886750000000	-1.402695000000
C	6.098936000000	4.898663000000	-1.493195000000
H	6.694412000000	5.571828000000	-0.840790000000
H	6.145227000000	5.347334000000	-2.507461000000
C	6.709258000000	3.491189000000	-1.533182000000
H	7.812920000000	3.582011000000	-1.632663000000
H	6.360605000000	2.958538000000	-2.443770000000
C	6.355201000000	2.663587000000	-0.290497000000
H	7.062407000000	1.814151000000	-0.193256000000
H	5.347521000000	2.213448000000	-0.408351000000
C	6.372074000000	3.493070000000	1.004110000000
H	7.185345000000	4.251616000000	0.964451000000
H	6.596758000000	2.849268000000	1.873431000000
C	3.088206000000	5.488349000000	0.768802000000
H	2.922797000000	6.406640000000	0.165059000000
H	2.291172000000	4.764278000000	0.474955000000
C	2.997269000000	5.796513000000	2.256915000000
H	1.971973000000	6.130718000000	2.515047000000
H	3.691784000000	6.627807000000	2.504133000000
C	3.393379000000	4.544196000000	3.034827000000
H	2.588686000000	3.775617000000	2.945603000000
H	3.469608000000	4.765932000000	4.122458000000
N	4.405406000000	4.953937000000	0.420153000000
N	4.648995000000	3.973838000000	2.586154000000

TS1*D-SR*

Electronic energy = -2681.01064 Hartree

C	2.425134000000	0.896138000000	0.440424000000
O	3.066822000000	1.479844000000	-0.453882000000
C	3.006519000000	0.484409000000	1.678042000000

C	0.967314000000	0.482672000000	0.155089000000
H	-2.569331000000	2.186571000000	1.384697000000
H	0.549221000000	1.238869000000	-0.533754000000
C	0.944205000000	-0.846053000000	-0.592149000000
C	0.171471000000	-0.967465000000	-1.763974000000
C	1.621479000000	-1.980813000000	-0.103628000000
H	-0.356347000000	-0.080103000000	-2.144813000000
H	2.232900000000	-1.888643000000	0.807657000000
C	0.065420000000	-2.201395000000	-2.423761000000
C	1.519596000000	-3.212162000000	-0.766025000000
H	-0.545391000000	-2.281701000000	-3.336662000000
H	2.055556000000	-4.089603000000	-0.372081000000
C	0.735790000000	-3.329253000000	-1.925432000000
H	0.652373000000	-4.297529000000	-2.442553000000
C	3.718931000000	0.762386000000	2.700509000000
C	4.330916000000	0.179831000000	3.879613000000
C	4.117237000000	-1.203439000000	4.122012000000
C	5.128207000000	0.905563000000	4.793844000000
H	3.488653000000	-1.767465000000	3.417299000000
H	5.283262000000	1.975752000000	4.598615000000
C	4.694777000000	-1.831375000000	5.229558000000
C	5.698432000000	0.271310000000	5.905691000000
H	4.520213000000	-2.905452000000	5.396223000000
H	6.315529000000	0.855478000000	6.605693000000
C	5.488101000000	-1.097965000000	6.129842000000
H	5.936811000000	-1.593451000000	7.004199000000
C	-5.064917000000	-2.586808000000	0.276921000000
C	-4.644362000000	-1.252792000000	0.346358000000
C	-4.179106000000	-3.656468000000	0.550971000000
H	-5.331787000000	-0.423572000000	0.125563000000
H	-4.543485000000	-4.692261000000	0.479361000000
C	-3.302834000000	-0.998449000000	0.695513000000
C	-2.843044000000	-3.423259000000	0.911768000000
H	-2.137822000000	-4.238340000000	1.125291000000
C	-2.443435000000	-2.086805000000	0.977009000000
H	-6.106239000000	-2.810775000000	-0.000151000000
C	-1.213050000000	-0.257671000000	1.210154000000
C	-2.479852000000	0.184696000000	0.855753000000
O	-1.196919000000	-1.628479000000	1.315731000000
C	0.099290000000	0.429349000000	1.448857000000
H	0.656948000000	-0.207809000000	2.170830000000
C	-0.120826000000	1.786101000000	2.108145000000
C	-0.339983000000	1.840988000000	3.500456000000
C	-0.183675000000	2.986810000000	1.370422000000
H	-0.287407000000	0.910829000000	4.087478000000
H	-0.038847000000	2.971458000000	0.281563000000
C	-0.616619000000	3.057765000000	4.139691000000
C	-0.479864000000	4.204112000000	2.005966000000
H	-0.777646000000	3.078745000000	5.228258000000
H	-0.556890000000	5.121227000000	1.403046000000
C	-0.693776000000	4.245452000000	3.392295000000
H	-0.924647000000	5.199372000000	3.890241000000
N	-2.946590000000	1.509715000000	0.705642000000
S	-2.786287000000	2.275198000000	-0.838373000000
O	-1.529202000000	1.876919000000	-1.522604000000
O	-3.079862000000	3.698050000000	-0.569281000000
C	-4.138442000000	1.514785000000	-1.729399000000
C	-3.892971000000	0.396685000000	-2.537249000000
C	-5.435180000000	2.014217000000	-1.537683000000
H	-2.866778000000	0.022941000000	-2.658545000000
H	-5.588836000000	2.896206000000	-0.899382000000
C	-4.978356000000	-0.240036000000	-3.152359000000
C	-6.504973000000	1.364838000000	-2.163422000000
H	-4.798239000000	-1.129705000000	-3.775353000000
H	-7.528457000000	1.744539000000	-2.017537000000
C	-6.297073000000	0.227136000000	-2.975521000000
C	-7.455670000000	-0.453698000000	-3.656705000000
H	-8.383225000000	-0.381404000000	-3.055378000000
H	-7.667355000000	0.019973000000	-4.639513000000
H	-7.244936000000	-1.523889000000	-3.847809000000
C	4.829271000000	3.116181000000	1.716708000000
C	5.004601000000	4.443225000000	-0.408901000000
H	4.446789000000	3.770175000000	-1.101332000000
H	4.699451000000	5.479265000000	-0.658139000000
C	6.515959000000	4.315548000000	-0.622352000000
H	7.024949000000	4.746136000000	0.266087000000
H	6.798327000000	4.969685000000	-1.473200000000
C	6.999457000000	2.883892000000	-0.895561000000

H	8.099089000000	2.844043000000	-0.737355000000
H	6.834969000000	2.632180000000	-1.964967000000
C	6.294066000000	1.840906000000	-0.019874000000
H	6.887514000000	0.904058000000	-0.007970000000
H	5.302572000000	1.573562000000	-0.438695000000
C	6.079467000000	2.303635000000	1.428758000000
H	6.953844000000	2.891925000000	1.788455000000
H	6.017553000000	1.427291000000	2.097220000000
C	3.286692000000	4.948852000000	1.272191000000
H	3.406805000000	5.987837000000	0.900849000000
H	2.430668000000	4.499107000000	0.715101000000
C	3.011982000000	4.930095000000	2.768618000000
H	2.050625000000	5.434711000000	2.987735000000
H	3.814909000000	5.484216000000	3.299979000000
C	2.968384000000	3.477412000000	3.226100000000
H	2.046622000000	2.992291000000	2.844316000000
H	2.922931000000	3.402680000000	4.333975000000
N	4.520825000000	4.221147000000	0.962766000000
N	4.127391000000	2.733085000000	2.765626000000

I*D-SR*

Electronic energy = -2681.030574 Hartree

C	2.463144000000	0.592575000000	0.765565000000
O	3.227284000000	1.128693000000	-0.084232000000
C	2.926564000000	0.089516000000	2.014760000000
C	1.007112000000	0.318566000000	0.343413000000
H	-2.570207000000	2.099780000000	1.529623000000
H	0.694557000000	1.156542000000	-0.306781000000
C	0.923603000000	-0.944244000000	-0.505975000000
C	0.182295000000	-0.928943000000	-1.704008000000
C	1.515261000000	-2.151939000000	-0.086898000000
H	-0.277157000000	0.016495000000	-2.029834000000
H	2.103482000000	-2.157080000000	0.844807000000
C	0.022692000000	-2.100581000000	-2.460285000000
C	1.357161000000	-3.321476000000	-0.843771000000
H	-0.560693000000	-2.073052000000	-3.394304000000
H	1.824753000000	-4.258672000000	-0.503377000000
C	0.605309000000	-3.302933000000	-2.030253000000
H	0.479086000000	-4.223056000000	-2.621649000000
C	3.705569000000	0.747922000000	2.895014000000
C	4.355494000000	0.217600000000	4.105810000000
C	4.054960000000	-1.093945000000	4.545897000000
C	5.292123000000	0.973497000000	4.848573000000
H	3.316981000000	-1.669813000000	3.966830000000
H	5.546920000000	1.991077000000	4.513954000000
C	4.677085000000	-1.629629000000	5.677041000000
C	5.907977000000	0.437749000000	5.989554000000
H	4.432225000000	-2.653525000000	5.999980000000
H	6.634942000000	1.043867000000	6.552286000000
C	5.605439000000	-0.866167000000	6.409833000000
H	6.087324000000	-1.286961000000	7.305430000000
C	-5.162800000000	-2.535532000000	0.144345000000
C	-4.701759000000	-1.220417000000	0.283203000000
C	-4.317356000000	-3.644802000000	0.386305000000
H	-5.358258000000	-0.360547000000	0.086679000000
H	-4.712693000000	-4.663876000000	0.259773000000
C	-3.360797000000	-1.025584000000	0.670902000000
C	-2.982694000000	-3.471445000000	0.784180000000
H	-2.307745000000	-4.317370000000	0.974196000000
C	-2.543096000000	-2.152999000000	0.919911000000
H	-6.204765000000	-2.713156000000	-0.162560000000
C	-1.260509000000	-0.377444000000	1.268096000000
C	-2.504604000000	0.121526000000	0.904117000000
O	-1.292346000000	-1.751390000000	1.308219000000
C	0.066022000000	0.243988000000	1.585842000000
H	0.576133000000	-0.448693000000	2.293926000000
C	-0.116810000000	1.573785000000	2.304857000000
C	-0.292307000000	1.578900000000	3.704708000000
C	-0.171966000000	2.806429000000	1.619022000000
H	-0.241349000000	0.625056000000	4.252221000000
H	-0.061893000000	2.829017000000	0.525847000000
C	-0.516726000000	2.775797000000	4.400894000000
C	-0.414399000000	4.004644000000	2.311759000000
H	-0.643416000000	2.755862000000	5.494089000000
H	-0.488411000000	4.946910000000	1.747931000000
C	-0.583236000000	3.995446000000	3.705676000000
H	-0.773328000000	4.933743000000	4.248714000000
N	-2.932098000000	1.465042000000	0.803445000000

S	-2.707246000000	2.303064000000	-0.692710000000
O	-1.416925000000	1.950201000000	-1.339716000000
O	-3.022510000000	3.710617000000	-0.370013000000
C	-4.009853000000	1.576933000000	-1.679503000000
C	-3.727527000000	0.470782000000	-2.492068000000
C	-5.309695000000	2.089853000000	-1.558898000000
H	-2.701113000000	0.083534000000	-2.555970000000
H	-5.491131000000	2.961119000000	-0.913166000000
C	-4.780141000000	-0.139374000000	-3.185779000000
C	-6.345952000000	1.468002000000	-2.264275000000
H	-4.572398000000	-1.020690000000	-3.812014000000
H	-7.371687000000	1.859278000000	-2.175915000000
C	-6.101362000000	0.343076000000	-3.083638000000
C	-7.222961000000	-0.309330000000	-3.849387000000
H	-8.186484000000	-0.231185000000	-3.308459000000
H	-7.363116000000	0.181346000000	-4.836764000000
H	-7.015961000000	-1.379901000000	-4.042544000000
C	4.697755000000	2.534745000000	1.552147000000
C	5.176427000000	4.119408000000	-0.308849000000
H	4.679523000000	3.565751000000	-1.136878000000
H	4.930965000000	5.189149000000	-0.451162000000
C	6.698052000000	3.946950000000	-0.358375000000
H	7.100662000000	4.205145000000	0.644125000000
H	7.111521000000	4.708658000000	-1.051169000000
C	7.158521000000	2.547115000000	-0.791828000000
H	8.211812000000	2.401841000000	-0.468822000000
H	7.168758000000	2.482438000000	-1.900626000000
C	6.267311000000	1.431436000000	-0.236220000000
H	6.798132000000	0.461500000000	-0.320489000000
H	5.336198000000	1.314283000000	-0.822217000000
C	5.862398000000	1.621242000000	1.229341000000
H	6.725921000000	1.989565000000	1.831502000000
H	5.592804000000	0.643267000000	1.664466000000
C	3.378575000000	4.557238000000	1.349370000000
H	3.544025000000	5.585002000000	0.973356000000
H	2.461891000000	4.157575000000	0.856483000000
C	3.219791000000	4.538168000000	2.862712000000
H	2.347486000000	5.145794000000	3.171751000000
H	4.125698000000	4.975498000000	3.332819000000
C	3.010049000000	3.097592000000	3.290913000000
H	1.982433000000	2.769758000000	3.045598000000
H	3.140055000000	2.963061000000	4.384965000000
N	4.540936000000	3.749749000000	0.967419000000
N	3.950553000000	2.191414000000	2.622261000000

II$_{\text{D-}SR}$

Electronic energy = -2681.033605 Hartree

C	1.720515000000	2.060998000000	5.509612000000
O	1.210558000000	2.327956000000	6.765753000000
C	3.009973000000	2.238624000000	5.240913000000
C	0.597011000000	1.657214000000	4.579818000000
H	1.955815000000	2.553085000000	7.355381000000
H	-0.083662000000	1.078264000000	5.238852000000
C	-0.225127000000	2.869051000000	4.149378000000
C	0.377762000000	3.997375000000	3.576739000000
C	-1.628444000000	2.834242000000	4.272462000000
H	1.468922000000	4.001488000000	3.454107000000
H	-2.112861000000	1.947874000000	4.711621000000
C	-0.400031000000	5.059730000000	3.094635000000
C	-2.409820000000	3.901887000000	3.809876000000
H	0.103484000000	5.899636000000	2.593346000000
H	-3.505763000000	3.856643000000	3.903329000000
C	-1.796925000000	5.016391000000	3.212241000000
H	-2.412453000000	5.844463000000	2.827937000000
C	4.331665000000	2.386450000000	5.214674000000
C	5.284129000000	1.280150000000	5.495146000000
C	4.956439000000	-0.045294000000	5.139566000000
C	6.523002000000	1.535144000000	6.124710000000
H	4.006787000000	-0.239382000000	4.621299000000
H	6.788522000000	2.567926000000	6.400668000000
C	5.846940000000	-1.090839000000	5.411398000000
C	7.408337000000	0.484564000000	6.402262000000
H	5.586459000000	-2.115743000000	5.107457000000
H	8.367787000000	0.694878000000	6.899070000000
C	7.073675000000	-0.831780000000	6.045108000000
H	7.774268000000	-1.654737000000	6.252210000000
C	6.266838000000	0.121786000000	0.492674000000
C	5.422524000000	1.172232000000	0.874834000000

C	5.927268000000	-1.227454000000	0.750114000000
H	5.692948000000	2.214670000000	0.657401000000
H	6.610402000000	-2.029805000000	0.432629000000
C	4.210488000000	0.861700000000	1.528107000000
C	4.733453000000	-1.559679000000	1.408560000000
H	4.453654000000	-2.599915000000	1.628627000000
C	3.907601000000	-0.495254000000	1.782656000000
H	7.211881000000	0.349654000000	-0.023873000000
C	2.260976000000	0.707601000000	2.690318000000
C	3.114176000000	1.645066000000	2.107529000000
O	2.743406000000	-0.592960000000	2.480311000000
C	0.958975000000	0.631894000000	3.443904000000
H	1.060483000000	-0.332873000000	3.982999000000
C	-0.241657000000	0.465864000000	2.511684000000
C	-0.447290000000	1.344528000000	1.430447000000
C	-1.197845000000	-0.531688000000	2.777212000000
H	0.278267000000	2.144377000000	1.210133000000
H	-1.037990000000	-1.224368000000	3.620354000000
C	-1.597751000000	1.223748000000	0.638195000000
C	-2.348159000000	-0.653048000000	1.981414000000
H	-1.748616000000	1.924699000000	-0.197063000000
H	-3.085111000000	-1.441765000000	2.198901000000
C	-2.551651000000	0.228839000000	0.908968000000
H	-3.453001000000	0.138225000000	0.283069000000
N	3.122507000000	3.025583000000	2.265442000000
S	2.706362000000	4.081131000000	1.126677000000
O	2.961818000000	5.437430000000	1.727239000000
O	1.367506000000	3.849250000000	0.504720000000
C	3.868732000000	3.924967000000	-0.255670000000
C	3.614465000000	2.961798000000	-1.246510000000
C	5.054591000000	4.672608000000	-0.286132000000
H	2.665359000000	2.407278000000	-1.222586000000
H	5.214006000000	5.457680000000	0.466973000000
C	4.573679000000	2.720456000000	-2.235984000000
C	6.006763000000	4.423303000000	-1.285851000000
H	4.377344000000	1.953379000000	-3.002036000000
H	6.936859000000	5.014162000000	-1.312462000000
C	5.791857000000	3.433555000000	-2.267353000000
C	4.620144000000	4.835527000000	5.295675000000
C	4.636309000000	7.280384000000	5.261230000000
H	4.822855000000	7.278648000000	6.353139000000
H	5.325918000000	8.032509000000	4.832780000000
C	3.168022000000	7.635301000000	4.961437000000
H	3.090994000000	8.086989000000	3.951266000000
H	2.855409000000	8.415683000000	5.687940000000
C	2.243778000000	6.415783000000	5.016594000000
H	2.438051000000	5.796857000000	4.115278000000
H	1.191251000000	6.744246000000	4.910502000000
C	2.362465000000	5.547422000000	6.275406000000
H	1.647084000000	4.708007000000	6.176900000000
H	2.054035000000	6.120993000000	7.175004000000
C	3.782219000000	4.963698000000	6.539809000000
H	3.723776000000	3.976691000000	7.030372000000
H	4.343311000000	5.620512000000	7.235532000000
C	5.685195000000	5.960691000000	3.393276000000
H	4.883190000000	5.919351000000	2.618356000000
H	6.246674000000	6.908599000000	3.286653000000
C	6.600476000000	4.749249000000	3.303120000000
H	7.445185000000	4.854375000000	4.017302000000
H	7.028292000000	4.672459000000	2.284348000000
C	5.783348000000	3.502823000000	3.574490000000
H	6.415198000000	2.603417000000	3.689309000000
H	5.037114000000	3.319220000000	2.764433000000
N	5.038505000000	5.976858000000	4.713959000000
N	4.958370000000	3.634479000000	4.798387000000
C	6.825667000000	3.138047000000	-3.324387000000
H	6.395034000000	3.211838000000	-4.344471000000
H	7.684110000000	3.834861000000	-3.265193000000
H	7.219852000000	2.105082000000	-3.218580000000

III$_{D\text{-}SR}$

Electronic energy = -2681.045967 Hartree

C	1.821159000000	2.158958000000	5.382955000000
O	1.585492000000	2.732901000000	6.442601000000
C	3.203190000000	1.607829000000	5.209416000000
C	0.637535000000	1.820647000000	4.457108000000
H	3.184083000000	0.517078000000	5.395425000000
H	-0.021049000000	1.264432000000	5.160372000000

C	-0.150939000000	3.096483000000	4.164526000000
C	-0.037224000000	3.792866000000	2.953591000000
C	-1.006832000000	3.610765000000	5.162300000000
H	0.613574000000	3.426141000000	2.156501000000
H	-1.113315000000	3.072096000000	6.115790000000
C	-0.746951000000	4.982809000000	2.737143000000
C	-1.718069000000	4.801179000000	4.950736000000
H	-0.614957000000	5.500052000000	1.775046000000
H	-2.384905000000	5.183916000000	5.738884000000
C	-1.588690000000	5.494995000000	3.734981000000
H	-2.149602000000	6.427646000000	3.567699000000
C	4.468259000000	2.115046000000	5.188634000000
C	5.616145000000	1.186352000000	5.412546000000
C	5.644756000000	-0.101301000000	4.835693000000
C	6.704302000000	1.597340000000	6.214684000000
H	4.833667000000	-0.410337000000	4.160403000000
H	6.696914000000	2.604322000000	6.660196000000
C	6.726231000000	-0.961270000000	5.068335000000
C	7.780864000000	0.732434000000	6.451582000000
H	6.739340000000	-1.951717000000	4.589179000000
H	8.615293000000	1.062194000000	7.089112000000
C	7.795780000000	-0.550639000000	5.879631000000
H	8.647166000000	-1.224748000000	6.057352000000
C	6.283748000000	0.113890000000	0.542006000000
C	5.446279000000	1.186965000000	0.870865000000
C	5.900033000000	-1.224976000000	0.791812000000
H	5.757713000000	2.218446000000	0.659614000000
H	6.578070000000	-2.046189000000	0.514298000000
C	4.194993000000	0.915872000000	1.466954000000
C	4.667074000000	-1.521738000000	1.391536000000
H	4.349829000000	-2.552226000000	1.606531000000
C	3.851058000000	-0.433490000000	1.718688000000
H	7.258808000000	0.316473000000	0.073425000000
C	2.206726000000	0.807575000000	2.563185000000
C	3.093232000000	1.727075000000	2.002348000000
O	2.663397000000	-0.502449000000	2.378624000000
C	0.901375000000	0.794416000000	3.307444000000
H	0.965368000000	-0.177943000000	3.842078000000
C	-0.325892000000	0.649150000000	2.399627000000

C	-0.361117000000	1.167204000000	1.092331000000
C	-1.465716000000	-0.010770000000	2.899331000000
H	0.510686000000	1.701530000000	0.685187000000
H	-1.446909000000	-0.433961000000	3.917608000000
C	-1.518519000000	1.036286000000	0.309597000000
C	-2.622655000000	-0.141549000000	2.118431000000
H	-1.530608000000	1.458706000000	-0.706715000000
H	-3.503065000000	-0.661740000000	2.526313000000
C	-2.652502000000	0.384534000000	0.817091000000
H	-3.558713000000	0.284321000000	0.200245000000
N	3.087655000000	3.107882000000	2.160805000000
S	2.789010000000	4.133181000000	0.951654000000
O	3.001580000000	5.508133000000	1.508244000000
O	1.502882000000	3.866949000000	0.227979000000
C	4.047998000000	3.924902000000	-0.339738000000
C	3.847696000000	2.960641000000	-1.341277000000
C	5.243295000000	4.656733000000	-0.294869000000
H	2.891470000000	2.420115000000	-1.382133000000
H	5.361644000000	5.447510000000	0.459717000000
C	4.868685000000	2.699361000000	-2.261811000000
C	6.258897000000	4.386183000000	-1.224018000000
H	4.714237000000	1.930002000000	-3.035124000000
H	7.197689000000	4.962418000000	-1.187593000000
C	6.096652000000	3.394233000000	-2.213503000000
C	4.480440000000	4.626625000000	5.266215000000
C	4.224351000000	7.049170000000	5.070671000000
H	4.305703000000	7.144580000000	6.171768000000
H	4.846160000000	7.855493000000	4.637888000000
C	2.765529000000	7.152339000000	4.613926000000
H	2.715379000000	7.228866000000	3.508577000000
H	2.351750000000	8.092356000000	5.038708000000
C	1.955941000000	5.936190000000	5.082670000000
H	2.092586000000	5.106851000000	4.354807000000
H	0.872642000000	6.162714000000	5.046867000000
C	2.352178000000	5.476702000000	6.490784000000
H	1.579184000000	4.787378000000	6.872762000000
H	2.372755000000	6.347767000000	7.179988000000
C	3.716555000000	4.721386000000	6.562833000000
H	3.546566000000	3.697005000000	6.937812000000

H	4.404303000000	5.220238000000	7.280530000000
C	5.564021000000	5.809052000000	3.383568000000
H	4.818588000000	5.766936000000	2.554458000000
H	6.092833000000	6.781234000000	3.340793000000
C	6.529041000000	4.641561000000	3.338749000000
H	7.328932000000	4.764144000000	4.100394000000
H	7.015287000000	4.585442000000	2.345313000000
C	5.736096000000	3.373403000000	3.554842000000
H	6.389271000000	2.487964000000	3.637419000000
H	5.008396000000	3.222278000000	2.726791000000
N	4.826323000000	5.773491000000	4.653337000000
N	4.893102000000	3.435010000000	4.780965000000
C	7.186446000000	3.095953000000	-3.211750000000
H	6.896180000000	3.423572000000	-4.232703000000
H	8.132624000000	3.609423000000	-2.952560000000
H	7.390792000000	2.007169000000	-3.271615000000

X*D*

Electronic energy = -2681.047021 Hartree

C	0.700089000000	3.651151000000	1.143870000000
O	0.212006000000	4.842818000000	0.651393000000
C	2.149404000000	3.607086000000	1.055236000000
C	-0.166621000000	2.702438000000	1.624488000000
H	2.661804000000	2.911769000000	1.733528000000
H	-0.763309000000	4.761522000000	0.554381000000
C	-1.616352000000	3.085938000000	1.593666000000
C	-2.273920000000	3.495400000000	2.773389000000
C	-2.349728000000	3.039347000000	0.386017000000
H	-1.706907000000	3.539910000000	3.716107000000
H	-1.849233000000	2.700602000000	-0.533844000000
C	-3.633124000000	3.838978000000	2.749863000000
C	-3.709361000000	3.387474000000	0.365029000000
H	-4.133561000000	4.152396000000	3.678840000000
H	-4.269168000000	3.333603000000	-0.580971000000
C	-4.354594000000	3.783957000000	1.546151000000
H	-5.422483000000	4.049611000000	1.530386000000
C	2.930723000000	4.429834000000	0.279744000000
C	4.418209000000	4.330785000000	0.326910000000
C	5.248396000000	5.473090000000	0.385529000000
C	5.020787000000	3.053363000000	0.311980000000
H	4.805126000000	6.479810000000	0.395922000000
H	4.360482000000	2.174920000000	0.217592000000
C	6.642433000000	5.341372000000	0.428443000000
C	6.415300000000	2.926235000000	0.357816000000
H	7.272319000000	6.242728000000	0.474704000000
H	6.862691000000	1.919997000000	0.340692000000
C	7.232308000000	4.066451000000	0.413968000000
H	8.328022000000	3.965110000000	0.445203000000
C	5.488716000000	-1.475762000000	2.493993000000
C	4.769502000000	-0.746400000000	1.539007000000
C	4.948864000000	-1.751922000000	3.771988000000
H	5.205896000000	-0.538839000000	0.554502000000
H	5.537007000000	-2.334047000000	4.497618000000
C	3.476083000000	-0.286456000000	1.867953000000
C	3.677804000000	-1.282726000000	4.134060000000
H	3.244716000000	-1.465217000000	5.127871000000
C	2.978464000000	-0.552206000000	3.168227000000
H	6.496477000000	-1.841398000000	2.243751000000
C	1.449561000000	0.692289000000	2.158511000000
C	2.438239000000	0.502353000000	1.192112000000
O	1.764204000000	0.039013000000	3.346998000000
C	0.081367000000	1.308160000000	2.196327000000
H	-0.152882000000	1.392656000000	3.282565000000
C	-0.969504000000	0.336819000000	1.626626000000
C	-0.846025000000	-0.151796000000	0.313322000000
C	-2.084078000000	-0.037958000000	2.397498000000
H	0.024726000000	0.107137000000	-0.309522000000
H	-2.186609000000	0.344394000000	3.425701000000
C	-1.832178000000	-0.994531000000	-0.219482000000
C	-3.070489000000	-0.882470000000	1.863860000000
H	-1.713413000000	-1.370230000000	-1.247659000000
H	-3.937384000000	-1.167823000000	2.479718000000
C	-2.949133000000	-1.360700000000	0.550104000000
H	-3.721119000000	-2.023751000000	0.129778000000
N	2.516202000000	1.182531000000	-0.008274000000
S	2.595641000000	0.620451000000	-1.508768000000
O	3.106160000000	1.750134000000	-2.347865000000
O	1.326859000000	-0.033468000000	-1.977616000000

C	3.843076000000	-0.679171000000	-1.621815000000
C	3.504249000000	-1.998423000000	-1.283731000000
C	5.157978000000	-0.343858000000	-1.976678000000
H	2.460426000000	-2.240039000000	-1.036606000000
H	5.385829000000	0.694727000000	-2.257856000000
C	4.504380000000	-2.976871000000	-1.261330000000
C	6.147350000000	-1.337576000000	-1.955737000000
H	4.244087000000	-4.010129000000	-0.981452000000
H	7.182322000000	-1.079967000000	-2.232915000000
C	5.843090000000	-2.663793000000	-1.582972000000
C	6.915866000000	-3.720382000000	-1.507204000000
H	6.594119000000	-4.664262000000	-1.992567000000
H	7.855442000000	-3.387523000000	-1.989468000000
H	7.151567000000	-3.968040000000	-0.449832000000
P	2.225907000000	5.482883000000	-1.033809000000
C	1.609172000000	7.070430000000	-0.365282000000
C	2.125327000000	7.511000000000	0.869610000000
C	0.665577000000	7.858656000000	-1.050829000000
H	2.826702000000	6.873550000000	1.428413000000
H	0.226987000000	7.509830000000	-1.995436000000
C	1.718804000000	8.743535000000	1.399840000000
C	0.258769000000	9.088036000000	-0.510772000000
H	2.124681000000	9.079412000000	2.365777000000
H	-0.483790000000	9.696751000000	-1.048225000000
C	0.788493000000	9.536001000000	0.709315000000
H	0.466467000000	10.500824000000	1.129209000000
C	3.602495000000	5.873877000000	-2.162916000000
C	4.073563000000	7.184565000000	-2.356591000000
C	4.197115000000	4.780337000000	-2.831312000000
H	3.602301000000	8.027541000000	-1.828929000000
H	3.820174000000	3.751928000000	-2.679556000000
C	5.159080000000	7.406344000000	-3.218520000000
C	5.285811000000	5.017897000000	-3.679605000000
H	5.532609000000	8.429664000000	-3.374206000000
H	5.761323000000	4.169659000000	-4.194342000000
C	5.768292000000	6.324242000000	-3.872039000000
H	6.625024000000	6.500233000000	-4.540363000000
C	1.044825000000	4.662491000000	-2.157293000000
C	0.654128000000	3.327186000000	-1.958071000000
C	0.659702000000	5.346181000000	-3.333318000000
H	1.021733000000	2.756540000000	-1.094759000000
H	1.047180000000	6.352404000000	-3.552036000000
C	-0.155664000000	2.689904000000	-2.910271000000
C	-0.179203000000	4.713736000000	-4.259972000000
H	-0.383549000000	1.623652000000	-2.765467000000
H	-0.485041000000	5.250629000000	-5.170541000000
C	-0.590862000000	3.386708000000	-4.045847000000
H	-1.225447000000	2.883187000000	-4.790992000000

TS2$_{D\text{-}SR}$

Electronic energy = -2681.012066 Hartree

C	-0.084989000000	3.162299000000	-1.071510000000
O	-1.257852000000	3.426736000000	-1.407473000000
C	0.903868000000	2.786928000000	-2.025558000000
C	0.252841000000	3.160655000000	0.444895000000
H	0.468080000000	2.608234000000	-3.015942000000
H	1.327847000000	3.364635000000	0.591914000000
C	-0.495709000000	4.237690000000	1.192424000000
C	0.223971000000	5.252051000000	1.856884000000
C	-1.906901000000	4.279172000000	1.234431000000
H	1.326372000000	5.225834000000	1.832600000000
H	-2.475893000000	3.512952000000	0.693414000000
C	-0.432802000000	6.282631000000	2.547740000000
C	-2.564733000000	5.307228000000	1.926635000000
H	0.152072000000	7.060781000000	3.063160000000
H	-3.665489000000	5.327077000000	1.947512000000
C	-1.835564000000	6.311819000000	2.584062000000
H	-2.359588000000	7.114790000000	3.125089000000
C	2.306163000000	2.538024000000	-1.897022000000
C	3.073791000000	2.496935000000	-3.214832000000
C	2.456273000000	2.077897000000	-4.413667000000
C	4.418781000000	2.920017000000	-3.285707000000
H	1.432184000000	1.688050000000	-4.389248000000
H	4.947637000000	3.245182000000	-2.380974000000
C	3.150985000000	2.100369000000	-5.628470000000
C	5.117072000000	2.940077000000	-4.501027000000
H	2.641583000000	1.762464000000	-6.543467000000

H	6.165758000000	3.274262000000	-4.518386000000
C	4.484292000000	2.533493000000	-5.683632000000
H	5.028058000000	2.546960000000	-6.640300000000
C	5.321606000000	-1.179265000000	1.889887000000
C	4.657036000000	-0.554691000000	0.826194000000
C	4.708047000000	-1.320744000000	3.157632000000
H	5.114097000000	-0.458711000000	-0.170334000000
H	5.253961000000	-1.825648000000	3.968851000000
C	3.357798000000	-0.063958000000	1.054373000000
C	3.418332000000	-0.823083000000	3.405397000000
H	2.932116000000	-0.914204000000	4.386907000000
C	2.781283000000	-0.191743000000	2.332993000000
H	6.336469000000	-1.576975000000	1.737995000000
C	1.308379000000	0.924177000000	1.075537000000
C	2.379898000000	0.635459000000	0.225560000000
O	1.559069000000	0.416545000000	2.346724000000
C	0.032894000000	1.728270000000	1.061090000000
H	-0.155830000000	1.904133000000	2.139179000000
C	-1.168301000000	0.936696000000	0.557387000000
C	-1.199498000000	0.370360000000	-0.733340000000
C	-2.272213000000	0.740628000000	1.409919000000
H	-0.356176000000	0.510364000000	-1.425473000000
H	-2.254896000000	1.180062000000	2.419982000000
C	-2.308430000000	-0.377928000000	-1.152808000000
C	-3.385996000000	-0.001463000000	0.987494000000
H	-2.308969000000	-0.813130000000	-2.163763000000
H	-4.240077000000	-0.141876000000	1.667904000000
C	-3.405645000000	-0.566220000000	-0.296849000000
H	-4.275978000000	-1.151801000000	-0.630933000000
N	2.688049000000	0.931634000000	-1.117041000000
S	2.501888000000	-0.360079000000	-2.244570000000
O	3.744852000000	-0.431521000000	-3.050573000000
O	1.195055000000	-0.250034000000	-2.952639000000
C	2.419276000000	-1.857471000000	-1.242642000000
C	1.253163000000	-2.182304000000	-0.532269000000
C	3.535848000000	-2.702730000000	-1.231060000000
H	0.367086000000	-1.535116000000	-0.584351000000
H	4.417002000000	-2.432862000000	-1.830394000000
C	1.241704000000	-3.342083000000	0.249884000000
C	3.497885000000	-3.869963000000	-0.457792000000
H	0.335187000000	-3.591288000000	0.823556000000
H	4.372973000000	-4.538597000000	-0.443661000000
C	2.364485000000	-4.196350000000	0.313976000000
C	2.810565000000	4.807386000000	-1.039445000000
C	3.022324000000	7.096846000000	-0.175418000000
H	1.950548000000	7.223273000000	-0.412369000000
H	3.153001000000	7.496339000000	0.849210000000
C	3.912158000000	7.841779000000	-1.188431000000
H	4.871783000000	8.135576000000	-0.712766000000
H	3.400621000000	8.787609000000	-1.464731000000
C	4.201467000000	6.994554000000	-2.432862000000
H	4.918147000000	6.189224000000	-2.160490000000
H	4.732002000000	7.612265000000	-3.186145000000
C	2.967340000000	6.357863000000	-3.083217000000
H	3.314391000000	5.736218000000	-3.933326000000
H	2.319180000000	7.148132000000	-3.515629000000
C	2.070181000000	5.459096000000	-2.184206000000
H	1.592220000000	4.671662000000	-2.785812000000
H	1.219876000000	6.025204000000	-1.753731000000
C	4.311906000000	5.247683000000	0.884161000000
H	4.993460000000	6.104743000000	1.056567000000
H	3.808392000000	5.024969000000	1.851447000000
C	5.057703000000	4.032553000000	0.365923000000
H	5.738765000000	3.630147000000	1.139958000000
H	5.679276000000	4.316793000000	-0.510184000000
C	4.041463000000	2.971291000000	-0.002178000000
H	3.536267000000	2.601701000000	0.911834000000
H	4.497130000000	2.084754000000	-0.477298000000
N	3.317900000000	5.659081000000	-0.111762000000
N	3.022062000000	3.481131000000	-0.942013000000
C	2.357772000000	-5.410019000000	1.206531000000
H	1.350746000000	-5.867867000000	1.268663000000
H	3.072188000000	-6.180993000000	0.857484000000
H	2.653226000000	-5.131037000000	2.241295000000

3a$_{SR}$

Electronic energy = -2219.036868 Hartree

C	0.406337000000	1.836416000000	-1.592903000000

O	-0.491148000000	1.163659000000	-2.079889000000
C	1.585674000000	2.285414000000	-2.410502000000
C	0.414207000000	2.378642000000	-0.146374000000
H	1.472532000000	3.187217000000	-3.034081000000
H	1.242473000000	3.117752000000	-0.140226000000
C	-0.844733000000	3.162653000000	0.237659000000
C	-0.718776000000	4.115726000000	1.271986000000
C	-2.103297000000	3.006396000000	-0.374081000000
H	0.262971000000	4.274173000000	1.748310000000
H	-2.224587000000	2.289354000000	-1.196583000000
C	-1.817533000000	4.870721000000	1.702027000000
C	-3.203898000000	3.767692000000	0.051532000000
H	-1.691561000000	5.605188000000	2.512021000000
H	-4.178150000000	3.628240000000	-0.441337000000
C	-3.070374000000	4.696083000000	1.093117000000
H	-3.936573000000	5.287792000000	1.425736000000
C	2.753679000000	1.609150000000	-2.317828000000
C	4.030936000000	2.024658000000	-2.941530000000
C	4.282404000000	3.390228000000	-3.210990000000
C	5.023115000000	1.078705000000	-3.283126000000
H	3.539431000000	4.146682000000	-2.916371000000
H	4.849680000000	0.010284000000	-3.097243000000
C	5.477956000000	3.795587000000	-3.815578000000
C	6.218938000000	1.486919000000	-3.888537000000
H	5.656685000000	4.864341000000	-4.008326000000
H	6.974540000000	0.732524000000	-4.155187000000
C	6.452699000000	2.843966000000	-4.158283000000
H	7.395308000000	3.161870000000	-4.628968000000
C	6.637329000000	-0.506637000000	0.976489000000
C	5.622205000000	-0.327684000000	0.029769000000
C	6.431412000000	-0.226476000000	2.348109000000
H	5.777274000000	-0.571095000000	-1.028097000000
H	7.253172000000	-0.382864000000	3.062818000000
C	4.372902000000	0.150571000000	0.475621000000
C	5.195732000000	0.244225000000	2.815144000000
H	5.011824000000	0.466804000000	3.875465000000
C	4.198358000000	0.419236000000	1.853246000000
H	7.618679000000	-0.880337000000	0.648117000000
C	2.255129000000	0.896514000000	0.887783000000
C	3.089846000000	0.468685000000	-0.134384000000
O	2.925688000000	0.881430000000	2.090261000000
C	0.833440000000	1.386694000000	0.995337000000
H	0.876752000000	2.056132000000	1.881697000000
C	-0.147892000000	0.284710000000	1.416819000000
C	-1.268989000000	-0.095705000000	0.659775000000
C	0.052352000000	-0.321557000000	2.677195000000
H	-1.420399000000	0.321164000000	-0.342854000000
H	0.929090000000	-0.039633000000	3.279816000000
C	-2.189907000000	-1.024060000000	1.171839000000
C	-0.856504000000	-1.262508000000	3.177332000000
H	-3.067817000000	-1.297220000000	0.566921000000
H	-0.683197000000	-1.715228000000	4.165703000000
C	-1.996292000000	-1.604958000000	2.431803000000
H	-2.727938000000	-2.322954000000	2.833221000000
N	2.718946000000	0.420652000000	-1.496020000000
S	2.594783000000	-1.117538000000	-2.292441000000
O	3.792908000000	-1.910958000000	-1.928985000000
O	2.285314000000	-0.791256000000	-3.697236000000
C	1.197136000000	-1.934595000000	-1.543257000000
C	-0.014716000000	-1.964381000000	-2.247381000000
C	1.367817000000	-2.631551000000	-0.335284000000
H	-0.112360000000	-1.396078000000	-3.182158000000
H	2.336466000000	-2.604718000000	0.183123000000
C	-1.066893000000	-2.732824000000	-1.734691000000
C	0.295873000000	-3.375225000000	0.166182000000
H	-2.020759000000	-2.774367000000	-2.283306000000
H	0.412192000000	-3.915484000000	1.118044000000
C	-0.927916000000	-3.457197000000	-0.533269000000
C	-2.045348000000	-4.321660000000	-0.012671000000
H	-3.019176000000	-4.052040000000	-0.465469000000
H	-1.855692000000	-5.391417000000	-0.245714000000
H	-2.136673000000	-4.238712000000	1.088331000000

TS3*D-SR*

Electronic energy = -2681.020888 Hartree

C	0.555705000000	3.064267000000	-1.249608000000
O	-0.199849000000	2.804564000000	-2.203212000000
C	1.956229000000	3.350160000000	-1.399988000000

C	0.099909000000	3.036390000000	0.229099000000
H	2.376763000000	3.107637000000	-2.384244000000
H	0.415573000000	4.023284000000	0.614372000000
C	-1.383701000000	2.960689000000	0.544583000000
C	-1.816313000000	3.460181000000	1.792886000000
C	-2.350853000000	2.403564000000	-0.316933000000
H	-1.077235000000	3.907926000000	2.480271000000
H	-2.026448000000	2.054664000000	-1.304526000000
C	-3.160673000000	3.397940000000	2.183615000000
C	-3.699542000000	2.347209000000	0.068843000000
H	-3.467573000000	3.791781000000	3.165083000000
H	-4.438778000000	1.911585000000	-0.621267000000
C	-4.111639000000	2.834692000000	1.317862000000
H	-5.170010000000	2.780274000000	1.616275000000
C	2.833724000000	3.608981000000	-0.296033000000
C	4.321079000000	3.563720000000	-0.524966000000
C	4.861220000000	2.876675000000	-1.633753000000
C	5.227492000000	4.150796000000	0.389553000000
H	4.202836000000	2.354162000000	-2.336668000000
H	4.854357000000	4.681778000000	1.275645000000
C	6.246264000000	2.780973000000	-1.819100000000
C	6.613414000000	4.054831000000	0.204949000000
H	6.623747000000	2.210529000000	-2.680368000000
H	7.292180000000	4.511413000000	0.941453000000
C	7.131862000000	3.371087000000	-0.905367000000
H	8.219784000000	3.286574000000	-1.047763000000
C	6.534711000000	-0.184895000000	1.424688000000
C	5.521490000000	-0.095081000000	0.463801000000
C	6.412139000000	0.454349000000	2.679062000000
H	5.634255000000	-0.550341000000	-0.527151000000
H	7.221937000000	0.359004000000	3.418484000000
C	4.359958000000	0.645034000000	0.774595000000
C	5.282239000000	1.224849000000	2.996074000000
H	5.180977000000	1.746619000000	3.958256000000
C	4.281971000000	1.304199000000	2.024200000000
H	7.447036000000	-0.755099000000	1.195475000000
C	2.441109000000	1.999580000000	0.893242000000
C	3.121696000000	0.968830000000	0.078013000000
O	3.134153000000	2.035294000000	2.145887000000
C	0.913603000000	2.000763000000	1.100999000000
H	0.792696000000	2.334976000000	2.152014000000
C	0.334218000000	0.590846000000	1.057201000000
C	-0.162165000000	0.011650000000	-0.130060000000
C	0.301933000000	-0.168363000000	2.243298000000
H	-0.112025000000	0.565483000000	-1.075338000000
H	0.695020000000	0.272279000000	3.173809000000
C	-0.697641000000	-1.284896000000	-0.114555000000
C	-0.227224000000	-1.469020000000	2.257655000000
H	-1.071621000000	-1.720622000000	-1.053062000000
H	-0.248064000000	-2.041064000000	3.198121000000
C	-0.737254000000	-2.029063000000	1.076013000000
H	-1.157681000000	-3.046380000000	1.080335000000
N	2.547599000000	0.614318000000	-1.051423000000
S	3.207554000000	-0.492003000000	-2.137767000000
O	4.546482000000	-0.059664000000	-2.645627000000
O	2.135264000000	-0.758202000000	-3.113597000000
C	3.480385000000	-2.000258000000	-1.176239000000
C	2.664664000000	-2.331238000000	-0.085782000000
C	4.500537000000	-2.864588000000	-1.601290000000
H	1.864401000000	-1.653860000000	0.238763000000
H	5.133022000000	-2.577122000000	-2.454327000000
C	2.888167000000	-3.537111000000	0.590073000000
C	4.702742000000	-4.071256000000	-0.919455000000
H	2.246867000000	-3.789772000000	1.449395000000
H	5.500890000000	-4.753471000000	-1.253318000000
C	3.906680000000	-4.426852000000	0.189659000000
C	2.484118000000	6.049564000000	-0.097913000000
C	2.284268000000	8.496053000000	-0.118913000000
H	1.726892000000	8.424305000000	-1.070575000000
H	1.728352000000	9.222847000000	0.503981000000
C	3.731767000000	8.968368000000	-0.366768000000
H	4.116336000000	9.496559000000	0.531380000000
H	3.705586000000	9.723148000000	-1.180833000000
C	4.689820000000	7.822297000000	-0.721760000000
H	4.900582000000	7.226702000000	0.193151000000
H	5.670324000000	8.245856000000	-1.020622000000
C	4.194801000000	6.870094000000	-1.817099000000
H	4.943060000000	6.060686000000	-1.934444000000

H	4.142983000000	7.399255000000	-2.791173000000
C	2.811442000000	6.211154000000	-1.561589000000
H	2.745824000000	5.225127000000	-2.047522000000
H	1.997300000000	6.810419000000	-2.013999000000
C	2.059752000000	7.253314000000	2.030505000000
H	2.466071000000	8.223003000000	2.379967000000
H	0.978250000000	7.233586000000	2.291139000000
C	2.794198000000	6.086138000000	2.660071000000
H	2.580499000000	6.030535000000	3.744706000000
H	3.890216000000	6.224556000000	2.545469000000
C	2.347512000000	4.800269000000	1.989449000000
H	1.283272000000	4.595010000000	2.235067000000
H	2.925232000000	3.936258000000	2.352678000000
N	2.216064000000	7.199690000000	0.572855000000
N	2.496684000000	4.855888000000	0.522573000000
C	4.155040000000	-5.706612000000	0.946848000000
H	4.779410000000	-5.520033000000	1.847463000000
H	3.207897000000	-6.161238000000	1.299908000000
H	4.688321000000	-6.453315000000	0.326446000000

3a'$_{SR}$

Electronic energy = -2219.063299 Hartree

C	0.690033000000	2.989235000000	-1.047471000000
O	-0.336829000000	3.221237000000	-1.676108000000
C	2.000685000000	2.893609000000	-1.730093000000
C	0.749521000000	2.874867000000	0.478437000000
H	1.996535000000	3.212464000000	-2.784335000000
H	1.249771000000	3.814330000000	0.804332000000
C	-0.579780000000	2.782346000000	1.189229000000
C	-0.769348000000	3.500011000000	2.386485000000
C	-1.618075000000	1.945351000000	0.732081000000
H	0.035773000000	4.159096000000	2.750962000000
H	-1.490425000000	1.380419000000	-0.200523000000
C	-1.961978000000	3.387197000000	3.116715000000
C	-2.811570000000	1.833171000000	1.458707000000
H	-2.090463000000	3.958009000000	4.049099000000
H	-3.612889000000	1.176456000000	1.087025000000
C	-2.988220000000	2.550347000000	2.652841000000
H	-3.927225000000	2.459387000000	3.220039000000
C	3.160741000000	2.478419000000	-1.147130000000
C	4.462679000000	2.668227000000	-1.839738000000
C	5.462186000000	1.671123000000	-1.943989000000
C	4.715152000000	3.933492000000	-2.425623000000
H	5.307636000000	0.665720000000	-1.537069000000
H	3.958502000000	4.725994000000	-2.326389000000
C	6.664985000000	1.938300000000	-2.612357000000
C	5.920634000000	4.196344000000	-3.087500000000
H	7.419896000000	1.140939000000	-2.683498000000
H	6.097606000000	5.191808000000	-3.522324000000
C	6.902708000000	3.197529000000	-3.183177000000
H	7.852625000000	3.402348000000	-3.700049000000
C	7.031377000000	1.773733000000	2.976528000000
C	6.255979000000	0.915501000000	2.192190000000
C	6.674707000000	3.133263000000	3.132104000000
H	6.513130000000	-0.145102000000	2.077036000000
H	7.303922000000	3.787153000000	3.755390000000
C	5.108706000000	1.440155000000	1.556671000000
C	5.541544000000	3.674376000000	2.510750000000
H	5.258945000000	4.730266000000	2.620042000000
C	4.772396000000	2.807837000000	1.722175000000
H	7.929216000000	1.387423000000	3.480056000000
C	3.143066000000	2.024330000000	0.311500000000
C	4.114517000000	0.878994000000	0.655365000000
O	3.661603000000	3.186583000000	1.051379000000
C	1.726237000000	1.729165000000	0.887480000000
H	1.858465000000	1.801800000000	1.985932000000
C	1.147007000000	0.351307000000	0.607869000000
C	0.922063000000	-0.151765000000	-0.690751000000
C	0.744342000000	-0.435336000000	1.704204000000
H	1.250589000000	0.418811000000	-1.568884000000
H	0.906804000000	-0.054314000000	2.724602000000
C	0.310773000000	-1.397985000000	-0.879923000000
C	0.127384000000	-1.681059000000	1.518868000000
H	0.157932000000	-1.776673000000	-1.901582000000
H	-0.181187000000	-2.272683000000	2.394021000000
C	-0.093598000000	-2.166825000000	0.222684000000
H	-0.572079000000	-3.145798000000	0.069791000000
N	3.881202000000	-0.275774000000	0.115196000000

S 4.870975000000 -1.633926000000 0.345435000000
O 5.142514000000 -1.921961000000 1.776742000000
O 5.998208000000 -1.498252000000 -0.612387000000
C 3.740881000000 -2.884273000000 -0.259018000000
C 3.508212000000 -2.994179000000 -1.637868000000
C 3.116055000000 -3.735565000000 0.659026000000
H 4.028938000000 -2.324590000000 -2.337239000000
H 3.330552000000 -3.623678000000 1.731102000000
C 2.616054000000 -3.969742000000 -2.094545000000
C 2.231525000000 -4.711424000000 0.180749000000
H 2.425332000000 -4.064137000000 -3.175202000000
H 1.735761000000 -5.387063000000 0.895242000000
C 1.958923000000 -4.839210000000 -1.195898000000
C 0.963158000000 -5.852528000000 -1.697829000000
H -0.039480000000 -5.387679000000 -1.820928000000
H 1.253993000000 -6.258163000000 -2.686885000000
H 0.847342000000 -6.698892000000 -0.993321000000

RC$_{P\text{-}SS}$

Electronic energy = -3255.193994 Hartree

C -1.379779000000 -1.103704000000 -0.095506000000
O -1.209242000000 -1.525698000000 1.052955000000
C -2.347750000000 -0.067785000000 -0.343747000000
C -0.605512000000 -1.656969000000 -1.302147000000
H 0.598285000000 -1.806791000000 1.301996000000
H -1.322959000000 -1.569501000000 -2.148606000000
C -0.257366000000 -3.135267000000 -1.149299000000
C 0.961023000000 -3.678155000000 -1.603428000000
C -1.213896000000 -4.017505000000 -0.600708000000
H 1.734432000000 -3.034412000000 -2.042998000000
H -2.177054000000 -3.627389000000 -0.240022000000
C 1.219223000000 -5.054670000000 -1.500966000000
C -0.957686000000 -5.390395000000 -0.497563000000
H 2.182396000000 -5.450117000000 -1.857031000000
H -1.718873000000 -6.053708000000 -0.059701000000
C 0.264954000000 -5.916063000000 -0.944109000000
H 0.471875000000 -6.993303000000 -0.856585000000
C -3.194461000000 0.821037000000 -0.448970000000
C -4.130597000000 1.889597000000 -0.531591000000
C -3.664721000000 3.208673000000 -0.769532000000
C -5.515380000000 1.664099000000 -0.329885000000
H -2.590647000000 3.371551000000 -0.934023000000
H -5.877555000000 0.645857000000 -0.132743000000
C -4.569061000000 4.276225000000 -0.784351000000
C -6.407532000000 2.742525000000 -0.357571000000
H -4.198467000000 5.297705000000 -0.959080000000
H -7.478510000000 2.556765000000 -0.188390000000
C -5.940778000000 4.049042000000 -0.577949000000
H -6.647275000000 4.892629000000 -0.588406000000
C 6.141216000000 -1.203106000000 0.721766000000
C 4.825995000000 -1.316540000000 1.186978000000
C 6.427243000000 -0.852563000000 -0.619129000000
H 4.600444000000 -1.568185000000 2.230418000000
H 7.474579000000 -0.766903000000 -0.945905000000
C 3.775145000000 -1.069538000000 0.277513000000
C 5.398889000000 -0.619736000000 -1.543002000000
H 5.595260000000 -0.360318000000 -2.592629000000
C 4.092739000000 -0.746310000000 -1.061434000000
H 6.974222000000 -1.389722000000 1.416080000000
C 1.871217000000 -0.822550000000 -0.952432000000
C 2.318426000000 -1.106833000000 0.333616000000
O 2.951604000000 -0.617650000000 -1.800632000000
C 0.587121000000 -0.741777000000 -1.737808000000
H 0.879147000000 -1.152355000000 -2.729074000000
C 0.160584000000 0.700668000000 -2.029820000000
C 0.569804000000 1.777872000000 -1.225736000000
C -0.636463000000 0.964371000000 -3.162669000000
H 1.194772000000 1.592820000000 -0.339578000000
H -0.954625000000 0.131246000000 -3.810877000000
C 0.200641000000 3.092128000000 -1.554703000000
C -1.018834000000 2.272543000000 -3.485000000000
H 0.546232000000 3.921302000000 -0.920437000000
H -1.639047000000 2.458633000000 -4.374883000000
C -0.592047000000 3.345354000000 -2.683730000000
H -0.873705000000 4.376828000000 -2.945467000000
N 1.570029000000 -1.477966000000 1.459757000000
S 1.597008000000 -0.555594000000 2.906832000000
O 3.001691000000 -0.407831000000 3.335609000000

O	0.590686000000	-1.215775000000	3.760910000000
C	1.005200000000	1.087933000000	2.482717000000
C	1.921755000000	2.138440000000	2.329001000000
C	-0.374389000000	1.296714000000	2.334989000000
H	2.995889000000	1.944337000000	2.460160000000
H	-1.090828000000	0.469778000000	2.448745000000
C	1.434693000000	3.422763000000	2.037721000000
C	-0.838185000000	2.582610000000	2.046567000000
H	2.146229000000	4.256153000000	1.925667000000
H	-1.921579000000	2.747681000000	1.948485000000
C	0.052243000000	3.668093000000	1.903416000000
C	-0.486318000000	5.049065000000	1.632568000000
H	0.320938000000	5.776238000000	1.419538000000
H	-1.188373000000	5.042547000000	0.773133000000
H	-1.064192000000	5.421620000000	2.504729000000
P	-3.680619000000	0.630718000000	3.354779000000
C	-5.318825000000	0.066453000000	2.714813000000
C	-5.296897000000	-1.122551000000	1.951217000000
C	-6.543863000000	0.739671000000	2.895473000000
H	-4.335317000000	-1.638084000000	1.793721000000
H	-6.574020000000	1.671649000000	3.480086000000
C	-6.475960000000	-1.630838000000	1.387715000000
C	-7.721599000000	0.236416000000	2.319267000000
H	-6.445025000000	-2.559064000000	0.796554000000
H	-8.672273000000	0.772513000000	2.465944000000
C	-7.692154000000	-0.947575000000	1.564520000000
H	-8.616701000000	-1.339355000000	1.113703000000
C	-3.561309000000	-0.284297000000	4.955786000000
C	-4.669623000000	-0.495289000000	5.803797000000
C	-2.296085000000	-0.783911000000	5.328530000000
H	-5.662054000000	-0.118120000000	5.511634000000
H	-1.421412000000	-0.656671000000	4.671147000000
C	-4.511431000000	-1.186520000000	7.012937000000
C	-2.142972000000	-1.472284000000	6.543699000000
H	-5.381136000000	-1.348956000000	7.668417000000
H	-1.150641000000	-1.860063000000	6.819954000000
C	-3.246372000000	-1.674434000000	7.385859000000
H	-3.125043000000	-2.219958000000	8.334469000000
C	-3.997013000000	2.364891000000	3.902866000000
C	-4.438363000000	3.311308000000	2.947532000000
C	-3.631718000000	2.821035000000	5.186729000000
H	-4.716762000000	2.981673000000	1.935601000000
H	-3.280612000000	2.100257000000	5.940404000000
C	-4.524542000000	4.671228000000	3.274325000000
C	-3.707401000000	4.186299000000	5.506585000000
H	-4.879611000000	5.387377000000	2.516941000000
H	-3.415769000000	4.524052000000	6.513081000000
C	-4.154136000000	5.116413000000	4.555206000000
H	-4.215026000000	6.185334000000	4.810571000000

TS1$_{P\text{-}SS}$

Electronic energy = -3255.179309 Hartree

C	-0.956210000000	0.523850000000	-0.034573000000
O	-0.206742000000	0.369269000000	0.957601000000
C	-1.974398000000	1.497424000000	-0.007418000000
C	-0.779530000000	-0.302634000000	-1.309738000000
H	0.935154000000	-0.849170000000	0.768685000000
H	-1.606544000000	0.005904000000	-1.984423000000
C	-0.902852000000	-1.812098000000	-1.118016000000
C	-0.816738000000	-2.640096000000	-2.257091000000
C	-1.143698000000	-2.416177000000	0.130191000000
H	-0.636563000000	-2.190357000000	-3.245703000000
H	-1.214396000000	-1.806506000000	1.039551000000
C	-0.968046000000	-4.028761000000	-2.154421000000
C	-1.304180000000	-3.807129000000	0.233914000000
H	-0.892569000000	-4.653506000000	-3.057537000000
H	-1.494179000000	-4.250560000000	1.223125000000
C	-1.217366000000	-4.620026000000	-0.904542000000
H	-1.334871000000	-5.711076000000	-0.820378000000
C	-2.977116000000	2.224760000000	0.208534000000
C	-3.623000000000	3.514580000000	0.110493000000
C	-2.928921000000	4.517800000000	-0.619250000000
C	-4.867959000000	3.837543000000	0.692822000000
H	-1.958763000000	4.270143000000	-1.074733000000
H	-5.410123000000	3.064531000000	1.255123000000
C	-3.475877000000	5.797777000000	-0.752991000000
C	-5.406059000000	5.123864000000	0.553972000000
H	-2.925211000000	6.561890000000	-1.322984000000

H	-6.377427000000	5.355675000000	1.016325000000	C	2.708545000000	3.136919000000	2.089090000000
C	-4.716061000000	6.108927000000	-0.168838000000	H	5.663858000000	2.624291000000	0.436331000000
H	-5.143318000000	7.117104000000	-0.278748000000	H	2.049721000000	3.949373000000	2.433602000000
C	5.739159000000	-2.988816000000	-1.365283000000	C	3.905902000000	3.455179000000	1.415028000000
C	4.679869000000	-2.603778000000	-0.534341000000	C	4.309547000000	4.887612000000	1.173642000000
C	5.793136000000	-2.606405000000	-2.726227000000	H	4.585412000000	5.056358000000	0.112412000000
H	4.642785000000	-2.890457000000	0.523754000000	H	3.497082000000	5.592424000000	1.436126000000
H	6.644494000000	-2.924168000000	-3.347022000000	H	5.198186000000	5.159768000000	1.781709000000
C	3.649204000000	-1.811389000000	-1.087650000000	P	-4.694940000000	0.520304000000	1.000937000000
C	4.773503000000	-1.834884000000	-3.300709000000	C	-3.860425000000	-0.545183000000	2.249139000000
H	4.784151000000	-1.535719000000	-4.358161000000	C	-2.592690000000	-0.183241000000	2.749872000000
C	3.719959000000	-1.465802000000	-2.459141000000	C	-4.451484000000	-1.756655000000	2.677360000000
H	6.549563000000	-3.606113000000	-0.949059000000	H	-2.121535000000	0.758739000000	2.441434000000
C	1.815024000000	-0.602906000000	-1.705786000000	H	-5.455460000000	-2.033680000000	2.320843000000
C	2.404798000000	-1.224023000000	-0.606614000000	C	-1.892235000000	-1.048199000000	3.606674000000
O	2.616692000000	-0.759437000000	-2.830974000000	C	-3.760316000000	-2.605747000000	3.553213000000
C	0.536681000000	0.106275000000	-2.072091000000	H	-0.872323000000	-0.786216000000	3.926398000000
H	0.366947000000	-0.229505000000	-3.116682000000	H	-4.226271000000	-3.549075000000	3.877059000000
C	0.649250000000	1.628052000000	-2.144491000000	C	-2.473559000000	-2.261907000000	4.003565000000
C	1.478828000000	2.358542000000	-1.275159000000	H	-1.920781000000	-2.943404000000	4.667856000000
C	-0.158216000000	2.329015000000	-3.062655000000	C	-6.474086000000	0.378791000000	1.460168000000
H	2.118139000000	1.825685000000	-0.556147000000	C	-7.472104000000	-0.029153000000	0.550737000000
H	-0.810077000000	1.765774000000	-3.750620000000	C	-6.854229000000	0.804826000000	2.754189000000
C	1.493127000000	3.761348000000	-1.320500000000	H	-7.189815000000	-0.364522000000	-0.458372000000
C	-0.143327000000	3.729853000000	-3.111548000000	H	-6.082814000000	1.126673000000	3.472439000000
H	2.145136000000	4.315765000000	-0.630492000000	C	-8.822439000000	-0.021376000000	0.934950000000
H	-0.777087000000	4.259607000000	-3.839285000000	C	-8.202419000000	0.804274000000	3.133531000000
C	0.683704000000	4.452234000000	-2.235192000000	H	-9.591792000000	-0.349742000000	0.219347000000
H	0.701253000000	5.552347000000	-2.270107000000	H	-8.483920000000	1.127960000000	4.147252000000
N	1.850418000000	-1.356360000000	0.674318000000	C	-9.191870000000	0.392808000000	2.223718000000
S	2.684212000000	-0.930466000000	2.110008000000	H	-10.251214000000	0.395068000000	2.521572000000
O	3.915380000000	-1.737242000000	2.218730000000	C	-4.588342000000	-0.401852000000	-0.590687000000
O	1.639810000000	-0.982775000000	3.152576000000	C	-4.304932000000	-1.777327000000	-0.680703000000
C	3.179715000000	0.779907000000	1.876517000000	C	-4.792582000000	0.341353000000	-1.774986000000
C	4.379113000000	1.060450000000	1.201195000000	H	-4.115925000000	-2.364849000000	0.228120000000
C	2.333639000000	1.806581000000	2.319000000000	H	-4.992557000000	1.422722000000	-1.713663000000
H	5.027025000000	0.235621000000	0.870609000000	C	-4.228976000000	-2.402926000000	-1.935233000000
H	1.389737000000	1.552929000000	2.820011000000	C	-4.723626000000	-0.287820000000	-3.025799000000
C	4.727923000000	2.396446000000	0.970497000000	H	-3.977852000000	-3.472232000000	-1.989938000000

H	-4.884242000000	0.300156000000	-3.942415000000
C	-4.437442000000	-1.661814000000	-3.107329000000
H	-4.366730000000	-2.153117000000	-4.089720000000

I*$_{P-SS}$*

Electronic energy = -3255.202568 Hartree

C	-1.159799000000	0.421723000000	-0.407623000000
O	-0.321485000000	0.296770000000	0.561852000000
C	-2.330679000000	1.096080000000	-0.235169000000
C	-0.816173000000	-0.111971000000	-1.796728000000
H	0.816071000000	-0.709303000000	0.373796000000
H	-1.580210000000	0.306791000000	-2.485803000000
C	-0.881890000000	-1.627726000000	-1.927226000000
C	-0.859488000000	-2.197267000000	-3.218691000000
C	-0.974277000000	-2.489061000000	-0.817229000000
H	-0.794038000000	-1.538462000000	-4.099731000000
H	-0.984713000000	-2.074461000000	0.199368000000
C	-0.916002000000	-3.585569000000	-3.398945000000
C	-1.033495000000	-3.879365000000	-0.996593000000
H	-0.889370000000	-4.006779000000	-4.415774000000
H	-1.098724000000	-4.533044000000	-0.113561000000
C	-1.001775000000	-4.434892000000	-2.283558000000
H	-1.038866000000	-5.526655000000	-2.419116000000
C	-3.517498000000	1.566325000000	0.117844000000
C	-3.951669000000	2.988863000000	0.056997000000
C	-2.964359000000	3.989386000000	0.212600000000
C	-5.277469000000	3.381286000000	-0.226697000000
H	-1.925115000000	3.679667000000	0.402078000000
H	-6.053007000000	2.621199000000	-0.395144000000
C	-3.300134000000	5.343329000000	0.103240000000
C	-5.611252000000	4.740627000000	-0.329807000000
H	-2.516209000000	6.106896000000	0.223182000000
H	-6.649242000000	5.028812000000	-0.556299000000
C	-4.627128000000	5.726182000000	-0.159991000000
H	-4.891210000000	6.791599000000	-0.241192000000
C	5.697516000000	-2.743128000000	-1.788685000000
C	4.608970000000	-2.444656000000	-0.959644000000
C	5.807608000000	-2.207148000000	-3.093217000000
H	4.527063000000	-2.854243000000	0.054804000000
H	6.680052000000	-2.460805000000	-3.714338000000
C	3.604546000000	-1.582154000000	-1.454796000000
C	4.816177000000	-1.362558000000	-3.611415000000
H	4.869933000000	-0.942068000000	-4.625481000000
C	3.732823000000	-1.080231000000	-2.773904000000
H	6.486420000000	-3.413964000000	-1.415974000000
C	1.810028000000	-0.280240000000	-1.998005000000
C	2.349576000000	-1.033733000000	-0.956026000000
O	2.655241000000	-0.317813000000	-3.102808000000
C	0.551679000000	0.486566000000	-2.312038000000
H	0.492152000000	0.396964000000	-3.416976000000
C	0.598725000000	1.984427000000	-2.014917000000
C	1.332851000000	2.515289000000	-0.939822000000
C	-0.195138000000	2.855805000000	-2.787767000000
H	1.954500000000	1.850026000000	-0.325258000000
H	-0.771090000000	2.452635000000	-3.637078000000
C	1.262453000000	3.884776000000	-0.640249000000
C	-0.268658000000	4.223589000000	-2.490159000000
H	1.837472000000	4.277696000000	0.210895000000
H	-0.899251000000	4.885696000000	-3.102909000000
C	0.460856000000	4.742952000000	-1.408805000000
H	0.406698000000	5.816167000000	-1.168709000000
N	1.720272000000	-1.286264000000	0.269785000000
S	2.478011000000	-1.158659000000	1.783231000000
O	3.636382000000	-2.072994000000	1.850092000000
O	1.367212000000	-1.278517000000	2.752492000000
C	3.110900000000	0.521631000000	1.851309000000
C	4.336063000000	0.819194000000	1.232670000000
C	2.336905000000	1.524430000000	2.453334000000
H	4.933435000000	0.013338000000	0.781809000000
H	1.375873000000	1.260353000000	2.915693000000
C	4.778460000000	2.147638000000	1.210881000000
C	2.807610000000	2.843536000000	2.437777000000
H	5.735422000000	2.389169000000	0.721950000000
H	2.210936000000	3.634826000000	2.918755000000
C	4.025495000000	3.180504000000	1.809188000000
C	4.490435000000	4.612409000000	1.737175000000
H	4.175259000000	5.077150000000	0.777229000000
H	4.062797000000	5.224670000000	2.555087000000

H	5.594726000000	4.688468000000	1.784598000000
P	-4.512750000000	0.239686000000	0.921594000000
C	-3.799873000000	-0.252779000000	2.529332000000
C	-2.399482000000	-0.292281000000	2.699798000000
C	-4.651066000000	-0.744036000000	3.546203000000
H	-1.691626000000	0.073441000000	1.933564000000
H	-5.743344000000	-0.726187000000	3.418478000000
C	-1.857815000000	-0.835942000000	3.875443000000
C	-4.096872000000	-1.263858000000	4.725027000000
H	-0.761506000000	-0.896013000000	3.964072000000
H	-4.762986000000	-1.638956000000	5.516821000000
C	-2.702374000000	-1.317246000000	4.888013000000
H	-2.273280000000	-1.742514000000	5.808196000000
C	-6.267433000000	0.621106000000	1.236738000000
C	-7.289238000000	0.040628000000	0.457716000000
C	-6.587782000000	1.600088000000	2.204691000000
H	-7.038996000000	-0.723078000000	-0.293783000000
H	-5.791351000000	2.062776000000	2.807193000000
C	-8.621402000000	0.438364000000	0.647330000000
C	-7.920429000000	1.987251000000	2.389035000000
H	-9.417485000000	-0.018867000000	0.040714000000
H	-8.166509000000	2.749471000000	3.143388000000
C	-8.938143000000	1.410446000000	1.609387000000
H	-9.984049000000	1.719845000000	1.755092000000
C	-4.446028000000	-1.218069000000	-0.163192000000
C	-4.512655000000	-2.508649000000	0.398969000000
C	-4.360885000000	-1.057508000000	-1.561051000000
H	-4.559488000000	-2.632929000000	1.491135000000
H	-4.293065000000	-0.048907000000	-1.994790000000
C	-4.485298000000	-3.632335000000	-0.438530000000
C	-4.324881000000	-2.187840000000	-2.388357000000
H	-4.517797000000	-4.639813000000	0.002008000000
H	-4.228592000000	-2.064072000000	-3.476727000000
C	-4.384808000000	-3.473945000000	-1.829073000000
H	-4.330546000000	-4.357697000000	-2.481177000000

II$_{P\text{-}SS}$

Electronic energy = -3255.189464 Hartree

C	1.119050000000	3.779055000000	2.874806000000
O	0.624066000000	3.838226000000	4.148667000000
C	2.293017000000	4.277316000000	2.505801000000
C	0.220831000000	3.082716000000	1.872911000000
H	1.268058000000	4.312352000000	4.708607000000
H	0.812572000000	2.994128000000	0.942371000000
C	-1.030194000000	3.883120000000	1.557080000000
C	-1.962343000000	4.255515000000	2.545912000000
C	-1.284015000000	4.232326000000	0.214766000000
H	-1.773279000000	3.987632000000	3.595016000000
H	-0.570174000000	3.910145000000	-0.560270000000
C	-3.120557000000	4.966972000000	2.199557000000
C	-2.443388000000	4.943071000000	-0.131653000000
H	-3.843900000000	5.246385000000	2.981509000000
H	-2.633742000000	5.196029000000	-1.186155000000
C	-3.366051000000	5.313108000000	0.860285000000
H	-4.281804000000	5.861061000000	0.589093000000
C	3.415244000000	4.809227000000	2.034782000000
C	4.701359000000	4.080757000000	1.902969000000
C	5.106080000000	3.214582000000	2.940665000000
C	5.465316000000	4.147713000000	0.719579000000
H	4.490175000000	3.145572000000	3.849551000000
H	5.123976000000	4.757603000000	-0.127696000000
C	6.251184000000	2.425175000000	2.797071000000
C	6.611591000000	3.354083000000	0.580480000000
H	6.544197000000	1.736515000000	3.603347000000
H	7.177068000000	3.386755000000	-0.362464000000
C	7.008290000000	2.493365000000	1.614794000000
H	7.902194000000	1.862349000000	1.496105000000
C	4.947336000000	-1.840830000000	2.366506000000
C	4.510443000000	-0.653193000000	1.764628000000
C	4.114184000000	-2.561418000000	3.256508000000
H	5.143654000000	-0.081602000000	1.071291000000
H	4.484798000000	-3.493401000000	3.710330000000
C	3.213611000000	-0.195985000000	2.061843000000
C	2.822145000000	-2.110894000000	3.575526000000
H	2.165465000000	-2.657767000000	4.267329000000
C	2.405063000000	-0.925505000000	2.962317000000
H	5.954651000000	-2.225790000000	2.144141000000
C	1.215129000000	0.832416000000	2.317615000000

C	2.420470000000	0.950632000000	1.624455000000
O	1.200559000000	-0.300305000000	3.126926000000
C	-0.058315000000	1.625752000000	2.356277000000
H	-0.383974000000	1.673098000000	3.417537000000
C	-1.180092000000	0.944134000000	1.577816000000
C	-1.103301000000	0.790861000000	0.178317000000
C	-2.306905000000	0.449124000000	2.258059000000
H	-0.233709000000	1.189231000000	-0.373183000000
H	-2.371502000000	0.569833000000	3.351348000000
C	-2.138910000000	0.150161000000	-0.517317000000
C	-3.340850000000	-0.196453000000	1.561202000000
H	-2.067542000000	0.041405000000	-1.610780000000
H	-4.214120000000	-0.581989000000	2.110035000000
C	-3.259066000000	-0.348453000000	0.169074000000
H	-4.068050000000	-0.853581000000	-0.381107000000
N	2.898477000000	1.954801000000	0.792731000000
S	2.762368000000	1.884600000000	-0.796159000000
O	3.813557000000	2.755948000000	-1.408926000000
O	1.359960000000	2.176262000000	-1.303439000000
C	3.127731000000	0.184747000000	-1.282809000000
C	2.203894000000	-0.841424000000	-1.024139000000
C	4.412307000000	-0.114365000000	-1.758492000000
H	1.194560000000	-0.599833000000	-0.661500000000
H	5.106703000000	0.716116000000	-1.953950000000
C	2.590763000000	-2.173358000000	-1.212995000000
C	4.778849000000	-1.452461000000	-1.955878000000
H	1.872588000000	-2.978966000000	-0.991709000000
H	5.787552000000	-1.690413000000	-2.330971000000
C	3.885120000000	-2.504121000000	-1.666658000000
C	4.306130000000	-3.945574000000	-1.800809000000
H	3.498889000000	-4.570606000000	-2.233454000000
H	5.206429000000	-4.053687000000	-2.437050000000
H	4.548660000000	-4.375664000000	-0.804870000000
P	3.158524000000	6.489209000000	1.347723000000
C	3.002213000000	6.418567000000	-0.451405000000
C	2.366054000000	5.288091000000	-0.998801000000
C	3.464556000000	7.463263000000	-1.281430000000
H	2.040487000000	4.455271000000	-0.361625000000
H	3.978051000000	8.335839000000	-0.850361000000
C	2.188296000000	5.194487000000	-2.385702000000
C	3.268918000000	7.364811000000	-2.664815000000
H	1.736211000000	4.273249000000	-2.781261000000
H	3.627582000000	8.170577000000	-3.322853000000
C	2.632046000000	6.235015000000	-3.213781000000
H	2.501248000000	6.161180000000	-4.304188000000
C	1.585746000000	7.070350000000	2.048014000000
C	0.441875000000	7.201637000000	1.240926000000
C	1.511227000000	7.309075000000	3.438113000000
H	0.498285000000	6.996271000000	0.162420000000
H	2.411120000000	7.208594000000	4.064984000000
C	-0.779942000000	7.566013000000	1.826925000000
C	0.286796000000	7.668855000000	4.013015000000
H	-1.680028000000	7.637077000000	1.200551000000
H	0.223366000000	7.851382000000	5.096365000000
C	-0.859321000000	7.792170000000	3.207390000000
H	-1.824677000000	8.057915000000	3.663290000000
C	4.434232000000	7.702740000000	1.809543000000
C	5.776455000000	7.319524000000	2.015212000000
C	4.060084000000	9.060162000000	1.927285000000
H	6.073668000000	6.264865000000	1.925666000000
H	3.011403000000	9.359114000000	1.779114000000
C	6.732154000000	8.294324000000	2.336972000000
C	5.026375000000	10.025332000000	2.242977000000
H	7.776834000000	7.991279000000	2.501827000000
H	4.731427000000	11.081534000000	2.332751000000
C	6.362008000000	9.643653000000	2.448586000000
H	7.117833000000	10.402855000000	2.700381000000

III$_{P\text{-}SS}$

Electronic energy = -3255.219741 Hartree

C	0.472398000000	3.334097000000	1.079487000000
O	0.361344000000	4.069594000000	0.081234000000
C	1.495772000000	3.654124000000	2.072227000000
C	-0.492053000000	2.166442000000	1.244450000000
H	1.675847000000	3.003754000000	2.938623000000
H	-0.702053000000	1.857096000000	0.203185000000
C	-1.767640000000	2.703142000000	1.869991000000
C	-1.849729000000	2.983073000000	3.249524000000

C	-2.867842000000	3.024785000000	1.051245000000
H	-0.994909000000	2.756801000000	3.906165000000
H	-2.805399000000	2.823176000000	-0.028783000000
C	-3.009038000000	3.548923000000	3.800364000000
C	-4.024708000000	3.598217000000	1.597305000000
H	-3.058292000000	3.750160000000	4.881671000000
H	-4.875552000000	3.839427000000	0.941921000000
C	-4.101324000000	3.858996000000	2.974973000000
H	-5.012674000000	4.301686000000	3.405367000000
C	2.233635000000	4.817046000000	1.961867000000
C	3.423567000000	5.030576000000	2.823667000000
C	3.489627000000	6.034558000000	3.816397000000
C	4.525418000000	4.157807000000	2.666265000000
H	2.637343000000	6.714481000000	3.959131000000
H	4.486689000000	3.378652000000	1.891148000000
C	4.627317000000	6.164053000000	4.623083000000
C	5.661770000000	4.289328000000	3.476725000000
H	4.659795000000	6.949006000000	5.393796000000
H	6.502322000000	3.592739000000	3.336464000000
C	5.718585000000	5.294784000000	4.454148000000
H	6.611383000000	5.400374000000	5.089152000000
C	6.156823000000	0.855786000000	0.761710000000
C	4.963783000000	0.976051000000	0.035810000000
C	6.153518000000	0.654326000000	2.161185000000
H	4.958598000000	1.155598000000	-1.047547000000
H	7.108609000000	0.553834000000	2.699163000000
C	3.739518000000	0.897305000000	0.735232000000
C	4.949812000000	0.587779000000	2.880363000000
H	4.921223000000	0.454288000000	3.970713000000
C	3.772767000000	0.719992000000	2.141500000000
H	7.117915000000	0.919759000000	0.229747000000
C	1.636499000000	0.946537000000	1.619617000000
C	2.313410000000	0.991953000000	0.374408000000
O	2.508967000000	0.736800000000	2.671328000000
C	0.179533000000	0.982132000000	1.977011000000
H	0.154618000000	1.166099000000	3.071364000000
C	-0.538351000000	-0.338830000000	1.720538000000
C	-0.745842000000	-0.793056000000	0.399937000000
C	-0.997848000000	-1.124822000000	2.792788000000

H	-0.366301000000	-0.190895000000	-0.442622000000
H	-0.842857000000	-0.773678000000	3.825511000000
C	-1.396884000000	-2.013261000000	0.167177000000
C	-1.651604000000	-2.344798000000	2.558239000000
H	-1.546537000000	-2.357929000000	-0.867658000000
H	-2.008252000000	-2.946611000000	3.408292000000
C	-1.851686000000	-2.793117000000	1.243885000000
H	-2.363926000000	-3.749519000000	1.057742000000
N	1.629460000000	1.077059000000	-0.788456000000
S	2.308886000000	0.779079000000	-2.247937000000
O	3.476566000000	1.670789000000	-2.573756000000
O	1.198921000000	0.734397000000	-3.223223000000
C	3.013737000000	-0.888728000000	-2.118115000000
C	2.348426000000	-1.889154000000	-1.394304000000
C	4.205617000000	-1.174829000000	-2.799839000000
H	1.415977000000	-1.659575000000	-0.859330000000
H	4.708356000000	-0.370787000000	-3.356984000000
C	2.892459000000	-3.179218000000	-1.347664000000
C	4.733153000000	-2.471455000000	-2.749670000000
H	2.371452000000	-3.961349000000	-0.772569000000
H	5.668764000000	-2.695994000000	-3.286854000000
C	4.091689000000	-3.494283000000	-2.020030000000
C	4.690629000000	-4.875326000000	-1.933666000000
H	3.919472000000	-5.643709000000	-1.728791000000
H	5.216680000000	-5.152243000000	-2.868947000000
H	5.437029000000	-4.933374000000	-1.111869000000
P	1.732989000000	6.081925000000	0.746663000000
C	2.650600000000	7.587892000000	1.277448000000
C	4.049275000000	7.616277000000	1.087733000000
C	2.007068000000	8.687281000000	1.876058000000
H	4.557423000000	6.764761000000	0.610448000000
H	0.916072000000	8.678693000000	2.014632000000
C	4.794383000000	8.721809000000	1.514003000000
C	2.759019000000	9.796406000000	2.297470000000
H	5.885250000000	8.731164000000	1.371541000000
H	2.249773000000	10.653669000000	2.763081000000
C	4.150806000000	9.812208000000	2.123589000000
H	4.737859000000	10.681047000000	2.457931000000
C	2.255835000000	5.932877000000	-0.995579000000

C	2.325743000000	7.127694000000	-1.748408000000
C	2.580605000000	4.700170000000	-1.591140000000
H	2.094677000000	8.096649000000	-1.280507000000
H	2.516186000000	3.755829000000	-1.034199000000
C	2.696997000000	7.076968000000	-3.098716000000
C	2.969431000000	4.662691000000	-2.938648000000
H	2.738787000000	8.006976000000	-3.685794000000
H	3.211853000000	3.677967000000	-3.366437000000
C	3.020191000000	5.845201000000	-3.691930000000
H	3.315320000000	5.807776000000	-4.751823000000
C	-0.031039000000	6.516650000000	0.929391000000
C	-0.613862000000	6.460881000000	2.211252000000
C	-0.814550000000	6.842634000000	-0.193100000000
H	-0.006569000000	6.174322000000	3.083298000000
H	-0.365062000000	6.846190000000	-1.196699000000
C	-1.979561000000	6.738093000000	2.366044000000
C	-2.177209000000	7.125177000000	-0.026002000000
H	-2.438577000000	6.670121000000	3.362869000000
H	-2.791285000000	7.371307000000	-0.905236000000
C	-2.761735000000	7.070314000000	1.249505000000
H	-3.836235000000	7.271908000000	1.372908000000

TS2$_{P\text{-}SS}$

Electronic energy = -3255.197478 Hartree

C	0.182189000000	3.407440000000	0.903431000000
O	0.068244000000	4.116796000000	-0.090330000000
C	1.252730000000	3.606558000000	1.910939000000
C	-0.655766000000	2.148519000000	1.120150000000
H	1.000938000000	3.348254000000	2.957765000000
H	-0.800528000000	1.740109000000	0.102198000000
C	-1.992240000000	2.575340000000	1.698861000000
C	-2.194944000000	2.761959000000	3.080559000000
C	-3.032258000000	2.928811000000	0.813312000000
H	-1.396158000000	2.509325000000	3.794974000000
H	-2.875089000000	2.809030000000	-0.269623000000
C	-3.407323000000	3.275550000000	3.566297000000
C	-4.241212000000	3.447729000000	1.295434000000
H	-3.547845000000	3.406608000000	4.650251000000
H	-5.039181000000	3.718678000000	0.587358000000
C	-4.434407000000	3.622799000000	2.675547000000
H	-5.384879000000	4.026796000000	3.056017000000
C	2.143504000000	4.711843000000	1.753859000000
C	3.282199000000	4.890276000000	2.707794000000
C	3.125061000000	5.567453000000	3.937533000000
C	4.562219000000	4.386810000000	2.386735000000
H	2.136442000000	5.977810000000	4.196389000000
H	4.701067000000	3.858012000000	1.432146000000
C	4.206588000000	5.742545000000	4.812374000000
C	5.646695000000	4.557199000000	3.260622000000
H	4.061535000000	6.281877000000	5.761142000000
H	6.631328000000	4.147230000000	2.987607000000
C	5.473784000000	5.238929000000	4.474523000000
H	6.324938000000	5.379250000000	5.158401000000
C	6.193243000000	0.879999000000	1.019254000000
C	5.036116000000	1.040561000000	0.248439000000
C	6.136942000000	0.878968000000	2.432304000000
H	5.069985000000	1.078718000000	-0.847627000000
H	7.064317000000	0.749756000000	3.011160000000
C	3.799965000000	1.205152000000	0.912834000000
C	4.925518000000	1.056596000000	3.114930000000
H	4.866513000000	1.089413000000	4.211243000000
C	3.779264000000	1.221552000000	2.332606000000
H	7.164523000000	0.758345000000	0.517335000000
C	1.675472000000	1.662062000000	1.734450000000
C	2.409142000000	1.383693000000	0.496733000000
O	2.528871000000	1.420495000000	2.832526000000
C	0.236346000000	1.193313000000	1.943543000000
H	0.044709000000	1.364458000000	3.025625000000
C	0.012865000000	-0.291588000000	1.685031000000
C	-1.114022000000	-0.759974000000	0.982087000000
C	0.933825000000	-1.239756000000	2.180841000000
H	-1.856577000000	-0.048557000000	0.594334000000
H	1.816259000000	-0.894959000000	2.738060000000
C	-1.313780000000	-2.135125000000	0.775511000000
C	0.736729000000	-2.611089000000	1.974332000000
H	-2.201912000000	-2.476655000000	0.222336000000
H	1.473156000000	-3.330463000000	2.363796000000
C	-0.390374000000	-3.066779000000	1.270857000000

H	-0.548262000000	-4.144414000000	1.111036000000
N	1.728357000000	1.351228000000	-0.636697000000
S	2.394078000000	0.975130000000	-2.126246000000
O	3.680833000000	1.691811000000	-2.395493000000
O	1.293762000000	1.126587000000	-3.096560000000
C	2.783015000000	-0.778675000000	-1.972221000000
C	1.815655000000	-1.661248000000	-1.462590000000
C	4.024701000000	-1.247008000000	-2.420092000000
H	0.846279000000	-1.279926000000	-1.109591000000
H	4.756307000000	-0.532452000000	-2.824142000000
C	2.115538000000	-3.025074000000	-1.391937000000
C	4.304830000000	-2.619285000000	-2.345236000000
H	1.362726000000	-3.715025000000	-0.980607000000
H	5.280417000000	-2.992604000000	-2.694834000000
C	3.360943000000	-3.528719000000	-1.829270000000
C	3.664036000000	-5.002125000000	-1.730854000000
H	2.889756000000	-5.608032000000	-2.245452000000
H	4.645735000000	-5.250411000000	-2.178108000000
H	3.679837000000	-5.336829000000	-0.672057000000
P	1.784556000000	6.040332000000	0.645823000000
C	2.819371000000	7.428763000000	1.262165000000
C	4.203797000000	7.407098000000	0.992458000000
C	2.280841000000	8.458606000000	2.055799000000
H	4.626591000000	6.612395000000	0.360307000000
H	1.200846000000	8.487329000000	2.260897000000
C	5.040964000000	8.389796000000	1.535786000000
C	3.122444000000	9.448188000000	2.587806000000
H	6.121909000000	8.357769000000	1.332691000000
H	2.694589000000	10.252455000000	3.205314000000
C	4.502133000000	9.409975000000	2.337006000000
H	5.161341000000	10.181886000000	2.762487000000
C	2.235148000000	5.967228000000	-1.128677000000
C	2.356452000000	7.169669000000	-1.859678000000
C	2.504741000000	4.736896000000	-1.749837000000
H	2.180455000000	8.139300000000	-1.368392000000
H	2.425572000000	3.799152000000	-1.184704000000
C	2.716780000000	7.126362000000	-3.213954000000
C	2.873370000000	4.699088000000	-3.102765000000
H	2.806030000000	8.063559000000	-3.784421000000
H	3.081683000000	3.719829000000	-3.556782000000
C	2.971902000000	5.891612000000	-3.835417000000
H	3.258058000000	5.861606000000	-4.898031000000
C	0.048121000000	6.630692000000	0.802167000000
C	-0.579814000000	6.500033000000	2.057245000000
C	-0.684236000000	7.115588000000	-0.296930000000
H	-0.014293000000	6.084993000000	2.905799000000
H	-0.213544000000	7.180917000000	-1.288287000000
C	-1.928379000000	6.852029000000	2.210662000000
C	-2.030735000000	7.473331000000	-0.135394000000
H	-2.418033000000	6.723458000000	3.187564000000
H	-2.601074000000	7.841658000000	-1.001609000000
C	-2.656751000000	7.338899000000	1.114076000000
H	-3.719277000000	7.600358000000	1.230831000000

IV*P-SS*

Electronic energy = -3255.21587 Hartree

C	-0.282871000000	3.651059000000	0.909125000000
O	-0.636006000000	4.618714000000	0.258847000000
C	0.928209000000	3.628075000000	1.822877000000
C	-0.958390000000	2.271504000000	0.888141000000
H	0.439391000000	3.835590000000	2.821163000000
H	-0.707191000000	1.923806000000	-0.140423000000
C	-2.464771000000	2.305298000000	1.049925000000
C	-3.125776000000	1.592525000000	2.069518000000
C	-3.238337000000	3.071124000000	0.149948000000
H	-2.552539000000	0.968536000000	2.770640000000
H	-2.730105000000	3.639525000000	-0.641544000000
C	-4.523074000000	1.649520000000	2.194324000000
C	-4.632238000000	3.131630000000	0.280024000000
H	-5.019886000000	1.080947000000	2.995347000000
H	-5.217559000000	3.738757000000	-0.427541000000
C	-5.281752000000	2.420759000000	1.302363000000
H	-6.377227000000	2.464206000000	1.399678000000
C	2.071865000000	4.558609000000	1.507108000000
C	3.249811000000	4.533147000000	2.417403000000
C	3.103532000000	4.558905000000	3.828960000000
C	4.572869000000	4.480463000000	1.911282000000
H	2.092639000000	4.566209000000	4.267127000000

H	4.706997000000	4.381674000000	0.824720000000	C	4.499973000000	-0.734926000000	-2.042391000000
C	4.214947000000	4.579680000000	4.681899000000	H	1.333035000000	-1.046091000000	-0.735783000000
C	5.687083000000	4.525384000000	2.759969000000	H	5.164871000000	0.036209000000	-2.458755000000
H	4.063459000000	4.611050000000	5.772685000000	C	2.773820000000	-2.658439000000	-0.953257000000
H	6.699251000000	4.494975000000	2.327445000000	C	4.912189000000	-2.069380000000	-1.926255000000
C	5.517313000000	4.580087000000	4.152452000000	H	2.090645000000	-3.403175000000	-0.516475000000
H	6.390841000000	4.606184000000	4.821578000000	H	5.920298000000	-2.354977000000	-2.265575000000
C	5.783371000000	0.859624000000	1.891502000000	C	4.062655000000	-3.050636000000	-1.376431000000
C	4.781506000000	1.105544000000	0.950460000000	C	4.510740000000	-4.481872000000	-1.228834000000
C	5.501536000000	0.903730000000	3.276180000000	H	3.802763000000	-5.177695000000	-1.724629000000
H	5.001764000000	1.130151000000	-0.122218000000	H	5.514515000000	-4.646282000000	-1.665313000000
H	6.312513000000	0.718585000000	3.997043000000	H	4.553133000000	-4.777624000000	-0.159425000000
C	3.478076000000	1.393902000000	1.411897000000	P	1.805866000000	6.035453000000	0.623111000000
C	4.222289000000	1.205651000000	3.758751000000	C	3.317036000000	7.063747000000	0.817663000000
H	4.001751000000	1.281207000000	4.831388000000	C	4.337164000000	6.976638000000	-0.151939000000
C	3.227382000000	1.458442000000	2.807663000000	C	3.511074000000	7.865290000000	1.958440000000
H	6.807834000000	0.648270000000	1.552762000000	H	4.192618000000	6.350095000000	-1.044271000000
C	1.253089000000	2.109895000000	1.891789000000	H	2.721232000000	7.946910000000	2.717784000000
C	2.218278000000	1.707888000000	0.763398000000	C	5.539087000000	7.676432000000	0.025539000000
O	1.953266000000	1.792627000000	3.120327000000	C	4.716695000000	8.559046000000	2.134123000000
C	-0.142743000000	1.420890000000	1.876355000000	H	6.331396000000	7.598530000000	-0.734295000000
H	-0.532941000000	1.606373000000	2.902059000000	H	4.862002000000	9.177420000000	3.032649000000
C	-0.140611000000	-0.078260000000	1.642472000000	C	5.733229000000	8.463801000000	1.170851000000
C	-0.885136000000	-0.669951000000	0.601812000000	H	6.679325000000	9.008334000000	1.312292000000
C	0.597835000000	-0.925095000000	2.498663000000	C	1.599341000000	5.989591000000	-1.201208000000
H	-1.484334000000	-0.042490000000	-0.072361000000	C	1.600763000000	7.179920000000	-1.960414000000
H	1.167361000000	-0.485180000000	3.329421000000	C	1.547649000000	4.747861000000	-1.853801000000
C	-0.889117000000	-2.062213000000	0.418162000000	H	1.691214000000	8.157583000000	-1.462275000000
C	0.605829000000	-2.313667000000	2.307913000000	H	1.573527000000	3.825336000000	-1.259838000000
H	-1.484130000000	-2.498676000000	-0.398295000000	C	1.522922000000	7.114570000000	-3.359204000000
H	1.195157000000	-2.951155000000	2.984568000000	C	1.479154000000	4.683230000000	-3.253281000000
C	-0.141406000000	-2.890555000000	1.268208000000	H	1.525327000000	8.043853000000	-3.949443000000
H	-0.148279000000	-3.982297000000	1.127422000000	H	1.456015000000	3.693196000000	-3.732819000000
N	1.739669000000	1.673431000000	-0.446462000000	C	1.459766000000	5.867478000000	-4.005430000000
S	2.683232000000	1.325619000000	-1.826996000000	H	1.408208000000	5.821504000000	-5.104194000000
O	3.895082000000	2.186687000000	-1.879993000000	C	0.428273000000	7.065053000000	1.310647000000
O	1.711670000000	1.367888000000	-2.939137000000	C	0.297927000000	7.064311000000	2.715352000000
C	3.215803000000	-0.379299000000	-1.607532000000	C	-0.514191000000	7.762240000000	0.531405000000
C	2.338870000000	-1.334416000000	-1.071878000000	H	1.003435000000	6.473347000000	3.320888000000

H	-0.459006000000	7.723334000000	-0.565343000000
C	-0.733850000000	7.786481000000	3.333523000000
C	-1.548580000000	8.476790000000	1.152663000000
H	-0.823161000000	7.781858000000	4.430648000000
H	-2.284558000000	9.014002000000	0.534949000000
C	-1.657329000000	8.498651000000	2.552628000000
H	-2.473210000000	9.058437000000	3.034602000000

V*P-SS*

Electronic energy = -3255.236207 Hartree

C	-0.099290000000	3.724575000000	0.985024000000
O	-0.479347000000	4.824627000000	0.451255000000
C	1.075670000000	3.583104000000	1.700141000000
C	-0.807876000000	2.377555000000	0.812285000000
H	1.709508000000	5.269466000000	2.901993000000
H	-0.568914000000	2.053408000000	-0.225452000000
C	-2.315729000000	2.444334000000	0.965226000000
C	-3.025186000000	1.661737000000	1.897473000000
C	-3.044664000000	3.325614000000	0.135714000000
H	-2.490787000000	0.952321000000	2.546282000000
H	-2.494558000000	3.956119000000	-0.577312000000
C	-4.422611000000	1.757987000000	2.003392000000
C	-4.437716000000	3.426155000000	0.246314000000
H	-4.956125000000	1.131987000000	2.735377000000
H	-4.985454000000	4.125601000000	-0.404243000000
C	-5.134931000000	2.641188000000	1.179841000000
H	-6.230138000000	2.716388000000	1.262242000000
C	1.929549000000	4.789857000000	1.916396000000
C	3.432840000000	4.608604000000	1.855409000000
C	4.208744000000	4.899531000000	2.995106000000
C	4.086251000000	4.139389000000	0.699319000000
H	3.709639000000	5.272000000000	3.903455000000
H	3.516995000000	3.891872000000	-0.204240000000
C	5.598911000000	4.718893000000	2.984234000000
C	5.475182000000	3.957089000000	0.684643000000
H	6.185792000000	4.946630000000	3.887140000000
H	5.944005000000	3.563356000000	-0.228557000000
C	6.237430000000	4.246688000000	1.826217000000
H	7.327985000000	4.097525000000	1.818073000000
C	5.671284000000	0.241641000000	2.493487000000
C	4.820404000000	0.586257000000	1.441592000000
C	5.265602000000	0.426751000000	3.836311000000
H	5.141178000000	0.486818000000	0.398762000000
H	5.954951000000	0.149882000000	4.649072000000
C	3.543815000000	1.107549000000	1.744241000000
C	4.017602000000	0.970058000000	4.162489000000
H	3.705219000000	1.137655000000	5.202377000000
C	3.166875000000	1.317361000000	3.100082000000
H	6.669558000000	-0.165601000000	2.277849000000
C	1.379042000000	2.140924000000	1.924565000000
C	2.421766000000	1.556148000000	0.945817000000
O	1.951492000000	1.868422000000	3.261835000000
C	-0.041181000000	1.478438000000	1.804377000000
H	-0.469387000000	1.660344000000	2.813681000000
C	-0.075123000000	-0.016325000000	1.554243000000
C	-0.766747000000	-0.576103000000	0.461017000000
C	0.553073000000	-0.895625000000	2.464363000000
H	-1.278213000000	0.080138000000	-0.256253000000
H	1.075454000000	-0.481907000000	3.338973000000
C	-0.830688000000	-1.967827000000	0.282005000000
C	0.502994000000	-2.284201000000	2.280050000000
H	-1.384119000000	-2.378688000000	-0.575978000000
H	1.004313000000	-2.947112000000	3.001805000000
C	-0.195751000000	-2.828372000000	1.189881000000
H	-0.252130000000	-3.919394000000	1.053994000000
N	2.093525000000	1.573388000000	-0.314603000000
S	3.100791000000	1.132150000000	-1.604463000000
O	4.382149000000	1.892685000000	-1.567443000000
O	2.232298000000	1.248635000000	-2.794593000000
C	3.498435000000	-0.607713000000	-1.372109000000
C	2.542993000000	-1.499576000000	-0.863993000000
C	4.769297000000	-1.050499000000	-1.766666000000
H	1.549058000000	-1.143399000000	-0.558914000000
H	5.499147000000	-0.325534000000	-2.156171000000
C	2.883168000000	-2.850568000000	-0.734692000000
C	5.085795000000	-2.409613000000	-1.639582000000
H	2.137277000000	-3.546355000000	-0.320680000000
H	6.082072000000	-2.763916000000	-1.947844000000

C	4.154108000000	-3.330470000000	-1.118377000000
C	4.498810000000	-4.789119000000	-0.960283000000
H	3.758747000000	-5.435064000000	-1.476261000000
H	5.500475000000	-5.021567000000	-1.369870000000
H	4.491634000000	-5.086763000000	0.109411000000
P	1.340226000000	6.232329000000	0.784613000000
C	2.768686000000	7.379502000000	1.129109000000
C	3.920970000000	7.385284000000	0.315574000000
C	2.784118000000	8.115116000000	2.333714000000
H	3.938678000000	6.803450000000	-0.616438000000
H	1.897182000000	8.123380000000	2.985435000000
C	5.057667000000	8.113001000000	0.695771000000
C	3.921687000000	8.843695000000	2.711690000000
H	5.950042000000	8.099354000000	0.052041000000
H	3.915175000000	9.414921000000	3.652575000000
C	5.062106000000	8.843404000000	1.893935000000
H	5.956179000000	9.412178000000	2.191573000000
C	1.458919000000	6.035673000000	-1.032263000000
C	1.531343000000	7.174791000000	-1.858998000000
C	1.531806000000	4.750615000000	-1.608236000000
H	1.505168000000	8.184462000000	-1.424320000000
H	1.470425000000	3.844556000000	-0.988803000000
C	1.660106000000	7.024999000000	-3.248408000000
C	1.705001000000	4.607208000000	-2.991711000000
H	1.700067000000	7.919261000000	-3.888694000000
H	1.801082000000	3.590150000000	-3.402209000000
C	1.758622000000	5.743896000000	-3.814142000000
H	1.882367000000	5.631529000000	-4.902141000000
C	-0.028234000000	7.244576000000	1.470806000000
C	-0.889987000000	6.712402000000	2.450438000000
C	-0.192777000000	8.576200000000	1.044742000000
H	-0.779389000000	5.668461000000	2.775130000000
H	0.494650000000	9.012938000000	0.306153000000
C	-1.900862000000	7.513048000000	2.999255000000
C	-1.226410000000	9.362705000000	1.575974000000
H	-2.566204000000	7.092263000000	3.767966000000
H	-1.357696000000	10.397593000000	1.225703000000
C	-2.078755000000	8.834166000000	2.556777000000
H	-2.884340000000	9.453679000000	2.979297000000

TS4*P-SS*

Electronic energy = -3255.229951 Hartree

C	-0.692618000000	3.729359000000	1.508528000000
O	-1.370048000000	4.764558000000	1.438071000000
C	0.649850000000	3.620795000000	2.021767000000
C	-1.097316000000	2.353361000000	0.930948000000
H	0.608903000000	5.560119000000	2.810607000000
H	-0.734678000000	2.408103000000	-0.120456000000
C	-2.582775000000	2.094698000000	0.906764000000
C	-3.197015000000	1.055026000000	1.631425000000
C	-3.396187000000	2.948079000000	0.128228000000
H	-2.586867000000	0.364112000000	2.231831000000
H	-2.925371000000	3.767649000000	-0.434152000000
C	-4.588552000000	0.872306000000	1.584837000000
C	-4.784858000000	2.767126000000	0.085208000000
H	-5.048467000000	0.049810000000	2.154149000000
H	-5.403494000000	3.444455000000	-0.523833000000
C	-5.388064000000	1.728075000000	0.813257000000
H	-6.478757000000	1.583843000000	0.775446000000
C	1.311574000000	4.799701000000	2.424239000000
C	2.670117000000	4.869454000000	3.009174000000
C	2.845700000000	5.482769000000	4.271359000000
C	3.807135000000	4.357184000000	2.346409000000
H	1.971167000000	5.909397000000	4.786981000000
H	3.727213000000	3.955000000000	1.328534000000
C	4.109851000000	5.530877000000	4.874403000000
C	5.070403000000	4.405564000000	2.949370000000
H	4.225059000000	5.999301000000	5.863911000000
H	5.934904000000	3.996717000000	2.405740000000
C	5.225918000000	4.983042000000	4.218479000000
H	6.218706000000	5.021572000000	4.692564000000
C	5.363366000000	0.452357000000	3.179813000000
C	4.678074000000	0.856506000000	2.033145000000
C	4.740155000000	0.504247000000	4.449772000000
H	5.174149000000	0.854156000000	1.057479000000
H	5.306215000000	0.189996000000	5.340352000000
C	3.346159000000	1.311527000000	2.161292000000
C	3.425092000000	0.953541000000	4.605590000000

H	2.934723000000	1.010786000000	5.586815000000
C	2.741101000000	1.357142000000	3.446837000000
H	6.402176000000	0.101734000000	3.097888000000
C	1.105482000000	2.189094000000	2.058553000000
C	2.354642000000	1.824443000000	1.233982000000
O	1.484752000000	1.819891000000	3.448479000000
C	-0.203539000000	1.379787000000	1.706817000000
H	-0.662808000000	1.240526000000	2.709002000000
C	0.002773000000	0.001460000000	1.110992000000
C	-0.486936000000	-0.340842000000	-0.164820000000
C	0.658204000000	-0.997454000000	1.863931000000
H	-1.014537000000	0.412160000000	-0.766586000000
H	1.030697000000	-0.758460000000	2.871373000000
C	-0.338915000000	-1.643481000000	-0.669103000000
C	0.820474000000	-2.294224000000	1.357444000000
H	-0.743810000000	-1.889875000000	-1.662482000000
H	1.334629000000	-3.055387000000	1.963826000000
C	0.312910000000	-2.626253000000	0.090645000000
H	0.417586000000	-3.650919000000	-0.297935000000
N	2.300308000000	2.046075000000	-0.049693000000
S	3.653435000000	2.002936000000	-1.080377000000
O	4.825142000000	2.684539000000	-0.458245000000
O	3.143106000000	2.507812000000	-2.372721000000
C	4.073555000000	0.266502000000	-1.278114000000
C	3.073526000000	-0.715004000000	-1.297230000000
C	5.422335000000	-0.062962000000	-1.486387000000
H	2.020010000000	-0.447974000000	-1.130742000000
H	6.185532000000	0.729007000000	-1.463119000000
C	3.442407000000	-2.048423000000	-1.514014000000
C	5.767584000000	-1.400528000000	-1.712837000000
H	2.658649000000	-2.820991000000	-1.516675000000
H	6.823623000000	-1.664649000000	-1.881198000000
C	4.787454000000	-2.416025000000	-1.725843000000
C	5.171819000000	-3.859107000000	-1.927452000000
H	5.402204000000	-4.344816000000	-0.954481000000
H	4.351644000000	-4.438522000000	-2.394768000000
H	6.074453000000	-3.957640000000	-2.562033000000
P	1.483255000000	6.292467000000	0.513127000000
C	3.201942000000	6.495312000000	-0.043443000000
C	3.684537000000	5.871688000000	-1.214535000000
C	4.118648000000	7.127249000000	0.832288000000
H	3.000021000000	5.340492000000	-1.890781000000
H	3.766617000000	7.571530000000	1.775678000000
C	5.055372000000	5.890883000000	-1.505895000000
C	5.482316000000	7.164670000000	0.517368000000
H	5.416762000000	5.372721000000	-2.406170000000
H	6.183070000000	7.661646000000	1.205259000000
C	5.955829000000	6.542309000000	-0.649678000000
H	7.031414000000	6.551553000000	-0.882485000000
C	0.458251000000	5.766252000000	-0.895071000000
C	-0.568972000000	6.578264000000	-1.422775000000
C	0.646408000000	4.464244000000	-1.401859000000
H	-0.730242000000	7.591533000000	-1.027863000000
H	1.413077000000	3.796219000000	-0.988041000000
C	-1.392096000000	6.085306000000	-2.443021000000
C	-0.172460000000	3.984734000000	-2.434899000000
H	-2.192055000000	6.723230000000	-2.848829000000
H	0.010233000000	2.972355000000	-2.825992000000
C	-1.199556000000	4.787754000000	-2.951324000000
H	-1.849833000000	4.408452000000	-3.754364000000
C	0.834661000000	7.906563000000	1.050855000000
C	-0.413278000000	7.886056000000	1.719425000000
C	1.501030000000	9.133367000000	0.846718000000
H	-0.941878000000	6.924506000000	1.845728000000
H	2.461830000000	9.156497000000	0.311794000000
C	-0.977379000000	9.086211000000	2.176089000000
C	0.929724000000	10.325264000000	1.315493000000
H	-1.951054000000	9.065851000000	2.688915000000
H	1.451856000000	11.280064000000	1.149627000000
C	-0.306529000000	10.304755000000	1.982471000000
H	-0.750173000000	11.243468000000	2.347993000000

4a*SS*

Electronic energy = -2219.06562 Hartree

C	-0.448726000000	4.021433000000	1.717042000000
O	-1.041591000000	5.084166000000	1.655270000000
C	0.788402000000	3.756160000000	2.507849000000
C	-0.807380000000	2.722915000000	0.967780000000

H	0.807734000000	5.670557000000	3.308537000000
H	-0.219601000000	2.817061000000	0.022531000000
C	-2.274627000000	2.579124000000	0.643967000000
C	-3.077412000000	1.559814000000	1.192221000000
C	-2.872931000000	3.517244000000	-0.224229000000
H	-2.635949000000	0.803861000000	1.857985000000
H	-2.258046000000	4.322385000000	-0.651440000000
C	-4.444382000000	1.480365000000	0.882866000000
C	-4.238058000000	3.439437000000	-0.530430000000
H	-5.053452000000	0.672782000000	1.317027000000
H	-4.687347000000	4.181945000000	-1.207458000000
C	-5.030039000000	2.419856000000	0.021913000000
H	-6.101418000000	2.355970000000	-0.221868000000
C	1.374313000000	4.720017000000	3.259252000000
C	2.643575000000	4.619825000000	3.999135000000
C	2.675913000000	4.829016000000	5.396759000000
C	3.850573000000	4.336118000000	3.319686000000
H	1.743089000000	5.070323000000	5.928990000000
H	3.860326000000	4.229738000000	2.224755000000
C	3.876937000000	4.696720000000	6.105283000000
C	5.052800000000	4.224074000000	4.032425000000
H	3.885085000000	4.839708000000	7.196592000000
H	5.981677000000	4.004195000000	3.485539000000
C	5.067995000000	4.389279000000	5.425388000000
H	6.011570000000	4.291783000000	5.983576000000
C	5.425936000000	0.499727000000	3.267259000000
C	4.753097000000	1.063648000000	2.180632000000
C	4.774745000000	0.324457000000	4.509994000000
H	5.271453000000	1.241432000000	1.233178000000
H	5.331079000000	-0.112089000000	5.353658000000
C	3.403219000000	1.444807000000	2.342698000000
C	3.441760000000	0.707657000000	4.701035000000
H	2.930697000000	0.600351000000	5.667245000000
C	2.776892000000	1.269973000000	3.603880000000
H	6.478676000000	0.201782000000	3.158348000000
C	1.167064000000	2.291149000000	2.363295000000
C	2.413363000000	2.081702000000	1.484733000000
O	1.500630000000	1.710567000000	3.651597000000
C	-0.141587000000	1.626057000000	1.813951000000
H	-0.753307000000	1.485069000000	2.732693000000
C	0.051210000000	0.270679000000	1.162121000000
C	-0.275718000000	0.038825000000	-0.188537000000
C	0.554190000000	-0.806246000000	1.924533000000
H	-0.686313000000	0.852401000000	-0.802290000000
H	0.791486000000	-0.651068000000	2.987229000000
C	-0.104473000000	-1.231715000000	-0.762194000000
C	0.742112000000	-2.070345000000	1.348811000000
H	-0.375511000000	-1.390919000000	-1.816821000000
H	1.140793000000	-2.893446000000	1.961059000000
C	0.408934000000	-2.289933000000	0.002426000000
H	0.538794000000	-3.286951000000	-0.445654000000
N	2.342836000000	2.511356000000	0.262356000000
S	3.701109000000	2.613725000000	-0.773604000000
O	4.814466000000	3.295866000000	-0.056483000000
O	3.171530000000	3.179214000000	-2.023585000000
C	4.212784000000	0.915280000000	-1.087451000000
C	3.272556000000	-0.121527000000	-1.145789000000
C	5.574097000000	0.676170000000	-1.334920000000
H	2.208221000000	0.074366000000	-0.954916000000
H	6.292123000000	1.507955000000	-1.279532000000
C	3.712328000000	-1.419501000000	-1.435015000000
C	5.991210000000	-0.625616000000	-1.636778000000
H	2.973439000000	-2.235224000000	-1.465138000000
H	7.057225000000	-0.817854000000	-1.836757000000
C	5.072389000000	-1.696270000000	-1.685051000000
C	5.538181000000	-3.101726000000	-1.965411000000
H	5.836109000000	-3.613670000000	-1.024837000000
H	4.738940000000	-3.711927000000	-2.429529000000
H	6.420076000000	-3.114417000000	-2.635672000000

TS3*P-SS*

Electronic energy = -3255.178836 Hartree

C	0.154650000000	3.699869000000	-0.175376000000
O	-0.859509000000	4.179606000000	-0.670063000000
C	1.172717000000	3.222618000000	-1.213086000000
C	0.203553000000	3.599095000000	1.357235000000
H	0.589653000000	2.595648000000	-1.908866000000
H	-0.767934000000	4.056958000000	1.627977000000

C	1.289282000000	4.374836000000	2.079633000000
C	2.661434000000	4.204726000000	1.811925000000
C	0.917767000000	5.210088000000	3.153012000000
H	2.980888000000	3.574542000000	0.975833000000
H	-0.151686000000	5.363219000000	3.368084000000
C	3.635074000000	4.817046000000	2.614511000000
C	1.887298000000	5.830375000000	3.954297000000
H	4.699132000000	4.656138000000	2.384096000000
H	1.573047000000	6.472537000000	4.791405000000
C	3.252291000000	5.625976000000	3.694699000000
H	4.015009000000	6.100229000000	4.331052000000
C	2.009295000000	4.083099000000	-1.993293000000
C	2.161119000000	3.763728000000	-3.449344000000
C	1.005398000000	3.652387000000	-4.255165000000
C	3.419035000000	3.596483000000	-4.064777000000
H	0.018108000000	3.786117000000	-3.784583000000
H	4.330398000000	3.631955000000	-3.450745000000
C	1.103551000000	3.387121000000	-5.626107000000
C	3.520826000000	3.349269000000	-5.441007000000
H	0.188584000000	3.301575000000	-6.232510000000
H	4.514020000000	3.219083000000	-5.897652000000
C	2.364746000000	3.243488000000	-6.228410000000
H	2.444586000000	3.041480000000	-7.307484000000
C	5.837123000000	0.019025000000	1.791751000000
C	4.867645000000	0.441252000000	0.873196000000
C	5.562060000000	-0.051108000000	3.177956000000
H	5.077307000000	0.490672000000	-0.204380000000
H	6.346277000000	-0.391359000000	3.870955000000
C	3.594272000000	0.806418000000	1.361164000000
C	4.304095000000	0.305286000000	3.685429000000
H	4.068609000000	0.260939000000	4.758077000000
C	3.349856000000	0.731895000000	2.757509000000
H	6.834505000000	-0.271096000000	1.427174000000
C	1.470902000000	1.448631000000	1.844619000000
C	2.344443000000	1.286894000000	0.778844000000
O	2.080892000000	1.142601000000	3.049304000000
C	0.142177000000	2.125354000000	1.930853000000
H	-0.025095000000	2.273650000000	3.016657000000
C	-1.049811000000	1.333579000000	1.406851000000

C	-1.119533000000	0.861770000000	0.078896000000
C	-2.135349000000	1.075881000000	2.267018000000
H	-0.281616000000	1.042582000000	-0.608443000000
H	-2.095464000000	1.439094000000	3.306680000000
C	-2.239183000000	0.144748000000	-0.367857000000
C	-3.257561000000	0.360803000000	1.821613000000
H	-2.266617000000	-0.216782000000	-1.407097000000
H	-4.092561000000	0.168312000000	2.512680000000
C	-3.312609000000	-0.108662000000	0.500516000000
H	-4.191195000000	-0.669886000000	0.147609000000
N	2.014057000000	1.624883000000	-0.526404000000
S	2.556642000000	0.695179000000	-1.799066000000
O	3.971752000000	1.008380000000	-2.168474000000
O	1.524766000000	0.798051000000	-2.862809000000
C	2.563767000000	-1.001774000000	-1.175825000000
C	1.460682000000	-1.524094000000	-0.482338000000
C	3.676743000000	-1.802560000000	-1.462943000000
H	0.589524000000	-0.894235000000	-0.258733000000
H	4.523990000000	-1.366466000000	-2.011118000000
C	1.489916000000	-2.858251000000	-0.063914000000
C	3.684010000000	-3.140712000000	-1.045787000000
H	0.628395000000	-3.264564000000	0.489371000000
H	4.556486000000	-3.773150000000	-1.273807000000
C	2.598877000000	-3.689805000000	-0.334658000000
C	2.619064000000	-5.118496000000	0.144633000000
H	1.676278000000	-5.646503000000	-0.115886000000
H	3.466422000000	-5.685654000000	-0.292250000000
H	2.717797000000	-5.163893000000	1.252776000000
P	2.890483000000	5.514392000000	-1.479153000000
C	1.911262000000	6.606486000000	-0.372193000000
C	2.463987000000	7.305862000000	0.718239000000
C	0.550575000000	6.788405000000	-0.697133000000
H	3.506763000000	7.142628000000	1.015643000000
H	0.099493000000	6.204746000000	-1.511713000000
C	1.664430000000	8.177299000000	1.469367000000
C	-0.244066000000	7.659272000000	0.060506000000
H	2.101787000000	8.702560000000	2.331125000000
H	-1.308612000000	7.775131000000	-0.191383000000
C	0.311933000000	8.359284000000	1.141612000000

H	-0.314087000000	9.037997000000	1.740617000000
C	4.540348000000	5.219518000000	-0.734848000000
C	4.969271000000	3.883028000000	-0.609091000000
C	5.390635000000	6.274547000000	-0.334611000000
H	4.329337000000	3.056939000000	-0.961698000000
H	5.086124000000	7.321569000000	-0.479055000000
C	6.222081000000	3.606570000000	-0.038995000000
C	6.642336000000	5.988419000000	0.228034000000
H	6.539316000000	2.560854000000	0.079135000000
H	7.301439000000	6.812149000000	0.541540000000
C	7.053144000000	4.653881000000	0.386986000000
H	8.032411000000	4.430717000000	0.837157000000
C	3.216684000000	6.525829000000	-2.980293000000
C	4.522976000000	6.769234000000	-3.444958000000
C	2.114415000000	7.010001000000	-3.714483000000
H	5.389161000000	6.368670000000	-2.899912000000
H	1.089798000000	6.799120000000	-3.377919000000
C	4.723344000000	7.500979000000	-4.625369000000
C	2.320436000000	7.735255000000	-4.894003000000
H	5.748114000000	7.681054000000	-4.984258000000
H	1.453502000000	8.100047000000	-5.465480000000
C	3.625135000000	7.984877000000	-5.351076000000
H	3.784194000000	8.551501000000	-6.280996000000

IV'$_{P\text{-}SS}$

Electronic energy = -3255.193061 Hartree

C	0.292814000000	3.513909000000	-0.056005000000
O	-0.727254000000	4.133108000000	-0.332147000000
C	1.213032000000	3.098114000000	-1.229907000000
C	0.502362000000	3.188099000000	1.454579000000
H	0.447749000000	2.956812000000	-2.021711000000
H	-0.541914000000	3.219061000000	1.816971000000
C	1.179913000000	4.353542000000	2.172605000000
C	2.553606000000	4.376913000000	2.478960000000
C	0.372810000000	5.419914000000	2.624211000000
H	3.208290000000	3.561092000000	2.153026000000
H	-0.697868000000	5.425638000000	2.369509000000
C	3.105314000000	5.424363000000	3.233395000000
C	0.917487000000	6.455268000000	3.392769000000
H	4.184910000000	5.420363000000	3.447600000000
H	0.266556000000	7.268425000000	3.747047000000
C	2.287515000000	6.460505000000	3.704415000000
H	2.715585000000	7.274991000000	4.308637000000
C	2.254384000000	4.059462000000	-1.783887000000
C	2.819502000000	3.614709000000	-3.094007000000
C	1.973607000000	3.339943000000	-4.196448000000
C	4.211528000000	3.451672000000	-3.291774000000
H	0.885684000000	3.459167000000	-4.086300000000
H	4.892252000000	3.626705000000	-2.445210000000
C	2.492247000000	2.914915000000	-5.424737000000
C	4.736487000000	3.051417000000	-4.529053000000
H	1.804790000000	2.700301000000	-6.257522000000
H	5.825835000000	2.939769000000	-4.647692000000
C	3.878372000000	2.771430000000	-5.601948000000
H	4.284584000000	2.438385000000	-6.569090000000
C	6.314880000000	0.374004000000	-0.786527000000
C	5.018124000000	0.744024000000	-1.165795000000
C	6.677690000000	0.215316000000	0.571989000000
H	4.727408000000	0.857770000000	-2.218559000000
H	7.705639000000	-0.081799000000	0.828479000000
C	4.066315000000	0.964846000000	-0.146594000000
C	5.747524000000	0.429462000000	1.601520000000
H	6.006300000000	0.315096000000	2.663569000000
C	4.460801000000	0.804185000000	1.208568000000
H	7.072068000000	0.201665000000	-1.566133000000
C	2.342664000000	1.424152000000	1.260911000000
C	2.669081000000	1.367841000000	-0.076503000000
O	3.418015000000	1.099399000000	2.057640000000
C	1.049195000000	1.806921000000	1.900673000000
H	1.276093000000	1.922072000000	2.981838000000
C	0.013214000000	0.676213000000	1.820030000000
C	-1.262925000000	0.834096000000	1.247031000000
C	0.325407000000	-0.552422000000	2.438793000000
H	-1.536547000000	1.765505000000	0.731977000000
H	1.327301000000	-0.695907000000	2.873294000000
C	-2.219631000000	-0.191022000000	1.340930000000
C	-0.628101000000	-1.572846000000	2.533724000000
H	-3.215142000000	-0.041967000000	0.895642000000

H	-0.368479000000	-2.515026000000	3.040370000000
C	-1.913566000000	-1.389643000000	1.997940000000
H	-2.668923000000	-2.185607000000	2.083200000000
N	1.722222000000	1.697996000000	-1.054103000000
S	1.432243000000	0.557402000000	-2.314605000000
O	2.672777000000	0.294707000000	-3.074841000000
O	0.203966000000	1.022700000000	-2.999102000000
C	1.064410000000	-0.959788000000	-1.433989000000
C	-0.275725000000	-1.341793000000	-1.282944000000
C	2.113965000000	-1.790376000000	-1.013010000000
H	-1.071030000000	-0.675982000000	-1.645089000000
H	3.157433000000	-1.491435000000	-1.183659000000
C	-0.562154000000	-2.578001000000	-0.695586000000
C	1.802878000000	-3.011093000000	-0.400491000000
H	-1.611373000000	-2.891542000000	-0.586858000000
H	2.619817000000	-3.666388000000	-0.059750000000
C	0.466198000000	-3.427416000000	-0.235767000000
C	0.132146000000	-4.735095000000	0.433096000000
H	-0.636751000000	-5.298022000000	-0.133821000000
H	1.023924000000	-5.381496000000	0.543670000000
H	-0.283666000000	-4.559602000000	1.448849000000
P	2.905953000000	5.543956000000	-1.207013000000
C	1.673293000000	6.674336000000	-0.431575000000
C	2.008842000000	7.617724000000	0.557717000000
C	0.375333000000	6.688056000000	-0.984905000000
H	2.993671000000	7.591170000000	1.039366000000
H	0.098943000000	5.928624000000	-1.729576000000
C	1.071638000000	8.578937000000	0.963441000000
C	-0.565076000000	7.635124000000	-0.558888000000
H	1.347725000000	9.309781000000	1.738468000000
H	-1.579192000000	7.620172000000	-0.985549000000
C	-0.215027000000	8.592883000000	0.405858000000
H	-0.950624000000	9.344350000000	0.731438000000
C	4.413004000000	5.558391000000	-0.109181000000
C	4.912080000000	4.307306000000	0.287975000000
C	5.118069000000	6.726365000000	0.255054000000
H	4.380331000000	3.401572000000	-0.039373000000
H	4.787962000000	7.712009000000	-0.105415000000
C	6.068213000000	4.210987000000	1.077670000000
C	6.269254000000	6.633981000000	1.051837000000
H	6.439067000000	3.217868000000	1.373841000000
H	6.811264000000	7.549324000000	1.335470000000
C	6.740596000000	5.377899000000	1.472469000000
H	7.647703000000	5.310813000000	2.092564000000
C	3.497507000000	6.489888000000	-2.679813000000
C	4.865592000000	6.648858000000	-2.970643000000
C	2.536720000000	6.996479000000	-3.576268000000
H	5.625009000000	6.237196000000	-2.291563000000
H	1.466415000000	6.860456000000	-3.366255000000
C	5.265372000000	7.313576000000	-4.139998000000
C	2.939114000000	7.661240000000	-4.741947000000
H	6.337504000000	7.423171000000	-4.363031000000
H	2.179369000000	8.051539000000	-5.435761000000
C	4.304908000000	7.822022000000	-5.026344000000
H	4.620200000000	8.339708000000	-5.945034000000

TS5*P-SS*

Electronic energy = -3255.198768 Hartree

C	0.715232000000	3.531330000000	0.214827000000
O	-0.104809000000	4.424170000000	-0.114598000000
C	1.832973000000	3.200750000000	-0.651726000000
C	0.531828000000	3.021111000000	1.681554000000
H	1.000658000000	4.521677000000	-2.083272000000
H	-0.560681000000	3.133822000000	1.824932000000
C	1.176598000000	4.044937000000	2.621587000000
C	2.300856000000	3.772667000000	3.430501000000
C	0.598193000000	5.331175000000	2.705264000000
H	2.777207000000	2.784406000000	3.416303000000
H	-0.272334000000	5.553975000000	2.072046000000
C	2.836350000000	4.756642000000	4.278517000000
C	1.125288000000	6.308074000000	3.558882000000
H	3.715725000000	4.516234000000	4.895949000000
H	0.648561000000	7.297919000000	3.606686000000
C	2.253819000000	6.029517000000	4.346536000000
H	2.670382000000	6.797934000000	5.015529000000
C	1.980628000000	4.100292000000	-1.799315000000
C	2.826949000000	3.777644000000	-2.993058000000
C	2.285571000000	3.981732000000	-4.280848000000

C	4.163946000000	3.335727000000	-2.887732000000
H	1.244757000000	4.327425000000	-4.373163000000
H	4.597142000000	3.160418000000	-1.897183000000
C	3.047274000000	3.740693000000	-5.430283000000
C	4.928592000000	3.098643000000	-4.039216000000
H	2.601992000000	3.898829000000	-6.424151000000
H	5.963227000000	2.739198000000	-3.934970000000
C	4.376100000000	3.299512000000	-5.313075000000
H	4.976635000000	3.104840000000	-6.214795000000
C	6.696868000000	0.264560000000	0.949289000000
C	5.628944000000	0.893840000000	0.296850000000
C	6.545102000000	-0.305591000000	2.236199000000
H	5.727373000000	1.306653000000	-0.715712000000
H	7.405893000000	-0.794351000000	2.716690000000
C	4.388790000000	0.951927000000	0.962456000000
C	5.313921000000	-0.267798000000	2.910440000000
H	5.175225000000	-0.711310000000	3.906423000000
C	4.262113000000	0.365467000000	2.244784000000
H	7.675439000000	0.204282000000	0.449483000000
C	2.270291000000	1.197673000000	1.737653000000
C	3.077753000000	1.497381000000	0.657472000000
O	2.980412000000	0.524384000000	2.715736000000
C	0.859840000000	1.565189000000	2.060778000000
H	0.807801000000	1.525799000000	3.170362000000
C	-0.192155000000	0.554933000000	1.588224000000
C	-1.153323000000	0.863253000000	0.606226000000
C	-0.268433000000	-0.693101000000	2.240086000000
H	-1.110759000000	1.821429000000	0.068727000000
H	0.485577000000	-0.947235000000	3.001641000000
C	-2.189336000000	-0.039676000000	0.314689000000
C	-1.301844000000	-1.592746000000	1.949884000000
H	-2.937842000000	0.225209000000	-0.447707000000
H	-1.356974000000	-2.552320000000	2.487034000000
C	-2.275909000000	-1.263443000000	0.992657000000
H	-3.097505000000	-1.962743000000	0.774370000000
N	2.717674000000	2.119833000000	-0.543505000000
S	2.499935000000	0.755710000000	-1.858888000000
O	3.881787000000	0.476933000000	-2.326377000000
O	1.433579000000	1.249822000000	-2.765044000000

C	1.924986000000	-0.784378000000	-1.126731000000
C	0.596143000000	-1.174902000000	-1.346046000000
C	2.829371000000	-1.619324000000	-0.451118000000
H	-0.077275000000	-0.508303000000	-1.901114000000
H	3.879541000000	-1.319262000000	-0.335418000000
C	0.174547000000	-2.424706000000	-0.881588000000
C	2.374845000000	-2.849817000000	0.038338000000
H	-0.859862000000	-2.747182000000	-1.072243000000
H	3.076151000000	-3.505994000000	0.577517000000
C	1.046352000000	-3.275031000000	-0.169404000000
C	0.553454000000	-4.590831000000	0.372463000000
H	-0.073808000000	-5.127388000000	-0.367731000000
H	1.388139000000	-5.255273000000	0.668111000000
H	-0.080350000000	-4.428771000000	1.271316000000
P	2.915941000000	5.968160000000	-1.262853000000
C	2.052596000000	7.077201000000	-0.088701000000
C	2.817190000000	7.908279000000	0.757112000000
C	0.656503000000	7.247029000000	-0.166661000000
H	3.906736000000	7.772521000000	0.821593000000
H	0.057238000000	6.559740000000	-0.778926000000
C	2.187909000000	8.910487000000	1.507982000000
C	0.036067000000	8.256281000000	0.584729000000
H	2.788371000000	9.552911000000	2.169440000000
H	-1.055464000000	8.381721000000	0.525361000000
C	0.798836000000	9.093384000000	1.414316000000
H	0.307270000000	9.885009000000	2.000409000000
C	4.456807000000	5.483398000000	-0.441351000000
C	4.316218000000	4.780216000000	0.777078000000
C	5.738615000000	5.669779000000	-0.999326000000
H	3.326933000000	4.654116000000	1.237424000000
H	5.858665000000	6.233933000000	-1.934670000000
C	5.444908000000	4.238787000000	1.405285000000
C	6.862198000000	5.130370000000	-0.357209000000
H	5.319469000000	3.677127000000	2.342562000000
H	7.860196000000	5.277494000000	-0.797014000000
C	6.717021000000	4.401762000000	0.835629000000
H	7.598632000000	3.961567000000	1.325205000000
C	3.286033000000	7.071899000000	-2.678577000000
C	4.098994000000	6.636372000000	-3.753674000000

C	2.676054000000	8.346785000000	-2.760979000000
H	4.565720000000	5.643104000000	-3.736860000000
H	2.036038000000	8.706485000000	-1.943589000000
C	4.308670000000	7.465846000000	-4.863275000000
C	2.886204000000	9.164365000000	-3.879642000000
H	4.945067000000	7.105440000000	-5.685508000000
H	2.405421000000	10.153502000000	-3.922190000000
C	3.704310000000	8.730357000000	-4.934353000000
H	3.866596000000	9.374855000000	-5.811507000000

4a'*SS*

Electronic energy = -2219.044984 Hartree

C	-0.050188000000	2.862439000000	0.003473000000
O	-1.142271000000	3.157034000000	-0.480956000000
C	1.184357000000	2.858878000000	-0.861904000000
C	0.049889000000	2.738209000000	1.540637000000
H	0.183528000000	4.223820000000	-2.058174000000
H	-1.010789000000	2.752400000000	1.859435000000
C	0.672730000000	4.048390000000	2.038566000000
C	1.835213000000	4.117983000000	2.831390000000
C	0.031494000000	5.255958000000	1.683156000000
H	2.362939000000	3.206894000000	3.141348000000
H	-0.878364000000	5.215913000000	1.064226000000
C	2.340053000000	5.358947000000	3.253724000000
C	0.535695000000	6.492736000000	2.103298000000
H	3.249182000000	5.387669000000	3.873419000000
H	0.016786000000	7.419225000000	1.813907000000
C	1.697826000000	6.550142000000	2.889675000000
H	2.099363000000	7.520666000000	3.218149000000
C	1.160685000000	3.733882000000	-1.919981000000
C	2.191989000000	4.140230000000	-2.866150000000
C	1.784200000000	4.915883000000	-3.981537000000
C	3.576307000000	3.871143000000	-2.713130000000
H	0.714255000000	5.144869000000	-4.104966000000
H	3.920251000000	3.286234000000	-1.852389000000
C	2.710802000000	5.379776000000	-4.921669000000
C	4.501621000000	4.352687000000	-3.642942000000
H	2.367028000000	5.968101000000	-5.785903000000
H	5.571526000000	4.136283000000	-3.502064000000
C	4.076297000000	5.098697000000	-4.756390000000
H	4.809781000000	5.461860000000	-5.492310000000
C	6.589822000000	1.018557000000	0.788343000000
C	5.406915000000	1.351128000000	0.116455000000
C	6.584729000000	0.605609000000	2.141174000000
H	5.411599000000	1.634985000000	-0.943260000000
H	7.534253000000	0.348288000000	2.633805000000
C	4.189843000000	1.269660000000	0.826779000000
C	5.388195000000	0.520717000000	2.867971000000
H	5.358893000000	0.208976000000	3.921360000000
C	4.220916000000	0.863521000000	2.182770000000
H	7.547140000000	1.075691000000	0.249095000000
C	2.092418000000	1.266505000000	1.708283000000
C	2.783535000000	1.523176000000	0.537412000000
O	2.955499000000	0.881653000000	2.714630000000
C	0.667266000000	1.455917000000	2.121614000000
H	0.714993000000	1.621639000000	3.220476000000
C	-0.191365000000	0.194557000000	1.948174000000
C	-1.416488000000	0.186966000000	1.253594000000
C	0.225945000000	-0.988636000000	2.594524000000
H	-1.772191000000	1.084742000000	0.729082000000
H	1.184854000000	-0.996130000000	3.135120000000
C	-2.214433000000	-0.970044000000	1.230758000000
C	-0.572298000000	-2.138554000000	2.574520000000
H	-3.172408000000	-0.953075000000	0.688842000000
H	-0.234252000000	-3.045804000000	3.098044000000
C	-1.802790000000	-2.131846000000	1.897108000000
H	-2.437077000000	-3.031551000000	1.889048000000
N	2.257482000000	1.951693000000	-0.694038000000
S	2.404995000000	0.719630000000	-2.022488000000
O	3.846421000000	0.625905000000	-2.329228000000
O	1.410774000000	1.091774000000	-3.044924000000
C	1.907019000000	-0.814090000000	-1.255756000000
C	0.568505000000	-1.219245000000	-1.362297000000
C	2.867740000000	-1.603333000000	-0.603870000000
H	-0.157438000000	-0.579286000000	-1.883511000000
H	3.912162000000	-1.265425000000	-0.550959000000
C	0.196590000000	-2.452361000000	-0.816443000000
C	2.466618000000	-2.823167000000	-0.048254000000

H	-0.848703000000	-2.783648000000	-0.899930000000
H	3.210265000000	-3.447904000000	0.470921000000
C	1.132920000000	-3.272536000000	-0.153194000000
C	0.726846000000	-4.611327000000	0.403872000000
H	0.958108000000	-5.424014000000	-0.318075000000
H	1.271599000000	-4.845629000000	1.339986000000
H	-0.359528000000	-4.652827000000	0.612261000000

RC*P-SR*

Electronic energy = -3255.19649 Hartree

C	2.674080000000	2.439422000000	0.954928000000
O	3.116418000000	3.404241000000	0.335319000000
C	3.247206000000	2.007178000000	2.211317000000
C	1.502088000000	1.609154000000	0.409014000000
H	-2.273429000000	2.494685000000	1.227867000000
H	0.953653000000	2.282919000000	-0.275722000000
C	1.999440000000	0.432149000000	-0.418636000000
C	1.511809000000	0.256506000000	-1.728163000000
C	2.878241000000	-0.530669000000	0.115889000000
H	0.822339000000	1.007673000000	-2.142823000000
H	3.275511000000	-0.406053000000	1.133075000000
C	1.881881000000	-0.870678000000	-2.478167000000
C	3.255069000000	-1.651012000000	-0.635282000000
H	1.485129000000	-1.001522000000	-3.496933000000
H	3.953109000000	-2.382538000000	-0.200448000000
C	2.750343000000	-1.829053000000	-1.933360000000
H	3.041812000000	-2.711368000000	-2.523620000000
C	3.698295000000	1.618221000000	3.290890000000
C	4.211799000000	1.125306000000	4.522330000000
C	3.420670000000	0.264356000000	5.326585000000
C	5.527339000000	1.451980000000	4.940056000000
H	2.400156000000	0.016499000000	4.999314000000
H	6.142720000000	2.098854000000	4.300341000000
C	3.937727000000	-0.258040000000	6.516390000000
C	6.034378000000	0.918499000000	6.129485000000
H	3.319211000000	-0.925825000000	7.134634000000
H	7.059227000000	1.167695000000	6.443516000000
C	5.245277000000	0.064512000000	6.918454000000
H	5.651800000000	-0.354146000000	7.851530000000
C	-3.785045000000	-2.332905000000	-1.001562000000
C	-3.613760000000	-0.985857000000	-0.659411000000
C	-2.783246000000	-3.296954000000	-0.735510000000
H	-4.391856000000	-0.239147000000	-0.873280000000
H	-2.950821000000	-4.345129000000	-1.025240000000
C	-2.405309000000	-0.607678000000	-0.039609000000
C	-1.578477000000	-2.943022000000	-0.108967000000
H	-0.786795000000	-3.674367000000	0.104960000000
C	-1.426791000000	-1.595960000000	0.226299000000
H	-4.718164000000	-2.651073000000	-1.490647000000
C	-0.582144000000	0.315942000000	0.957972000000
C	-1.831087000000	0.632064000000	0.446291000000
O	-0.343873000000	-1.034542000000	0.852348000000
C	0.532506000000	1.136058000000	1.536189000000
H	1.105267000000	0.447578000000	2.193035000000
C	0.000908000000	2.268983000000	2.408127000000
C	-0.392248000000	1.984363000000	3.732058000000
C	-0.150617000000	3.585839000000	1.927687000000
H	-0.279077000000	0.957767000000	4.116125000000
H	0.137877000000	3.839803000000	0.897896000000
C	-0.925225000000	2.984526000000	4.555971000000
C	-0.692656000000	4.587641000000	2.750152000000
H	-1.221871000000	2.743150000000	5.588058000000
H	-0.816808000000	5.604224000000	2.348708000000
C	-1.079961000000	4.291929000000	4.064942000000
H	-1.501576000000	5.078663000000	4.708505000000
N	-2.469550000000	1.890656000000	0.415267000000
S	-2.221158000000	2.901246000000	-0.970067000000
O	-0.861711000000	2.716161000000	-1.540294000000
O	-2.688162000000	4.232365000000	-0.533781000000
C	-3.382374000000	2.183479000000	-2.126442000000
C	-2.922578000000	1.300009000000	-3.110536000000
C	-4.747209000000	2.475333000000	-1.978880000000
H	-1.847546000000	1.088931000000	-3.194167000000
H	-5.072645000000	3.179127000000	-1.199325000000
C	-3.857540000000	0.685814000000	-3.954640000000
C	-5.663886000000	1.852033000000	-2.831645000000
H	-3.507782000000	-0.020907000000	-4.722925000000
H	-6.738045000000	2.069786000000	-2.722985000000

C	-5.237297000000	0.945346000000	-3.829226000000
C	-6.237224000000	0.294810000000	-4.749377000000
H	-7.156619000000	-0.001720000000	-4.206156000000
H	-6.549351000000	0.997397000000	-5.551688000000
H	-5.817510000000	-0.603766000000	-5.241102000000
P	7.156861000000	0.500669000000	1.797476000000
C	8.983917000000	0.565867000000	1.529347000000
C	9.812166000000	0.450250000000	2.669566000000
C	9.584041000000	0.805113000000	0.276668000000
H	9.354597000000	0.279165000000	3.657975000000
H	8.953104000000	0.904707000000	-0.619188000000
C	11.205603000000	0.541792000000	2.554255000000
C	10.980228000000	0.912424000000	0.165983000000
H	11.836721000000	0.438665000000	3.450502000000
H	11.433927000000	1.100728000000	-0.819398000000
C	11.794927000000	0.776133000000	1.299900000000
H	12.888856000000	0.857094000000	1.209390000000
C	6.897366000000	-1.293409000000	2.195928000000
C	7.884176000000	-2.293618000000	2.081819000000
C	5.605663000000	-1.660234000000	2.633830000000
H	8.897866000000	-2.024646000000	1.748432000000
H	4.833823000000	-0.884171000000	2.747186000000
C	7.579545000000	-3.630557000000	2.386797000000
C	5.299197000000	-2.996253000000	2.926390000000
H	8.359857000000	-4.401500000000	2.290553000000
H	4.284748000000	-3.261983000000	3.261670000000
C	6.287837000000	-3.987242000000	2.804415000000
H	6.052467000000	-5.036305000000	3.040318000000
C	6.477372000000	0.554038000000	0.078823000000
C	5.743958000000	1.696784000000	-0.296063000000
C	6.621140000000	-0.505235000000	-0.843444000000
H	5.601315000000	2.520002000000	0.420353000000
H	7.174962000000	-1.411440000000	-0.552880000000
C	5.155346000000	1.780014000000	-1.568694000000
C	6.044754000000	-0.413858000000	-2.117440000000
H	4.549314000000	2.661902000000	-1.823025000000
H	6.152640000000	-1.248397000000	-2.827348000000
C	5.306272000000	0.725725000000	-2.479887000000
H	4.827636000000	0.779581000000	-3.469255000000

TS1$_{P\text{-}SR}$

Electronic energy = -3255.174421 Hartree

C	2.794943000000	1.270567000000	-0.140328000000
O	3.513718000000	1.510162000000	-1.120130000000
C	3.264923000000	1.085445000000	1.184720000000
C	1.276747000000	0.991696000000	-0.354293000000
H	-2.466973000000	2.472791000000	1.091375000000
H	0.908649000000	1.788004000000	-1.030060000000
C	1.126317000000	-0.335638000000	-1.088031000000
C	0.185563000000	-0.460326000000	-2.128522000000
C	1.861973000000	-1.474025000000	-0.702678000000
H	-0.391043000000	0.425613000000	-2.432490000000
H	2.610061000000	-1.389230000000	0.101435000000
C	-0.028422000000	-1.698337000000	-2.754860000000
C	1.651385000000	-2.709953000000	-1.329794000000
H	-0.766995000000	-1.777139000000	-3.568505000000
H	2.237199000000	-3.587799000000	-1.015862000000
C	0.698833000000	-2.829721000000	-2.354589000000
H	0.531031000000	-3.800652000000	-2.845505000000
C	3.653893000000	1.151831000000	2.396105000000
C	3.708761000000	0.301797000000	3.586522000000
C	2.939229000000	-0.891194000000	3.581388000000
C	4.467570000000	0.595685000000	4.741573000000
H	2.342655000000	-1.134646000000	2.689548000000
H	5.089906000000	1.500547000000	4.768459000000
C	2.931572000000	-1.746349000000	4.689227000000
C	4.460696000000	-0.265356000000	5.847928000000
H	2.319474000000	-2.660695000000	4.661772000000
H	5.068629000000	-0.016320000000	6.731098000000
C	3.691055000000	-1.438677000000	5.830427000000
H	3.682204000000	-2.110564000000	6.701904000000
C	-4.414408000000	-2.644032000000	0.816515000000
C	-4.164314000000	-1.271078000000	0.699083000000
C	-3.366927000000	-3.569608000000	1.040080000000
H	-4.977834000000	-0.554610000000	0.516073000000
H	-3.599986000000	-4.642151000000	1.118379000000
C	-2.830916000000	-0.828468000000	0.806478000000
C	-2.034778000000	-3.146314000000	1.165798000000

H	-1.206192000000	-3.846892000000	1.338726000000
C	-1.807960000000	-1.773050000000	1.050557000000
H	-5.447532000000	-3.013844000000	0.730606000000
C	-0.804828000000	0.207060000000	0.932962000000
C	-2.157019000000	0.454984000000	0.731799000000
O	-0.600595000000	-1.137424000000	1.160011000000
C	0.452875000000	1.025410000000	0.984061000000
H	1.084710000000	0.500582000000	1.731602000000
C	0.201300000000	2.422564000000	1.532780000000
C	0.442659000000	2.688086000000	2.896517000000
C	-0.310618000000	3.461385000000	0.727075000000
H	0.862766000000	1.892602000000	3.531871000000
H	-0.489405000000	3.283699000000	-0.343173000000
C	0.167776000000	3.950556000000	3.445761000000
C	-0.612374000000	4.716689000000	1.279304000000
H	0.386280000000	4.144258000000	4.506486000000
H	-1.040234000000	5.499312000000	0.634697000000
C	-0.370442000000	4.967281000000	2.640327000000
H	-0.590369000000	5.955287000000	3.071953000000
N	-2.832526000000	1.681103000000	0.541517000000
S	-3.019866000000	2.289225000000	-1.069382000000
O	-1.791026000000	2.087425000000	-1.881091000000
O	-3.578456000000	3.644057000000	-0.883154000000
C	-4.263298000000	1.174603000000	-1.706365000000
C	-3.867653000000	0.001381000000	-2.365675000000
C	-5.612908000000	1.465894000000	-1.467384000000
H	-2.800893000000	-0.203817000000	-2.531113000000
H	-5.884452000000	2.400858000000	-0.956328000000
C	-4.852895000000	-0.904136000000	-2.772920000000
C	-6.583472000000	0.547643000000	-1.888546000000
H	-4.551203000000	-1.836431000000	-3.275106000000
H	-7.648040000000	0.764083000000	-1.708258000000
C	-6.221690000000	-0.653097000000	-2.536107000000
C	-7.262894000000	-1.657141000000	-2.957584000000
H	-8.289112000000	-1.296496000000	-2.754161000000
H	-7.186433000000	-1.885714000000	-4.040445000000
H	-7.124950000000	-2.617411000000	-2.417558000000
P	4.306650000000	3.299947000000	2.903928000000
C	3.619840000000	4.520625000000	4.092105000000
C	3.246032000000	4.048223000000	5.370729000000
C	3.386491000000	5.873052000000	3.761848000000
H	3.401005000000	2.989388000000	5.630133000000
H	3.672836000000	6.251653000000	2.769999000000
C	2.659693000000	4.915420000000	6.303046000000
C	2.783908000000	6.731833000000	4.693504000000
H	2.374714000000	4.536116000000	7.296186000000
H	2.602385000000	7.783555000000	4.423966000000
C	2.419683000000	6.257765000000	5.964013000000
H	1.946649000000	6.934881000000	6.691300000000
C	6.104567000000	3.241784000000	3.253279000000
C	6.734843000000	4.161111000000	4.117518000000
C	6.863820000000	2.200923000000	2.670054000000
H	6.147958000000	4.970366000000	4.577073000000
H	6.369323000000	1.466084000000	2.015264000000
C	8.106805000000	4.044541000000	4.386872000000
C	8.236387000000	2.099168000000	2.935228000000
H	8.591529000000	4.766402000000	5.061828000000
H	8.820519000000	1.288784000000	2.473524000000
C	8.860340000000	3.018260000000	3.795096000000
H	9.936558000000	2.931143000000	4.007972000000
C	4.147292000000	4.031922000000	1.246540000000
C	2.873055000000	4.480971000000	0.836955000000
C	5.198297000000	3.981343000000	0.308836000000
H	2.044864000000	4.519857000000	1.556237000000
H	6.193874000000	3.625812000000	0.609763000000
C	2.660104000000	4.878344000000	-0.489193000000
C	4.974168000000	4.371340000000	-1.017589000000
H	1.659762000000	5.219715000000	-0.794009000000
H	5.796318000000	4.312092000000	-1.745989000000
C	3.705851000000	4.813432000000	-1.421749000000
H	3.529888000000	5.100720000000	-2.469094000000

$I_{P\text{-}SR}$

Electronic energy = -3255.190938 Hartree

C	2.827677000000	-0.249155000000	0.977518000000
O	3.790287000000	-0.646836000000	0.253715000000
C	3.086453000000	0.250239000000	2.252875000000
C	1.374301000000	-0.422631000000	0.474739000000

H	-1.959979000000	1.398818000000	1.900121000000	H	0.771715000000	-1.656400000000	2.140674000000
H	1.063390000000	0.546434000000	0.030225000000	C	0.312538000000	0.348499000000	2.716013000000
C	1.274624000000	-1.468683000000	-0.618074000000	C	0.250745000000	-0.002860000000	4.079385000000
C	0.522116000000	-1.208529000000	-1.778560000000	C	0.250629000000	1.715463000000	2.376441000000
C	1.853436000000	-2.743575000000	-0.458013000000	H	0.296862000000	-1.066858000000	4.359826000000
H	0.065052000000	-0.215763000000	-1.901805000000	H	0.313173000000	2.019781000000	1.323631000000
H	2.460762000000	-2.952537000000	0.434700000000	C	0.150040000000	0.978466000000	5.075590000000
C	0.336355000000	-2.204090000000	-2.751490000000	C	0.133459000000	2.700351000000	3.368681000000
C	1.671178000000	-3.737567000000	-1.428521000000	H	0.126044000000	0.680541000000	6.134624000000
H	-0.260981000000	-1.985577000000	-3.650727000000	H	0.069686000000	3.759037000000	3.074542000000
H	2.131650000000	-4.728040000000	-1.288926000000	C	0.089875000000	2.336656000000	4.723806000000
C	0.907145000000	-3.473657000000	-2.578003000000	H	0.006367000000	3.109002000000	5.503681000000
H	0.762587000000	-4.256398000000	-3.338836000000	N	-2.430600000000	0.978163000000	1.083542000000
C	3.719098000000	1.194102000000	2.961714000000	S	-2.139067000000	2.070027000000	-0.222422000000
C	4.192808000000	1.046280000000	4.364781000000	O	-1.020132000000	1.607835000000	-1.081963000000
C	3.641099000000	0.012913000000	5.158502000000	O	-2.097416000000	3.407908000000	0.412652000000
C	5.192966000000	1.861224000000	4.942098000000	C	-3.641662000000	1.896535000000	-1.176721000000
H	2.883022000000	-0.633119000000	4.693439000000	C	-3.636835000000	1.099936000000	-2.328871000000
H	5.674577000000	2.653643000000	4.350530000000	C	-4.812113000000	2.517651000000	-0.715935000000
C	4.054564000000	-0.179724000000	6.480185000000	H	-2.703719000000	0.619315000000	-2.654459000000
C	5.610319000000	1.665319000000	6.267483000000	H	-4.779828000000	3.139786000000	0.190009000000
H	3.604940000000	-0.987925000000	7.078092000000	C	-4.838431000000	0.915676000000	-3.024940000000
H	6.396194000000	2.310992000000	6.688738000000	C	-6.001690000000	2.319986000000	-1.425131000000
C	5.039125000000	0.649607000000	7.047464000000	H	-4.848240000000	0.279038000000	-3.923153000000
H	5.363852000000	0.498058000000	8.088072000000	H	-6.928609000000	2.797303000000	-1.070326000000
C	-5.042336000000	-2.495010000000	-0.512269000000	C	-6.037037000000	1.515756000000	-2.586934000000
C	-4.452518000000	-1.305265000000	-0.067066000000	C	-7.320301000000	1.327558000000	-3.353420000000
C	-4.326908000000	-3.716377000000	-0.522146000000	H	-8.204389000000	1.370368000000	-2.687565000000
H	-5.010729000000	-0.358220000000	-0.062900000000	H	-7.445275000000	2.129046000000	-4.113261000000
H	-4.820902000000	-4.629486000000	-0.886811000000	H	-7.335471000000	0.360834000000	-3.893407000000
C	-3.113827000000	-1.353177000000	0.373072000000	P	4.242889000000	2.692598000000	2.023971000000
C	-2.998908000000	-3.787340000000	-0.074124000000	C	3.280545000000	2.864158000000	0.483994000000
H	-2.424539000000	-4.723999000000	-0.072786000000	C	2.113687000000	3.660615000000	0.502943000000
C	-2.429621000000	-2.591892000000	0.369533000000	C	3.657056000000	2.202417000000	-0.700815000000
H	-6.085089000000	-2.481892000000	-0.864235000000	H	1.820752000000	4.190031000000	1.420112000000
C	-0.986371000000	-1.098340000000	1.149684000000	H	4.546322000000	1.561651000000	-0.728762000000
C	-2.153942000000	-0.393646000000	0.882057000000	C	1.313281000000	3.762628000000	-0.640922000000
O	-1.164655000000	-2.433013000000	0.867374000000	C	2.849831000000	2.312282000000	-1.842809000000
C	0.384724000000	-0.738869000000	1.651425000000	H	0.375757000000	4.335617000000	-0.599585000000

H	3.133252000000	1.766142000000	-2.754378000000
C	1.677866000000	3.080909000000	-1.812878000000
H	1.027935000000	3.133455000000	-2.698263000000
C	3.994178000000	4.301489000000	2.862151000000
C	4.380595000000	5.502108000000	2.226867000000
C	3.290478000000	4.347311000000	4.082220000000
H	4.881573000000	5.474343000000	1.247245000000
H	2.956858000000	3.415587000000	4.560335000000
C	4.096381000000	6.735249000000	2.829603000000
C	3.003977000000	5.586721000000	4.675025000000
H	4.399979000000	7.668821000000	2.332388000000
H	2.453930000000	5.617610000000	5.627576000000
C	3.411865000000	6.778020000000	4.056015000000
H	3.185759000000	7.747789000000	4.524727000000
C	6.001907000000	2.461088000000	1.600065000000
C	6.352646000000	1.196851000000	1.069126000000
C	6.996238000000	3.428696000000	1.851386000000
H	5.579328000000	0.420350000000	0.893960000000
H	6.732634000000	4.399823000000	2.294829000000
C	7.695745000000	0.933110000000	0.763627000000
C	8.336379000000	3.141967000000	1.553381000000
H	7.967049000000	-0.047194000000	0.343564000000
H	9.111629000000	3.896211000000	1.756499000000
C	8.686972000000	1.898323000000	1.004146000000
H	9.740033000000	1.677336000000	0.772409000000

II$_{P\text{-}SR}$

Electronic energy = -3255.182405 Hartree

C	1.226044000000	1.719645000000	5.852954000000
O	0.469235000000	1.660036000000	7.003980000000
C	2.553197000000	1.662834000000	5.903473000000
C	0.350766000000	1.809441000000	4.621967000000
H	1.080718000000	1.571415000000	7.762117000000
H	-0.607146000000	1.369446000000	4.969517000000
C	-0.008890000000	3.226634000000	4.194961000000
C	0.966838000000	4.148737000000	3.791914000000
C	-1.368771000000	3.585852000000	4.099023000000
H	2.026711000000	3.868151000000	3.864486000000
H	-2.143333000000	2.863740000000	4.403025000000
C	0.597549000000	5.382383000000	3.239260000000
C	-1.741686000000	4.835174000000	3.583191000000
H	1.389155000000	6.046674000000	2.864502000000
H	-2.807984000000	5.097849000000	3.504875000000
C	-0.756762000000	5.731833000000	3.135984000000
H	-1.049387000000	6.697919000000	2.695705000000
C	3.857963000000	1.726467000000	6.166467000000
C	4.798872000000	0.576308000000	6.118996000000
C	4.352382000000	-0.633155000000	5.536733000000
C	6.120699000000	0.630921000000	6.617867000000
H	3.328741000000	-0.683266000000	5.140304000000
H	6.507863000000	1.545627000000	7.085358000000
C	5.203113000000	-1.737135000000	5.437365000000
C	6.970727000000	-0.479367000000	6.519174000000
H	4.840413000000	-2.655090000000	4.953000000000
H	7.998143000000	-0.408448000000	6.906755000000
C	6.519962000000	-1.664528000000	5.921078000000
H	7.193443000000	-2.529362000000	5.825394000000
C	6.471983000000	-0.731710000000	1.907978000000
C	5.821287000000	0.457891000000	2.258539000000
C	5.758811000000	-1.946427000000	1.769369000000
H	6.377156000000	1.401037000000	2.352671000000
H	6.297816000000	-2.861653000000	1.480627000000
C	4.430669000000	0.421813000000	2.479348000000
C	4.375822000000	-2.007245000000	2.002707000000
H	3.805363000000	-2.943387000000	1.918423000000
C	3.753298000000	-0.809599000000	2.368435000000
H	7.557843000000	-0.721732000000	1.728718000000
C	2.243933000000	0.693496000000	3.060179000000
C	3.432286000000	1.416874000000	2.884586000000
O	2.449886000000	-0.655837000000	2.726887000000
C	0.795878000000	0.839515000000	3.470359000000
H	0.591020000000	-0.165862000000	3.895921000000
C	-0.163565000000	0.983546000000	2.290155000000
C	0.064585000000	1.922955000000	1.267220000000
C	-1.342175000000	0.213662000000	2.266114000000
H	0.977842000000	2.540337000000	1.269783000000
H	-1.521772000000	-0.528689000000	3.061989000000
C	-0.881550000000	2.086772000000	0.244281000000

C -2.286704000000 0.378400000000 1.241538000000
H -0.692695000000 2.829622000000 -0.545973000000
H -3.201494000000 -0.234490000000 1.234073000000
C -2.058185000000 1.320735000000 0.226704000000
H -2.796312000000 1.454510000000 -0.579369000000
N 3.750201000000 2.741309000000 3.166676000000
S 3.783304000000 3.832407000000 1.960801000000
O 4.151404000000 5.139016000000 2.587920000000
O 2.578993000000 3.795555000000 1.076354000000
C 5.148247000000 3.418621000000 0.845443000000
C 4.967187000000 2.427696000000 -0.133702000000
C 6.391638000000 4.046263000000 0.999638000000
H 3.974816000000 1.973357000000 -0.264491000000
H 6.486018000000 4.857390000000 1.736168000000
C 6.054986000000 2.031103000000 -0.918827000000
C 7.473343000000 3.640014000000 0.203417000000
H 5.915456000000 1.241874000000 -1.674764000000
H 8.451112000000 4.136130000000 0.319130000000
C 7.329691000000 2.616335000000 -0.755909000000
C 8.495921000000 2.155316000000 -1.593417000000
H 8.260992000000 2.202788000000 -2.676977000000
H 9.399271000000 2.769906000000 -1.413240000000
H 8.755317000000 1.098642000000 -1.369757000000
P 4.447556000000 3.385441000000 6.688997000000
C 4.506770000000 3.360960000000 8.519909000000
C 4.492290000000 2.147933000000 9.237250000000
C 4.550301000000 4.589076000000 9.215933000000
H 4.446283000000 1.188553000000 8.701889000000
H 4.528124000000 5.538394000000 8.659347000000
C 4.534001000000 2.166551000000 10.640000000000
C 4.598646000000 4.597596000000 10.616678000000
H 4.519686000000 1.217055000000 11.195730000000
H 4.631782000000 5.556804000000 11.154665000000
C 4.591730000000 3.387316000000 11.329689000000
H 4.623463000000 3.397136000000 12.429620000000
C 6.114568000000 3.773346000000 6.073325000000
C 7.111455000000 4.266841000000 6.941789000000
C 6.402244000000 3.496066000000 4.720919000000
H 6.886567000000 4.465619000000 7.999676000000
H 5.592263000000 3.122770000000 4.057929000000
C 8.406009000000 4.481981000000 6.450243000000
C 7.707664000000 3.704122000000 4.251433000000
H 9.186569000000 4.867615000000 7.123091000000
H 7.938733000000 3.475614000000 3.200574000000
C 8.705923000000 4.192813000000 5.108467000000
H 9.727865000000 4.348459000000 4.730176000000
C 3.301953000000 4.726592000000 6.302225000000
C 2.058478000000 4.786776000000 6.971033000000
C 3.697146000000 5.755673000000 5.427524000000
H 1.740313000000 3.977320000000 7.642810000000
H 4.633158000000 5.684906000000 4.859941000000
C 1.219657000000 5.886740000000 6.768830000000
C 2.850952000000 6.859118000000 5.245946000000
H 0.240308000000 5.924041000000 7.267290000000
H 3.153094000000 7.652249000000 4.547145000000
C 1.621967000000 6.928496000000 5.916071000000
H 0.955482000000 7.788604000000 5.754108000000

III*P-SR*

Electronic energy = -3255.198083 Hartree

C 2.122671000000 2.325219000000 5.267278000000
O 2.200266000000 3.334212000000 5.975495000000
C 3.342938000000 1.505341000000 5.133037000000
C 0.789216000000 1.907309000000 4.643735000000
H 3.263325000000 0.407768000000 5.100002000000
H 0.237800000000 1.467863000000 5.506579000000
C -0.000107000000 3.154136000000 4.268072000000
C 0.078662000000 3.738083000000 2.994554000000
C -0.763884000000 3.797776000000 5.262964000000
H 0.653793000000 3.263055000000 2.194367000000
H -0.832275000000 3.351417000000 6.267789000000
C -0.576455000000 4.947023000000 2.721146000000
C -1.425055000000 5.004672000000 4.992431000000
H -0.467823000000 5.387265000000 1.719156000000
H -2.018777000000 5.491531000000 5.781475000000
C -1.329694000000 5.586155000000 3.716687000000
H -1.845989000000 6.534715000000 3.501277000000
C 4.580070000000 2.074184000000 5.230331000000

C	5.797576000000	1.221394000000	5.299388000000
C	5.918224000000	0.110148000000	4.434183000000
C	6.830227000000	1.449419000000	6.240070000000
H	5.149848000000	-0.065647000000	3.672021000000
H	6.763220000000	2.292245000000	6.941611000000
C	7.033422000000	-0.735096000000	4.497263000000
C	7.942534000000	0.601248000000	6.303105000000
H	7.103447000000	-1.579899000000	3.795436000000
H	8.730431000000	0.798463000000	7.045749000000
C	8.053295000000	-0.492620000000	5.429438000000
H	8.933882000000	-1.151108000000	5.475064000000
C	6.035690000000	-0.400950000000	0.505522000000
C	5.347182000000	0.740605000000	0.933897000000
C	5.512467000000	-1.698670000000	0.717983000000
H	5.768114000000	1.741109000000	0.773319000000
H	6.075923000000	-2.575750000000	0.365162000000
C	4.106283000000	0.576382000000	1.584293000000
C	4.288397000000	-1.888645000000	1.378721000000
H	3.872613000000	-2.887683000000	1.571416000000
C	3.620236000000	-0.734267000000	1.798284000000
H	7.003447000000	-0.286943000000	-0.005703000000
C	2.185876000000	0.639638000000	2.801725000000
C	3.152843000000	1.481010000000	2.236447000000
O	2.470014000000	-0.698673000000	2.530494000000
C	0.918040000000	0.742578000000	3.606689000000
H	0.968555000000	-0.176486000000	4.231710000000
C	-0.349410000000	0.571416000000	2.766134000000
C	-0.387606000000	0.910745000000	1.401427000000
C	-1.516102000000	0.077829000000	3.381430000000
H	0.508825000000	1.322231000000	0.909931000000
H	-1.494985000000	-0.199034000000	4.448619000000
C	-1.577809000000	0.763835000000	0.672271000000
C	-2.704999000000	-0.066256000000	2.653048000000
H	-1.593992000000	1.042515000000	-0.392445000000
H	-3.608464000000	-0.452963000000	3.149310000000
C	-2.738794000000	0.277762000000	1.291966000000
H	-3.670619000000	0.166203000000	0.716565000000
N	3.269206000000	2.854177000000	2.379090000000
S	2.888786000000	3.740031000000	1.050591000000

O	2.740168000000	5.156296000000	1.462048000000
O	1.780749000000	3.103600000000	0.262052000000
C	4.306353000000	3.719129000000	-0.079960000000
C	4.275607000000	2.910432000000	-1.226340000000
C	5.448338000000	4.472673000000	0.227739000000
H	3.359298000000	2.345135000000	-1.447833000000
H	5.438364000000	5.130933000000	1.107434000000
C	5.410048000000	2.830973000000	-2.043633000000
C	6.580474000000	4.376868000000	-0.593370000000
H	5.387801000000	2.188086000000	-2.938336000000
H	7.480672000000	4.963416000000	-0.347452000000
C	6.585866000000	3.549519000000	-1.737232000000
C	7.800248000000	3.450948000000	-2.626224000000
H	7.606008000000	3.902630000000	-3.622202000000
H	8.673077000000	3.972335000000	-2.187348000000
H	8.086365000000	2.394125000000	-2.806598000000
P	4.848975000000	3.917662000000	5.258367000000
C	4.011073000000	5.303194000000	4.370830000000
C	2.687590000000	5.718376000000	4.631785000000
C	4.835766000000	6.114783000000	3.557497000000
H	2.024955000000	5.119447000000	5.263796000000
H	5.874323000000	5.832347000000	3.349619000000
C	2.203544000000	6.905088000000	4.070072000000
C	4.339882000000	7.305859000000	3.011772000000
H	1.158903000000	7.188576000000	4.264141000000
H	4.996073000000	7.915506000000	2.372704000000
C	3.021545000000	7.704142000000	3.261053000000
H	2.629367000000	8.631096000000	2.816575000000
C	4.903593000000	4.444743000000	7.007376000000
C	5.170310000000	5.797001000000	7.306235000000
C	4.662308000000	3.527274000000	8.047156000000
H	5.315070000000	6.522927000000	6.491613000000
H	4.412196000000	2.483748000000	7.806043000000
C	5.228731000000	6.215998000000	8.643297000000
C	4.717249000000	3.955160000000	9.380970000000
H	5.438049000000	7.271191000000	8.874363000000
H	4.522102000000	3.237137000000	10.191484000000
C	5.006363000000	5.296113000000	9.680627000000
H	5.045333000000	5.629296000000	10.728738000000

C	6.556705000000	3.982559000000	4.590207000000
C	6.745836000000	3.429054000000	3.305766000000
C	7.640979000000	4.533971000000	5.293281000000
H	5.878966000000	3.024038000000	2.756864000000
H	7.493364000000	4.960412000000	6.296464000000
C	8.025135000000	3.421078000000	2.737440000000
C	8.920286000000	4.520741000000	4.715315000000
H	8.165698000000	2.991627000000	1.734136000000
H	9.771594000000	4.946020000000	5.267836000000
C	9.113973000000	3.961594000000	3.442527000000
H	10.119973000000	3.947712000000	2.996292000000

X_P

Electronic energy = -3255.196532 Hartree

C	0.558101000000	3.722122000000	1.247054000000
O	0.135847000000	4.972476000000	0.846677000000
C	2.000946000000	3.540831000000	1.150043000000
C	-0.333571000000	2.734271000000	1.578371000000
H	2.477353000000	2.955400000000	1.943295000000
H	-0.845545000000	4.964344000000	0.899862000000
C	-1.775804000000	3.131739000000	1.594576000000
C	-2.274144000000	3.943877000000	2.641289000000
C	-2.668121000000	2.739762000000	0.567502000000
H	-1.588758000000	4.250160000000	3.447115000000
H	-2.299662000000	2.099095000000	-0.246868000000
C	-3.619333000000	4.345319000000	2.665867000000
C	-4.010928000000	3.141238000000	0.594091000000
H	-3.988325000000	4.970577000000	3.493344000000
H	-4.690094000000	2.817340000000	-0.209151000000
C	-4.491570000000	3.942119000000	1.643012000000
H	-5.547725000000	4.251347000000	1.663678000000
C	2.811499000000	3.899526000000	0.115856000000
C	4.259360000000	3.578180000000	0.118136000000
C	5.034479000000	3.706772000000	1.290719000000
C	4.867690000000	3.083754000000	-1.058219000000
H	4.565044000000	4.093134000000	2.208088000000
H	4.249084000000	2.885420000000	-1.946932000000
C	6.388129000000	3.345477000000	1.292519000000
C	6.223404000000	2.731092000000	-1.051321000000
H	6.975698000000	3.437542000000	2.218050000000
H	6.680643000000	2.321869000000	-1.965128000000
C	6.988823000000	2.862538000000	0.119505000000
H	8.051344000000	2.575442000000	0.121142000000
C	5.909947000000	-0.146116000000	2.357054000000
C	4.991767000000	0.088610000000	1.327432000000
C	5.508464000000	-0.169473000000	3.713722000000
H	5.324765000000	0.129174000000	0.284257000000
H	6.253722000000	-0.364285000000	4.500020000000
C	3.637028000000	0.298784000000	1.659517000000
C	4.174263000000	0.069687000000	4.073375000000
H	3.839956000000	0.082802000000	5.120634000000
C	3.276419000000	0.311485000000	3.027674000000
H	6.968854000000	-0.308402000000	2.103860000000
C	1.435537000000	0.830183000000	1.888557000000
C	2.416272000000	0.614541000000	0.904224000000
O	1.962515000000	0.639506000000	3.169426000000
C	-0.005674000000	1.275670000000	1.960606000000
H	-0.224778000000	1.220213000000	3.050762000000
C	-0.987318000000	0.294618000000	1.313223000000
C	-0.775204000000	-0.208499000000	0.017895000000
C	-2.149000000000	-0.089630000000	2.010680000000
H	0.134653000000	0.048970000000	-0.540087000000
H	-2.324052000000	0.300615000000	3.026346000000
C	-1.709101000000	-1.074121000000	-0.570688000000
C	-3.089126000000	-0.948244000000	1.421010000000
H	-1.500926000000	-1.467107000000	-1.577652000000
H	-3.992714000000	-1.236703000000	1.980169000000
C	-2.873489000000	-1.440951000000	0.123740000000
H	-3.606819000000	-2.120091000000	-0.338353000000
N	2.332487000000	0.956093000000	-0.426463000000
S	2.599254000000	0.098313000000	-1.758163000000
O	3.045163000000	1.070595000000	-2.810847000000
O	1.437315000000	-0.771410000000	-2.144251000000
C	3.956910000000	-1.058885000000	-1.505886000000
C	3.757374000000	-2.190242000000	-0.698749000000
C	5.210073000000	-0.791392000000	-2.072101000000
H	2.757204000000	-2.399434000000	-0.292138000000
H	5.318417000000	0.089604000000	-2.720637000000
C	4.842491000000	-3.026728000000	-0.418103000000

C	6.285635000000	-1.646168000000	-1.789547000000
H	4.695084000000	-3.904871000000	0.230582000000
H	7.274490000000	-1.440645000000	-2.230422000000
C	6.126463000000	-2.763456000000	-0.944658000000
C	1.367921000000	4.366314000000	-1.879858000000
C	-0.135594000000	5.031736000000	-3.693242000000
H	-0.952858000000	4.669329000000	-3.040889000000
H	-0.449889000000	6.026513000000	-4.068937000000
C	0.120471000000	4.047070000000	-4.836267000000
H	0.783804000000	4.505762000000	-5.601034000000
H	-0.853969000000	3.867169000000	-5.339480000000
C	0.711324000000	2.730932000000	-4.308141000000
H	1.811285000000	2.797596000000	-4.179734000000
H	0.557931000000	1.924537000000	-5.052688000000
C	0.093478000000	2.321287000000	-2.969286000000
H	0.320341000000	1.252577000000	-2.791081000000
H	-1.014508000000	2.393068000000	-3.033228000000
C	0.606264000000	3.092509000000	-1.722934000000
H	1.281284000000	2.405262000000	-1.148186000000
H	-0.228428000000	3.300775000000	-1.021980000000
C	1.892798000000	6.377560000000	-3.267824000000
H	1.985442000000	6.348631000000	-4.374346000000
H	1.364052000000	7.322665000000	-3.010566000000
C	3.255220000000	6.321871000000	-2.602362000000
H	3.770688000000	7.296207000000	-2.705236000000
H	3.895787000000	5.547949000000	-3.073331000000
C	3.050362000000	5.968611000000	-1.140184000000
H	2.425006000000	6.725507000000	-0.616422000000
H	4.011269000000	5.890508000000	-0.600762000000
N	1.053696000000	5.245537000000	-2.858871000000
N	2.376550000000	4.663681000000	-1.028780000000
C	7.293384000000	-3.650412000000	-0.591565000000
H	7.036363000000	-4.724142000000	-0.697818000000
H	8.175141000000	-3.442627000000	-1.228444000000
H	7.598969000000	-3.496839000000	0.465697000000

TS2*P-SR*

Electronic energy = -3255.191276 Hartree

C	0.173691000000	3.288031000000	0.829354000000
O	0.066872000000	3.996400000000	-0.169708000000
C	1.215532000000	3.564564000000	1.856405000000
C	-0.624086000000	2.009668000000	1.092339000000
H	0.978197000000	3.282964000000	2.897007000000
H	-1.432625000000	2.322938000000	1.790281000000
C	-1.329680000000	1.359303000000	-0.082819000000
C	-2.430410000000	0.522625000000	0.201049000000
C	-0.923137000000	1.501886000000	-1.423157000000
H	-2.759790000000	0.398999000000	1.245352000000
H	-0.071764000000	2.138311000000	-1.681689000000
C	-3.094330000000	-0.175490000000	-0.816332000000
C	-1.592405000000	0.807830000000	-2.443584000000
H	-3.942418000000	-0.831209000000	-0.566181000000
H	-1.250652000000	0.937673000000	-3.482176000000
C	-2.672781000000	-0.037359000000	-2.148350000000
H	-3.189991000000	-0.582979000000	-2.952958000000
C	2.120432000000	4.653622000000	1.703894000000
C	3.221161000000	4.814667000000	2.694284000000
C	2.954914000000	4.827870000000	4.084008000000
C	4.569418000000	4.913511000000	2.282047000000
H	1.912258000000	4.750551000000	4.431473000000
H	4.804246000000	4.850865000000	1.209334000000
C	3.989856000000	4.946115000000	5.020404000000
C	5.605163000000	5.049999000000	3.216748000000
H	3.753401000000	4.958055000000	6.095838000000
H	6.645665000000	5.120040000000	2.864459000000
C	5.322375000000	5.067483000000	4.590757000000
H	6.135789000000	5.166832000000	5.325448000000
C	6.399723000000	1.510761000000	1.407263000000
C	5.302030000000	1.492679000000	0.539942000000
C	6.226673000000	1.492419000000	2.810332000000
H	5.423754000000	1.538579000000	-0.550080000000
H	7.109917000000	1.520713000000	3.466553000000
C	4.000998000000	1.451264000000	1.093737000000
C	4.949953000000	1.457992000000	3.385706000000
H	4.792131000000	1.473423000000	4.472423000000
C	3.863993000000	1.436353000000	2.507276000000
H	7.416491000000	1.553485000000	0.989236000000

C	1.781325000000	1.584531000000	1.743500000000
C	2.632479000000	1.447466000000	0.566825000000
O	2.562880000000	1.434164000000	2.898609000000
C	0.354590000000	1.095223000000	1.942530000000
H	0.160358000000	1.351122000000	3.004318000000
C	0.165852000000	-0.406005000000	1.847453000000
C	0.426494000000	-1.122856000000	0.661446000000
C	-0.295628000000	-1.114203000000	2.974606000000
H	0.777602000000	-0.583433000000	-0.227095000000
H	-0.499469000000	-0.565411000000	3.908615000000
C	0.228971000000	-2.508905000000	0.610515000000
C	-0.494857000000	-2.502399000000	2.925589000000
H	0.437836000000	-3.043013000000	-0.328200000000
H	-0.854611000000	-3.036263000000	3.818575000000
C	-0.233107000000	-3.204349000000	1.739428000000
H	-0.389624000000	-4.293207000000	1.695042000000
N	2.058352000000	1.430163000000	-0.629799000000
S	2.790307000000	1.161541000000	-2.098231000000
O	4.199415000000	1.645818000000	-2.196380000000
O	1.832210000000	1.636081000000	-3.124716000000
C	2.807916000000	-0.635061000000	-2.162384000000
C	1.715415000000	-1.295591000000	-2.743993000000
C	3.871227000000	-1.351667000000	-1.592665000000
H	0.899602000000	-0.703043000000	-3.181854000000
H	4.727005000000	-0.816061000000	-1.157245000000
C	1.690326000000	-2.695875000000	-2.743454000000
C	3.822590000000	-2.751120000000	-1.593147000000
H	0.836555000000	-3.219954000000	-3.201678000000
H	4.652222000000	-3.320212000000	-1.144924000000
C	2.733392000000	-3.446758000000	-2.160114000000
C	2.664242000000	-4.951003000000	-2.105427000000
H	2.095720000000	-5.367216000000	-2.960197000000
H	3.672680000000	-5.409450000000	-2.100050000000
H	2.150437000000	-5.284049000000	-1.176899000000
P	1.804421000000	5.972765000000	0.563303000000
C	2.862250000000	7.368408000000	1.144138000000
C	4.109747000000	7.604627000000	0.530535000000
C	2.479118000000	8.152061000000	2.249684000000
H	4.419896000000	7.001804000000	-0.335136000000
H	1.506191000000	7.988598000000	2.733089000000
C	4.960687000000	8.604892000000	1.021205000000
C	3.333326000000	9.150963000000	2.737984000000
H	5.933122000000	8.777318000000	0.535834000000
H	3.022663000000	9.756536000000	3.602610000000
C	4.576335000000	9.377824000000	2.127295000000
H	5.245976000000	10.161426000000	2.513119000000
C	2.227695000000	5.882435000000	-1.213135000000
C	2.168925000000	7.054861000000	-2.000549000000
C	2.698464000000	4.684564000000	-1.770025000000
H	1.842164000000	8.008774000000	-1.558894000000
H	2.752734000000	3.779499000000	-1.150833000000
C	2.558290000000	7.006843000000	-3.345541000000
C	3.100058000000	4.644280000000	-3.114348000000
H	2.507406000000	7.918400000000	-3.960375000000
H	3.460797000000	3.693007000000	-3.527889000000
C	3.022853000000	5.801834000000	-3.901552000000
H	3.332732000000	5.768870000000	-4.957254000000
C	0.074265000000	6.575114000000	0.747706000000
C	-0.489573000000	6.565780000000	2.040463000000
C	-0.706988000000	6.989012000000	-0.347159000000
H	0.100318000000	6.187452000000	2.889555000000
H	-0.298509000000	6.942200000000	-1.365900000000
C	-1.807386000000	7.003538000000	2.238312000000
C	-2.025310000000	7.421281000000	-0.143357000000
H	-2.238402000000	6.994894000000	3.251042000000
H	-2.631932000000	7.733560000000	-1.006774000000
C	-2.576629000000	7.437387000000	1.147418000000
H	-3.613279000000	7.773121000000	1.301234000000

IV$_{P\text{-}SR}$

Electronic energy = -3255.211374 Hartree

C	-0.075497000000	3.353012000000	0.521217000000
O	-0.313278000000	4.171546000000	-0.348540000000
C	0.987411000000	3.534120000000	1.601435000000
C	-0.822653000000	2.028794000000	0.746406000000
H	0.356936000000	3.762193000000	2.510649000000
H	-1.755613000000	2.330278000000	1.272876000000
C	-1.288242000000	1.289246000000	-0.494653000000

C	-2.282820000000	0.299996000000	-0.343684000000
C	-0.769283000000	1.532435000000	-1.780243000000
H	-2.699191000000	0.101820000000	0.656753000000
H	0.007331000000	2.289374000000	-1.932975000000
C	-2.730667000000	-0.449342000000	-1.440040000000
C	-1.224590000000	0.788450000000	-2.880308000000
H	-3.499725000000	-1.223498000000	-1.295705000000
H	-0.803989000000	0.999607000000	-3.875657000000
C	-2.199007000000	-0.208341000000	-2.717003000000
H	-2.550638000000	-0.790356000000	-3.582912000000
C	2.076012000000	4.546776000000	1.361959000000
C	3.179717000000	4.622443000000	2.357938000000
C	2.919500000000	4.646937000000	3.753380000000
C	4.537262000000	4.683981000000	1.958774000000
H	1.878459000000	4.576585000000	4.106063000000
H	4.767667000000	4.607321000000	0.885909000000
C	3.952533000000	4.756161000000	4.692265000000
C	5.573391000000	4.816385000000	2.894621000000
H	3.711955000000	4.780564000000	5.767140000000
H	6.615985000000	4.871381000000	2.544531000000
C	5.289499000000	4.854815000000	4.268223000000
H	6.102245000000	4.949072000000	5.004445000000
C	5.966403000000	0.958228000000	2.253696000000
C	5.042772000000	1.120923000000	1.219923000000
C	5.568429000000	1.072780000000	3.606228000000
H	5.349490000000	1.095957000000	0.166952000000
H	6.319260000000	0.952861000000	4.402214000000
C	3.694897000000	1.400214000000	1.547031000000
C	4.246939000000	1.362741000000	3.958390000000
H	3.930022000000	1.488771000000	5.001645000000
C	3.328161000000	1.531315000000	2.914358000000
H	7.021001000000	0.759573000000	2.013545000000
C	1.417464000000	2.060625000000	1.820152000000
C	2.481446000000	1.653577000000	0.785013000000
O	2.033841000000	1.855805000000	3.109669000000
C	0.067070000000	1.281325000000	1.807355000000
H	-0.353235000000	1.548057000000	2.797925000000
C	0.213203000000	-0.222553000000	1.817147000000
C	0.486572000000	-0.989576000000	0.665712000000
C	0.132652000000	-0.889247000000	3.058840000000
H	0.558851000000	-0.499077000000	-0.311090000000
H	-0.072243000000	-0.301946000000	3.967905000000
C	0.666544000000	-2.376867000000	0.758107000000
C	0.318767000000	-2.275592000000	3.154372000000
H	0.876778000000	-2.948593000000	-0.157081000000
H	0.252785000000	-2.770481000000	4.135405000000
C	0.585253000000	-3.026125000000	1.999242000000
H	0.728368000000	-4.115649000000	2.066572000000
N	2.078236000000	1.603280000000	-0.452173000000
S	3.049708000000	1.230201000000	-1.804073000000
O	4.463726000000	1.650838000000	-1.640165000000
O	2.278572000000	1.739896000000	-2.960780000000
C	2.971250000000	-0.563533000000	-1.878377000000
C	1.917735000000	-1.153208000000	-2.594110000000
C	3.957305000000	-1.346267000000	-1.260538000000
H	1.163448000000	-0.514644000000	-3.074231000000
H	4.787027000000	-0.870110000000	-0.721231000000
C	1.854428000000	-2.548908000000	-2.681179000000
C	3.873904000000	-2.740961000000	-1.355967000000
H	1.029763000000	-3.014935000000	-3.243100000000
H	4.646594000000	-3.360491000000	-0.874606000000
C	2.825486000000	-3.366237000000	-2.063282000000
C	2.722169000000	-4.867917000000	-2.122855000000
H	2.208086000000	-5.207421000000	-3.043123000000
H	3.718211000000	-5.350278000000	-2.079410000000
H	2.136252000000	-5.253591000000	-1.259936000000
P	1.733133000000	6.000145000000	0.473790000000
C	2.998599000000	7.246258000000	0.955096000000
C	4.164556000000	7.376284000000	0.172726000000
C	2.878516000000	7.990479000000	2.144178000000
H	4.265702000000	6.798839000000	-0.757574000000
H	1.974266000000	7.904443000000	2.762071000000
C	5.197238000000	8.232882000000	0.580474000000
C	3.913350000000	8.845224000000	2.548645000000
H	6.105255000000	8.322254000000	-0.034902000000
H	3.810158000000	9.420166000000	3.481240000000
C	5.075791000000	8.965226000000	1.770702000000
H	5.888292000000	9.634157000000	2.093030000000

C	1.845236000000	6.043017000000	-1.359166000000
C	1.747306000000	7.261942000000	-2.063368000000
C	2.131672000000	4.856947000000	-2.050744000000
H	1.564710000000	8.203514000000	-1.522286000000
H	2.224948000000	3.918469000000	-1.488605000000
C	1.912225000000	7.276323000000	-3.456060000000
C	2.300245000000	4.872950000000	-3.443135000000
H	1.835635000000	8.227331000000	-4.005384000000
H	2.518256000000	3.923996000000	-3.953836000000
C	2.186196000000	6.082209000000	-4.145999000000
H	2.318941000000	6.099062000000	-5.238816000000
C	0.097400000000	6.730403000000	0.933753000000
C	-0.244349000000	6.678858000000	2.302263000000
C	-0.839668000000	7.229519000000	0.008770000000
H	0.466739000000	6.234751000000	3.016930000000
H	-0.610574000000	7.217768000000	-1.065913000000
C	-1.483878000000	7.165026000000	2.744707000000
C	-2.079919000000	7.708806000000	0.453910000000
H	-1.736366000000	7.125427000000	3.815430000000
H	-2.806796000000	8.094492000000	-0.277244000000
C	-2.402785000000	7.686488000000	1.820701000000
H	-3.379116000000	8.061316000000	2.163735000000

V$_{P\text{-}SR}$

Electronic energy = -3255.234956 Hartree

C	-0.177689000000	3.259877000000	1.116246000000
O	-0.712159000000	4.284020000000	0.559757000000
C	1.029296000000	3.319807000000	1.787049000000
C	-0.760846000000	1.847190000000	1.126262000000
H	1.125384000000	5.227100000000	2.772658000000
H	-1.745530000000	1.905557000000	1.640881000000
C	-1.057813000000	1.245868000000	-0.234393000000
C	-1.863765000000	0.091165000000	-0.324301000000
C	-0.569210000000	1.813795000000	-1.425156000000
H	-2.258129000000	-0.363153000000	0.598180000000
H	0.041112000000	2.722491000000	-1.373835000000
C	-2.153086000000	-0.493694000000	-1.564406000000
C	-0.869549000000	1.239953000000	-2.670850000000
H	-2.775226000000	-1.400811000000	-1.609121000000
H	-0.484591000000	1.713106000000	-3.587685000000
C	-1.655491000000	0.080039000000	-2.746485000000
H	-1.891429000000	-0.370278000000	-3.723303000000
C	1.659074000000	4.663261000000	1.967653000000
C	3.125530000000	4.640348000000	2.347571000000
C	3.470582000000	4.868101000000	3.694528000000
C	4.147836000000	4.289627000000	1.445664000000
H	2.680332000000	5.133214000000	4.414733000000
H	3.922874000000	4.087535000000	0.392641000000
C	4.794180000000	4.719397000000	4.133648000000
C	5.471524000000	4.144197000000	1.877628000000
H	5.037486000000	4.878267000000	5.194998000000
H	6.237274000000	3.834756000000	1.151003000000
C	5.799535000000	4.352211000000	3.226306000000
H	6.835483000000	4.219410000000	3.572536000000
C	5.497716000000	0.673462000000	4.458232000000
C	5.009106000000	0.795767000000	3.155635000000
C	4.692749000000	1.019235000000	5.568337000000
H	5.624447000000	0.545405000000	2.283406000000
H	5.101575000000	0.917045000000	6.585863000000
C	3.687886000000	1.262107000000	2.959530000000
C	3.388165000000	1.494083000000	5.404127000000
H	2.752587000000	1.773855000000	6.255645000000
C	2.899186000000	1.610991000000	4.091265000000
H	6.522068000000	0.308277000000	4.622040000000
C	1.480362000000	2.017566000000	2.317357000000
C	2.851197000000	1.509348000000	1.795592000000
O	1.676212000000	2.067694000000	3.800216000000
C	0.216397000000	1.081281000000	2.097812000000
H	-0.258791000000	1.143769000000	3.094701000000
C	0.525772000000	-0.378787000000	1.870668000000
C	0.981038000000	-0.879385000000	0.633234000000
C	0.397870000000	-1.282088000000	2.946839000000
H	1.093385000000	-0.197673000000	-0.215913000000
H	0.046325000000	-0.906397000000	3.921212000000
C	1.299769000000	-2.234949000000	0.477934000000
C	0.716172000000	-2.640377000000	2.796703000000
H	1.658351000000	-2.591958000000	-0.499080000000

H	0.610253000000	-3.325035000000	3.652234000000
C	1.169402000000	-3.121453000000	1.558685000000
H	1.421007000000	-4.186304000000	1.436775000000
N	2.967917000000	1.442034000000	0.500321000000
S	4.343768000000	1.135189000000	-0.419857000000
O	5.528015000000	0.559106000000	0.271652000000
O	4.549750000000	2.375291000000	-1.219017000000
C	3.695865000000	-0.137605000000	-1.506810000000
C	2.559184000000	0.113765000000	-2.292763000000
C	4.354317000000	-1.371995000000	-1.555772000000
H	2.039056000000	1.079080000000	-2.228763000000
H	5.235054000000	-1.534971000000	-0.918742000000
C	2.072338000000	-0.900698000000	-3.121328000000
C	3.863331000000	-2.370777000000	-2.409810000000
H	1.159414000000	-0.717621000000	-3.708736000000
H	4.375837000000	-3.344566000000	-2.453720000000
C	2.715043000000	-2.157423000000	-3.198163000000
C	2.166053000000	-3.237409000000	-4.094156000000
H	2.158017000000	-2.912366000000	-5.155477000000
H	2.760226000000	-4.168798000000	-4.027473000000
H	1.116399000000	-3.479731000000	-3.826700000000
P	1.185674000000	5.807541000000	0.496220000000
C	2.663806000000	6.930330000000	0.454631000000
C	3.532511000000	7.022453000000	-0.648227000000
C	2.945746000000	7.684155000000	1.615705000000
H	3.328527000000	6.446086000000	-1.561501000000
H	2.269558000000	7.634204000000	2.483310000000
C	4.672058000000	7.841513000000	-0.585292000000
C	4.084607000000	8.494953000000	1.677772000000
H	5.345123000000	7.900935000000	-1.453907000000
H	4.297309000000	9.067894000000	2.592897000000
C	4.954217000000	8.572919000000	0.576630000000
H	5.851823000000	9.207701000000	0.626572000000
C	1.095447000000	5.182631000000	-1.210265000000
C	0.078790000000	5.648711000000	-2.069741000000
C	2.044763000000	4.255998000000	-1.681165000000
H	-0.690462000000	6.336633000000	-1.691392000000
H	2.815515000000	3.821235000000	-1.032757000000
C	0.040478000000	5.212246000000	-3.399877000000
C	2.003151000000	3.833047000000	-3.020247000000
H	-0.756780000000	5.576283000000	-4.065240000000
H	2.767060000000	3.120873000000	-3.365437000000
C	1.008355000000	4.313183000000	-3.883226000000
H	0.978549000000	3.983264000000	-4.932933000000
C	-0.114580000000	7.044201000000	0.877422000000
C	-1.158774000000	6.778193000000	1.784231000000
C	-0.039302000000	8.304883000000	0.247145000000
H	-1.242117000000	5.781204000000	2.234097000000
H	0.783120000000	8.524762000000	-0.449637000000
C	-2.105039000000	7.772982000000	2.069058000000
C	-1.008045000000	9.283073000000	0.513480000000
H	-2.912741000000	7.562069000000	2.786011000000
H	-0.949767000000	10.258592000000	0.007642000000
C	-2.037253000000	9.021804000000	1.431380000000
H	-2.790560000000	9.794197000000	1.649230000000

TS4$_{P\text{-}SR}$

Electronic energy = -3255.223842 Hartree

C	-0.576568000000	2.966523000000	1.323153000000
O	-1.357693000000	3.842789000000	0.905639000000
C	0.733891000000	3.194492000000	1.855055000000
C	-0.898251000000	1.461906000000	1.306986000000
H	0.343752000000	5.201198000000	2.368479000000
H	-1.842849000000	1.342597000000	1.879955000000
C	-1.202913000000	0.909559000000	-0.075076000000
C	-1.815106000000	-0.357108000000	-0.191135000000
C	-0.945773000000	1.637265000000	-1.253242000000
H	-2.029264000000	-0.935375000000	0.720994000000
H	-0.498745000000	2.638280000000	-1.191556000000
C	-2.144892000000	-0.891031000000	-1.443679000000
C	-1.291662000000	1.112006000000	-2.509228000000
H	-2.616579000000	-1.883624000000	-1.507595000000
H	-1.094234000000	1.709632000000	-3.412044000000
C	-1.886243000000	-0.154718000000	-2.611558000000
H	-2.160915000000	-0.562563000000	-3.596859000000
C	1.174090000000	4.522906000000	2.097094000000
C	2.457804000000	4.825902000000	2.780508000000
C	2.442923000000	5.541244000000	3.998093000000

C	3.703548000000	4.429907000000	2.244888000000	C	3.505305000000	0.095370000000	-1.755677000000
H	1.477994000000	5.867508000000	4.416504000000	C	2.244032000000	0.287039000000	-2.342487000000
H	3.746134000000	3.931239000000	1.266414000000	C	4.286920000000	-1.023116000000	-2.073622000000
C	3.635845000000	5.816319000000	4.680918000000	H	1.621258000000	1.146455000000	-2.061181000000
C	4.895399000000	4.709350000000	2.924791000000	H	5.259383000000	-1.156552000000	-1.579318000000
H	3.605024000000	6.361992000000	5.636420000000	C	1.764902000000	-0.663622000000	-3.248563000000
H	5.853353000000	4.391002000000	2.488172000000	C	3.797162000000	-1.957082000000	-2.998130000000
C	4.866037000000	5.397110000000	4.147495000000	H	0.759201000000	-0.528519000000	-3.674892000000
H	5.803007000000	5.614287000000	4.682473000000	H	4.407370000000	-2.839215000000	-3.248039000000
C	5.637428000000	0.986775000000	4.178558000000	C	2.530958000000	-1.798759000000	-3.596071000000
C	5.052481000000	1.020296000000	2.911010000000	C	1.990303000000	-2.810924000000	-4.572416000000
C	4.883137000000	1.294128000000	5.335064000000	H	1.838194000000	-2.357425000000	-5.574065000000
H	5.631239000000	0.798394000000	2.006798000000	H	2.672672000000	-3.674138000000	-4.690763000000
H	5.370947000000	1.268898000000	6.321971000000	H	1.002849000000	-3.194509000000	-4.242709000000
C	3.683440000000	1.359259000000	2.797662000000	P	1.341900000000	5.861407000000	0.154590000000
C	3.530549000000	1.636706000000	5.252875000000	C	3.009329000000	6.598358000000	0.056629000000
H	2.931352000000	1.891652000000	6.137523000000	C	3.942638000000	6.269629000000	-0.948422000000
C	2.947632000000	1.664374000000	3.975202000000	C	3.420093000000	7.418930000000	1.135680000000
H	6.700232000000	0.721969000000	4.277801000000	H	3.651077000000	5.627260000000	-1.789958000000
C	1.390751000000	1.926723000000	2.302024000000	H	2.714015000000	7.663857000000	1.942522000000
C	2.757757000000	1.549049000000	1.688575000000	C	5.257281000000	6.753333000000	-0.873620000000
O	1.673330000000	2.008280000000	3.758025000000	C	4.730565000000	7.904908000000	1.197689000000
C	0.244703000000	0.828367000000	2.168738000000	H	5.975189000000	6.482136000000	-1.662165000000
H	-0.148303000000	0.820809000000	3.202662000000	H	5.034030000000	8.536149000000	2.046216000000
C	0.739904000000	-0.572160000000	1.895866000000	C	5.656474000000	7.568957000000	0.195869000000
C	1.138465000000	-1.002494000000	0.613484000000	H	6.691200000000	7.939459000000	0.252331000000
C	0.870658000000	-1.477120000000	2.970258000000	C	0.929929000000	5.161117000000	-1.474887000000
H	1.048940000000	-0.318029000000	-0.235033000000	C	-0.172371000000	5.633409000000	-2.221000000000
H	0.567128000000	-1.158103000000	3.980331000000	C	1.723113000000	4.111401000000	-1.988284000000
C	1.656523000000	-2.288211000000	0.410577000000	H	-0.810805000000	6.431422000000	-1.816521000000
C	1.386594000000	-2.766996000000	2.772697000000	H	2.559597000000	3.685229000000	-1.414615000000
H	1.968028000000	-2.586015000000	-0.602470000000	C	-0.449093000000	5.088414000000	-3.481357000000
H	1.481184000000	-3.454498000000	3.627192000000	C	1.460169000000	3.602867000000	-3.270009000000
C	1.783615000000	-3.176507000000	1.490172000000	H	-1.310259000000	5.462796000000	-4.055210000000
H	2.192303000000	-4.186555000000	1.333295000000	H	2.112663000000	2.815039000000	-3.673989000000
N	2.810961000000	1.507369000000	0.388107000000	C	0.376732000000	4.086122000000	-4.018493000000
S	4.173549000000	1.273883000000	-0.575004000000	H	0.171211000000	3.680051000000	-5.020816000000
O	5.356189000000	0.615468000000	0.039619000000	C	0.189853000000	7.255726000000	0.420562000000
O	4.385375000000	2.590125000000	-1.239698000000	C	-1.121517000000	6.948121000000	0.851327000000

C	0.555937000000	8.599764000000	0.189429000000
H	-1.407655000000	5.890579000000	0.994141000000
H	1.569200000000	8.843107000000	-0.161481000000
C	-2.046525000000	7.984393000000	1.054096000000
C	-0.376770000000	9.624854000000	0.399650000000
H	-3.067170000000	7.739300000000	1.385332000000
H	-0.086077000000	10.670175000000	0.214131000000
C	-1.677417000000	9.320571000000	0.835570000000
H	-2.405831000000	10.129483000000	0.999471000000

4a*SR*

Electronic energy = -2219.065023 Hartree

C	-0.503116000000	2.818314000000	1.269917000000
O	-1.235045000000	3.634730000000	0.732399000000
C	0.862966000000	3.101811000000	1.803764000000
C	-0.845247000000	1.334936000000	1.503004000000
H	0.633551000000	5.127453000000	1.447078000000
H	-1.842060000000	1.311175000000	1.989743000000
C	-0.974160000000	0.532080000000	0.219527000000
C	-1.718967000000	-0.664841000000	0.236453000000
C	-0.359790000000	0.927130000000	-0.983739000000
H	-2.207346000000	-0.982718000000	1.170807000000
H	0.226678000000	1.854533000000	-1.024160000000
C	-1.827969000000	-1.460585000000	-0.912328000000
C	-0.470835000000	0.134931000000	-2.135768000000
H	-2.408164000000	-2.395240000000	-0.876336000000
H	0.024878000000	0.458587000000	-3.062669000000
C	-1.198607000000	-1.064293000000	-2.103023000000
H	-1.280404000000	-1.687157000000	-3.006940000000
C	1.360347000000	4.367828000000	1.795607000000
C	2.673489000000	4.848413000000	2.231329000000
C	2.749657000000	6.050690000000	2.976997000000
C	3.876748000000	4.176704000000	1.905557000000
H	1.822991000000	6.598664000000	3.207798000000
H	3.867966000000	3.301183000000	1.243308000000
C	3.980360000000	6.523992000000	3.446284000000
C	5.107754000000	4.664866000000	2.360675000000
H	4.019316000000	7.446988000000	4.044460000000
H	6.029475000000	4.130221000000	2.086305000000
C	5.163629000000	5.827699000000	3.145624000000
H	6.132337000000	6.204360000000	3.507807000000
C	5.660308000000	0.170932000000	3.676549000000
C	4.889004000000	0.245808000000	2.513747000000
C	5.204719000000	0.746539000000	4.885663000000
H	5.230284000000	-0.205796000000	1.573987000000
H	5.835417000000	0.676313000000	5.785343000000
C	3.643018000000	0.906098000000	2.579361000000
C	3.969083000000	1.400274000000	4.973703000000
H	3.606013000000	1.849368000000	5.908003000000
C	3.198037000000	1.463011000000	3.805322000000
H	6.631020000000	-0.344811000000	3.653262000000
C	1.456282000000	1.845007000000	2.388884000000
C	2.623137000000	1.239131000000	1.598203000000
O	1.993677000000	2.069281000000	3.725802000000
C	0.216417000000	0.880475000000	2.555085000000
H	-0.184597000000	1.214453000000	3.533526000000
C	0.568742000000	-0.581800000000	2.703547000000
C	1.071991000000	-1.362621000000	1.638604000000
C	0.446177000000	-1.183238000000	3.973163000000
H	1.190399000000	-0.925395000000	0.640504000000
H	0.059223000000	-0.584437000000	4.813257000000
C	1.453086000000	-2.694415000000	1.849341000000
C	0.817800000000	-2.519561000000	4.182865000000
H	1.861770000000	-3.273002000000	1.007508000000
H	0.714787000000	-2.965505000000	5.183891000000
C	1.326926000000	-3.279370000000	3.119066000000
H	1.628216000000	-4.325717000000	3.280170000000
N	2.525387000000	1.197709000000	0.302957000000
S	3.821516000000	0.615426000000	-0.629451000000
O	4.050106000000	-0.833745000000	-0.392622000000
O	4.948904000000	1.574117000000	-0.498975000000
C	3.111095000000	0.799316000000	-2.263363000000
C	2.731512000000	2.071442000000	-2.721517000000
C	3.002877000000	-0.331958000000	-3.078451000000
H	2.832306000000	2.948165000000	-2.065433000000
H	3.307844000000	-1.310931000000	-2.682379000000
C	2.217229000000	2.195905000000	-4.015382000000
C	2.499011000000	-0.184544000000	-4.379505000000

H	1.904128000000	3.187272000000	-4.378808000000
H	2.410267000000	-1.069933000000	-5.028116000000
C	2.093941000000	1.073199000000	-4.867604000000
C	1.535669000000	1.231744000000	-6.258156000000
H	2.143545000000	1.942998000000	-6.855058000000
H	1.507340000000	0.267829000000	-6.800883000000
H	0.503539000000	1.639055000000	-6.229819000000

TS3*P-SR*

Electronic energy = -3255.199823 Hartree

C	0.186033000000	2.899577000000	-0.558441000000
O	-0.961653000000	2.544989000000	-0.804761000000
C	1.192670000000	2.875443000000	-1.690187000000
C	0.631581000000	3.279526000000	0.856585000000
H	0.774180000000	2.308726000000	-2.536653000000
H	1.562175000000	3.875468000000	0.777095000000
C	-0.348140000000	4.075057000000	1.702531000000
C	0.194931000000	4.893741000000	2.717734000000
C	-1.748802000000	3.963297000000	1.609226000000
H	1.290629000000	4.986160000000	2.804414000000
H	-2.174585000000	3.312064000000	0.835849000000
C	-0.631207000000	5.584215000000	3.613719000000
C	-2.577405000000	4.669192000000	2.496976000000
H	-0.183209000000	6.213377000000	4.398362000000
H	-3.670277000000	4.570210000000	2.407166000000
C	-2.027343000000	5.476951000000	3.502956000000
H	-2.682470000000	6.016605000000	4.204047000000
C	2.085933000000	3.907256000000	-2.071468000000
C	2.920586000000	3.660277000000	-3.294527000000
C	2.434345000000	3.936932000000	-4.588353000000
C	4.215762000000	3.119686000000	-3.158007000000
H	1.419335000000	4.348369000000	-4.700096000000
H	4.592995000000	2.876718000000	-2.153468000000
C	3.229569000000	3.697353000000	-5.716965000000
C	5.012822000000	2.876749000000	-4.286378000000
H	2.836515000000	3.918921000000	-6.721149000000
H	6.016273000000	2.442301000000	-4.161230000000
C	4.523620000000	3.170971000000	-5.567721000000
H	5.146446000000	2.978198000000	-6.454596000000
C	6.726333000000	0.505710000000	0.045858000000
C	5.479428000000	0.617858000000	-0.583178000000
C	6.874287000000	0.670378000000	1.442497000000
H	5.359519000000	0.459315000000	-1.662352000000
H	7.868016000000	0.563155000000	1.903014000000
C	4.344873000000	0.914505000000	0.207404000000
C	5.767101000000	0.966633000000	2.250652000000
H	5.848444000000	1.101261000000	3.338782000000
C	4.533434000000	1.092080000000	1.603976000000
H	7.613328000000	0.275821000000	-0.563799000000
C	2.384871000000	1.455137000000	1.233042000000
C	2.910783000000	1.146889000000	-0.020662000000
O	3.362606000000	1.440353000000	2.211877000000
C	1.069274000000	1.994183000000	1.693679000000
H	1.288721000000	2.407349000000	2.698019000000
C	-0.057662000000	0.991418000000	1.875032000000
C	-0.328890000000	-0.005934000000	0.919973000000
C	-0.874989000000	1.079228000000	3.020205000000
H	0.296137000000	-0.077893000000	0.019968000000
H	-0.671240000000	1.859074000000	3.770782000000
C	-1.393192000000	-0.898333000000	1.111009000000
C	-1.939757000000	0.186856000000	3.211528000000
H	-1.588440000000	-1.670266000000	0.352406000000
H	-2.565362000000	0.267985000000	4.113685000000
C	-2.201689000000	-0.805894000000	2.254838000000
H	-3.036571000000	-1.508514000000	2.401382000000
N	2.140156000000	1.210900000000	-1.171224000000
S	2.286833000000	0.124599000000	-2.422183000000
O	3.701455000000	-0.173643000000	-2.765469000000
O	1.374193000000	0.592259000000	-3.495033000000
C	1.608485000000	-1.407574000000	-1.764703000000
C	0.318875000000	-1.801539000000	-2.150034000000
C	2.358236000000	-2.170405000000	-0.855591000000
H	-0.232429000000	-1.186194000000	-2.875483000000
H	3.381440000000	-1.865243000000	-0.592697000000
C	-0.227820000000	-2.968910000000	-1.602049000000
C	1.785287000000	-3.322428000000	-0.302274000000
H	-1.235923000000	-3.289641000000	-1.910656000000
H	2.363915000000	-3.919141000000	0.420486000000

C	0.484707000000	-3.738333000000	-0.657562000000
C	-0.145988000000	-4.948275000000	-0.017791000000
H	-0.773964000000	-4.646451000000	0.848859000000
H	-0.805823000000	-5.489462000000	-0.724807000000
H	0.615685000000	-5.657395000000	0.360870000000
P	2.328655000000	5.473265000000	-1.324395000000
C	0.754902000000	6.165420000000	-0.704634000000
C	0.697632000000	7.031841000000	0.403531000000
C	-0.427179000000	5.850417000000	-1.410406000000
H	1.606371000000	7.258294000000	0.977916000000
H	-0.384510000000	5.160305000000	-2.265166000000
C	-0.531012000000	7.576338000000	0.802683000000
C	-1.651379000000	6.392550000000	-0.999032000000
H	-0.573568000000	8.232076000000	1.684175000000
H	-2.571326000000	6.122953000000	-1.538149000000
C	-1.705014000000	7.255238000000	0.106832000000
H	-2.669942000000	7.665501000000	0.439328000000
C	3.610806000000	5.649031000000	-0.016229000000
C	4.178422000000	4.491903000000	0.546421000000
C	4.080818000000	6.923965000000	0.368822000000
H	3.838446000000	3.500183000000	0.209465000000
H	3.677697000000	7.831062000000	-0.107578000000
C	5.191699000000	4.603874000000	1.511773000000
C	5.085244000000	7.030779000000	1.340748000000
H	5.638195000000	3.689940000000	1.931233000000
H	5.449636000000	8.024296000000	1.642904000000
C	5.637337000000	5.871784000000	1.914548000000
H	6.433298000000	5.960226000000	2.669414000000
C	2.913992000000	6.601142000000	-2.642501000000
C	4.270978000000	6.561853000000	-3.024176000000
C	2.007779000000	7.417453000000	-3.344607000000
H	4.979844000000	5.922261000000	-2.478763000000
H	0.949913000000	7.452705000000	-3.045476000000
C	4.709480000000	7.321115000000	-4.116630000000
C	2.457263000000	8.183762000000	-4.430595000000
H	5.765397000000	7.274421000000	-4.421932000000
H	1.747260000000	8.823400000000	-4.976218000000
C	3.804020000000	8.130659000000	-4.821642000000
H	4.151376000000	8.725052000000	-5.680264000000

IV'$_{P\text{-}SR}$

Electronic energy = -3255.207914 Hartree

C	0.460885000000	2.765158000000	-0.532174000000
O	-0.697103000000	2.424598000000	-0.736232000000
C	1.476130000000	2.690652000000	-1.711797000000
C	0.989057000000	3.139371000000	0.853453000000
H	0.826630000000	2.554839000000	-2.600822000000
H	1.816730000000	3.846989000000	0.666002000000
C	0.017366000000	3.835196000000	1.786501000000
C	0.554810000000	4.767062000000	2.702128000000
C	-1.365539000000	3.572887000000	1.827356000000
H	1.636348000000	4.978353000000	2.688320000000
H	-1.787244000000	2.845201000000	1.124230000000
C	-0.266204000000	5.436674000000	3.619386000000
C	-2.188958000000	4.250294000000	2.740466000000
H	0.175749000000	6.162583000000	4.319192000000
H	-3.269280000000	4.038495000000	2.752827000000
C	-1.648229000000	5.183405000000	3.638033000000
H	-2.298569000000	5.707589000000	4.355099000000
C	2.527779000000	3.773932000000	-1.908719000000
C	3.683268000000	3.508107000000	-2.789058000000
C	3.565518000000	2.685296000000	-3.942625000000
C	4.956379000000	4.093398000000	-2.553410000000
H	2.602581000000	2.217306000000	-4.186687000000
H	5.109819000000	4.712149000000	-1.656553000000
C	4.655918000000	2.460249000000	-4.792851000000
C	6.038369000000	3.886197000000	-3.417764000000
H	4.521846000000	1.808911000000	-5.670929000000
H	7.006476000000	4.360271000000	-3.192811000000
C	5.902186000000	3.059287000000	-4.543767000000
H	6.755384000000	2.881401000000	-5.215557000000
C	6.757867000000	0.444557000000	-1.477244000000
C	5.398289000000	0.610589000000	-1.760150000000
C	7.262829000000	0.549610000000	-0.159504000000
H	5.008449000000	0.527795000000	-2.781017000000
H	8.338481000000	0.406291000000	0.023443000000
C	4.525926000000	0.901230000000	-0.690845000000
C	6.415646000000	0.840013000000	0.919184000000

H	6.782967000000	0.933981000000	1.950833000000
C	5.063166000000	1.015947000000	0.613861000000
H	7.452446000000	0.232311000000	-2.303623000000
C	2.901412000000	1.459092000000	0.805556000000
C	3.103779000000	1.180918000000	-0.537979000000
O	4.081462000000	1.366266000000	1.509501000000
C	1.735171000000	1.958359000000	1.601611000000
H	2.199675000000	2.468265000000	2.471358000000
C	0.841581000000	0.869854000000	2.188107000000
C	0.768969000000	-0.419023000000	1.632552000000
C	0.112098000000	1.137114000000	3.367794000000
H	1.354033000000	-0.656360000000	0.733995000000
H	0.185530000000	2.126667000000	3.841670000000
C	-0.018512000000	-1.414554000000	2.231496000000
C	-0.691428000000	0.150525000000	3.954392000000
H	-0.038358000000	-2.420640000000	1.789784000000
H	-1.253379000000	0.382106000000	4.872117000000
C	-0.760251000000	-1.132035000000	3.387150000000
H	-1.376763000000	-1.914194000000	3.856491000000
N	2.121119000000	1.352791000000	-1.535332000000
S	1.717406000000	0.076371000000	-2.616353000000
O	2.944336000000	-0.713848000000	-2.840573000000
O	0.980882000000	0.707296000000	-3.736432000000
C	0.546050000000	-1.006271000000	-1.794532000000
C	-0.774920000000	-0.577031000000	-1.588656000000
C	0.955093000000	-2.304377000000	-1.458657000000
H	-1.075392000000	0.447004000000	-1.851237000000
H	1.992225000000	-2.610759000000	-1.655028000000
C	-1.687951000000	-1.468797000000	-1.018148000000
C	0.018876000000	-3.184391000000	-0.897644000000
H	-2.722292000000	-1.135586000000	-0.839920000000
H	0.330388000000	-4.208323000000	-0.637199000000
C	-1.312396000000	-2.783178000000	-0.664394000000
C	-2.302490000000	-3.709770000000	-0.009448000000
H	-2.435245000000	-3.439672000000	1.061043000000
H	-3.301977000000	-3.640465000000	-0.483382000000
H	-1.969729000000	-4.764921000000	-0.048994000000
P	2.341288000000	5.395423000000	-1.365061000000
C	0.551381000000	5.787896000000	-1.181929000000
C	-0.027917000000	6.412314000000	-0.060614000000
C	-0.276553000000	5.398571000000	-2.258556000000
H	0.585687000000	6.699505000000	0.800856000000
H	0.167919000000	4.891239000000	-3.127236000000
C	-1.412665000000	6.632562000000	-0.011966000000
C	-1.657208000000	5.621979000000	-2.206844000000
H	-1.852821000000	7.092834000000	0.884812000000
H	-2.291801000000	5.298574000000	-3.045157000000
C	-2.228480000000	6.237514000000	-1.081134000000
H	-3.316112000000	6.398695000000	-1.033685000000
C	3.166289000000	5.885896000000	0.233064000000
C	3.981112000000	4.903226000000	0.831030000000
C	3.006696000000	7.132811000000	0.874869000000
H	4.104487000000	3.934274000000	0.319449000000
H	2.394168000000	7.922766000000	0.414881000000
C	4.603173000000	5.145179000000	2.064600000000
C	3.628570000000	7.373238000000	2.110627000000
H	5.225317000000	4.360004000000	2.519864000000
H	3.491928000000	8.344332000000	2.610675000000
C	4.419899000000	6.378732000000	2.710098000000
H	4.898852000000	6.569999000000	3.682417000000
C	3.023035000000	6.562009000000	-2.619204000000
C	3.821558000000	7.673199000000	-2.291183000000
C	2.711397000000	6.305591000000	-3.970766000000
H	4.106145000000	7.865117000000	-1.247625000000
H	2.135636000000	5.406661000000	-4.233940000000
C	4.286796000000	8.528429000000	-3.302772000000
C	3.178132000000	7.160896000000	-4.975727000000
H	4.916221000000	9.391323000000	-3.037294000000
H	2.939413000000	6.944299000000	-6.027737000000
C	3.963593000000	8.277313000000	-4.644003000000
H	4.335225000000	8.945469000000	-5.435688000000

TS5*P-SR*

Electronic energy = -3255.194937 Hartree

C	0.407325000000	3.152738000000	-0.553903000000
O	-0.563517000000	3.638143000000	-1.177092000000
C	1.715691000000	3.045988000000	-1.170535000000
C	0.220029000000	3.074017000000	0.999210000000

H	0.990904000000	4.203051000000	-2.791891000000
H	0.624275000000	4.083809000000	1.243792000000
C	-1.250255000000	3.122262000000	1.358846000000
C	-1.732204000000	4.099603000000	2.247802000000
C	-2.152104000000	2.159498000000	0.865619000000
H	-1.036828000000	4.861198000000	2.636218000000
H	-1.785118000000	1.384357000000	0.179279000000
C	-3.079166000000	4.114946000000	2.646515000000
C	-3.497102000000	2.172782000000	1.254390000000
H	-3.435789000000	4.886428000000	3.346775000000
H	-4.186597000000	1.410222000000	0.860445000000
C	-3.966841000000	3.148691000000	2.149928000000
H	-5.023773000000	3.156122000000	2.457682000000
C	1.954360000000	3.950745000000	-2.308409000000
C	3.027435000000	3.714490000000	-3.333576000000
C	2.700627000000	3.827782000000	-4.700855000000
C	4.366141000000	3.439937000000	-2.978616000000
H	1.657866000000	4.034300000000	-4.985538000000
H	4.640492000000	3.336775000000	-1.921753000000
C	3.678499000000	3.667261000000	-5.690621000000
C	5.346285000000	3.286403000000	-3.969624000000
H	3.400489000000	3.751172000000	-6.752203000000
H	6.384245000000	3.069495000000	-3.674256000000
C	5.008887000000	3.401047000000	-5.327920000000
H	5.780282000000	3.275221000000	-6.103079000000
C	6.894461000000	1.076743000000	0.785725000000
C	5.718535000000	1.269966000000	0.050312000000
C	6.889039000000	1.063394000000	2.201171000000
H	5.707958000000	1.246534000000	-1.047058000000
H	7.831499000000	0.900217000000	2.745384000000
C	4.515501000000	1.462252000000	0.759092000000
C	5.701048000000	1.247005000000	2.925863000000
H	5.674111000000	1.241512000000	4.024587000000
C	4.539084000000	1.445046000000	2.174324000000
H	7.844963000000	0.922539000000	0.252940000000
C	2.445140000000	1.898000000000	1.598145000000
C	3.136790000000	1.762512000000	0.404795000000
O	3.293877000000	1.715346000000	2.675944000000
C	1.019276000000	2.133645000000	1.978893000000
H	1.077122000000	2.734204000000	2.912773000000
C	0.344826000000	0.830992000000	2.438199000000
C	0.815761000000	-0.431275000000	2.041193000000
C	-0.721030000000	0.885870000000	3.363354000000
H	1.662961000000	-0.496086000000	1.345192000000
H	-1.090309000000	1.861077000000	3.712542000000
C	0.233052000000	-1.607549000000	2.538162000000
C	-1.318875000000	-0.285620000000	3.844452000000
H	0.633382000000	-2.582231000000	2.222797000000
H	-2.154659000000	-0.214654000000	4.557251000000
C	-0.843564000000	-1.541916000000	3.433334000000
H	-1.299137000000	-2.464592000000	3.825042000000
N	2.650413000000	2.031283000000	-0.885628000000
S	2.564447000000	0.605024000000	-2.000174000000
O	3.874114000000	-0.082053000000	-1.910543000000
O	2.048189000000	1.148574000000	-3.274144000000
C	1.302711000000	-0.525543000000	-1.418640000000
C	-0.049764000000	-0.155671000000	-1.522406000000
C	1.680750000000	-1.795002000000	-0.958456000000
H	-0.326092000000	0.825611000000	-1.934727000000
H	2.748346000000	-2.055387000000	-0.921151000000
C	-1.027583000000	-1.069497000000	-1.112731000000
C	0.682538000000	-2.702298000000	-0.582576000000
H	-2.090297000000	-0.787553000000	-1.178953000000
H	0.968402000000	-3.708246000000	-0.236412000000
C	-0.682858000000	-2.350735000000	-0.632119000000
C	-1.744654000000	-3.296052000000	-0.138020000000
H	-1.983814000000	-3.072066000000	0.924602000000
H	-2.685907000000	-3.196056000000	-0.712950000000
H	-1.412971000000	-4.351318000000	-0.188238000000
P	2.457937000000	5.825261000000	-1.634283000000
C	1.219243000000	6.459378000000	-0.450261000000
C	1.631362000000	7.114832000000	0.730604000000
C	-0.156089000000	6.363583000000	-0.752644000000
H	2.701537000000	7.205597000000	0.965185000000
H	-0.494199000000	5.833201000000	-1.651616000000
C	0.675569000000	7.645428000000	1.609196000000
C	-1.104101000000	6.891722000000	0.134307000000
H	1.004641000000	8.148225000000	2.530957000000

H	-2.174098000000	6.776657000000	-0.091015000000
C	-0.692241000000	7.530790000000	1.313851000000
H	-1.442515000000	7.933745000000	2.011128000000
C	4.026103000000	5.712531000000	-0.732974000000
C	4.060268000000	4.922591000000	0.439764000000
C	5.213795000000	6.314061000000	-1.202533000000
H	3.139018000000	4.460860000000	0.820372000000
H	5.195593000000	6.950331000000	-2.097739000000
C	5.275108000000	4.712285000000	1.107458000000
C	6.420840000000	6.102256000000	-0.524231000000
H	5.299141000000	4.080879000000	2.006959000000
H	7.341966000000	6.573507000000	-0.898808000000
C	6.456910000000	5.293118000000	0.623826000000
H	7.407941000000	5.117169000000	1.148258000000
C	2.618525000000	7.081862000000	-2.945130000000
C	3.544122000000	6.885400000000	-4.000191000000
C	1.752858000000	8.198890000000	-2.983546000000
H	4.213993000000	6.014415000000	-4.001735000000
H	1.029407000000	8.361386000000	-2.171651000000
C	3.605727000000	7.803152000000	-5.057095000000
C	1.820033000000	9.105794000000	-4.050084000000
H	4.330496000000	7.640033000000	-5.868868000000
H	1.143867000000	9.973996000000	-4.065980000000
C	2.745221000000	8.912518000000	-5.088328000000
H	2.793619000000	9.626042000000	-5.924686000000

4a'$_{SR}$

Electronic energy = -2219.042882 Hartree

C	0.222284000000	2.304604000000	-0.631733000000
O	-0.606396000000	1.470797000000	-0.968647000000
C	1.495814000000	2.523527000000	-1.429247000000
C	0.192969000000	3.069715000000	0.683836000000
H	0.742867000000	4.265321000000	-2.259659000000
H	0.865580000000	3.941360000000	0.557913000000
C	-1.153036000000	3.567499000000	1.160535000000
C	-1.228998000000	4.849426000000	1.742946000000
C	-2.326983000000	2.788998000000	1.079067000000
H	-0.318708000000	5.467768000000	1.808737000000
H	-2.277952000000	1.791491000000	0.624409000000
C	-2.443560000000	5.345760000000	2.238428000000
C	-3.542984000000	3.290914000000	1.565438000000
H	-2.481571000000	6.348588000000	2.690652000000
H	-4.450656000000	2.672571000000	1.492138000000
C	-3.606847000000	4.565090000000	2.150282000000
H	-4.562757000000	4.951452000000	2.535719000000
C	1.619539000000	3.592461000000	-2.258780000000
C	2.701379000000	4.029433000000	-3.140810000000
C	2.545302000000	5.286266000000	-3.779355000000
C	3.891741000000	3.299599000000	-3.390370000000
H	1.622479000000	5.861508000000	-3.603201000000
H	4.043932000000	2.315270000000	-2.934356000000
C	3.536257000000	5.805071000000	-4.620007000000
C	4.880769000000	3.822855000000	-4.230186000000
H	3.388929000000	6.783165000000	-5.102534000000
H	5.795244000000	3.237880000000	-4.411315000000
C	4.712517000000	5.073758000000	-4.847953000000
H	5.494123000000	5.473782000000	-5.511665000000
C	6.526191000000	0.023893000000	0.610828000000
C	5.428639000000	0.421211000000	-0.162880000000
C	6.479423000000	0.023210000000	2.024231000000
H	5.468071000000	0.402179000000	-1.258332000000
H	7.360750000000	-0.300761000000	2.597782000000
C	4.249645000000	0.825891000000	0.502718000000
C	5.328373000000	0.438638000000	2.707031000000
H	5.267454000000	0.463088000000	3.803897000000
C	4.245751000000	0.836524000000	1.918401000000
H	7.449509000000	-0.294991000000	0.104441000000
C	2.255370000000	1.601164000000	1.303143000000
C	2.928105000000	1.310867000000	0.114030000000
O	3.059953000000	1.318685000000	2.388963000000
C	0.922117000000	2.147453000000	1.746154000000
H	1.159484000000	2.834617000000	2.585304000000
C	0.018441000000	1.064883000000	2.321918000000
C	-0.097829000000	-0.204911000000	1.727481000000
C	-0.758986000000	1.357819000000	3.460277000000
H	0.513399000000	-0.456286000000	0.849943000000
H	-0.675962000000	2.350282000000	3.930083000000
C	-0.989004000000	-1.153134000000	2.252111000000

C	-1.644590000000	0.409264000000	3.988447000000
H	-1.074262000000	-2.137647000000	1.770683000000
H	-2.245185000000	0.657116000000	4.876684000000
C	-1.766030000000	-0.850013000000	3.380564000000
H	-2.462659000000	-1.597559000000	3.789815000000
N	2.495973000000	1.538051000000	-1.215142000000
S	2.466020000000	0.179247000000	-2.385205000000
O	3.877385000000	-0.197403000000	-2.614850000000
O	1.613253000000	0.657155000000	-3.485899000000
C	1.672183000000	-1.181565000000	-1.546235000000
C	0.303518000000	-1.399174000000	-1.760745000000
C	2.433245000000	-2.012821000000	-0.706651000000
H	-0.262568000000	-0.712306000000	-2.403660000000
H	3.507471000000	-1.824190000000	-0.568945000000
C	-0.306979000000	-2.486776000000	-1.122868000000
C	1.795802000000	-3.081553000000	-0.068238000000
H	-1.380272000000	-2.670822000000	-1.284776000000
H	2.379421000000	-3.735476000000	0.598339000000
C	0.422278000000	-3.342556000000	-0.271205000000
C	-0.237451000000	-4.514268000000	0.408766000000
H	-1.336006000000	-4.501569000000	0.274335000000
H	0.140991000000	-5.475001000000	0.000185000000
H	-0.020387000000	-4.521956000000	1.496567000000

DBU

Electronic energy = -462.0014428 Hartree

C	0.367060000000	-0.727941000000	-0.120617000000
C	-0.848444000000	1.476633000000	0.275366000000
H	-1.082551000000	1.473583000000	1.370802000000
H	-0.553767000000	2.521021000000	0.040783000000
C	-2.113064000000	1.146820000000	-0.523228000000
H	-1.807996000000	0.915867000000	-1.565297000000
H	-2.730815000000	2.066713000000	-0.586396000000
C	-2.958299000000	0.008551000000	0.065189000000
H	-3.683459000000	-0.331961000000	-0.704955000000
H	-3.569247000000	0.392131000000	0.910556000000
C	-2.107303000000	-1.176907000000	0.542141000000
H	-2.748750000000	-2.077312000000	0.636418000000
H	-1.718119000000	-0.984478000000	1.565277000000
C	-0.925404000000	-1.487835000000	-0.389283000000
H	-1.229022000000	-1.338986000000	-1.449637000000
H	-0.620638000000	-2.546119000000	-0.301088000000
C	1.589916000000	1.348947000000	0.312655000000
H	1.525866000000	2.386140000000	-0.082305000000
H	1.713612000000	1.442389000000	1.420668000000
C	2.783100000000	0.616005000000	-0.286485000000
H	3.728122000000	1.106975000000	0.024433000000
H	2.727915000000	0.676007000000	-1.394410000000
C	2.729040000000	-0.848367000000	0.150293000000
H	3.002649000000	-0.933309000000	1.229391000000
H	3.497217000000	-1.443094000000	-0.391783000000
N	0.337143000000	0.666872000000	-0.011327000000
N	1.434039000000	-1.464352000000	-0.060384000000

PPh_3

Electronic energy = -1036.159737 Hartree

P	-3.064828000000	-0.096487000000	-1.638598000000
C	-4.386162000000	-0.924805000000	-0.639120000000
C	-4.910301000000	-2.133497000000	-1.148176000000
C	-4.896305000000	-0.417675000000	0.573462000000
H	-4.529055000000	-2.529758000000	-2.103349000000
H	-4.500281000000	0.524947000000	0.980460000000
C	-5.906185000000	-2.830609000000	-0.449818000000
C	-5.903784000000	-1.110205000000	1.264294000000
H	-6.300724000000	-3.774642000000	-0.856155000000
H	-6.294572000000	-0.702586000000	2.209516000000
C	-6.408148000000	-2.318308000000	0.758256000000
H	-7.196904000000	-2.859578000000	1.302726000000
C	-1.549501000000	-0.970635000000	-1.029381000000
C	-1.532255000000	-1.841148000000	0.079652000000
C	-0.350499000000	-0.747770000000	-1.741565000000
H	-2.460370000000	-2.026773000000	0.640950000000
H	-0.357726000000	-0.081990000000	-2.619846000000
C	-0.338453000000	-2.468108000000	0.471101000000
C	0.843487000000	-1.363860000000	-1.340731000000
H	-0.338587000000	-3.147020000000	1.337908000000
H	1.771563000000	-1.176057000000	-1.902309000000
C	0.851550000000	-2.228825000000	-0.233747000000

H	1.786156000000	-2.720869000000	0.076041000000
C	-2.920055000000	1.533167000000	-0.770326000000
C	-3.827135000000	2.546074000000	-1.152200000000
C	-1.960491000000	1.816615000000	0.223154000000
H	-4.570973000000	2.338619000000	-1.938526000000
H	-1.245142000000	1.037924000000	0.527683000000
C	-3.790255000000	3.806296000000	-0.538904000000
C	-1.915880000000	3.083874000000	0.826140000000
H	-4.507863000000	4.584010000000	-0.842503000000
H	-1.160543000000	3.292368000000	1.599609000000
C	-2.831321000000	4.079464000000	0.450802000000
H	-2.795312000000	5.071639000000	0.926035000000

RC$_{D.d\text{-}SS}$

Electronic energy = -2681.053464 Hartree

C	-0.592337000000	-1.947143000000	-0.444594000000
O	-1.726794000000	-1.911284000000	0.027164000000
C	0.218447000000	-3.146246000000	-0.394637000000
C	0.003173000000	-0.723826000000	-1.165477000000
H	0.327365000000	-1.171682000000	2.407153000000
H	-0.262566000000	0.135693000000	-0.521059000000
C	-0.697216000000	-0.539094000000	-2.503277000000
C	-0.824651000000	-1.606696000000	-3.413967000000
C	-1.165576000000	0.733442000000	-2.879278000000
H	-0.458008000000	-2.608634000000	-3.138561000000
H	-1.050068000000	1.572142000000	-2.177684000000
C	-1.413200000000	-1.406330000000	-4.671820000000
C	-1.748551000000	0.936912000000	-4.138348000000
H	-1.510190000000	-2.251339000000	-5.370634000000
H	-2.098215000000	1.942107000000	-4.420635000000
C	-1.875688000000	-0.132467000000	-5.038860000000
H	-2.333651000000	0.026213000000	-6.027071000000
C	0.961510000000	-4.128768000000	-0.350964000000
C	1.885283000000	-5.205222000000	-0.234651000000
C	3.277897000000	-4.926913000000	-0.199931000000
C	1.442523000000	-6.545821000000	-0.099576000000
H	3.616568000000	-3.886573000000	-0.308562000000
H	0.364266000000	-6.758864000000	-0.130949000000
C	4.194725000000	-5.966272000000	-0.012341000000
C	2.371674000000	-7.577552000000	0.076316000000
H	5.270616000000	-5.739173000000	0.027273000000
H	2.020423000000	-8.615043000000	0.181377000000
C	3.746673000000	-7.291289000000	0.128030000000
H	4.472341000000	-8.105354000000	0.275387000000
C	5.001657000000	-1.169724000000	3.638070000000
C	3.690491000000	-0.795127000000	3.315388000000
C	5.902401000000	-1.625282000000	2.645351000000
H	2.985928000000	-0.424626000000	4.074746000000
H	6.926599000000	-1.906034000000	2.933469000000
C	3.288992000000	-0.893819000000	1.969631000000
C	5.522055000000	-1.714052000000	1.295755000000
H	6.215462000000	-2.052507000000	0.512655000000
C	4.211924000000	-1.333967000000	0.995948000000
H	5.345213000000	-1.099389000000	4.681107000000
C	2.309126000000	-0.931718000000	-0.076532000000
C	2.047821000000	-0.654533000000	1.253784000000
O	3.616789000000	-1.352028000000	-0.243246000000
C	1.550013000000	-0.753531000000	-1.353858000000
H	1.792338000000	-1.622898000000	-2.002401000000
C	2.024896000000	0.508352000000	-2.079072000000
C	2.303298000000	1.687273000000	-1.361385000000
C	2.127971000000	0.525289000000	-3.482956000000
H	2.254653000000	1.681339000000	-0.261695000000
H	1.909420000000	-0.390574000000	-4.053511000000
C	2.659464000000	2.863899000000	-2.037453000000
C	2.484870000000	1.700781000000	-4.159176000000
H	2.875822000000	3.776497000000	-1.460701000000
H	2.556551000000	1.697605000000	-5.257571000000
C	2.747330000000	2.876546000000	-3.438285000000
H	3.028408000000	3.799617000000	-3.968042000000
N	0.799974000000	-0.346885000000	1.822148000000
S	0.542113000000	1.172590000000	2.501855000000
O	1.803597000000	1.954125000000	2.459055000000
O	-0.198598000000	0.952524000000	3.764394000000
C	-0.602715000000	1.966704000000	1.356114000000
C	-0.181476000000	3.075433000000	0.612242000000
C	-1.908530000000	1.464392000000	1.221341000000
H	0.836261000000	3.462891000000	0.758555000000
H	-2.232740000000	0.610488000000	1.831172000000
C	-1.069430000000	3.666068000000	-0.300499000000
C	-2.778351000000	2.059752000000	0.303986000000
H	-0.735169000000	4.531166000000	-0.894497000000
H	-3.794144000000	1.652363000000	0.182865000000
C	-2.373865000000	3.164971000000	-0.479079000000

C	0.039348000000	-3.554407000000	3.364000000000
C	-0.232160000000	-5.884231000000	4.326450000000
H	-0.009570000000	-5.770783000000	5.415836000000
H	-1.087435000000	-6.587828000000	4.270469000000
C	0.961150000000	-6.518692000000	3.606047000000
H	0.819356000000	-6.374516000000	2.515364000000
H	0.921389000000	-7.615567000000	3.770663000000
C	2.330240000000	-5.982073000000	4.047351000000
H	3.080777000000	-6.240813000000	3.269568000000
H	2.658044000000	-6.493561000000	4.977761000000
C	2.314271000000	-4.465424000000	4.269846000000
H	3.352816000000	-4.075944000000	4.292529000000
H	1.885267000000	-4.223102000000	5.265917000000
C	1.537781000000	-3.711952000000	3.183311000000
H	1.728735000000	-4.177608000000	2.193797000000
H	1.923995000000	-2.684065000000	3.098568000000
C	-2.175882000000	-4.419685000000	3.951400000000
H	-2.681277000000	-5.391246000000	3.767083000000
H	-2.390970000000	-4.145279000000	5.012318000000
C	-2.706570000000	-3.349268000000	3.008170000000
H	-3.775173000000	-3.149624000000	3.224870000000
H	-2.635826000000	-3.697340000000	1.956680000000
C	-1.862374000000	-2.090407000000	3.156734000000
H	-2.070640000000	-1.562373000000	4.114943000000
H	-2.110534000000	-1.378744000000	2.343025000000
N	-0.736124000000	-4.625672000000	3.764624000000
N	-0.440398000000	-2.373539000000	3.074161000000
C	-3.310168000000	3.756328000000	-1.500735000000
H	-2.905042000000	4.688173000000	-1.939933000000
H	-3.485082000000	3.037219000000	-2.329647000000
H	-4.302231000000	3.983985000000	-1.060059000000

TS1$_{D.d\text{-}SS}$

Electronic energy = -2681.051145 Hartree

C	-0.910606000000	-1.986920000000	-0.587170000000
O	-2.033278000000	-2.010051000000	-0.088064000000
C	-0.071401000000	-3.166820000000	-0.641899000000
C	-0.359527000000	-0.707664000000	-1.240572000000
H	0.061269000000	-1.489654000000	2.256171000000
H	-0.605877000000	0.105482000000	-0.530709000000
C	-1.107692000000	-0.460526000000	-2.544398000000
C	-1.209558000000	-1.470542000000	-3.521779000000
C	-1.642688000000	0.809951000000	-2.824326000000
H	-0.793793000000	-2.470839000000	-3.320420000000
H	-1.548748000000	1.606862000000	-2.072141000000
C	-1.833460000000	-1.215147000000	-4.751670000000
C	-2.261052000000	1.069321000000	-4.055920000000
H	-1.908231000000	-2.015924000000	-5.503331000000
H	-2.665606000000	2.072143000000	-4.262255000000
C	-2.358793000000	0.057898000000	-5.024047000000
H	-2.843966000000	0.261036000000	-5.990891000000
C	0.705740000000	-4.122789000000	-0.684742000000
C	1.680707000000	-5.159491000000	-0.661600000000
C	3.058555000000	-4.813228000000	-0.646872000000
C	1.308031000000	-6.526076000000	-0.592597000000
H	3.343254000000	-3.752763000000	-0.705082000000
H	0.241081000000	-6.791528000000	-0.610009000000
C	4.030055000000	-5.813569000000	-0.539099000000
C	2.291385000000	-7.517584000000	-0.497960000000
H	5.093809000000	-5.533613000000	-0.511452000000
H	1.994936000000	-8.575906000000	-0.444053000000
C	3.651629000000	-7.165292000000	-0.462449000000
H	4.420084000000	-7.948424000000	-0.377594000000
C	4.970051000000	-1.404079000000	3.219881000000
C	3.640372000000	-1.004221000000	3.031939000000
C	5.801110000000	-1.747582000000	2.127026000000
H	3.000493000000	-0.698277000000	3.870673000000
H	6.843649000000	-2.047827000000	2.311503000000
C	3.136191000000	-0.961780000000	1.714799000000
C	5.321948000000	-1.701256000000	0.808489000000
H	5.951965000000	-1.956368000000	-0.055706000000
C	3.991642000000	-1.305816000000	0.644067000000
H	5.383722000000	-1.438574000000	4.239200000000
C	2.011939000000	-0.859832000000	-0.258921000000
C	1.833891000000	-0.685974000000	1.108660000000
O	3.314207000000	-1.241211000000	-0.545063000000
C	1.173137000000	-0.712206000000	-1.494320000000
H	1.397280000000	-1.596658000000	-2.129567000000
C	1.582325000000	0.521126000000	-2.298828000000
C	1.631099000000	1.786827000000	-1.688194000000
C	1.840050000000	0.420905000000	-3.677940000000
H	1.431687000000	1.878793000000	-0.610857000000
H	1.793006000000	-0.565100000000	-4.166057000000
C	1.924271000000	2.931455000000	-2.442949000000

C	2.132415000000	1.564388000000	-4.436088000000
H	1.957870000000	3.912709000000	-1.945207000000
H	2.326726000000	1.469175000000	-5.515394000000
C	2.173952000000	2.825260000000	-3.820742000000
H	2.404379000000	3.723432000000	-4.414018000000
N	0.604756000000	-0.494851000000	1.764409000000
S	0.378659000000	0.807693000000	2.783717000000
O	1.624295000000	1.220039000000	3.483759000000
O	-0.840366000000	0.484878000000	3.563372000000
C	-0.039600000000	2.170747000000	1.679343000000
C	0.862892000000	3.230423000000	1.517878000000
C	-1.265950000000	2.155693000000	0.995900000000
H	1.801563000000	3.219494000000	2.090353000000
H	-1.976236000000	1.332582000000	1.164075000000
C	0.544202000000	4.270980000000	0.632876000000
C	-1.565801000000	3.200720000000	0.113491000000
H	1.251739000000	5.105271000000	0.501664000000
H	-2.527473000000	3.195140000000	-0.424052000000
C	-0.660692000000	4.264624000000	-0.098920000000
C	-0.085918000000	-3.715005000000	3.009059000000
C	-0.254409000000	-6.072393000000	3.905817000000
H	-0.004077000000	-5.983503000000	4.990486000000
H	-1.089124000000	-6.799600000000	3.850929000000
C	0.936446000000	-6.642974000000	3.130075000000
H	0.757920000000	-6.469685000000	2.049021000000
H	0.936741000000	-7.745072000000	3.260433000000
C	2.299761000000	-6.074609000000	3.548702000000
H	3.034217000000	-6.283551000000	2.741436000000
H	2.671755000000	-6.603196000000	4.452413000000
C	2.242059000000	-4.567261000000	3.821033000000
H	3.266715000000	-4.142098000000	3.822355000000
H	1.838384000000	-4.368698000000	4.837133000000
C	1.410142000000	-3.804925000000	2.783011000000
H	1.592602000000	-4.224382000000	1.772220000000
H	1.758745000000	-2.761613000000	2.729996000000
C	-2.264308000000	-4.674826000000	3.601453000000
H	-2.727463000000	-5.653347000000	3.354119000000
H	-2.480671000000	-4.482503000000	4.679042000000
C	-2.848196000000	-3.568374000000	2.734020000000
H	-3.916752000000	-3.419569000000	2.986886000000
H	-2.791329000000	-3.846063000000	1.661315000000
C	-2.046150000000	-2.292823000000	2.943632000000
H	-2.211802000000	-1.853701000000	3.951776000000
H	-2.339790000000	-1.513067000000	2.215251000000
N	-0.814802000000	-4.811023000000	3.402531000000
N	-0.624377000000	-2.543781000000	2.770502000000
C	-0.957673000000	5.335639000000	-1.115585000000
H	-0.444440000000	6.286865000000	-0.873653000000
H	-0.605166000000	5.017126000000	-2.121263000000
H	-2.044187000000	5.535597000000	-1.198308000000

I$_{D.d\text{-}SS}$

Electronic energy = -2681.060813 Hartree

C	-1.005086000000	-1.792247000000	-0.575505000000
O	-2.173395000000	-1.708073000000	-0.195384000000
C	-0.229278000000	-3.011082000000	-0.447418000000
C	-0.336431000000	-0.640501000000	-1.340467000000
H	-0.249109000000	-2.268375000000	2.295359000000
H	-0.586005000000	0.267372000000	-0.759172000000
C	-0.991628000000	-0.514773000000	-2.710387000000
C	-1.057637000000	-1.618260000000	-3.584014000000
C	-1.480518000000	0.728724000000	-3.150959000000
H	-0.678111000000	-2.599078000000	-3.255115000000
H	-1.414667000000	1.595743000000	-2.477210000000
C	-1.601238000000	-1.480719000000	-4.869733000000
C	-2.019293000000	0.870070000000	-4.437943000000
H	-1.649282000000	-2.353034000000	-5.539652000000
H	-2.388516000000	1.852633000000	-4.769795000000
C	-2.081488000000	-0.234212000000	-5.302004000000
H	-2.503984000000	-0.124199000000	-6.312436000000
C	0.527619000000	-3.975809000000	-0.331081000000
C	1.496909000000	-4.997131000000	-0.127609000000
C	2.874338000000	-4.645881000000	-0.106399000000
C	1.123279000000	-6.344227000000	0.110339000000
H	3.164179000000	-3.601285000000	-0.293293000000
H	0.057449000000	-6.614681000000	0.090768000000
C	3.841472000000	-5.618682000000	0.168841000000
C	2.102633000000	-7.310442000000	0.370724000000
H	4.903411000000	-5.331901000000	0.197075000000
H	1.804672000000	-8.354365000000	0.551387000000
C	3.461225000000	-6.950917000000	0.408618000000
H	4.226323000000	-7.712804000000	0.621091000000
C	4.728073000000	-0.916841000000	3.470312000000
C	3.360802000000	-0.786582000000	3.189558000000

C	5.683915000000	-1.066390000000	2.437464000000
H	2.616168000000	-0.614699000000	3.979684000000
H	6.750670000000	-1.157296000000	2.692557000000
C	2.946432000000	-0.819242000000	1.840536000000
C	5.295706000000	-1.079755000000	1.088588000000
H	6.021580000000	-1.178040000000	0.268699000000
C	3.928149000000	-0.949652000000	0.829341000000
H	5.069590000000	-0.882926000000	4.516403000000
C	1.972378000000	-0.824214000000	-0.210549000000
C	1.657649000000	-0.776015000000	1.143156000000
O	3.343450000000	-0.955087000000	-0.406650000000
C	1.208206000000	-0.726136000000	-1.497145000000
H	1.435274000000	-1.637900000000	-2.094645000000
C	1.681896000000	0.479374000000	-2.310727000000
C	1.707399000000	1.756139000000	-1.719549000000
C	2.004969000000	0.353981000000	-3.672841000000
H	1.457132000000	1.864820000000	-0.653381000000
H	1.974069000000	-0.640050000000	-4.145874000000
C	2.035504000000	2.887583000000	-2.479081000000
C	2.337800000000	1.484254000000	-4.435247000000
H	2.044995000000	3.879058000000	-1.999747000000
H	2.583218000000	1.370127000000	-5.502288000000
C	2.350404000000	2.756088000000	-3.841910000000
H	2.608254000000	3.643424000000	-4.440227000000
N	0.369837000000	-0.892709000000	1.674032000000
S	-0.214394000000	0.251536000000	2.706320000000
O	0.796048000000	0.747241000000	3.686605000000
O	-1.498313000000	-0.296594000000	3.220691000000
C	-0.617542000000	1.664352000000	1.659920000000
C	0.265879000000	2.751780000000	1.592424000000
C	-1.774057000000	1.624649000000	0.864652000000
H	1.148155000000	2.761282000000	2.248773000000
H	-2.452680000000	0.761362000000	0.932352000000
C	-0.000042000000	3.795582000000	0.694793000000
C	-2.024326000000	2.678245000000	-0.025198000000
H	0.695531000000	4.648329000000	0.635543000000
H	-2.929148000000	2.648023000000	-0.653086000000
C	-1.135182000000	3.769004000000	-0.142138000000
C	0.062012000000	-4.042181000000	3.317305000000
C	0.326239000000	-6.162330000000	4.653515000000
H	0.398327000000	-5.869137000000	5.727315000000
H	-0.299529000000	-7.076832000000	4.630673000000
C	1.709382000000	-6.501142000000	4.094094000000
H	1.636434000000	-6.544616000000	2.987698000000
H	1.959722000000	-7.532914000000	4.416624000000
C	2.822732000000	-5.538976000000	4.529431000000
H	3.691692000000	-5.672497000000	3.850737000000
H	3.179938000000	-5.803044000000	5.547514000000
C	2.368521000000	-4.075684000000	4.507079000000
H	3.250150000000	-3.403284000000	4.523853000000
H	1.789486000000	-3.828587000000	5.422664000000
C	1.538613000000	-3.728824000000	3.268716000000
H	1.971668000000	-4.208894000000	2.366020000000
H	1.597794000000	-2.644697000000	3.067405000000
C	-1.910397000000	-5.297179000000	4.004785000000
H	-2.133823000000	-6.379380000000	4.090838000000
H	-2.272127000000	-4.814555000000	4.941441000000
C	-2.604151000000	-4.692439000000	2.788724000000
H	-3.702732000000	-4.757311000000	2.911116000000
H	-2.336378000000	-5.279092000000	1.884186000000
C	-2.167523000000	-3.246786000000	2.617656000000
H	-2.614075000000	-2.569461000000	3.375477000000
H	-2.449734000000	-2.837672000000	1.625684000000
N	-0.448408000000	-5.154697000000	3.909550000000
N	-0.718717000000	-3.155770000000	2.730398000000
C	-1.362002000000	4.846306000000	-1.171173000000
H	-0.987571000000	5.830637000000	-0.826615000000
H	-0.818960000000	4.601585000000	-2.110838000000
H	-2.434254000000	4.951417000000	-1.428509000000

TS2$_{D.d-SS}$

Electronic energy = -2681.030487 Hartree

C	-0.302257000000	-2.458426000000	-2.106600000000
O	0.009102000000	-3.113378000000	-3.142318000000
C	-0.437436000000	-3.039481000000	-0.818570000000
C	-0.521833000000	-0.948349000000	-2.151633000000
H	0.030069000000	-4.686084000000	-2.778972000000
H	-0.757590000000	-0.610695000000	-1.124132000000
C	-1.657699000000	-0.509172000000	-3.053166000000
C	-1.793615000000	-0.967795000000	-4.379026000000
C	-2.579906000000	0.433432000000	-2.556056000000
H	-1.079626000000	-1.710496000000	-4.764301000000
H	-2.467092000000	0.789352000000	-1.519969000000

C	-2.832199000000	-0.488941000000	-5.191730000000
C	-3.612671000000	0.916648000000	-3.372591000000
H	-2.929049000000	-0.854026000000	-6.226237000000
H	-4.320417000000	1.660125000000	-2.974626000000
C	-3.742159000000	0.457227000000	-4.692909000000
H	-4.552097000000	0.837546000000	-5.334444000000
C	-0.259345000000	-3.123642000000	0.418560000000
C	-0.374120000000	-3.679019000000	1.733520000000
C	0.721135000000	-3.702610000000	2.631163000000
C	-1.603162000000	-4.264253000000	2.136090000000
H	1.640718000000	-3.183560000000	2.328485000000
H	-2.465047000000	-4.214603000000	1.453911000000
C	0.593789000000	-4.316459000000	3.882596000000
C	-1.721133000000	-4.868210000000	3.394618000000
H	1.448015000000	-4.311136000000	4.576057000000
H	-2.684627000000	-5.304400000000	3.700147000000
C	-0.622047000000	-4.905471000000	4.268889000000
H	-0.719500000000	-5.375871000000	5.259143000000
C	5.682949000000	-2.101905000000	0.731181000000
C	4.329992000000	-1.819116000000	0.968543000000
C	6.240581000000	-1.971224000000	-0.563831000000
H	3.883078000000	-1.908568000000	1.970534000000
H	7.306854000000	-2.195916000000	-0.718331000000
C	3.535954000000	-1.412293000000	-0.118199000000
C	5.464355000000	-1.546149000000	-1.655959000000
H	5.885758000000	-1.430616000000	-2.664661000000
C	4.117993000000	-1.272373000000	-1.394637000000
H	6.329109000000	-2.421823000000	1.562951000000
C	1.953720000000	-0.780568000000	-1.622646000000
C	2.118957000000	-1.089823000000	-0.258508000000
O	3.173186000000	-0.879158000000	-2.294719000000
C	0.858828000000	-0.346118000000	-2.563308000000
H	1.113803000000	-0.828520000000	-3.531084000000
C	0.794845000000	1.154229000000	-2.816264000000
C	0.841209000000	2.085595000000	-1.761292000000
C	0.624810000000	1.622192000000	-4.134553000000
H	0.968686000000	1.752368000000	-0.719524000000
H	0.582246000000	0.896796000000	-4.961991000000
C	0.715761000000	3.457703000000	-2.028113000000
C	0.498877000000	2.993102000000	-4.399111000000
H	0.751968000000	4.172002000000	-1.191216000000
H	0.365235000000	3.340753000000	-5.435223000000

C	0.543436000000	3.917208000000	-3.343211000000
H	0.444381000000	4.994825000000	-3.546622000000
N	1.280194000000	-1.252080000000	0.824051000000
S	0.322900000000	-0.049700000000	1.381625000000
O	-1.077107000000	-0.051070000000	0.841140000000
O	1.041976000000	1.260028000000	1.379006000000
C	0.173640000000	-0.567623000000	3.102857000000
C	-1.061442000000	-1.007843000000	3.593495000000
C	1.304911000000	-0.523976000000	3.928935000000
H	-1.926026000000	-1.013507000000	2.914696000000
H	2.255511000000	-0.148331000000	3.522305000000
C	-1.152759000000	-1.445189000000	4.921101000000
C	1.198116000000	-0.963450000000	5.254694000000
H	-2.118979000000	-1.808321000000	5.306277000000
H	2.083771000000	-0.936369000000	5.909767000000
C	-0.026113000000	-1.442044000000	5.769275000000
C	0.882557000000	-5.856093000000	-1.348100000000
C	1.663976000000	-6.922024000000	0.793415000000
H	1.564034000000	-6.137083000000	1.577799000000
H	1.319199000000	-7.867617000000	1.255728000000
C	3.118231000000	-7.086123000000	0.354683000000
H	3.135809000000	-7.682630000000	-0.582643000000
H	3.625418000000	-7.716582000000	1.113749000000
C	3.878351000000	-5.765799000000	0.184180000000
H	4.812536000000	-5.958830000000	-0.384550000000
H	4.200235000000	-5.378396000000	1.173255000000
C	3.046618000000	-4.691451000000	-0.519614000000
H	3.713811000000	-3.896366000000	-0.899630000000
H	2.371924000000	-4.176032000000	0.193462000000
C	2.211753000000	-5.219308000000	-1.689017000000
H	2.792610000000	-5.950977000000	-2.294022000000
H	1.971579000000	-4.387258000000	-2.380012000000
C	-0.685739000000	-7.102945000000	0.039406000000
H	-0.594575000000	-8.052290000000	0.602676000000
H	-1.157679000000	-6.361666000000	0.722339000000
C	-1.515143000000	-7.304393000000	-1.222037000000
H	-2.557935000000	-7.556125000000	-0.948037000000
H	-1.110237000000	-8.159251000000	-1.803256000000
C	-1.473082000000	-6.035790000000	-2.058693000000
H	-2.056594000000	-5.218510000000	-1.578059000000
H	-1.903674000000	-6.196751000000	-3.067532000000
N	0.680928000000	-6.647441000000	-0.276346000000

N	-0.095996000000	-5.592246000000	-2.214132000000
C	-0.131227000000	-1.962548000000	7.180276000000
H	-0.988287000000	-1.509519000000	7.719451000000
H	-0.296336000000	-3.061966000000	7.185113000000
H	0.787391000000	-1.758855000000	7.763879000000

II$_{D.d\text{-}SS}$

Electronic energy = -2681.042613 Hartree

C	-0.303203000000	-2.454841000000	-2.033727000000
O	0.148746000000	-3.109527000000	-3.049100000000
C	-0.458548000000	-2.966104000000	-0.753756000000
C	-0.634948000000	-0.968306000000	-2.215762000000
H	0.103891000000	-4.540367000000	-2.710131000000
H	-0.966733000000	-0.565920000000	-1.238861000000
C	-1.689493000000	-0.639118000000	-3.249623000000
C	-1.680348000000	-1.220157000000	-4.535542000000
C	-2.690221000000	0.303060000000	-2.939194000000
H	-0.907497000000	-1.966220000000	-4.770555000000
H	-2.696389000000	0.765611000000	-1.939667000000
C	-2.652613000000	-0.865179000000	-5.482473000000
C	-3.657403000000	0.663273000000	-3.888804000000
H	-2.636082000000	-1.329779000000	-6.480960000000
H	-4.426275000000	1.407635000000	-3.630123000000
C	-3.641696000000	0.080378000000	-5.165709000000
H	-4.398686000000	0.363155000000	-5.913502000000
C	0.055911000000	-2.652236000000	0.442889000000
C	-0.292300000000	-3.400778000000	1.680014000000
C	0.692334000000	-3.735177000000	2.638977000000
C	-1.613121000000	-3.857090000000	1.894561000000
H	1.715272000000	-3.357843000000	2.498310000000
H	-2.378656000000	-3.584604000000	1.151731000000
C	0.372763000000	-4.511623000000	3.761681000000
C	-1.936767000000	-4.618373000000	3.025770000000
H	1.157574000000	-4.758307000000	4.493820000000
H	-2.976268000000	-4.946507000000	3.183429000000
C	-0.944286000000	-4.957022000000	3.964124000000
H	-1.199633000000	-5.554492000000	4.852583000000
C	5.612524000000	-1.794456000000	0.813503000000
C	4.220393000000	-1.793121000000	0.974353000000
C	6.212917000000	-1.371237000000	-0.396465000000
H	3.750485000000	-2.122460000000	1.913164000000
H	7.309269000000	-1.382225000000	-0.488748000000
C	3.426608000000	-1.362807000000	-0.106130000000
C	5.437455000000	-0.929613000000	-1.480897000000
H	5.886568000000	-0.592369000000	-2.425526000000
C	4.052566000000	-0.935847000000	-1.297280000000
H	6.255388000000	-2.126774000000	1.642670000000
C	1.861267000000	-0.730113000000	-1.635235000000
C	2.002635000000	-1.240390000000	-0.344428000000
O	3.105177000000	-0.561092000000	-2.210339000000
C	0.749992000000	-0.324769000000	-2.569716000000
H	1.037325000000	-0.768971000000	-3.546372000000
C	0.650246000000	1.183031000000	-2.765066000000
C	0.714161000000	2.075770000000	-1.678102000000
C	0.421569000000	1.696097000000	-4.057286000000
H	0.901248000000	1.699920000000	-0.661811000000
H	0.362899000000	1.002614000000	-4.910164000000
C	0.548446000000	3.453874000000	-1.884634000000
C	0.252321000000	3.072710000000	-4.261533000000
H	0.604042000000	4.138601000000	-1.024382000000
H	0.070116000000	3.455493000000	-5.277523000000
C	0.314774000000	3.957632000000	-3.173670000000
H	0.183451000000	5.039281000000	-3.331560000000
N	1.051228000000	-1.589903000000	0.644475000000
S	0.409234000000	-0.189060000000	1.507476000000
O	-1.011028000000	0.023438000000	1.135397000000
O	1.420129000000	0.880899000000	1.336351000000
C	0.446856000000	-0.690216000000	3.224872000000
C	-0.746540000000	-1.065747000000	3.856340000000
C	1.675980000000	-0.720801000000	3.897263000000
H	-1.690178000000	-1.017719000000	3.296057000000
H	2.589131000000	-0.384955000000	3.384381000000
C	-0.694951000000	-1.517843000000	5.178572000000
C	1.706413000000	-1.180127000000	5.220210000000
H	-1.625113000000	-1.831105000000	5.677553000000
H	2.666117000000	-1.214108000000	5.759553000000
C	0.529309000000	-1.599446000000	5.876037000000
C	0.827628000000	-5.837019000000	-1.287412000000
C	1.458498000000	-7.015812000000	0.850314000000
H	1.376253000000	-6.277195000000	1.681178000000
H	1.050734000000	-7.968178000000	1.245356000000
C	2.916103000000	-7.236817000000	0.448652000000
H	2.933038000000	-7.782275000000	-0.518969000000
H	3.365966000000	-7.931078000000	1.188023000000

C	3.751462000000	-5.954014000000	0.367428000000
H	4.699325000000	-6.176233000000	-0.167663000000
H	4.045575000000	-5.626961000000	1.387182000000
C	3.006342000000	-4.812978000000	-0.330606000000
H	3.724348000000	-4.031449000000	-0.643685000000
H	2.318183000000	-4.304184000000	0.374091000000
C	2.202358000000	-5.258907000000	-1.553773000000
H	2.777405000000	-5.988905000000	-2.165602000000
H	2.015903000000	-4.391043000000	-2.217532000000
C	-0.880151000000	-6.930386000000	0.053588000000
H	-0.905012000000	-7.858503000000	0.657705000000
H	-1.302395000000	-6.114509000000	0.681152000000
C	-1.674763000000	-7.102137000000	-1.235187000000
H	-2.745897000000	-7.255258000000	-0.998200000000
H	-1.321792000000	-8.008242000000	-1.771585000000
C	-1.490359000000	-5.865850000000	-2.101568000000
H	-2.023114000000	-4.988082000000	-1.668634000000
H	-1.882023000000	-6.023222000000	-3.127272000000
N	0.533483000000	-6.624225000000	-0.227983000000
N	-0.083297000000	-5.511692000000	-2.196420000000
C	0.568922000000	-2.144155000000	7.279966000000
H	-0.187211000000	-1.654615000000	7.927011000000
H	0.337395000000	-3.230957000000	7.283549000000
H	1.562869000000	-2.009512000000	7.748195000000

TS3$_{D.d\text{-}SS}$

Electronic energy = -2681.023037 Hartree

C	-1.038735000000	-2.410453000000	-0.281142000000
O	-2.172457000000	-2.226414000000	0.171532000000
C	-0.203660000000	-3.576209000000	-0.086655000000
C	-0.416343000000	-1.334439000000	-1.209101000000
H	-1.519572000000	-2.273486000000	3.200532000000
H	-0.654258000000	-0.387452000000	-0.689457000000
C	-1.122274000000	-1.315131000000	-2.553411000000
C	-1.257311000000	-2.491710000000	-3.317921000000
C	-1.610754000000	-0.105220000000	-3.081397000000
H	-0.882061000000	-3.445009000000	-2.911150000000
H	-1.490102000000	0.819104000000	-2.495741000000
C	-1.872221000000	-2.459560000000	-4.577574000000
C	-2.224359000000	-0.069911000000	-4.342984000000
H	-1.975788000000	-3.388201000000	-5.160003000000
H	-2.597217000000	0.885804000000	-4.742614000000
C	-2.357951000000	-1.247128000000	-5.094884000000
H	-2.840154000000	-1.221198000000	-6.084090000000
C	1.070086000000	-3.724645000000	-0.008042000000
C	2.148694000000	-4.691536000000	-0.013955000000
C	3.240735000000	-4.616616000000	0.882374000000
C	2.096873000000	-5.774791000000	-0.928141000000
H	3.286994000000	-3.793097000000	1.606789000000
H	1.247104000000	-5.836449000000	-1.623571000000
C	4.248568000000	-5.589069000000	0.863942000000
C	3.103323000000	-6.747667000000	-0.936516000000
H	5.090703000000	-5.505752000000	1.567757000000
H	3.046072000000	-7.582919000000	-1.651786000000
C	4.185321000000	-6.658787000000	-0.043104000000
H	4.978305000000	-7.421886000000	-0.057050000000
C	5.014047000000	-1.020106000000	3.181244000000
C	3.628013000000	-0.926561000000	3.010318000000
C	5.865714000000	-1.392219000000	2.113110000000
H	2.959591000000	-0.617786000000	3.824892000000
H	6.952110000000	-1.449276000000	2.280666000000
C	3.086505000000	-1.212242000000	1.735593000000
C	5.351445000000	-1.690608000000	0.843087000000
H	5.991199000000	-1.996813000000	0.003826000000
C	3.966112000000	-1.587723000000	0.683276000000
H	5.452920000000	-0.795793000000	4.165157000000
C	1.921239000000	-1.755899000000	-0.176898000000
C	1.745360000000	-1.264563000000	1.159740000000
O	3.286602000000	-1.888882000000	-0.454942000000
C	1.122843000000	-1.391026000000	-1.417448000000
H	1.343350000000	-2.171202000000	-2.175851000000
C	1.592598000000	-0.055863000000	-1.991596000000
C	1.516029000000	1.123888000000	-1.224633000000
C	2.053803000000	0.029784000000	-3.317123000000
H	1.149450000000	1.081867000000	-0.188396000000
H	2.107317000000	-0.885001000000	-3.928122000000
C	1.889415000000	2.358950000000	-1.772081000000
C	2.429604000000	1.264867000000	-3.868767000000
H	1.816206000000	3.266851000000	-1.153646000000
H	2.785636000000	1.312554000000	-4.909433000000
C	2.347757000000	2.434565000000	-3.098012000000
H	2.641084000000	3.403979000000	-3.529396000000
N	0.501203000000	-1.066216000000	1.646374000000
S	0.033783000000	-0.068195000000	2.819300000000

O	1.075607000000	0.463218000000	3.742886000000
O	-1.159413000000	-0.714364000000	3.481613000000
C	-0.609121000000	1.325697000000	1.882329000000
C	0.072553000000	2.549588000000	1.927381000000
C	-1.694103000000	1.128667000000	1.010580000000
H	0.912139000000	2.667837000000	2.627540000000
H	-2.206665000000	0.154347000000	0.971529000000
C	-0.325886000000	3.583583000000	1.067369000000
C	-2.074879000000	2.173806000000	0.161057000000
H	0.209489000000	4.546179000000	1.095276000000
H	-2.917436000000	2.020759000000	-0.532161000000
C	-1.389177000000	3.409388000000	0.158349000000
C	-0.837526000000	-4.199108000000	3.309219000000
C	-0.162832000000	-6.608155000000	3.515102000000
H	-0.309059000000	-6.840406000000	4.595351000000
H	-0.525294000000	-7.494544000000	2.960378000000
C	1.315682000000	-6.396339000000	3.192026000000
H	1.382806000000	-5.906654000000	2.199975000000
H	1.786751000000	-7.391665000000	3.060313000000
C	2.084792000000	-5.596141000000	4.250126000000
H	3.022672000000	-5.218238000000	3.792328000000
H	2.394099000000	-6.260605000000	5.084858000000
C	1.273088000000	-4.422724000000	4.810818000000
H	1.951524000000	-3.712048000000	5.324189000000
H	0.566442000000	-4.772308000000	5.594054000000
C	0.501365000000	-3.644448000000	3.737744000000
H	1.112537000000	-3.524929000000	2.816858000000
H	0.299768000000	-2.614736000000	4.084843000000
C	-2.374998000000	-5.967876000000	2.594451000000
H	-2.175654000000	-6.843453000000	1.943670000000
H	-3.003669000000	-6.329555000000	3.440754000000
C	-3.081812000000	-4.874666000000	1.808133000000
H	-4.099250000000	-5.211049000000	1.530489000000
H	-2.520204000000	-4.651473000000	0.876837000000
C	-3.131483000000	-3.602520000000	2.636980000000
H	-3.813140000000	-3.706719000000	3.511271000000
H	-3.465709000000	-2.749670000000	2.020422000000
N	-1.072572000000	-5.516628000000	3.117817000000
N	-1.786933000000	-3.296714000000	3.105063000000
C	-1.750210000000	4.488867000000	-0.828823000000
H	-1.487385000000	5.496680000000	-0.451634000000
H	-1.196850000000	4.339731000000	-1.781944000000
H	-2.830647000000	4.478172000000	-1.073811000000

III$_{D.d\text{-}SS}$

Electronic energy = -2681.039581 Hartree

C	-1.080156000000	-2.349691000000	-0.341642000000
O	-2.178636000000	-2.062833000000	0.172677000000
C	-0.309963000000	-3.544140000000	-0.148543000000
C	-0.466413000000	-1.323419000000	-1.347978000000
H	-1.447947000000	-2.517073000000	3.049425000000
H	-0.742399000000	-0.349000000000	-0.905476000000
C	-1.128075000000	-1.406544000000	-2.707582000000
C	-1.153493000000	-2.618414000000	-3.429157000000
C	-1.720945000000	-0.266755000000	-3.283615000000
H	-0.710434000000	-3.520032000000	-2.974956000000
H	-1.698902000000	0.684290000000	-2.728286000000
C	-1.754256000000	-2.686072000000	-4.693258000000
C	-2.324754000000	-0.332239000000	-4.549384000000
H	-1.769691000000	-3.641618000000	-5.240191000000
H	-2.782094000000	0.569979000000	-4.984484000000
C	-2.342341000000	-1.541909000000	-5.259668000000
H	-2.815385000000	-1.595428000000	-6.252187000000
C	1.040127000000	-3.509978000000	-0.066788000000
C	1.879751000000	-4.730563000000	0.048395000000
C	3.112026000000	-4.773161000000	0.744696000000
C	1.403716000000	-5.939594000000	-0.517052000000
H	3.496203000000	-3.874822000000	1.245626000000
H	0.431504000000	-5.909681000000	-1.032302000000
C	3.849904000000	-5.963819000000	0.840270000000
C	2.139990000000	-7.125695000000	-0.422562000000
H	4.804809000000	-5.965014000000	1.388748000000
H	1.751803000000	-8.046374000000	-0.886127000000
C	3.375035000000	-7.145348000000	0.251832000000
H	3.957820000000	-8.076423000000	0.321882000000
C	5.121127000000	-0.256016000000	2.468701000000
C	3.726713000000	-0.346236000000	2.485454000000
C	5.876884000000	-0.833940000000	1.421058000000
H	3.125716000000	0.092185000000	3.292454000000
H	6.974146000000	-0.745266000000	1.433917000000
C	3.079815000000	-1.023867000000	1.424409000000
C	5.261120000000	-1.518823000000	0.365398000000
H	5.832256000000	-1.977043000000	-0.453505000000
C	3.862007000000	-1.595686000000	0.381543000000

H	5.640341000000	0.269864000000	3.283419000000
C	1.727658000000	-2.110842000000	-0.207024000000
C	1.698240000000	-1.301799000000	1.078283000000
O	3.132761000000	-2.230655000000	-0.553662000000
C	1.070589000000	-1.410054000000	-1.455480000000
H	1.323708000000	-2.102226000000	-2.284056000000
C	1.732157000000	-0.075026000000	-1.751716000000
C	1.413005000000	1.091576000000	-1.025742000000
C	2.727638000000	0.010376000000	-2.744597000000
H	0.638876000000	1.059958000000	-0.247378000000
H	2.989227000000	-0.893391000000	-3.316430000000
C	2.071122000000	2.302804000000	-1.281932000000
C	3.386771000000	1.220917000000	-3.006453000000
H	1.801221000000	3.195724000000	-0.697074000000
H	4.159447000000	1.264040000000	-3.789500000000
C	3.061495000000	2.373167000000	-2.274116000000
H	3.577713000000	3.323840000000	-2.477836000000
N	0.509970000000	-1.033388000000	1.565758000000
S	0.052633000000	-0.111201000000	2.857126000000
O	1.113995000000	0.452521000000	3.733074000000
O	-1.018907000000	-0.907300000000	3.542698000000
C	-0.728099000000	1.237577000000	1.973941000000
C	-0.188988000000	2.524892000000	2.104107000000
C	-1.796465000000	0.967628000000	1.100610000000
H	0.647982000000	2.691518000000	2.797177000000
H	-2.179886000000	-0.059778000000	0.971670000000
C	-0.727276000000	3.563669000000	1.331734000000
C	-2.312249000000	2.021678000000	0.337565000000
H	-0.310587000000	4.578935000000	1.425958000000
H	-3.136904000000	1.815572000000	-0.363239000000
C	-1.785143000000	3.329612000000	0.428688000000
C	-0.763772000000	-4.423534000000	3.177724000000
C	-0.125684000000	-6.830882000000	3.464789000000
H	-0.375037000000	-7.040329000000	4.530976000000
H	-0.447253000000	-7.722098000000	2.893141000000
C	1.377643000000	-6.646952000000	3.272586000000
H	1.542941000000	-6.201122000000	2.272613000000
H	1.839799000000	-7.653396000000	3.221017000000
C	2.069800000000	-5.818666000000	4.361931000000
H	3.046821000000	-5.472105000000	3.963941000000
H	2.300676000000	-6.454116000000	5.243404000000
C	1.237549000000	-4.613442000000	4.813770000000
H	1.883916000000	-3.892606000000	5.353779000000
H	0.466202000000	-4.923409000000	5.551242000000
C	0.558156000000	-3.871234000000	3.654354000000
H	1.226130000000	-3.828420000000	2.765933000000
H	0.361373000000	-2.821183000000	3.937745000000
C	-2.238747000000	-6.192247000000	2.338030000000
H	-1.970126000000	-7.039384000000	1.673744000000
H	-2.916830000000	-6.595447000000	3.125490000000
C	-2.901046000000	-5.083269000000	1.537827000000
H	-3.889841000000	-5.420690000000	1.172339000000
H	-2.256653000000	-4.823431000000	0.665299000000
C	-3.027842000000	-3.838178000000	2.399141000000
H	-3.749461000000	-3.977553000000	3.235747000000
H	-3.329218000000	-2.976426000000	1.777348000000
N	-0.985360000000	-5.738033000000	2.970408000000
N	-1.710948000000	-3.524206000000	2.941446000000
C	-2.313936000000	4.433706000000	-0.449454000000
H	-2.106855000000	5.434129000000	-0.022260000000
H	-1.834729000000	4.397189000000	-1.451766000000
H	-3.406279000000	4.340576000000	-0.609717000000

RC$_{D.d\text{-}SR}$

Electronic energy = -2681.04391 Hartree

C	0.419355000000	-0.400026000000	1.081959000000
O	0.789840000000	-1.124441000000	1.997197000000
C	0.246638000000	-0.875376000000	-0.278400000000
C	0.099700000000	1.095024000000	1.328887000000
H	-2.848913000000	2.450837000000	-1.762205000000
H	0.599702000000	1.300529000000	2.295821000000
C	0.722754000000	2.005177000000	0.291653000000
C	1.845593000000	2.775430000000	0.659945000000
C	0.261569000000	2.092876000000	-1.037887000000
H	2.210541000000	2.726370000000	1.698072000000
H	-0.629705000000	1.538317000000	-1.356108000000
C	2.497856000000	3.599305000000	-0.268590000000
C	0.913811000000	2.912460000000	-1.969994000000
H	3.370508000000	4.192182000000	0.045307000000
H	0.517185000000	2.962945000000	-2.994819000000
C	2.037276000000	3.664786000000	-1.592782000000
H	2.548953000000	4.306322000000	-2.326704000000
C	0.016345000000	-1.178982000000	-1.449123000000
C	-0.249340000000	-1.457359000000	-2.821067000000

C	-1.400563000000	-2.192716000000	-3.200478000000
C	0.622366000000	-0.964707000000	-3.825904000000
H	-2.089178000000	-2.556636000000	-2.425195000000
H	1.504608000000	-0.379402000000	-3.529066000000
C	-1.661085000000	-2.434370000000	-4.554238000000
C	0.352998000000	-1.221088000000	-5.175838000000
H	-2.561207000000	-3.001478000000	-4.835890000000
H	1.038887000000	-0.841464000000	-5.948374000000
C	-0.785510000000	-1.958382000000	-5.544957000000
H	-0.989864000000	-2.161060000000	-6.607235000000
C	-4.908293000000	-2.275351000000	-1.863472000000
C	-4.671524000000	-0.899281000000	-1.750167000000
C	-4.283211000000	-3.206852000000	-0.999049000000
H	-5.190530000000	-0.173499000000	-2.389295000000
H	-4.495319000000	-4.280396000000	-1.114007000000
C	-3.777251000000	-0.457037000000	-0.754938000000
C	-3.402676000000	-2.784769000000	0.009873000000
H	-2.907274000000	-3.487259000000	0.694188000000
C	-3.178801000000	-1.408004000000	0.098739000000
H	-5.605362000000	-2.641635000000	-2.632291000000
C	-2.387372000000	0.580352000000	0.724089000000
C	-3.250269000000	0.838176000000	-0.336758000000
O	-2.361600000000	-0.780380000000	0.993867000000
C	-1.445982000000	1.300140000000	1.651625000000
H	-1.565699000000	0.728369000000	2.595916000000
C	-1.717585000000	2.752614000000	1.997628000000
C	-1.817427000000	3.108351000000	3.358290000000
C	-1.765996000000	3.779757000000	1.033766000000
H	-1.775147000000	2.317437000000	4.124676000000
H	-1.696321000000	3.526466000000	-0.028078000000
C	-1.957100000000	4.448395000000	3.749484000000
C	-1.908724000000	5.118476000000	1.420748000000
H	-2.031259000000	4.701212000000	4.818368000000
H	-1.954362000000	5.901671000000	0.649177000000
C	-1.999153000000	5.459973000000	2.778401000000
H	-2.107104000000	6.513394000000	3.078600000000
N	-3.506168000000	2.068114000000	-0.959897000000
S	-5.064410000000	2.685270000000	-1.083688000000
O	-6.045536000000	1.627559000000	-0.753147000000
O	-5.127425000000	3.415439000000	-2.376117000000
C	-5.173796000000	3.909865000000	0.226835000000
C	-5.228045000000	3.473916000000	1.561944000000

C	-5.225942000000	5.272445000000	-0.089483000000
H	-5.205241000000	2.398800000000	1.790232000000
H	-5.202622000000	5.586066000000	-1.142905000000
C	-5.316718000000	4.423334000000	2.582461000000
C	-5.324165000000	6.210838000000	0.949428000000
H	-5.344379000000	4.086883000000	3.630437000000
H	-5.370800000000	7.284167000000	0.705955000000
C	-5.368271000000	5.806206000000	2.296691000000
C	-5.459620000000	6.809481000000	3.416427000000
H	-5.510933000000	7.847752000000	3.035903000000
H	-6.356388000000	6.631071000000	4.045394000000
H	-4.578349000000	6.734304000000	4.088009000000
C	-2.443407000000	2.680773000000	-4.152093000000
C	-3.166382000000	3.009522000000	-6.570051000000
H	-4.281306000000	3.085033000000	-6.597705000000
H	-2.797132000000	3.759598000000	-7.301045000000
C	-2.733738000000	1.621037000000	-7.040912000000
H	-1.657071000000	1.486200000000	-6.804623000000
H	-2.802686000000	1.605679000000	-8.148403000000
C	-3.567791000000	0.475177000000	-6.458934000000
H	-3.058865000000	-0.485404000000	-6.684754000000
H	-4.553611000000	0.431831000000	-6.969437000000
C	-3.773258000000	0.611710000000	-4.944692000000
H	-4.021207000000	-0.377506000000	-4.511865000000
H	-4.640252000000	1.268838000000	-4.718307000000
C	-2.539686000000	1.166683000000	-4.219888000000
H	-1.607490000000	0.749886000000	-4.657182000000
H	-2.538804000000	0.829196000000	-3.168158000000
C	-2.774769000000	4.911301000000	-5.075688000000
H	-2.560074000000	5.396018000000	-6.050306000000
H	-3.821383000000	5.177913000000	-4.798833000000
C	-1.804856000000	5.393472000000	-4.004370000000
H	-1.937423000000	6.480199000000	-3.829655000000
H	-0.762012000000	5.239170000000	-4.355091000000
C	-2.051416000000	4.592775000000	-2.731943000000
H	-3.012689000000	4.899637000000	-2.261071000000
H	-1.248008000000	4.776514000000	-1.986248000000
N	-2.669085000000	3.460403000000	-5.265103000000
N	-2.134282000000	3.163558000000	-2.974361000000

TS1$_{D.d\text{-}SR}$

Electronic energy = -2681.042218 Hartree

C	0.429638000000	-0.356830000000	1.107541000000
O	0.817352000000	-1.065166000000	2.028216000000
C	0.236460000000	-0.855760000000	-0.242237000000
C	0.108774000000	1.140856000000	1.335327000000
H	-2.667304000000	2.521077000000	-1.983547000000
H	0.610499000000	1.359839000000	2.298381000000
C	0.724508000000	2.040255000000	0.285305000000
C	1.821856000000	2.849555000000	0.646533000000
C	0.283939000000	2.074652000000	-1.053714000000
H	2.169230000000	2.843096000000	1.691697000000
H	-0.589132000000	1.485480000000	-1.359131000000
C	2.472522000000	3.656421000000	-0.297885000000
C	0.941671000000	2.869435000000	-2.003743000000
H	3.324910000000	4.280491000000	0.011088000000
H	0.576421000000	2.869175000000	-3.041871000000
C	2.040593000000	3.660618000000	-1.633261000000
H	2.556052000000	4.282438000000	-2.381372000000
C	-0.011459000000	-1.170184000000	-1.406544000000
C	-0.302012000000	-1.441995000000	-2.775087000000
C	-1.453919000000	-2.184362000000	-3.137884000000
C	0.542927000000	-0.930082000000	-3.793775000000
H	-2.120412000000	-2.566511000000	-2.352448000000
H	1.427785000000	-0.342314000000	-3.509729000000
C	-1.743324000000	-2.411069000000	-4.488543000000
C	0.246039000000	-1.173159000000	-5.140691000000
H	-2.644719000000	-2.983022000000	-4.755912000000
H	0.912978000000	-0.780825000000	-5.923541000000
C	-0.894705000000	-1.915735000000	-5.493149000000
H	-1.120545000000	-2.108634000000	-6.552951000000
C	-4.866850000000	-2.324496000000	-1.793884000000
C	-4.623472000000	-0.946403000000	-1.726967000000
C	-4.258858000000	-3.227146000000	-0.887777000000
H	-5.135406000000	-0.241067000000	-2.393911000000
H	-4.474287000000	-4.303386000000	-0.966177000000
C	-3.742791000000	-0.469037000000	-0.734901000000
C	-3.391896000000	-2.770872000000	0.117904000000
H	-2.910252000000	-3.449527000000	0.835458000000
C	-3.163547000000	-1.392161000000	0.162034000000
H	-5.556398000000	-2.714489000000	-2.557985000000
C	-2.374704000000	0.615588000000	0.728299000000
C	-3.213169000000	0.844284000000	-0.362027000000
O	-2.360139000000	-0.739866000000	1.047982000000
C	-1.438879000000	1.347762000000	1.652128000000
H	-1.559493000000	0.785814000000	2.602015000000
C	-1.703437000000	2.803699000000	1.986243000000
C	-1.743596000000	3.182462000000	3.343653000000
C	-1.804562000000	3.812733000000	1.008613000000
H	-1.661543000000	2.405496000000	4.121044000000
H	-1.797374000000	3.532305000000	-0.048861000000
C	-1.878541000000	4.528188000000	3.717113000000
C	-1.940545000000	5.157305000000	1.377429000000
H	-1.908585000000	4.799469000000	4.783638000000
H	-2.032341000000	5.926273000000	0.595645000000
C	-1.972876000000	5.522327000000	2.731631000000
H	-2.078601000000	6.579780000000	3.018388000000
N	-3.434394000000	2.040994000000	-1.052414000000
S	-4.976501000000	2.641441000000	-1.207325000000
O	-6.005364000000	1.596364000000	-0.979093000000
O	-4.996066000000	3.435603000000	-2.470334000000
C	-5.150726000000	3.817854000000	0.143664000000
C	-5.179611000000	3.343647000000	1.466661000000
C	-5.260055000000	5.185579000000	-0.131873000000
H	-5.112151000000	2.264433000000	1.665354000000
H	-5.260336000000	5.519424000000	-1.179548000000
C	-5.295152000000	4.259946000000	2.514656000000
C	-5.384913000000	6.091233000000	0.933144000000
H	-5.301416000000	3.893981000000	3.553200000000
H	-5.476241000000	7.168628000000	0.721434000000
C	-5.398655000000	5.647657000000	2.268975000000
C	-5.508101000000	6.615339000000	3.418334000000
H	-5.634408000000	7.658015000000	3.068169000000
H	-6.367695000000	6.368072000000	4.075136000000
H	-4.598090000000	6.576606000000	4.054114000000
C	-2.449245000000	2.663760000000	-4.144861000000
C	-3.002639000000	3.042577000000	-6.590116000000
H	-4.100191000000	3.218247000000	-6.695140000000
H	-2.513385000000	3.753604000000	-7.288395000000
C	-2.661421000000	1.618901000000	-7.026499000000
H	-1.614236000000	1.396171000000	-6.731302000000
H	-2.668195000000	1.603167000000	-8.136064000000
C	-3.618977000000	0.549348000000	-6.491653000000
H	-3.176454000000	-0.449962000000	-6.686597000000
H	-4.573858000000	0.581793000000	-7.058261000000
C	-3.900235000000	0.710553000000	-4.992219000000

H	-4.249616000000	-0.253633000000	-4.572405000000
H	-4.723821000000	1.433167000000	-4.809522000000
C	-2.672366000000	1.167574000000	-4.196040000000
H	-1.750188000000	0.669051000000	-4.562612000000
H	-2.774900000000	0.850708000000	-3.142929000000
C	-2.580252000000	4.914697000000	-5.052199000000
H	-2.312858000000	5.385549000000	-6.019450000000
H	-3.612036000000	5.245433000000	-4.794307000000
C	-1.600081000000	5.321910000000	-3.959412000000
H	-1.654789000000	6.414679000000	-3.784882000000
H	-0.564407000000	5.091033000000	-4.288057000000
C	-1.930716000000	4.551022000000	-2.690026000000
H	-2.857901000000	4.944548000000	-2.217514000000
H	-1.114466000000	4.650227000000	-1.944383000000
N	-2.572031000000	3.458338000000	-5.247433000000
N	-2.133387000000	3.137158000000	-2.956318000000

$I_{D.d\text{-}SR}$

Electronic energy = -2681.048253 Hartree

C	0.370692000000	-0.342003000000	1.014948000000
O	0.668677000000	-1.075599000000	1.949070000000
C	0.254464000000	-0.813546000000	-0.353587000000
C	0.087799000000	1.163700000000	1.240506000000
H	-2.146095000000	2.767753000000	-2.446306000000
H	0.522003000000	1.353503000000	2.241751000000
C	0.816034000000	2.048474000000	0.252312000000
C	1.928070000000	2.791308000000	0.700556000000
C	0.473273000000	2.121537000000	-1.113728000000
H	2.198640000000	2.755038000000	1.767537000000
H	-0.407476000000	1.580406000000	-1.481725000000
C	2.689800000000	3.567671000000	-0.184505000000
C	1.246096000000	2.880220000000	-2.005239000000
H	3.551310000000	4.139645000000	0.192480000000
H	0.965129000000	2.903165000000	-3.069100000000
C	2.358961000000	3.603979000000	-1.547760000000
H	2.964492000000	4.196824000000	-2.250552000000
C	0.085268000000	-1.088065000000	-1.541825000000
C	-0.110594000000	-1.309258000000	-2.935894000000
C	-1.243328000000	-2.021021000000	-3.404728000000
C	0.812461000000	-0.777431000000	-3.873676000000
H	-1.969185000000	-2.418314000000	-2.681756000000
H	1.682107000000	-0.213611000000	-3.506246000000
C	-1.437159000000	-2.199384000000	-4.779663000000
C	0.610876000000	-0.972419000000	-5.245920000000
H	-2.324196000000	-2.748848000000	-5.129603000000
H	1.338764000000	-0.567350000000	-5.965551000000
C	-0.511291000000	-1.685498000000	-5.703817000000
H	-0.661960000000	-1.842036000000	-6.782860000000
C	-4.709800000000	-2.124817000000	-2.309915000000
C	-4.423966000000	-0.756114000000	-2.218918000000
C	-4.220921000000	-3.046370000000	-1.352121000000
H	-4.846043000000	-0.030979000000	-2.926448000000
H	-4.465689000000	-4.114672000000	-1.451355000000
C	-3.625161000000	-0.306927000000	-1.147156000000
C	-3.436061000000	-2.618361000000	-0.269426000000
H	-3.046538000000	-3.312545000000	0.488185000000
C	-3.164776000000	-1.248201000000	-0.200718000000
H	-5.338587000000	-2.491639000000	-3.135600000000
C	-2.354506000000	0.731302000000	0.431701000000
C	-3.078250000000	0.989324000000	-0.732930000000
O	-2.422208000000	-0.625585000000	0.754734000000
C	-1.471823000000	1.424250000000	1.434616000000
H	-1.687675000000	0.860145000000	2.366283000000
C	-1.698954000000	2.887464000000	1.766164000000
C	-1.749358000000	3.260775000000	3.124896000000
C	-1.755512000000	3.903621000000	0.792203000000
H	-1.706232000000	2.477959000000	3.899677000000
H	-1.763503000000	3.622767000000	-0.266727000000
C	-1.849142000000	4.607902000000	3.504058000000
C	-1.853516000000	5.250009000000	1.167667000000
H	-1.890204000000	4.874165000000	4.571506000000
H	-1.913054000000	6.025568000000	0.389195000000
C	-1.894515000000	5.609901000000	2.522943000000
H	-1.971943000000	6.668699000000	2.814087000000
N	-3.160743000000	2.182286000000	-1.454052000000
S	-4.637368000000	2.847651000000	-1.760403000000
O	-5.748513000000	1.859544000000	-1.743394000000
O	-4.475079000000	3.720481000000	-2.965794000000
C	-4.942034000000	3.956926000000	-0.371782000000
C	-5.106001000000	3.413858000000	0.914766000000
C	-4.990595000000	5.340917000000	-0.575113000000
H	-5.086776000000	2.323899000000	1.057258000000
H	-4.884288000000	5.731270000000	-1.597652000000
C	-5.286543000000	4.274508000000	2.000715000000

C	-5.184450000000	6.190534000000	0.525017000000
H	-5.395160000000	3.851717000000	3.011821000000
H	-5.225896000000	7.280598000000	0.369039000000
C	-5.324544000000	5.676097000000	1.828079000000
C	-5.489746000000	6.585218000000	3.017832000000
H	-5.617436000000	7.642062000000	2.713152000000
H	-6.366809000000	6.296554000000	3.632971000000
H	-4.600013000000	6.528283000000	3.680905000000
C	-1.968761000000	2.896643000000	-4.482003000000
C	-2.289976000000	3.331809000000	-6.949360000000
H	-3.353726000000	3.583308000000	-7.172322000000
H	-1.676162000000	4.009492000000	-7.579164000000
C	-1.998012000000	1.888170000000	-7.351991000000
H	-1.002012000000	1.599782000000	-6.953807000000
H	-1.894125000000	1.871207000000	-8.456637000000
C	-3.068809000000	0.882059000000	-6.919375000000
H	-2.678710000000	-0.142499000000	-7.093339000000
H	-3.965591000000	0.986586000000	-7.566250000000
C	-3.468190000000	1.045704000000	-5.447169000000
H	-3.902993000000	0.099513000000	-5.068381000000
H	-4.263243000000	1.810422000000	-5.318910000000
C	-2.292729000000	1.422836000000	-4.540822000000
H	-1.376878000000	0.850627000000	-4.800339000000
H	-2.531079000000	1.145354000000	-3.496249000000
C	-1.959889000000	5.169318000000	-5.333158000000
H	-1.634997000000	5.649628000000	-6.276915000000
H	-2.992639000000	5.520063000000	-5.113033000000
C	-1.018067000000	5.523054000000	-4.187904000000
H	-1.047545000000	6.613052000000	-3.993913000000
H	0.024416000000	5.266440000000	-4.472663000000
C	-1.433435000000	4.746322000000	-2.948621000000
H	-2.373690000000	5.151596000000	-2.515363000000
H	-0.652922000000	4.790757000000	-2.161869000000
N	-1.991851000000	3.714998000000	-5.560443000000
N	-1.670576000000	3.349770000000	-3.273195000000

$X_{D.d}$

Electronic energy = -2681.049567 Hartree

C	0.613045000000	1.638610000000	-1.173255000000
O	1.664864000000	2.304458000000	-1.763840000000
C	0.218531000000	0.462264000000	-1.862998000000
C	-0.026796000000	2.110419000000	-0.051489000000
H	-1.716413000000	3.372229000000	-2.546059000000
H	1.879809000000	3.055150000000	-1.165313000000
C	0.449078000000	3.402175000000	0.520258000000
C	1.732061000000	3.489707000000	1.112805000000
C	-0.351601000000	4.566136000000	0.462750000000
H	2.352601000000	2.582278000000	1.187686000000
H	-1.334340000000	4.505827000000	-0.026457000000
C	2.202470000000	4.706876000000	1.631944000000
C	0.121836000000	5.776655000000	0.986917000000
H	3.199314000000	4.753552000000	2.096612000000
H	-0.515459000000	6.672512000000	0.927974000000
C	1.396222000000	5.853614000000	1.572697000000
H	1.761462000000	6.807315000000	1.983428000000
C	-0.165176000000	-0.519294000000	-2.503712000000
C	-0.687785000000	-1.590857000000	-3.281875000000
C	-2.045857000000	-1.978593000000	-3.134196000000
C	0.110322000000	-2.251203000000	-4.252634000000
H	-2.673242000000	-1.471250000000	-2.389611000000
H	1.164172000000	-1.958589000000	-4.367720000000
C	-2.585006000000	-2.986740000000	-3.940298000000
C	-0.441468000000	-3.256835000000	-5.054844000000
H	-3.642568000000	-3.264139000000	-3.813595000000
H	0.187505000000	-3.759009000000	-5.805657000000
C	-1.789197000000	-3.628296000000	-4.904857000000
H	-2.217597000000	-4.418255000000	-5.539975000000
C	-6.170218000000	0.052283000000	-2.516050000000
C	-5.325507000000	1.155623000000	-2.329869000000
C	-5.965032000000	-1.165567000000	-1.826005000000
H	-5.498130000000	2.112447000000	-2.842543000000
H	-6.655035000000	-2.007672000000	-1.987379000000
C	-4.235547000000	1.031673000000	-1.436343000000
C	-4.895559000000	-1.312338000000	-0.929437000000
H	-4.709674000000	-2.247475000000	-0.382010000000
C	-4.054234000000	-0.207096000000	-0.766390000000
H	-7.020141000000	0.139060000000	-3.210168000000
C	-2.379263000000	1.087430000000	-0.132845000000
C	-3.128426000000	1.884433000000	-0.990587000000
O	-2.930494000000	-0.177567000000	0.005810000000
C	-1.118760000000	1.292475000000	0.654165000000
H	-0.705723000000	0.265528000000	0.762904000000
C	-1.363096000000	1.800507000000	2.077162000000
C	-0.454165000000	1.449033000000	3.093617000000

C	-2.438005000000	2.650426000000	2.390942000000
H	0.391575000000	0.783691000000	2.854964000000
H	-3.158336000000	2.931221000000	1.609216000000
C	-0.610195000000	1.938649000000	4.397905000000
C	-2.595223000000	3.142859000000	3.696118000000
H	0.108298000000	1.651120000000	5.181000000000
H	-3.438578000000	3.812837000000	3.918482000000
C	-1.683866000000	2.790767000000	4.702865000000
H	-1.810073000000	3.177634000000	5.725717000000
N	-2.721499000000	3.164872000000	-1.359401000000
S	-3.761917000000	4.407605000000	-1.595076000000
O	-4.841399000000	4.124901000000	-2.589500000000
O	-2.891090000000	5.591580000000	-1.850244000000
C	-4.573551000000	4.663111000000	-0.005538000000
C	-5.615608000000	3.810921000000	0.397939000000
C	-4.099978000000	5.670019000000	0.847122000000
H	-5.998745000000	3.043368000000	-0.290086000000
H	-3.299013000000	6.334059000000	0.490902000000
C	-6.157156000000	3.956853000000	1.681007000000
C	-4.662246000000	5.809703000000	2.123254000000
H	-6.967827000000	3.283651000000	2.002591000000
H	-4.292715000000	6.601729000000	2.793962000000
C	-5.689610000000	4.951682000000	2.567213000000
C	-6.262038000000	5.071691000000	3.957001000000
H	-5.954064000000	6.015934000000	4.446417000000
H	-7.370253000000	5.033098000000	3.946484000000
H	-5.919936000000	4.233448000000	4.602163000000
C	-1.197283000000	2.780413000000	-4.463372000000
C	-0.427615000000	2.064699000000	-6.749437000000
H	-1.145241000000	2.442218000000	-7.514418000000
H	0.580752000000	2.182401000000	-7.193851000000
C	-0.670548000000	0.582539000000	-6.457685000000
H	-0.136686000000	0.324218000000	-5.519188000000
H	-0.177912000000	-0.012345000000	-7.253950000000
C	-2.151557000000	0.195538000000	-6.360501000000
H	-2.230317000000	-0.782413000000	-5.842340000000
H	-2.573167000000	0.046302000000	-7.377451000000
C	-2.974504000000	1.252105000000	-5.617178000000
H	-3.960971000000	0.832290000000	-5.333660000000
H	-3.200058000000	2.113790000000	-6.281682000000
C	-2.306864000000	1.756773000000	-4.339030000000
H	-1.926222000000	0.910145000000	-3.729829000000
H	-3.064889000000	2.250075000000	-3.701680000000
C	0.458306000000	4.119571000000	-5.638004000000
H	1.264836000000	3.888179000000	-6.360650000000
H	-0.107793000000	4.986847000000	-6.047620000000
C	1.041507000000	4.455638000000	-4.268025000000
H	1.660287000000	5.371582000000	-4.336903000000
H	1.690724000000	3.622651000000	-3.928507000000
C	-0.092164000000	4.638875000000	-3.271610000000
H	-0.639447000000	5.596546000000	-3.407699000000
H	0.273883000000	4.642761000000	-2.226142000000
N	-0.429168000000	2.946339000000	-5.569424000000
N	-1.043098000000	3.550531000000	-3.401185000000

TS2*D.d-SR*

Electronic energy = -2681.025154 Hartree

C	-0.043822000000	4.009870000000	0.068851000000
O	-1.240567000000	4.249112000000	-0.232392000000
C	0.997303000000	4.111753000000	-0.881172000000
C	0.353299000000	3.552531000000	1.476357000000
H	-1.524204000000	4.238822000000	-1.896270000000
H	1.367145000000	3.964770000000	1.660127000000
C	-0.559842000000	4.037112000000	2.581286000000
C	0.012274000000	4.660409000000	3.709841000000
C	-1.957980000000	3.840127000000	2.557648000000
H	1.102746000000	4.817624000000	3.740830000000
H	-2.420861000000	3.364946000000	1.684928000000
C	-0.781460000000	5.078840000000	4.787809000000
C	-2.751970000000	4.262255000000	3.634479000000
H	-0.312170000000	5.563262000000	5.657859000000
H	-3.840651000000	4.102071000000	3.598569000000
C	-2.170361000000	4.880287000000	4.752550000000
H	-2.798439000000	5.207168000000	5.595527000000
C	1.802489000000	3.776453000000	-1.785588000000
C	2.778906000000	4.117980000000	-2.786002000000
C	3.299926000000	5.442298000000	-2.738134000000
C	3.205601000000	3.263347000000	-3.827420000000
H	2.996791000000	6.100315000000	-1.910138000000
H	2.862737000000	2.222495000000	-3.861112000000
C	4.196326000000	5.894572000000	-3.713343000000
C	4.100987000000	3.729456000000	-4.800165000000
H	4.593689000000	6.919323000000	-3.650662000000
H	4.424141000000	3.041923000000	-5.596570000000

C	4.596452000000	5.042105000000	-4.756858000000
H	5.304596000000	5.395711000000	-5.521479000000
C	6.145904000000	0.611516000000	-0.516238000000
C	4.879838000000	0.791948000000	-1.088237000000
C	6.348407000000	0.696970000000	0.881471000000
H	4.712538000000	0.701124000000	-2.169577000000
H	7.356942000000	0.545174000000	1.295370000000
C	3.790736000000	1.065069000000	-0.231335000000
C	5.282828000000	0.974233000000	1.750439000000
H	5.414855000000	1.050891000000	2.839101000000
C	4.028860000000	1.157626000000	1.160458000000
H	7.004926000000	0.395492000000	-1.169659000000
C	1.858635000000	1.550167000000	0.871344000000
C	2.360303000000	1.322785000000	-0.410866000000
O	2.873318000000	1.458212000000	1.820061000000
C	0.563951000000	1.974553000000	1.529429000000
H	0.768376000000	1.796450000000	2.603937000000
C	-0.688510000000	1.182986000000	1.203042000000
C	-1.162846000000	1.018129000000	-0.112734000000
C	-1.446857000000	0.646118000000	2.264329000000
H	-0.541863000000	1.384927000000	-0.943141000000
H	-1.088416000000	0.773802000000	3.297678000000
C	-2.371454000000	0.349947000000	-0.354220000000
C	-2.651797000000	-0.029573000000	2.023168000000
H	-2.722946000000	0.222259000000	-1.389742000000
H	-3.227184000000	-0.440027000000	2.867109000000
C	-3.123723000000	-0.172546000000	0.709188000000
H	-4.071782000000	-0.697372000000	0.514533000000
N	1.642036000000	1.497953000000	-1.597599000000
S	1.642874000000	0.326334000000	-2.743557000000
O	2.974995000000	-0.021837000000	-3.328035000000
O	0.596300000000	0.728406000000	-3.741937000000
C	1.046670000000	-1.153167000000	-1.908756000000
C	-0.272907000000	-1.577979000000	-2.116910000000
C	1.886113000000	-1.828943000000	-1.008790000000
H	-0.895515000000	-1.038942000000	-2.844770000000
H	2.929928000000	-1.509467000000	-0.879824000000
C	-0.763647000000	-2.672465000000	-1.396622000000
C	1.373755000000	-2.915040000000	-0.286757000000
H	-1.802447000000	-3.003726000000	-1.554803000000
H	2.025415000000	-3.440689000000	0.428966000000
C	0.042392000000	-3.347815000000	-0.456209000000
C	-0.630712000000	4.845209000000	-3.614754000000
C	0.759217000000	5.435301000000	-5.522069000000
H	1.523991000000	5.818622000000	-4.818322000000
H	1.322196000000	4.848776000000	-6.271884000000
C	-0.015828000000	6.594421000000	-6.179772000000
H	-0.373683000000	6.290207000000	-7.185926000000
H	0.697424000000	7.429792000000	-6.343996000000
C	-1.222564000000	7.068593000000	-5.357378000000
H	-2.018110000000	6.294228000000	-5.415704000000
H	-1.656297000000	7.970144000000	-5.836956000000
C	-0.946536000000	7.375264000000	-3.877580000000
H	-1.905916000000	7.670534000000	-3.406156000000
H	-0.277022000000	8.256182000000	-3.789903000000
C	-0.326948000000	6.209445000000	-3.056280000000
H	-0.681933000000	6.234141000000	-2.009466000000
H	0.775092000000	6.309480000000	-3.008989000000
C	-0.373267000000	3.175651000000	-5.394088000000
H	-0.377695000000	3.295467000000	-6.495592000000
H	0.444135000000	2.477953000000	-5.109088000000
C	-1.702655000000	2.616232000000	-4.896035000000
H	-1.793485000000	1.563754000000	-5.224265000000
H	-2.550799000000	3.189530000000	-5.328176000000
C	-1.733345000000	2.678615000000	-3.373447000000
H	-0.967294000000	1.976304000000	-2.978951000000
H	-2.723136000000	2.397026000000	-2.963584000000
N	-0.109960000000	4.498535000000	-4.803642000000
N	-1.430649000000	4.034829000000	-2.931574000000
C	-0.521384000000	-4.484513000000	0.357371000000
H	-1.051628000000	-5.220282000000	-0.281509000000
H	0.268701000000	-5.020604000000	0.917754000000
H	-1.260732000000	-4.108760000000	1.096891000000

II$_{D.d\text{-}SR}$

Electronic energy = -2681.029667 Hartree

C	0.582617000000	0.844045000000	-0.201130000000
O	1.361491000000	-0.157338000000	-0.521850000000
C	-0.140873000000	1.579804000000	-1.096918000000
C	0.511442000000	1.149140000000	1.303068000000
H	1.625183000000	-0.058959000000	-1.834744000000
H	0.693395000000	0.137158000000	1.729492000000
C	1.623091000000	2.035553000000	1.850615000000
C	1.466981000000	2.770737000000	3.043390000000

C	2.865479000000	2.098656000000	1.184868000000
H	0.509165000000	2.749050000000	3.582624000000
H	3.006440000000	1.502180000000	0.271842000000
C	2.514445000000	3.556417000000	3.547360000000
C	3.912086000000	2.884919000000	1.687101000000
H	2.364600000000	4.128709000000	4.475752000000
H	4.872453000000	2.920225000000	1.149405000000
C	3.741272000000	3.621369000000	2.870165000000
H	4.561018000000	4.241951000000	3.263123000000
C	-1.311574000000	1.924292000000	-1.634164000000
C	-1.384567000000	2.806835000000	-2.829343000000
C	-2.175654000000	2.458799000000	-3.946410000000
C	-0.577736000000	3.964218000000	-2.917376000000
H	-2.809861000000	1.562113000000	-3.891848000000
H	0.034760000000	4.239171000000	-2.046794000000
C	-2.165123000000	3.243018000000	-5.111888000000
C	-0.575250000000	4.751548000000	-4.074650000000
H	-2.794163000000	2.951059000000	-5.967778000000
H	0.045002000000	5.660522000000	-4.112948000000
C	-1.370351000000	4.397023000000	-5.182056000000
H	-1.369865000000	5.019637000000	-6.089519000000
C	-4.371659000000	-2.910253000000	-0.704916000000
C	-3.946217000000	-1.624502000000	-1.065961000000
C	-4.030008000000	-3.476188000000	0.545955000000
H	-4.209846000000	-1.191171000000	-2.041090000000
H	-4.382767000000	-4.487330000000	0.798011000000
C	-3.161007000000	-0.895450000000	-0.148687000000
C	-3.245977000000	-2.770262000000	1.472111000000
H	-2.964714000000	-3.193431000000	2.446585000000
C	-2.830465000000	-1.492828000000	1.091791000000
H	-4.983934000000	-3.495165000000	-1.408145000000
C	-1.876940000000	0.512661000000	1.093545000000
C	-2.527765000000	0.405341000000	-0.130718000000
O	-2.052268000000	-0.640286000000	1.829951000000
C	-0.946429000000	1.513001000000	1.731398000000
H	-1.015362000000	1.287191000000	2.817539000000
C	-1.404862000000	2.962418000000	1.573193000000
C	-2.609873000000	3.341366000000	2.198148000000
C	-0.663576000000	3.947210000000	0.894800000000
H	-3.208385000000	2.580116000000	2.721473000000
H	0.281215000000	3.678900000000	0.404891000000
C	-3.069040000000	4.663412000000	2.140134000000
C	-1.120472000000	5.271646000000	0.836764000000
H	-4.021438000000	4.930759000000	2.622253000000
H	-0.529233000000	6.025273000000	0.294343000000
C	-2.322879000000	5.635611000000	1.458478000000
H	-2.682842000000	6.674300000000	1.404449000000
N	-2.581386000000	1.350008000000	-1.182957000000
S	-3.929312000000	2.521365000000	-0.937994000000
O	-3.414216000000	3.895262000000	-1.110616000000
O	-4.637863000000	2.077408000000	0.282409000000
C	-5.058953000000	2.196969000000	-2.298411000000
C	-5.150270000000	3.122147000000	-3.347905000000
C	-5.889972000000	1.069221000000	-2.248514000000
H	-4.509777000000	4.014411000000	-3.336133000000
H	-5.842005000000	0.390880000000	-1.384990000000
C	-6.061806000000	2.883587000000	-4.383887000000
C	-6.788914000000	0.842698000000	-3.299084000000
H	-6.137486000000	3.607900000000	-5.210646000000
H	-7.442013000000	-0.043685000000	-3.268137000000
C	-6.886725000000	1.738186000000	-4.385485000000
C	-7.858391000000	1.496406000000	-5.512281000000
H	-8.440117000000	0.567240000000	-5.360062000000
H	-8.576960000000	2.336776000000	-5.608444000000
H	-7.332182000000	1.414082000000	-6.486287000000
C	0.835690000000	-0.261373000000	-3.789629000000
C	-0.453780000000	-0.115584000000	-5.958732000000
H	-1.368996000000	0.497202000000	-5.788905000000
H	-0.066215000000	0.186058000000	-6.953611000000
C	-0.802488000000	-1.602170000000	-5.999869000000
H	0.141129000000	-2.186303000000	-5.956240000000
H	-1.236372000000	-1.814880000000	-6.998766000000
C	-1.779214000000	-2.053782000000	-4.907791000000
H	-1.790213000000	-3.164315000000	-4.877254000000
H	-2.812381000000	-1.744968000000	-5.175651000000
C	-1.418759000000	-1.494836000000	-3.526564000000
H	-1.908660000000	-2.095584000000	-2.735958000000
H	-1.818606000000	-0.468148000000	-3.393853000000
C	0.088488000000	-1.468173000000	-3.256856000000
H	0.576011000000	-2.391295000000	-3.640287000000
H	0.277490000000	-1.458446000000	-2.165411000000
C	1.200144000000	1.599396000000	-5.312956000000
H	1.221831000000	1.702153000000	-6.415904000000
H	0.555113000000	2.414060000000	-4.919023000000

C	2.605790000000	1.694511000000	-4.732464000000
H	3.018881000000	2.705410000000	-4.918117000000
H	3.270362000000	0.964900000000	-5.241846000000
C	2.546175000000	1.402376000000	-3.239898000000
H	2.086707000000	2.248219000000	-2.679393000000
H	3.560984000000	1.250212000000	-2.816496000000
N	0.582858000000	0.298989000000	-5.001257000000
N	1.756391000000	0.212949000000	-2.970800000000

TS3$_{D.d\text{-}SR}$

Electronic energy = -2681.016552 Hartree

C	0.916046000000	4.388409000000	-0.702862000000
O	0.103187000000	4.583258000000	-1.644423000000
C	2.325154000000	4.574641000000	-0.772391000000
C	0.531258000000	3.848706000000	0.666222000000
H	0.910347000000	4.843242000000	-3.039937000000
H	1.128159000000	4.441090000000	1.386765000000
C	-0.913909000000	3.921005000000	1.104041000000
C	-1.180412000000	4.162932000000	2.468256000000
C	-2.005709000000	3.705267000000	0.237711000000
H	-0.336758000000	4.336130000000	3.156798000000
H	-1.814734000000	3.534170000000	-0.827504000000
C	-2.492883000000	4.186390000000	2.960374000000
C	-3.319932000000	3.734163000000	0.727559000000
H	-2.674320000000	4.377538000000	4.029225000000
H	-4.159143000000	3.565467000000	0.035117000000
C	-3.570752000000	3.969769000000	2.087842000000
H	-4.603811000000	3.986861000000	2.467702000000
C	3.338785000000	3.938794000000	-0.318460000000
C	4.755959000000	3.911382000000	-0.036934000000
C	5.652258000000	3.218722000000	-0.886860000000
C	5.250851000000	4.517500000000	1.141976000000
H	5.267585000000	2.697072000000	-1.775835000000
H	4.549254000000	5.041625000000	1.806864000000
C	7.011483000000	3.142628000000	-0.560563000000
C	6.612623000000	4.437181000000	1.458822000000
H	7.693903000000	2.585031000000	-1.219087000000
H	6.987552000000	4.908769000000	2.379933000000
C	7.494648000000	3.746941000000	0.611447000000
H	8.562052000000	3.673073000000	0.869679000000
C	6.454329000000	-0.420559000000	0.715375000000
C	5.421100000000	-0.165670000000	-0.190977000000
C	6.463769000000	0.160979000000	2.004733000000
H	5.456745000000	-0.574921000000	-1.205877000000
H	7.297539000000	-0.051422000000	2.691202000000
C	4.361100000000	0.683917000000	0.203500000000
C	5.432176000000	1.011355000000	2.420517000000
H	5.422916000000	1.488766000000	3.409902000000
C	4.396319000000	1.240076000000	1.509145000000
H	7.284927000000	-1.075429000000	0.412630000000
C	2.572357000000	2.106532000000	0.557284000000
C	3.156524000000	1.224477000000	-0.431091000000
O	3.323740000000	2.036590000000	1.746374000000
C	1.103108000000	2.358412000000	0.861453000000
H	1.072374000000	2.246769000000	1.962840000000
C	0.191546000000	1.266649000000	0.323072000000
C	-0.259496000000	1.212957000000	-1.011343000000
C	-0.213851000000	0.246755000000	1.206959000000
H	0.053405000000	1.985343000000	-1.723795000000
H	0.131876000000	0.278517000000	2.252471000000
C	-1.104671000000	0.175582000000	-1.433331000000
C	-1.049639000000	-0.797812000000	0.784724000000
H	-1.446748000000	0.145596000000	-2.477420000000
H	-1.353328000000	-1.578813000000	1.498425000000
C	-1.502814000000	-0.833727000000	-0.542310000000
H	-2.162863000000	-1.645560000000	-0.883946000000
N	2.604735000000	1.097062000000	-1.654607000000
S	3.271277000000	0.191427000000	-2.839052000000
O	4.726658000000	0.451170000000	-3.089177000000
O	2.385990000000	0.345666000000	-4.032420000000
C	3.102884000000	-1.526150000000	-2.309232000000
C	1.997792000000	-1.906666000000	-1.532505000000
C	4.049285000000	-2.470440000000	-2.730596000000
H	1.262858000000	-1.153935000000	-1.213962000000
H	4.907362000000	-2.141926000000	-3.335749000000
C	1.852835000000	-3.250679000000	-1.169892000000
C	3.885839000000	-3.812338000000	-2.360551000000
H	0.987598000000	-3.547453000000	-0.555730000000
H	4.627541000000	-4.558652000000	-2.687159000000
C	2.790953000000	-4.225380000000	-1.572997000000
C	0.860781000000	3.838763000000	-4.805727000000
C	0.868944000000	2.369406000000	-6.743612000000
H	0.083997000000	3.021129000000	-7.179086000000
H	1.600014000000	2.179371000000	-7.554135000000

C	0.256289000000	1.056224000000	-6.232408000000
H	1.044867000000	0.283465000000	-6.139290000000
H	-0.464098000000	0.707485000000	-7.003604000000
C	-0.418836000000	1.199520000000	-4.863757000000
H	0.380101000000	1.210485000000	-4.094431000000
H	-1.002398000000	0.277351000000	-4.664347000000
C	-1.333488000000	2.415161000000	-4.685548000000
H	-1.781090000000	2.356925000000	-3.672512000000
H	-2.184861000000	2.370215000000	-5.397261000000
C	-0.648148000000	3.804539000000	-4.819125000000
H	-0.986993000000	4.478708000000	-4.010762000000
H	-0.952330000000	4.295730000000	-5.769320000000
C	3.027061000000	2.949718000000	-5.586908000000
H	3.220583000000	1.858231000000	-5.617536000000
H	3.506878000000	3.431754000000	-6.467390000000
C	3.564639000000	3.505801000000	-4.275153000000
H	4.666064000000	3.593799000000	-4.330360000000
H	3.327350000000	2.807248000000	-3.449525000000
C	2.903093000000	4.842680000000	-3.960806000000
H	3.156852000000	5.612557000000	-4.724220000000
H	3.202288000000	5.221821000000	-2.964753000000
N	1.566163000000	3.135412000000	-5.705255000000
N	1.457331000000	4.652200000000	-3.939591000000
C	2.638166000000	-5.662809000000	-1.144698000000
H	1.577268000000	-5.985173000000	-1.173422000000
H	3.226847000000	-6.347262000000	-1.787041000000
H	2.990235000000	-5.804938000000	-0.099333000000

III$_{D.d\text{-}SR}$

Electronic energy = -2681.035968 Hartree

C	-1.112615000000	2.577495000000	3.202941000000
O	-2.232905000000	2.150294000000	2.774252000000
C	-0.536772000000	3.824256000000	2.868596000000
C	-0.174334000000	1.711868000000	4.043483000000
H	-2.849999000000	3.269318000000	1.945395000000
H	0.263376000000	2.354917000000	4.831989000000
C	-0.711680000000	0.460984000000	4.692289000000
C	-0.294599000000	0.143447000000	6.002262000000
C	-1.579895000000	-0.434136000000	4.029615000000
H	0.383707000000	0.833913000000	6.529991000000
H	-1.925969000000	-0.193191000000	3.017285000000
C	-0.725131000000	-1.029969000000	6.638333000000
C	-2.013240000000	-1.606222000000	4.666654000000
H	-0.385526000000	-1.255663000000	7.661042000000
H	-2.691477000000	-2.291346000000	4.134700000000
C	-1.588421000000	-1.911644000000	5.969263000000
H	-1.930297000000	-2.834276000000	6.463229000000
C	0.769693000000	3.978510000000	2.575853000000
C	1.390457000000	5.331240000000	2.444624000000
C	2.302499000000	5.682798000000	1.422796000000
C	1.000959000000	6.338156000000	3.360492000000
H	2.616193000000	4.953653000000	0.665398000000
H	0.297327000000	6.061044000000	4.160339000000
C	2.796079000000	6.994324000000	1.321499000000
C	1.499823000000	7.642453000000	3.263914000000
H	3.497081000000	7.239105000000	0.508717000000
H	1.192529000000	8.401805000000	4.000276000000
C	2.400509000000	7.979460000000	2.237357000000
H	2.794103000000	9.004543000000	2.157314000000
C	6.411000000000	3.342101000000	2.314672000000
C	5.345851000000	2.997548000000	1.478390000000
C	6.199284000000	3.612767000000	3.686594000000
H	5.510311000000	2.771789000000	0.417813000000
H	7.056367000000	3.884818000000	4.321889000000
C	4.047088000000	2.923851000000	2.032098000000
C	4.923572000000	3.542415000000	4.258741000000
H	4.741667000000	3.753220000000	5.321412000000
C	3.860743000000	3.192199000000	3.412979000000
H	7.427926000000	3.401383000000	1.900426000000
C	1.739813000000	2.752445000000	2.654011000000
C	2.724158000000	2.641779000000	1.488645000000
O	2.583373000000	3.087423000000	3.819491000000
C	1.044197000000	1.411978000000	3.056291000000
H	1.803451000000	0.888526000000	3.669579000000
C	0.642424000000	0.457582000000	1.953081000000
C	-0.218179000000	0.829495000000	0.902761000000
C	1.051043000000	-0.887746000000	2.031517000000
H	-0.509513000000	1.880124000000	0.809829000000
H	1.724106000000	-1.196533000000	2.846537000000
C	-0.694918000000	-0.118778000000	-0.009773000000
C	0.592388000000	-1.841192000000	1.108808000000
H	-1.372845000000	0.195584000000	-0.818453000000
H	0.920133000000	-2.888027000000	1.197186000000
C	-0.296402000000	-1.462227000000	0.091965000000

H	-0.672635000000	-2.209531000000	-0.623822000000
N	2.249842000000	2.416645000000	0.288589000000
S	3.248100000000	2.486013000000	-1.069055000000
O	4.347982000000	1.488906000000	-1.017103000000
O	3.570867000000	3.913094000000	-1.359973000000
C	2.095261000000	1.918706000000	-2.318979000000
C	1.784816000000	2.762115000000	-3.391437000000
C	1.585701000000	0.613593000000	-2.243745000000
H	2.220494000000	3.771282000000	-3.423130000000
H	1.850087000000	-0.036658000000	-1.398168000000
C	0.923540000000	2.294836000000	-4.395799000000
C	0.723038000000	0.168736000000	-3.249975000000
H	0.673268000000	2.951743000000	-5.243855000000
H	0.306463000000	-0.849104000000	-3.186898000000
C	0.374952000000	0.997939000000	-4.340286000000
C	-2.738596000000	4.569121000000	0.305788000000
C	-2.192499000000	6.177072000000	-1.452603000000
H	-2.682436000000	5.492248000000	-2.173341000000
H	-2.553188000000	7.195610000000	-1.699514000000
C	-0.665648000000	6.067745000000	-1.562030000000
H	-0.176822000000	6.918367000000	-1.041151000000
H	-0.397727000000	6.159962000000	-2.636846000000
C	-0.150156000000	4.742650000000	-0.986560000000
H	-0.092920000000	4.801971000000	0.120223000000
H	0.893242000000	4.575207000000	-1.315017000000
C	-1.013867000000	3.536865000000	-1.372960000000
H	-0.439164000000	2.619702000000	-1.145269000000
H	-1.166619000000	3.521188000000	-2.472397000000
C	-2.394090000000	3.450640000000	-0.651502000000
H	-2.470302000000	2.515668000000	-0.063367000000
H	-3.219845000000	3.399244000000	-1.394426000000
C	-2.792643000000	6.973437000000	0.841109000000
H	-2.013215000000	7.718300000000	0.579359000000
H	-3.780939000000	7.464236000000	0.690778000000
C	-2.612001000000	6.506628000000	2.277893000000
H	-2.885225000000	7.322037000000	2.975391000000
H	-1.553329000000	6.222980000000	2.459205000000
C	-3.453226000000	5.260481000000	2.525508000000
H	-4.542936000000	5.487607000000	2.496023000000
H	-3.218340000000	4.819371000000	3.512816000000
N	-2.669776000000	5.854128000000	-0.105594000000
N	-3.140051000000	4.246688000000	1.524532000000
C	-0.552400000000	0.492395000000	-5.415951000000
H	-1.520112000000	0.156897000000	-4.988333000000
H	-0.764374000000	1.269976000000	-6.174734000000
H	-0.115959000000	-0.383171000000	-5.940623000000

12. References:

1. (a)R. Löser, M. Chlupacova, A. Marecek, V. Opletalova and M. Gütschow, *Helv. Chim. Acta*, **2004**, *87*, 2597; (b) M. Morimoto, H. Fukumoto, T. Nozoe, A. Hagiwara and K. Komai, *J. Agric. Food Chem.* **2007**, *55*, 700.
2. Z-Q. Rong, M. Wang, C. H. E. Chow, Y. Zhao, *Chem. Eur. J.* **2016**, *22*, 9483–9487.
3. J. Xie, P. Xu, Y. Zhu, J. Wang, W-C. C. Lee, X. P. Zhang *J. Am. Chem. Soc.* **2021**, 143, 11670.
4. H. Wang, J. R. Denton, H. M. L. Davies, *Org. Lett.* **2011**, *13*, 4316–4319.
5. (Org.Lett.2017, 19, 3191−3194)
6. M. S. Manna, V. Kumar, S. Mukherjee, *Chem. Commun.* **2012**, *48*, 5193–5195.
7. K. Bera, I. N. N. Namboothiri, *Org. Biomol. Chem.* **2014**, *12*, 6425–6431.
8. F. Neese, *WIREs Comput. Mol. Sci.* **2022**, *12*, e1606.
9. Y. Zhang, W. Yang, *Phys. Rev. Lett.* **1998**, *80*, 890-890.
10. (a) S. Grimme, J. Antony, S. Hartreerlich, H. A. Krieg, *J. Chem. Phys.* **2010**, *132*, 154104; (b) S. Grimme, S. Hartreerlich, L. Goerigk, *J. Comput. Chem.* **2011**, *32*, 1456-1465.
11. F. Weigend, *Phys. Chem. Chem. Phys.* **2006**, *8*, 1057-1065.
12. (a) M. Cossi, N. Rega, G. Scalmani, V. Barone, *J. Comput. Chem.* **2003**, *24*, 669−681. (b) V. Barone, M. Cossi, *J. Phys. Chem. A* **1998**, *102*, 1995−2001.
13. C. Riplinger, P. Pinski, U. Becker, E. F. Valeev, F. Neese, *J. Chem. Phys.* **2016**, *144*, 024109.
14. (a) T. Gatzenmeier, M. Turberg, D. Yepes, Y. Xie, F. Neese, G. Bistoni, B. List, *J. Am. Chem. Soc.* **2018**, *140*, 12671−12676; (b) C. Zhu, F. Mandrelli, H. Zhou, R. Maji, B. List, *J. Am. Chem. Soc.* **2021**, *143*, 3312−3317; (c) I. Harden, F. Neese, G. Bistoni, *Chem. Sci.* **2022**, *13*, 8848-8859; (d) G. G. Gerosa, S. A. Schwengers, R. Maji, C. K. De, B. List, *Angew. Chem. Int. Ed.* **2020**, *59*, 20485 –20488.
15. L. Falivene, Z. Cao, A. Petta, L. Serra, A. Poater, R. Oliva, V. Scarano, L. Cavallo, *Nat. Chem.* **2019**, *11*, 872–879.
16. T. Lu, F. Chen, *J. Comput. Chem.* **2012**, *33*, 580–592.
17. (a) F. M. Bickelhaupt, *J. Comput. Chem.* **1999**, *20*, 114 –128. (b) W. J. van Zeist, F. M. Bickelhaupt, *Org. Biomol. Chem.* **2010**, *8*, 3118-3127.
18. (a) J. Ho, M. L. A. Coote, *Theor. Chem. Acc.* **2009**, 125, 3–21; (b) J. A. Keith, K. A. Grice, C. P. Kubiak, E. A. Carter, *J. Am. Chem. Soc.* **2013**, 135, 15823−15829; (c) J. A. Keith, E. A. Carter, *J. Am. Chem. Soc.* **2012**, 134, 7580–7583.

13. NMR spectra and HPLC chromatograms:

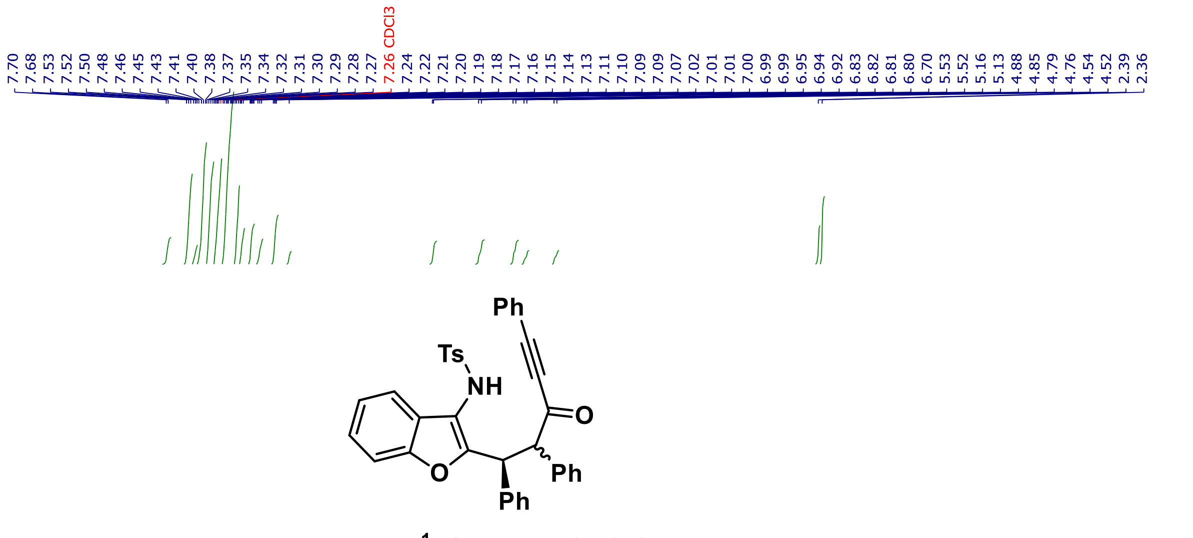

A, 1:7 dr ^{1}H(500MHz, $CDCl_3$)

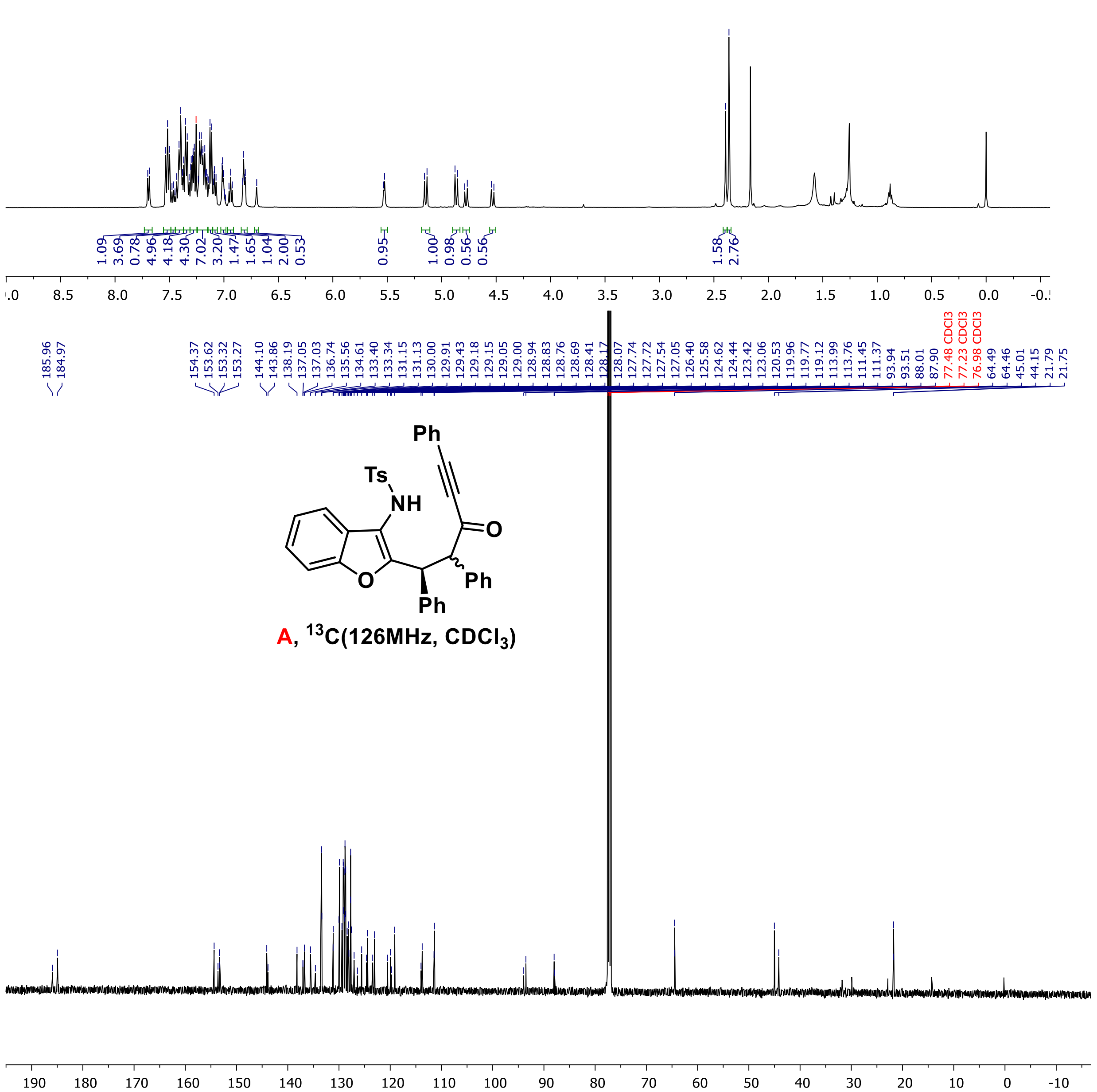

A, ^{13}C(126MHz, $CDCl_3$)

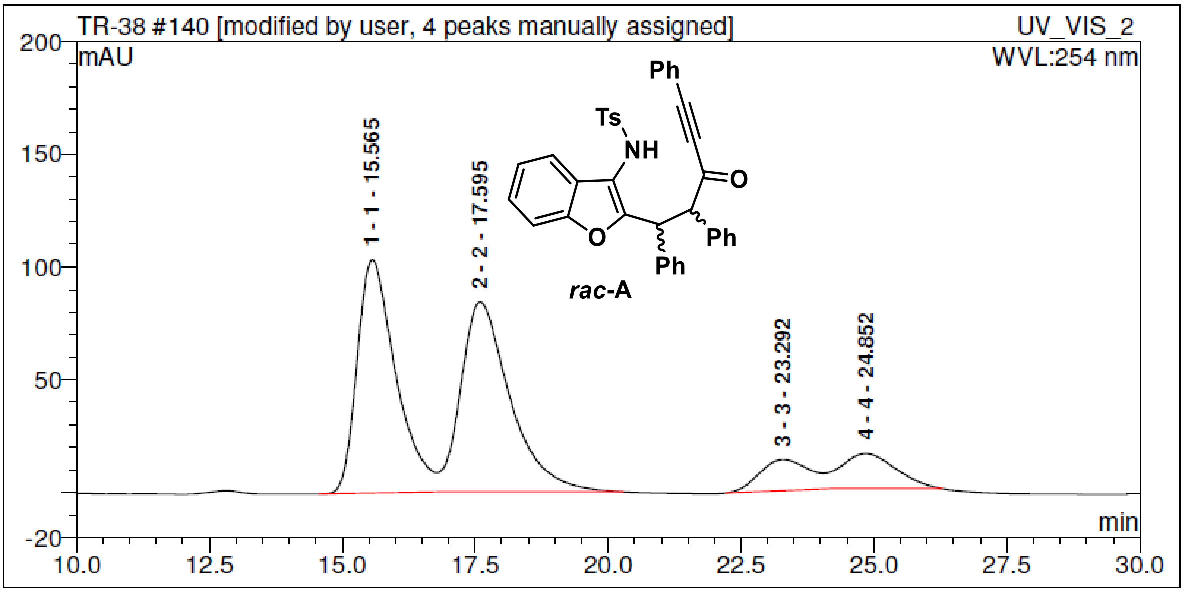

Peak Name	Ret.Time (detected) min	Area mAU*min	Rel.Area(ident.) %	Height mAU	Amount
1 1	15.57	88.15827	41.49711319	103.3562	n.a.
2 2	17.60	90.30376	42.50702045	84.13357	n.a.
3 3	23.29	16.16849	7.610694519	13.71956	n.a.
4 4	24.85	17.814	8.385171843	15.455	n.a.

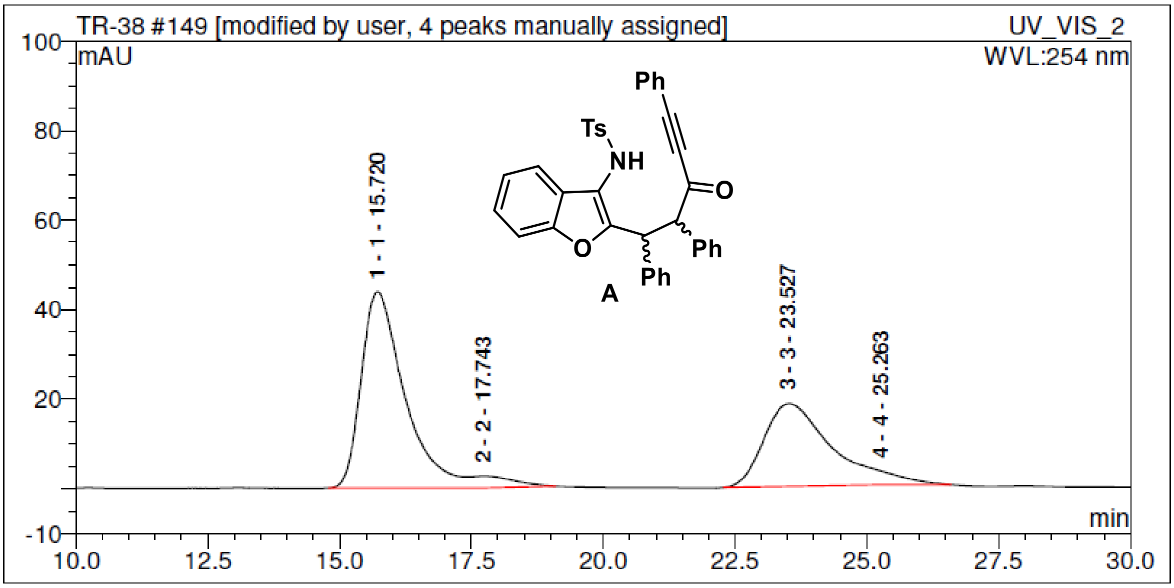

Peak Name	Ret.Time (detected) min	Area mAU*min	Rel.Area(ident.) %	Height mAU	Amount
1 1	15.72	42.09199	58.55196375	43.7907	n.a.
2 2	17.74	1.870579	2.602064032	2.63749	n.a.
3 3	23.53	26.31776	36.60925896	18.46781	n.a.
4 4	25.26	1.608	2.236713253	3.090	n.a.

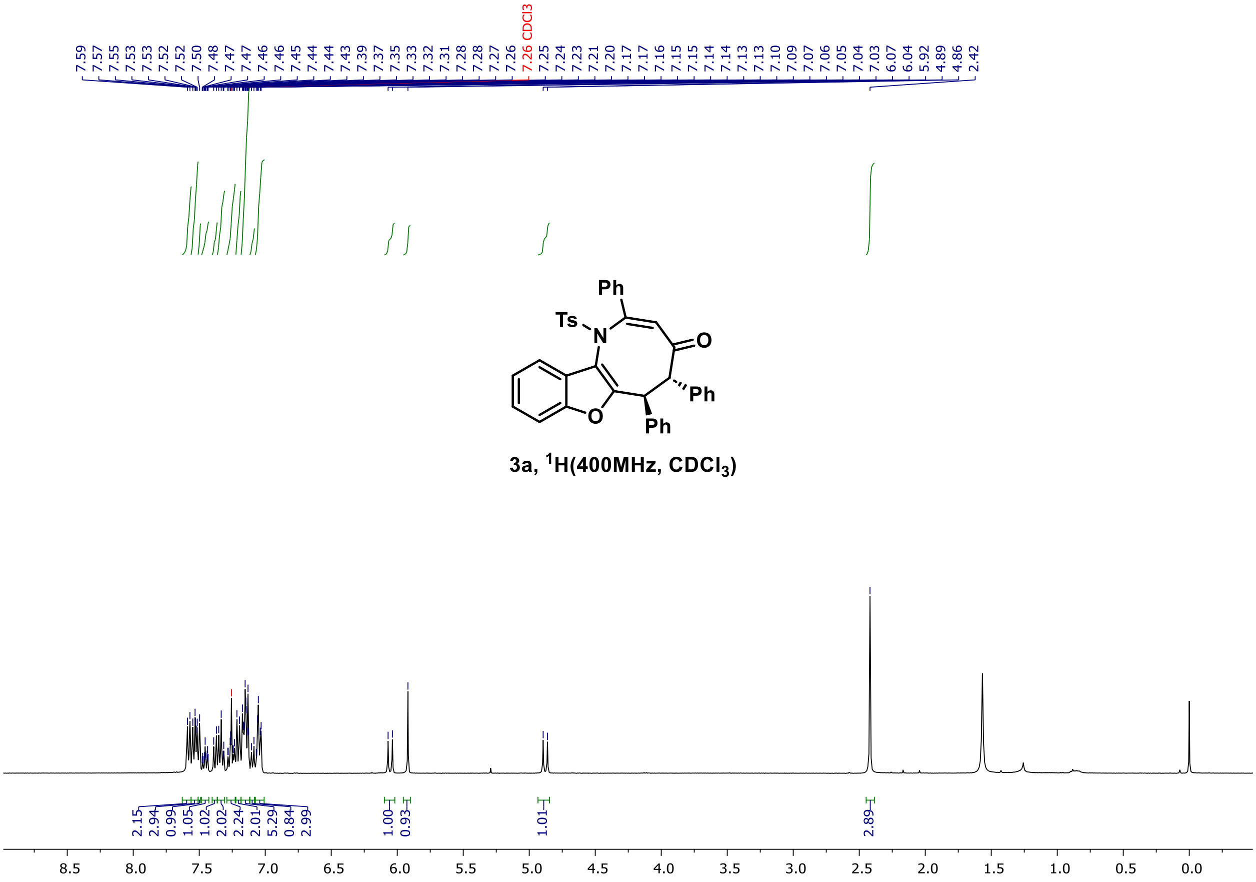

3a, ^{1}H(400MHz, $CDCl_3$)

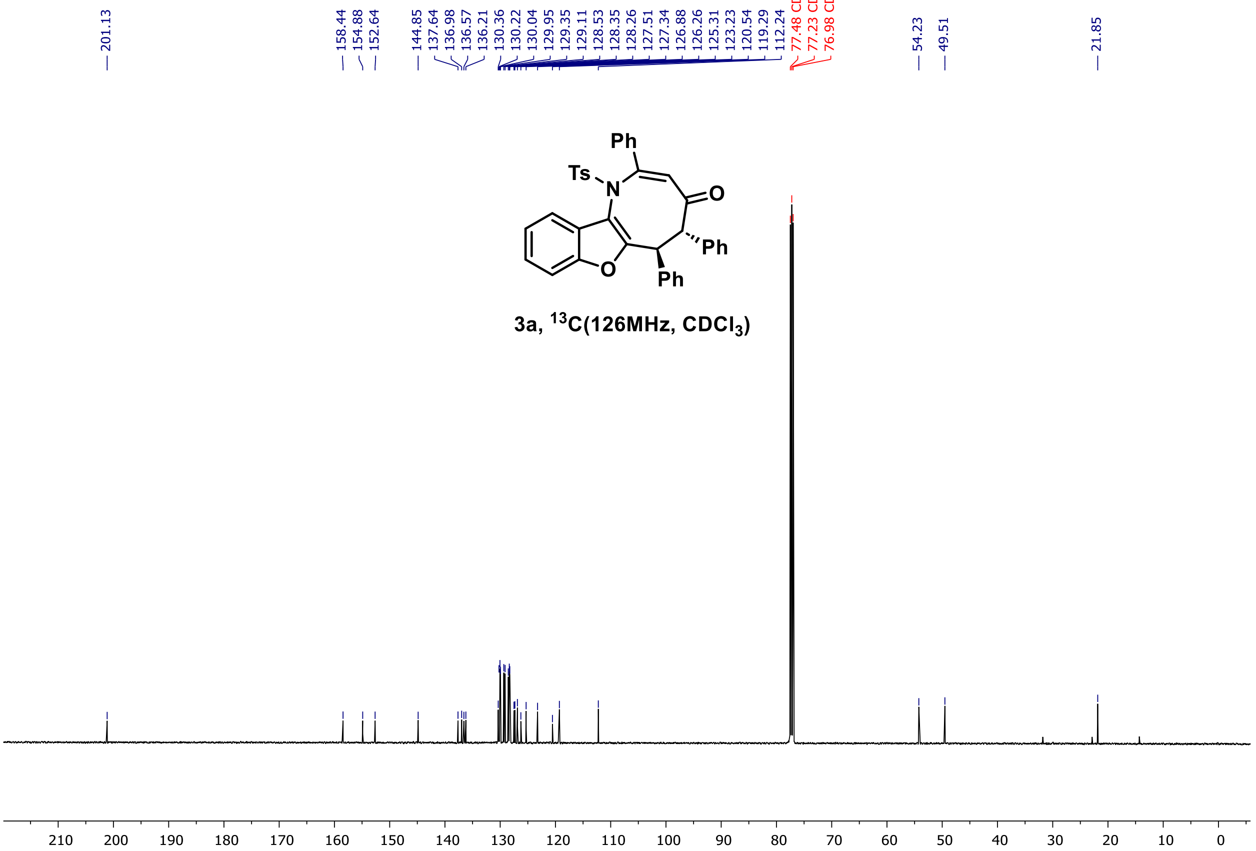

3a, ^{13}C(126MHz, $CDCl_3$)

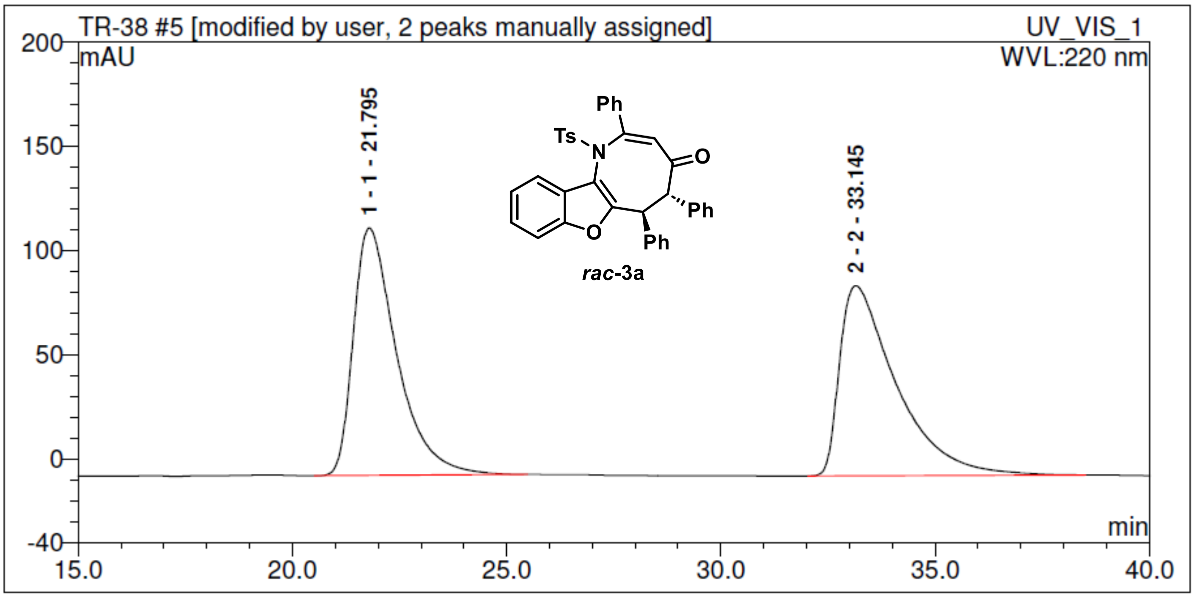

	Peak Name	Ret.Time (detected) min	Area mAU*min	Rel.Area(ident.) %	Height mAU	Amount
1	1	21.80	138.8226	49.83190877	118.6878	n.a.
2	2	33.15	139.759	50.16809123	91.203	n.a.

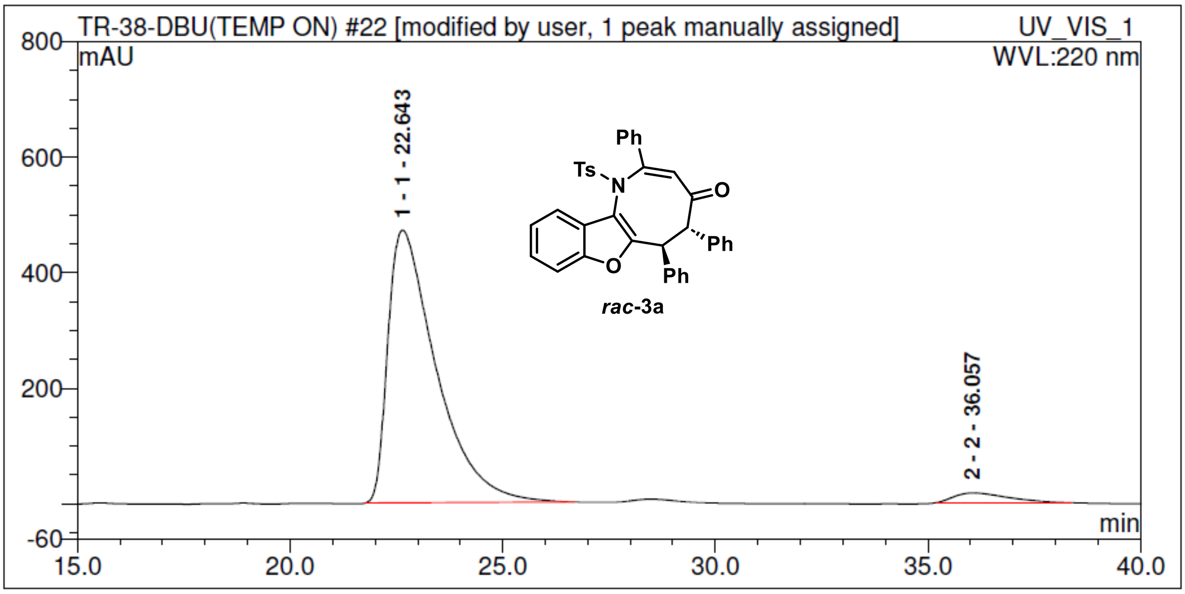

	Peak Name	Ret.Time (detected) min	Area mAU*min	Rel.Area(ident.) %	Height mAU	Amount
1	1	22.64	620.7385	96.14471419	471.9738	n.a.
2	2	36.06	24.891	3.855285815	17.045	n.a.

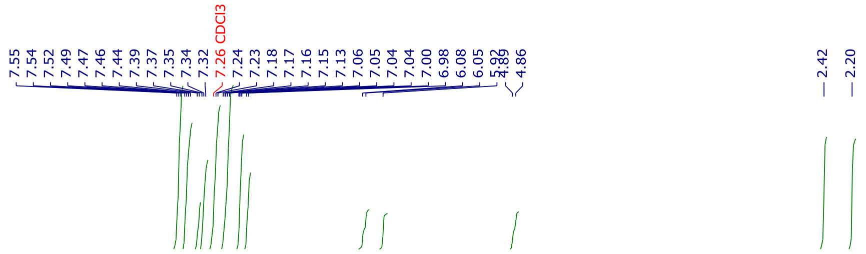

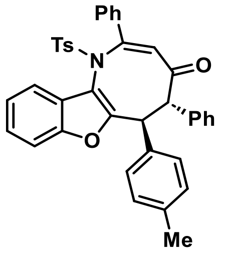

3b, ^{1}H(500MHz, CDCl$_3$)

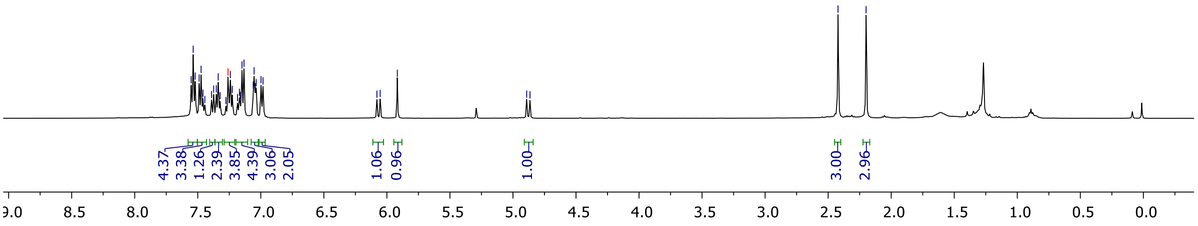

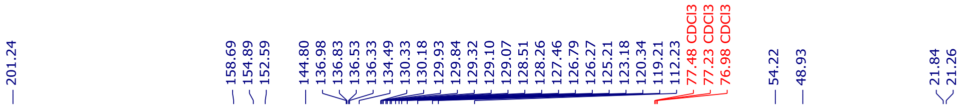

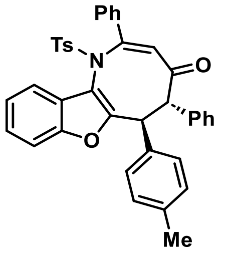

3b, ^{13}C(126MHz, CDCl$_3$)

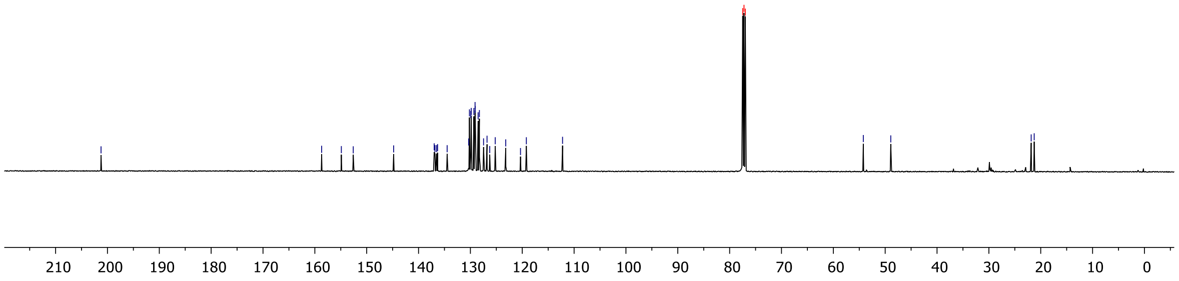

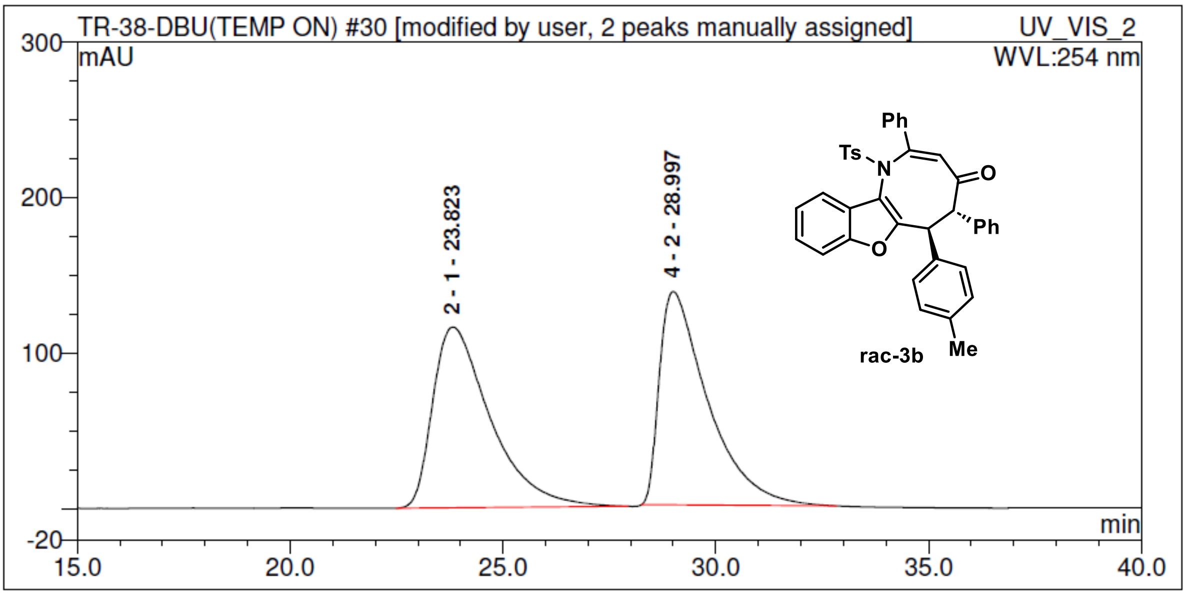

	Peak Name	Ret.Time (detected) min	Area mAU*min	Rel.Area(ident.) %	Height mAU	Amount
2	1	23.82	182.4996	50.22228492	116.2221	n.a.
4	2	29.00	180.884	49.77771508	137.199	n.a.

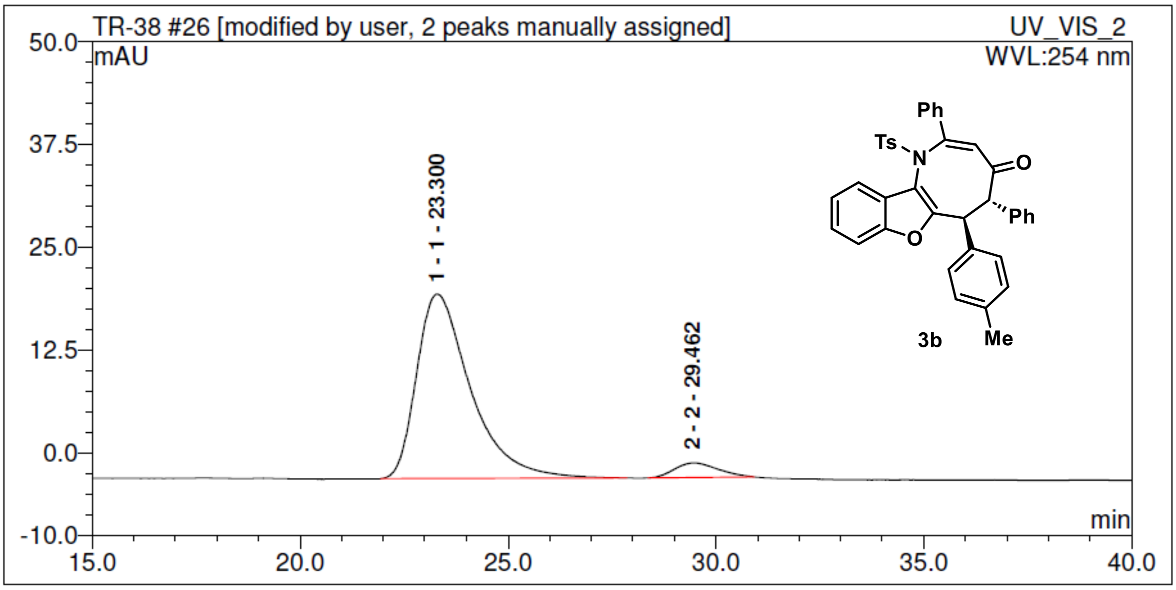

	Peak Name	Ret.Time (detected) min	Area mAU*min	Rel.Area(ident.) %	Height mAU	Amount
1	1	23.30	33.74022	94.03168428	22.4313	n.a.
2	2	29.46	2.142	5.968315718	1.770	n.a.

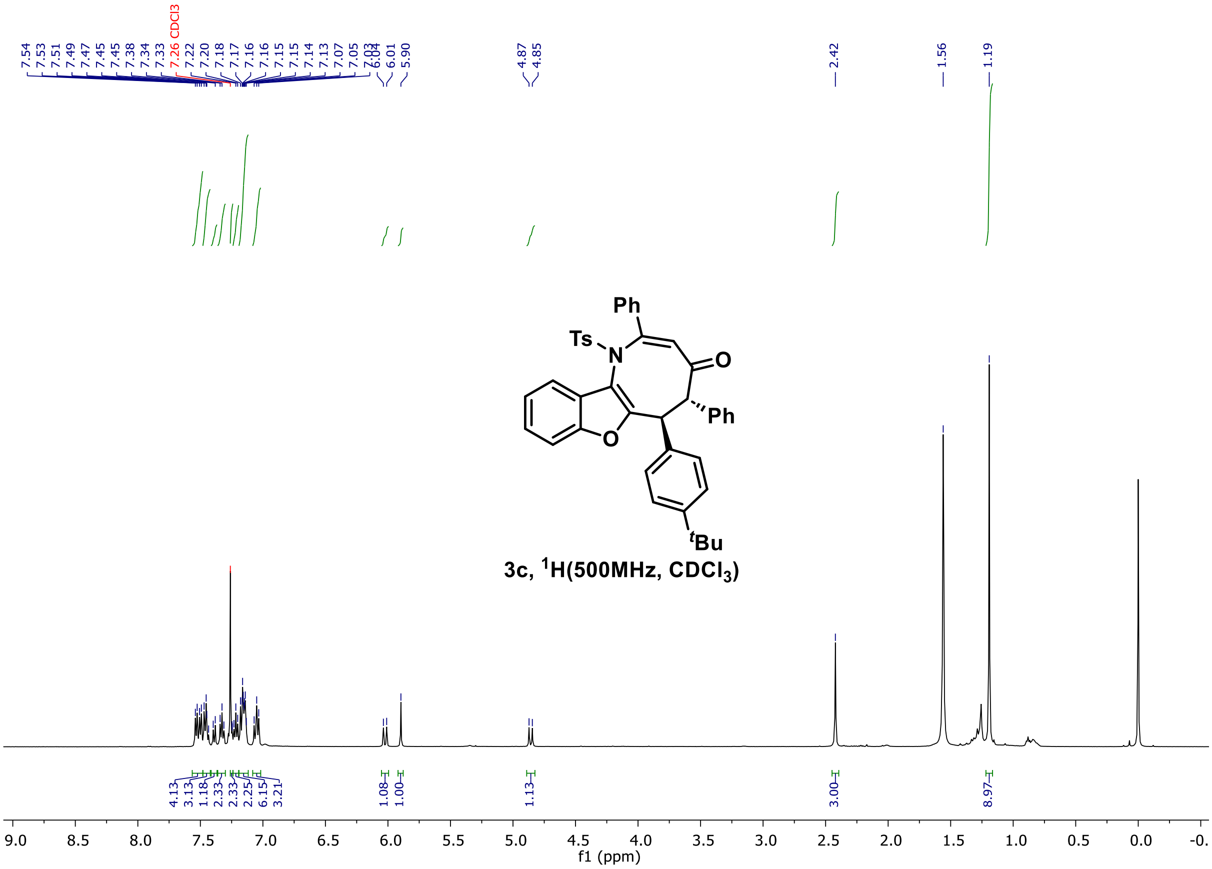
7.54 7.53 7.51 7.49 7.47 7.45 7.45 7.38 7.34 7.33 7.26 CDCl3 7.22 7.20 7.18 7.17 7.16 7.16 7.15 7.15 7.14 7.13 7.07 7.05 7.03 6.04 6.01 5.90
4.87 4.85
2.42
1.56
1.19
Ph
Ts
N
O
Ph
O
tBu
3c, 1H(500MHz, CDCl3)
4.13 3.13 1.18 2.33 2.33 2.25 6.15 3.21
1.08 1.00
1.13
3.00
8.97
9.0 8.5 8.0 7.5 7.0 6.5 6.0 5.5 5.0 4.5 4.0 3.5 3.0 2.5 2.0 1.5 1.0 0.5 0.0 -0.
f1 (ppm)

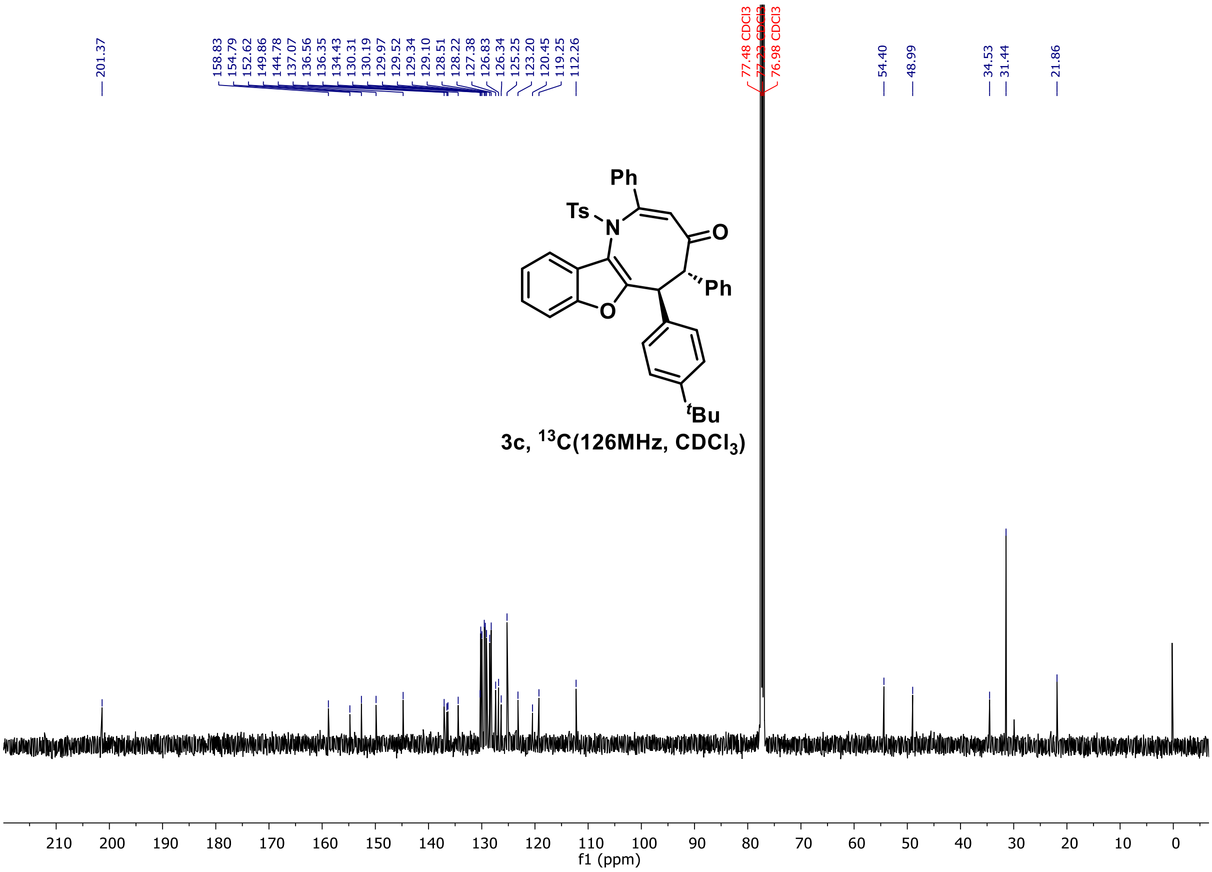
201.37
158.83 154.79 152.62 149.86 144.78 137.07 136.56 136.35 134.43 130.31 130.19 129.97 129.52 129.34 129.10 128.51 128.22 127.38 126.83 126.34 125.25 123.20 120.45 119.25 112.26
77.48 CDCl3 77.23 CDCl3 76.98 CDCl3
54.40
48.99
34.53
31.44
21.86
Ph
Ts
N
O
Ph
O
tBu
3c, 13C(126MHz, CDCl3)
210 200 190 180 170 160 150 140 130 120 110 100 90 80 70 60 50 40 30 20 10 0
f1 (ppm)

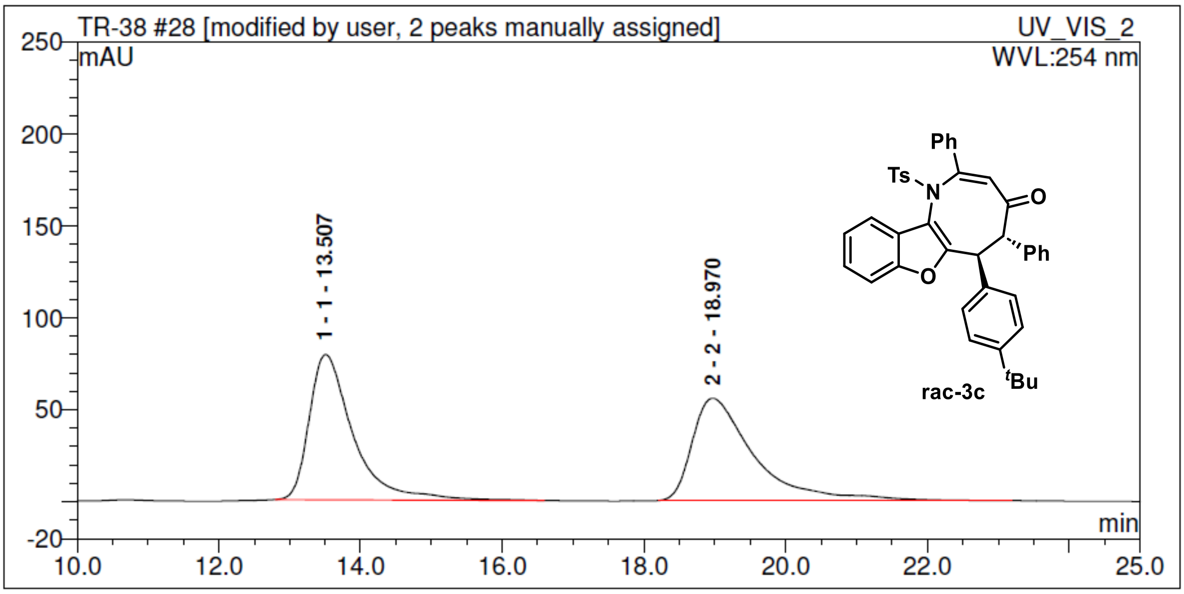

Peak Name		Ret.Time (detected) min	Area mAU*min	Rel.Area(ident.) %	Height mAU	Amount
1	1	13.51	56.70619	50.21380361	78.9684	n.a.
2	2	18.97	56.223	49.78619639	55.645	n.a.

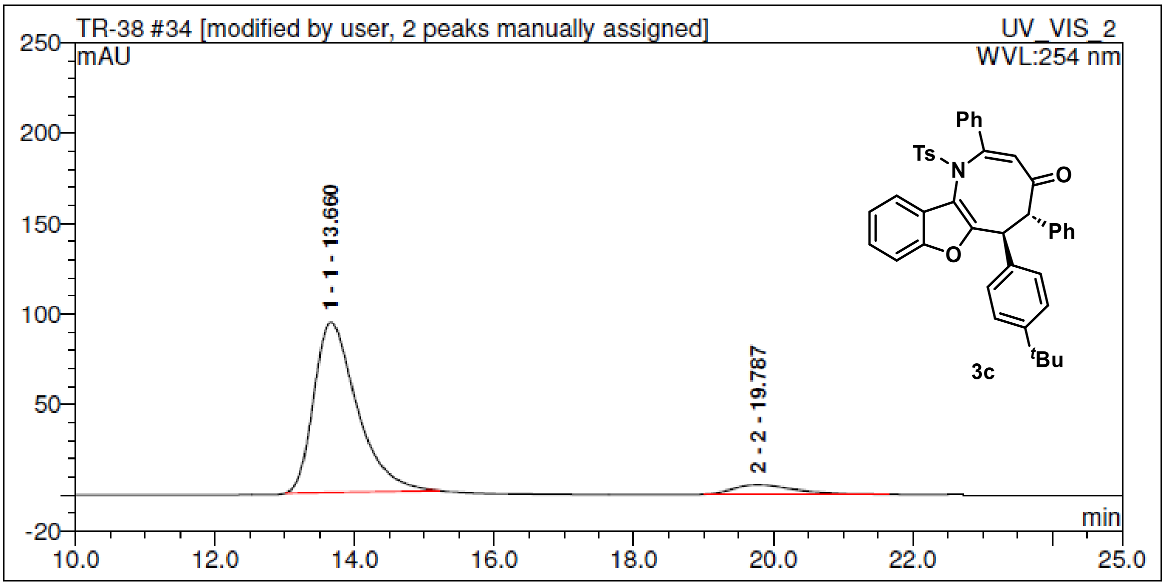

Peak Name		Ret.Time (detected) min	Area mAU*min	Rel.Area(ident.) %	Height mAU	Amount
1	1	13.66	67.37451	92.63017039	93.85835	n.a.
2	2	19.79	5.360	7.369829606	5.230	n.a.

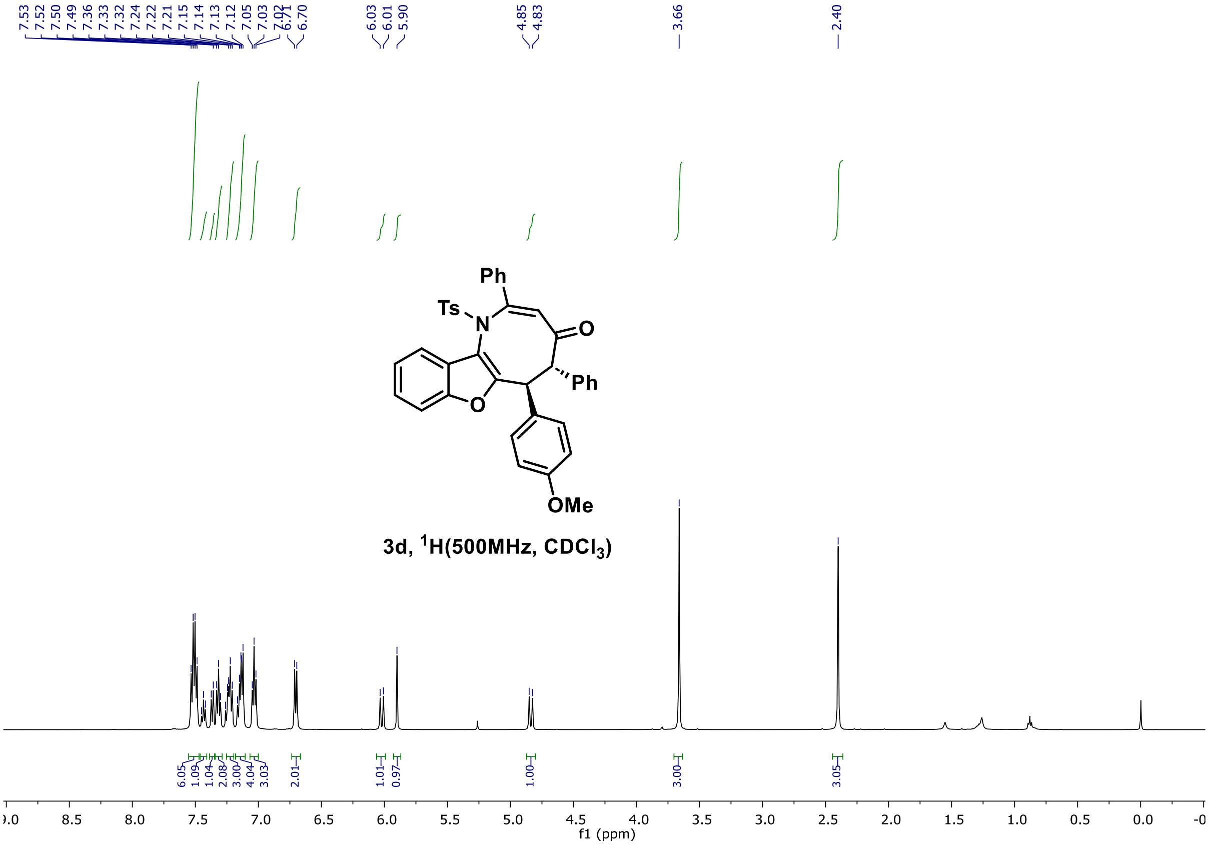

3d, ^{1}H(500MHz, $CDCl_3$)

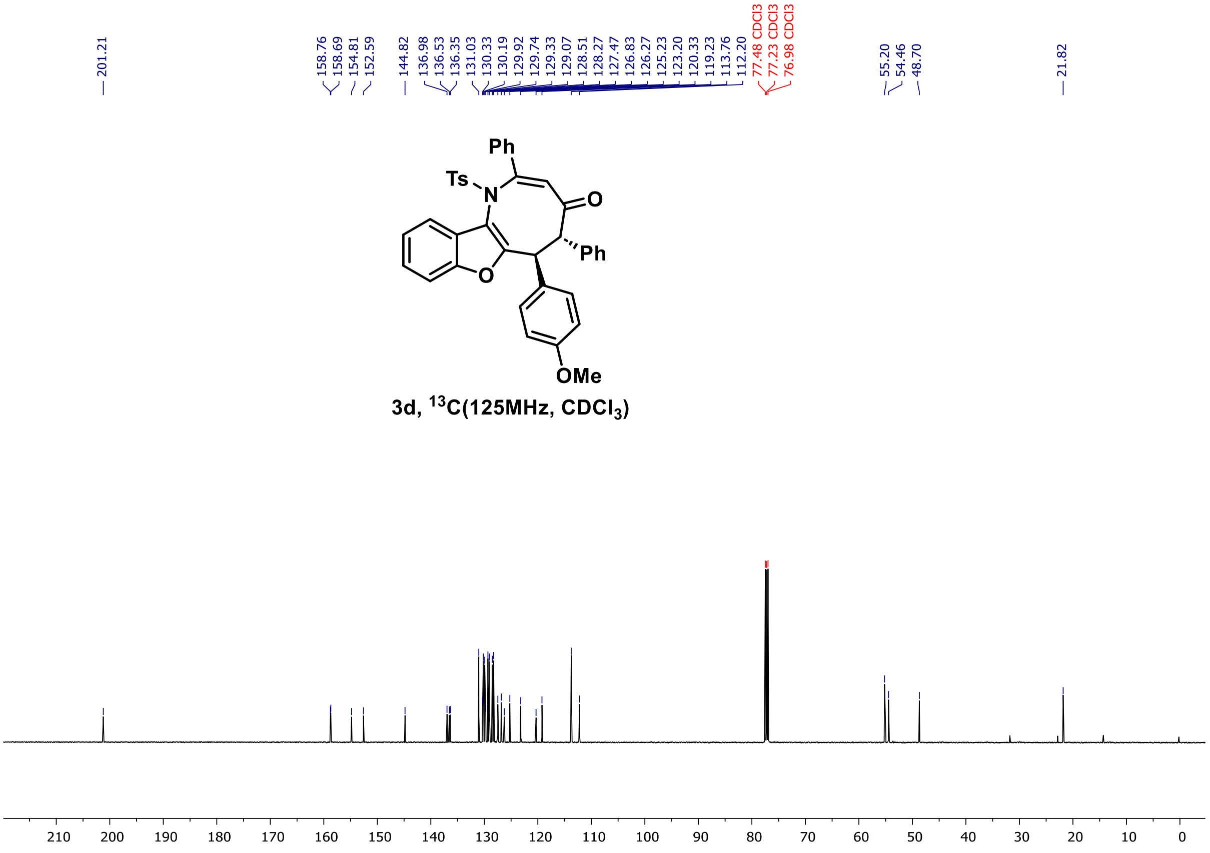

3d, ^{13}C(125MHz, $CDCl_3$)

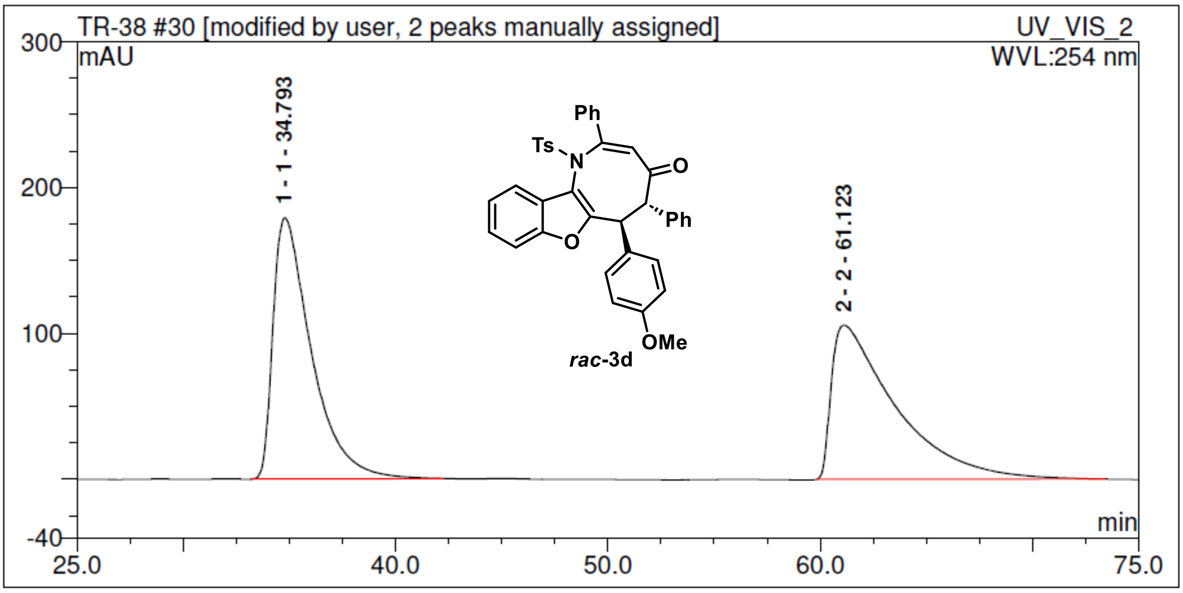

Peak Name	Ret.Time (detected)	Area	Rel.Area(ident.)	Height	Amount
	min	mAU*min	%	mAU	
1 1	34.79	374.3632	50.3459118	179.1022	n.a.
2 2	61.12	369.219	49.6540882	105.626	n.a.

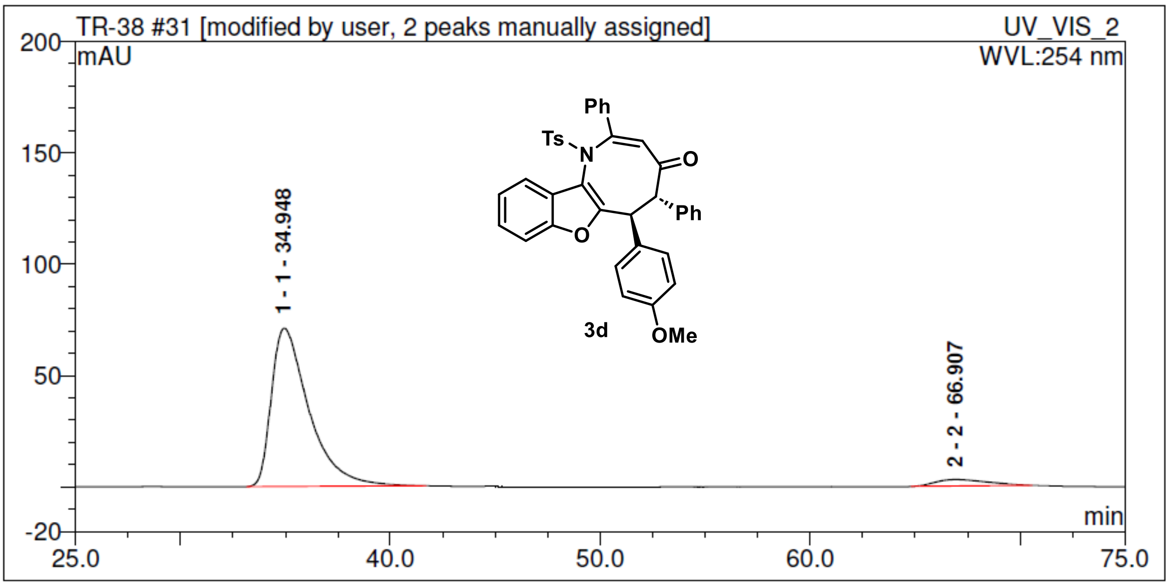

Peak Name	Ret.Time (detected)	Area	Rel.Area(ident.)	Height	Amount
	min	mAU*min	%	mAU	
1 1	34.95	147.4666	94.64957633	70.97561	n.a.
2 2	66.91	8.336	5.350423668	2.935	n.a.

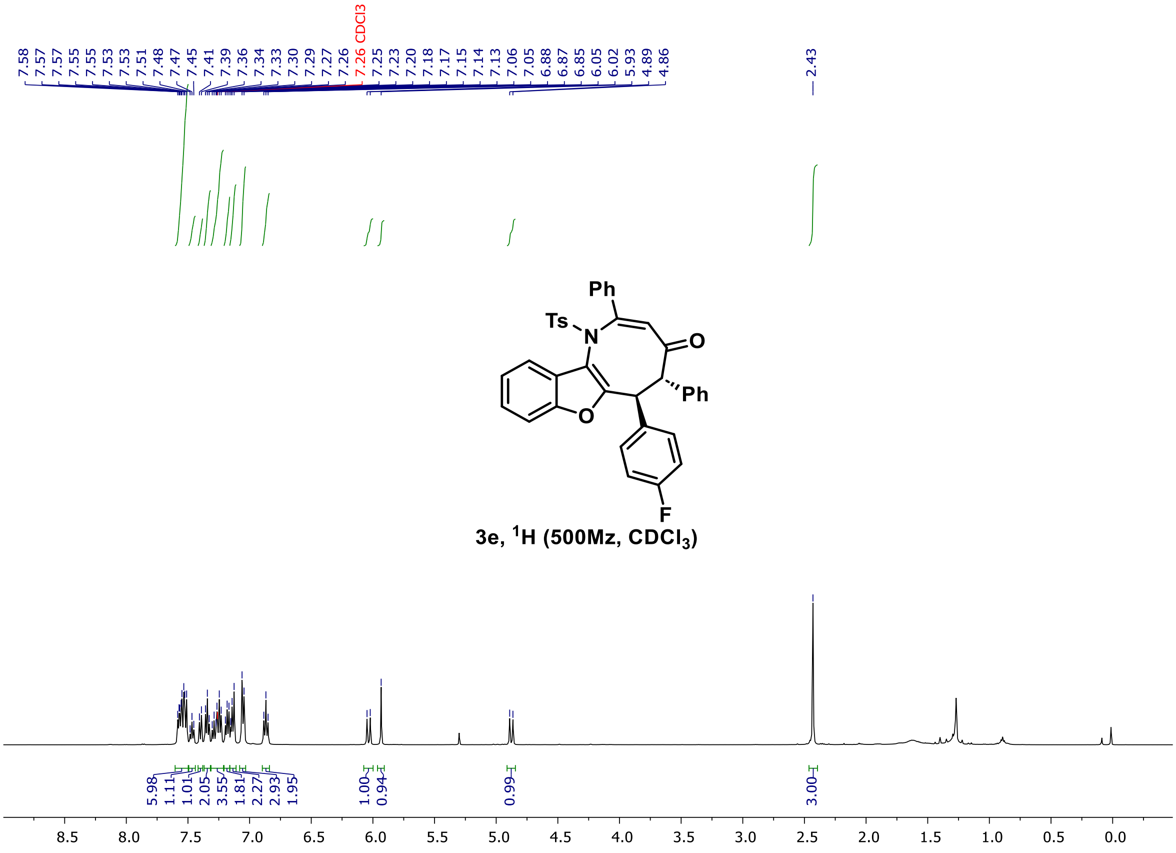

3e, ^{1}H (500Mz, $CDCl_3$)

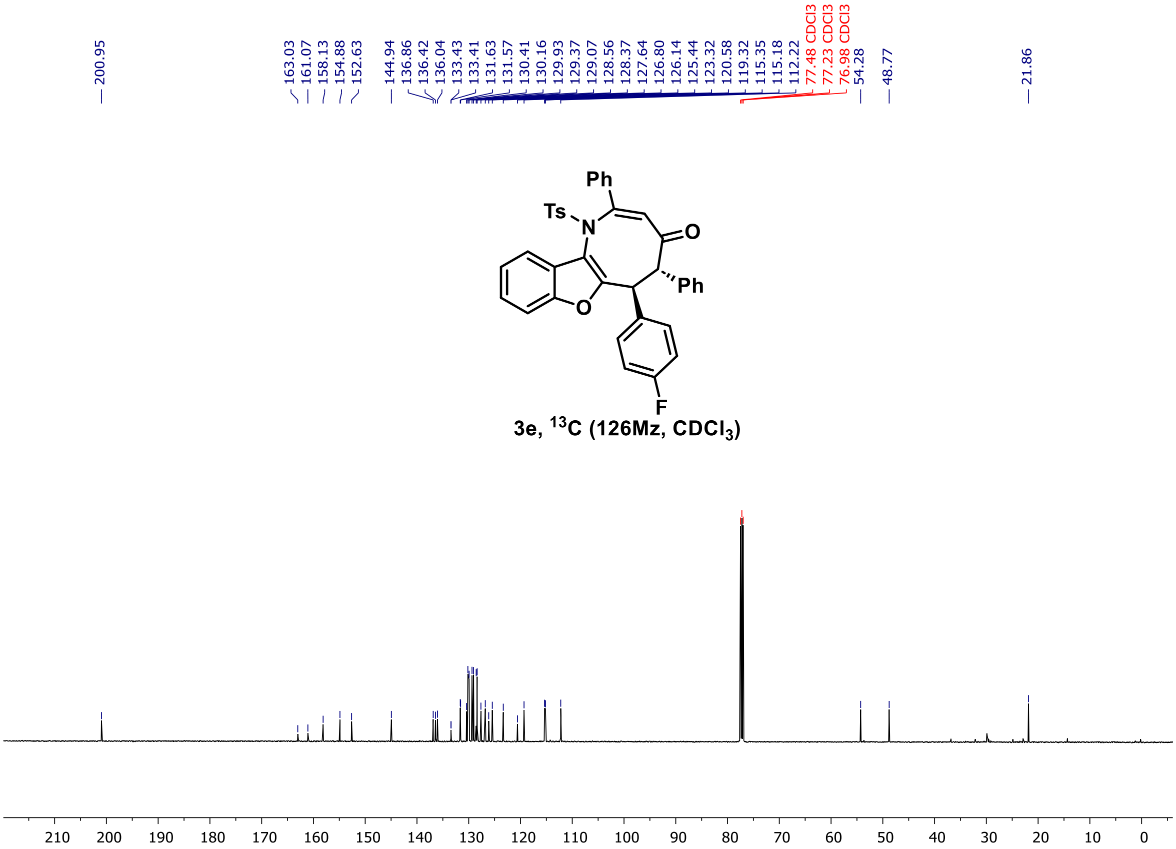

3e, ^{13}C (126Mz, $CDCl_3$)

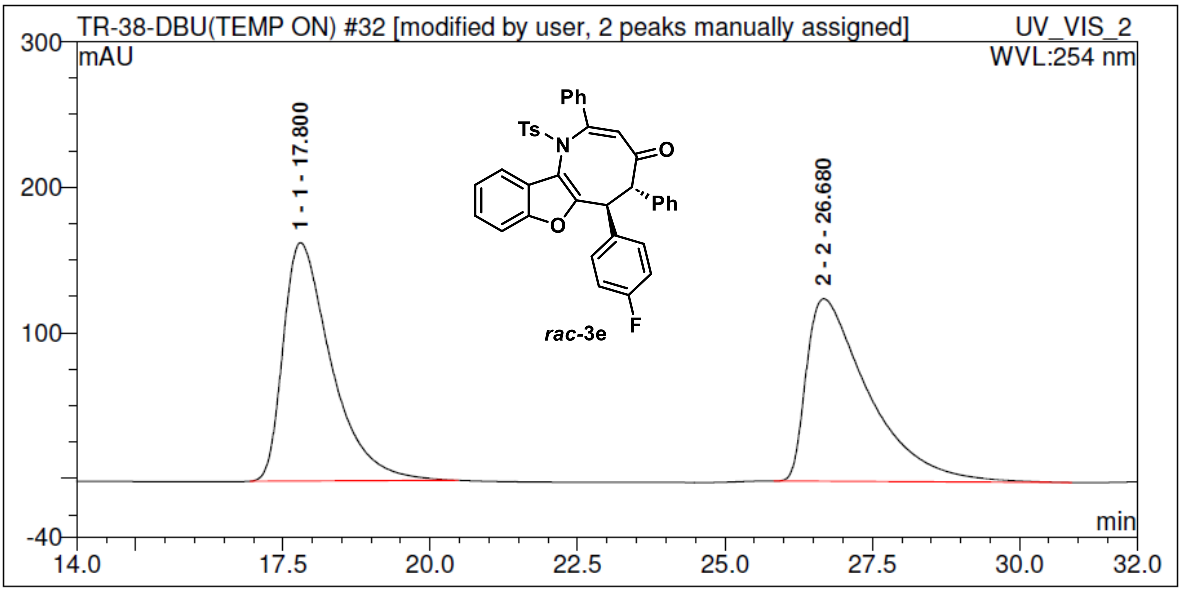

	Peak Name	Ret.Time (detected) min	Area mAU*min	Rel.Area(ident.) %	Height mAU	Amount
1	1	17.80	154.9426	50.18496531	163.6066	n.a.
2	2	26.68	153.800	49.81503469	125.327	n.a.

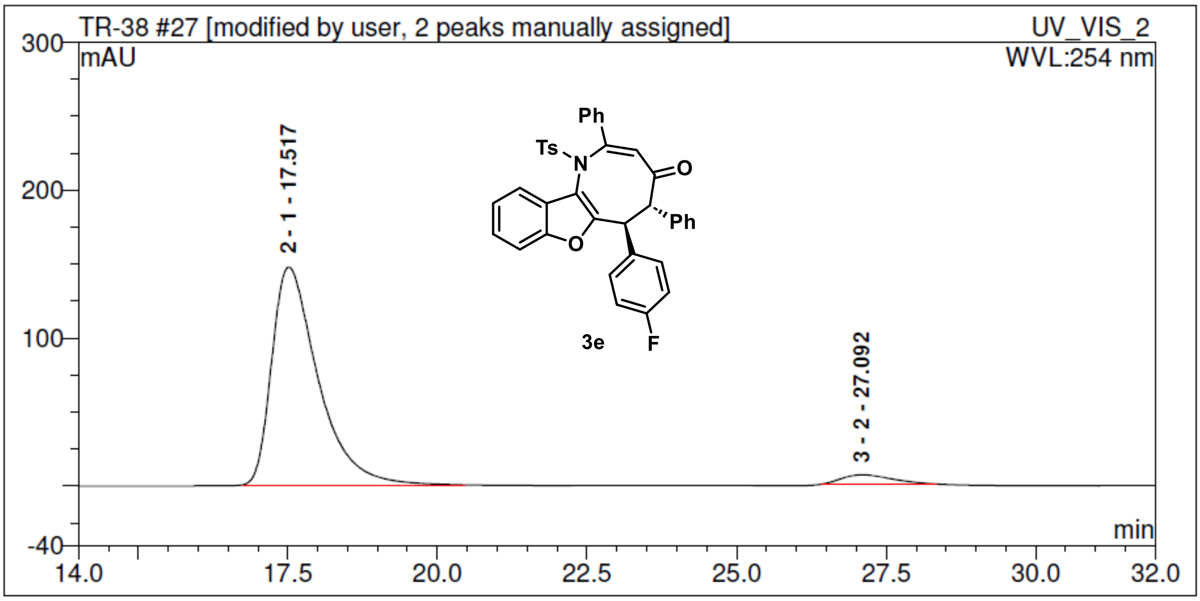

	Peak Name	Ret.Time (detected) min	Area mAU*min	Rel.Area(ident.) %	Height mAU	Amount
2	1	17.52	132.0255	95.53904341	147.3565	n.a.
3	2	27.09	6.165	4.460956592	6.295	n.a.

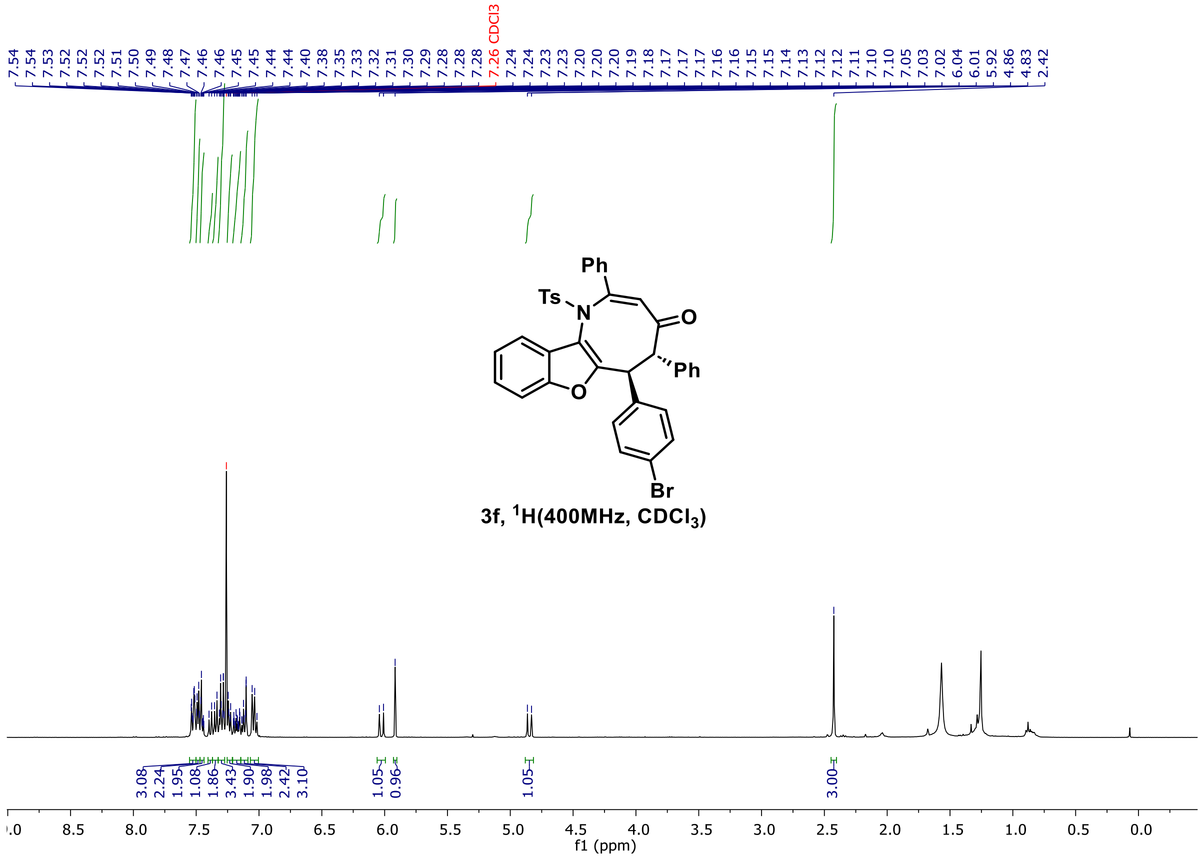

3f, ^{1}H(400MHz, $CDCl_3$)

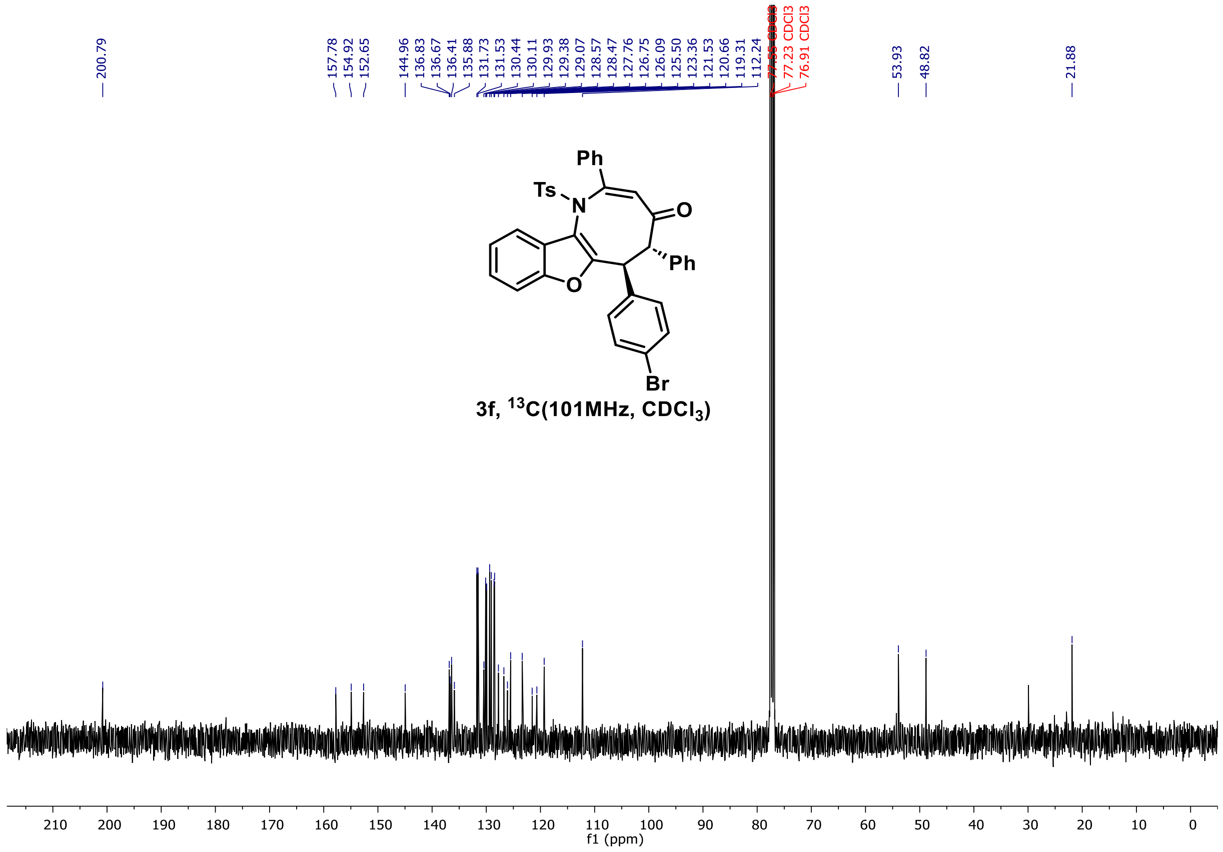

3f, ^{13}C(101MHz, $CDCl_3$)

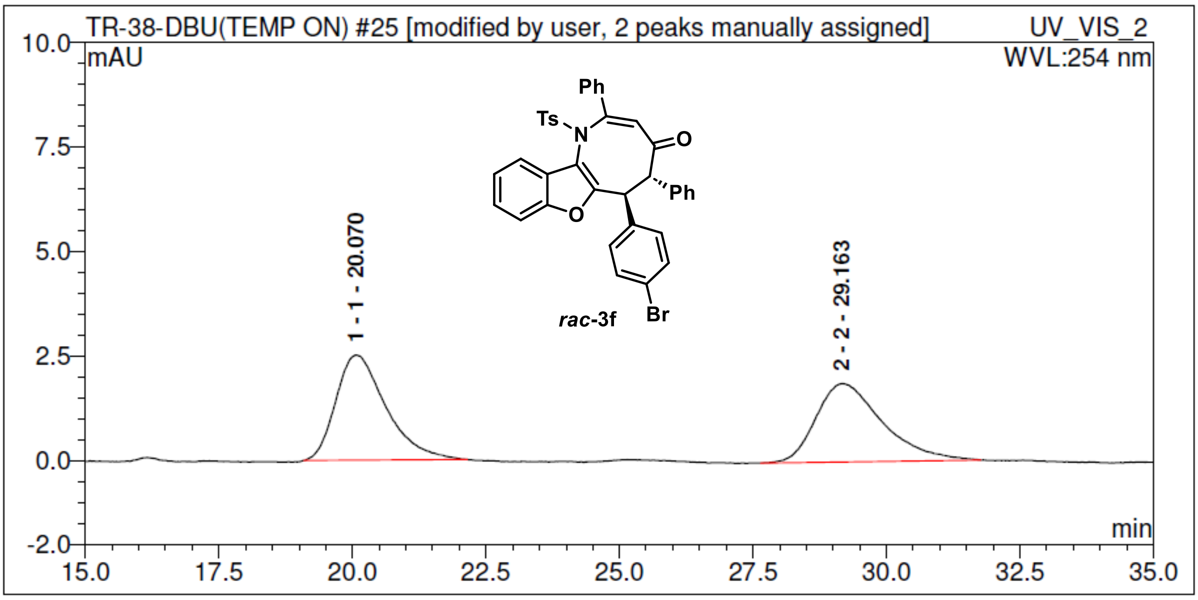

	Peak Name	Ret.Time (detected) min	Area mAU*min	Rel.Area(ident.) %	Height mAU	Amount
1	1	20.07	2.738028	50.36411161	2.51326	n.a.
2	2	29.16	2.698	49.63588839	1.875	n.a.

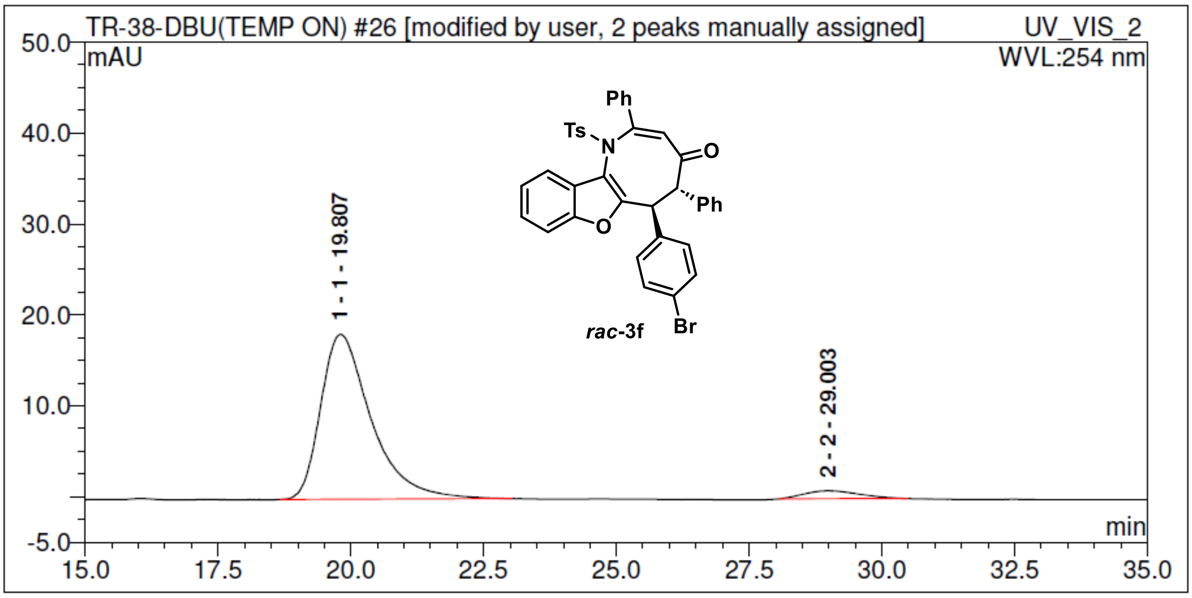

	Peak Name	Ret.Time (detected) min	Area mAU*min	Rel.Area(ident.) %	Height mAU	Amount
1	1	19.81	20.04272	95.04408312	18.13922	n.a.
2	2	29.00	1.045	4.955916877	0.863	n.a.

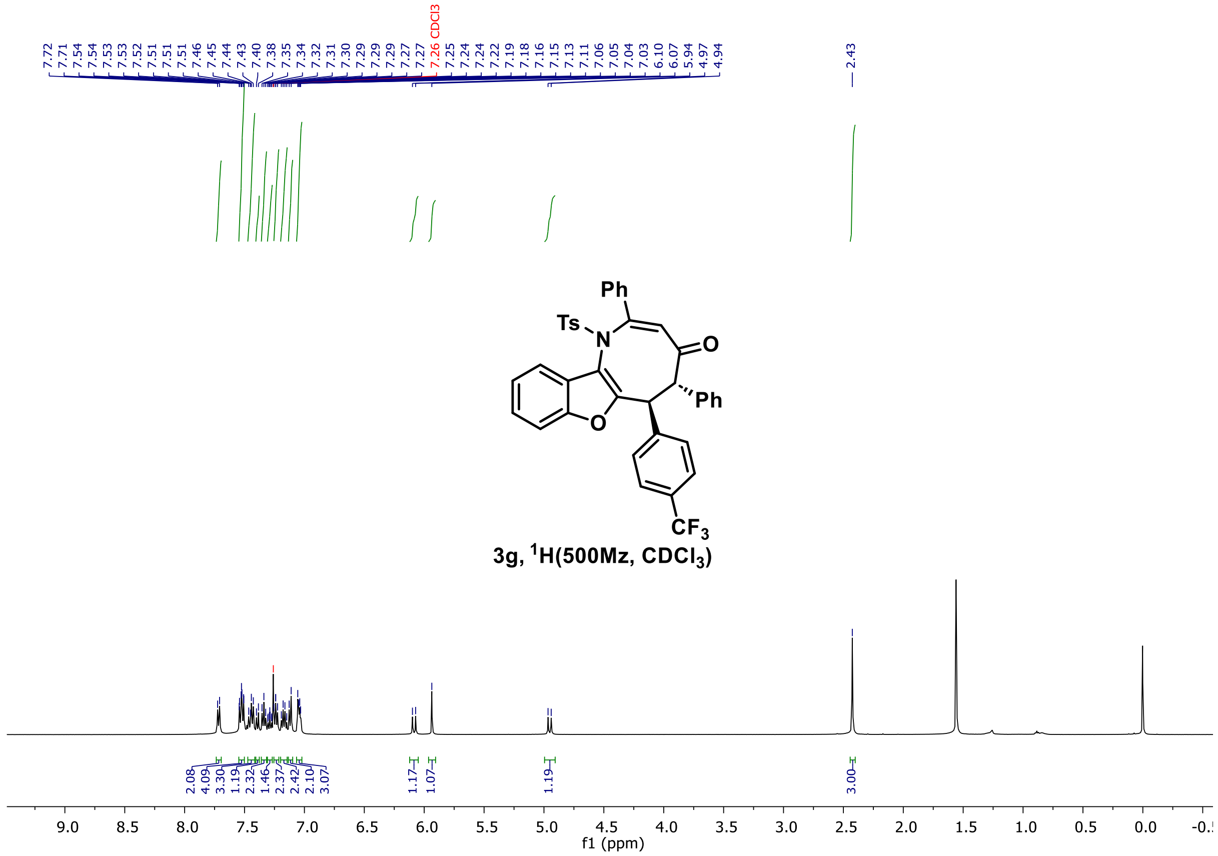

3g, ^{1}H(500Mz, $CDCl_3$)

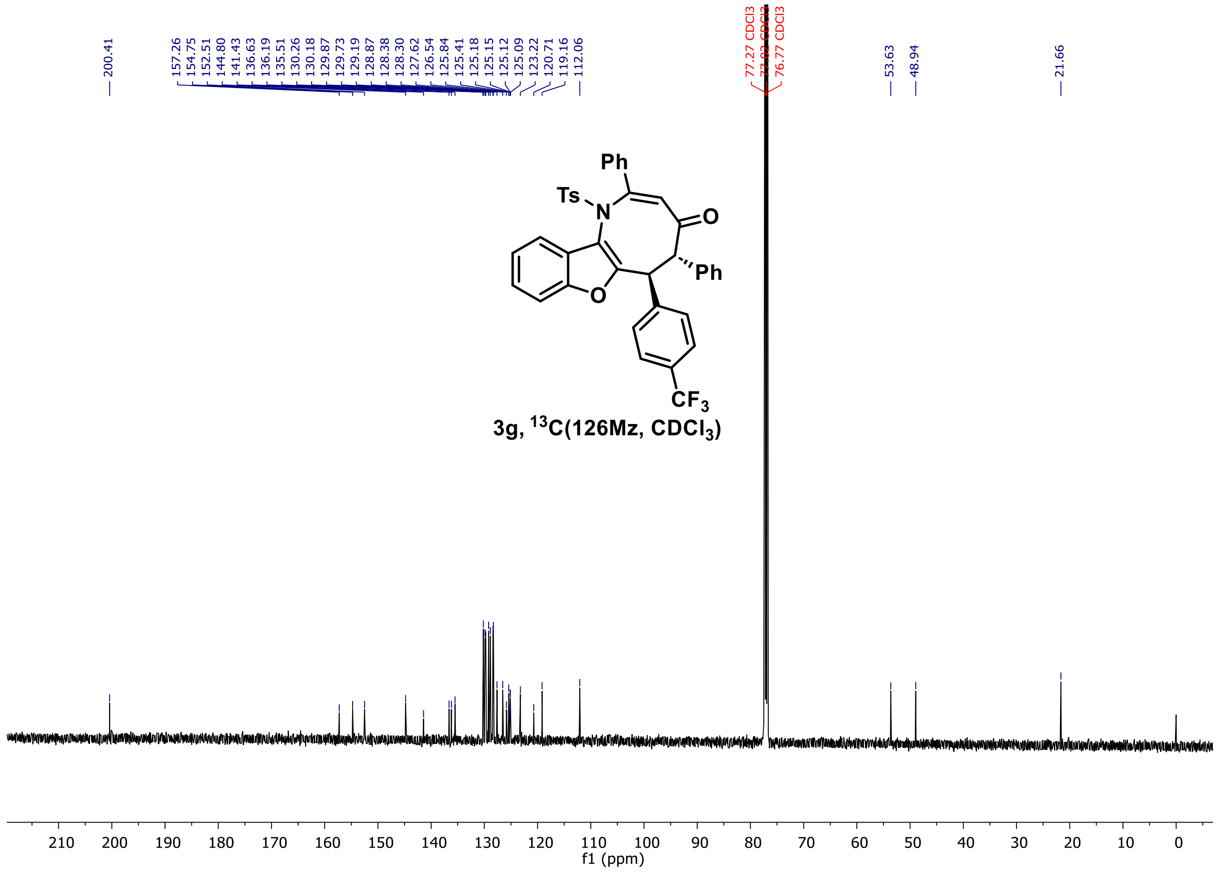

3g, ^{13}C(126Mz, $CDCl_3$)

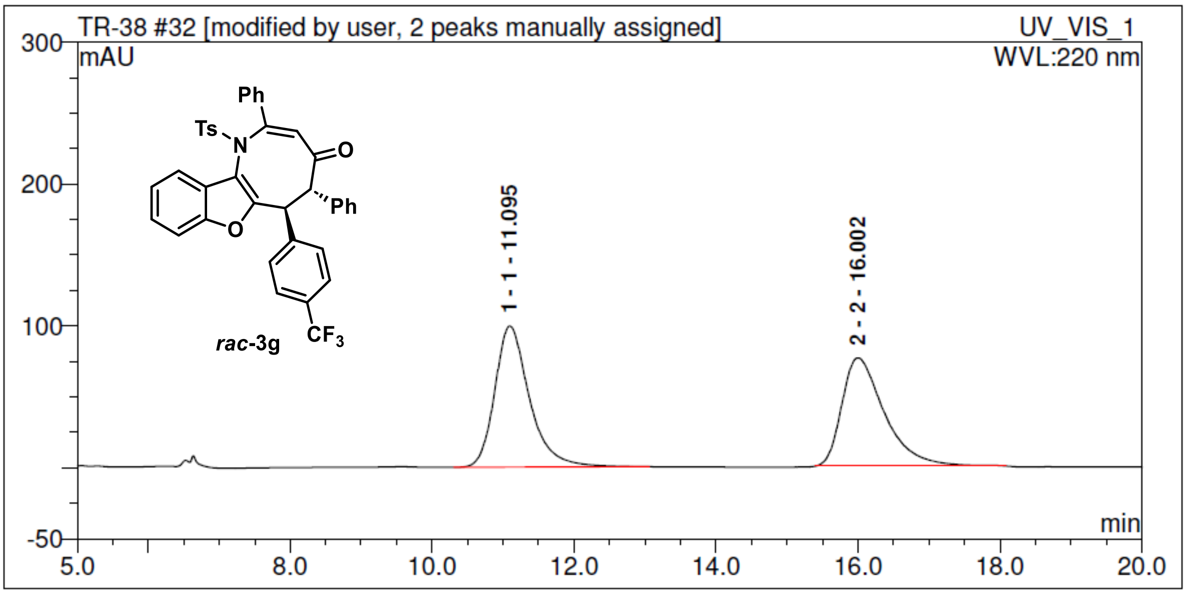

	Peak Name	Ret.Time (detected) min	Area mAU*min	Rel.Area(ident.) %	Height mAU	Amount
1	1	11.10	56.5721	51.65807812	99.50785	n.a.
2	2	16.00	52.940	48.34192188	76.216	n.a.

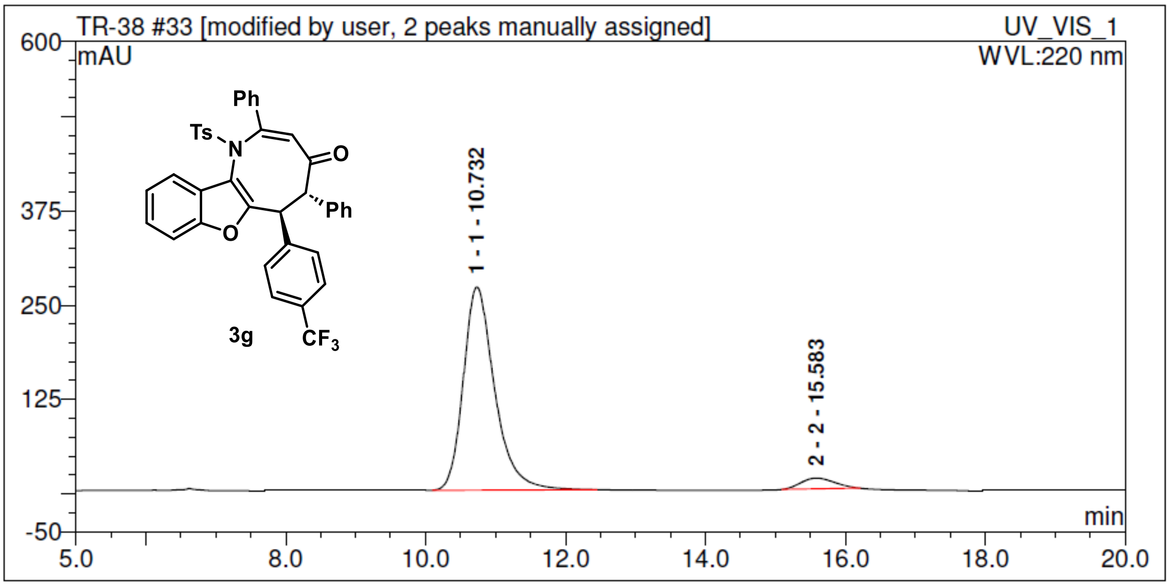

	Peak Name	Ret.Time (detected) min	Area mAU*min	Rel.Area(ident.) %	Height mAU	Amount
1	1	10.73	139.9703	94.60437186	269.1159	n.a.
2	2	15.58	7.983	5.395628145	14.332	n.a.

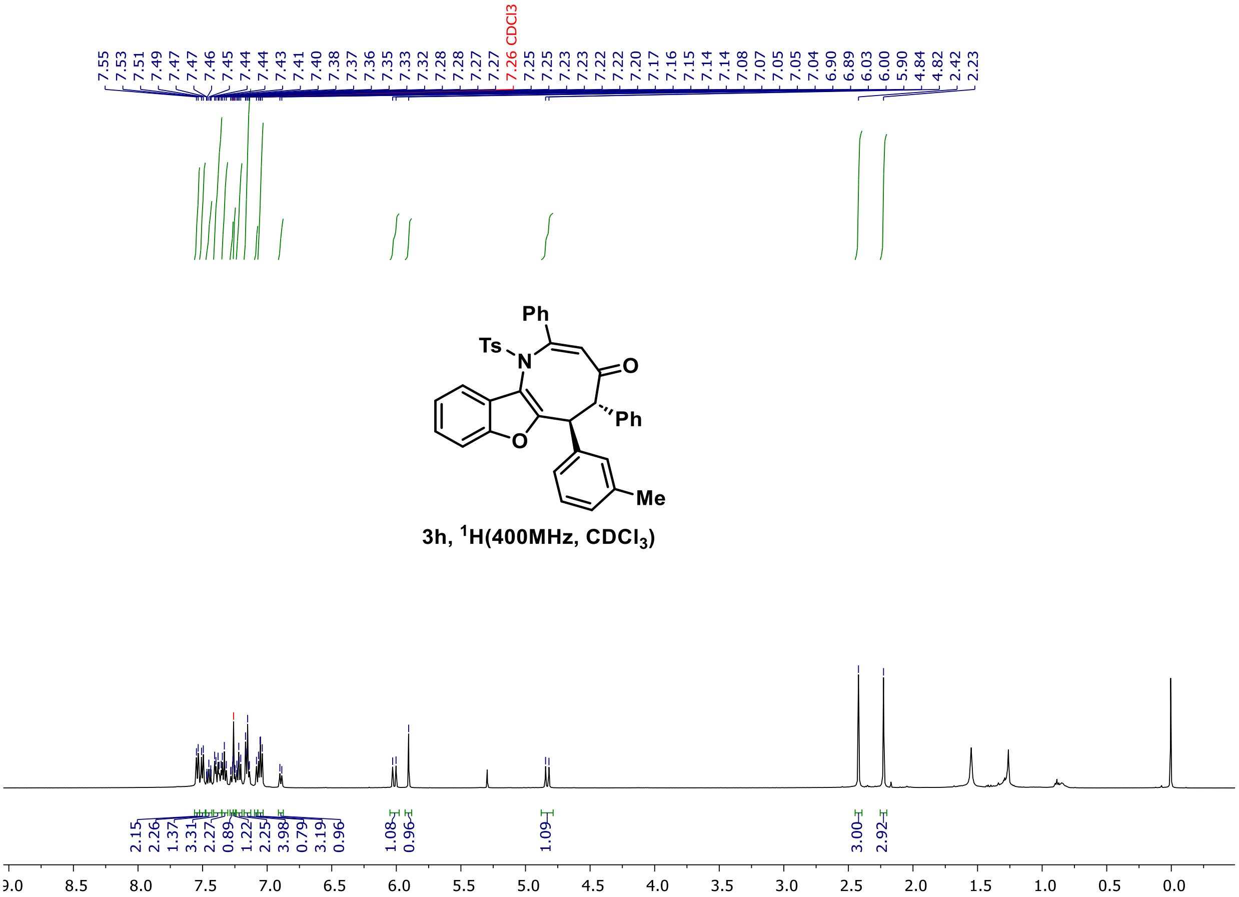

3h, ^1H(400MHz, $CDCl_3$)

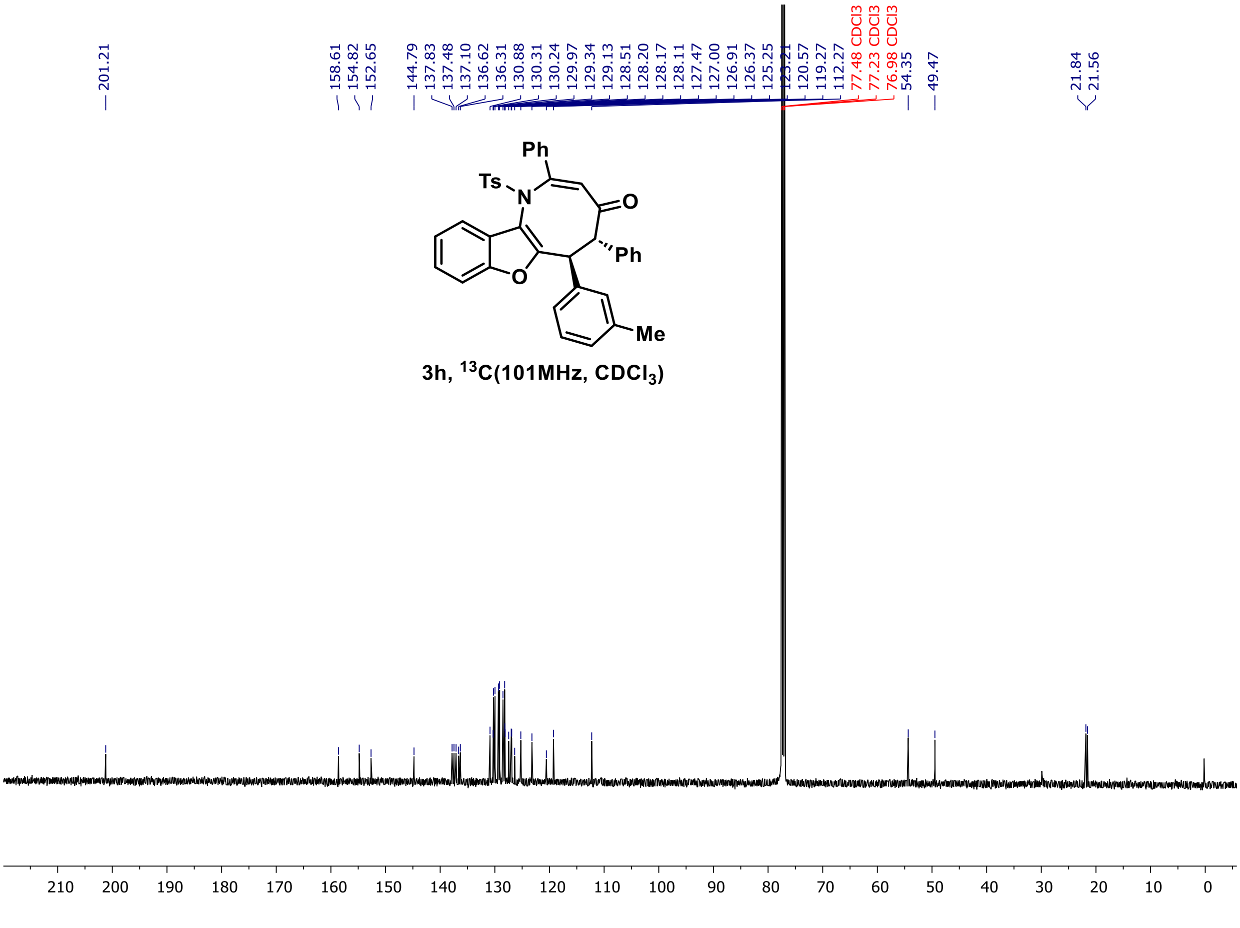

3h, ^{13}C(101MHz, $CDCl_3$)

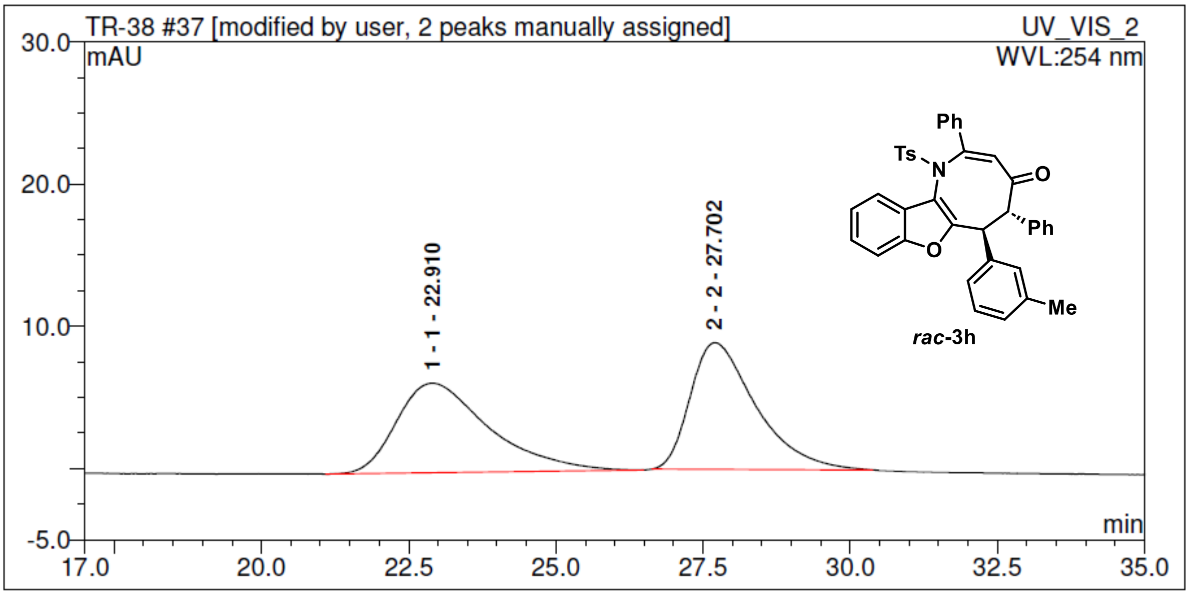

Peak	Name	Ret.Time (detected) min	Area mAU*min	Rel.Area(ident.) %	Height mAU	Amount
1	1	22.91	11.40874	49.76460207	6.29139	n.a.
2	2	27.70	11.517	50.23539793	8.908	n.a.

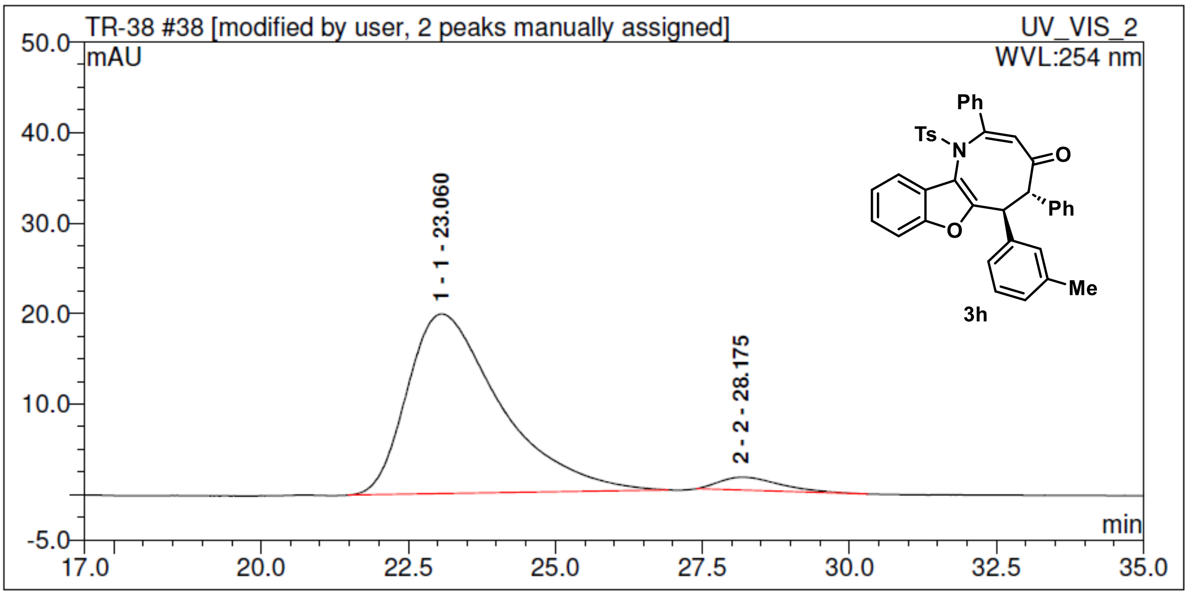

Peak	Name	Ret.Time (detected) min	Area mAU*min	Rel.Area(ident.) %	Height mAU	Amount
1	1	23.06	36.53315	95.56842633	19.83159	n.a.
2	2	28.18	1.694	4.431573672	1.415	n.a.

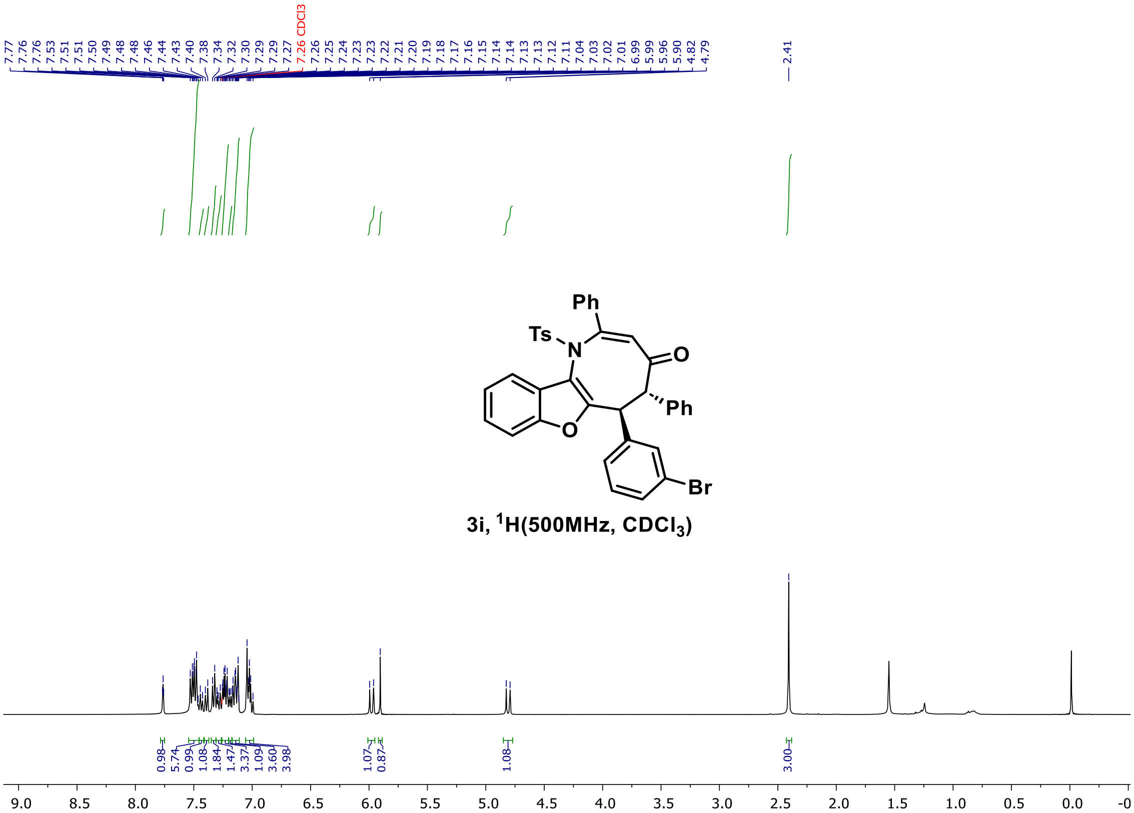
7.77 7.76 7.76 7.53 7.51 7.51 7.50 7.49 7.48 7.48 7.46 7.44 7.43 7.40 7.38 7.34 7.32 7.30 7.29 7.29 7.27 7.26 CDCl3 7.26 7.25 7.24 7.23 7.23 7.22 7.21 7.20 7.19 7.18 7.17 7.16 7.15 7.14 7.14 7.13 7.13 7.12 7.11 7.04 7.03 7.02 7.01 6.99 5.99 5.96 5.90 4.82 4.79
2.41
Ph
Ts
N
O
Ph
O
Br
3i, 1H(500MHz, CDCl3)
0.98 5.74 0.99 1.08 1.84 1.47 3.37 1.09 3.60 3.98
1.07 0.87
1.08
3.00
9.0 8.5 8.0 7.5 7.0 6.5 6.0 5.5 5.0 4.5 4.0 3.5 3.0 2.5 2.0 1.5 1.0 0.5 0.0 -0

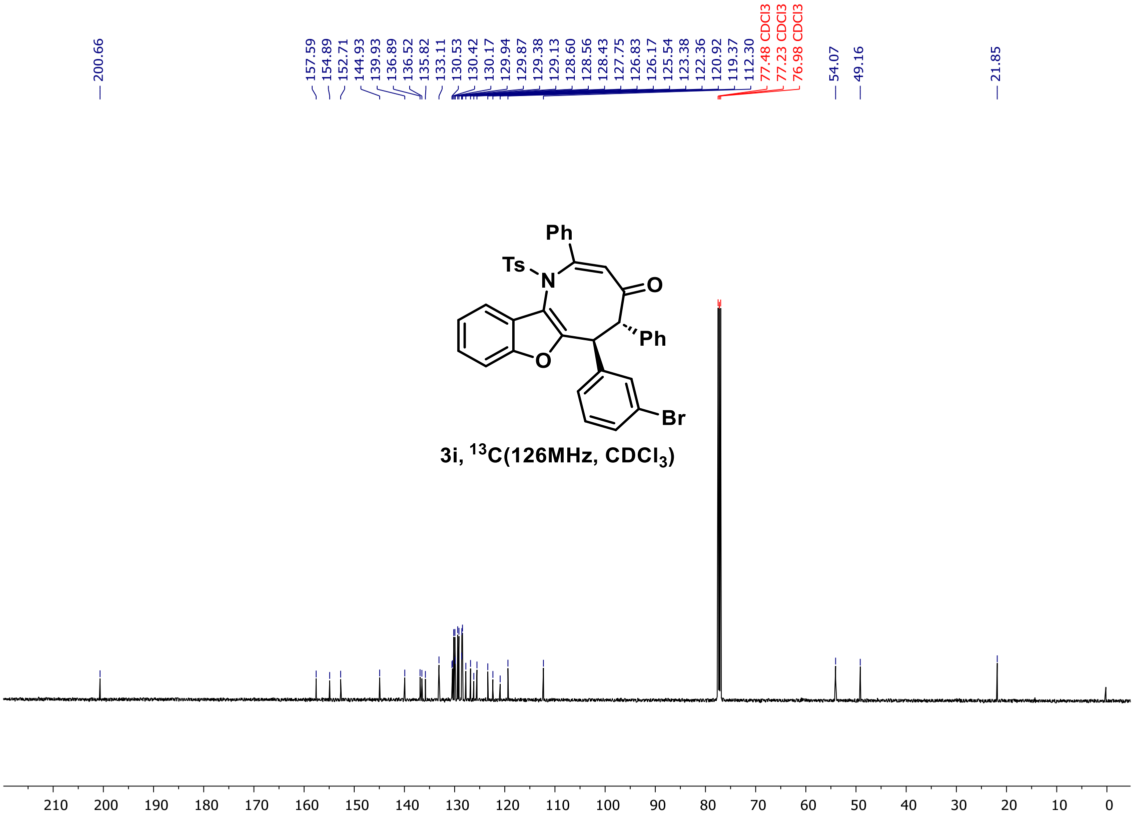
200.66
157.59 154.89 152.71 144.93 139.93 136.89 136.52 135.82 133.11 130.53 130.42 130.17 129.94 129.87 129.38 129.13 128.60 128.56 128.43 127.75 126.83 126.17 125.54 123.38 122.36 120.92 119.37 112.30
77.48 CDCl3 77.23 CDCl3 76.98 CDCl3
54.07 49.16
21.85
Ph
Ts
N
O
Ph
O
Br
3i, 13C(126MHz, CDCl3)
210 200 190 180 170 160 150 140 130 120 110 100 90 80 70 60 50 40 30 20 10 0

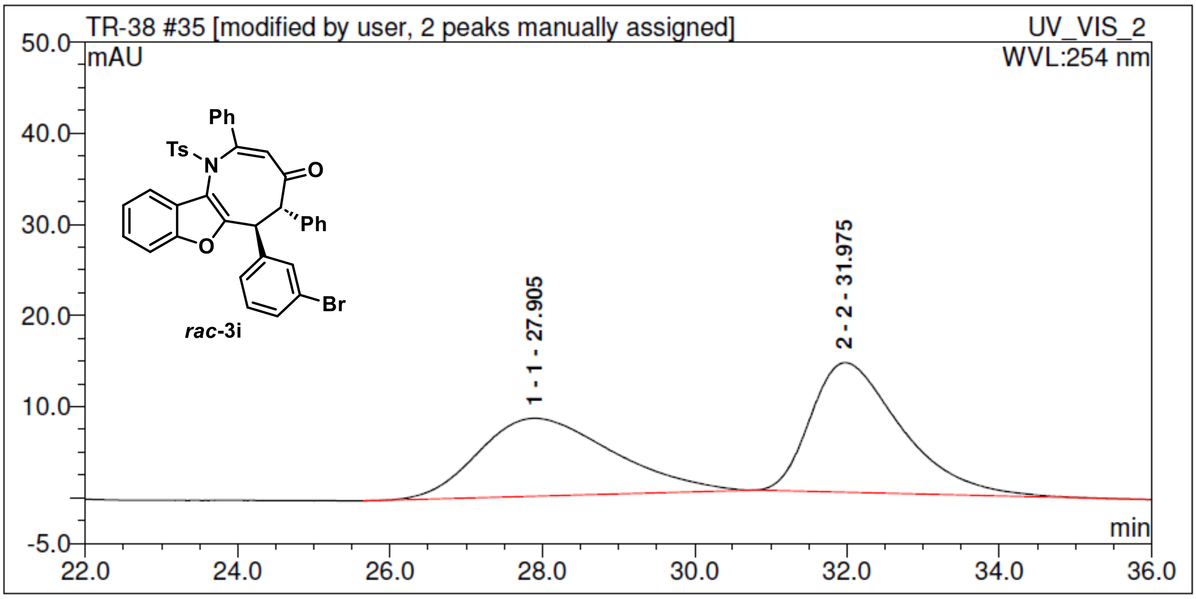

	Peak Name	Ret.Time (detected) min	Area mAU*min	Rel.Area(ident.) %	Height mAU	Amount
1	1	27.91	17.44212	47.09660617	8.53579	n.a.
2	2	31.98	19.593	52.90339383	14.206	n.a.

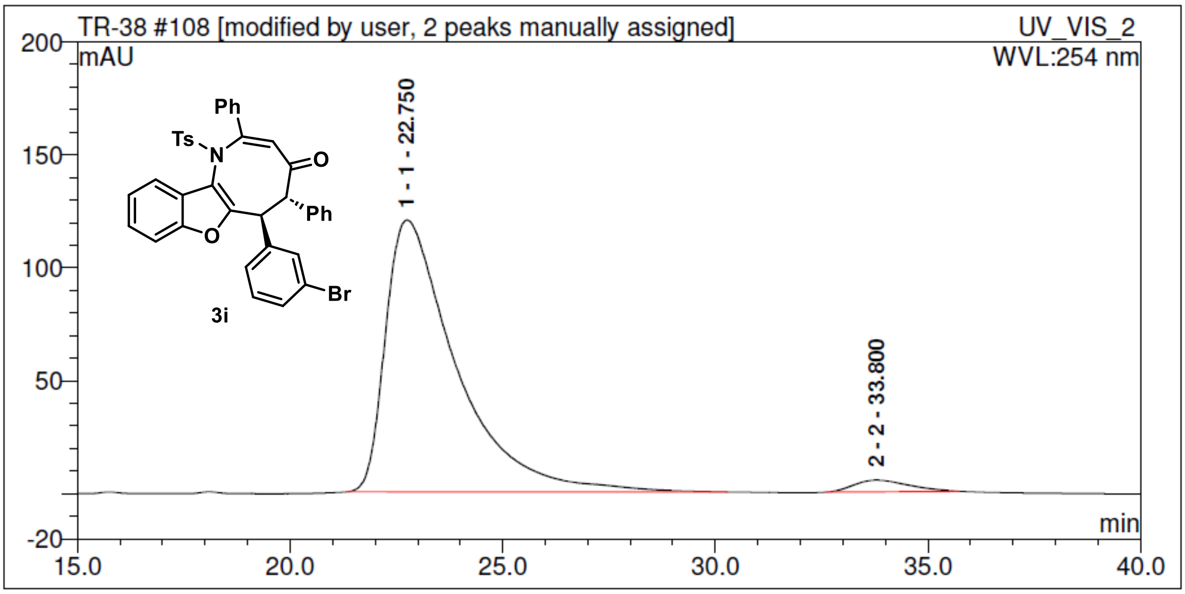

	Peak Name	Ret.Time (detected) min	Area mAU*min	Rel.Area(ident.) %	Height mAU	Amount
1	1	22.75	232.2966	96.79321216	120.3302	n.a.
2	2	33.80	7.696	3.20678784	5.158	n.a.

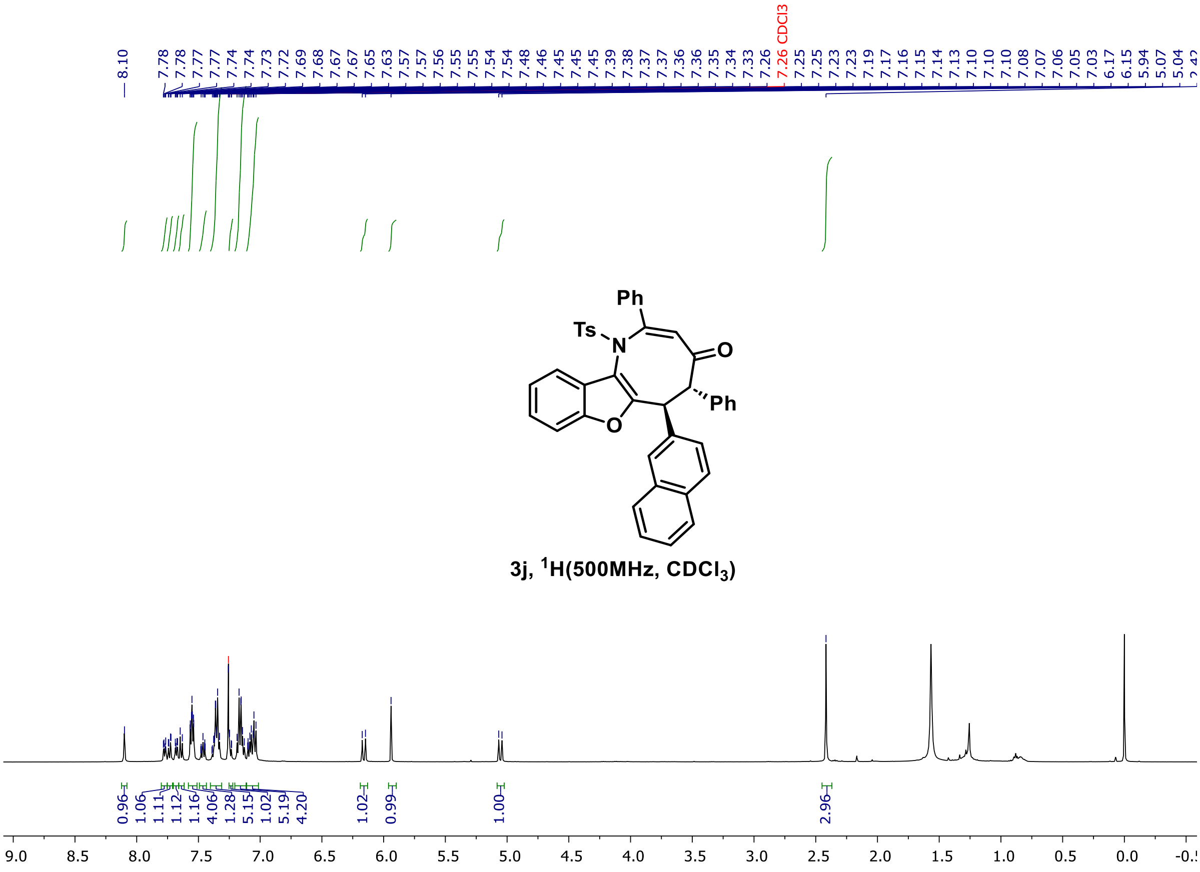
Ph
Ts
N
O
Ph
O
3j, ¹H(500MHz, CDCl₃)

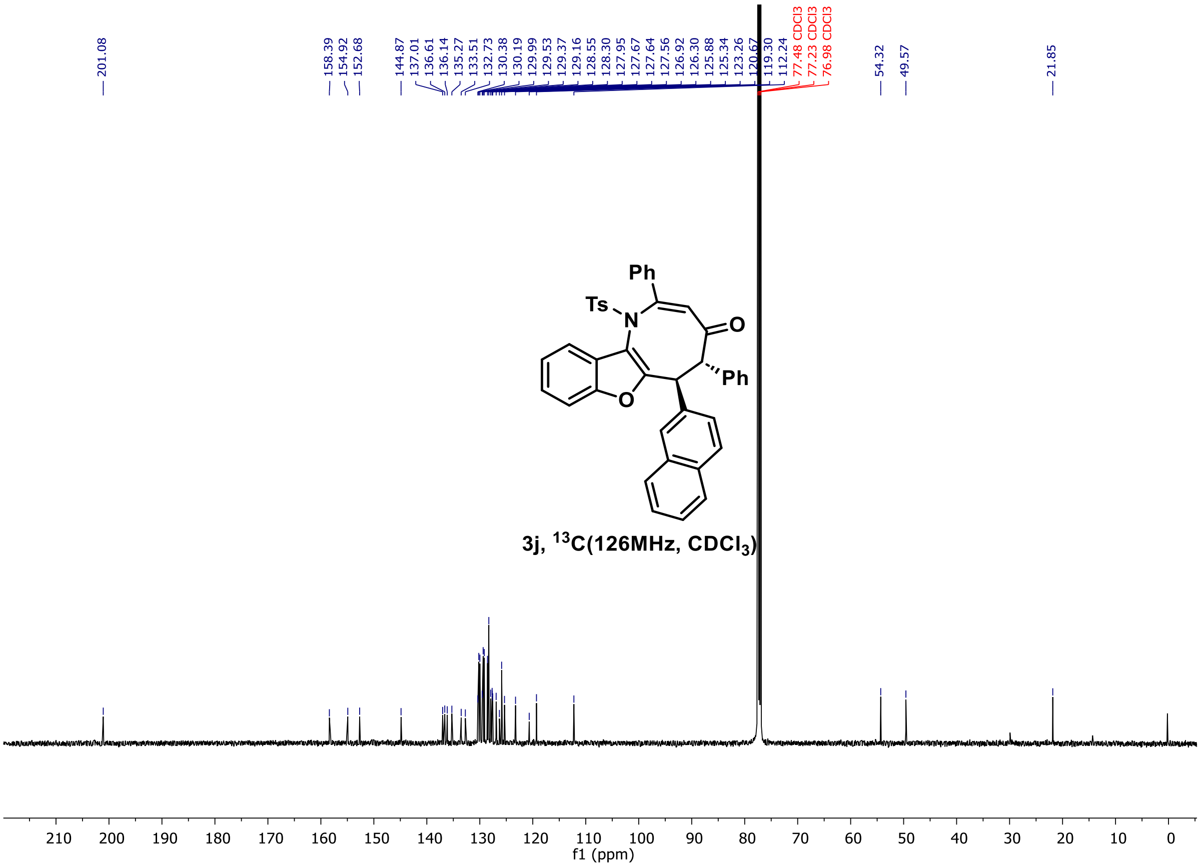
Ph
Ts
N
O
Ph
O
3j, ¹³C(126MHz, CDCl₃)
f1 (ppm)

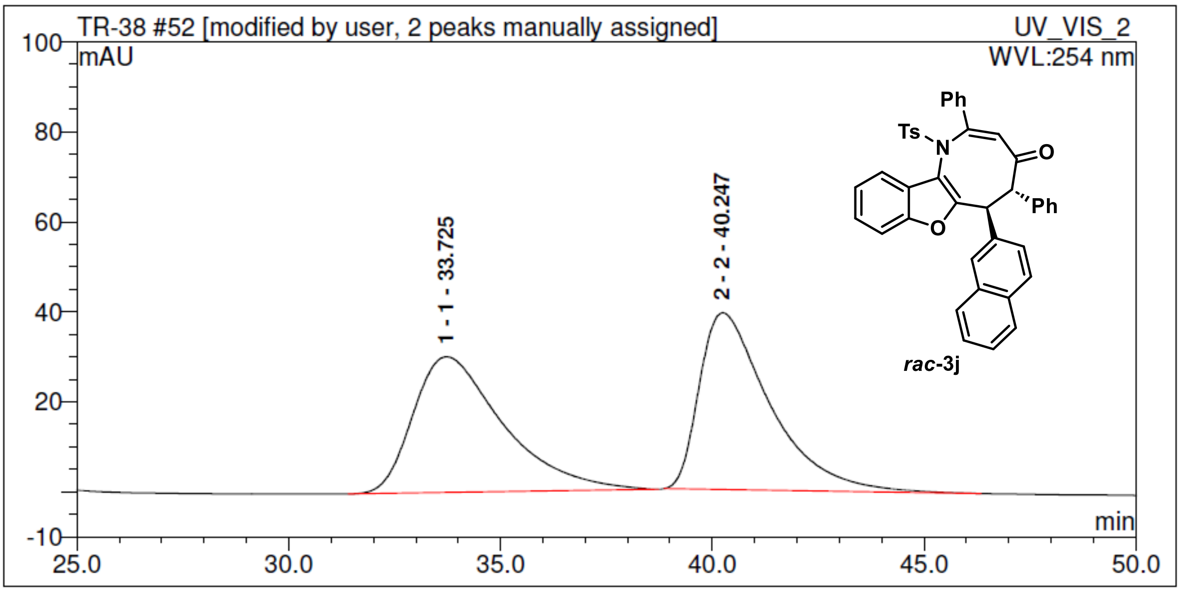

	Peak Name	Ret.Time (detected) min	Area mAU*min	Rel.Area(ident.) %	Height mAU	Amount
1	1	33.73	74.28044	49.45925474	30.16289	n.a.
2	2	40.25	75.905	50.54074526	39.287	n.a.

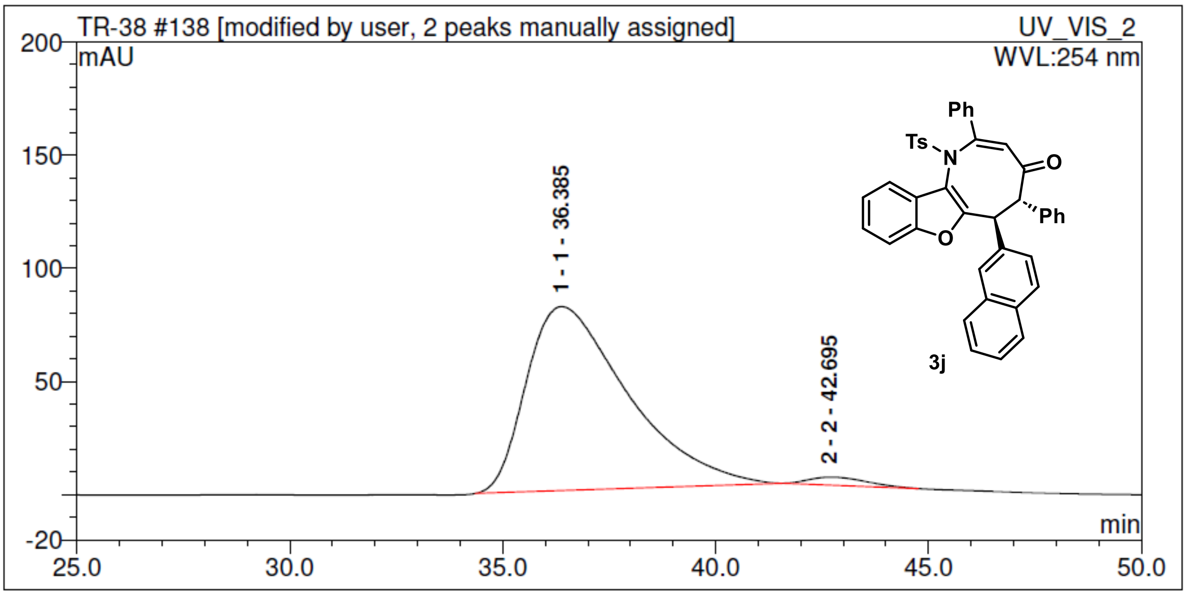

	Peak Name	Ret.Time (detected) min	Area mAU*min	Rel.Area(ident.) %	Height mAU	Amount
1	1	36.39	224.7408	97.60599271	81.34282	n.a.
2	2	42.70	5.512	2.394007294	3.491	n.a.

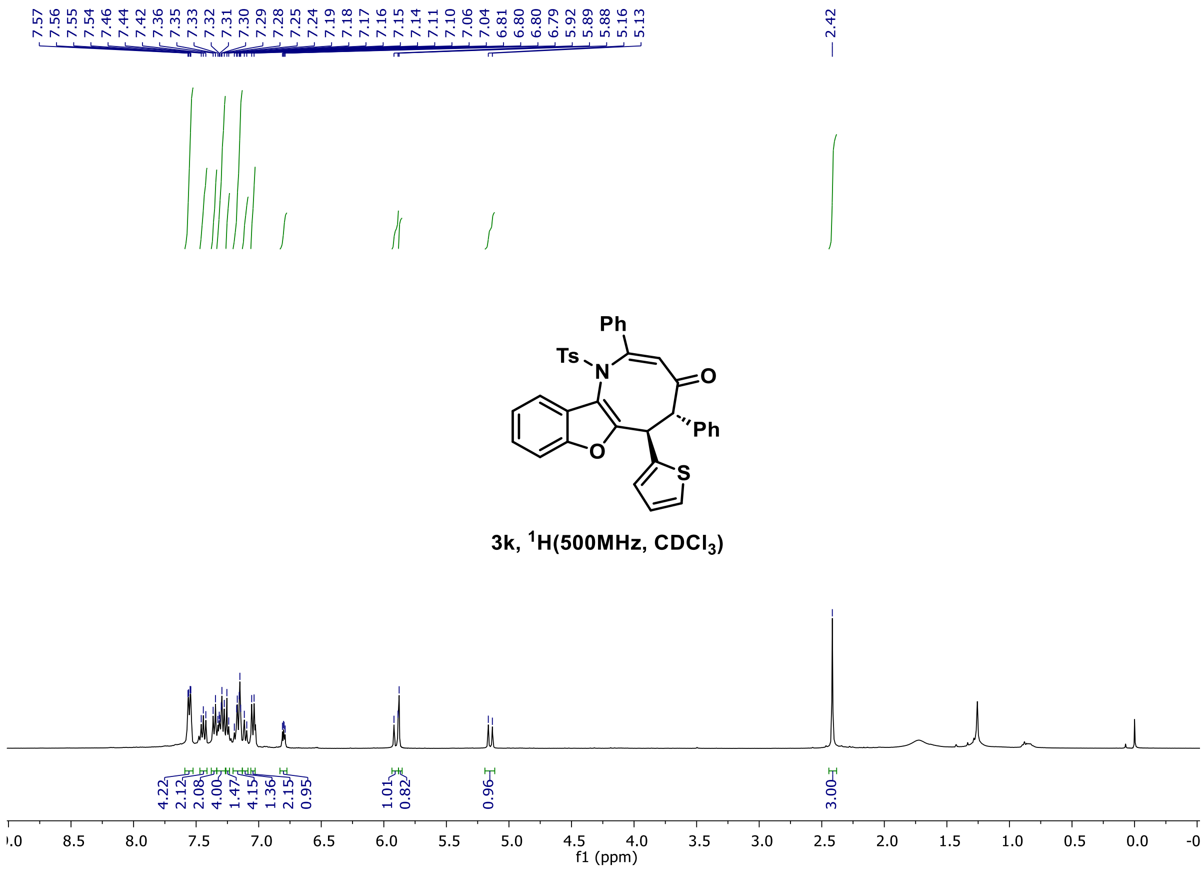

3k, ^{1}H(500MHz, CDCl$_3$)

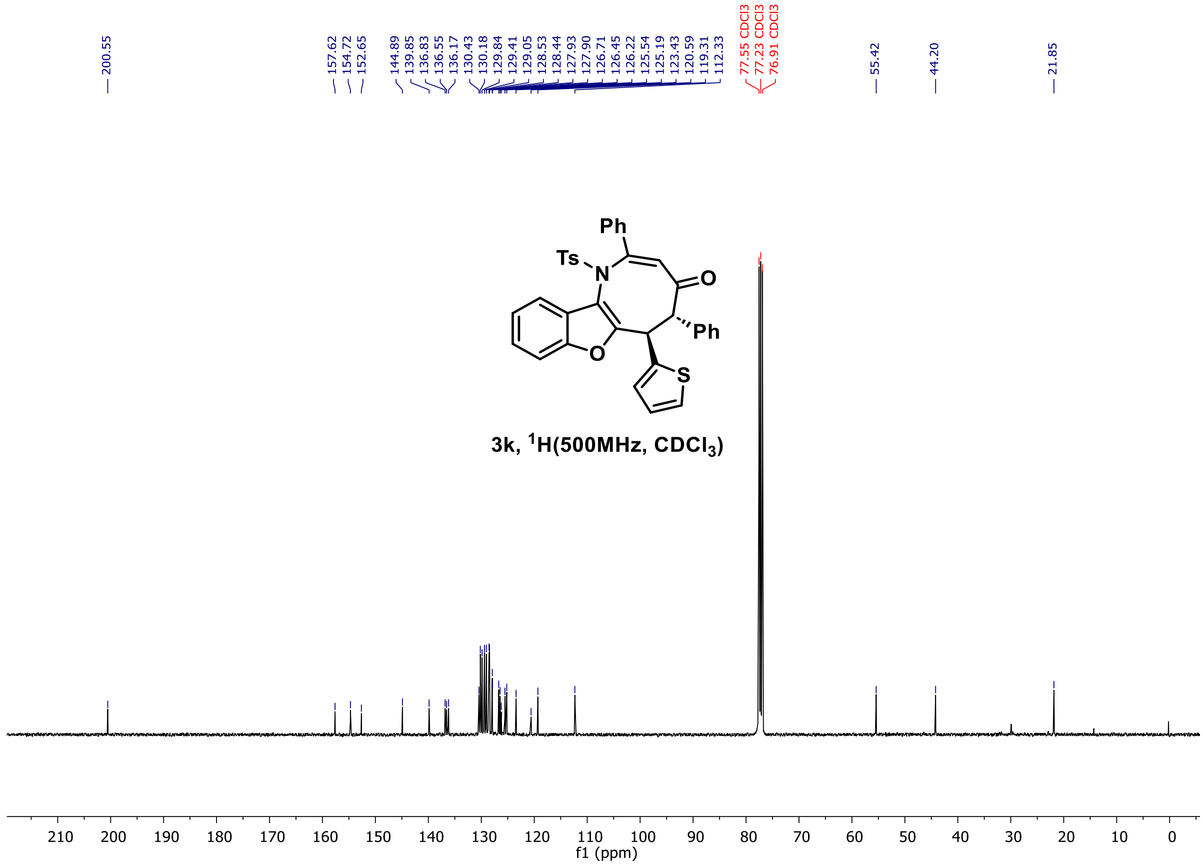

3k, ^{1}H(500MHz, CDCl$_3$)

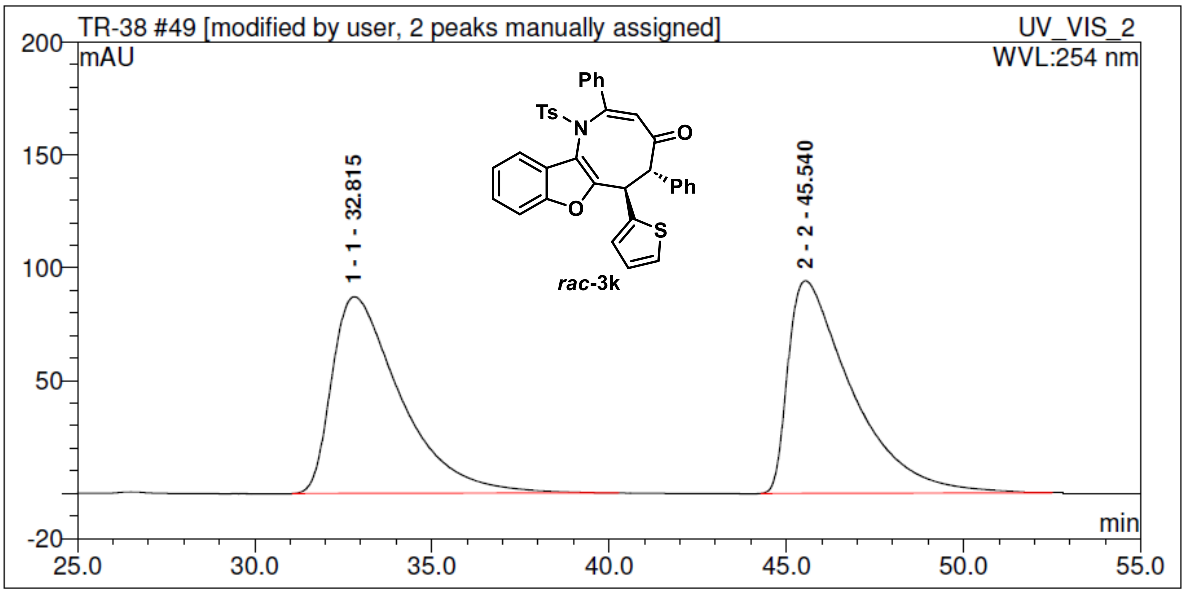

	Peak Name	Ret.Time (detected) min	Area mAU*min	Rel.Area(ident.) %	Height mAU	Amount
1	1	32.82	197.1547	49.97850332	87.21427	n.a.
2	2	45.54	197.324	50.02149668	94.196	n.a.

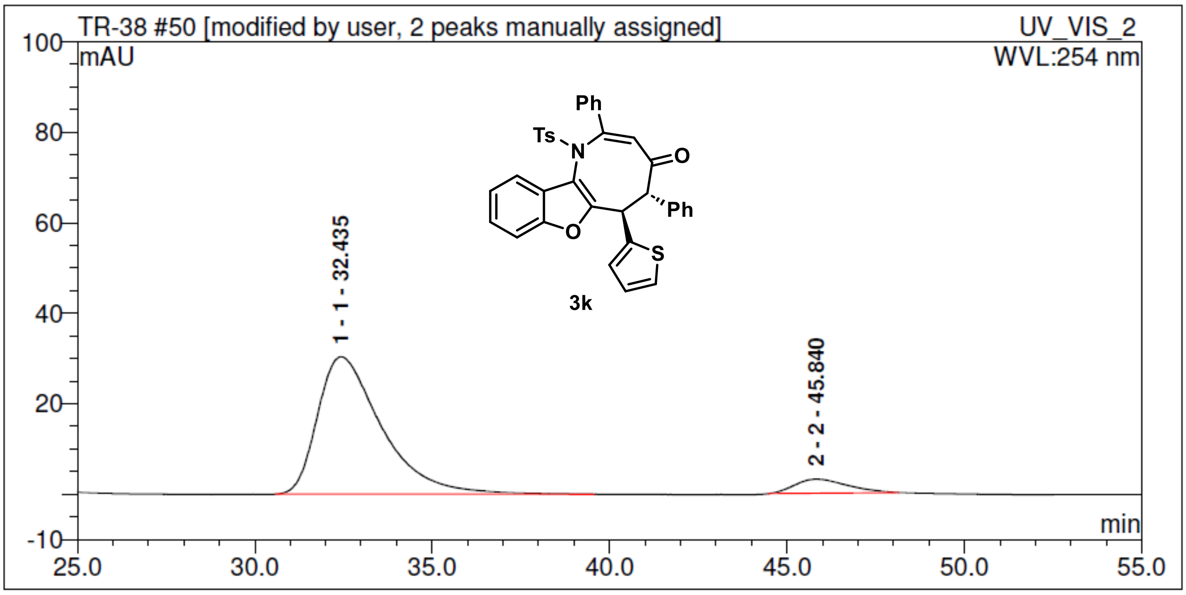

	Peak Name	Ret.Time (detected) min	Area mAU*min	Rel.Area(ident.) %	Height mAU	Amount
1	1	32.44	63.66954	92.29889345	30.37564	n.a.
2	2	45.84	5.312	7.701106546	3.075	n.a.

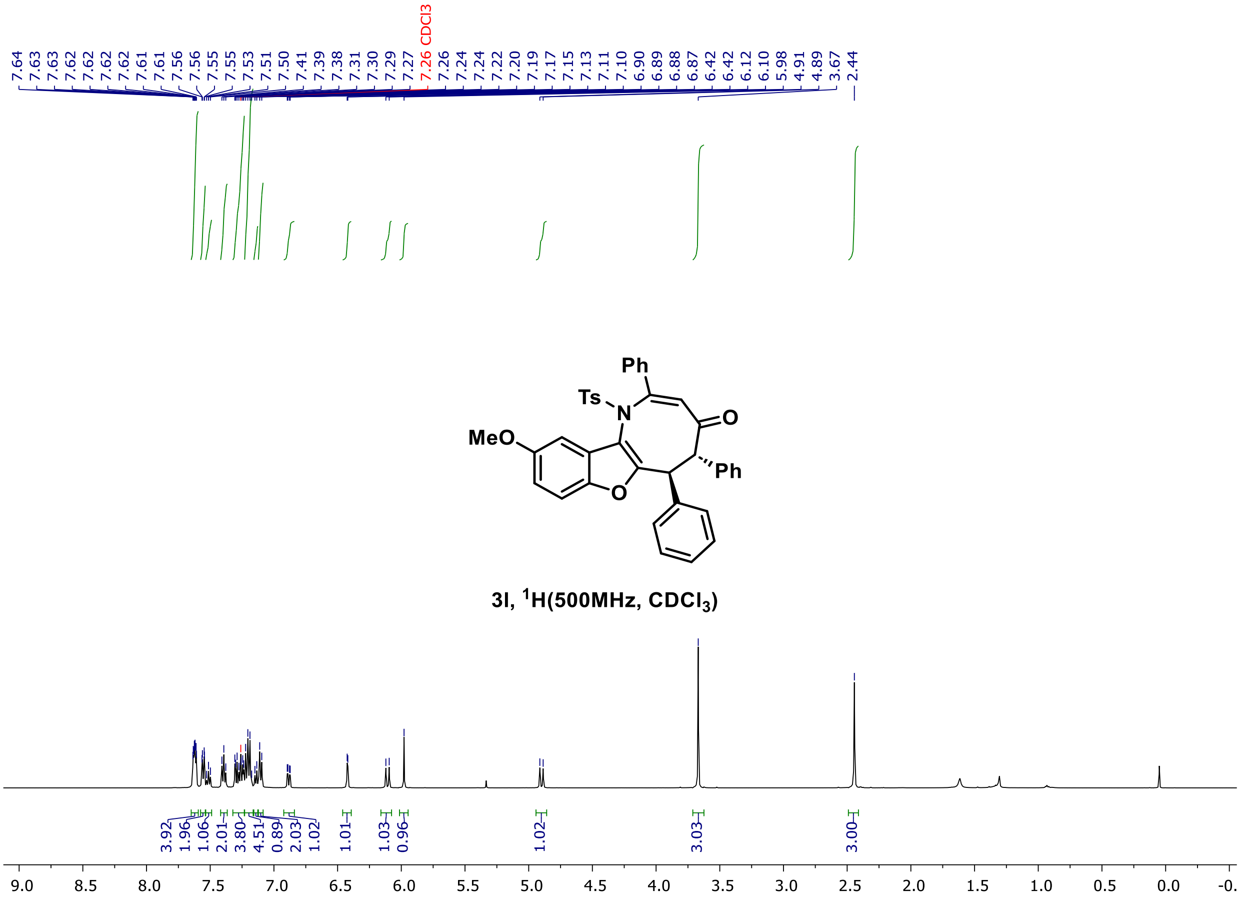

3l, ^{1}H(500MHz, $CDCl_3$)

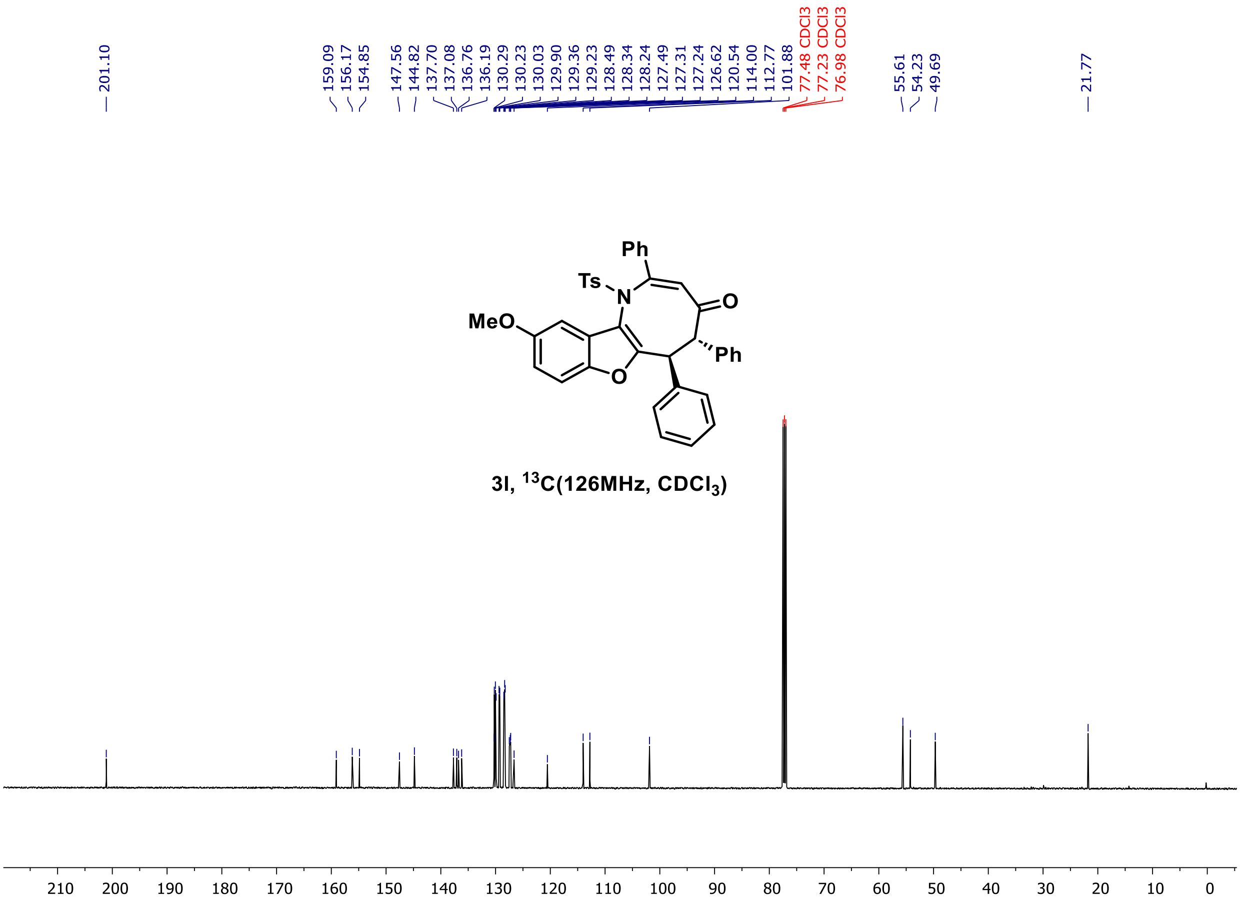

3l, ^{13}C(126MHz, $CDCl_3$)

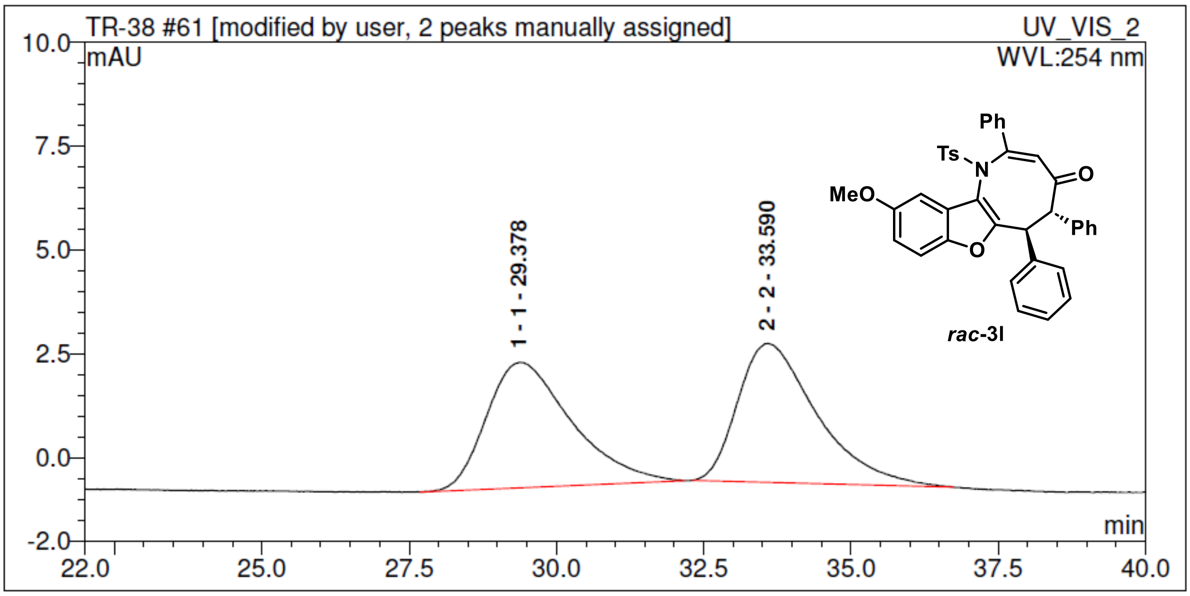

	Peak Name	Ret.Time (detected) min	Area mAU*min	Rel.Area(ident.) %	Height mAU	Amount
1	1	29.38	5.051715	49.32568142	3.02229	n.a.
2	2	33.59	5.190	50.67431858	3.345	n.a.

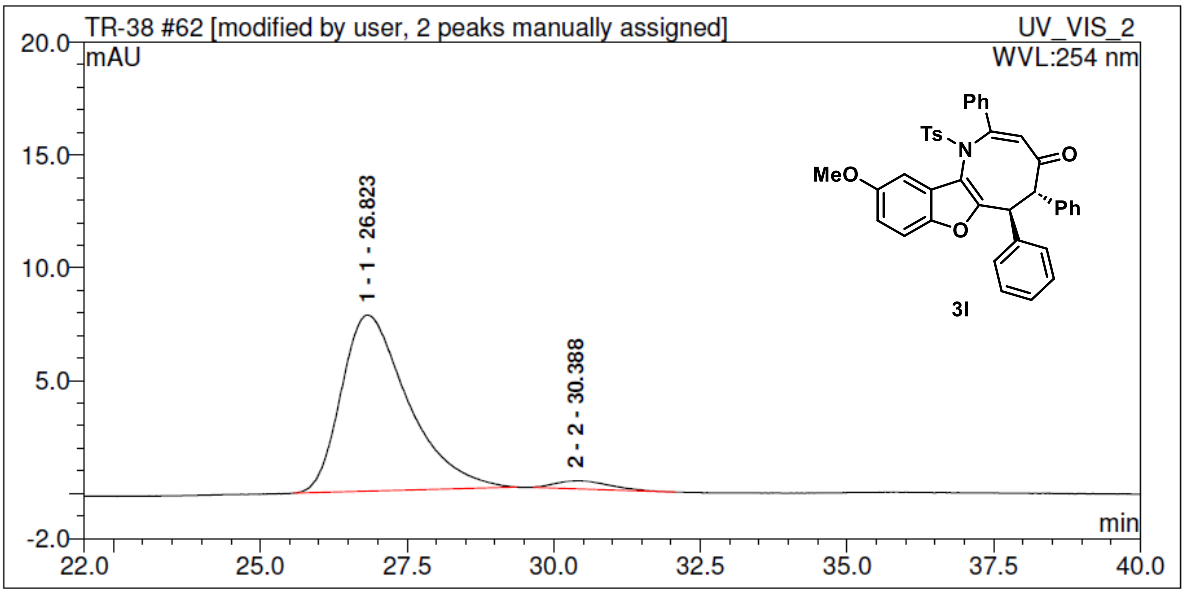

	Peak Name	Ret.Time (detected) min	Area mAU*min	Rel.Area(ident.) %	Height mAU	Amount
1	1	26.82	10.30699	96.44237086	7.80265	n.a.
2	2	30.39	0.380	3.557629141	0.357	n.a.

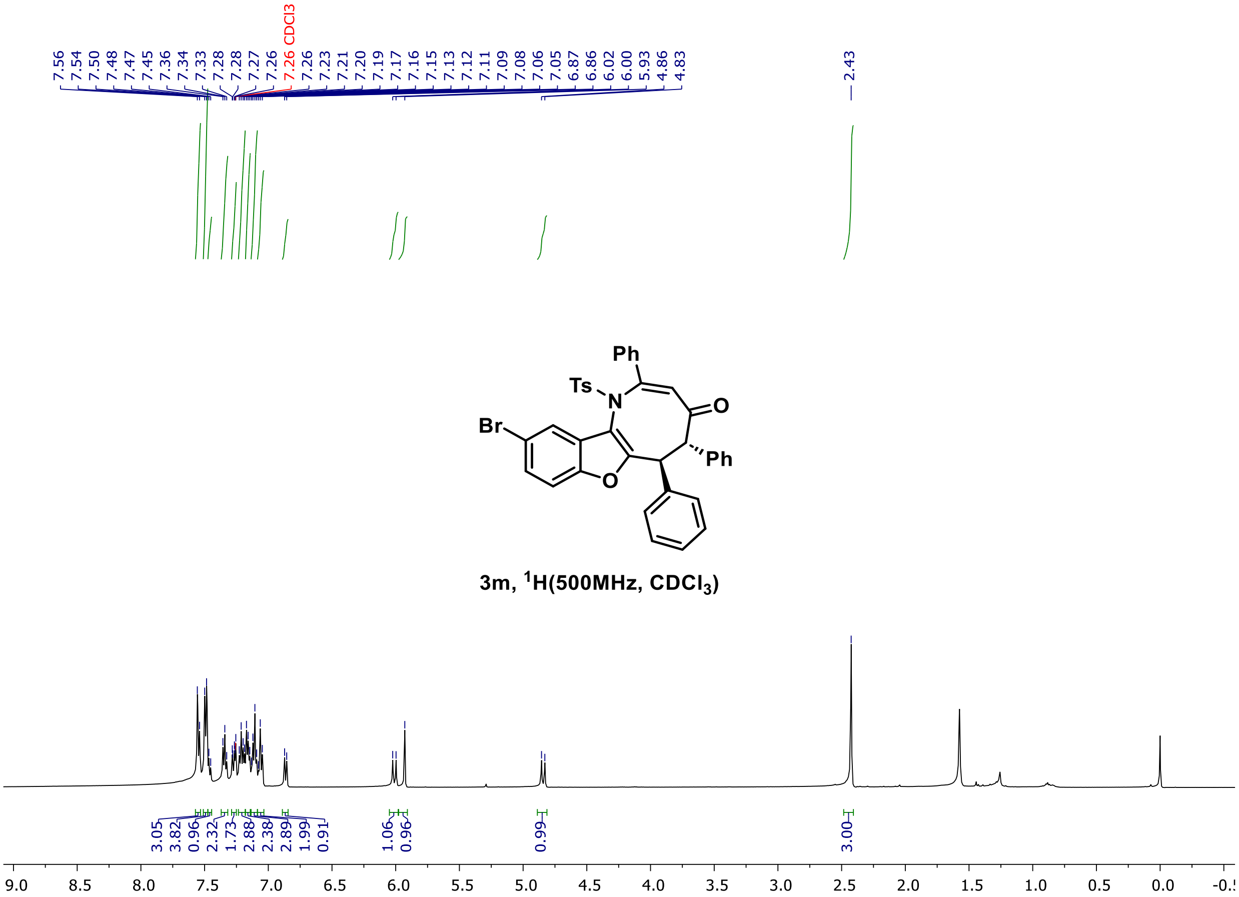

3m, $^{1}H(500MHz, CDCl_3)$

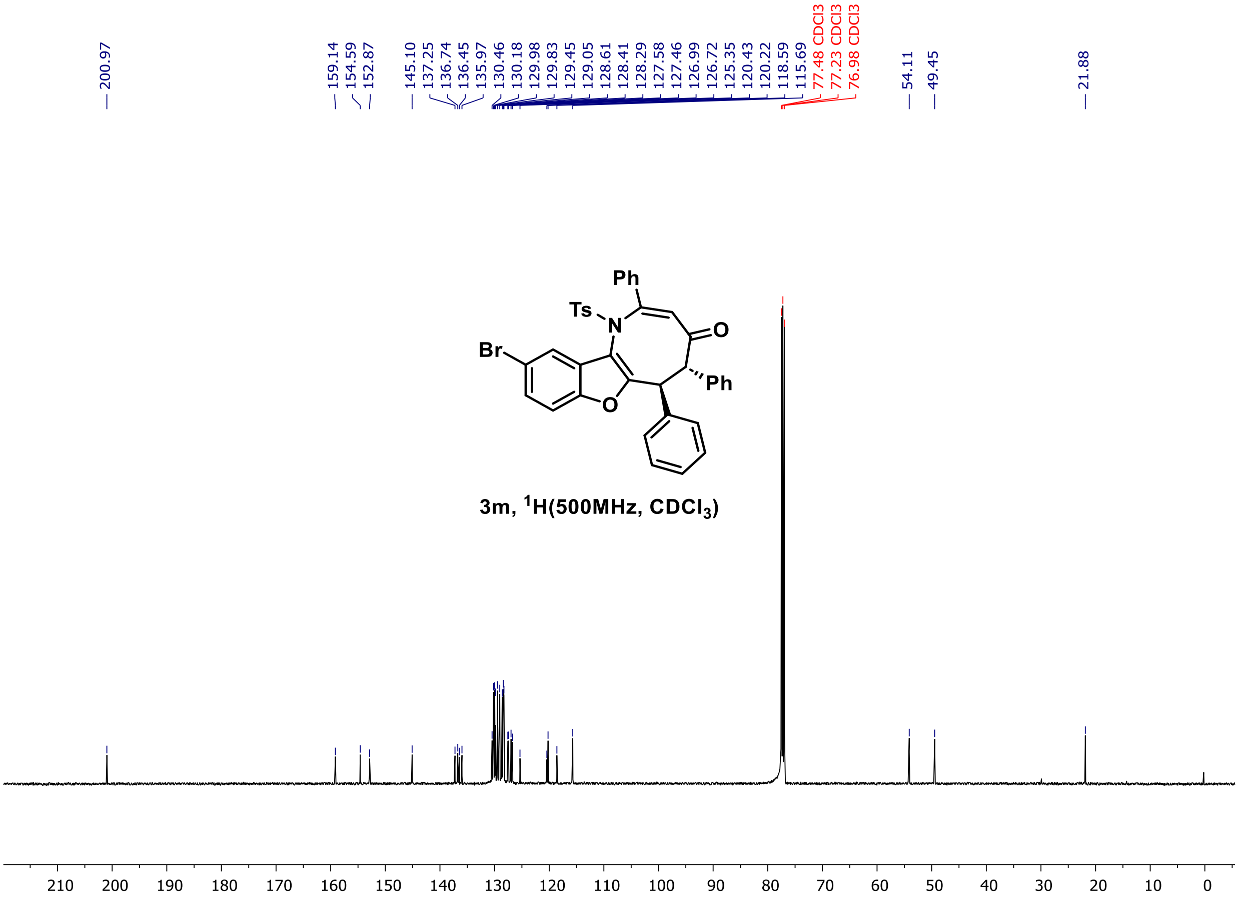

3m, $^{1}H(500MHz, CDCl_3)$

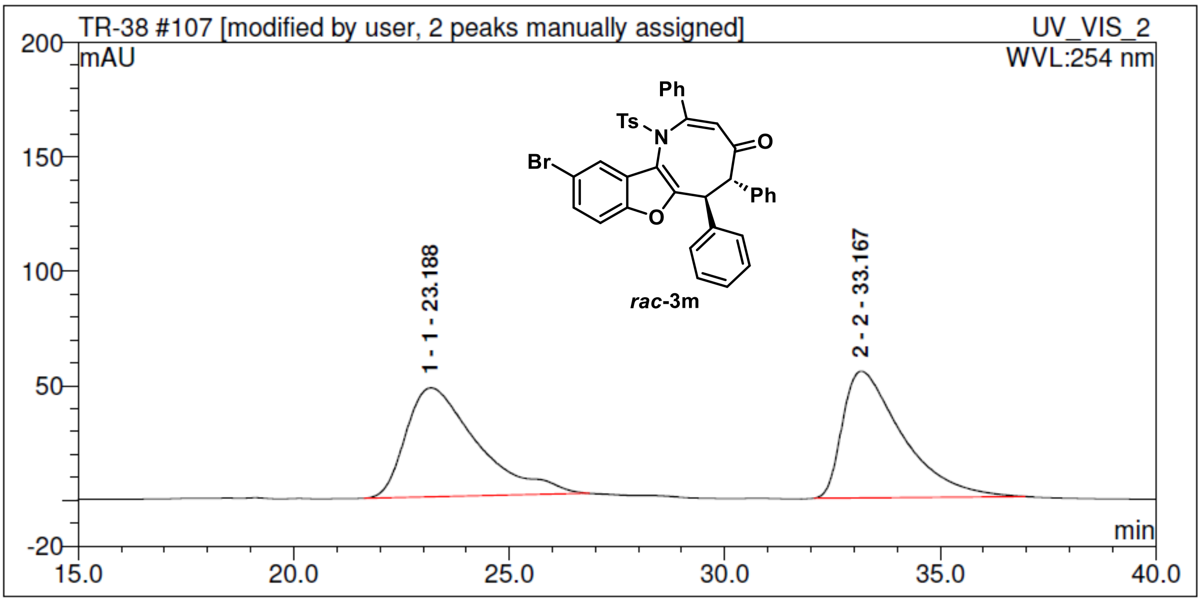

	Peak Name	Ret.Time (detected) min	Area mAU*min	Rel.Area(ident.) %	Height mAU	Amount
1	1	23.19	91.46353	50.69076583	47.57757	n.a.
2	2	33.17	88.971	49.30923417	55.331	n.a.

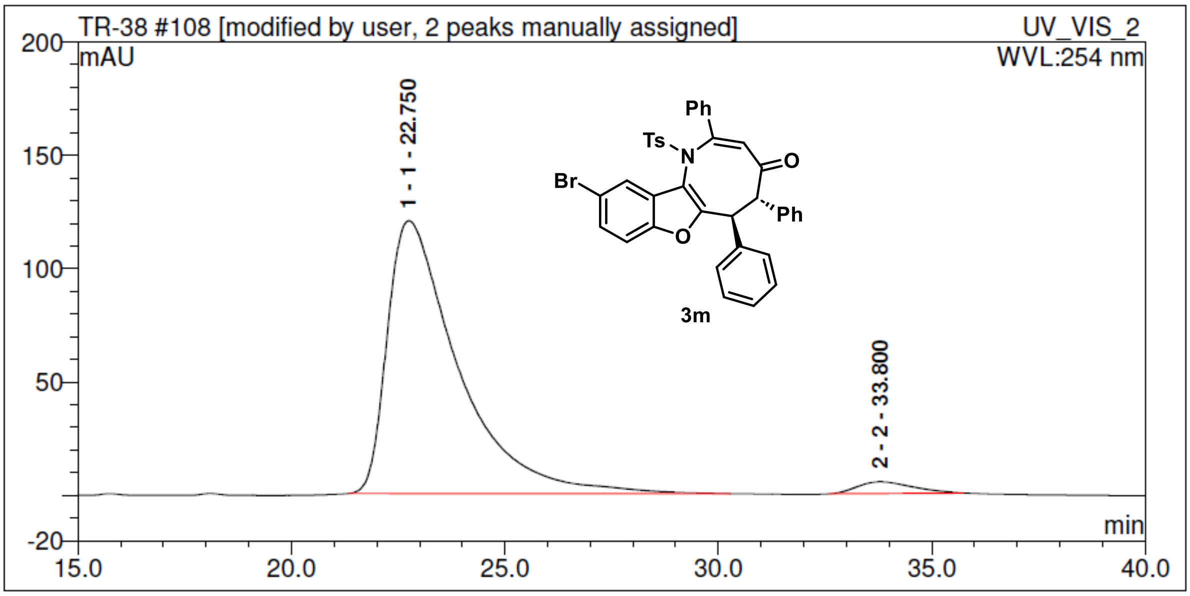

	Peak Name	Ret.Time (detected) min	Area mAU*min	Rel.Area(ident.) %	Height mAU	Amount
1	1	22.75	232.2966	96.79321216	120.3302	n.a.
2	2	33.80	7.696	3.20678784	5.158	n.a.

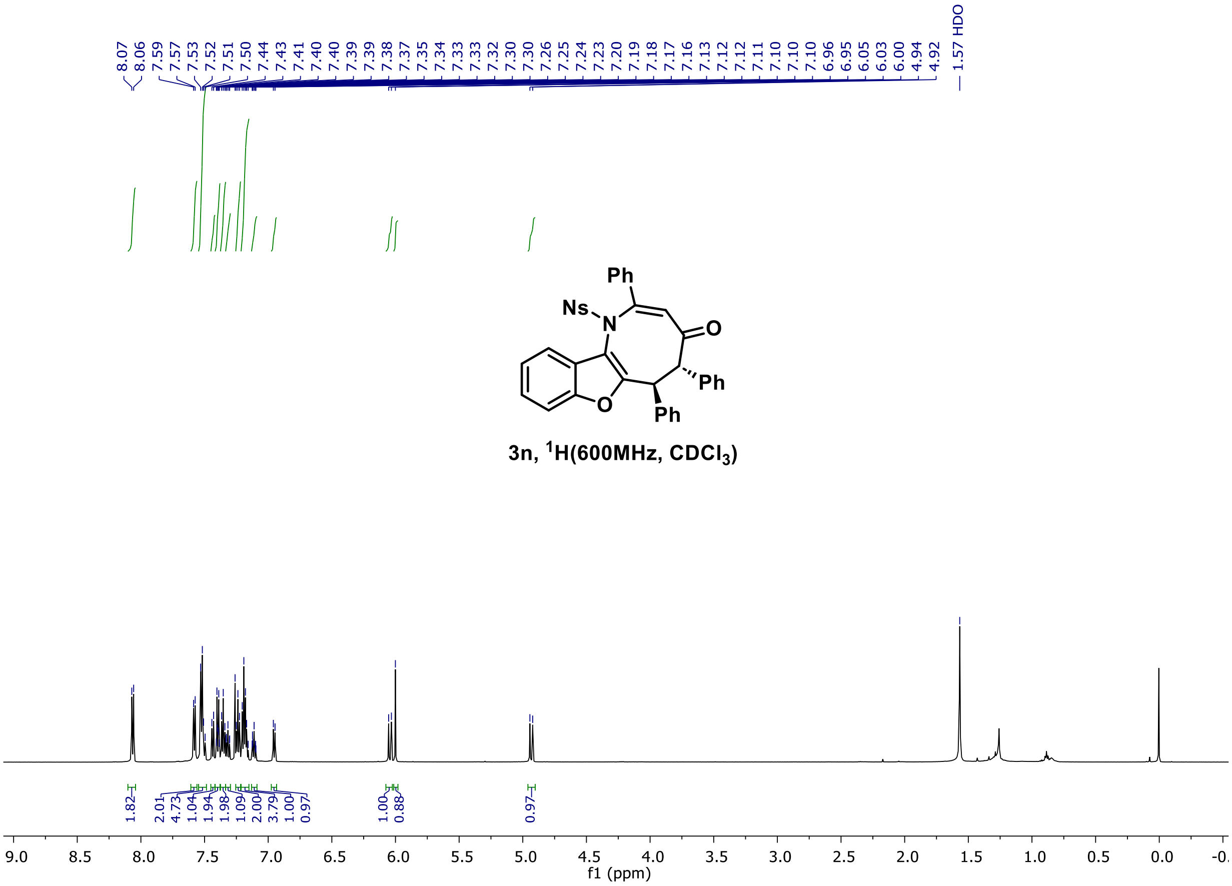
8.07 8.06 7.59 7.57 7.53 7.52 7.51 7.50 7.44 7.43 7.41 7.40 7.40 7.39 7.39 7.38 7.37 7.35 7.34 7.33 7.33 7.32 7.30 7.30 7.26 7.25 7.24 7.23 7.20 7.19 7.18 7.17 7.16 7.13 7.12 7.12 7.11 7.10 7.10 7.10 6.96 6.95 6.05 6.03 6.00 4.94 4.92
1.57 HDO
Ph
Ns
N
O
Ph
Ph
3n, ^{1}H(600MHz, $CDCl_3$)
1.82 2.01 4.73 1.04 1.94 1.98 1.09 2.00 3.79 1.00 0.97 1.00 0.88 0.97
f1 (ppm)

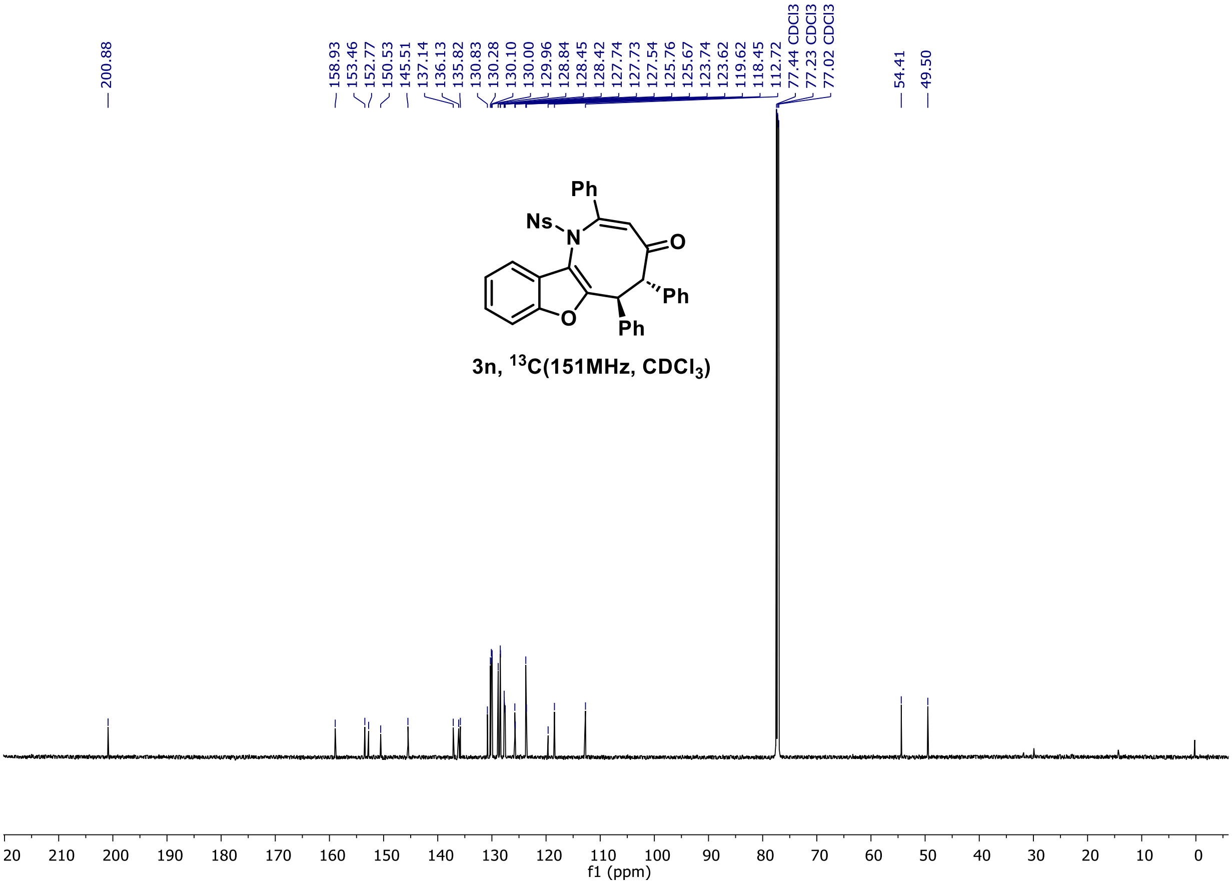
200.88
158.93 153.46 152.77 150.53 145.51 137.14 136.13 135.82 130.83 130.28 130.10 130.00 129.96 128.84 128.45 128.42 127.74 127.73 127.54 125.76 125.67 123.74 123.62 119.62 118.45 112.72 77.44 CDCl3 77.23 CDCl3 77.02 CDCl3
54.41
49.50
Ph
Ns
N
O
Ph
Ph
3n, ^{13}C(151MHz, $CDCl_3$)
f1 (ppm)

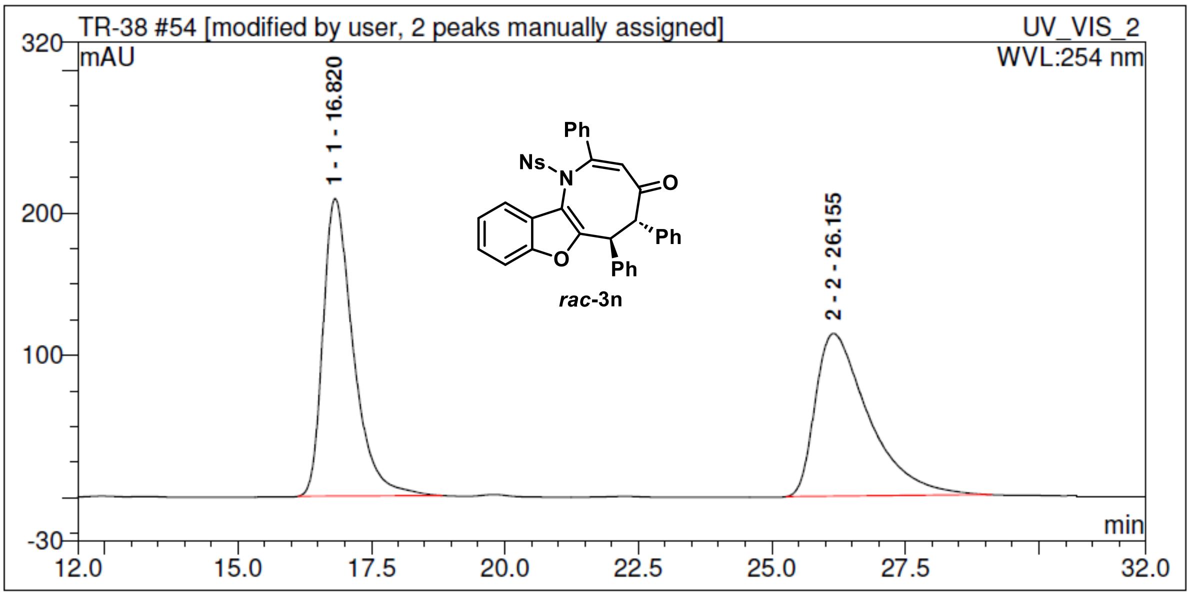

	Peak Name	Ret.Time (detected) min	Area mAU*min	Rel.Area(ident.) %	Height mAU	Amount
1	1	16.82	137.5071	50.86889527	208.9822	n.a.
2	2	26.16	132.810	49.13110473	114.243	n.a.

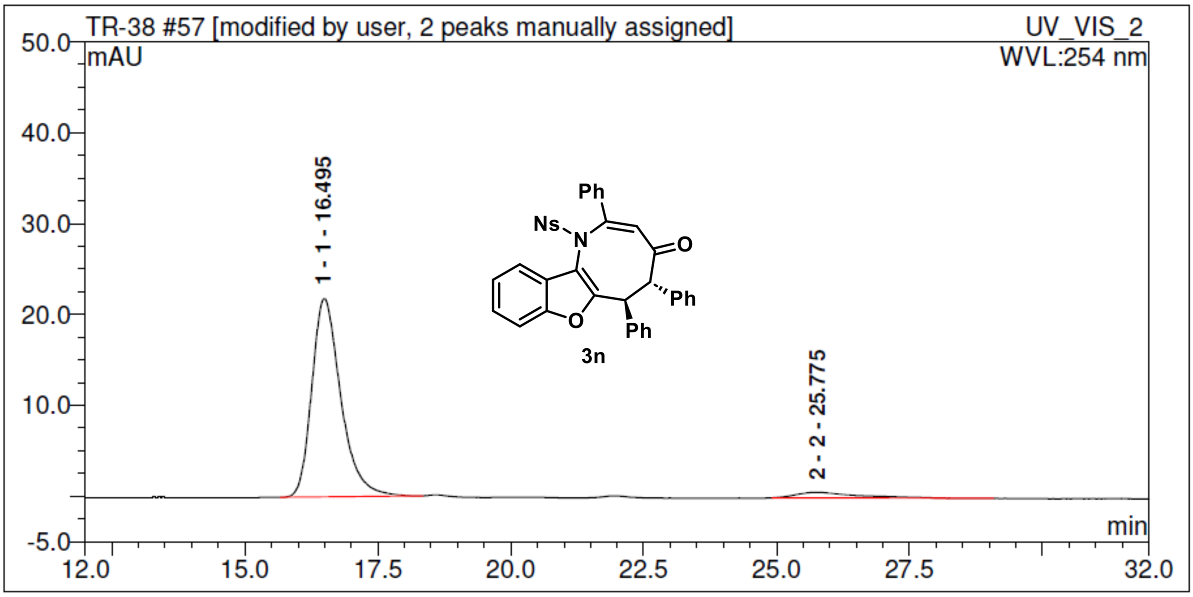

	Peak Name	Ret.Time (detected) min	Area mAU*min	Rel.Area(ident.) %	Height mAU	Amount
1	1	16.50	13.81931	94.15014287	21.79473	n.a.
2	2	25.78	0.859	5.849857129	0.613	n.a.

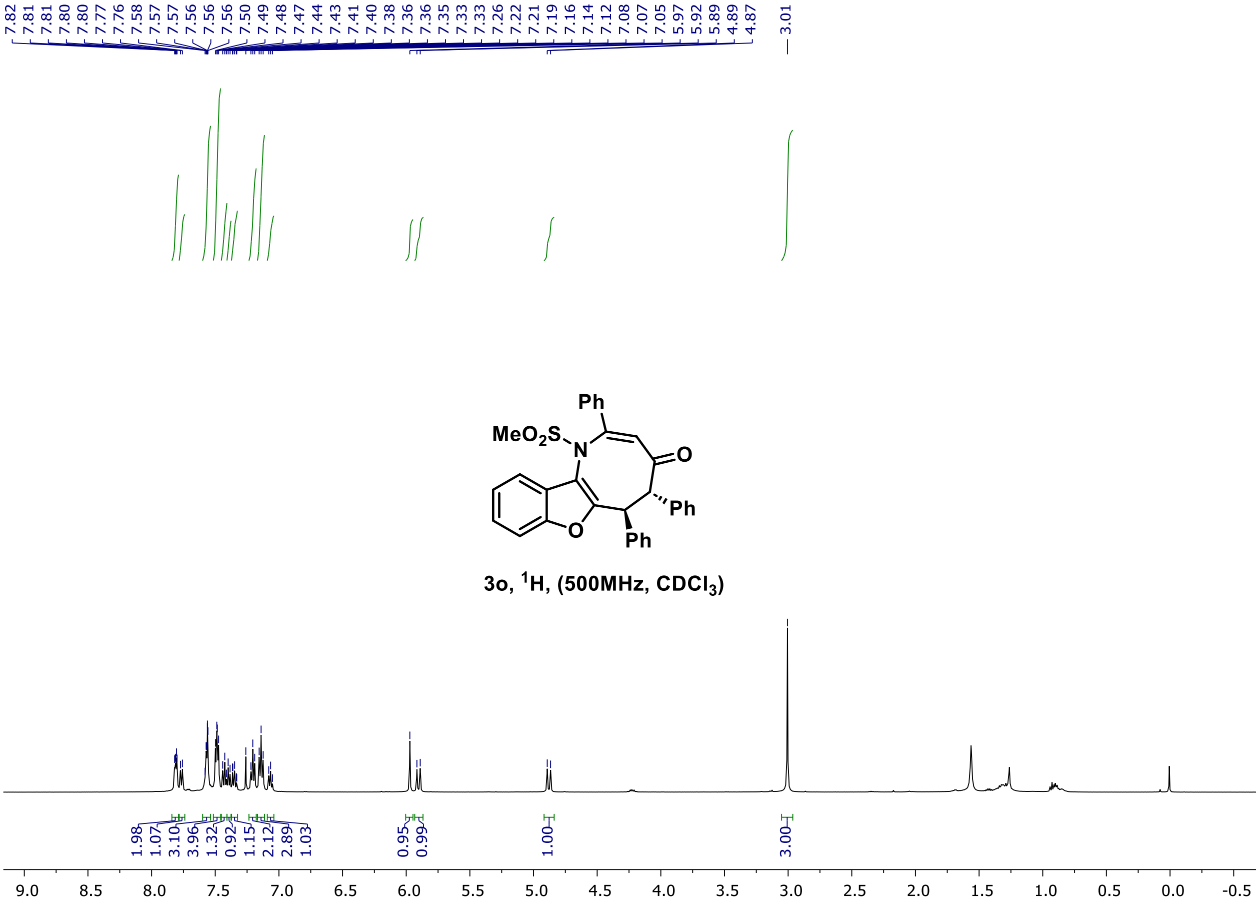

3o, ^1H, (500MHz, $CDCl_3$)

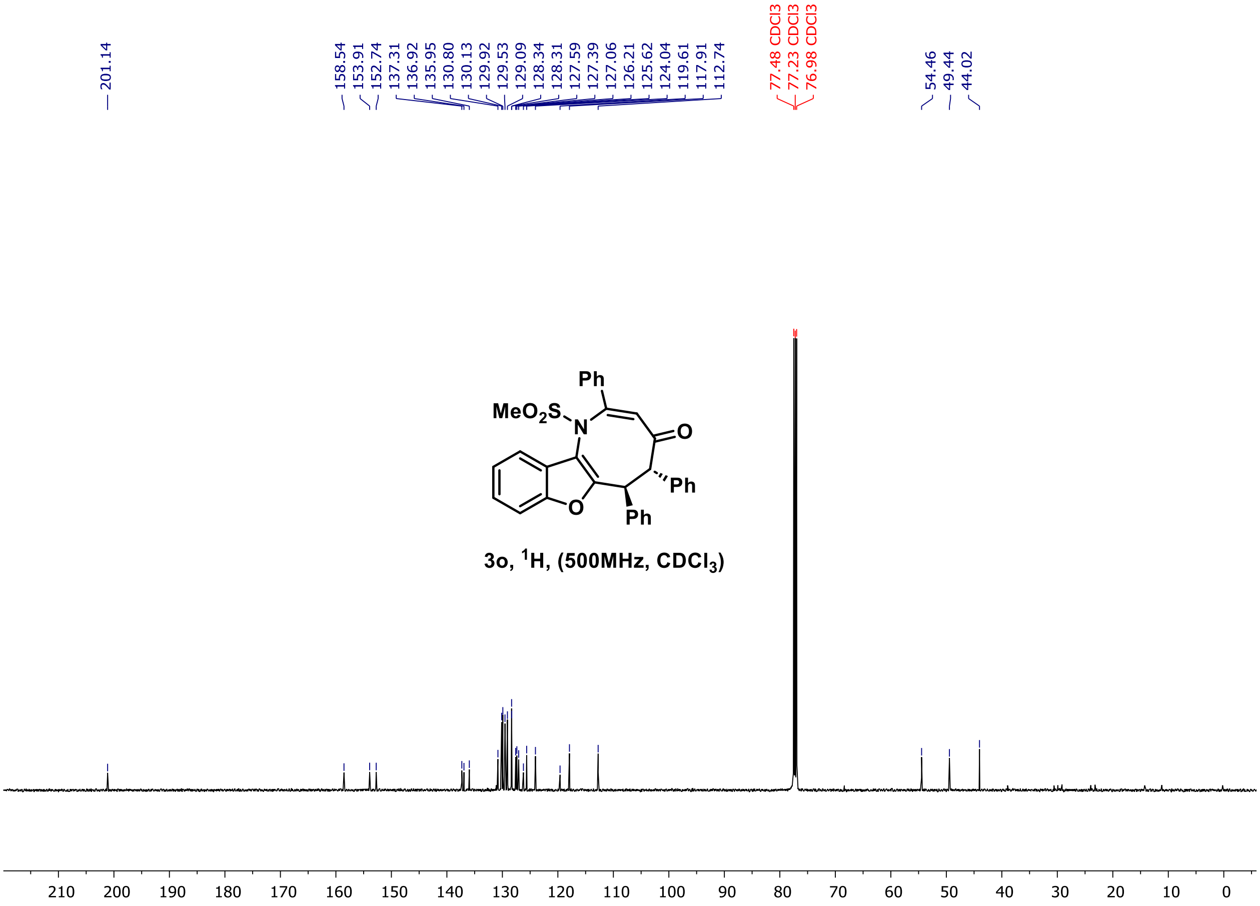

3o, ^1H, (500MHz, $CDCl_3$)

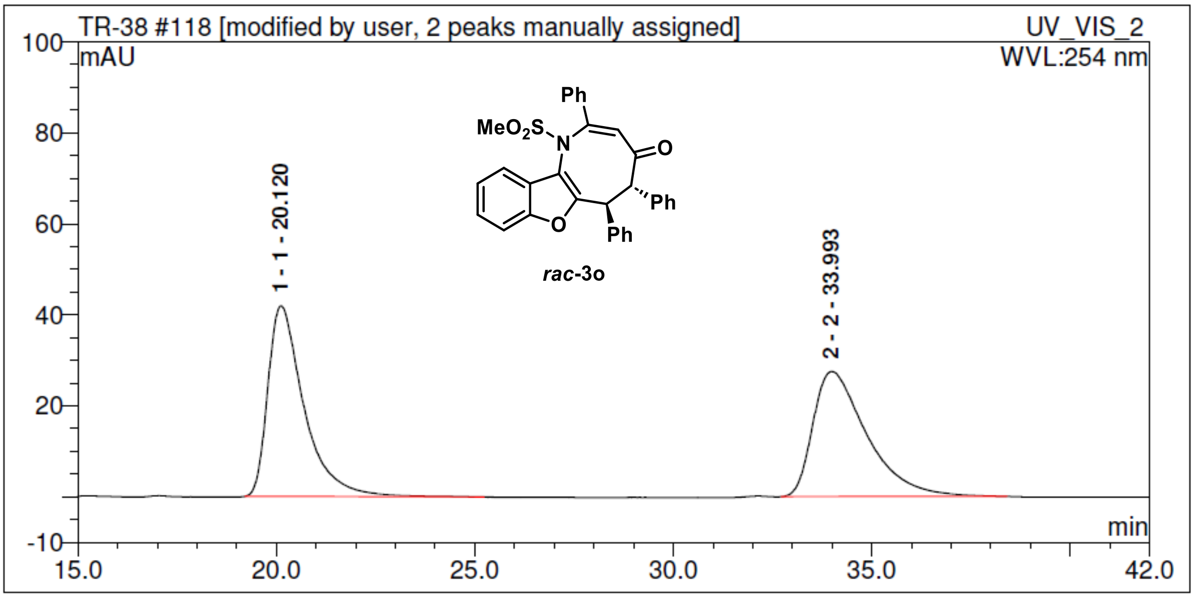

Peak	Name	Ret.Time (detected) min	Area mAU*min	Rel.Area(ident.) %	Height mAU	Amount
1	1	20.12	44.3637	50.63492978	41.90451	n.a.
2	2	33.99	43.251	49.36507022	27.504	n.a.

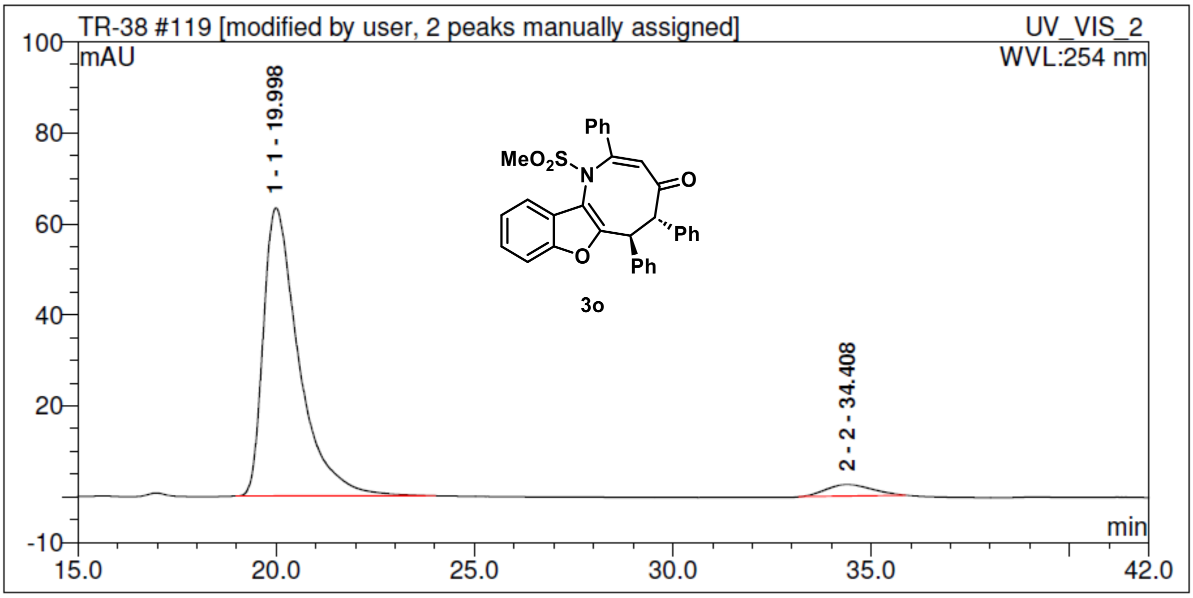

Peak	Name	Ret.Time (detected) min	Area mAU*min	Rel.Area(ident.) %	Height mAU	Amount
1	1	20.00	65.69633	95.10951237	63.29514	n.a.
2	2	34.41	3.378	4.890487627	2.524	n.a.

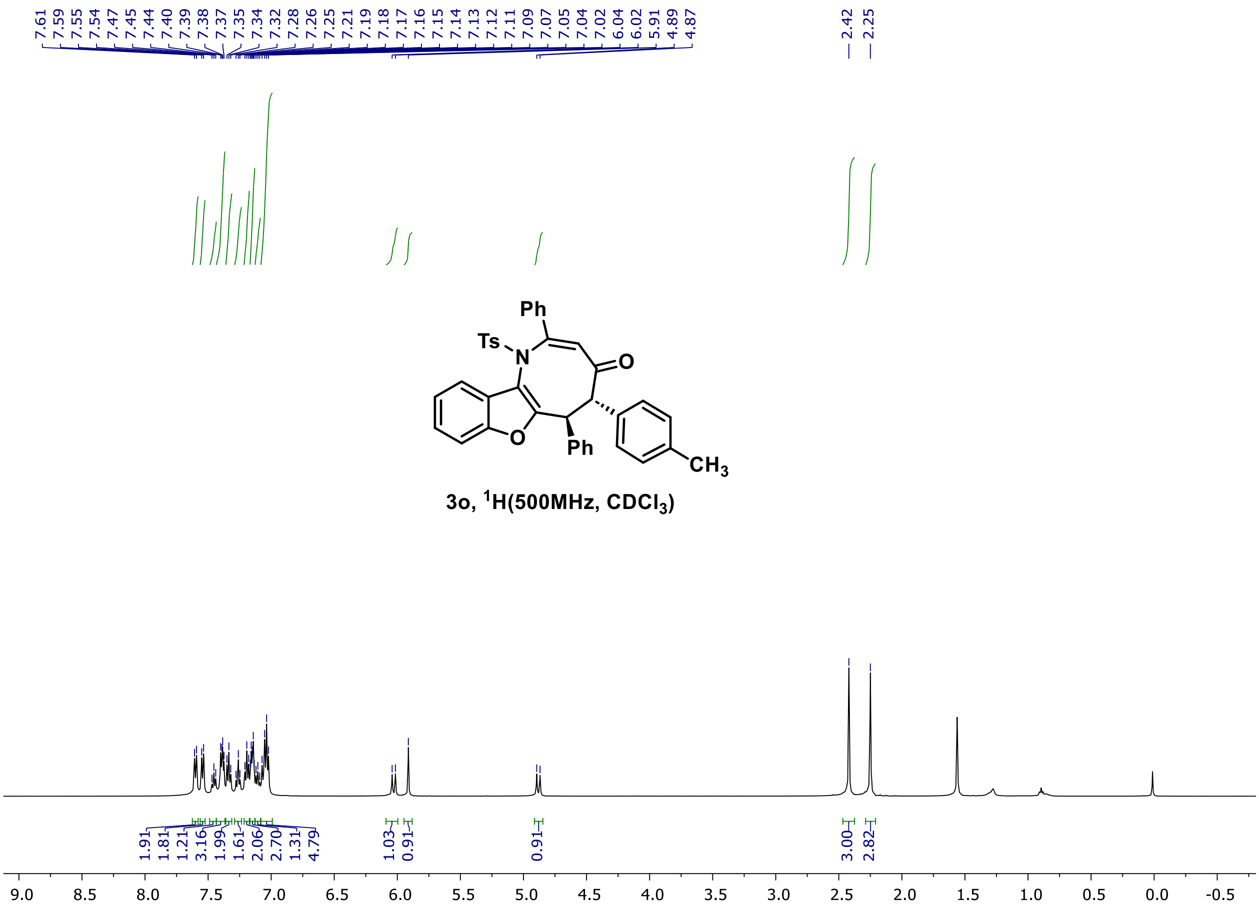

3o, ^{1}H(500MHz, $CDCl_3$)

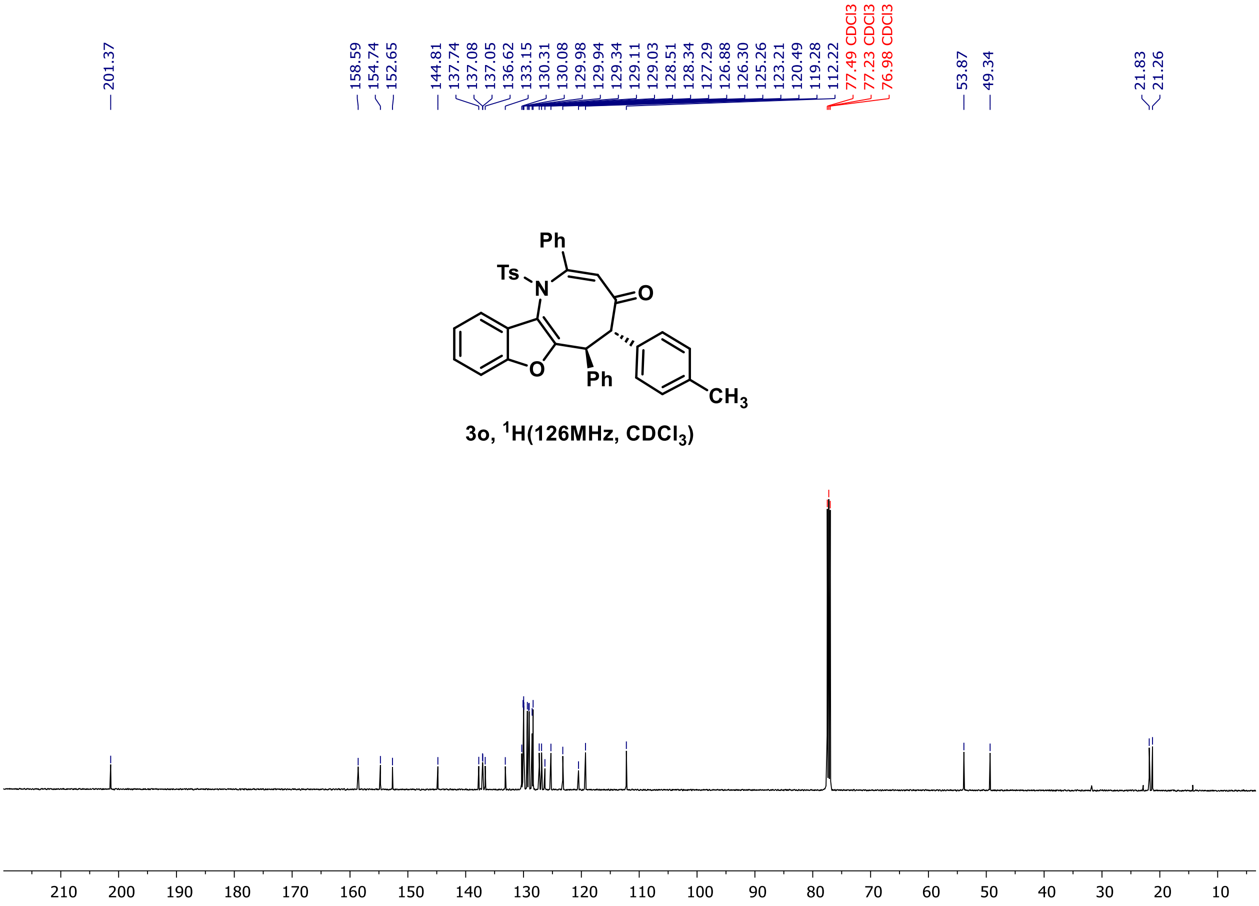

3o, ^{1}H(126MHz, $CDCl_3$)

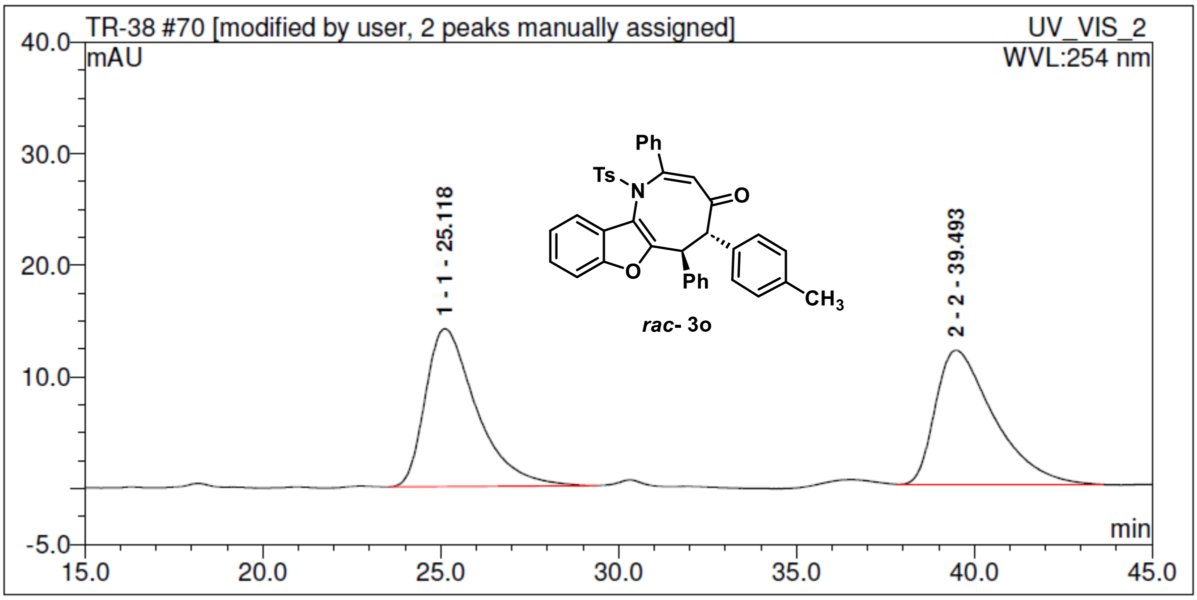

	Peak Name	Ret.Time (detected) min	Area mAU*min	Rel.Area(ident.) %	Height mAU	Amount
1	1	25.12	23.60194	50.65014694	14.15328	n.a.
2	2	39.49	22.996	49.34985306	12.043	n.a.

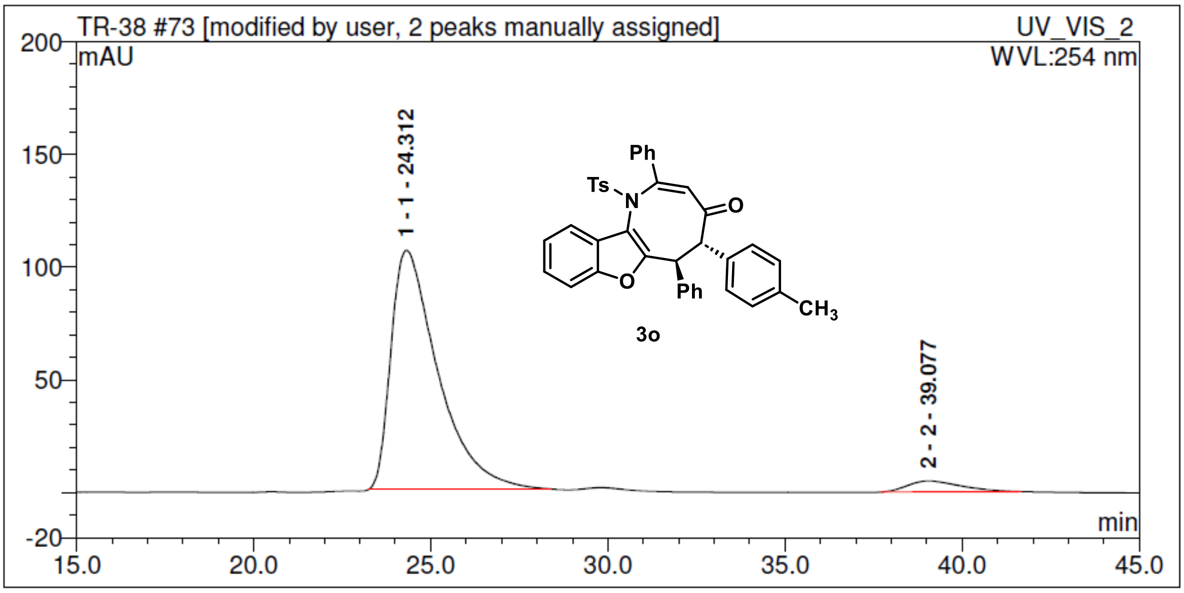

	Peak Name	Ret.Time (detected) min	Area mAU*min	Rel.Area(ident.) %	Height mAU	Amount
1	1	24.31	163.0002	95.13994582	106.054	n.a.
2	2	39.08	8.327	4.860054185	4.789	n.a.

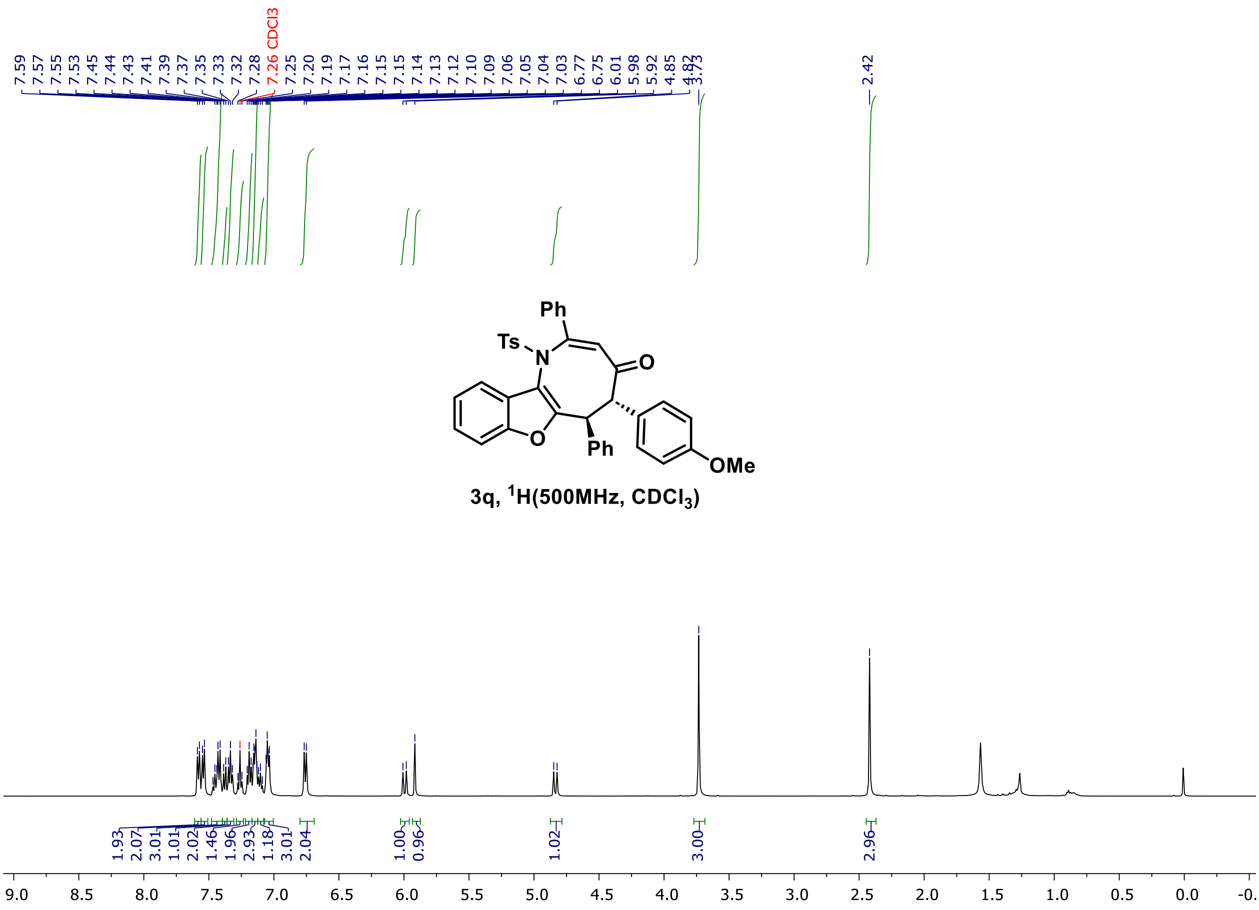
7.59 7.57 7.55 7.53 7.45 7.44 7.43 7.41 7.39 7.37 7.35 7.33 7.32 7.28 7.26 CDCl3 7.25 7.20 7.19 7.17 7.16 7.15 7.15 7.14 7.13 7.12 7.10 7.09 7.06 7.05 7.04 7.03 6.77 6.75 6.01 5.98 5.92 4.85 4.82 3.73
2.42
Ph
Ts
N
O
O
Ph
OMe
3q, 1H(500MHz, CDCl3)
1.93 2.07 3.01 1.01 2.02 1.46 1.96 2.93 1.18 3.01 2.04
1.00 0.96
1.02
3.00
2.96
9.0 8.5 8.0 7.5 7.0 6.5 6.0 5.5 5.0 4.5 4.0 3.5 3.0 2.5 2.0 1.5 1.0 0.5 0.0 -0.

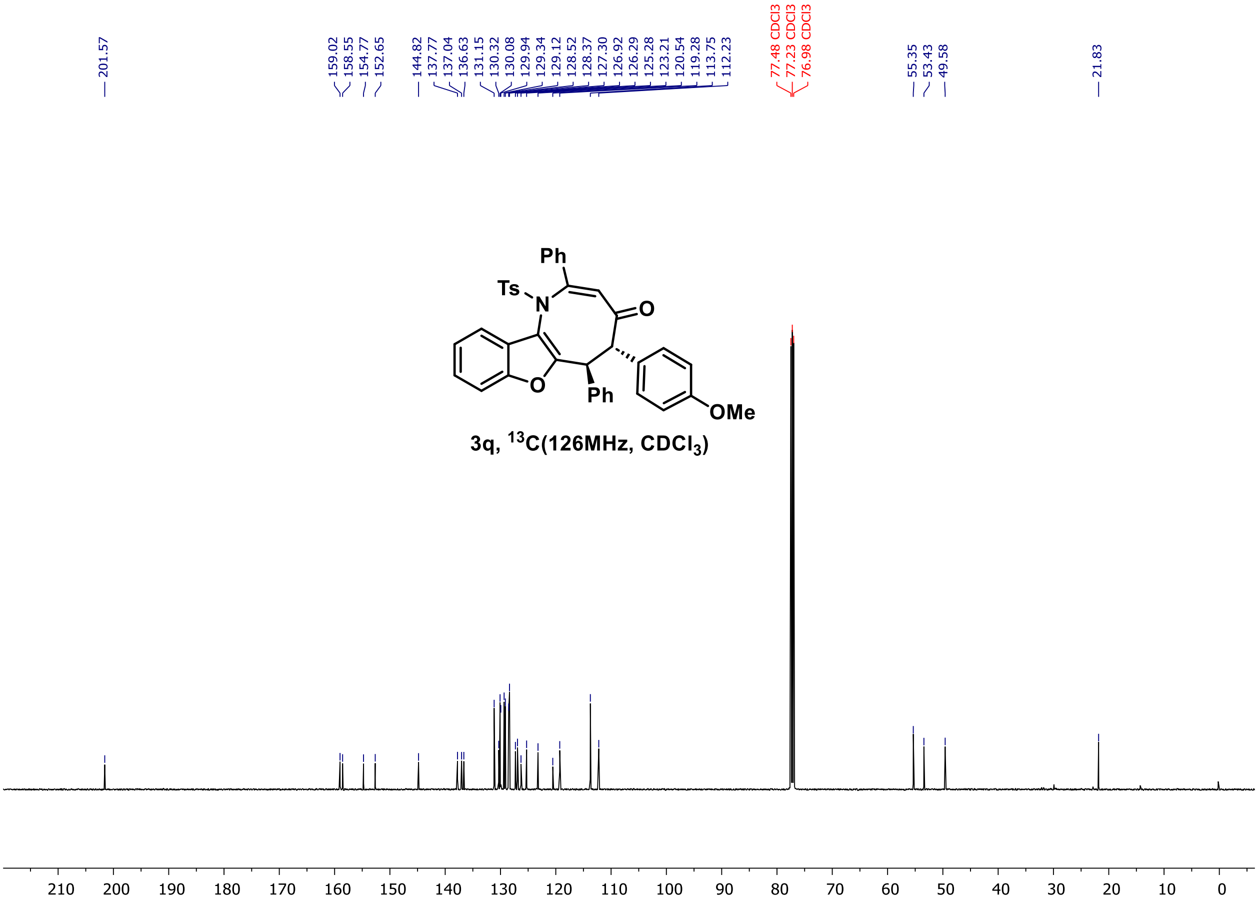
201.57
159.02 158.55 154.77 152.65
144.82 137.77 137.04 136.63 131.15 130.32 130.08 129.94 129.34 129.12 128.52 128.37 127.30 126.92 126.29 125.28 123.21 120.54 119.28 113.75 112.23
77.48 CDCl3 77.23 CDCl3 76.98 CDCl3
55.35 53.43 49.58
21.83
Ph
Ts
N
O
O
Ph
OMe
3q, 13C(126MHz, CDCl3)
210 200 190 180 170 160 150 140 130 120 110 100 90 80 70 60 50 40 30 20 10 0

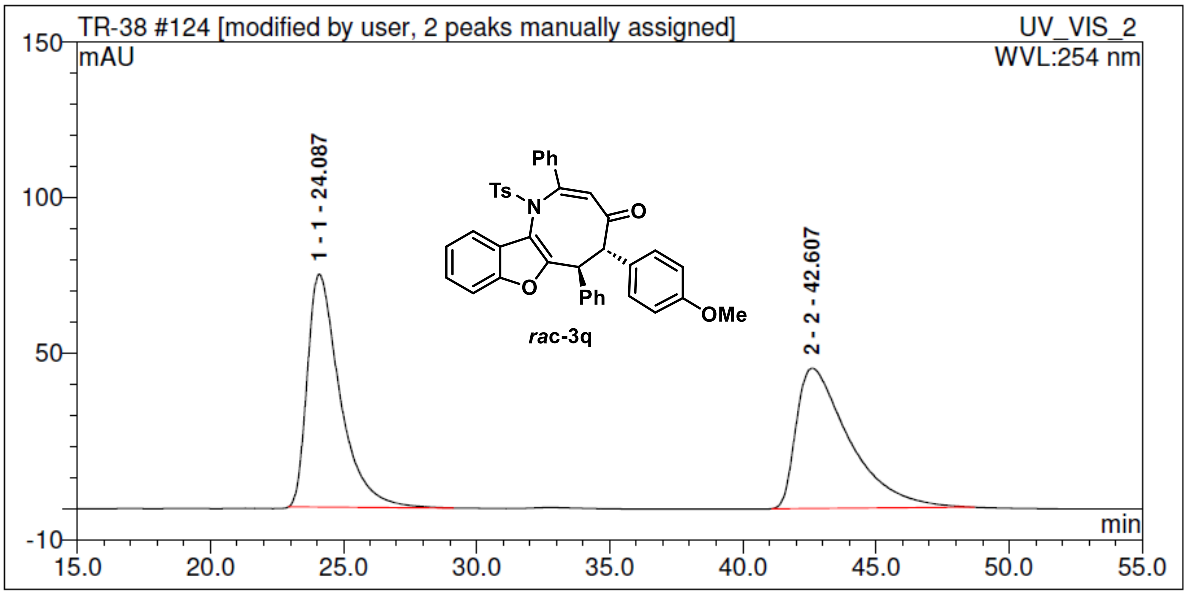

Peak	Name	Ret.Time (detected) min	Area mAU*min	Rel.Area(ident.) %	Height mAU	Amount
1	1	24.09	106.4465	50.45145325	74.91073	n.a.
2	2	42.61	104.541	49.54854675	45.075	n.a.

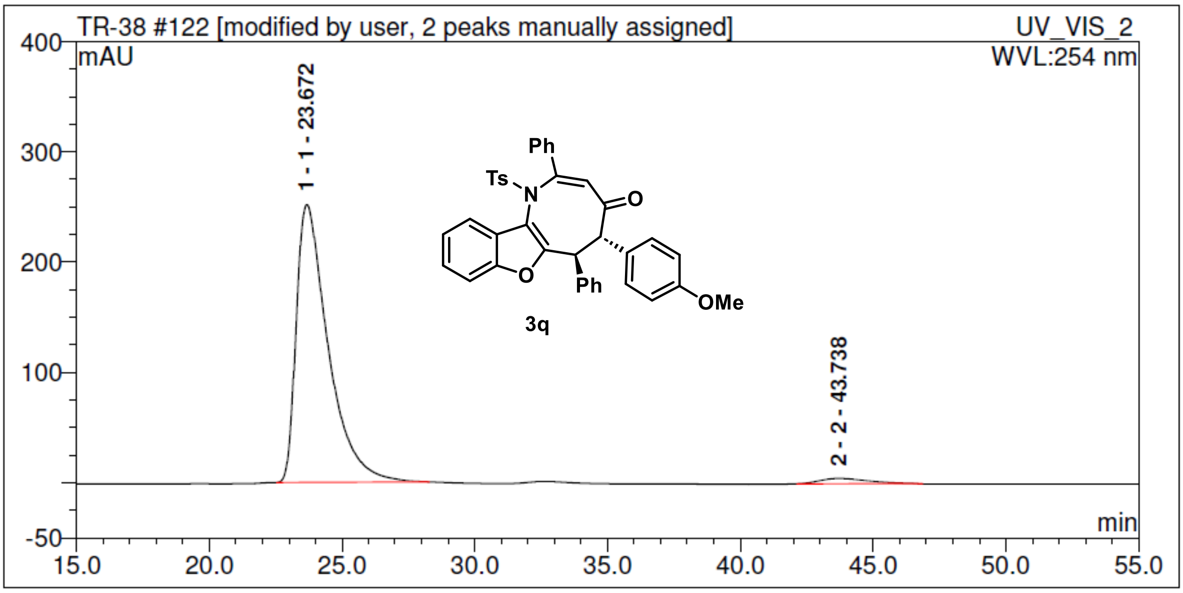

Peak	Name	Ret.Time (detected) min	Area mAU*min	Rel.Area(ident.) %	Height mAU	Amount
1	1	23.67	350.5285	97.243201	251.7992	n.a.
2	2	43.74	9.937	2.756799	4.662	n.a.

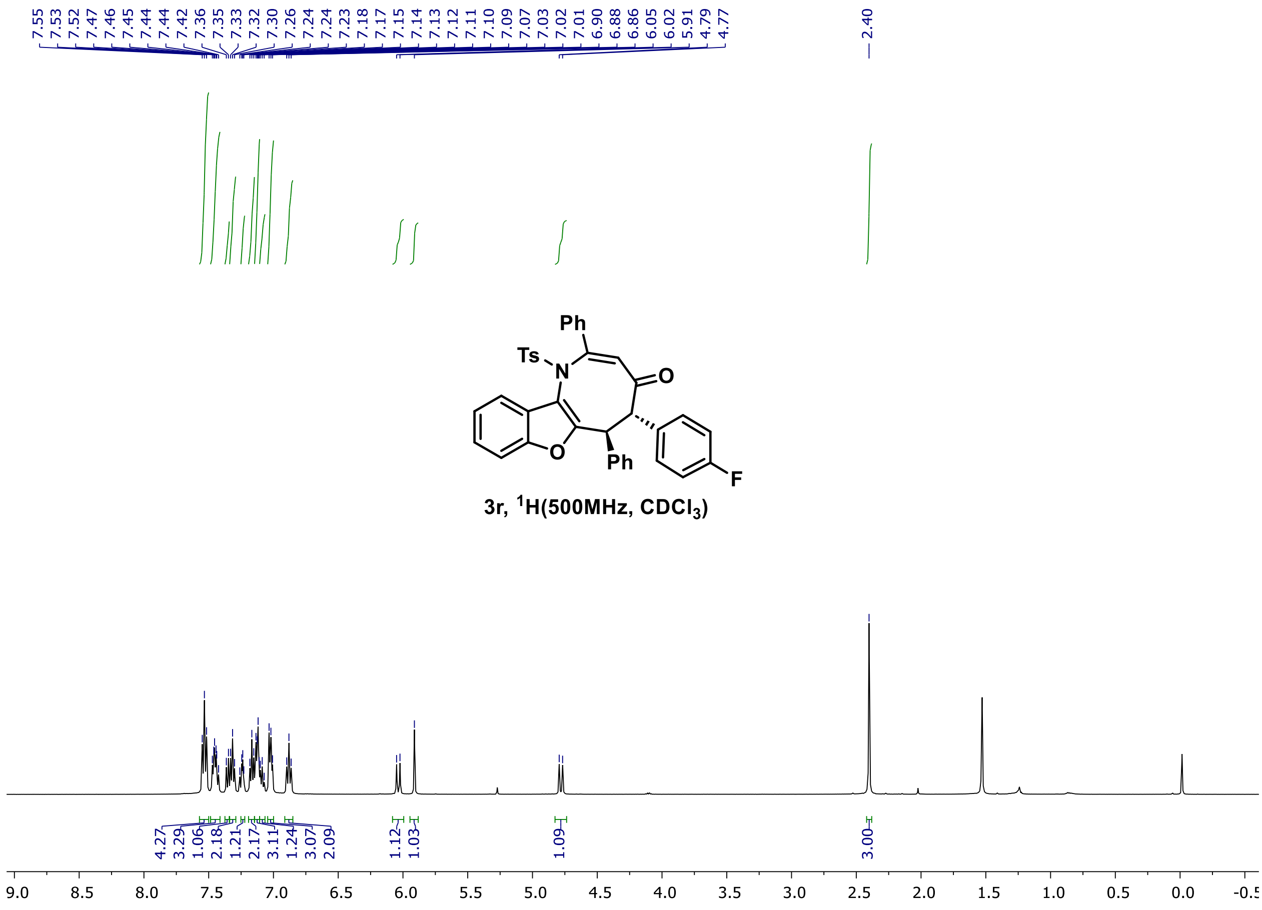

3r, ^{1}H(500MHz, $CDCl_3$)

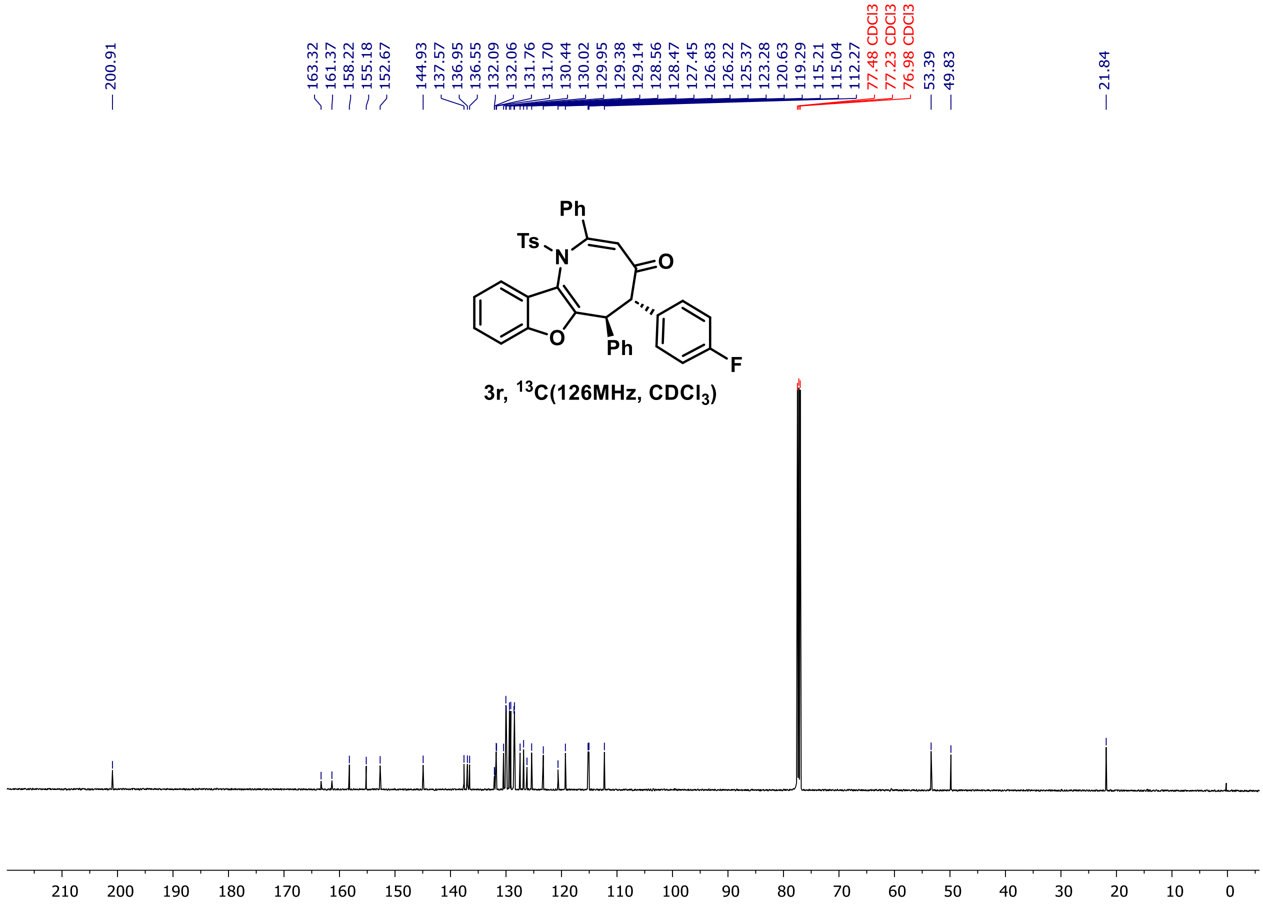

3r, ^{13}C(126MHz, $CDCl_3$)

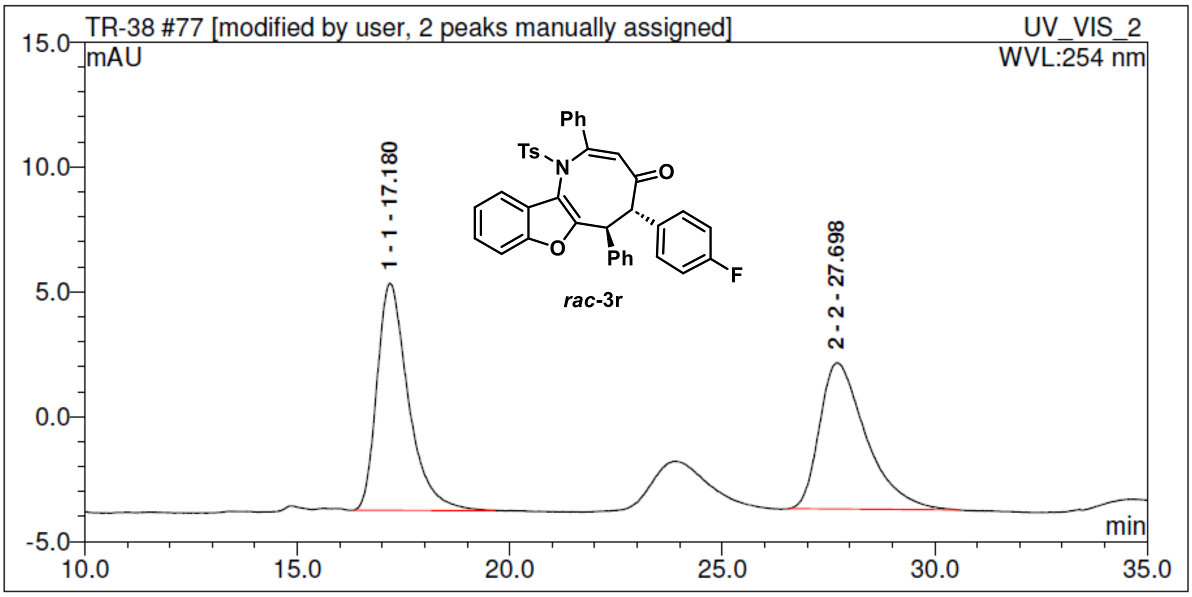

	Peak Name	Ret.Time (detected) min	Area mAU*min	Rel.Area(ident.) %	Height mAU	Amount
1	1	17.18	7.805655	51.11440283	9.09754	n.a.
2	2	27.70	7.465	48.88559717	5.857	n.a.

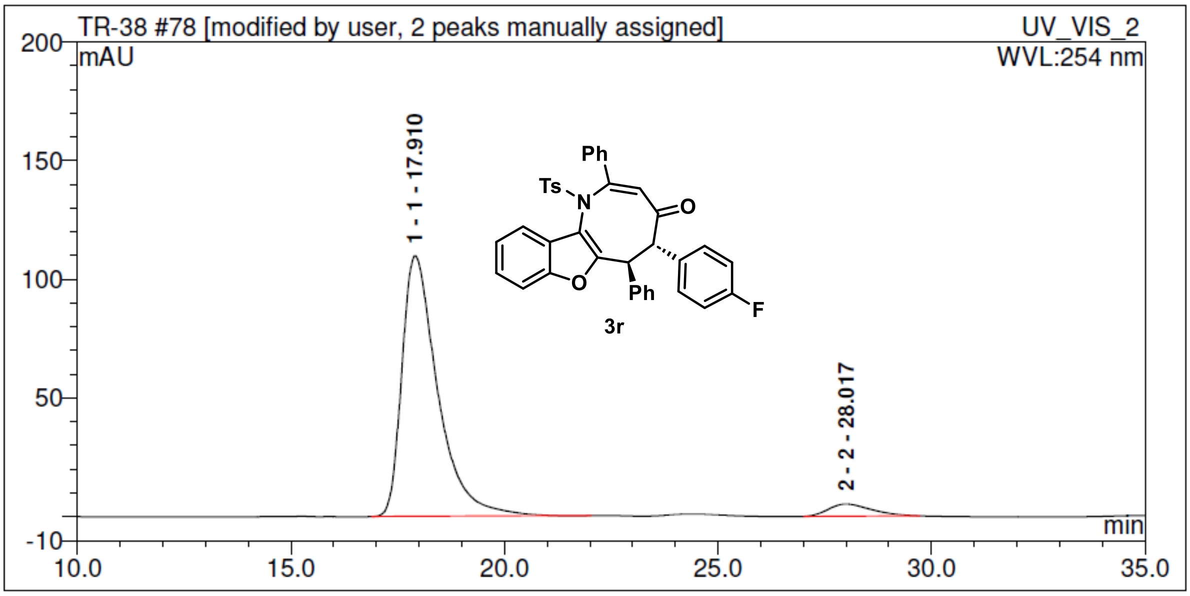

	Peak Name	Ret.Time (detected) min	Area mAU*min	Rel.Area(ident.) %	Height mAU	Amount
1	1	17.91	106.6434	94.74286942	109.7575	n.a.
2	2	28.02	5.917	5.257130585	5.076	n.a.

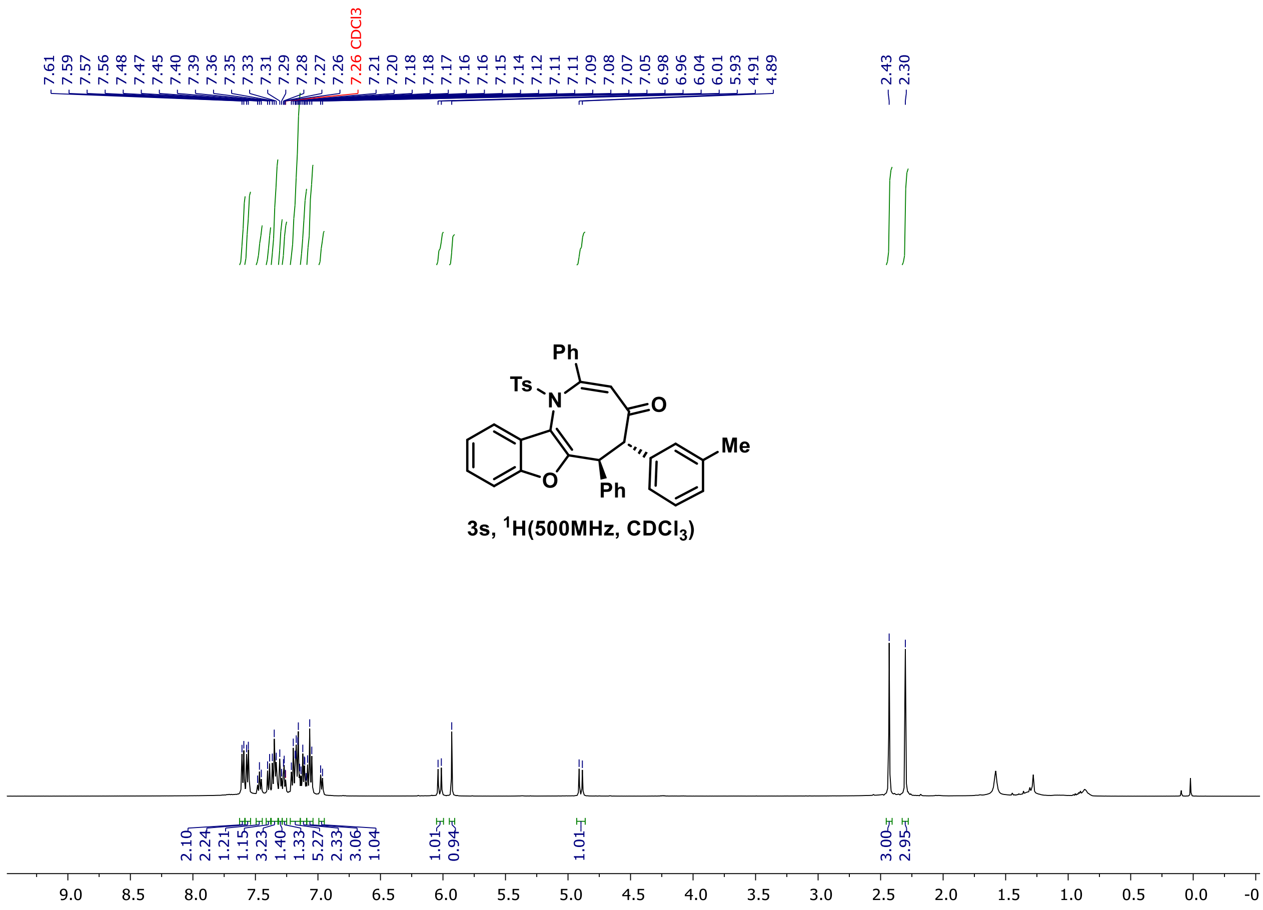

3s, ^{1}H(500MHz, $CDCl_3$)

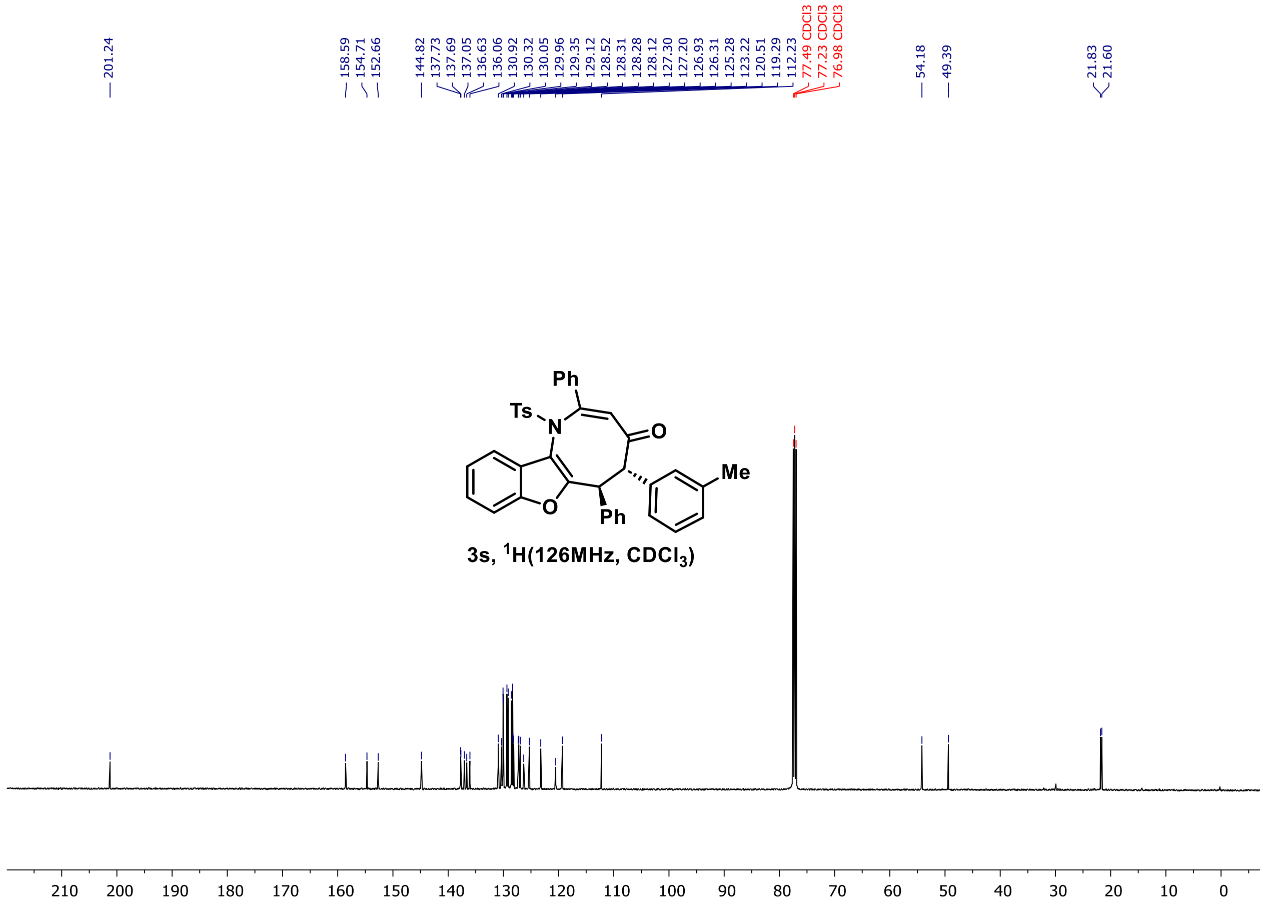

3s, ^{1}H(126MHz, $CDCl_3$)

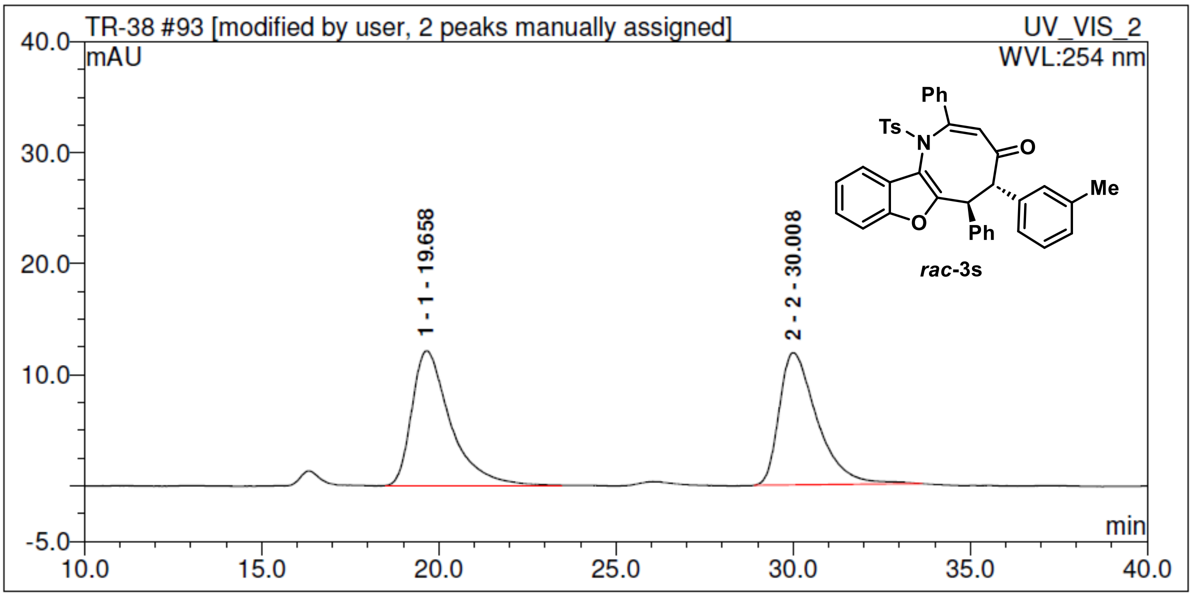

	Peak Name	Ret.Time (detected) min	Area mAU*min	Rel.Area(ident.) %	Height mAU	Amount
1	1	19.66	15.23536	50.71244921	12.11948	n.a.
2	2	30.01	14.807	49.28755079	11.902	n.a.

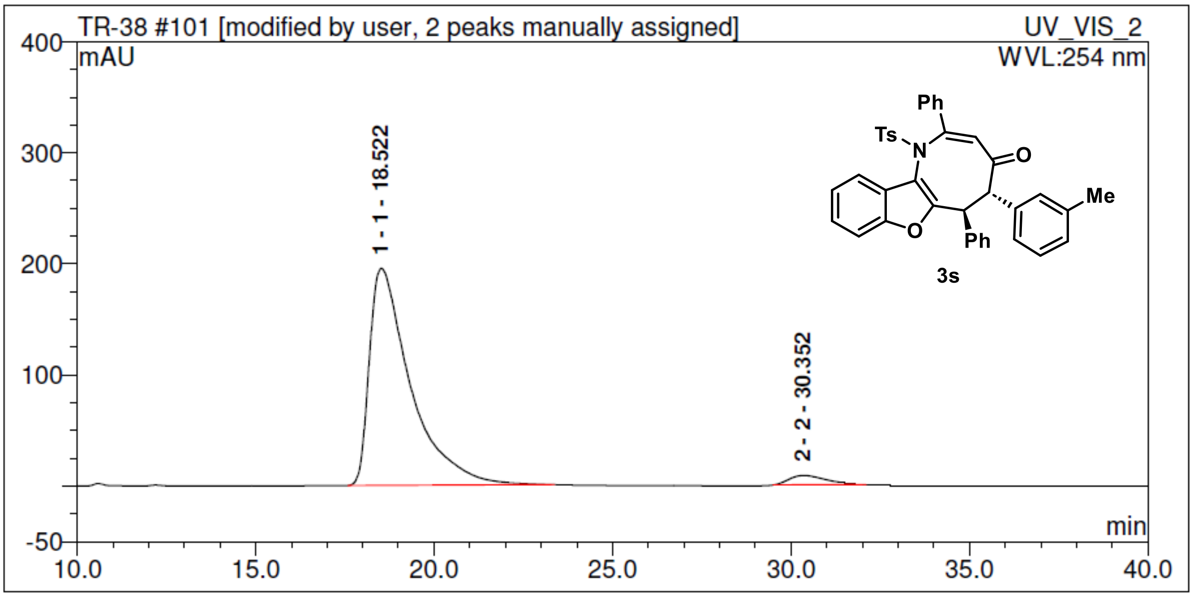

	Peak Name	Ret.Time (detected) min	Area mAU*min	Rel.Area(ident.) %	Height mAU	Amount
1	1	18.52	260.983	96.24843256	195.3939	n.a.
2	2	30.35	10.173	3.751567444	8.493	n.a.

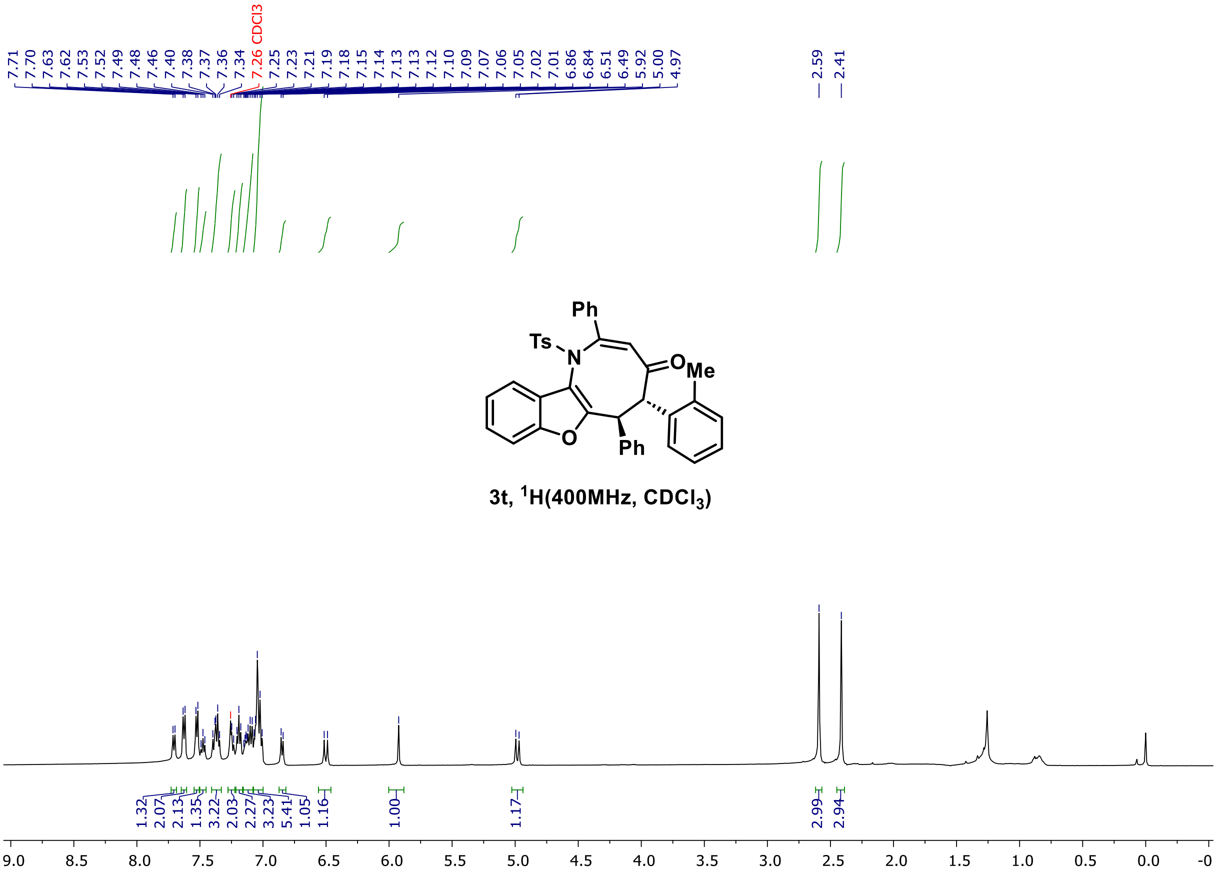

3t, ^{1}H(400MHz, $CDCl_3$)

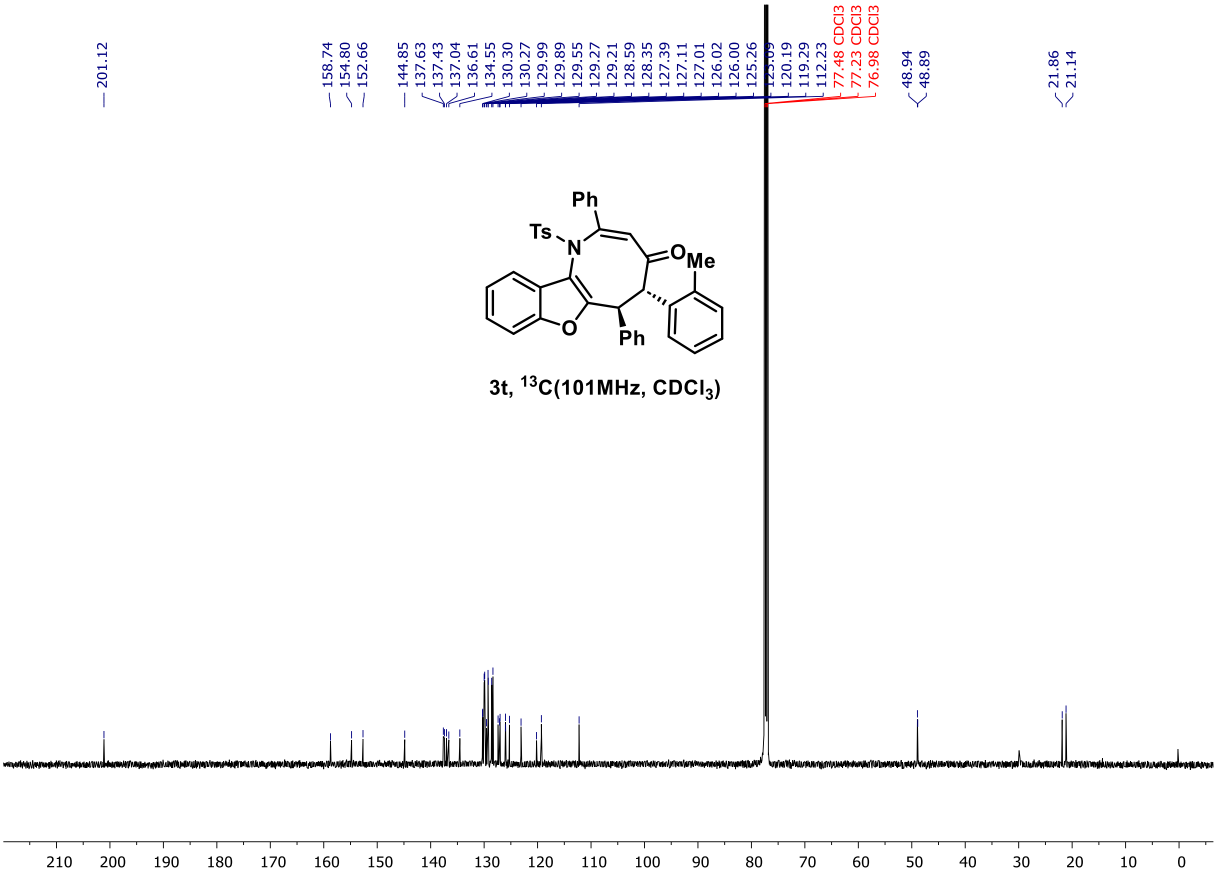

3t, ^{13}C(101MHz, $CDCl_3$)

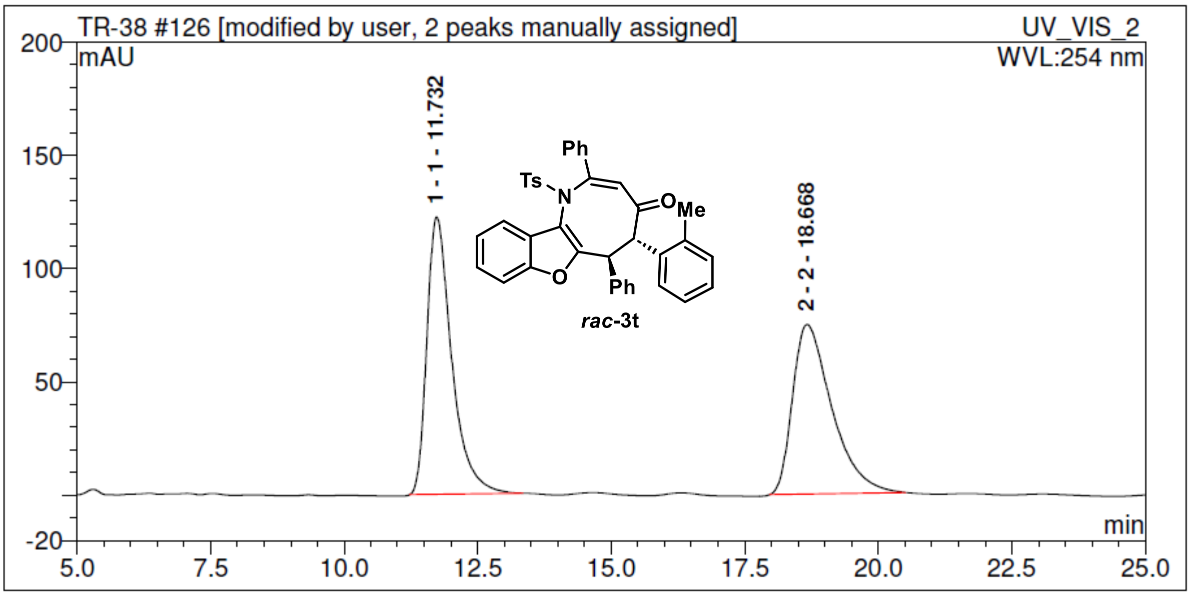

	Peak Name	Ret.Time (detected) min	Area mAU*min	Rel.Area(ident.) %	Height mAU	Amount
1	1	11.73	65.95974	50.83827517	122.3954	n.a.
2	2	18.67	63.785	49.16172483	74.836	n.a.

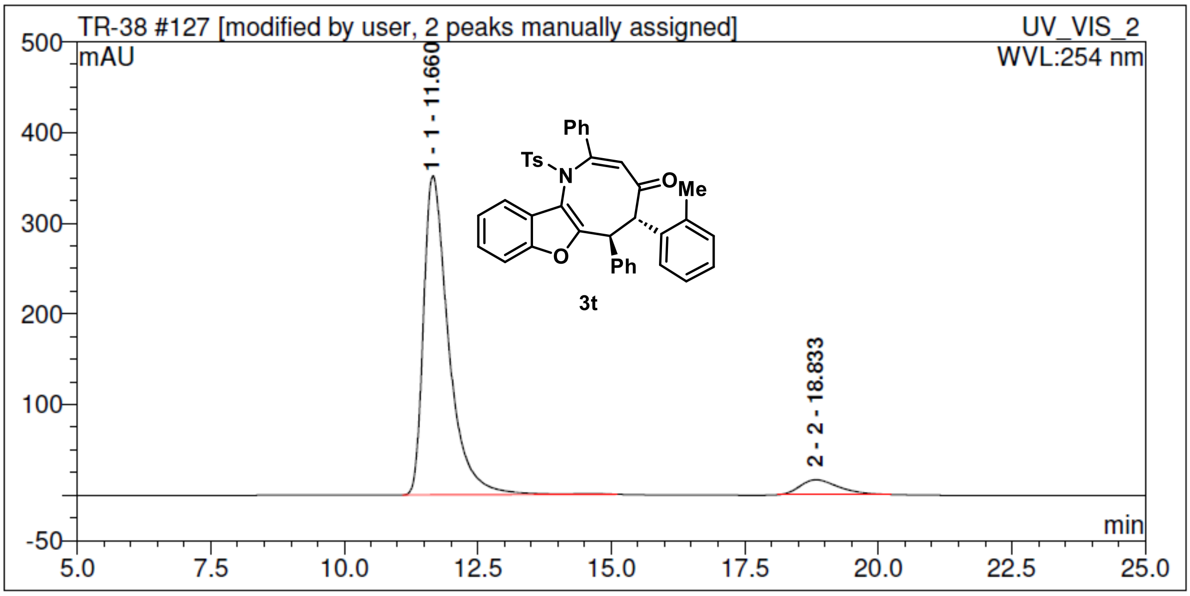

	Peak Name	Ret.Time (detected) min	Area mAU*min	Rel.Area(ident.) %	Height mAU	Amount
1	1	11.66	192.1611	93.3678117	351.8106	n.a.
2	2	18.83	13.650	6.632188304	16.314	n.a.

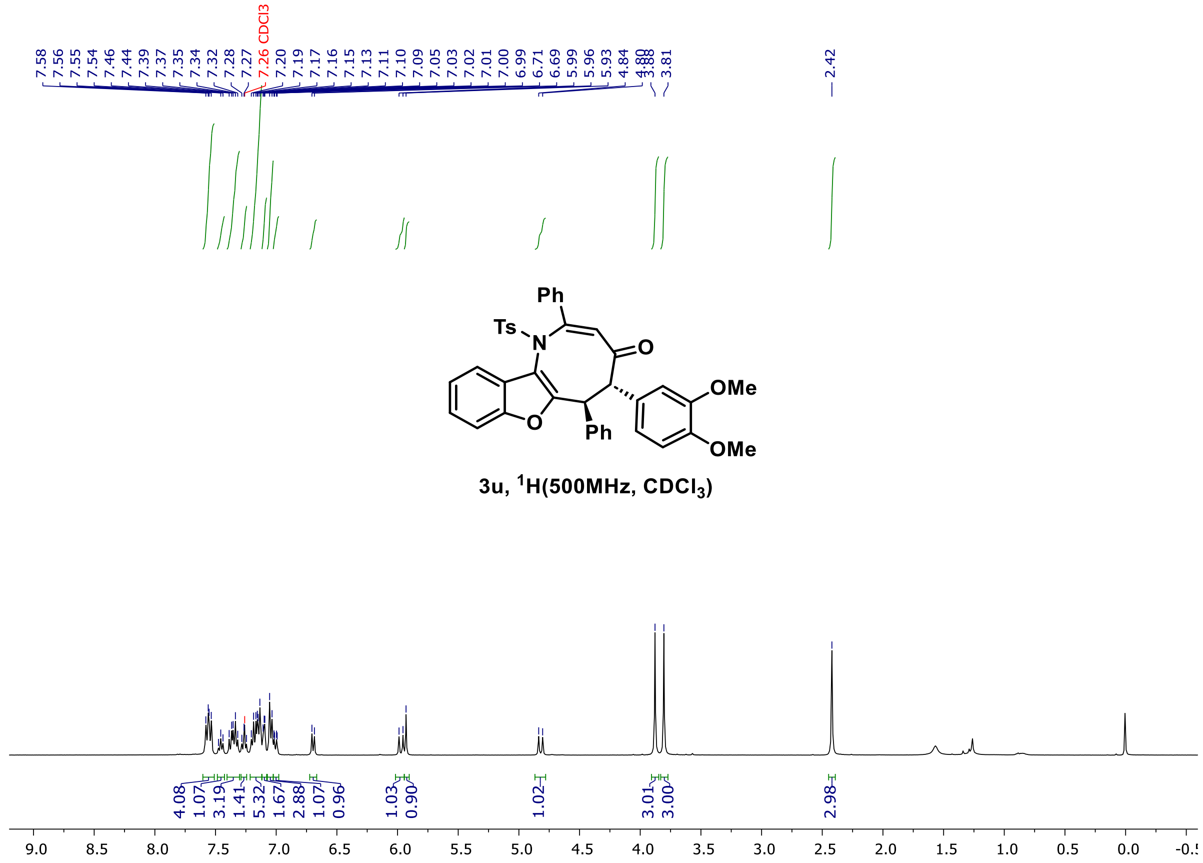

3u, ^{1}H(500MHz, $CDCl_3$)

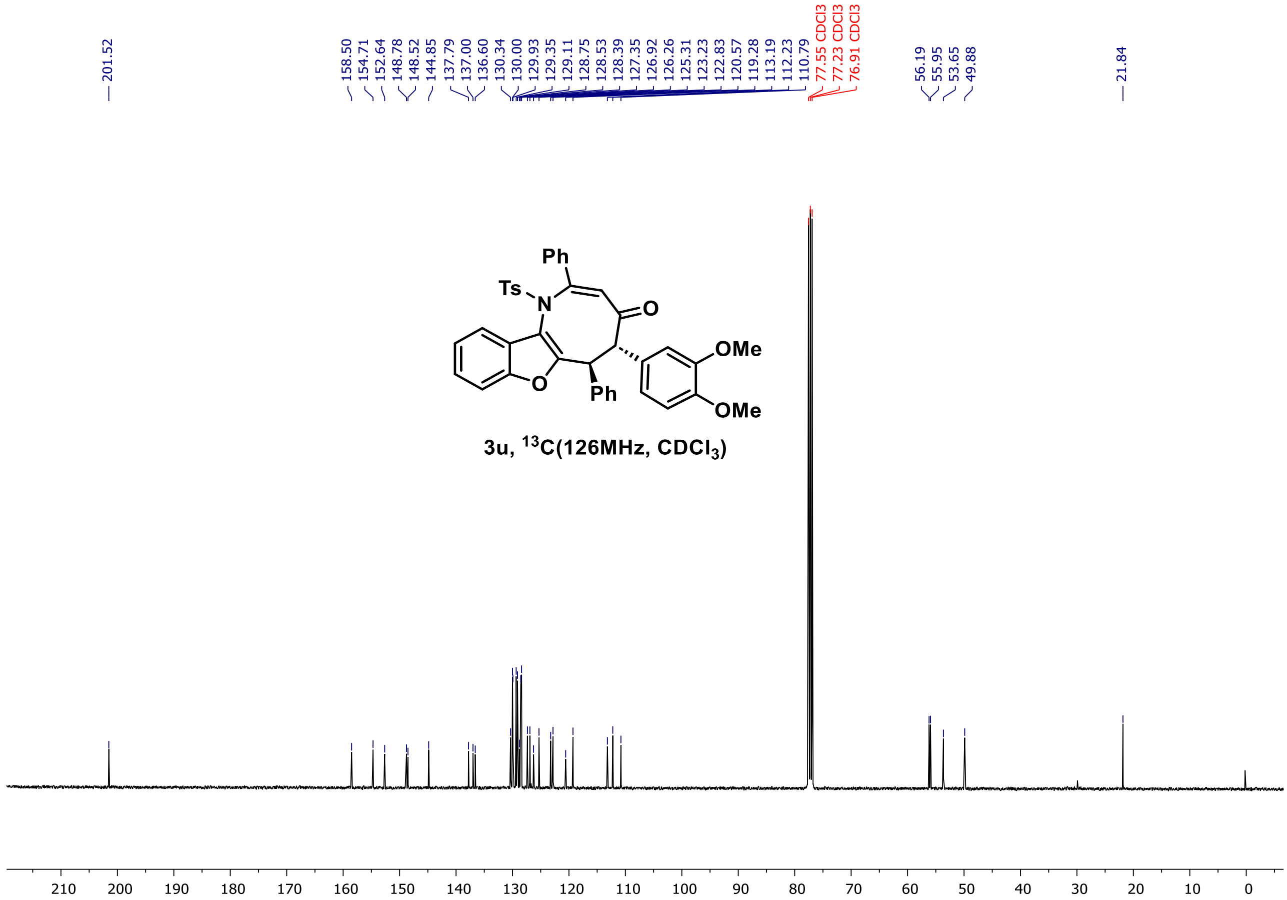

3u, ^{13}C(126MHz, $CDCl_3$)

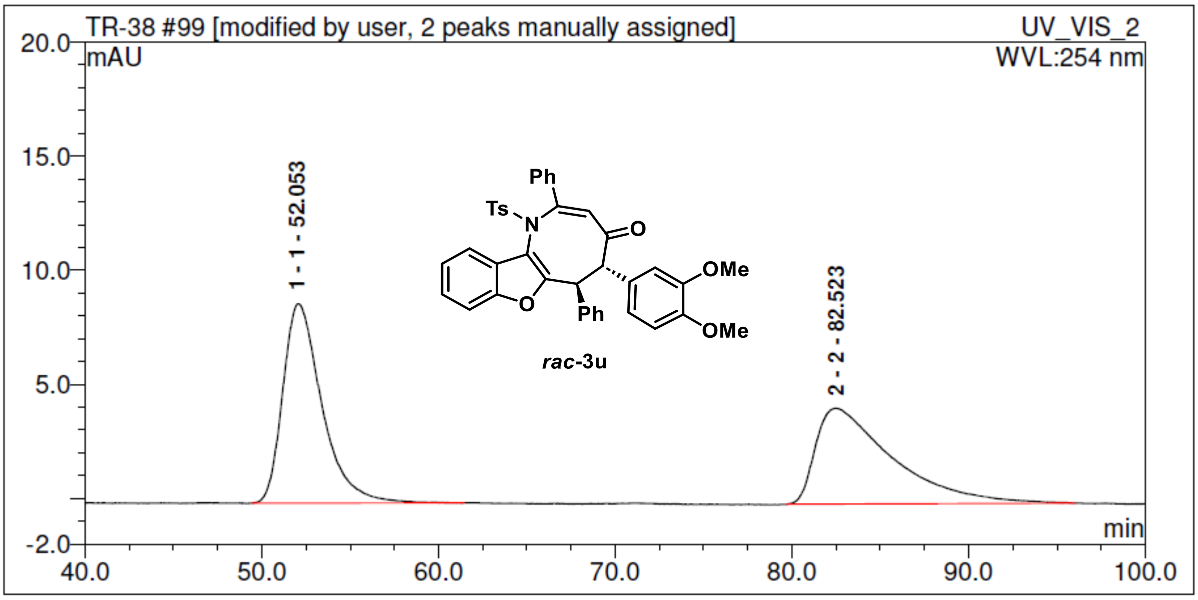

Peak Name		Ret.Time (detected) min	Area mAU*min	Rel.Area(ident.) %	Height mAU	Amount
1	1	52.05	22.46386	51.58048786	8.73886	n.a.
2	2	82.52	21.087	48.41951214	4.202	n.a.

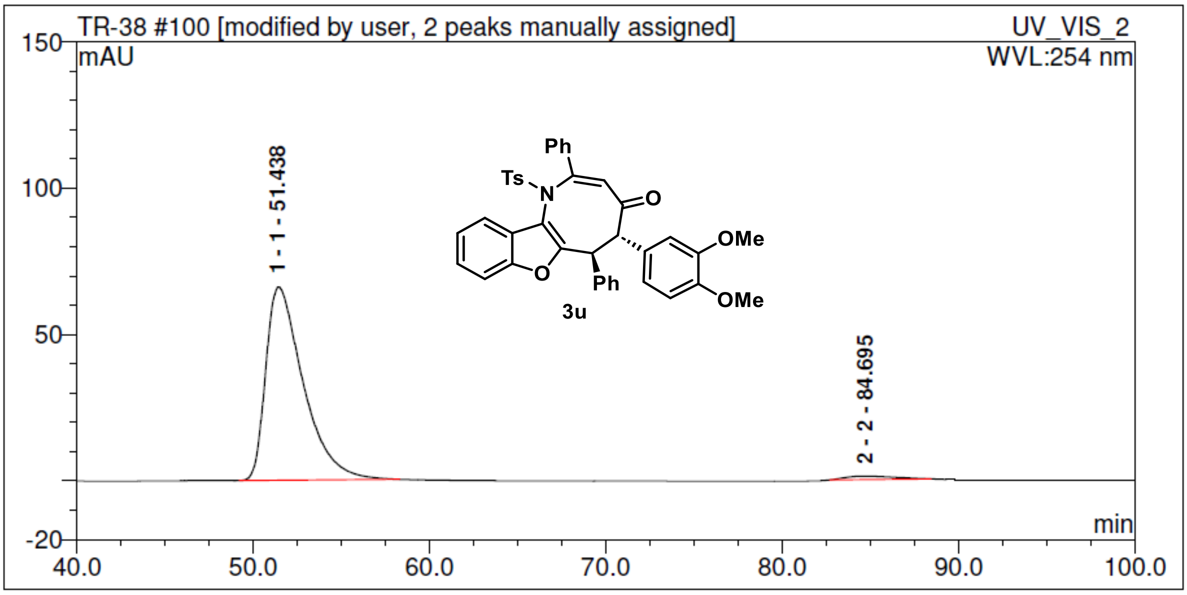

Peak Name		Ret.Time (detected) min	Area mAU*min	Rel.Area(ident.) %	Height mAU	Amount
1	1	51.44	163.0721	97.66420204	66.05943	n.a.
2	2	84.70	3.900	2.33579796	1.208	n.a.

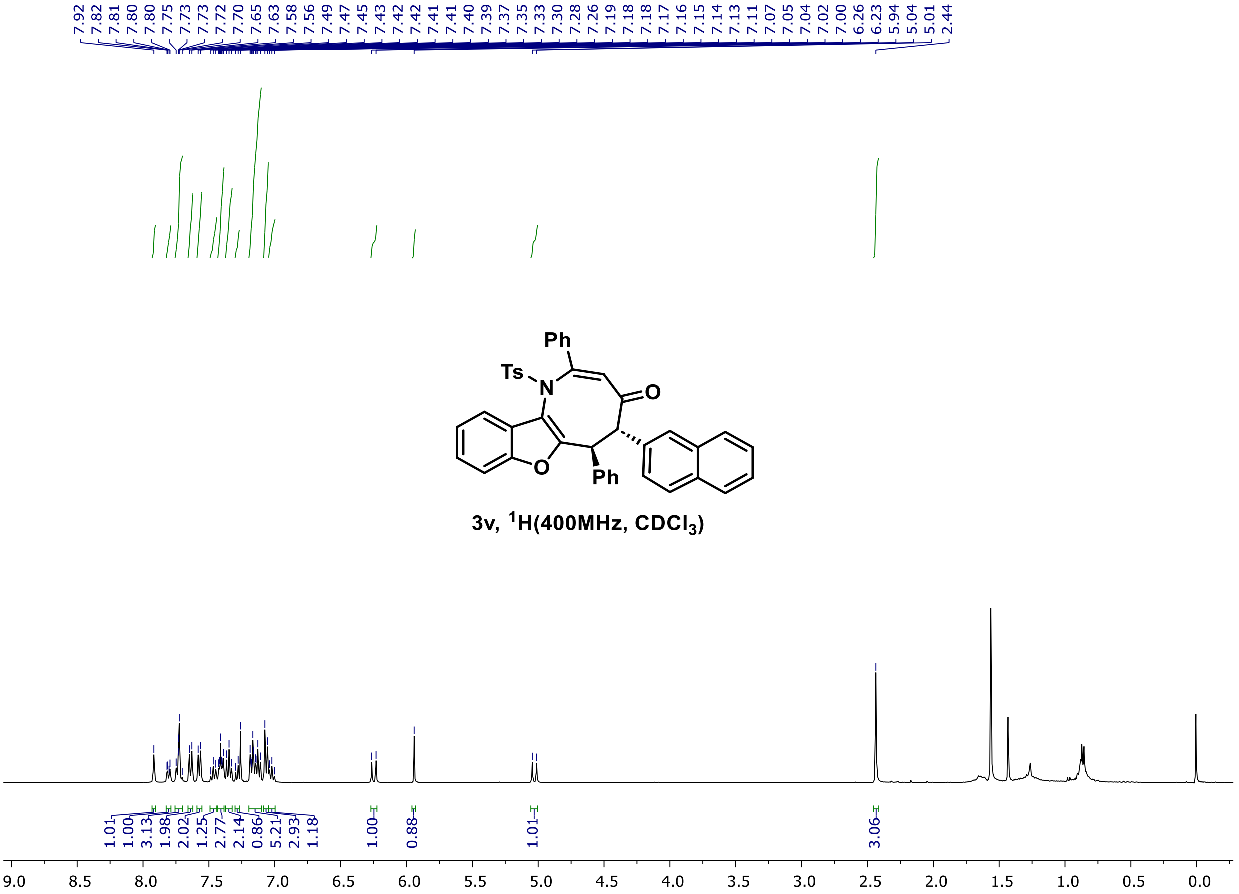

3v, ^{1}H(400MHz, CDCl$_3$)

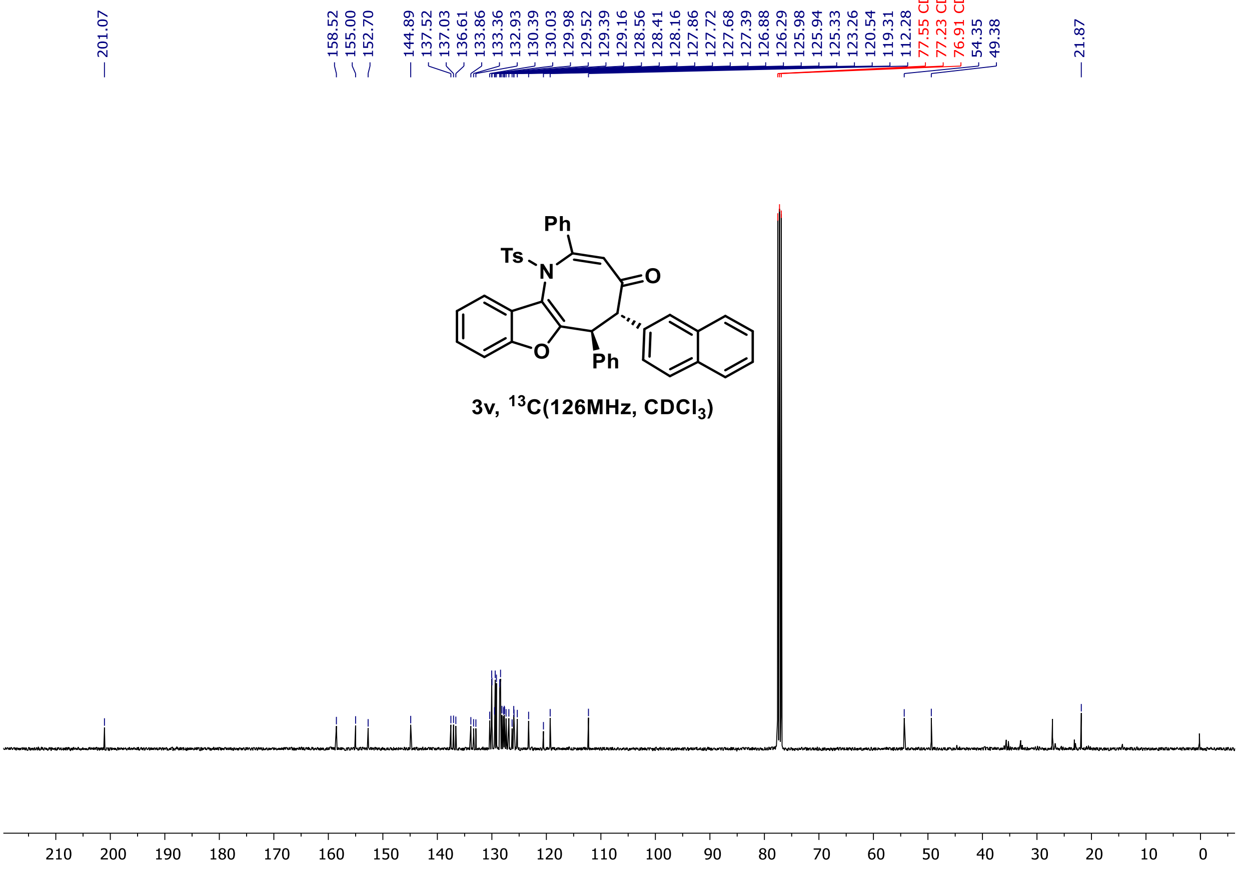

3v, ^{13}C(126MHz, CDCl$_3$)

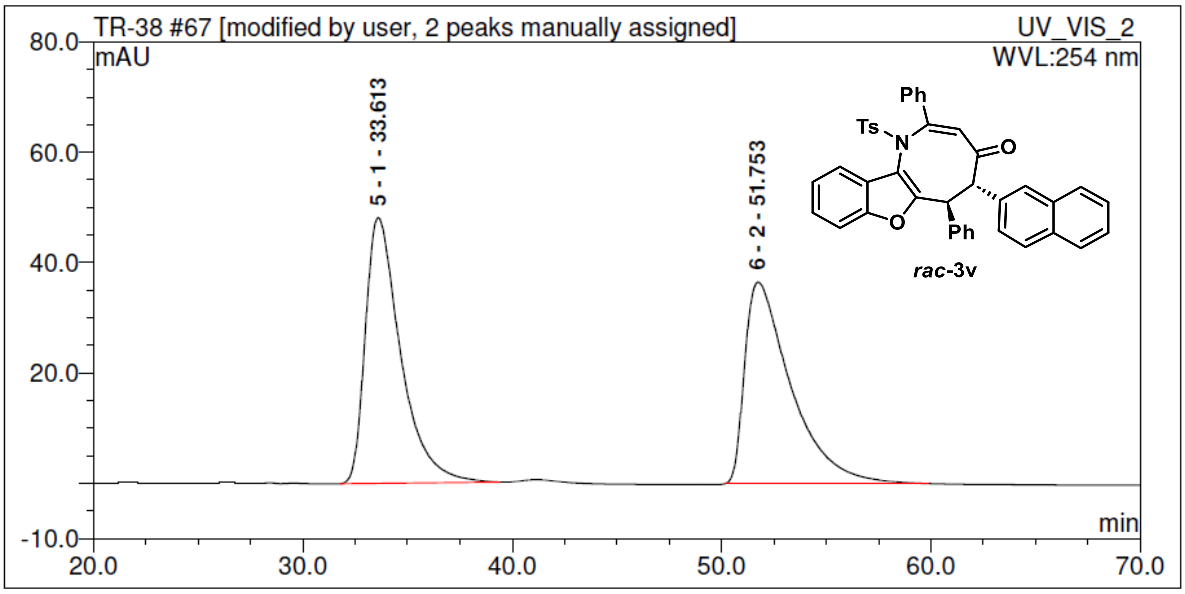

	Peak Name	Ret.Time (detected) min	Area mAU*min	Rel.Area(ident.) %	Height mAU	Amount
5	1	33.61	94.11407	50.21493189	48.17318	n.a.
6	2	51.75	93.308	49.78505894	36.518	n.a.

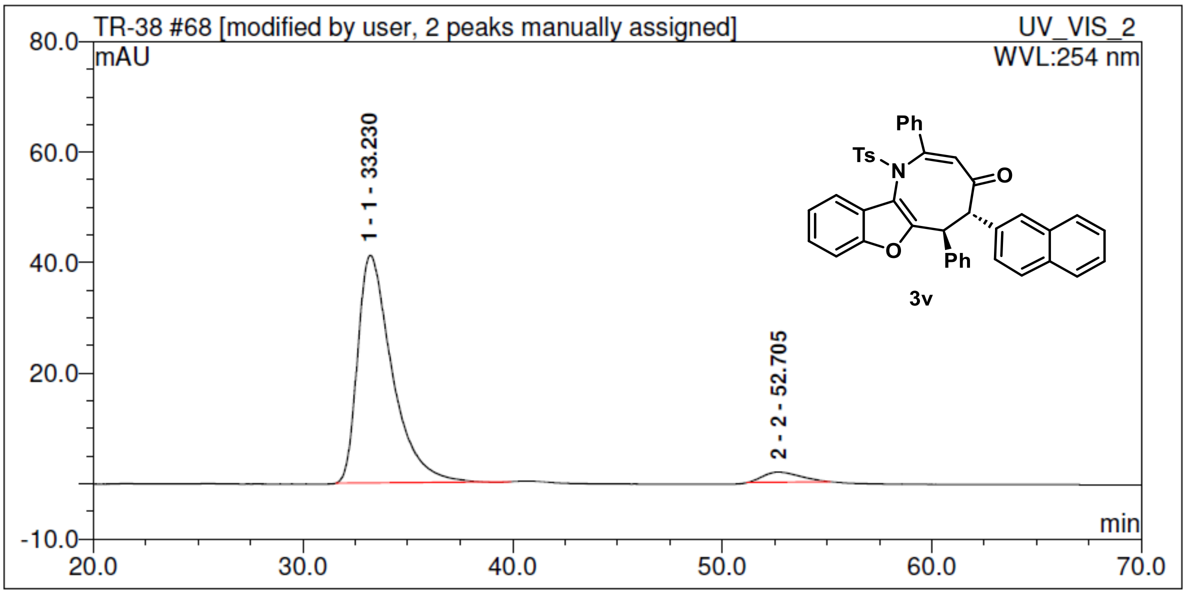

	Peak Name	Ret.Time (detected) min	Area mAU*min	Rel.Area(ident.) %	Height mAU	Amount
1	1	33.23	78.38773	95.40113695	41.1868	n.a.
2	2	52.71	3.779	4.598863047	1.845	n.a.

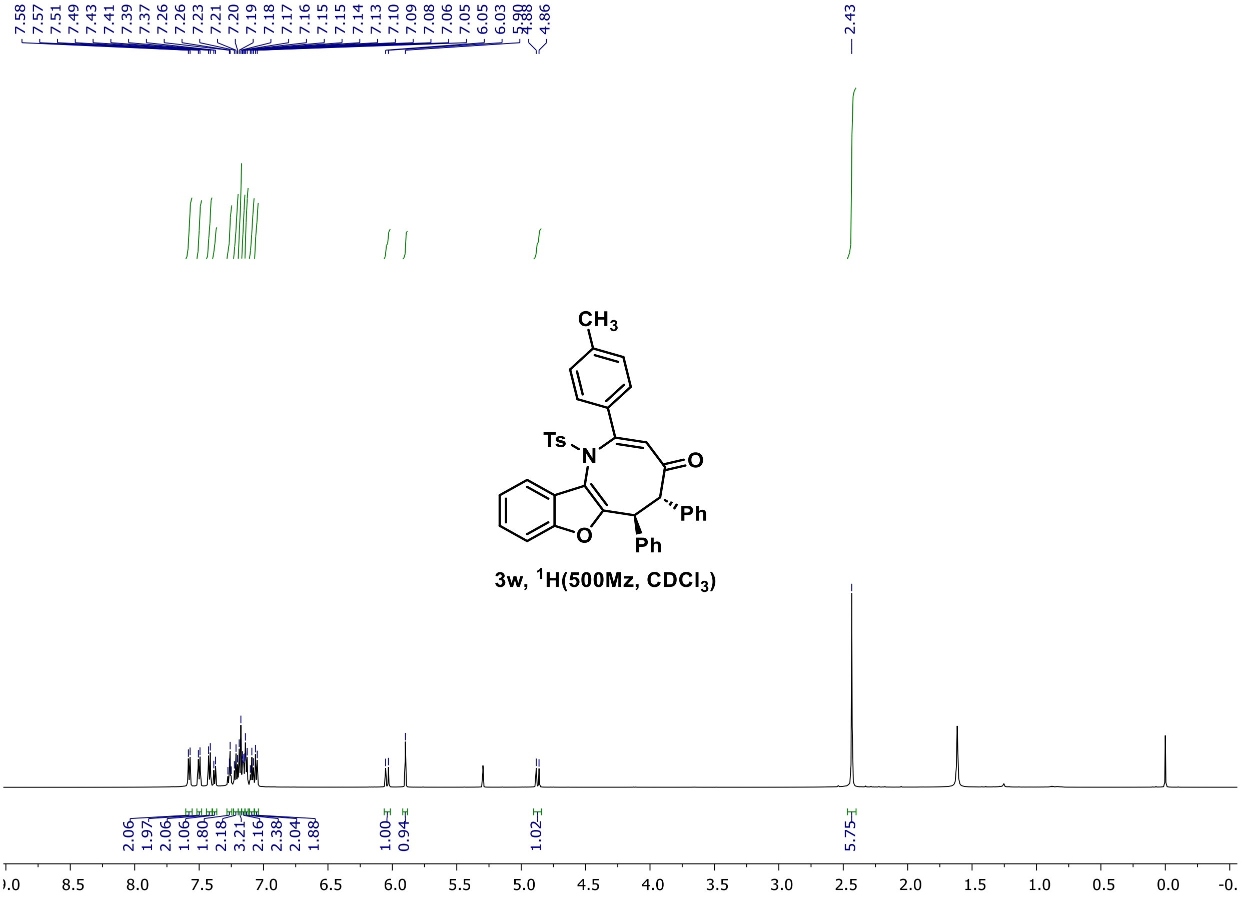
3w, 1H(500Mz, CDCl3)

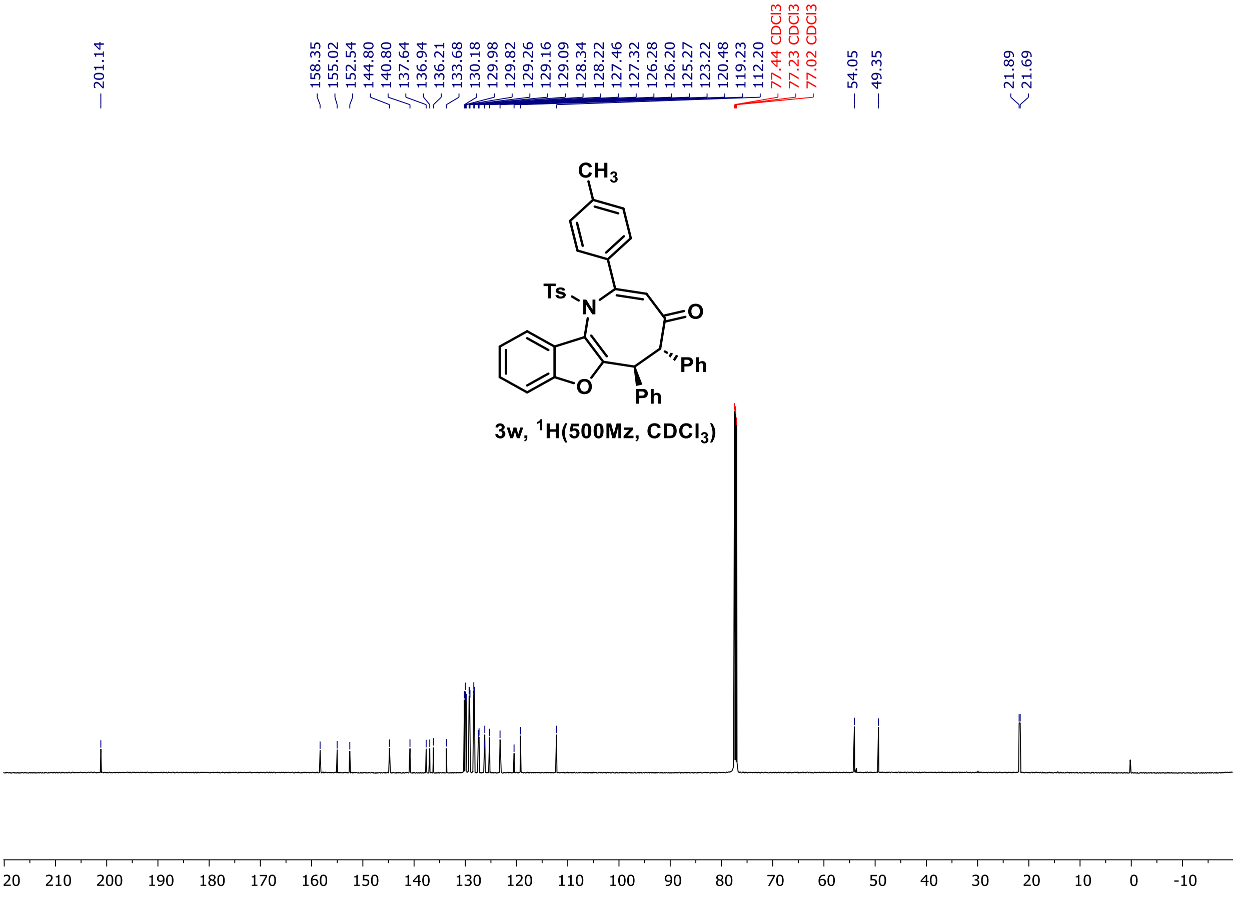
3w, 1H(500Mz, CDCl3)

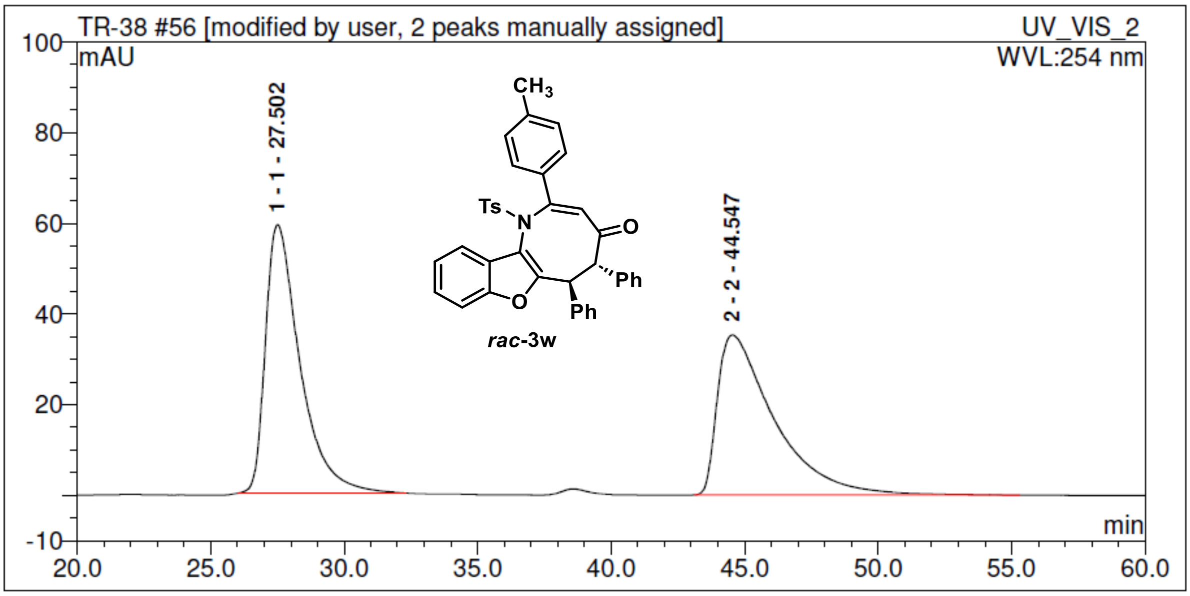

	Peak Name	Ret.Time (detected) min	Area mAU*min	Rel.Area(ident.) %	Height mAU	Amount
1	1	27.50	88.88218	50.49432129	59.23712	n.a.
2	2	44.55	87.142	49.50567871	35.281	n.a.

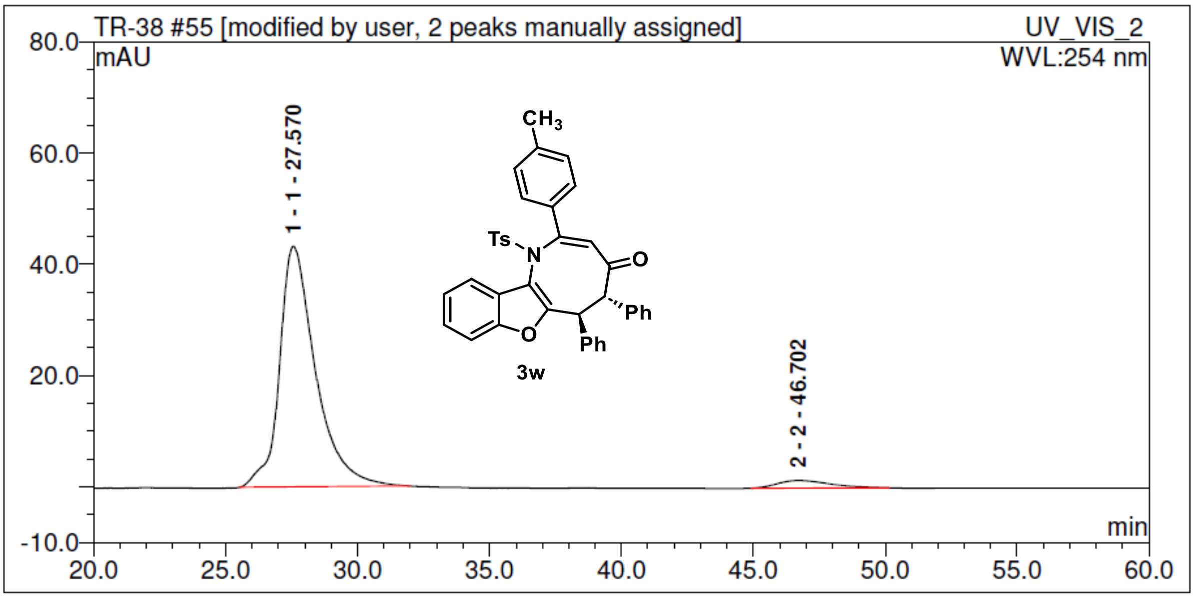

	Peak Name	Ret.Time (detected) min	Area mAU*min	Rel.Area(ident.) %	Height mAU	Amount
1	1	27.57	68.64275	95.63727645	43.30863	n.a.
2	2	46.70	3.131	4.362723555	1.348	n.a.

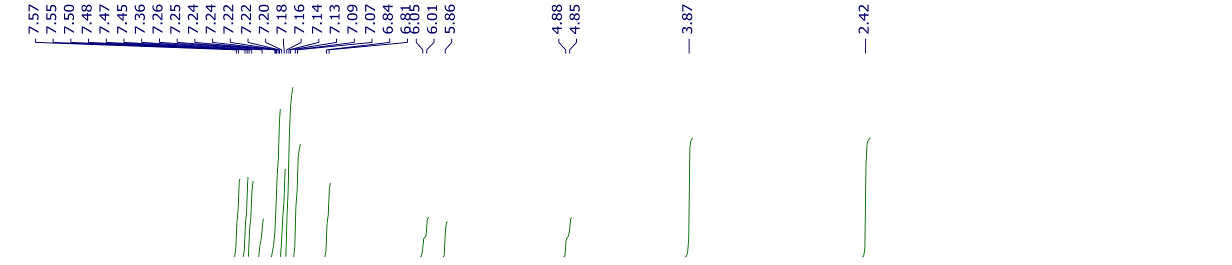

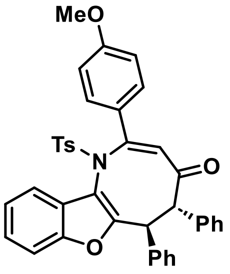

3x, ^{1}H(500MHz, $CDCl_3$)

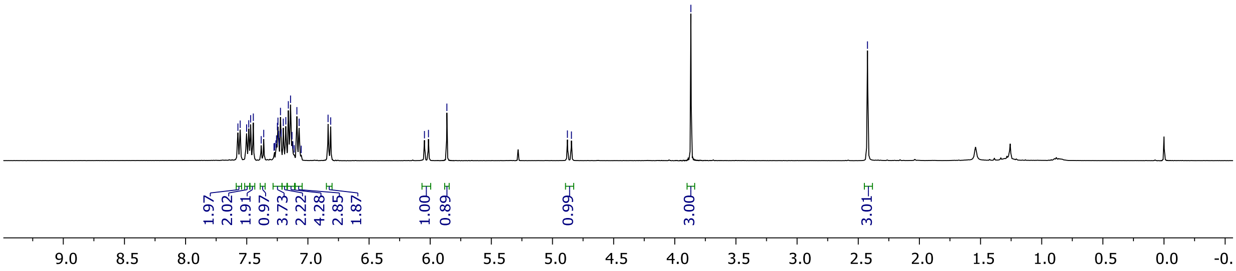

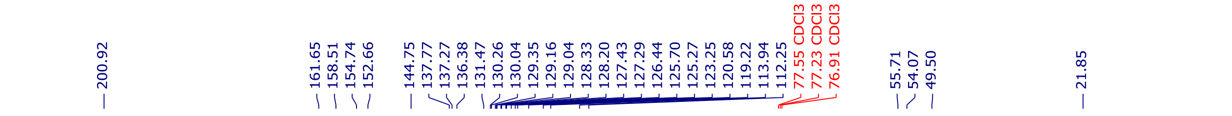

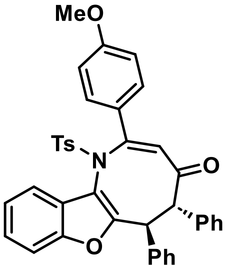

3x, ^{13}C(126MHz, $CDCl_3$)

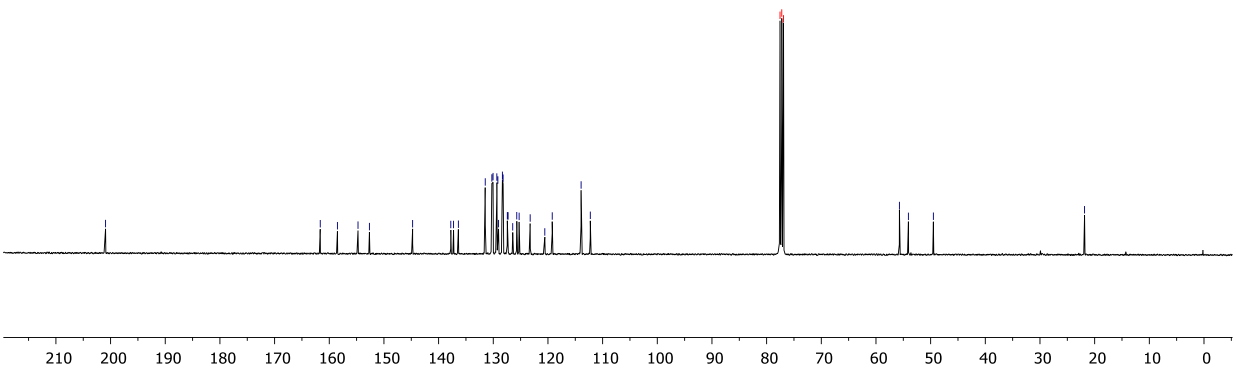

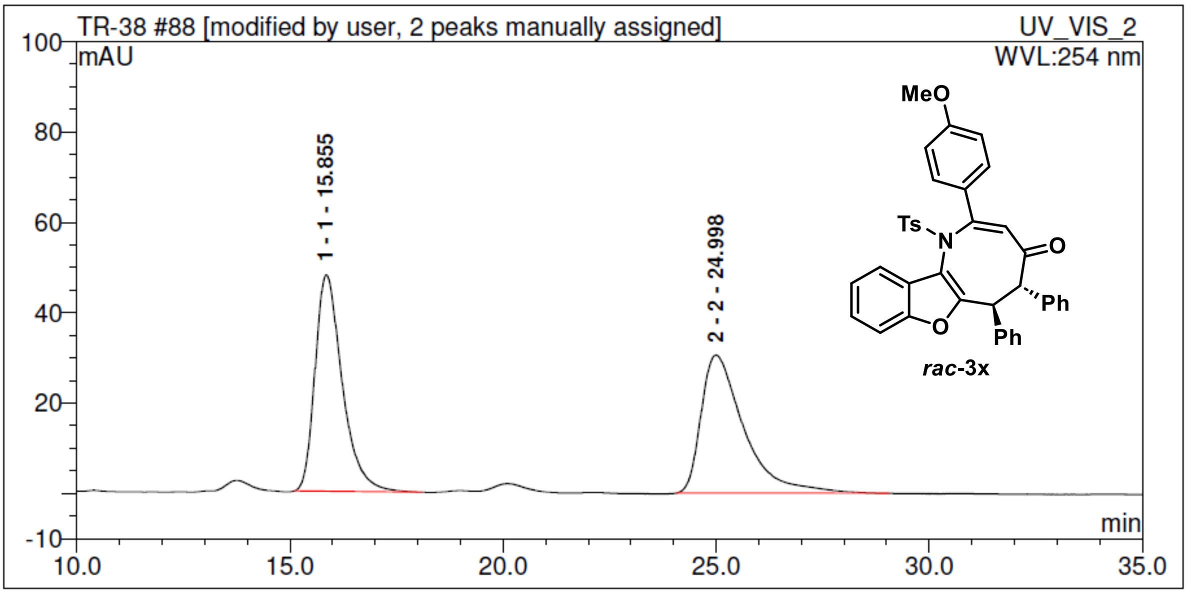

	Peak Name	Ret.Time (detected) min	Area mAU*min	Rel.Area(ident.) %	Height mAU	Amount
1	1	15.86	35.54304	49.98616004	47.90847	n.a.
2	2	25.00	35.563	50.01383996	30.515	n.a.

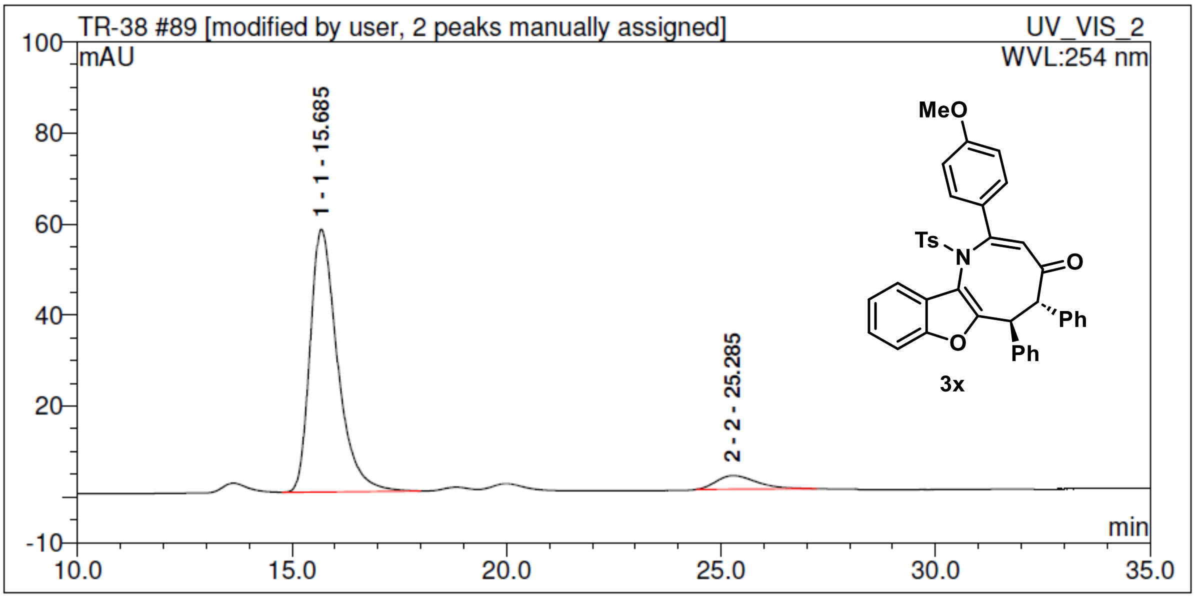

	Peak Name	Ret.Time (detected) min	Area mAU*min	Rel.Area(ident.) %	Height mAU	Amount
1	1	15.69	42.81368	92.97901971	57.73141	n.a.
2	2	25.29	3.233	7.020980292	2.984	n.a.

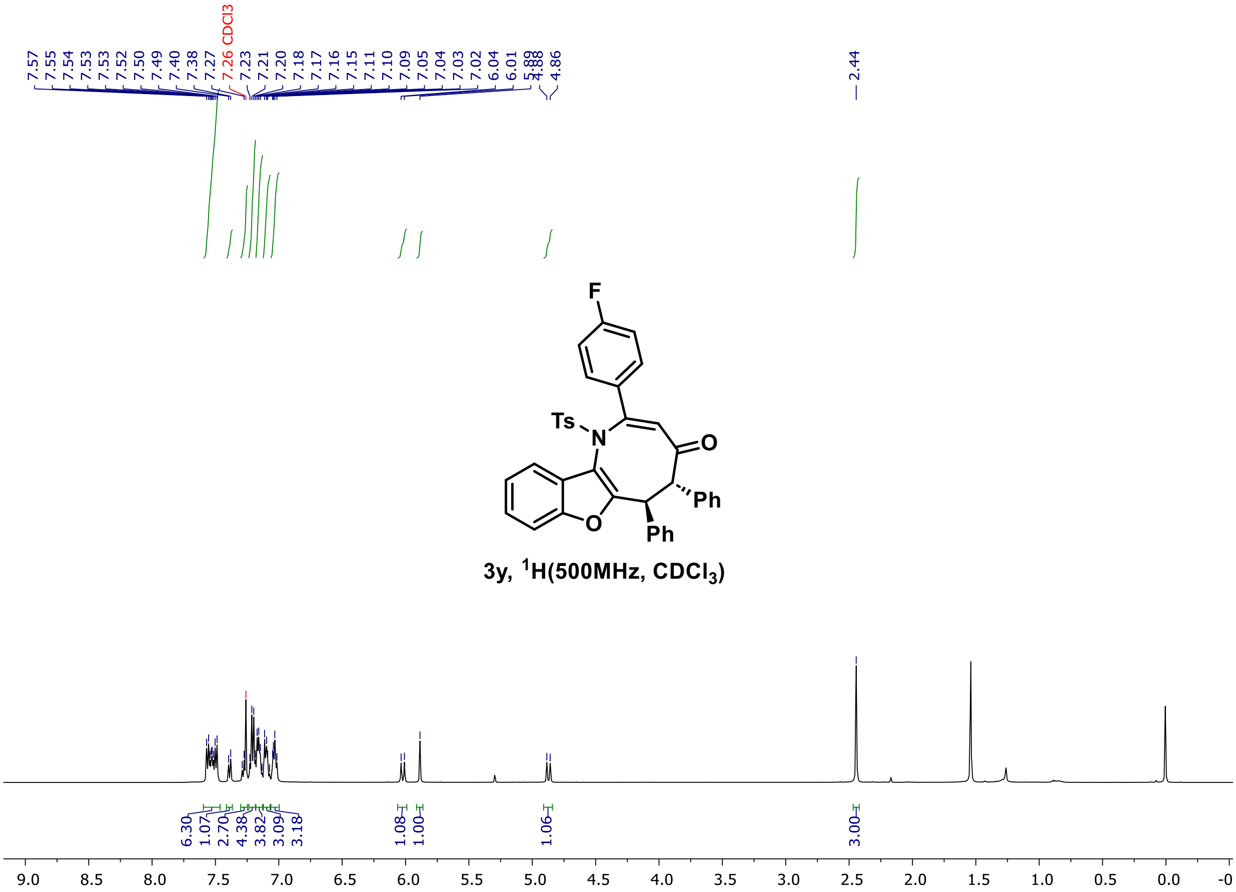

3y, ^{1}H(500MHz, $CDCl_3$)

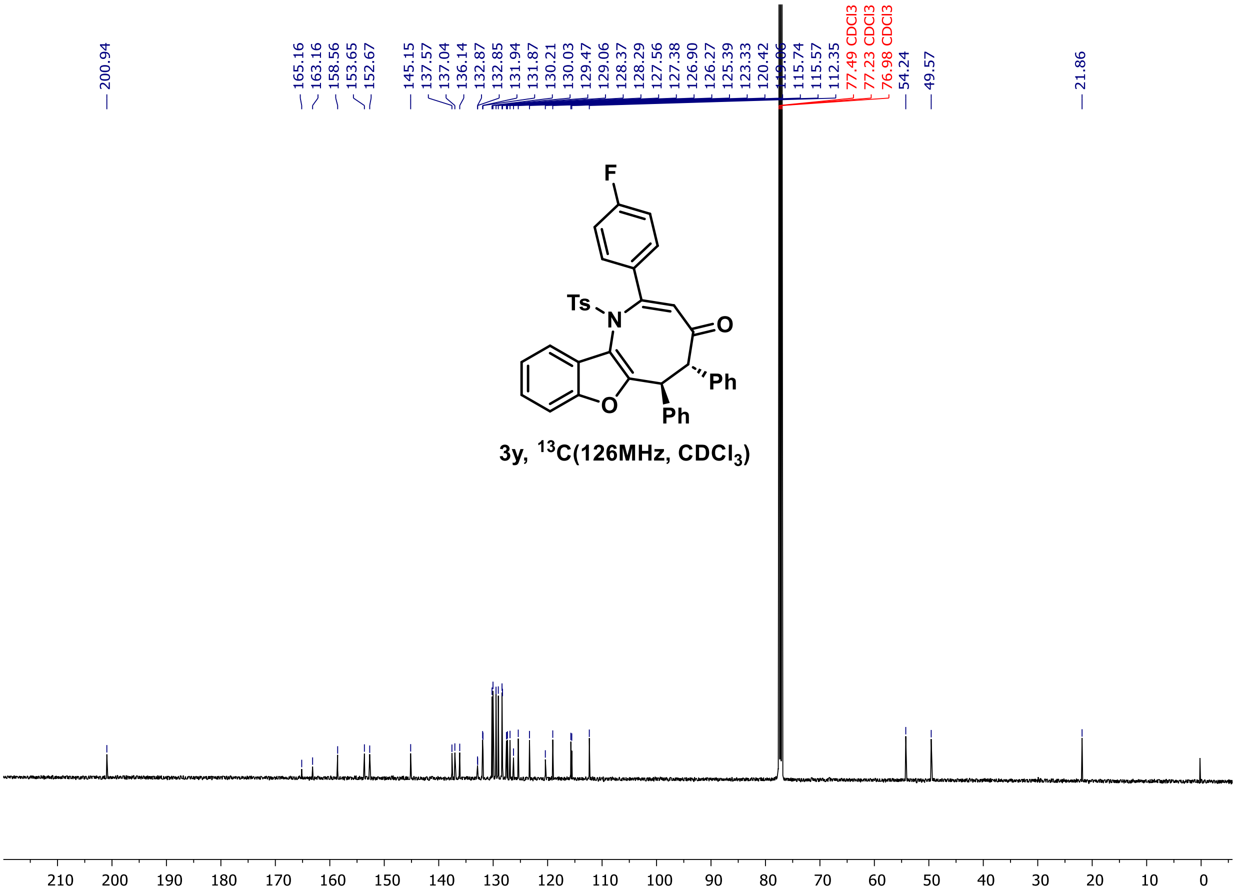

3y, ^{13}C(126MHz, $CDCl_3$)

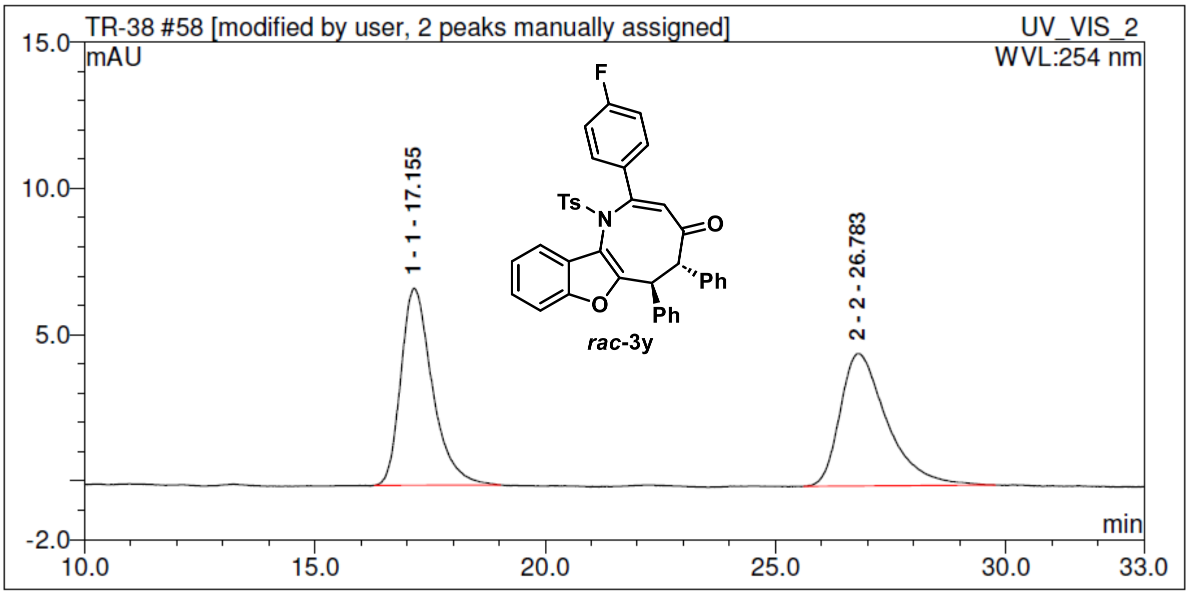

	Peak Name	Ret.Time (detected)	Area	Rel.Area(ident.)	Height	Amount
		min	mAU*min	%	mAU	
1	1	17.16	5.335576	50.0065085	6.72911	n.a.
2	2	26.78	5.334	49.9934915	4.519	n.a.

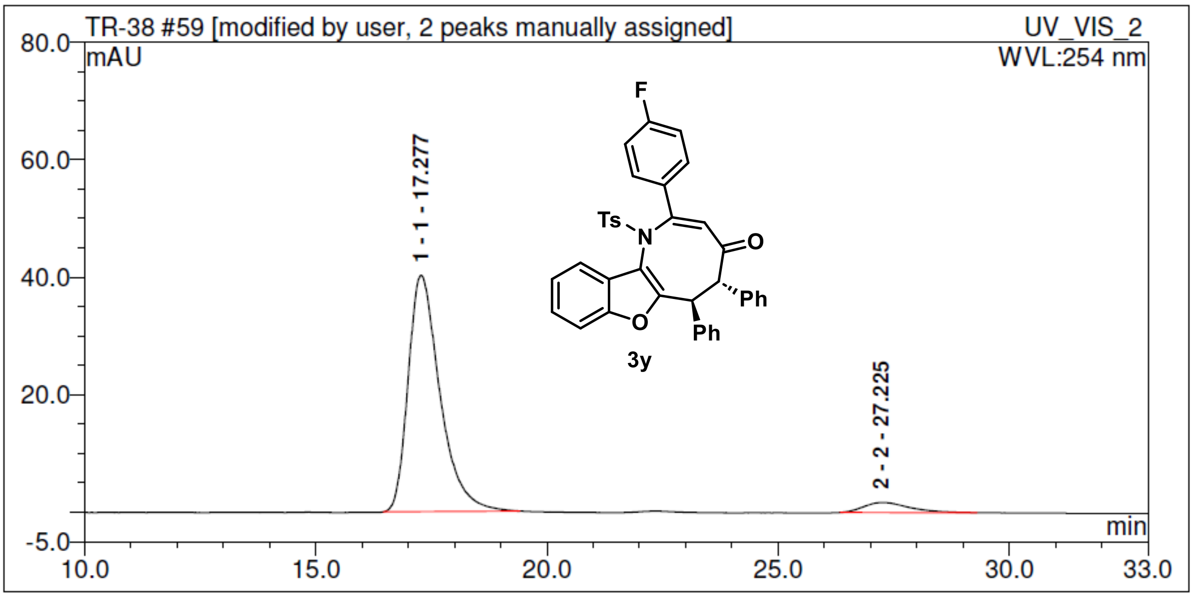

	Peak Name	Ret.Time (detected)	Area	Rel.Area(ident.)	Height	Amount
		min	mAU*min	%	mAU	
1	1	17.28	32.09874	94.26833368	40.21837	n.a.
2	2	27.23	1.952	5.731666321	1.695	n.a.

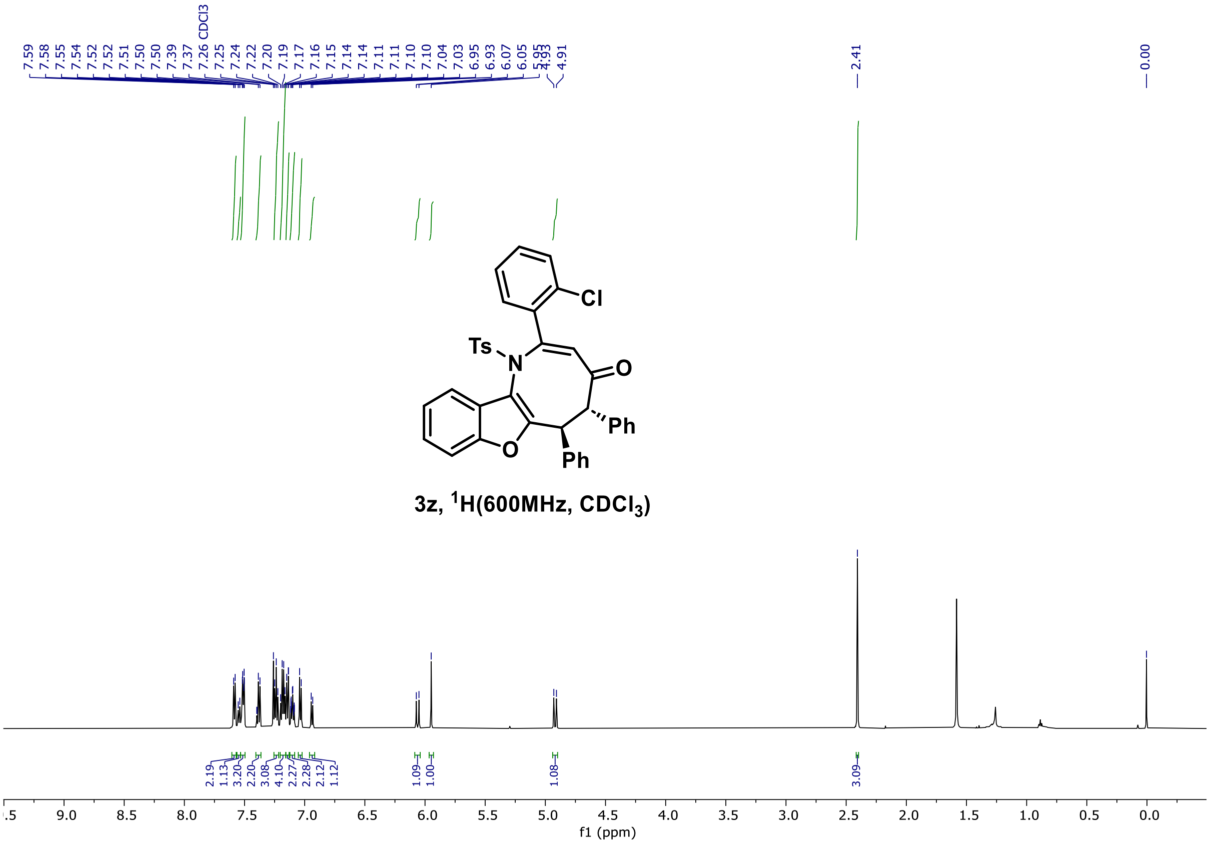

Ts
N
O
O
Cl
Ph
Ph
3z, ¹H(600MHz, CDCl₃)
f1 (ppm)

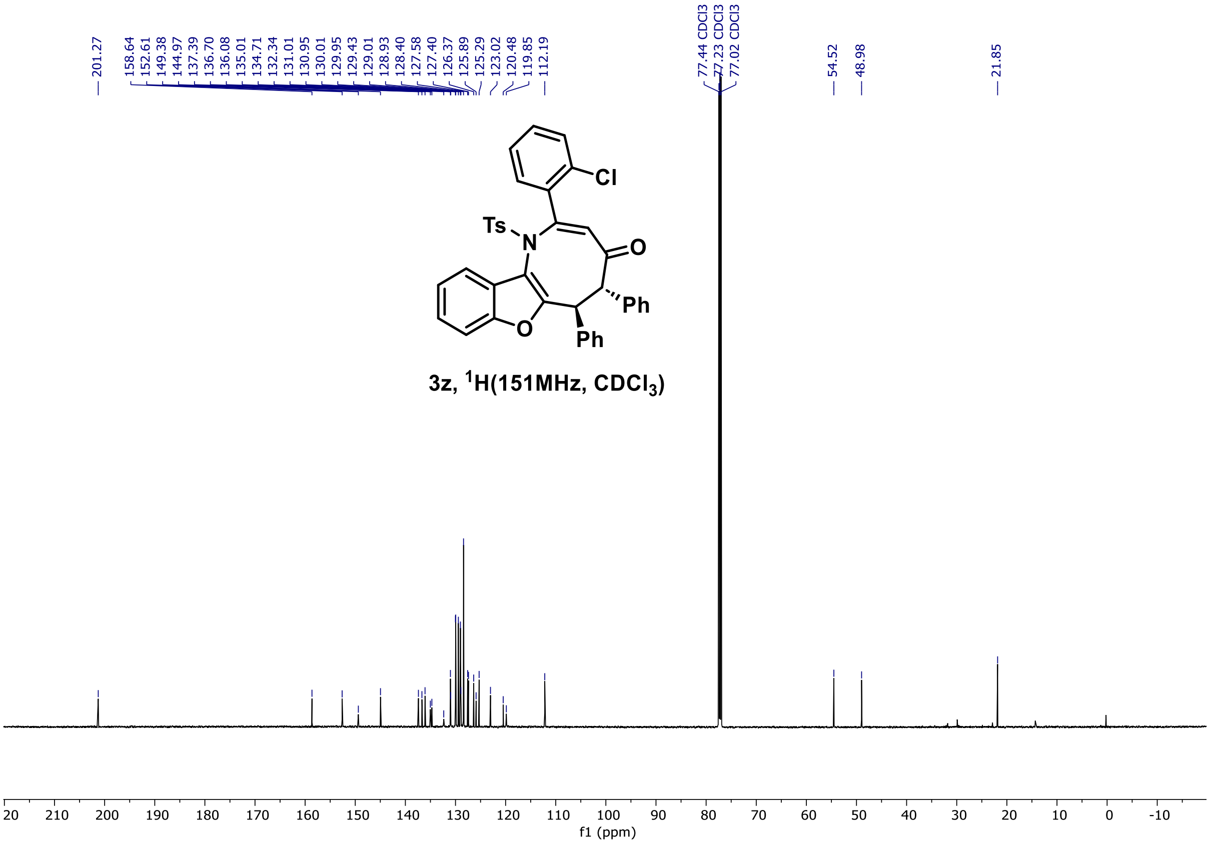

Ts
N
O
O
Cl
Ph
Ph
3z, ¹H(151MHz, CDCl₃)
f1 (ppm)

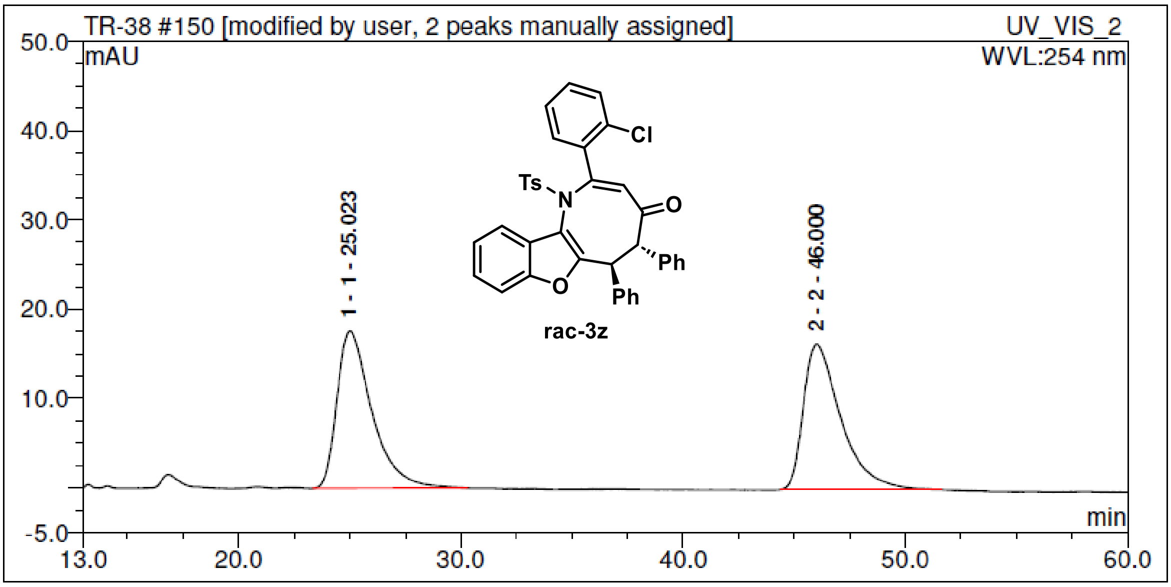

	Peak Name	Ret.Time (detected)	Area	Rel.Area(ident.)	Height	Amount
		min	mAU*min	%	mAU	
1	1	25.02	32.03932	50.29656806	17.57069	n.a.
2	2	46.00	31.661	49.70343194	16.227	n.a.

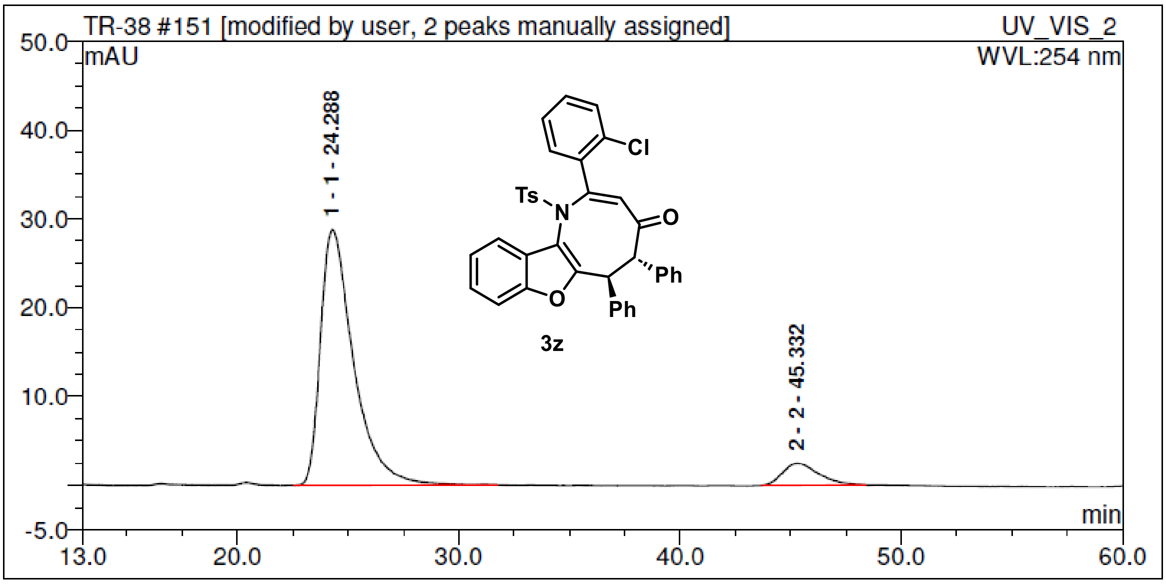

	Peak Name	Ret.Time (detected)	Area	Rel.Area(ident.)	Height	Amount
		min	mAU*min	%	mAU	
1	1	24.29	49.90013	91.58220514	28.79213	n.a.
2	2	45.33	4.587	8.417794858	2.455	n.a.

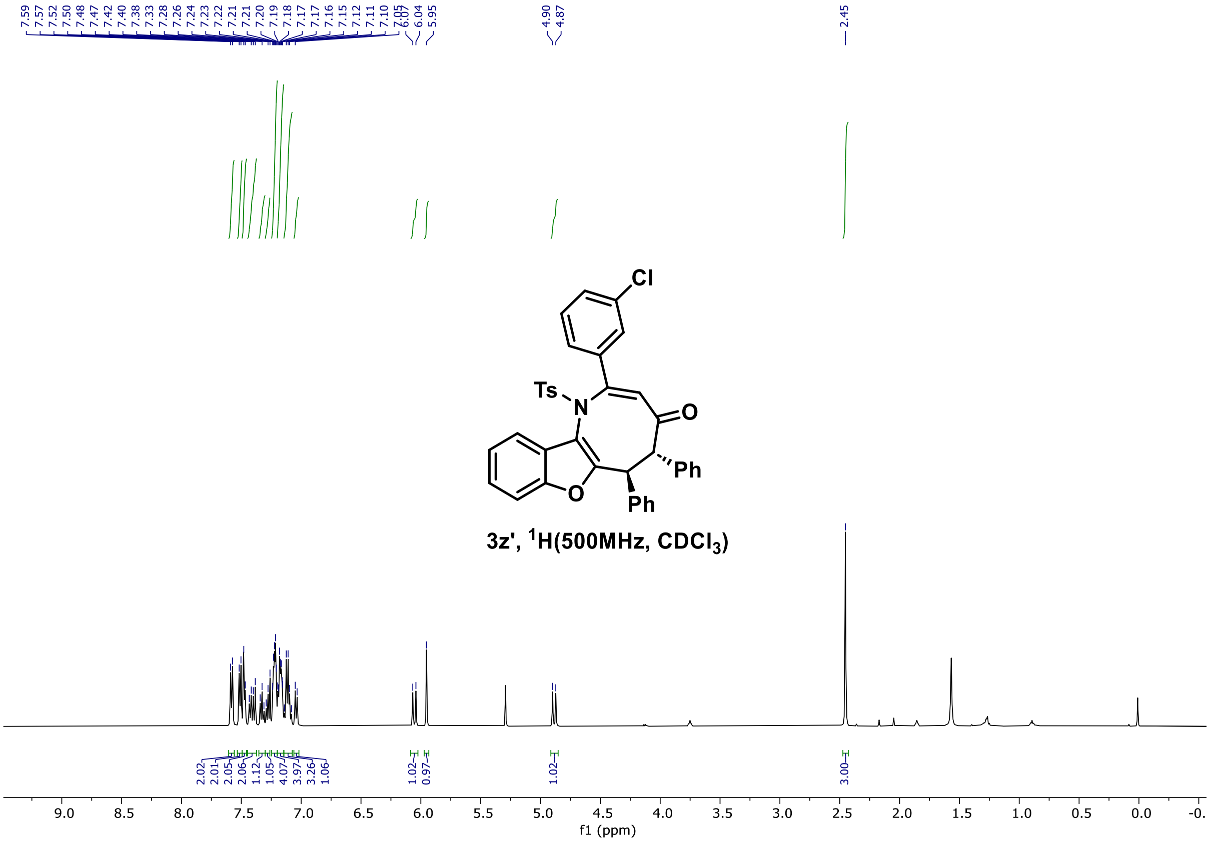
7.59 7.57 7.52 7.50 7.48 7.47 7.42 7.40 7.38 7.33 7.28 7.26 7.24 7.23 7.22 7.21 7.21 7.20 7.19 7.18 7.17 7.17 7.16 7.15 7.12 7.11 7.10 7.05 6.07 6.04 5.95
4.90 4.87
2.45
Cl
Ts
N
O
O
Ph
Ph
3z', 1H(500MHz, CDCl3)
2.02 2.01 2.05 2.06 1.12 1.05 4.07 3.97 3.26 1.06
1.02 0.97
1.02
3.00
9.0 8.5 8.0 7.5 7.0 6.5 6.0 5.5 5.0 4.5 4.0 3.5 3.0 2.5 2.0 1.5 1.0 0.5 0.0 -0.
f1 (ppm)

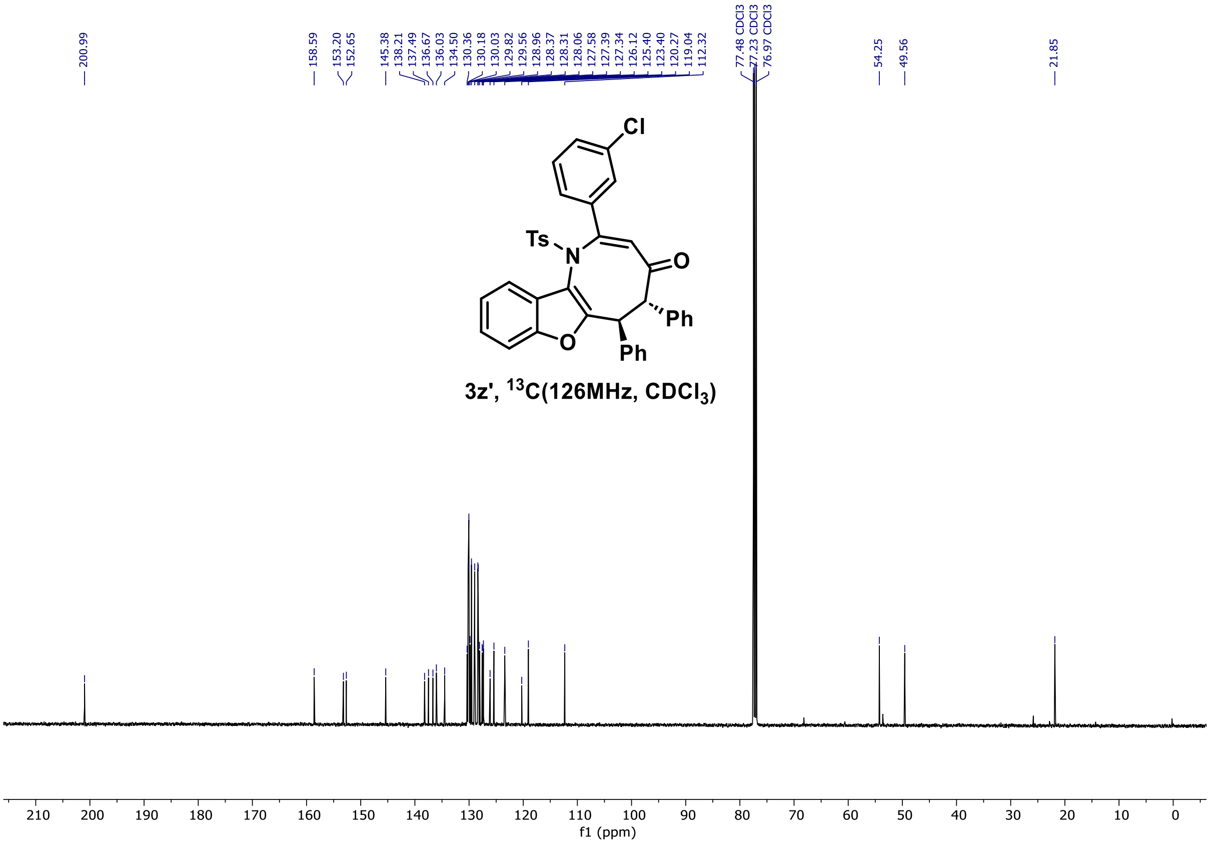
200.99
158.59 153.20 152.65
145.38 138.21 137.49 136.67 136.03 134.50 130.36 130.18 130.03 129.82 129.56 128.96 128.37 128.31 128.06 127.58 127.39 127.34 126.12 125.40 123.40 120.27 119.04 112.32
77.48 CDCl3 77.23 CDCl3 76.97 CDCl3
54.25 49.56
21.85
Cl
Ts
N
O
O
Ph
Ph
3z', 13C(126MHz, CDCl3)
210 200 190 180 170 160 150 140 130 120 110 100 90 80 70 60 50 40 30 20 10 0
f1 (ppm)

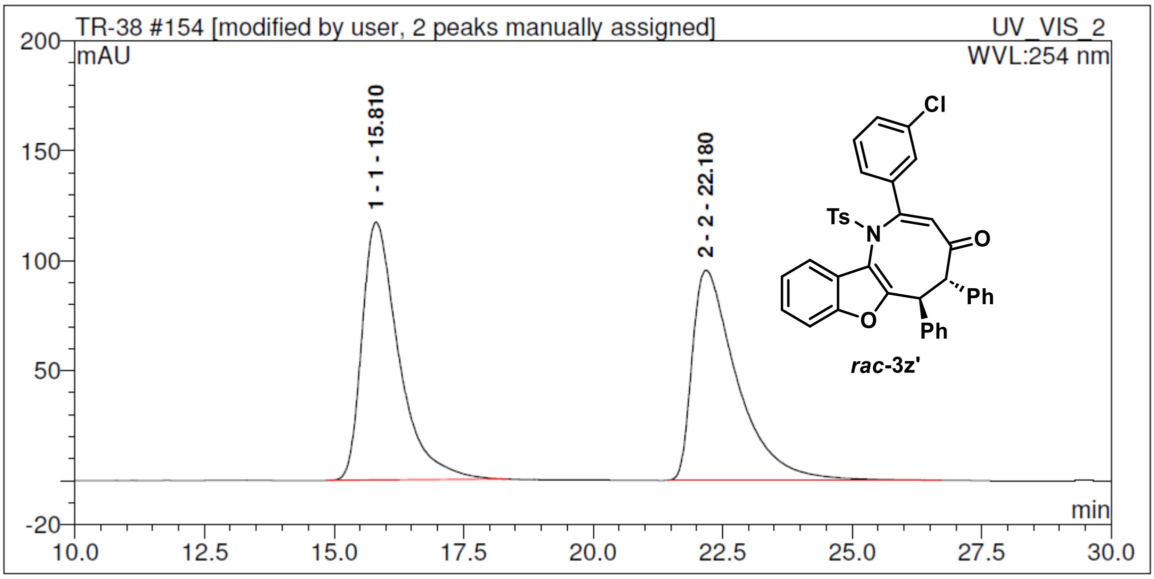

Peak Name		Ret.Time (detected) min	Area mAU*min	Rel.Area(ident.) %	Height mAU	Amount
1	1	15.81	96.98689	50.17819154	117.309	n.a.
2	2	22.18	96.298	49.82180846	95.546	n.a.

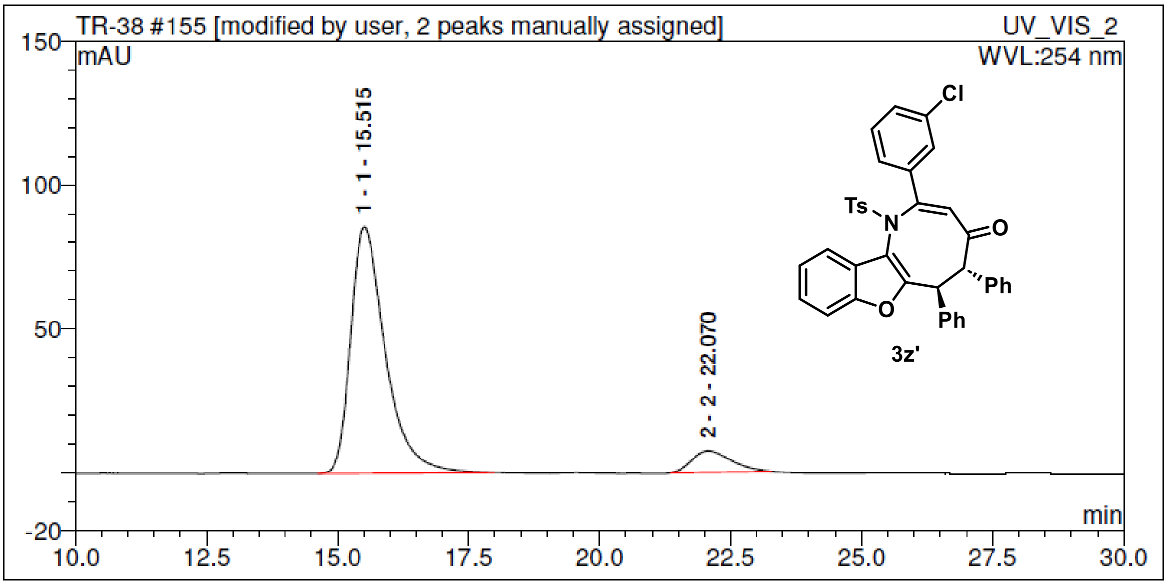

Peak Name		Ret.Time (detected) min	Area mAU*min	Rel.Area(ident.) %	Height mAU	Amount
1	1	15.52	65.45626	91.16591043	85.4996	n.a.
2	2	22.07	6.343	8.834089575	7.296	n.a.

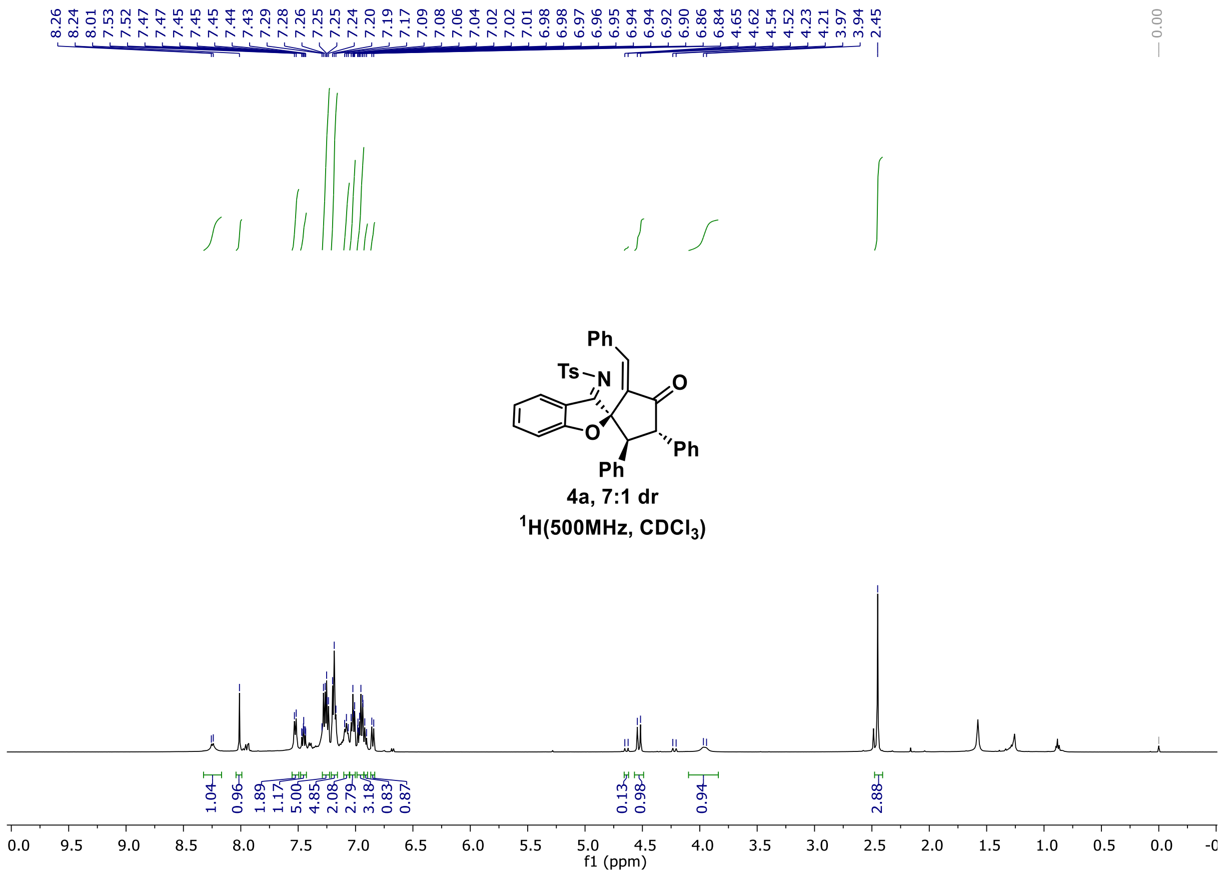
4a, 7:1 dr
^{1}H(500MHz, CDCl$_3$)
f1 (ppm)

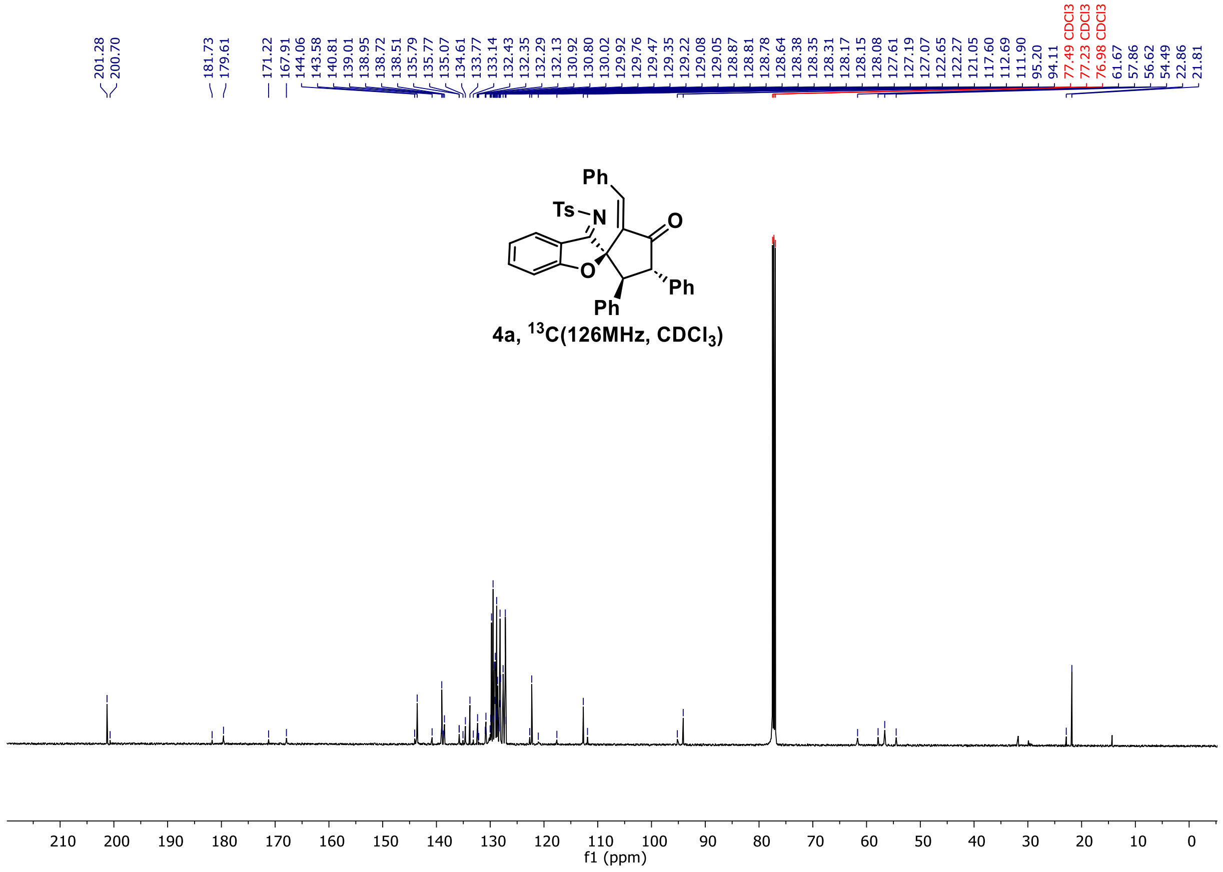
4a, ^{13}C(126MHz, CDCl$_3$)
f1 (ppm)

The major diastereomer is confirmed from the crystal structure of **4k**. The enantiomeric ratio of the crystal is found to be 98%ee and defines the major isomer as (2*R*,4'*S*,5'*S*,2'*E*). The 3*J* Ph-C5'H-C4'H-Ph couplings of major and minor diastereoisomers are same in 1H-NMR. So, the relative stereochemistry on these positions is identical in both of the diastereomers.

Possible isomers:

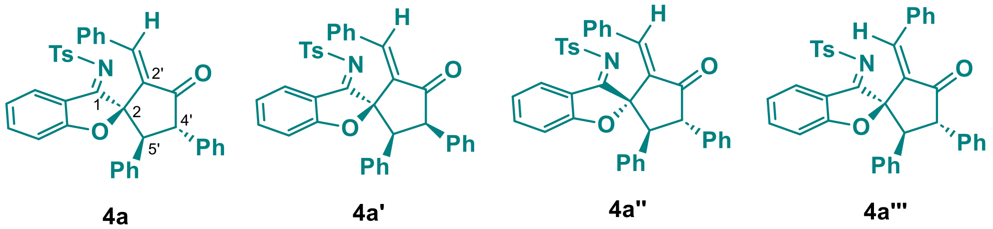

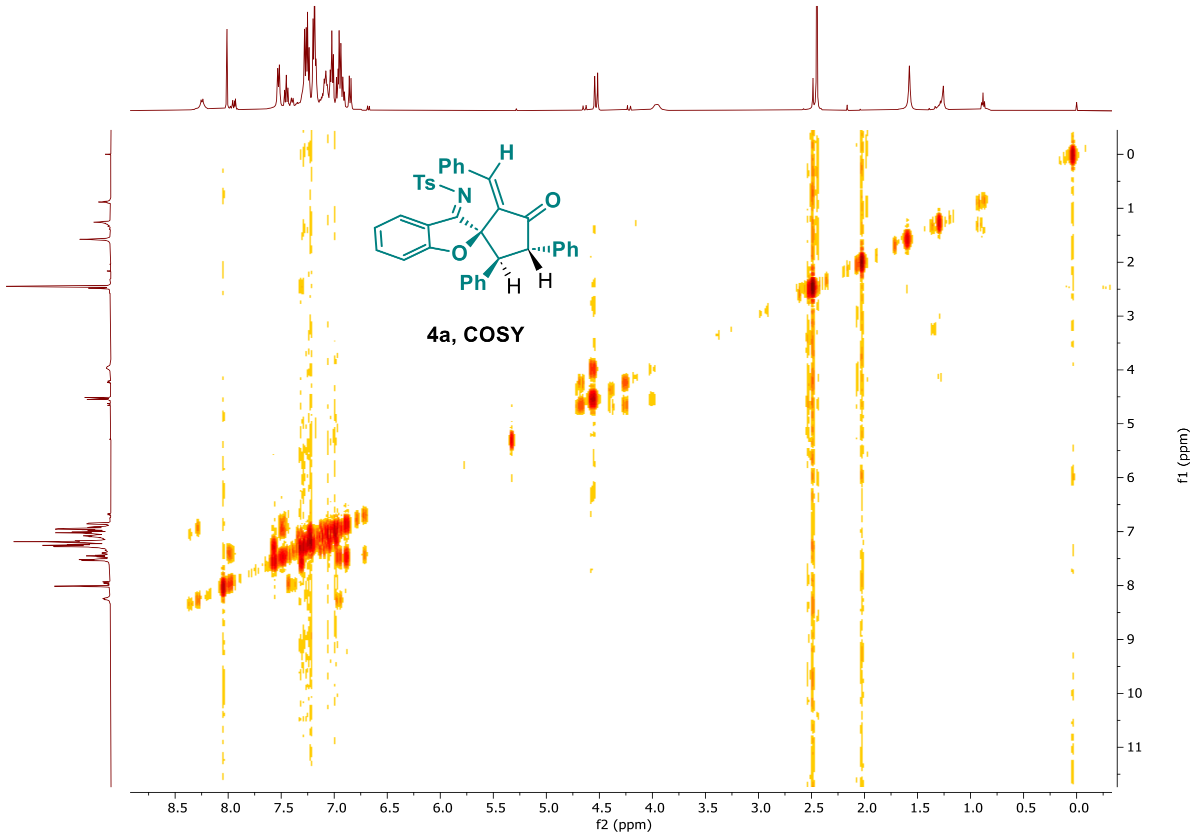

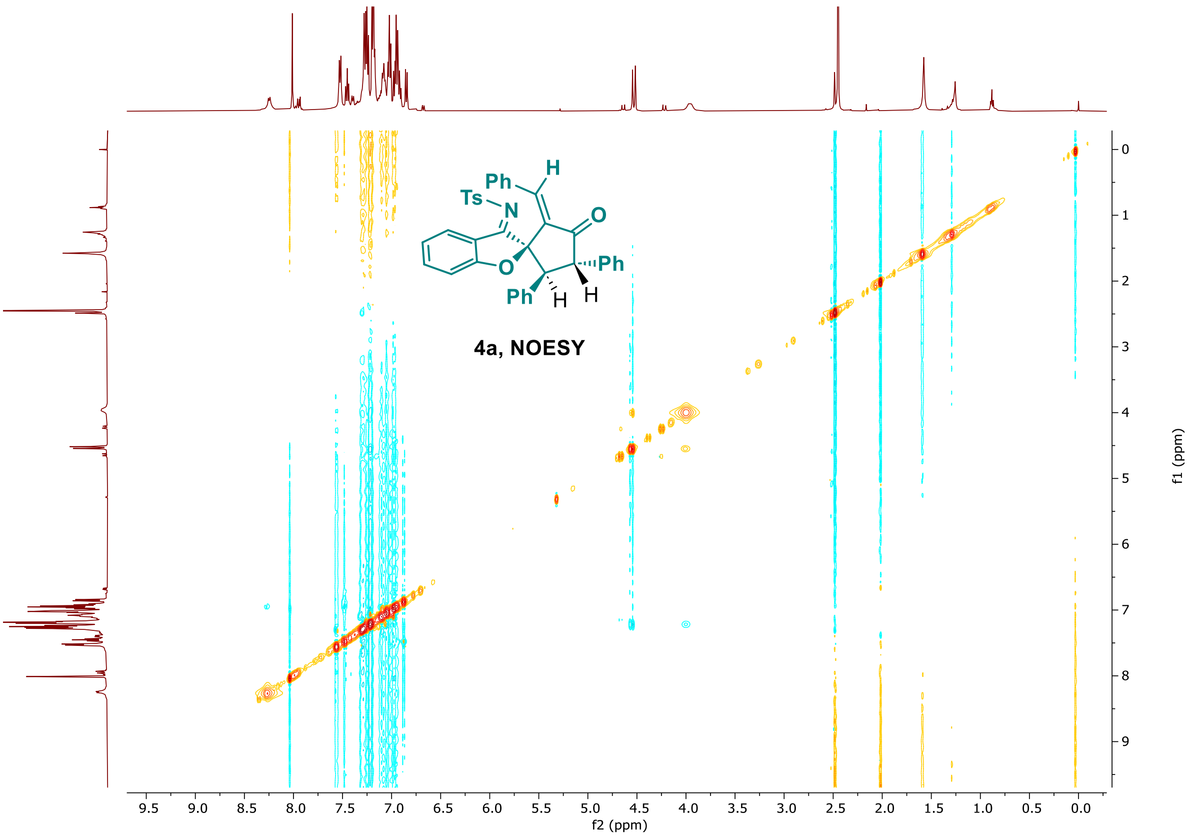
Ts
N
Ph
H
O
O
Ph
Ph
H
H
4a, NOESY
f1 (ppm)
f2 (ppm)

In HMBC, the interaction between C1 and C5'-H (3 bond interaction), is absent in major isomer, whereas, it is present in minor isomer. Hence, the diastereoselectivity correspond to the spiro center(

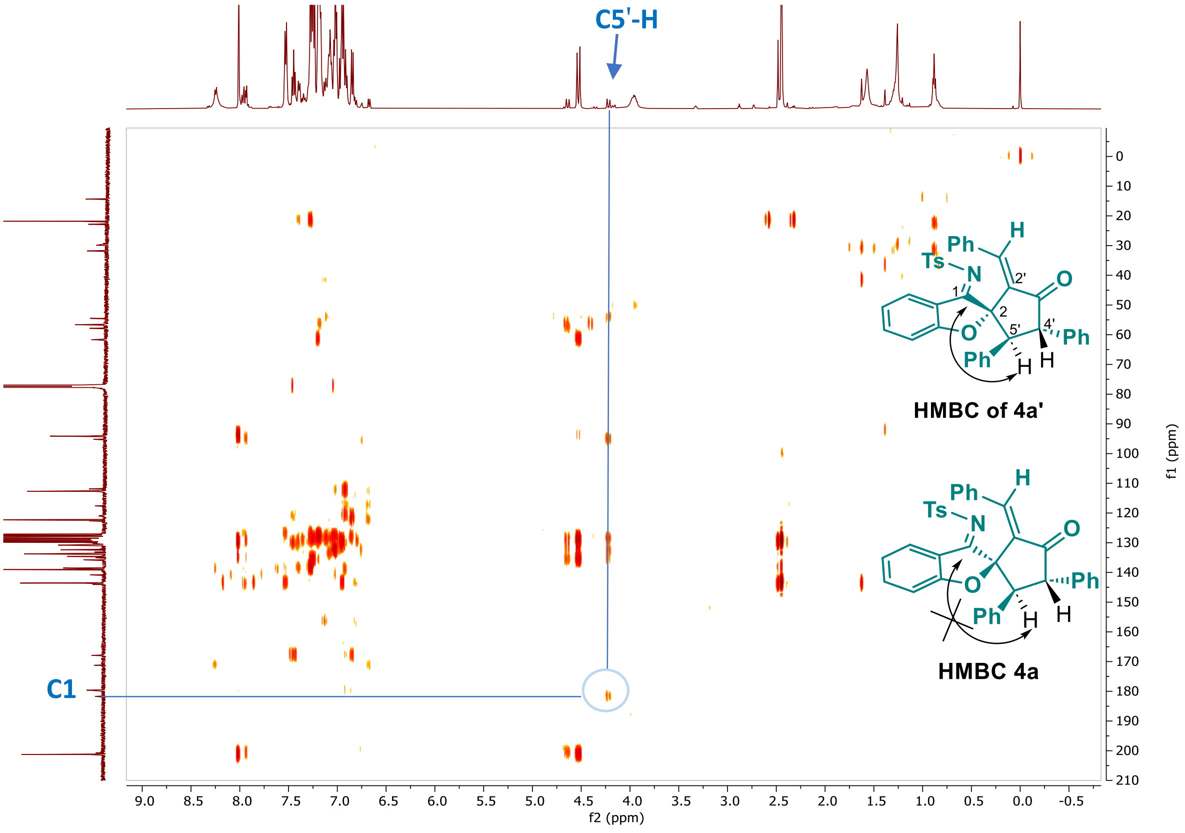

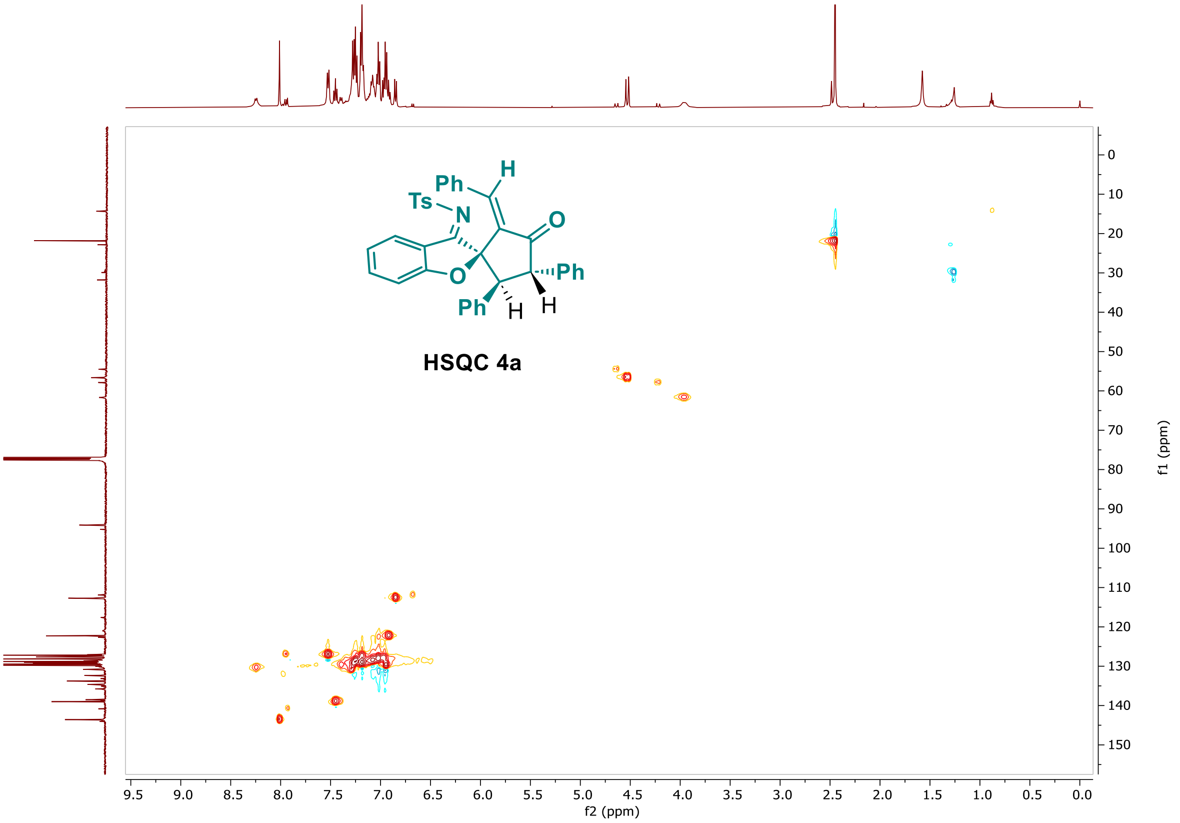

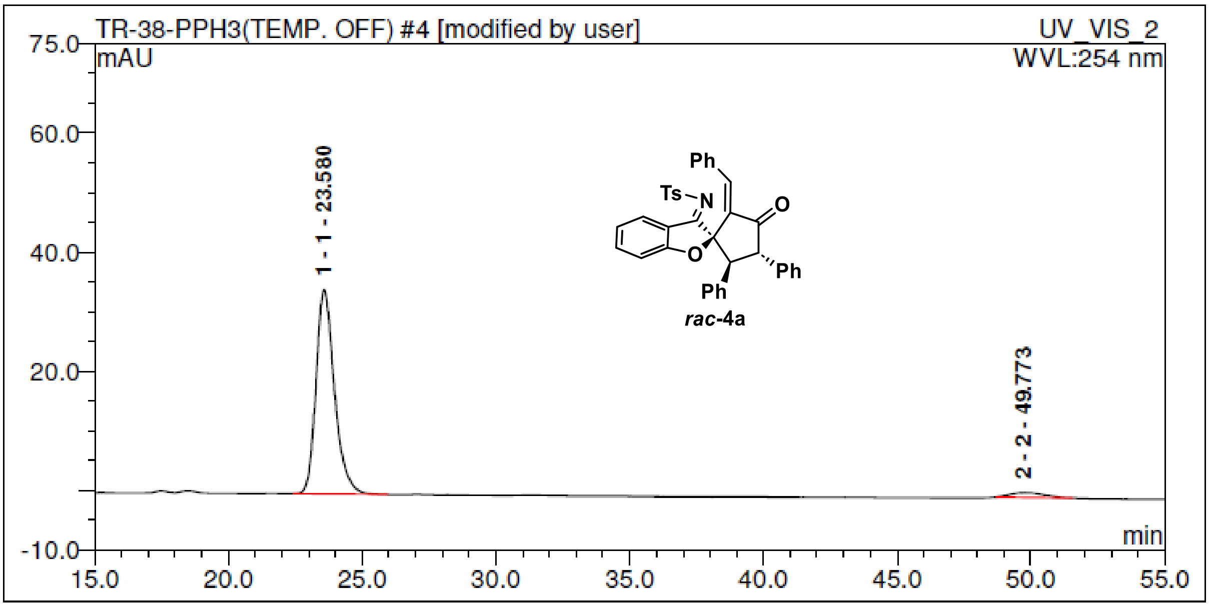

	Peak Name	Ret.Time (detected) min	Area mAU*min	Rel.Area(ident.) %	Height mAU	Amount
1	1	23.58	27.43192	95.86291217	34.29574	n.a.
2	2	49.77	1.184	4.137087826	0.830	n.a.

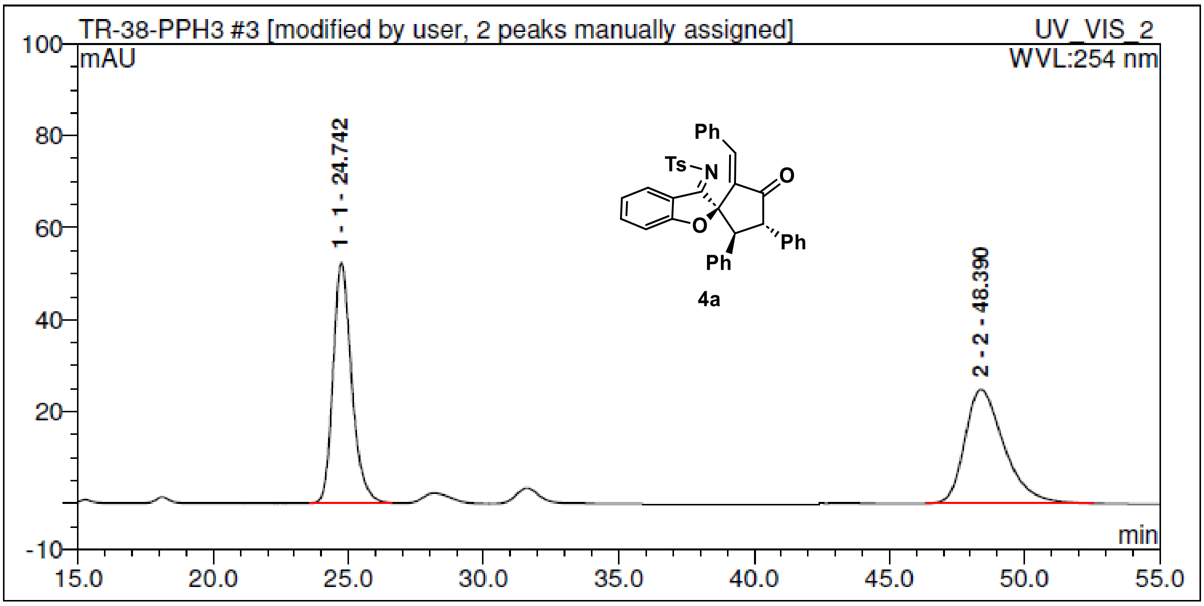

	Peak Name	Ret.Time (detected) min	Area mAU*min	Rel.Area(ident.) %	Height mAU	Amount
1	1	24.74	41.55797	49.91453761	52.22303	n.a.
2	2	48.39	41.700	50.08546239	24.754	n.a.

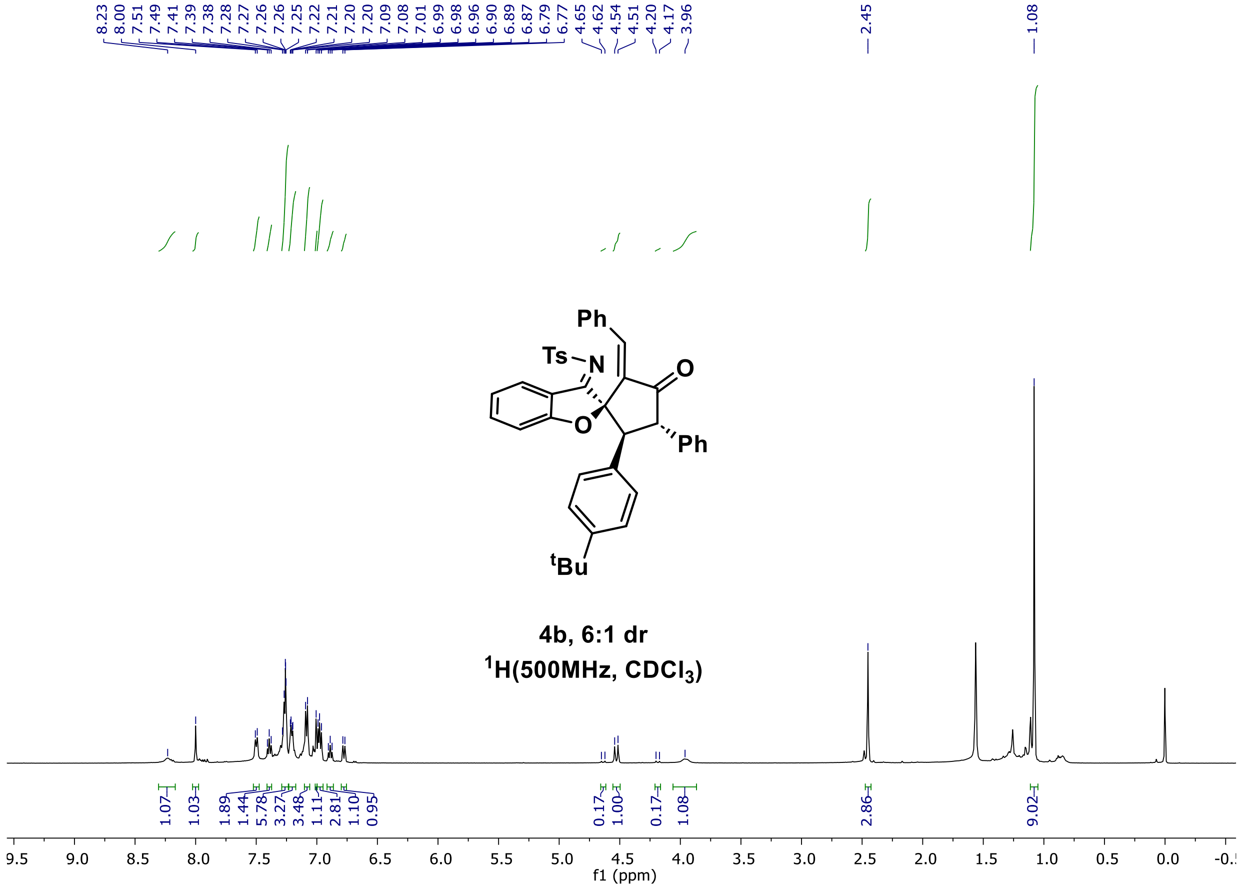
8.23
8.00
7.51
7.49
7.41
7.39
7.38
7.28
7.27
7.26
7.25
7.22
7.21
7.20
7.20
7.09
7.08
7.01
6.99
6.98
6.96
6.90
6.89
6.87
6.79
6.77
4.65
4.62
4.54
4.51
4.20
4.17
3.96
2.45
1.08
Ph
Ts
N
O
O
Ph
tBu
4b, 6:1 dr
1H(500MHz, CDCl3)
1.07
1.03
1.89
1.44
5.78
3.27
3.48
1.11
2.81
1.10
0.95
0.17
1.00
0.17
1.08
2.86
9.02
9.5
9.0
8.5
8.0
7.5
7.0
6.5
6.0
5.5
5.0
4.5
4.0
3.5
3.0
2.5
2.0
1.5
1.0
0.5
0.0
f1 (ppm)

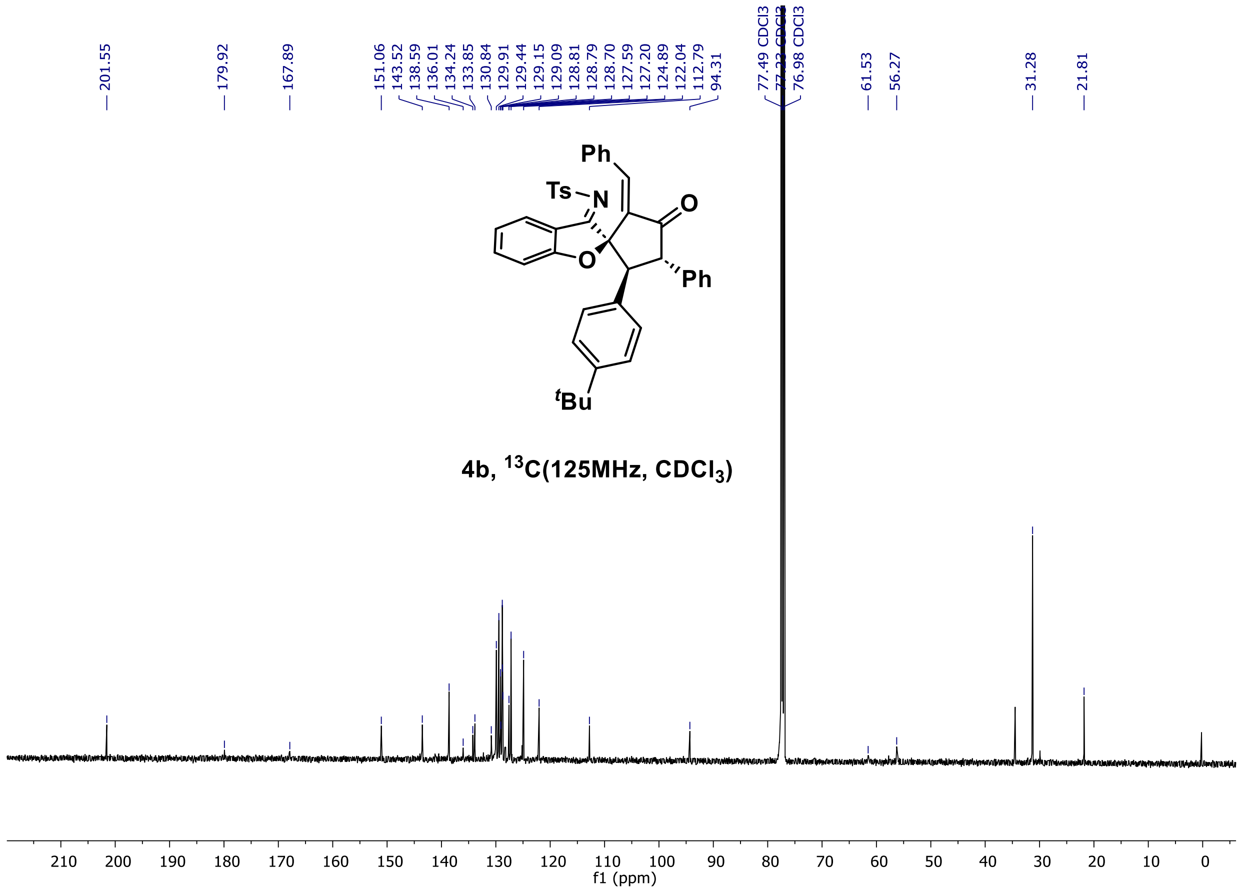
201.55
179.92
167.89
151.06
143.52
138.59
136.01
134.24
133.85
130.84
129.91
129.44
129.15
129.09
128.81
128.79
128.70
127.59
127.20
124.89
122.04
112.79
94.31
77.49 CDCl3
76.98 CDCl3
61.53
56.27
31.28
21.81
Ph
Ts
N
O
O
Ph
tBu
4b, 13C(125MHz, CDCl3)
210
200
190
180
170
160
150
140
130
120
110
100
90
80
70
60
50
40
30
20
10
0
f1 (ppm)

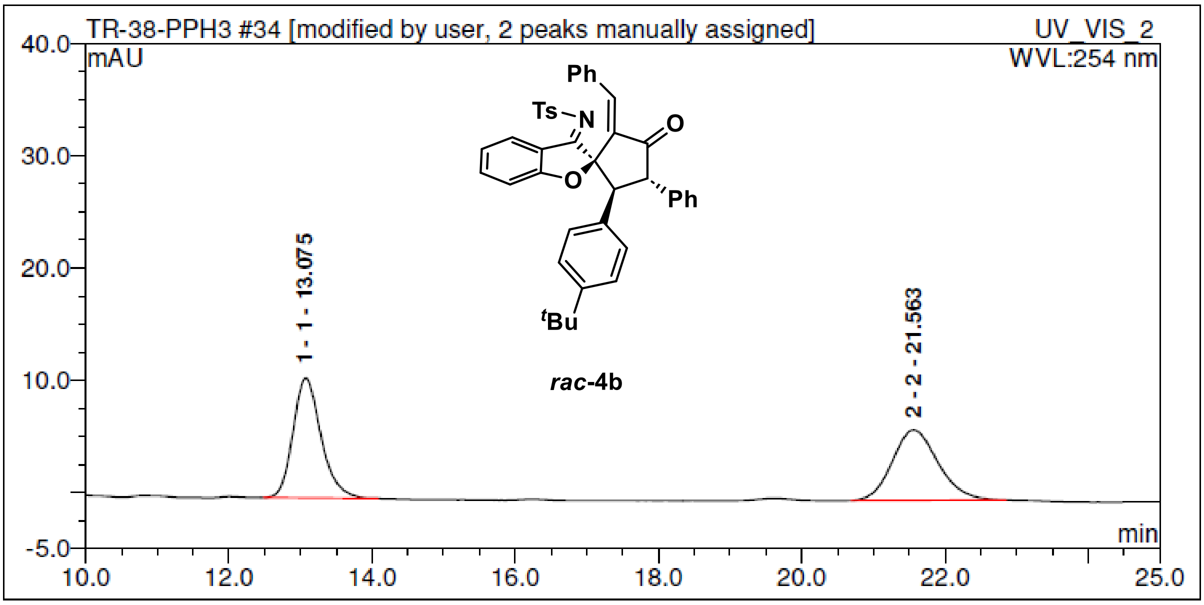

Peak	Name	Ret.Time (detected) min	Area mAU*min	Rel.Area(ident.) %	Height mAU	Amount
1	1	13.08	4.855707	51.00516658	10.67309	n.a.
2	2	21.56	4.664	48.99483342	6.271	n.a.

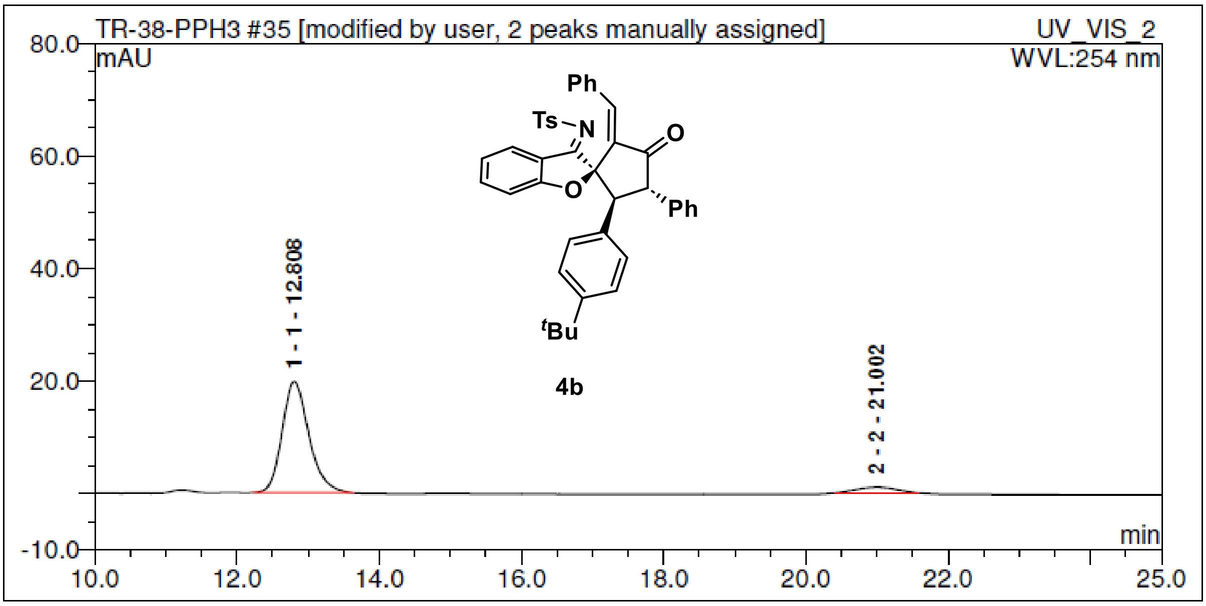

Peak	Name	Ret.Time (detected) min	Area mAU*min	Rel.Area(ident.) %	Height mAU	Amount
1	1	12.81	8.653137	92.97864357	19.76039	n.a.
2	2	21.00	0.653	7.021356427	1.026	n.a.

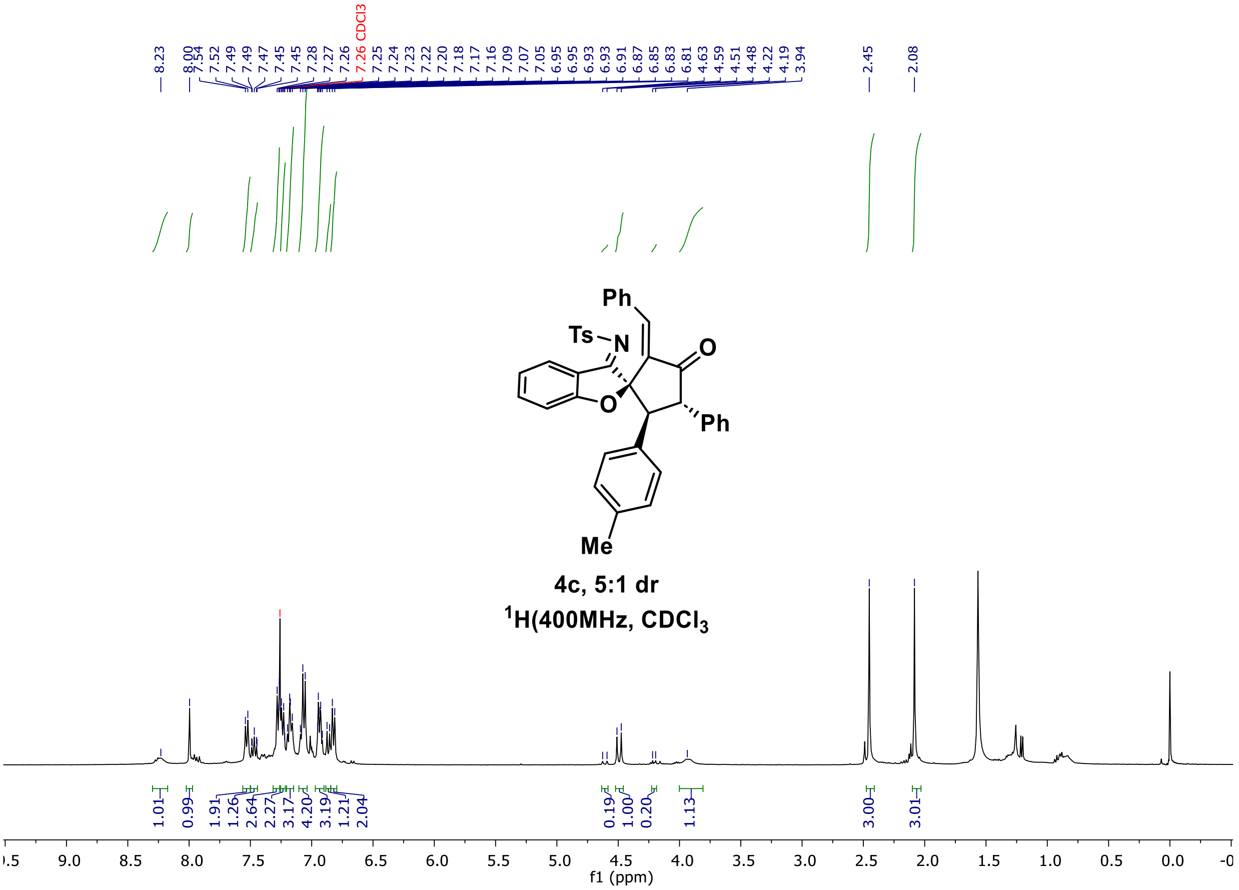

4c, 5:1 dr

^{1}H(400MHz, $CDCl_3$)

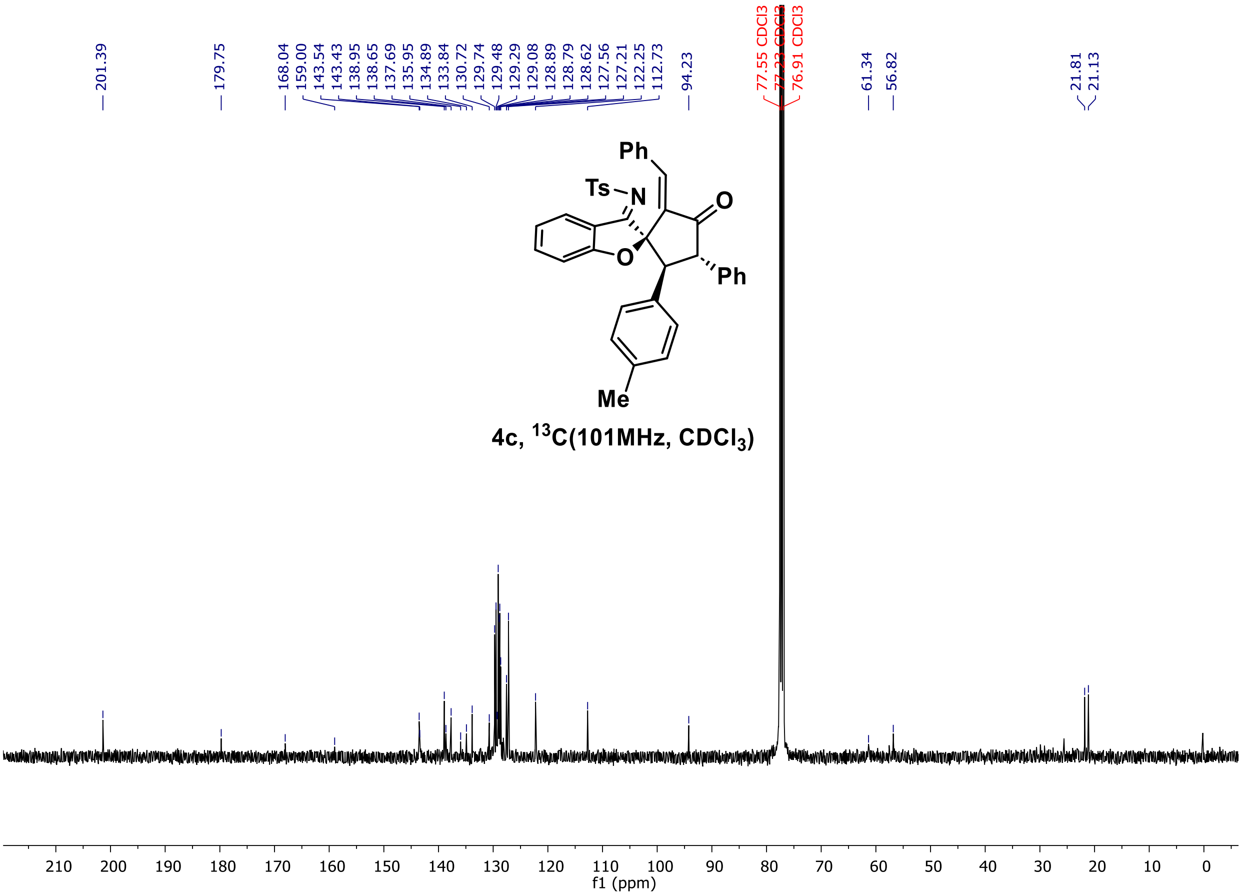

4c, ^{13}C(101MHz, $CDCl_3$)

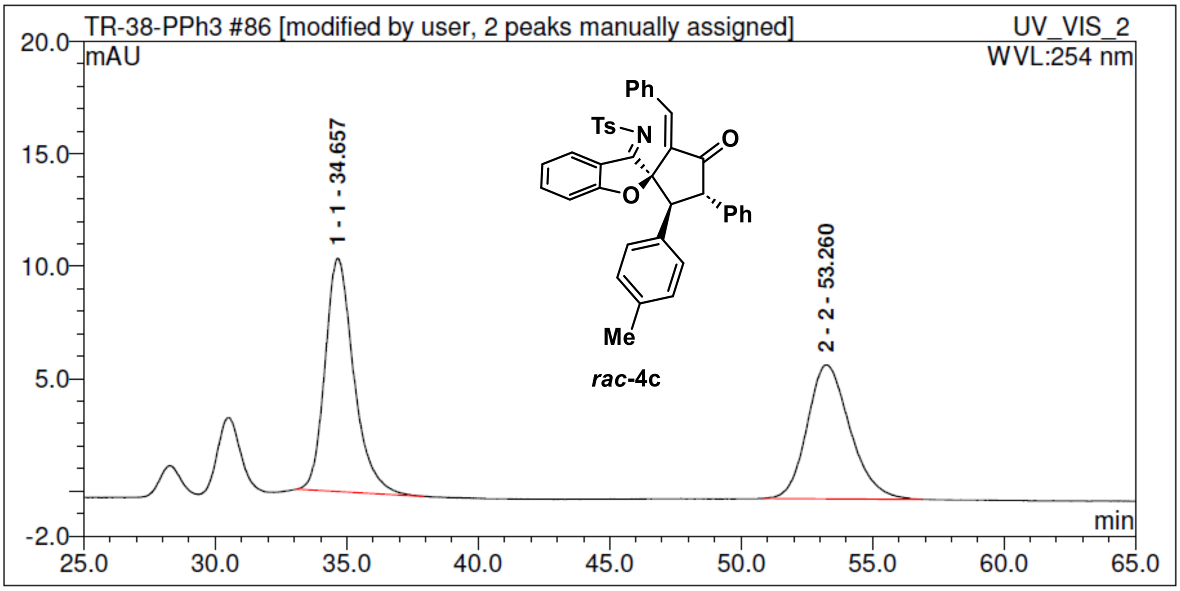

	Peak Name	Ret.Time (detected)	Area	Rel.Area(ident.)	Height	Amount
		min	mAU*min	%	mAU	
1	1	34.66	13.20142	54.04118852	10.3779	n.a.
2	2	53.26	11.227	45.95881148	5.953	n.a.

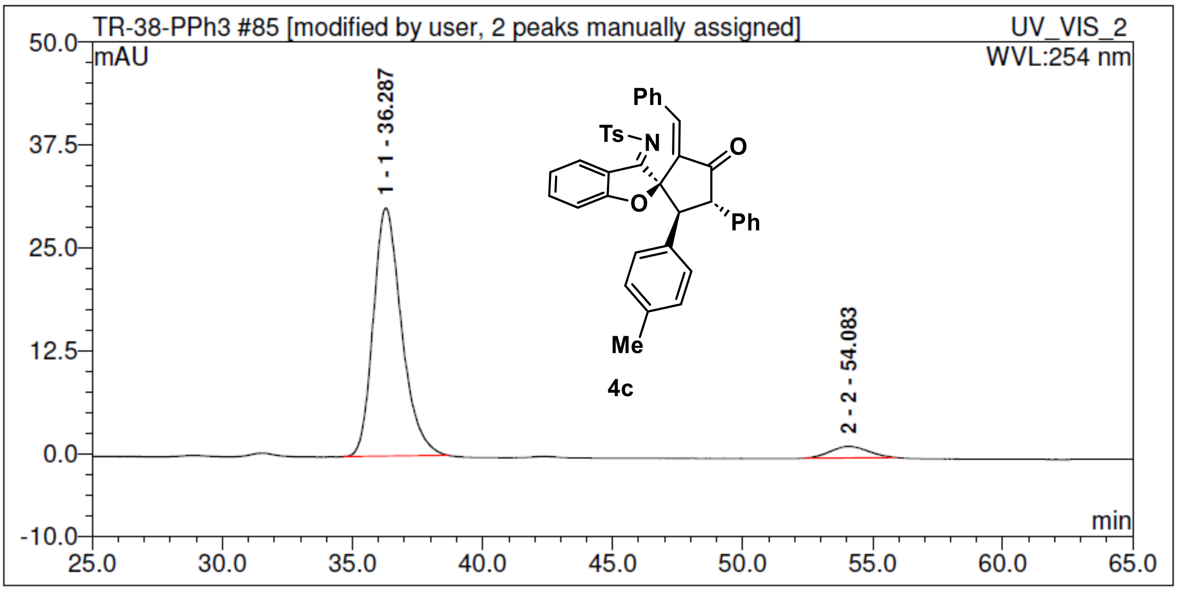

	Peak Name	Ret.Time (detected)	Area	Rel.Area(ident.)	Height	Amount
		min	mAU*min	%	mAU	
1	1	36.29	38.09243	94.20908568	30.12368	n.a.
2	2	54.08	2.341	5.790914316	1.386	n.a.

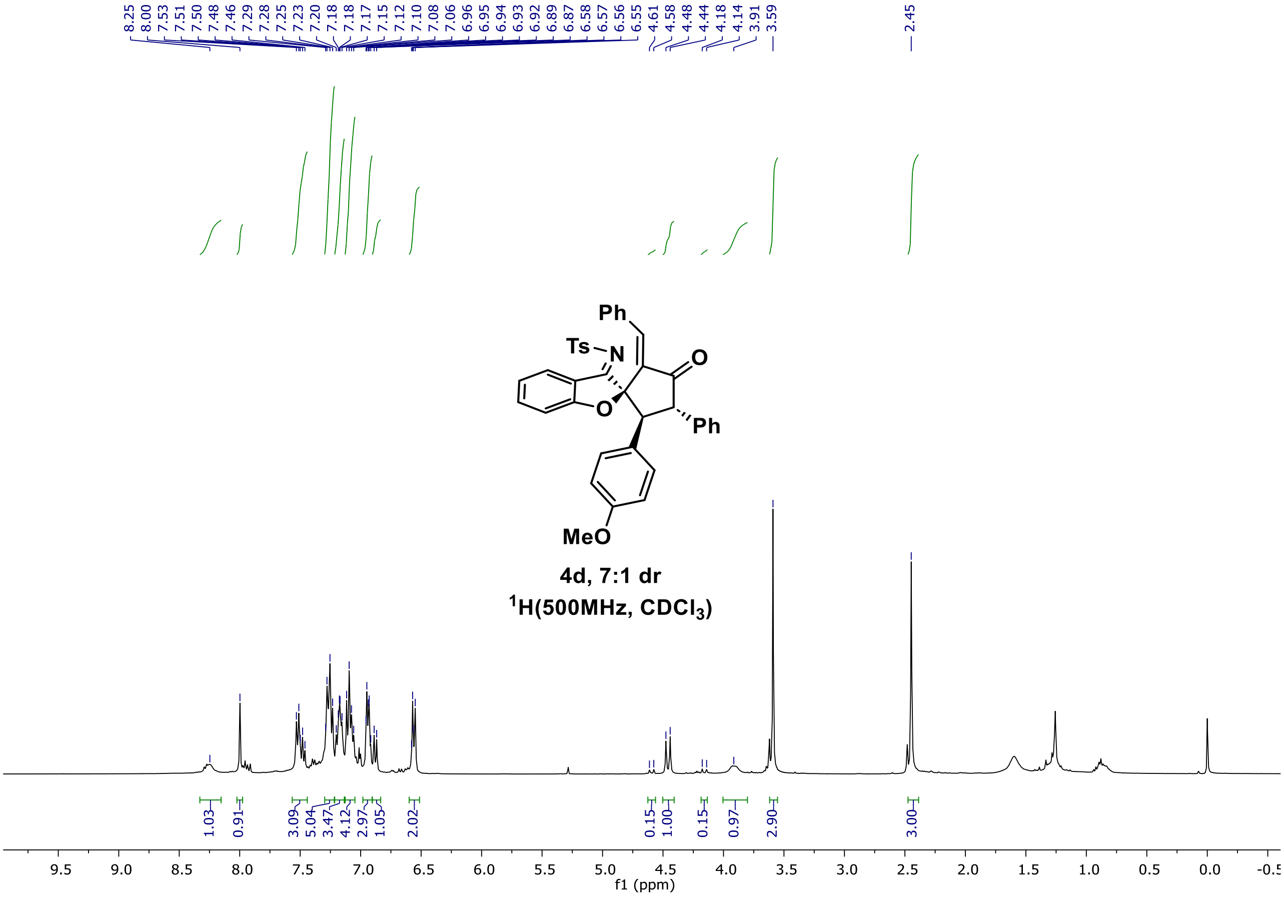
8.25 8.00 7.53 7.51 7.50 7.48 7.46 7.29 7.28 7.25 7.23 7.20 7.18 7.18 7.17 7.15 7.12 7.10 7.08 7.06 6.96 6.95 6.94 6.93 6.92 6.89 6.87 6.58 6.57 6.56 6.55 4.61 4.58 4.48 4.44 4.18 4.14 3.91 3.59
2.45
Ph
Ts
N
O
O
Ph
MeO
4d, 7:1 dr
1H(500MHz, CDCl3)
1.03 0.91 3.09 5.04 3.47 4.12 2.97 1.05 2.02
0.15 1.00 0.15 0.97 2.90
3.00
f1 (ppm)

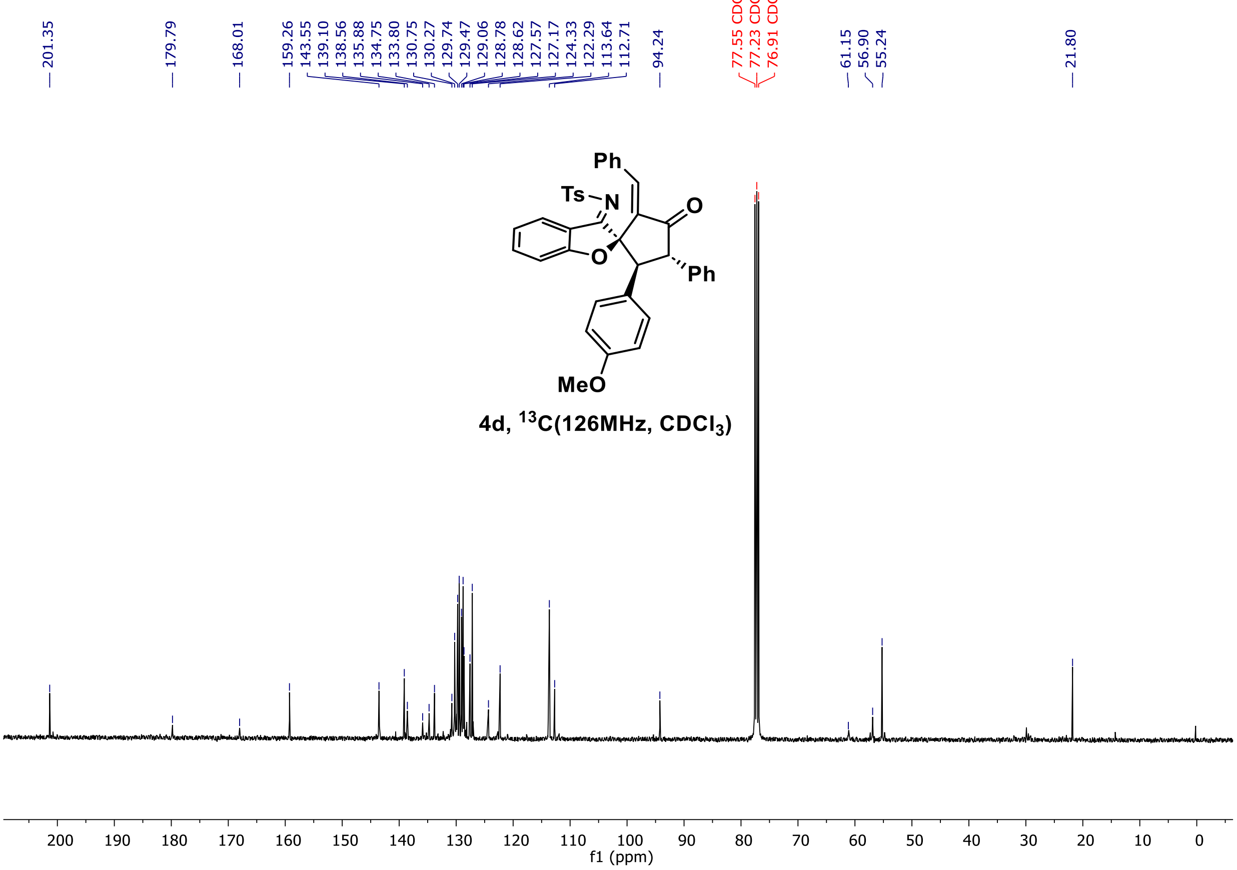
201.35
179.79
168.01
159.26 143.55 139.10 138.56 135.88 134.75 133.80 130.75 130.27 129.74 129.47 129.06 128.78 128.62 127.57 127.17 124.33 122.29 113.64 112.71
94.24
77.55 CDCl3
77.23 CDCl3
76.91 CDCl3
61.15 56.90 55.24
21.80
Ph
Ts
N
O
O
Ph
MeO
4d, 13C(126MHz, CDCl3)
f1 (ppm)

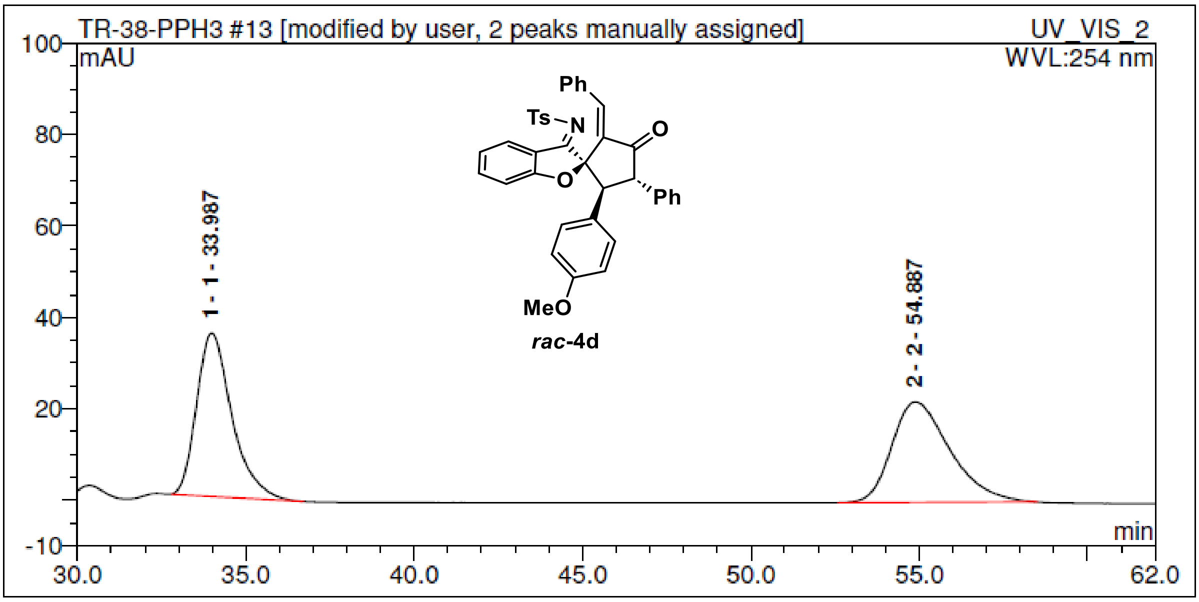

Peak	Name	Ret.Time (detected) min	Area mAU*min	Rel.Area(ident.) %	Height mAU	Amount
1	1	33.99	43.4933	49.72191614	35.73856	n.a.
2	2	54.89	43.980	50.27808386	21.959	n.a.

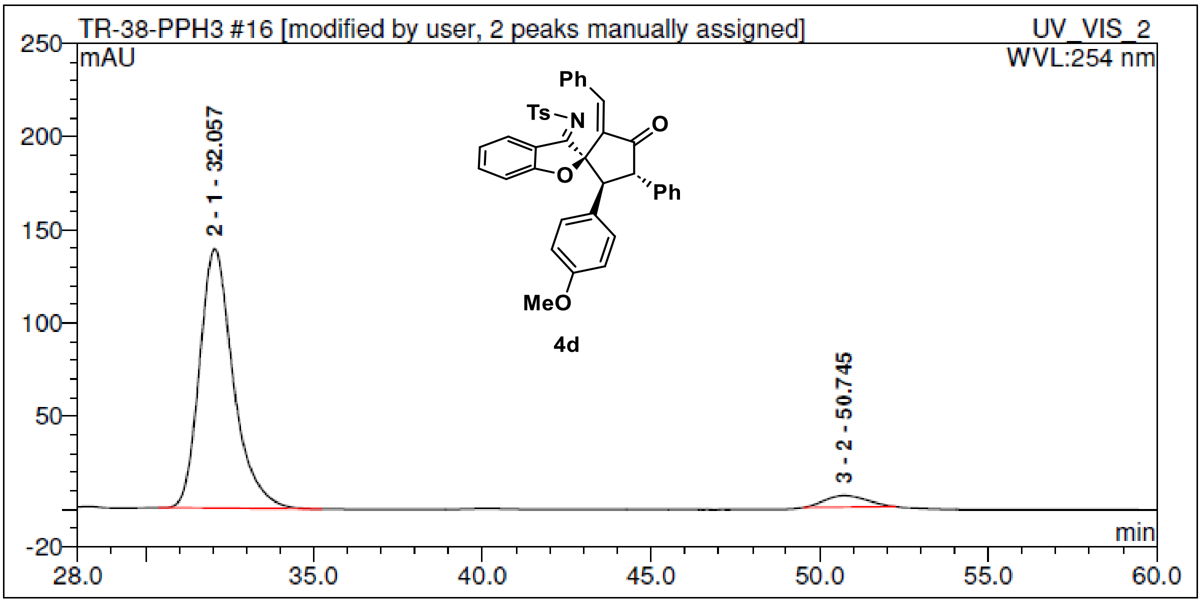

Peak	Name	Ret.Time (detected) min	Area mAU*min	Rel.Area(ident.) %	Height mAU	Amount
2	1	32.06	161.7017	94.73468066	138.9955	n.a.
3	2	50.75	8.987	5.265319343	6.241	n.a.

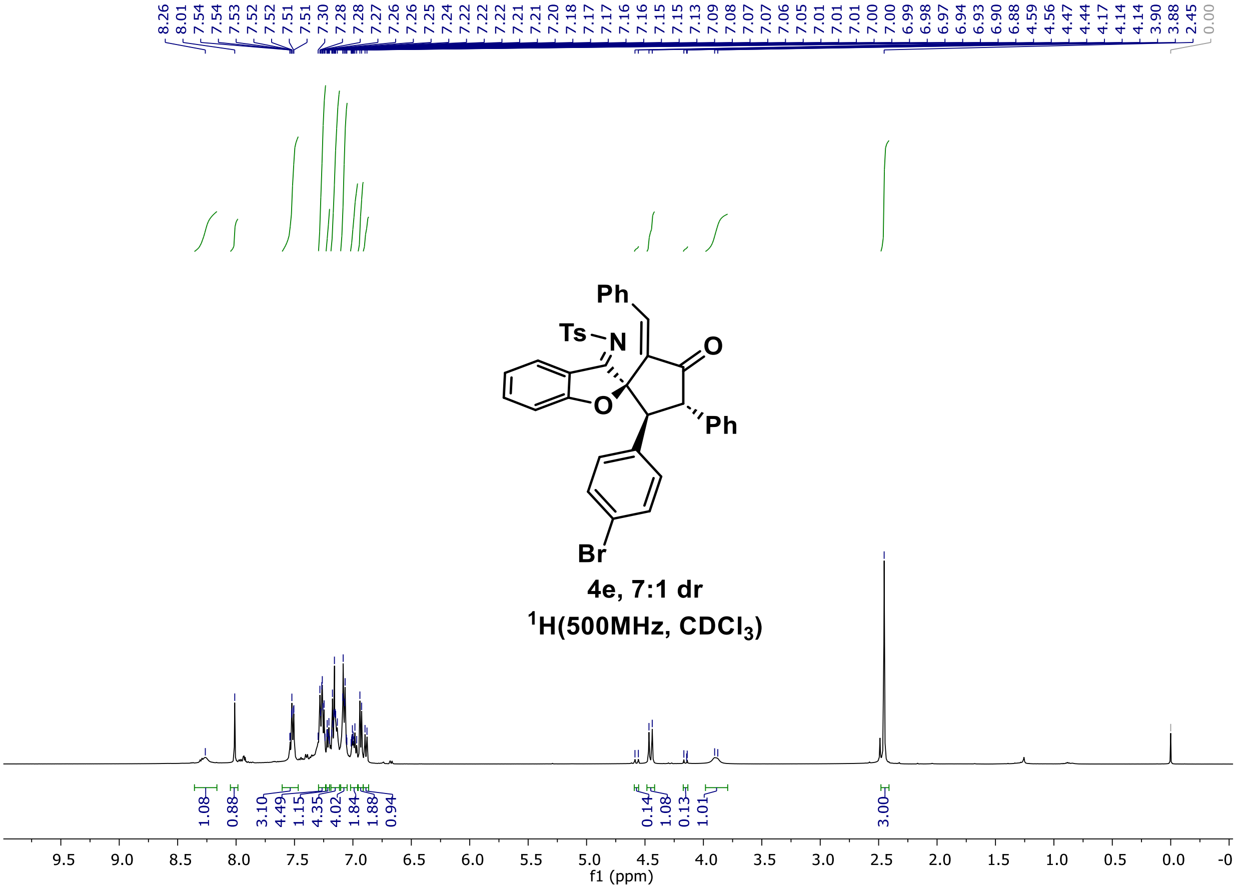
8.26 8.01 7.54 7.54 7.53 7.52 7.52 7.51 7.51 7.30 7.28 7.28 7.27 7.26 7.26 7.25 7.24 7.22 7.22 7.22 7.21 7.21 7.20 7.18 7.17 7.17 7.16 7.16 7.15 7.15 7.13 7.09 7.08 7.07 7.07 7.06 7.05 7.01 7.01 7.01 7.00 6.99 6.98 6.97 6.94 6.93 6.90 6.88 4.59 4.56 4.47 4.44 4.17 4.14 3.90 3.88 2.45 0.00
Ph
Ts
N
O
O
Ph
Br
4e, 7:1 dr
1H(500MHz, CDCl3)
1.08 0.88 3.10 4.49 1.15 4.35 4.02 1.84 1.88 0.94 0.14 1.08 0.13 1.01 3.00
9.5 9.0 8.5 8.0 7.5 7.0 6.5 6.0 5.5 5.0 4.5 4.0 3.5 3.0 2.5 2.0 1.5 1.0 0.5 0.0 -0
f1 (ppm)

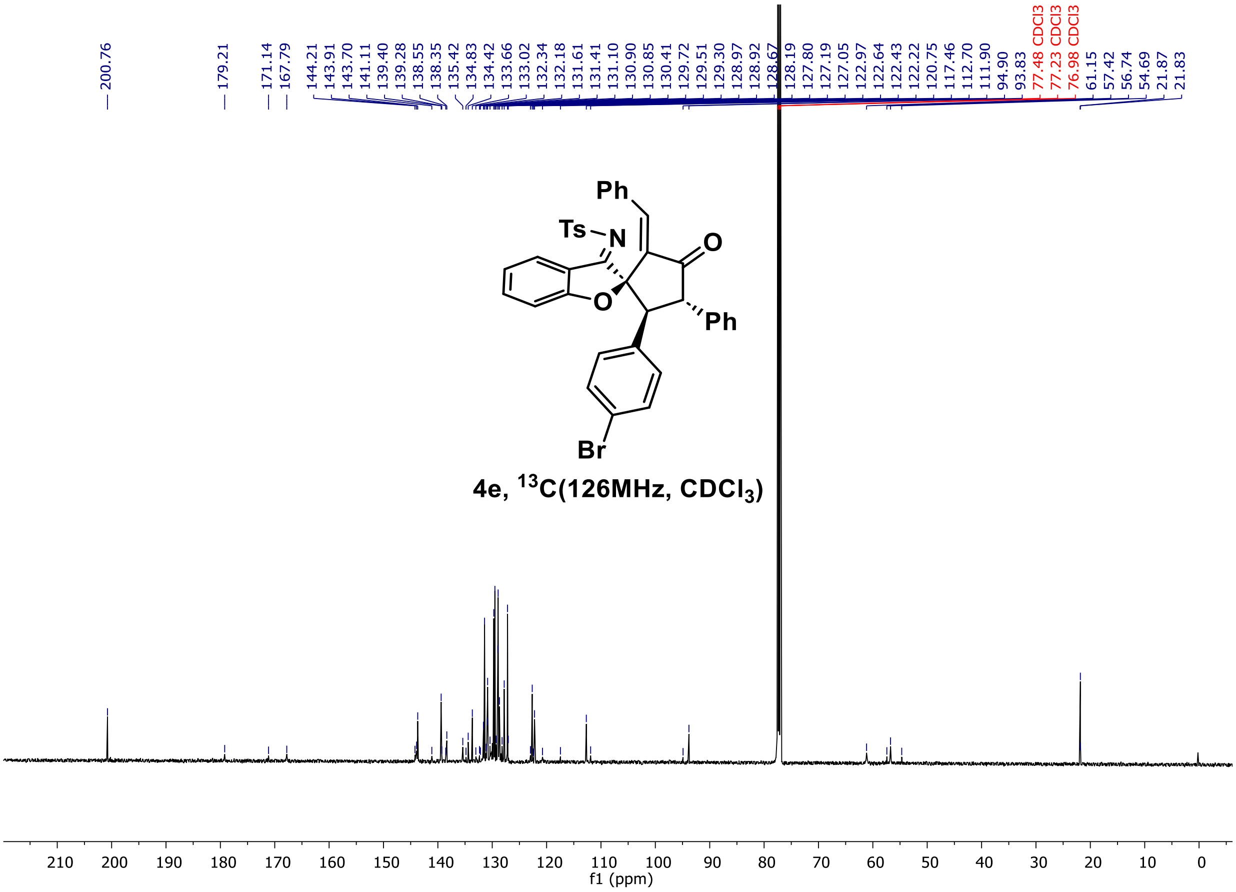
200.76 179.21 171.14 167.79 144.21 143.91 143.70 141.11 139.40 139.28 138.55 138.35 135.42 134.83 134.42 133.66 133.02 132.34 132.18 131.61 131.41 131.10 130.90 130.85 130.41 129.72 129.51 129.30 128.97 128.92 128.67 128.19 127.80 127.19 127.05 122.97 122.64 122.43 122.22 120.75 117.46 112.70 111.90 94.90 93.83 77.48 CDCl3 77.23 CDCl3 76.98 CDCl3 61.15 57.42 56.74 54.69 21.87 21.83
Ph
Ts
N
O
O
Ph
Br
4e, 13C(126MHz, CDCl3)
210 200 190 180 170 160 150 140 130 120 110 100 90 80 70 60 50 40 30 20 10 0
f1 (ppm)

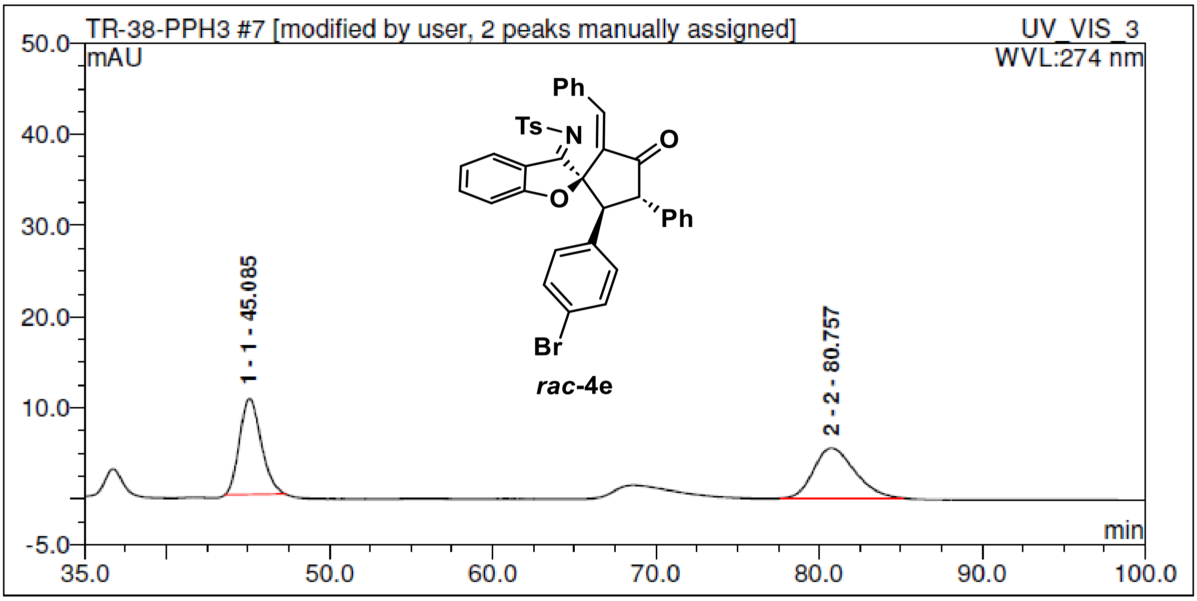

	Peak Name	Ret.Time (detected) min	Area mAU*min	Rel.Area(ident.) %	Height mAU	Amount
1	1	45.09	16.22445	50.15749296	10.50694	n.a.
2	2	80.76	16.123	49.84250704	5.508	n.a.

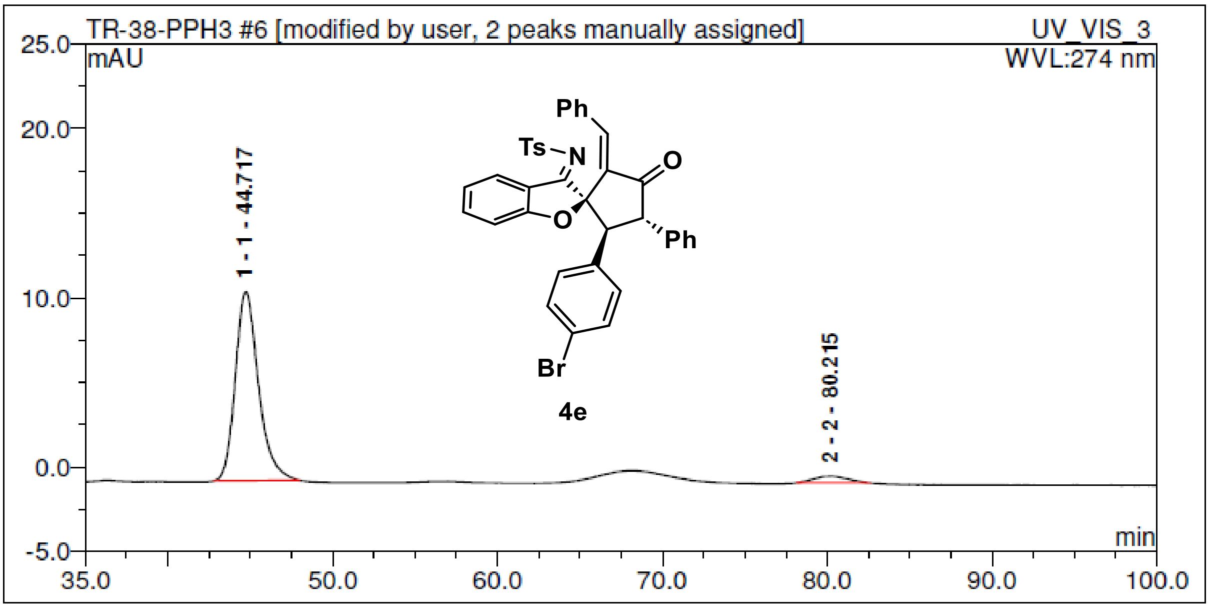

	Peak Name	Ret.Time (detected) min	Area mAU*min	Rel.Area(ident.) %	Height mAU	Amount
1	1	44.72	18.22969	95.14498327	11.15445	n.a.
2	2	80.22	0.930	4.855016733	0.396	n.a.

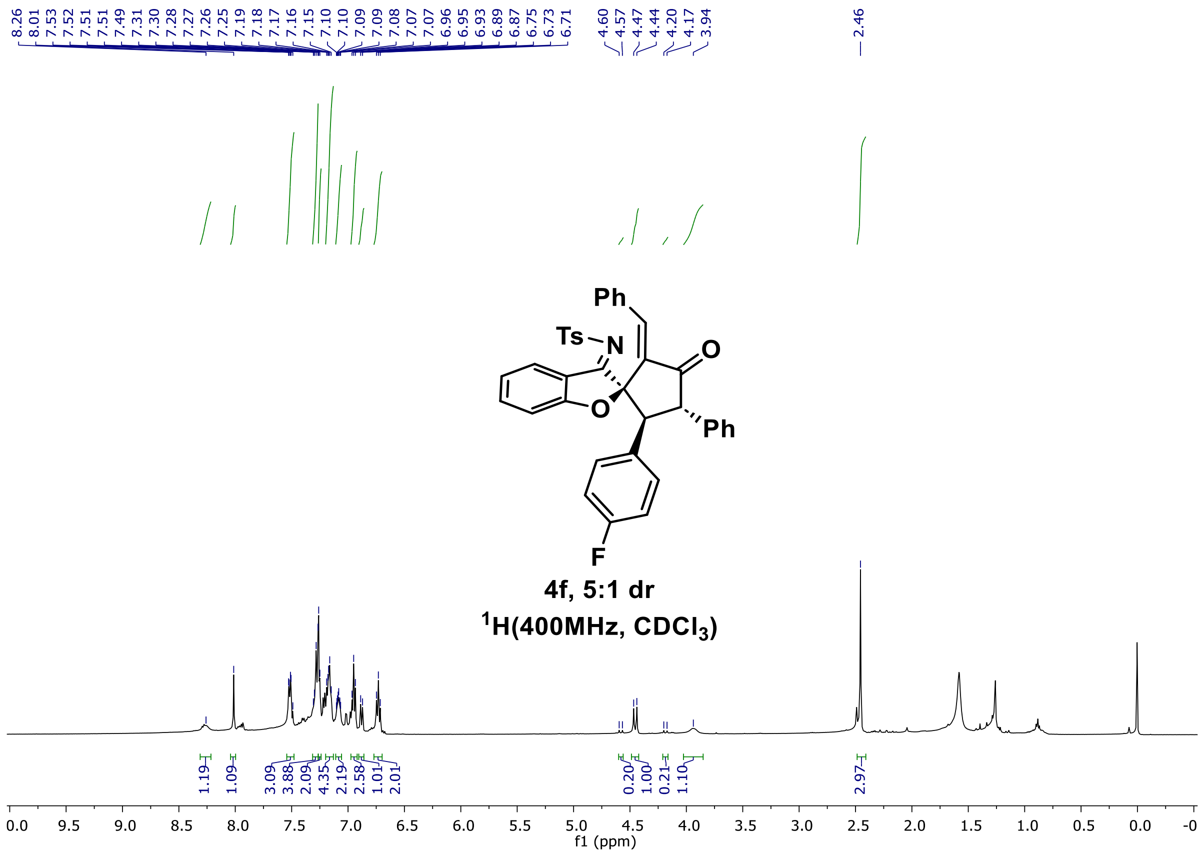
4f, 5:1 dr
^{1}H(400MHz, CDCl$_3$)
f1 (ppm)

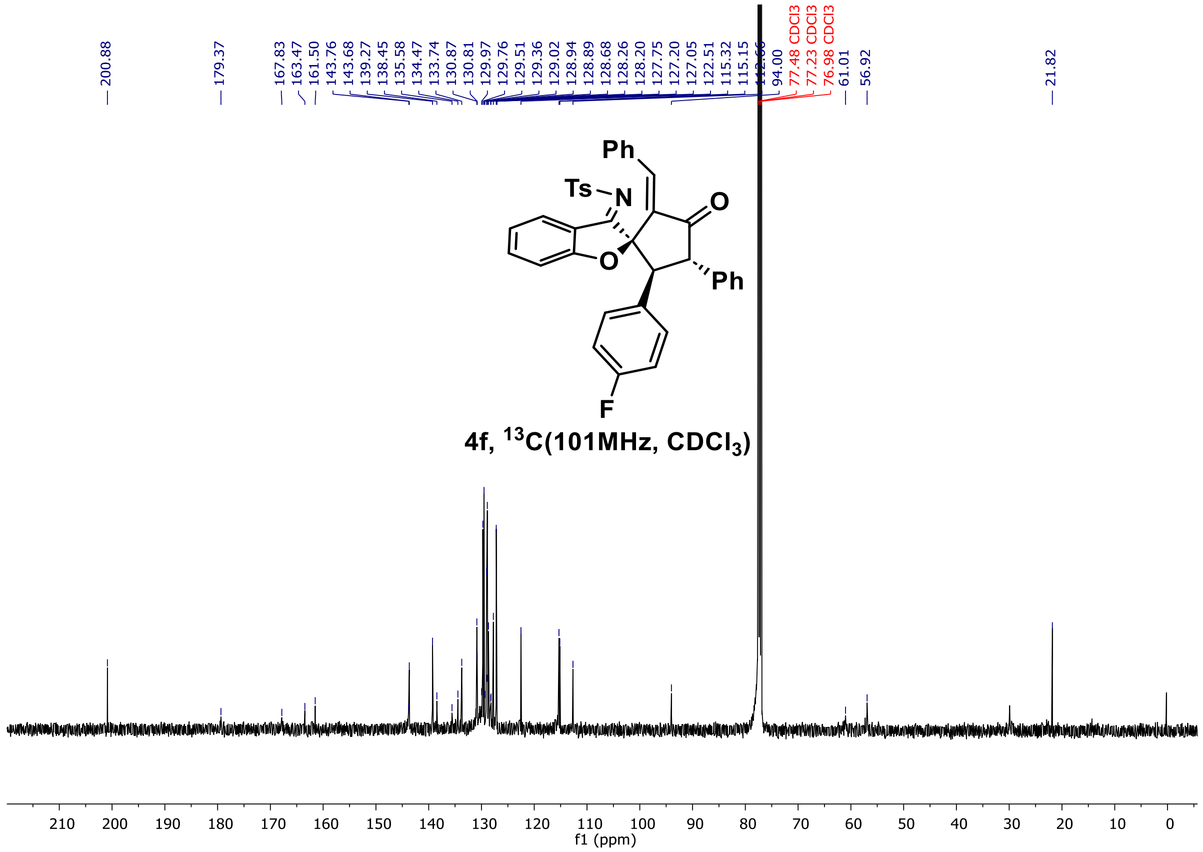
4f, ^{13}C(101MHz, CDCl$_3$)
77.48 CDCl3
77.23 CDCl3
76.98 CDCl3
f1 (ppm)

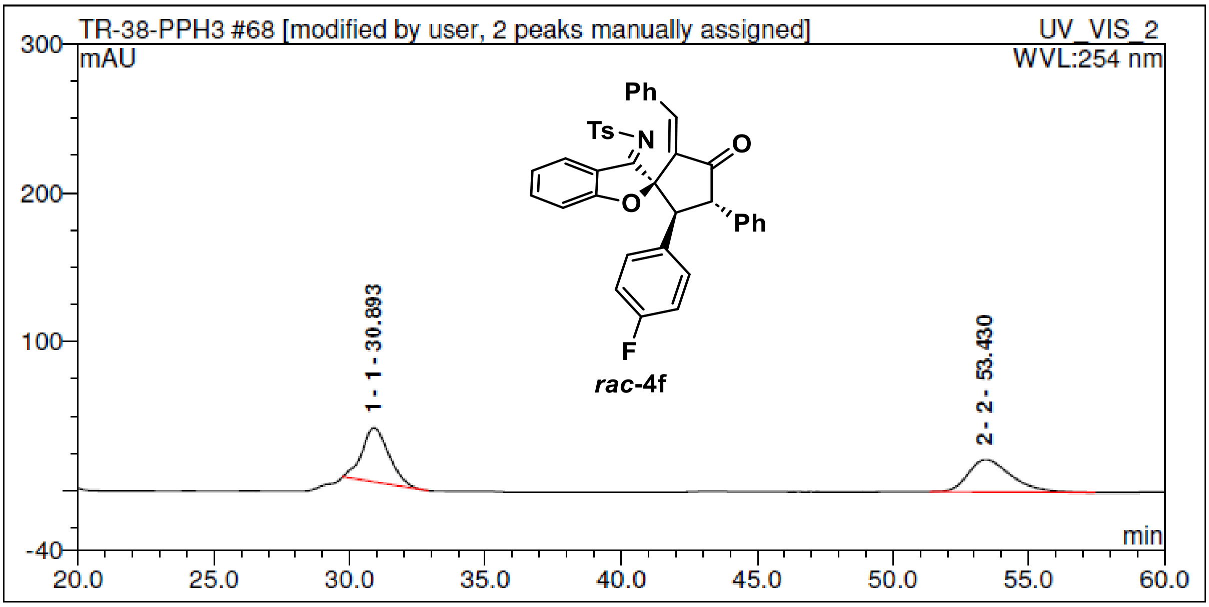

Peak Name		Ret.Time (detected) min	Area mAU*min	Rel.Area(ident.) %	Height mAU	Amount
1	1	30.89	41.67435	51.09812449	36.24735	n.a.
2	2	53.43	39.883	48.90187551	21.696	n.a.

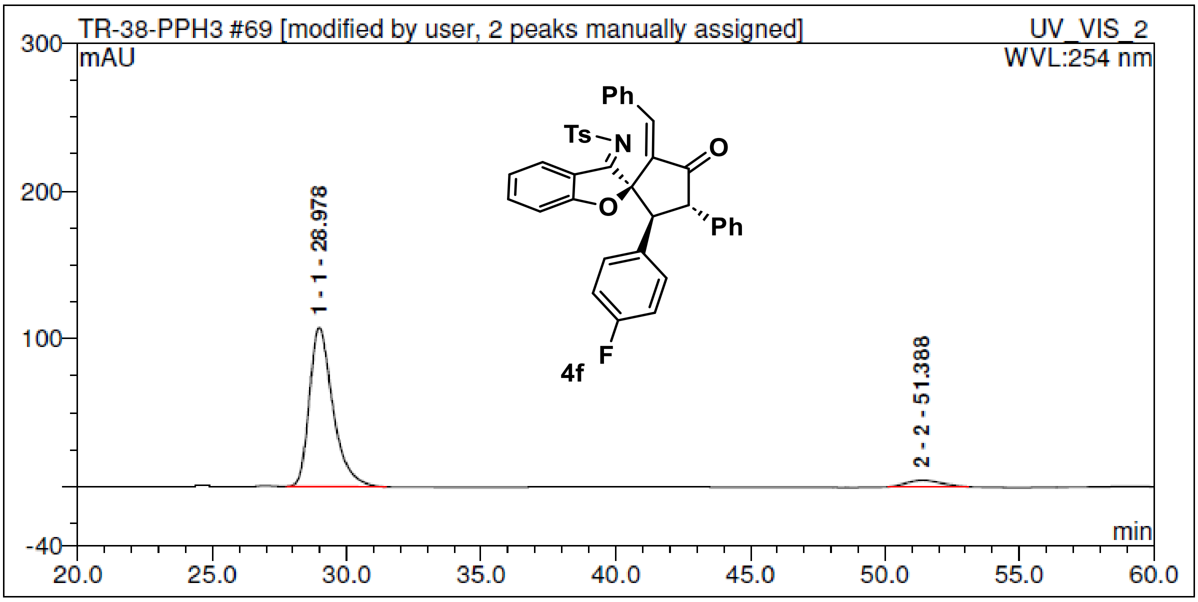

Peak Name		Ret.Time (detected) min	Area mAU*min	Rel.Area(ident.) %	Height mAU	Amount
1	1	28.98	108.8931	94.4147708	107.2221	n.a.
2	2	51.39	6.442	5.585229195	4.291	n.a.

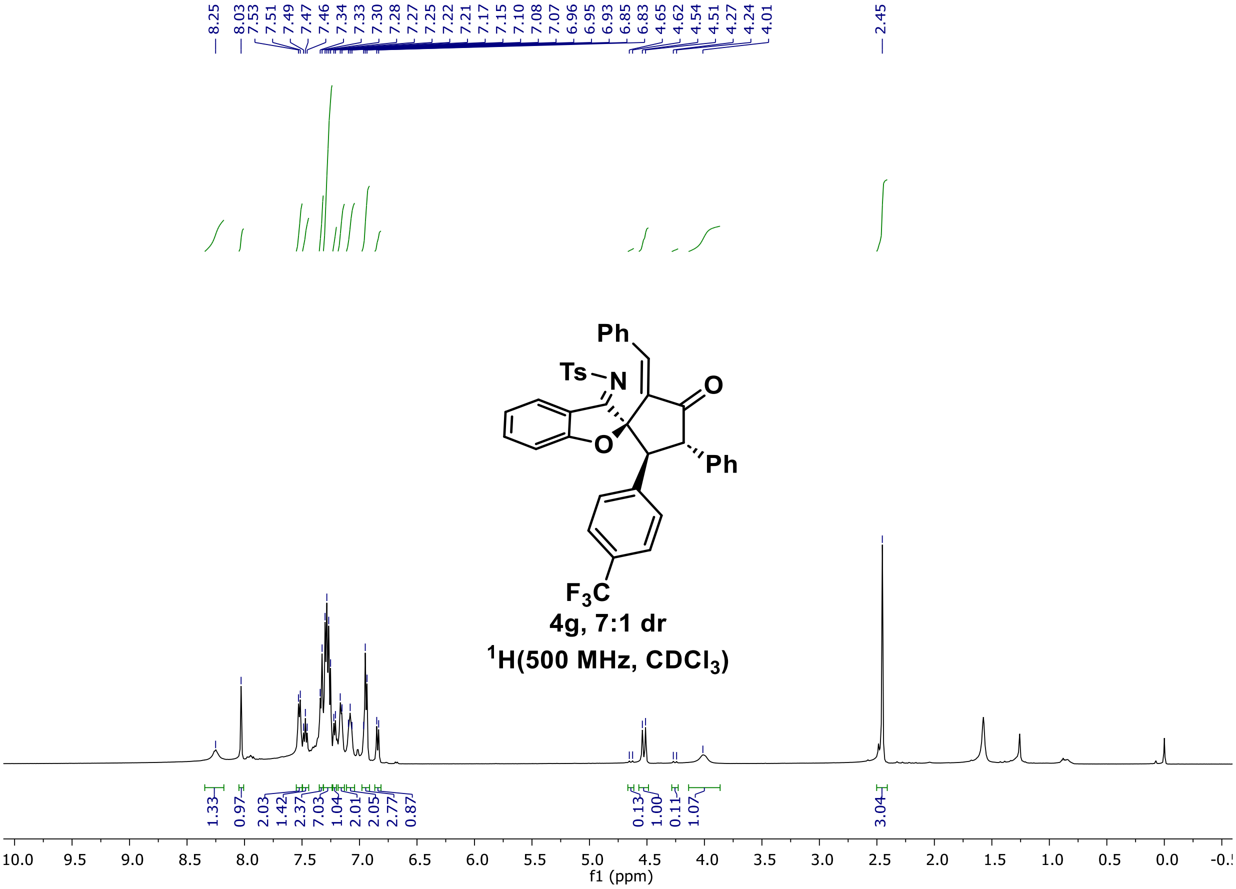
8.25
8.03
7.53
7.51
7.49
7.47
7.46
7.34
7.33
7.30
7.28
7.27
7.25
7.22
7.21
7.17
7.15
7.10
7.08
7.07
6.96
6.95
6.93
6.85
6.83
4.65
4.62
4.54
4.51
4.27
4.24
4.01
2.45
Ph
Ts
N
O
O
Ph
F3C
4g, 7:1 dr
1H(500 MHz, CDCl3)
1.33
0.97
2.03
1.42
2.37
7.03
1.04
2.01
2.05
2.77
0.87
0.13
1.00
0.11
1.07
3.04
f1 (ppm)

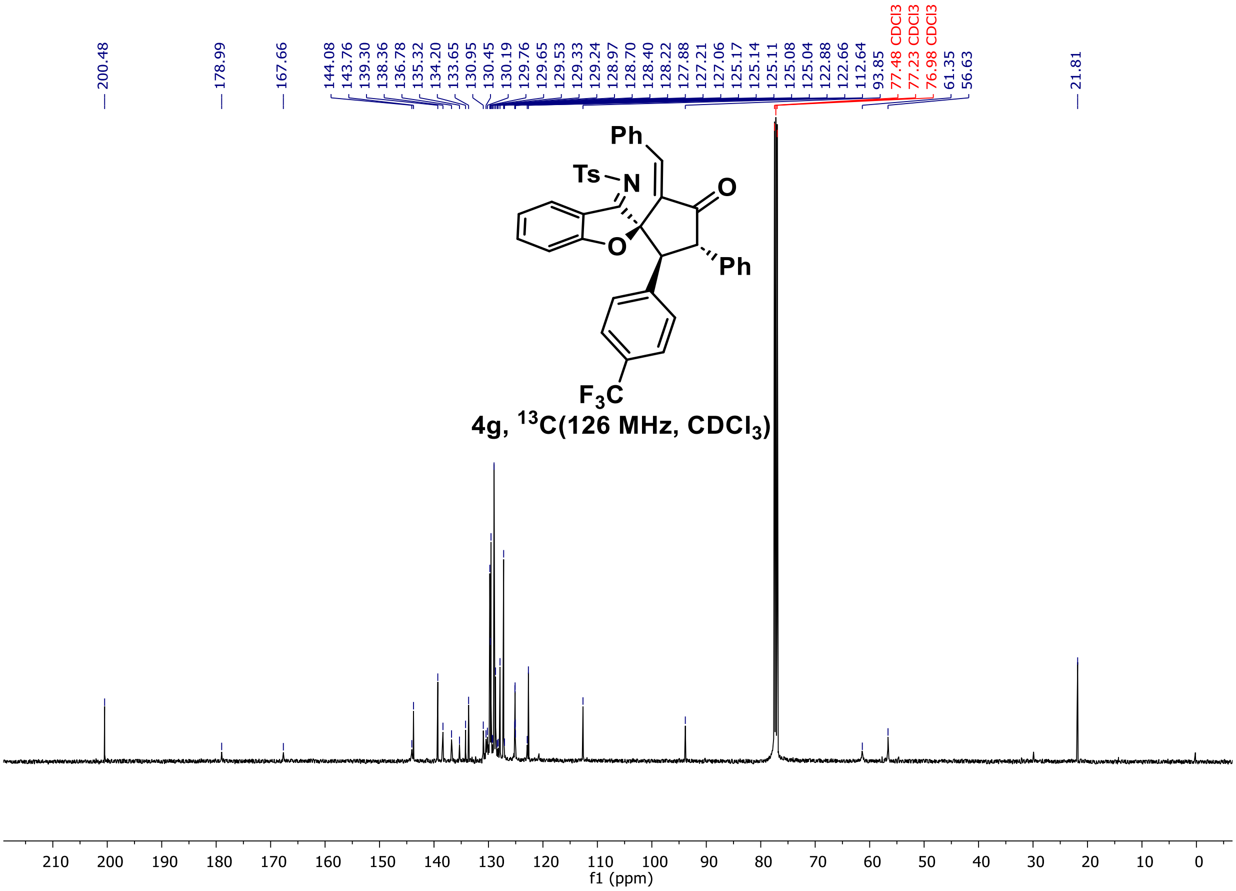
200.48
178.99
167.66
144.08
143.76
139.30
138.36
136.78
135.32
134.20
133.65
130.95
130.45
130.19
129.76
129.65
129.53
129.33
129.24
128.97
128.70
128.40
128.22
127.88
127.21
127.06
125.17
125.14
125.11
125.08
125.04
122.88
122.66
112.64
93.85
77.48 CDCl3
77.23 CDCl3
76.98 CDCl3
61.35
56.63
21.81
Ph
Ts
N
O
O
Ph
F3C
4g, 13C(126 MHz, CDCl3)
f1 (ppm)

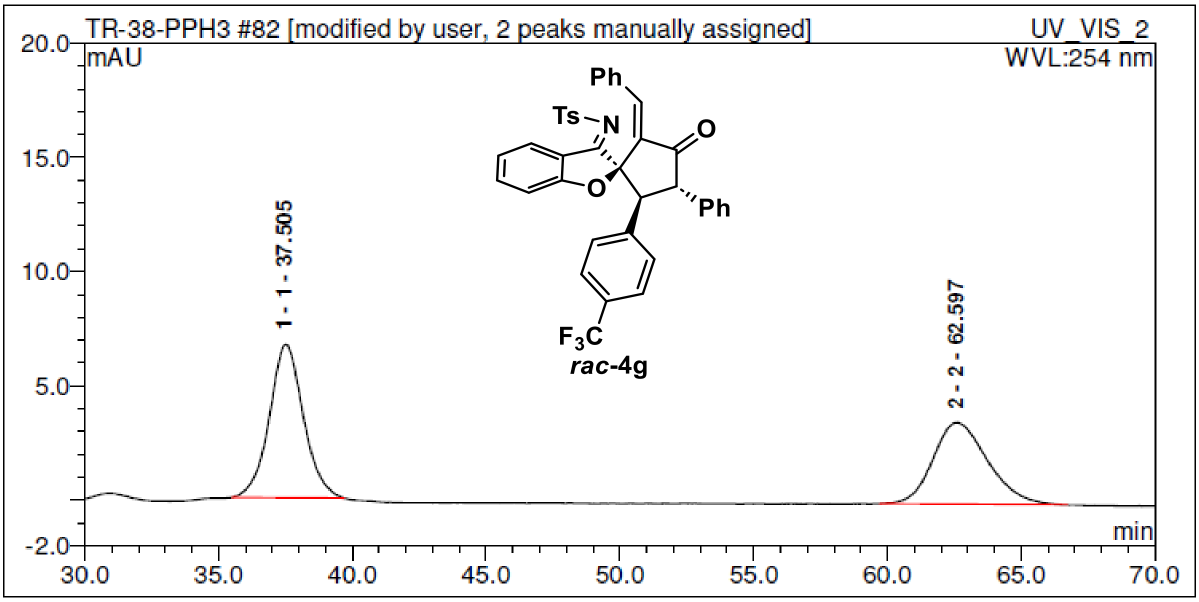

	Peak Name	Ret.Time (detected) min	Area mAU*min	Rel.Area(ident.) %	Height mAU	Amount
1	1	37.51	9.910386	53.90258566	6.70198	n.a.
2	2	62.60	8.475	46.09741434	3.569	n.a.

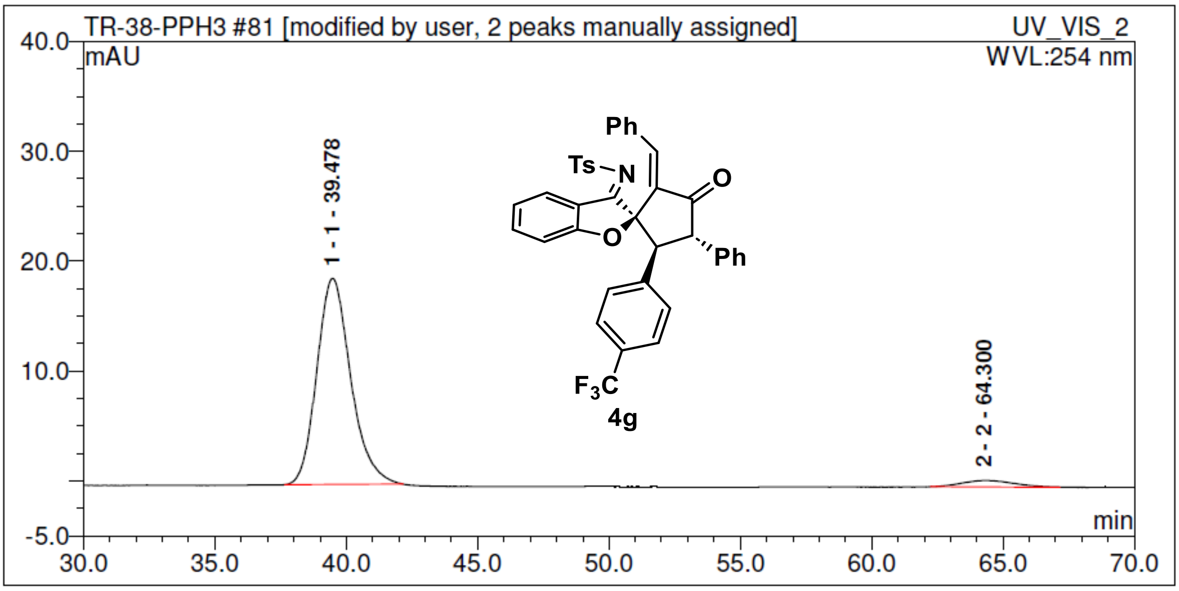

	Peak Name	Ret.Time (detected) min	Area mAU*min	Rel.Area(ident.) %	Height mAU	Amount
1	1	39.48	28.06724	95.40284578	18.76341	n.a.
2	2	64.30	1.352	4.597154221	0.600	n.a.

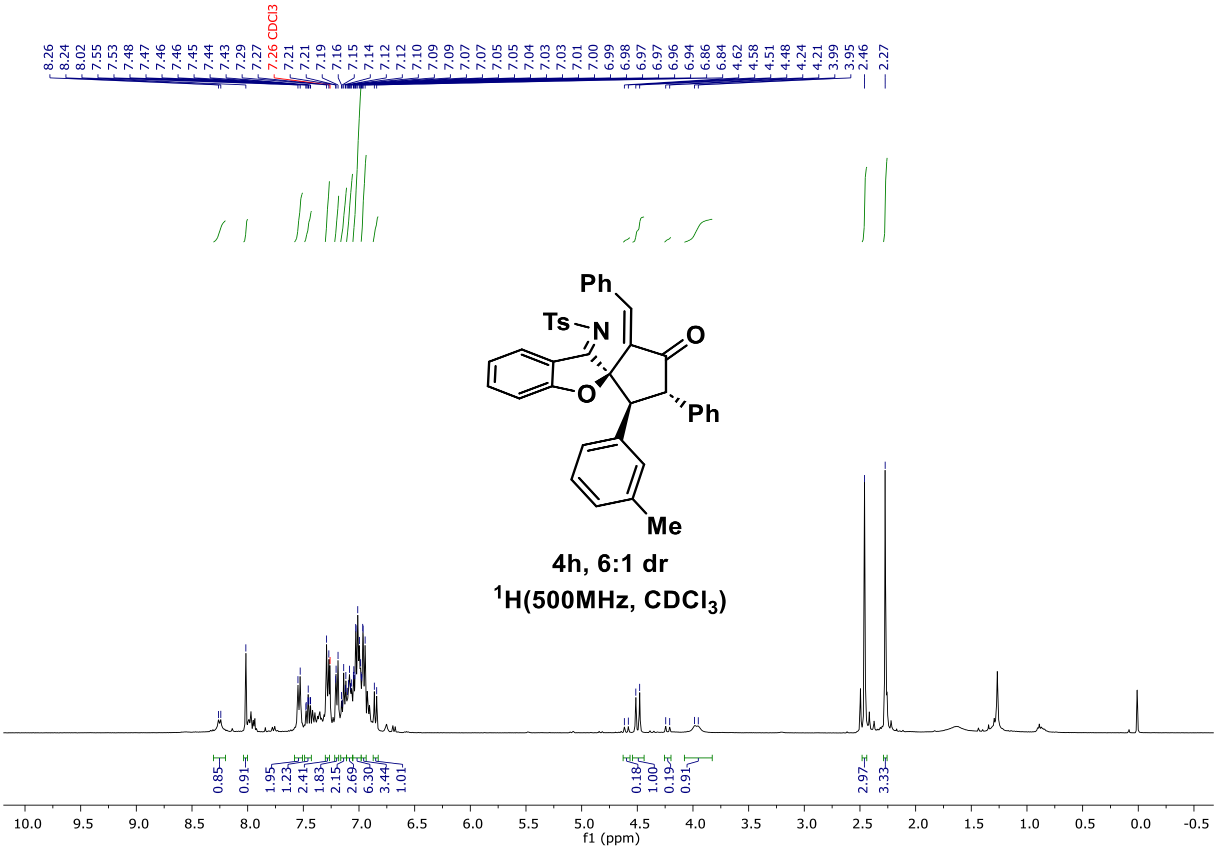
Ph
Ts
N
O
O
Ph
Me
4h, 6:1 dr
1H(500MHz, CDCl3)
f1 (ppm)

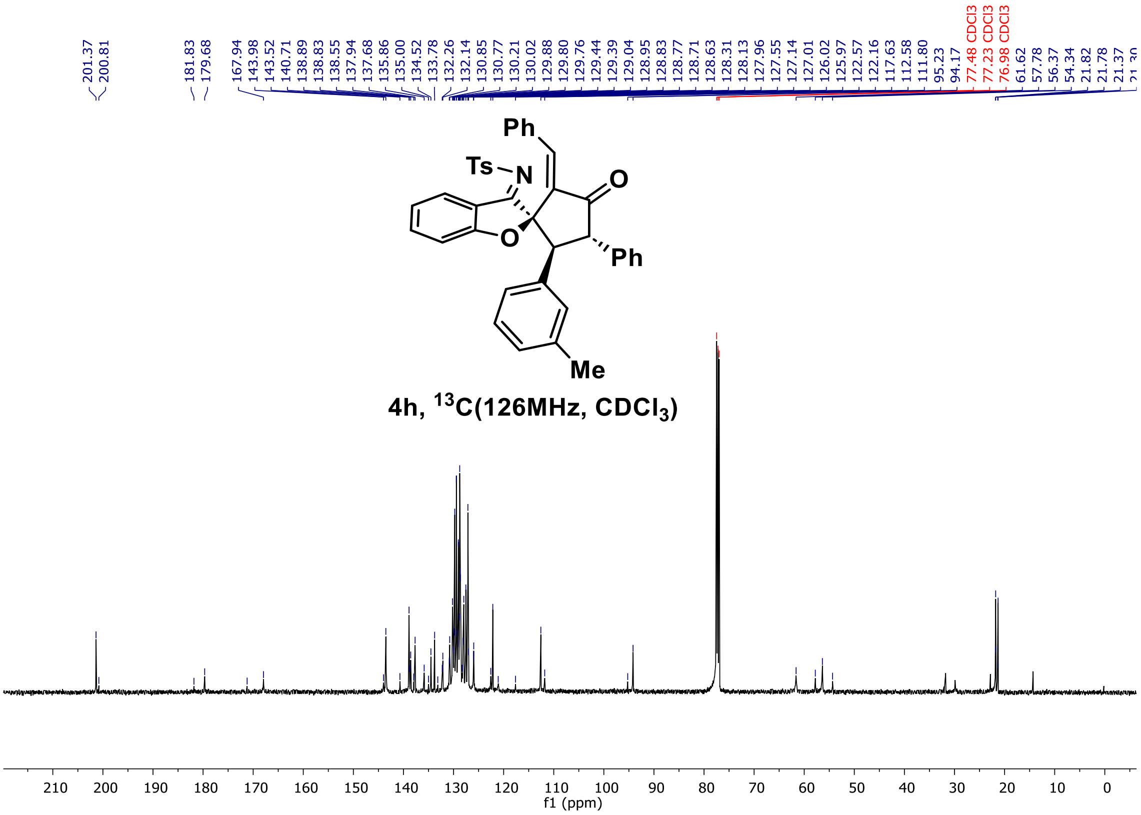
Ph
Ts
N
O
O
Ph
Me
4h, 13C(126MHz, CDCl3)
f1 (ppm)

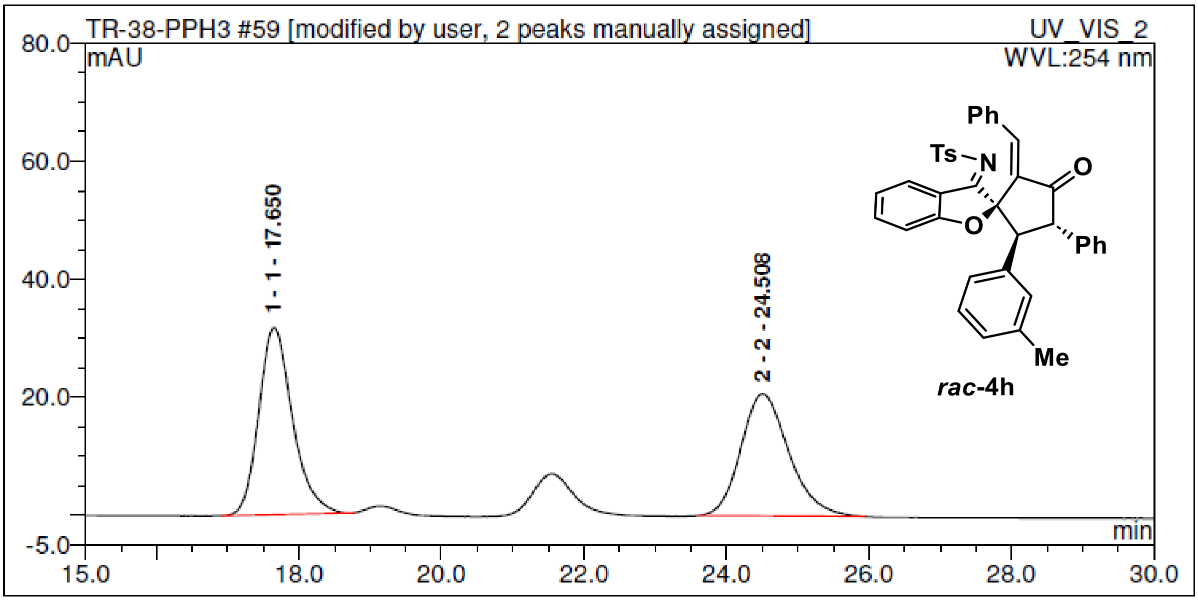

Peak Name	Ret.Time (detected) min	Area mAU*min	Rel.Area(ident.) %	Height mAU	Amount
1 1	17.65	17.38231	51.77620323	31.66638	n.a.
2 2	24.51	16.190	48.22379677	20.666	n.a.

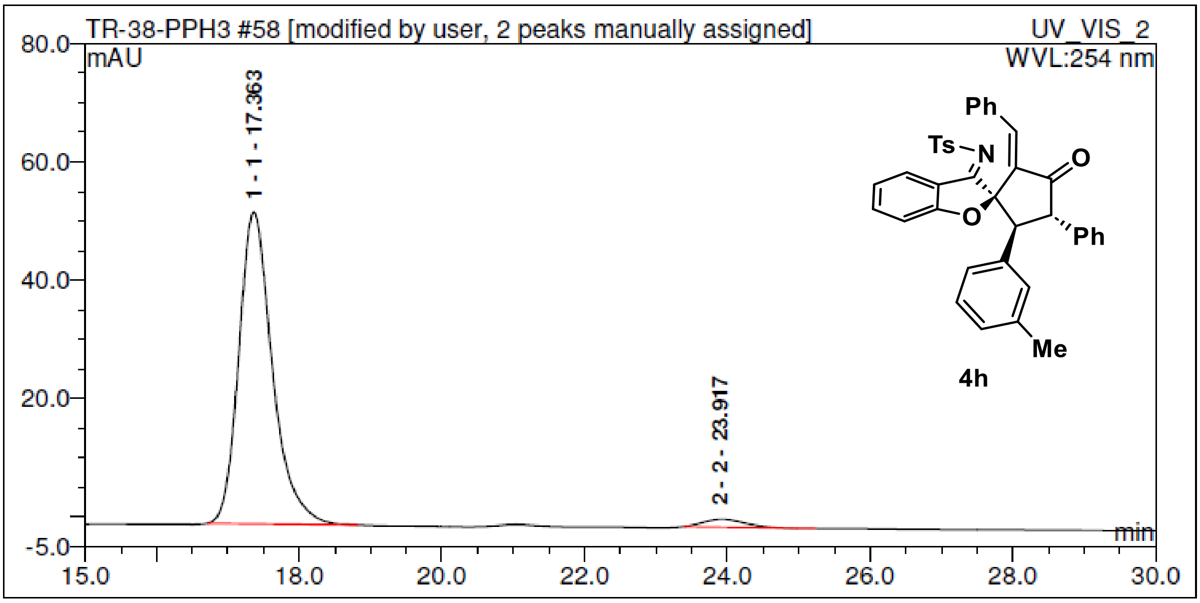

Peak Name	Ret.Time (detected) min	Area mAU*min	Rel.Area(ident.) %	Height mAU	Amount
1 1	17.36	28.69547	97.03752584	52.6323	n.a.
2 2	23.92	0.876	2.962474159	1.328	n.a.

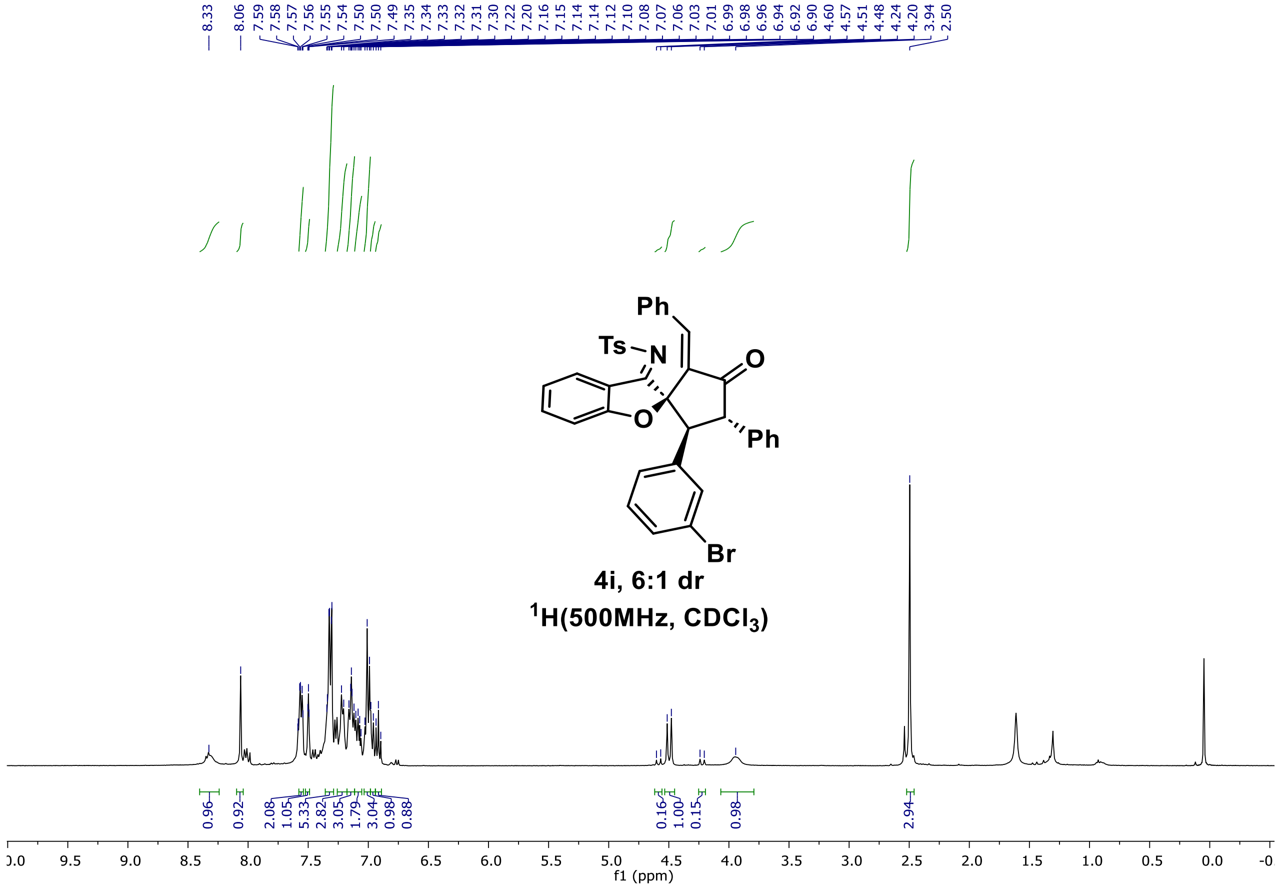
8.33
8.06
7.59
7.58
7.57
7.56
7.55
7.54
7.50
7.49
7.35
7.34
7.33
7.32
7.31
7.30
7.22
7.20
7.16
7.15
7.14
7.14
7.12
7.10
7.08
7.07
7.06
7.03
7.01
6.99
6.98
6.96
6.94
6.92
6.90
4.60
4.57
4.51
4.48
4.24
4.20
3.94
2.50
Ph
Ts
N
O
O
Ph
Br
4i, 6:1 dr
^{1}H(500MHz, CDCl$_3$)
0.96
0.92
2.08
1.05
5.33
2.82
3.05
1.79
3.04
0.98
0.88
0.16
1.00
0.15
0.98
2.94
f1 (ppm)

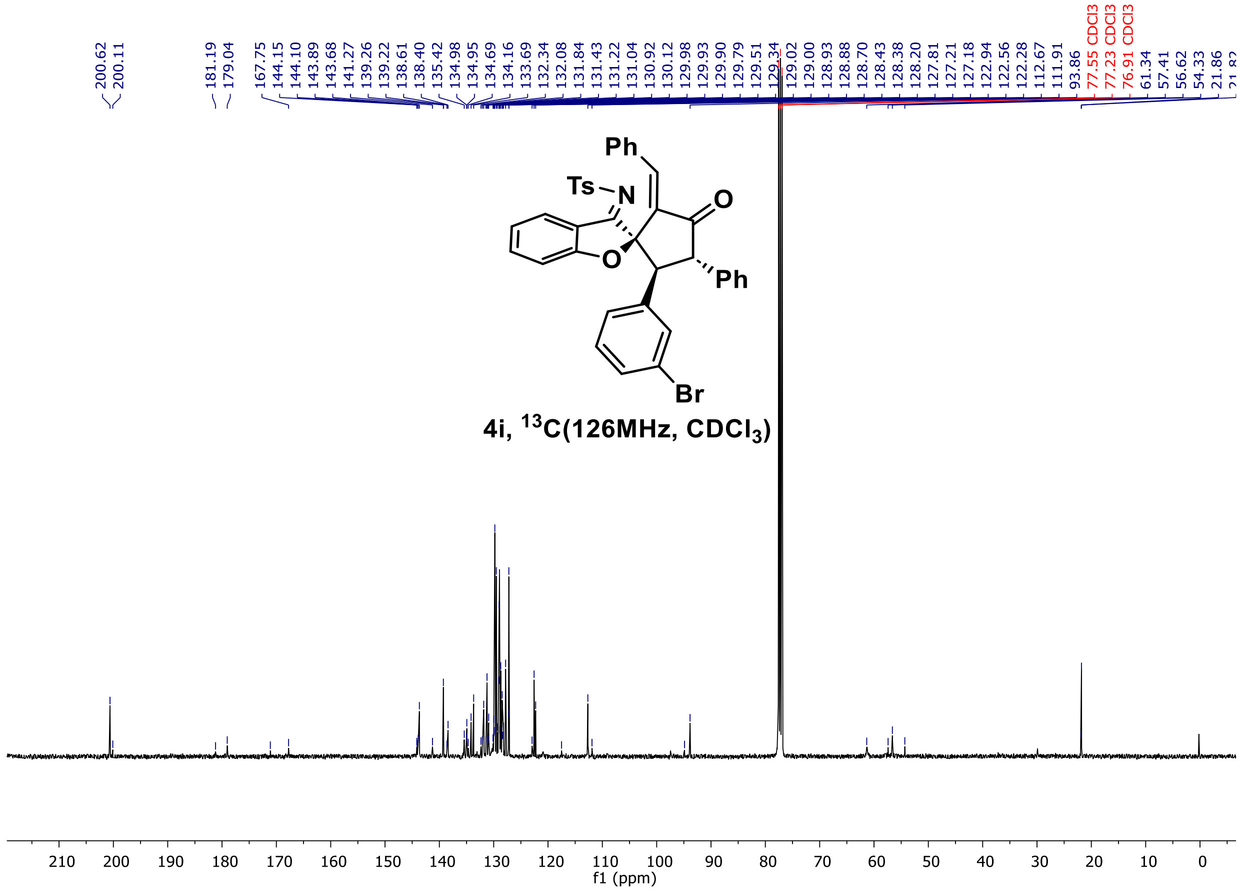
200.62
200.11
181.19
179.04
167.75
144.15
144.10
143.89
143.68
141.27
139.26
139.22
138.61
138.40
135.42
134.98
134.95
134.69
134.16
133.69
132.34
132.08
131.84
131.43
131.22
131.04
130.92
130.12
129.98
129.93
129.90
129.79
129.51
129.34
129.02
129.00
128.93
128.88
128.70
128.43
128.38
128.20
127.81
127.21
127.18
122.94
122.56
122.28
112.67
111.91
93.86
77.55 CDCl3
77.23 CDCl3
76.91 CDCl3
61.34
57.41
56.62
54.33
21.86
Ph
Ts
N
O
O
Ph
Br
4i, ^{13}C(126MHz, CDCl$_3$)
f1 (ppm)

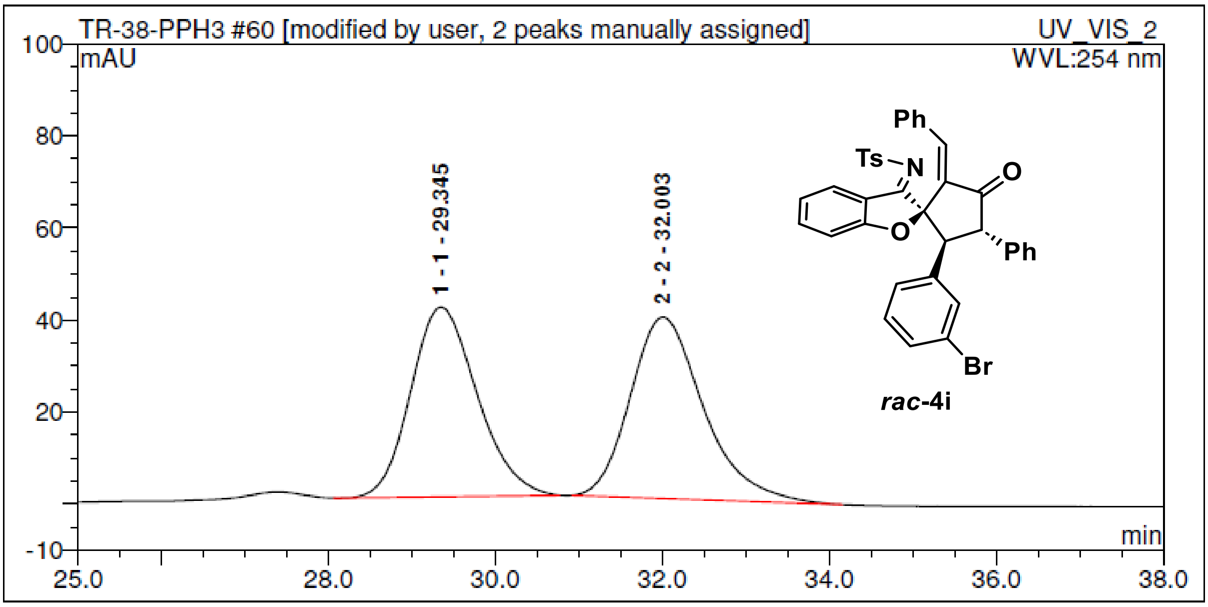

Peak Name	Ret.Time (detected) min	Area mAU*min	Rel.Area(ident.) %	Height mAU	Amount
1 1	29.35	38.02729	48.65895907	41.21283	n.a.
2 2	32.00	40.123	51.34104093	39.374	n.a.

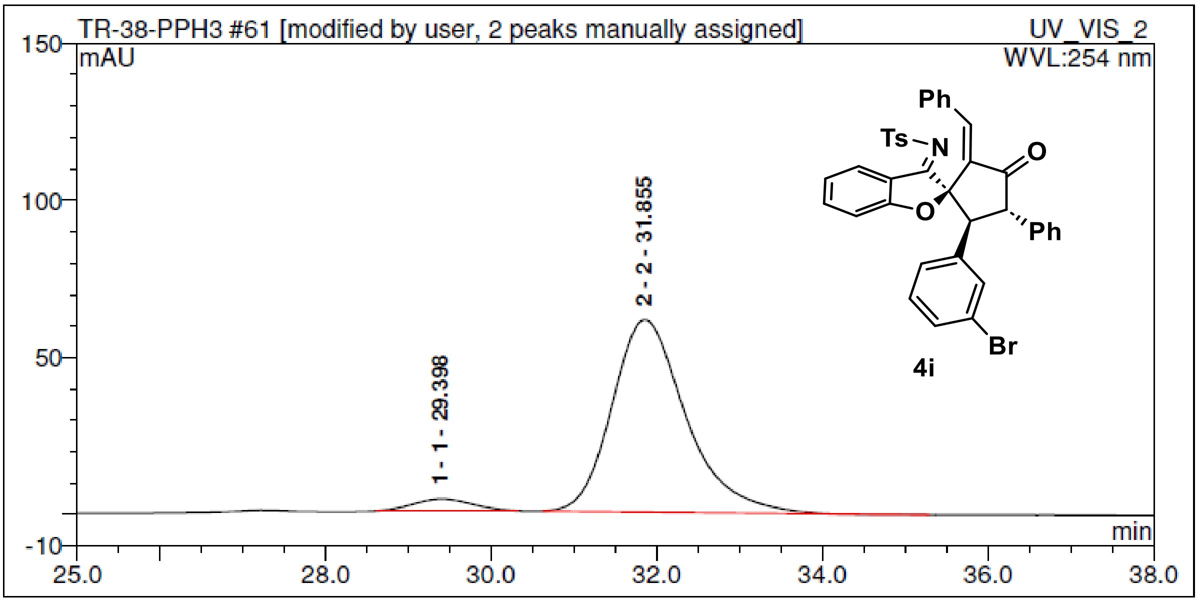

Peak Name	Ret.Time (detected) min	Area mAU*min	Rel.Area(ident.) %	Height mAU	Amount
1 1	29.40	3.108808	4.68021073	3.7932	n.a.
2 2	31.86	63.316	95.31978927	61.205	n.a.

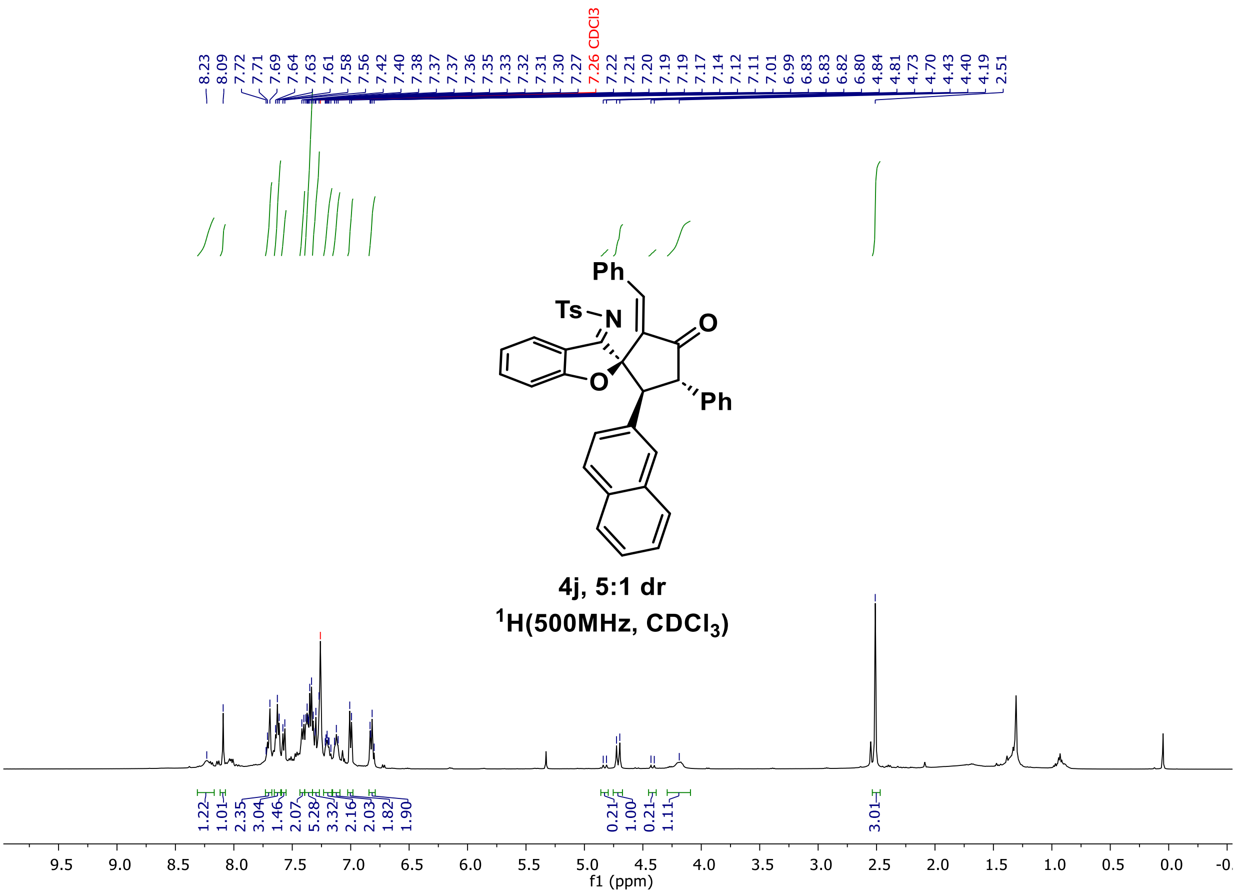
8.23
8.09
7.72
7.71
7.69
7.64
7.63
7.61
7.58
7.56
7.42
7.40
7.38
7.37
7.36
7.35
7.33
7.32
7.31
7.30
7.27
7.26 CDCl3
7.22
7.21
7.20
7.19
7.19
7.17
7.14
7.12
7.11
7.01
6.99
6.83
6.83
6.82
6.80
4.84
4.81
4.73
4.70
4.43
4.40
4.19
2.51
Ph
Ts
N
O
O
Ph
4j, 5:1 dr
^{1}H(500MHz, CDCl$_3$)
1.22
1.01
2.35
3.04
1.46
2.07
5.28
3.32
2.16
2.03
1.82
1.90
0.21
1.00
0.21
1.11
3.01
f1 (ppm)

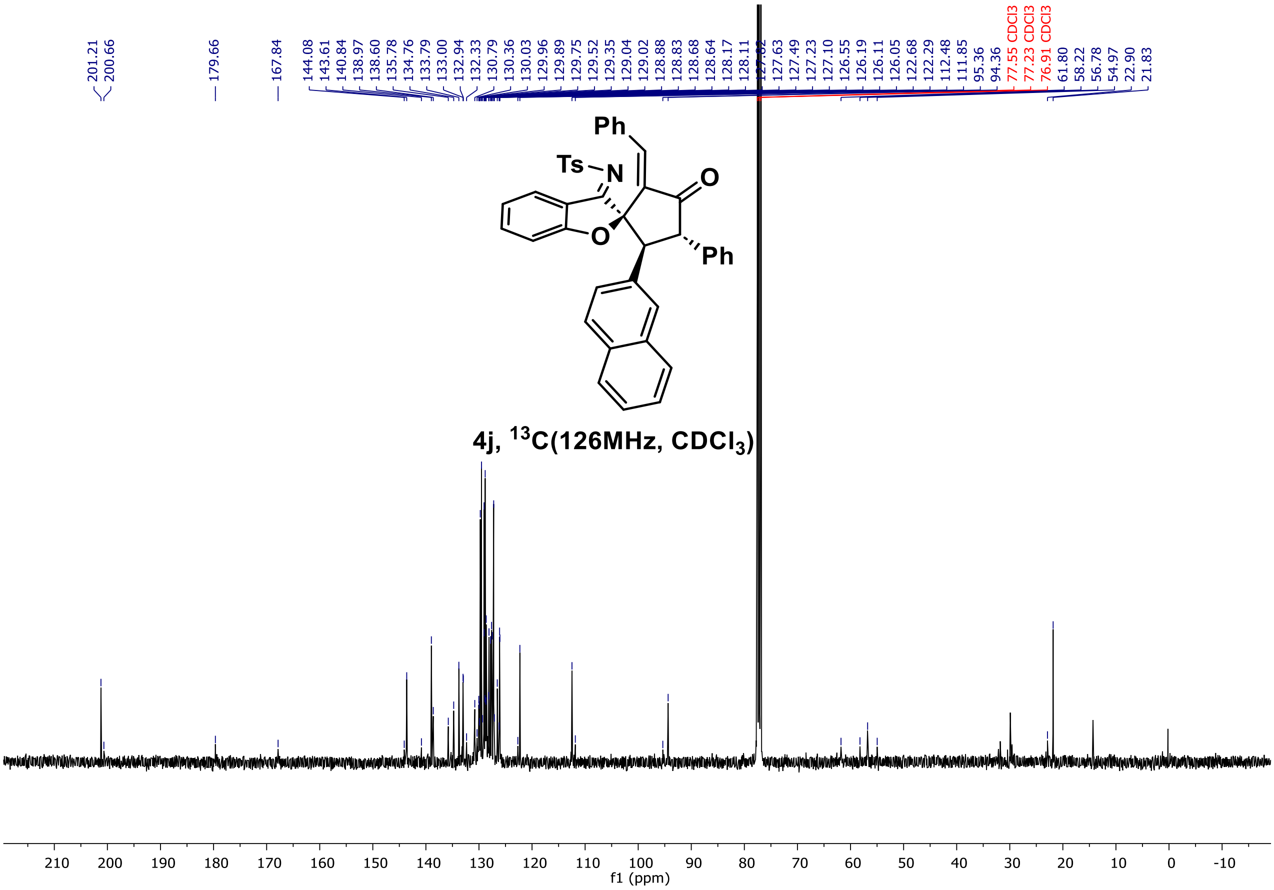
201.21
200.66
179.66
167.84
144.08
143.61
140.84
138.97
138.60
135.78
134.76
133.79
133.00
132.94
132.33
130.79
130.36
130.03
129.96
129.89
129.75
129.52
129.35
129.04
129.02
128.88
128.83
128.68
128.64
128.17
128.11
127.63
127.49
127.23
127.10
126.55
126.19
126.11
126.05
122.68
122.29
112.48
111.85
95.36
94.36
77.55 CDCl3
77.23 CDCl3
76.91 CDCl3
61.80
58.22
56.78
54.97
22.90
21.83
Ph
Ts
N
O
O
Ph
4j, ^{13}C(126MHz, CDCl$_3$)
f1 (ppm)

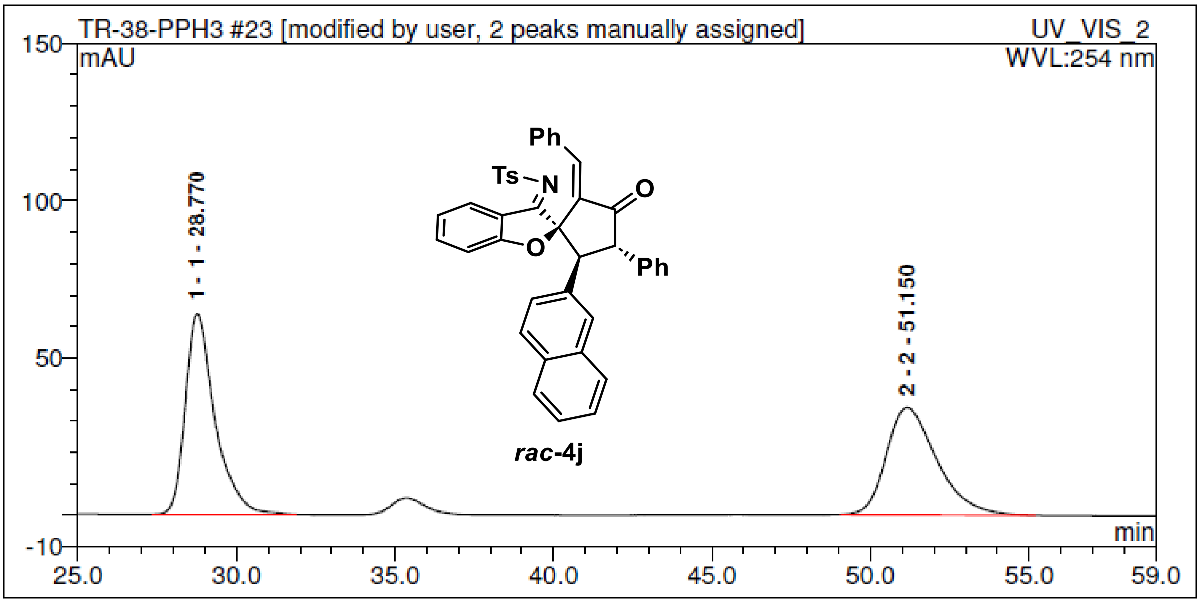

Peak Name	Ret.Time (detected) min	Area mAU*min	Rel.Area(ident.) %	Height mAU	Amount
1 1	28.77	68.97712	52.31836393	63.72139	n.a.
2 2	51.15	62.864	47.68163607	34.054	n.a.

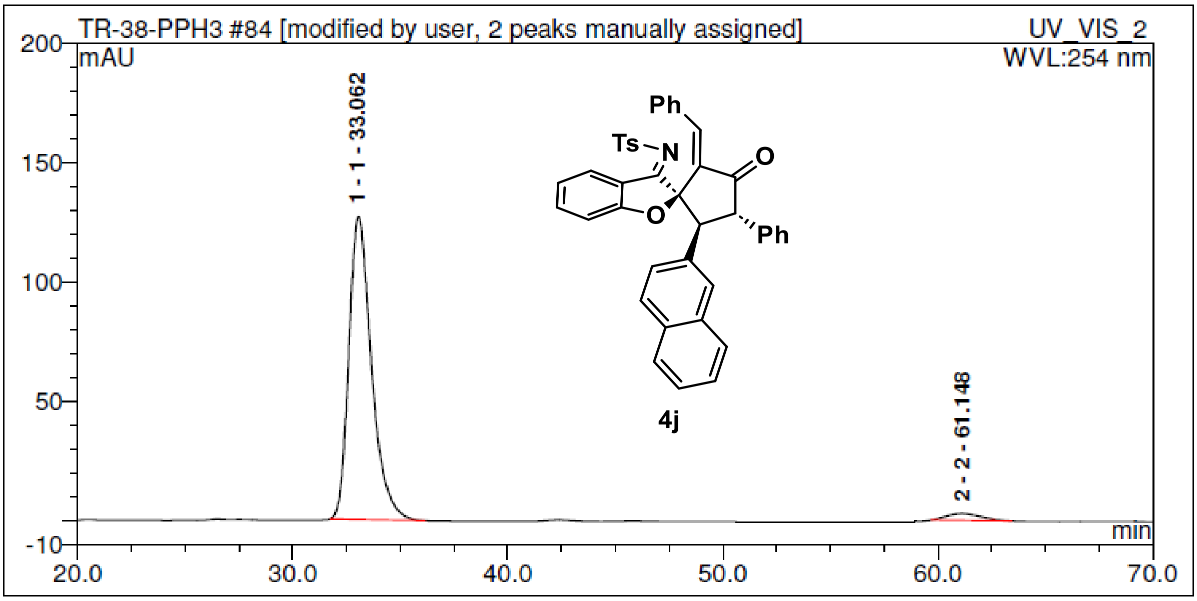

Peak Name	Ret.Time (detected) min	Area mAU*min	Rel.Area(ident.) %	Height mAU	Amount
1 1	33.06	154.7893	96.72712488	126.6964	n.a.
2 2	61.15	5.237	3.272875119	2.814	n.a.

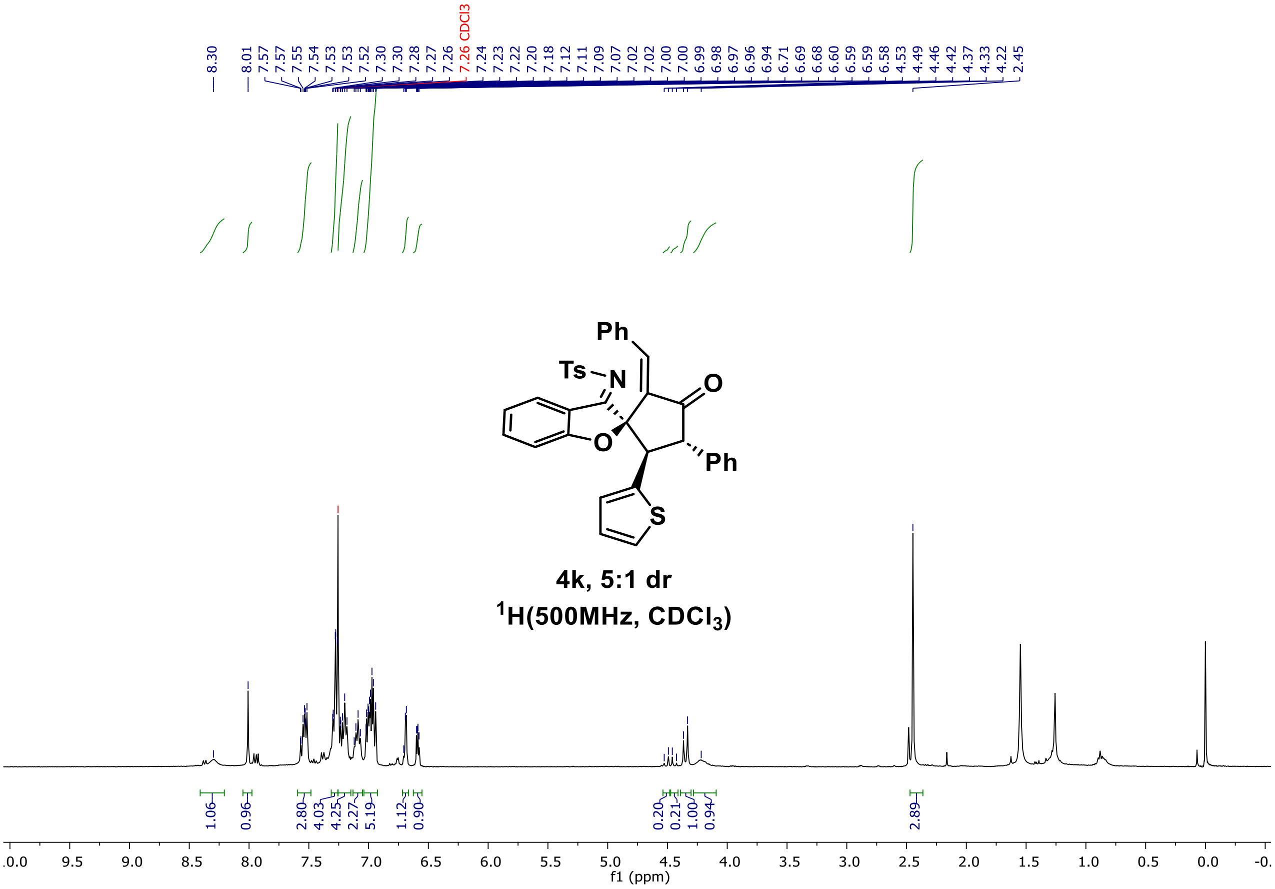
8.30 8.01 7.57 7.55 7.54 7.53 7.53 7.52 7.30 7.30 7.28 7.27 7.26 7.24 7.23 7.22 7.20 7.18 7.12 7.11 7.09 7.07 7.02 7.02 7.02 7.00 7.00 6.99 6.98 6.97 6.96 6.94 6.71 6.69 6.68 6.60 6.59 6.59 6.58 4.53 4.49 4.46 4.42 4.37 4.33 4.22 2.45
7.26 CDCl3
Ph
Ts
N
O
O
Ph
S
4k, 5:1 dr
^{1}H(500MHz, $CDCl_3$)
1.06 0.96 2.80 4.03 4.25 2.27 5.19 1.12 0.90 0.20 0.21 1.00 0.94 2.89
f1 (ppm)

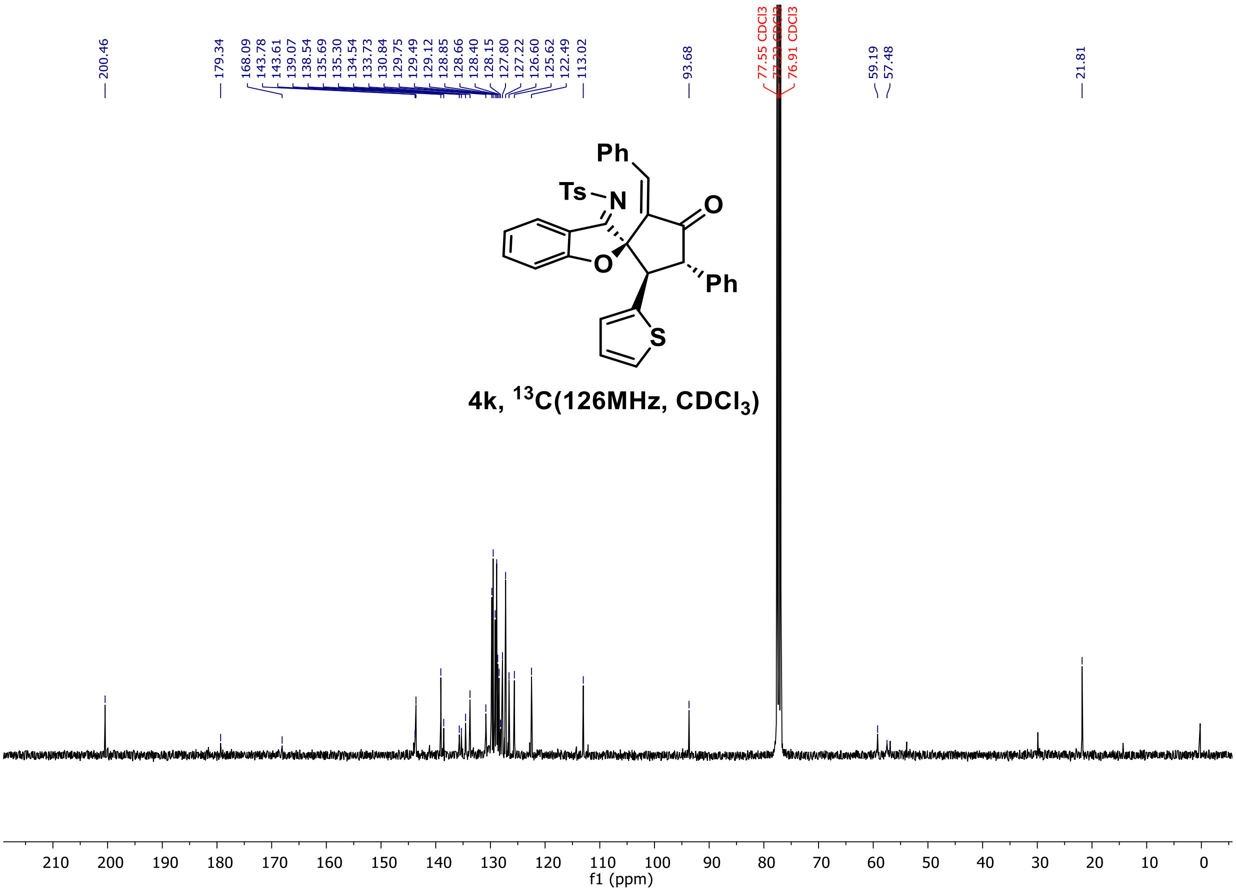
200.46 179.34 168.09 143.78 143.61 139.07 138.54 135.69 135.30 134.54 133.73 130.84 129.75 129.49 129.12 128.85 128.66 128.40 128.15 127.80 127.22 126.60 125.62 122.49 113.02 93.68 59.19 57.48 21.81
77.55 CDCl3 77.23 CDCl3 76.91 CDCl3
Ph
Ts
N
O
O
Ph
S
4k, ^{13}C(126MHz, $CDCl_3$)
f1 (ppm)

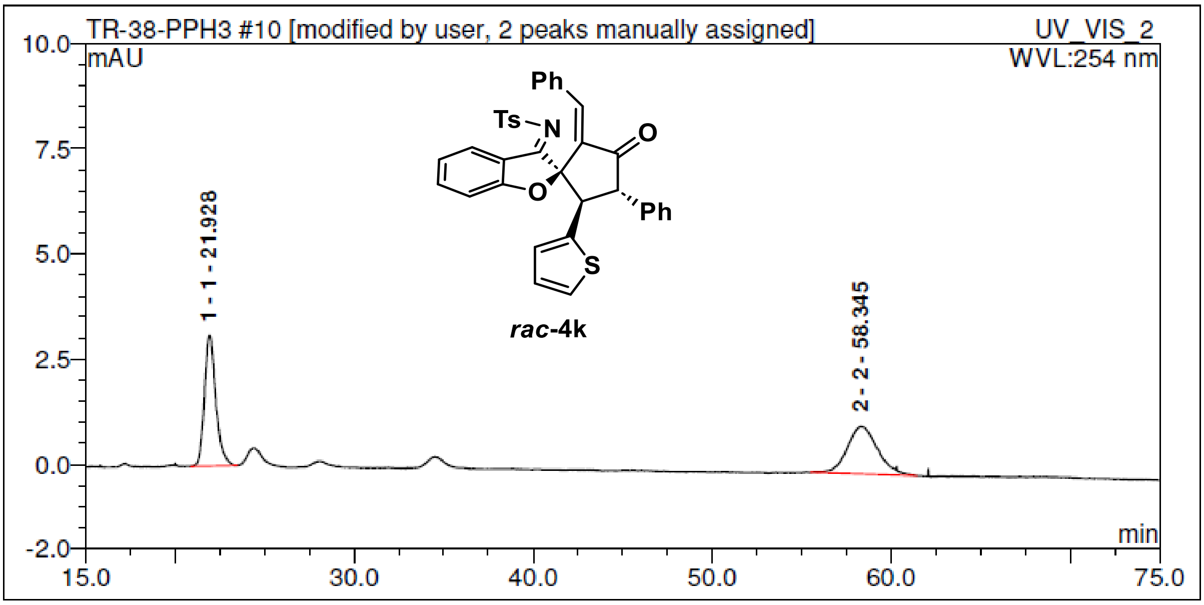

Peak Name		Ret.Time (detected) min	Area mAU*min	Rel.Area(ident.) %	Height mAU	Amount
1	1	21.93	2.294709	50.77919131	3.10386	n.a.
2	2	58.35	2.224	49.22080869	1.130	n.a.

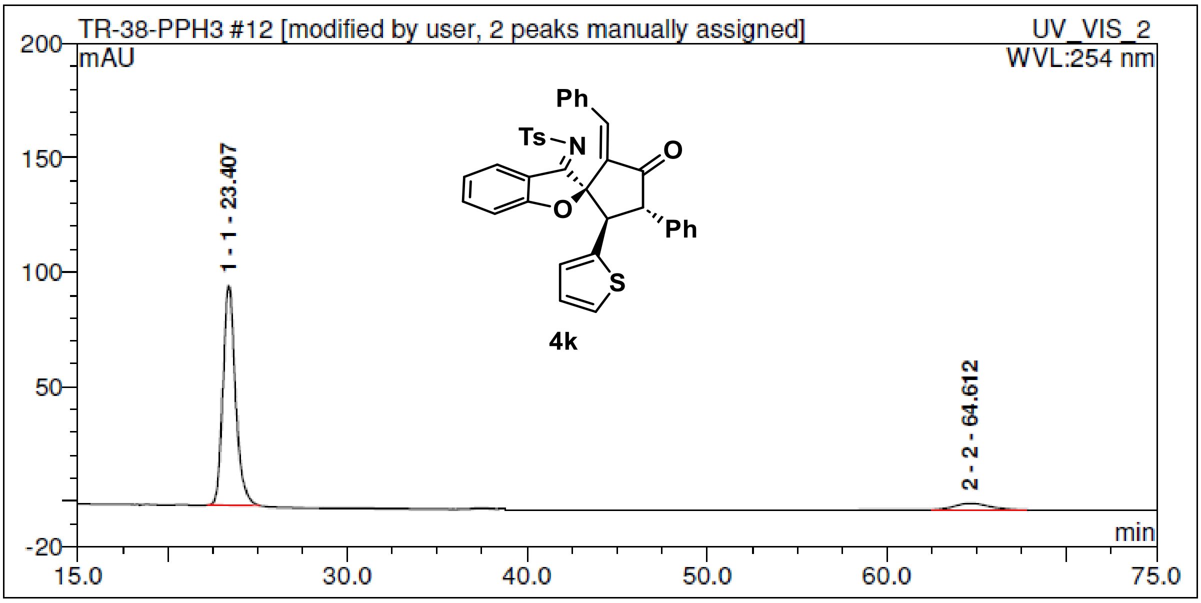

Peak Name		Ret.Time (detected) min	Area mAU*min	Rel.Area(ident.) %	Height mAU	Amount
1	1	23.41	78.23692	92.69827787	95.89997	n.a.
2	2	64.61	6.163	7.30172213	2.889	n.a.

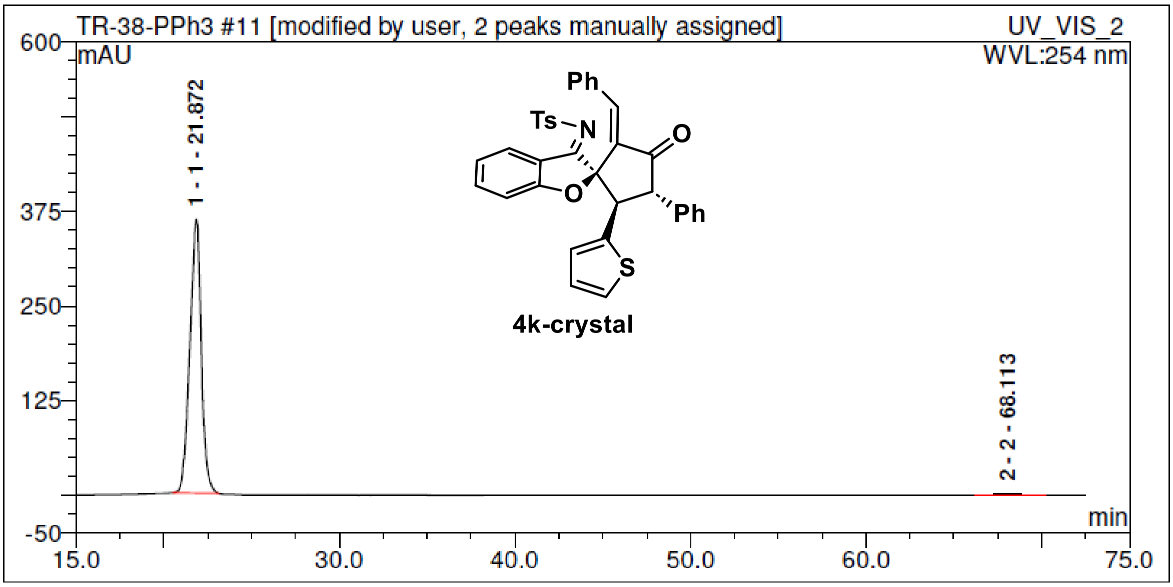

	Peak Name	Ret.Time (detected) min	Area mAU*min	Rel.Area(ident.) %	Height mAU	Amount
1	1	21.87	273.2586	99.16145018	361.7164	n.a.
2	2	68.11	2.311	0.8385498233	1.067	n.a.

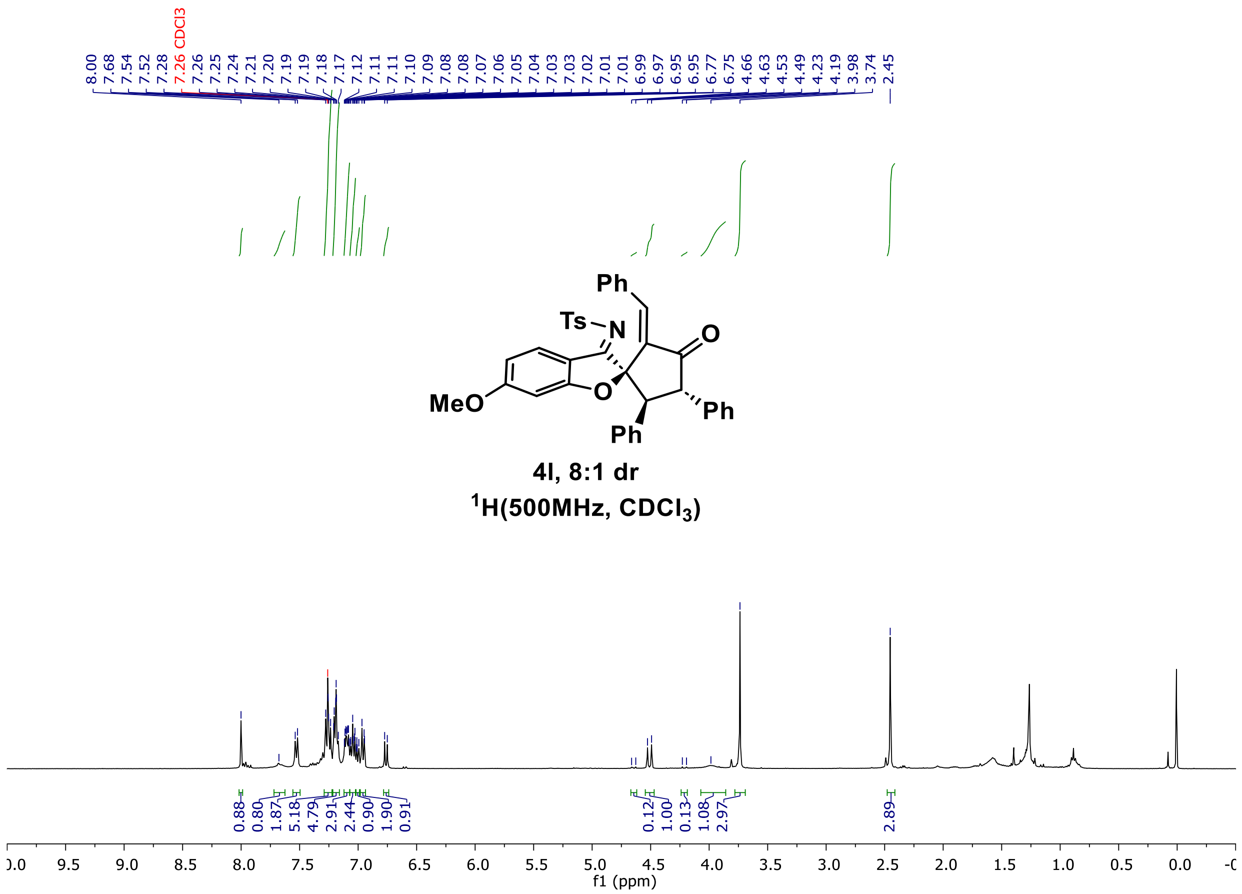
8.00 7.68 7.54 7.52 7.28 7.26 CDCl3 7.26 7.25 7.24 7.21 7.20 7.19 7.19 7.18 7.17 7.12 7.11 7.11 7.10 7.09 7.08 7.08 7.07 7.06 7.05 7.04 7.03 7.03 7.02 7.01 7.01 6.99 6.97 6.95 6.95 6.77 6.75 4.66 4.63 4.53 4.49 4.23 4.19 3.98 3.74 2.45
Ph
Ts
N
O
MeO
O
Ph
Ph
4l, 8:1 dr
1H(500MHz, CDCl3)
0.88 0.80 1.87 5.18 4.79 2.91 2.44 0.90 1.90 0.91 0.12 1.00 0.13 1.08 2.97 2.89
f1 (ppm)

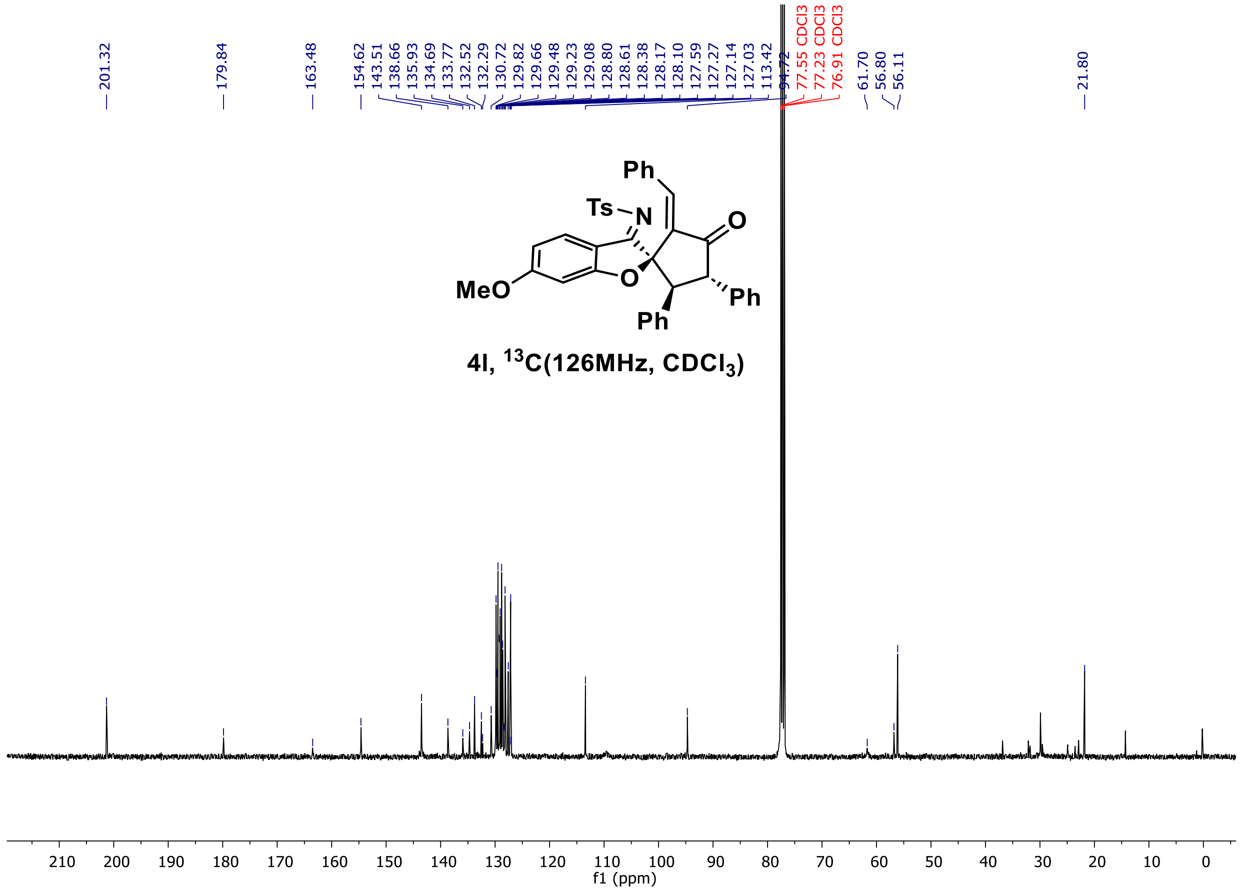
201.32 179.84 163.48 154.62 143.51 138.66 135.93 134.69 133.77 132.52 132.29 130.72 129.82 129.66 129.48 129.23 129.08 128.80 128.61 128.38 128.17 128.10 127.59 127.27 127.14 127.03 113.42 94.72 77.55 CDCl3 77.23 CDCl3 76.91 CDCl3 61.70 56.80 56.11 21.80
Ph
Ts
N
O
MeO
O
Ph
Ph
4l, 13C(126MHz, CDCl3)
f1 (ppm)

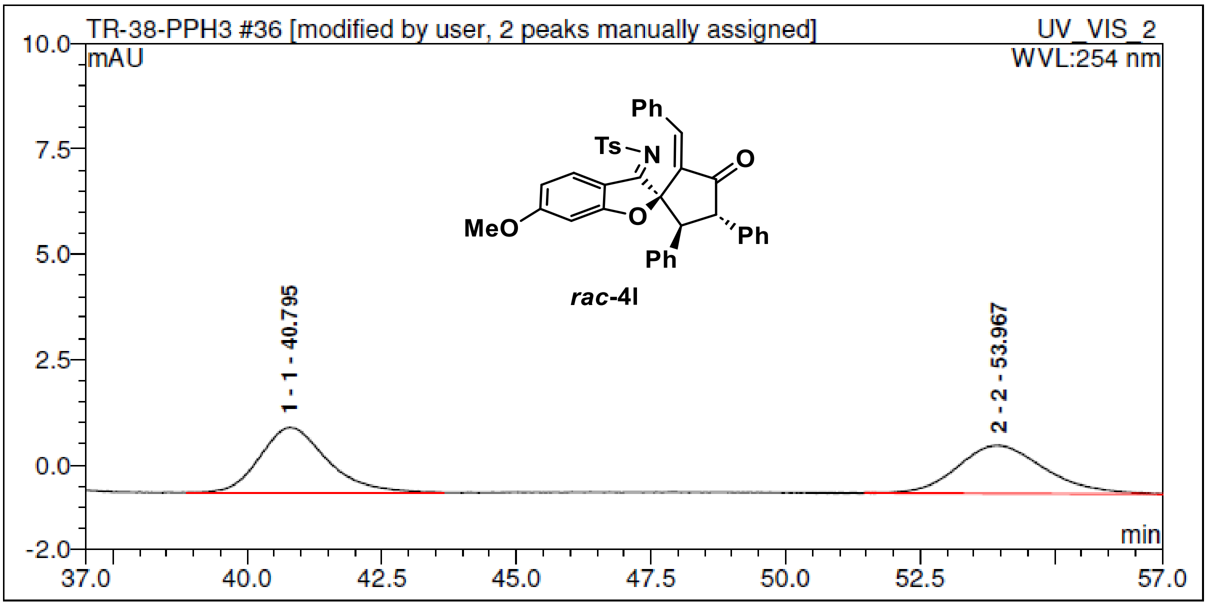

Peak	Name	Ret.Time (detected) min	Area mAU*min	Rel.Area(ident.) %	Height mAU	Amount
1	1	40.80	2.219567	50.72849322	1.53693	n.a.
2	2	53.97	2.156	49.27150678	1.143	n.a.

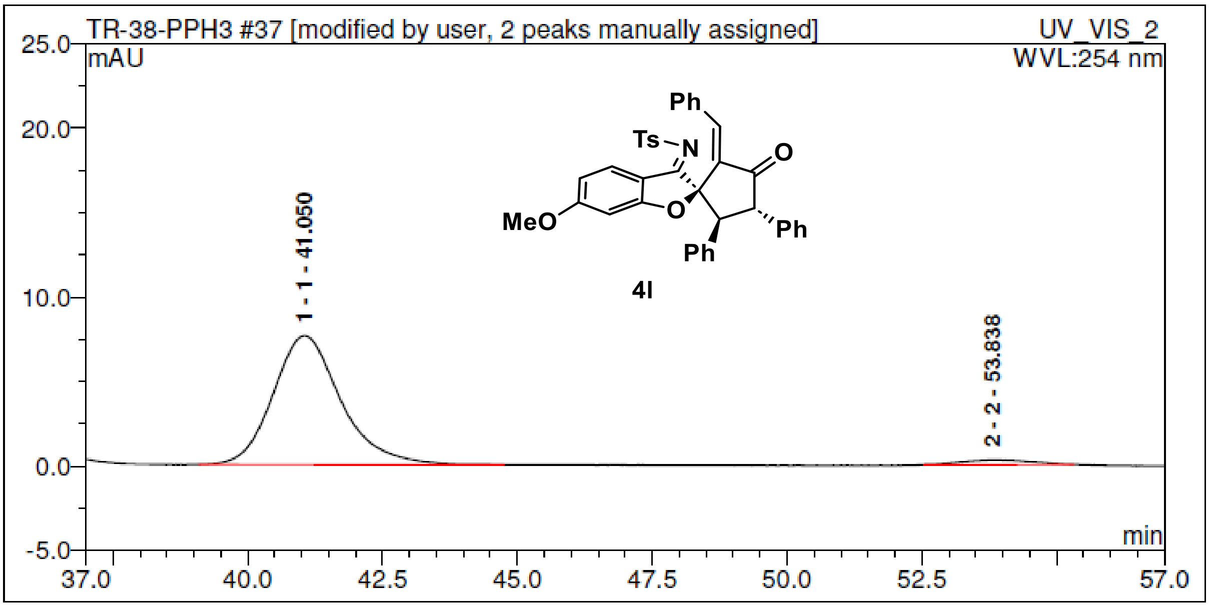

Peak	Name	Ret.Time (detected) min	Area mAU*min	Rel.Area(ident.) %	Height mAU	Amount
1	1	41.05	11.10198	96.400452	7.61653	n.a.
2	2	53.84	0.415	3.599548	0.274	n.a.

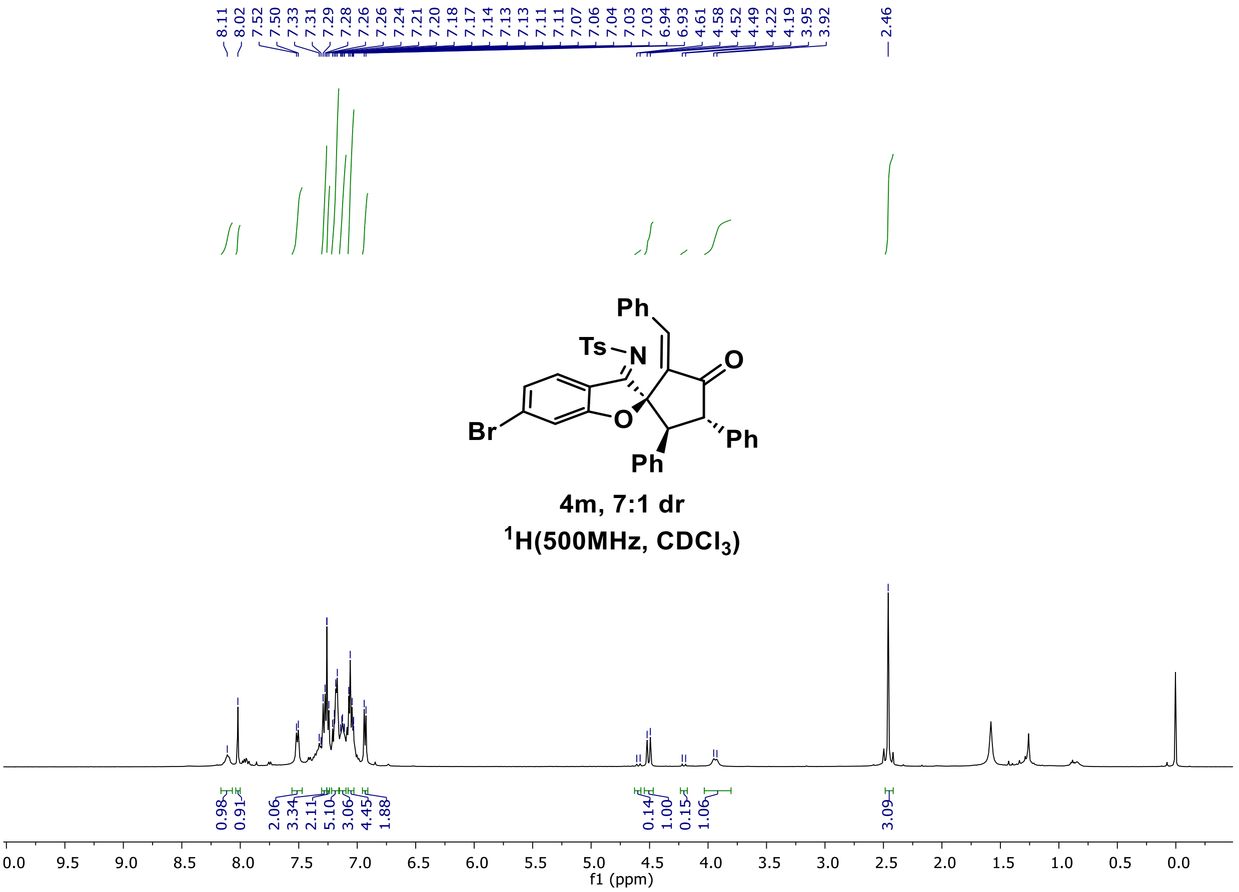
4m, 7:1 dr
1H(500MHz, CDCl3)
f1 (ppm)

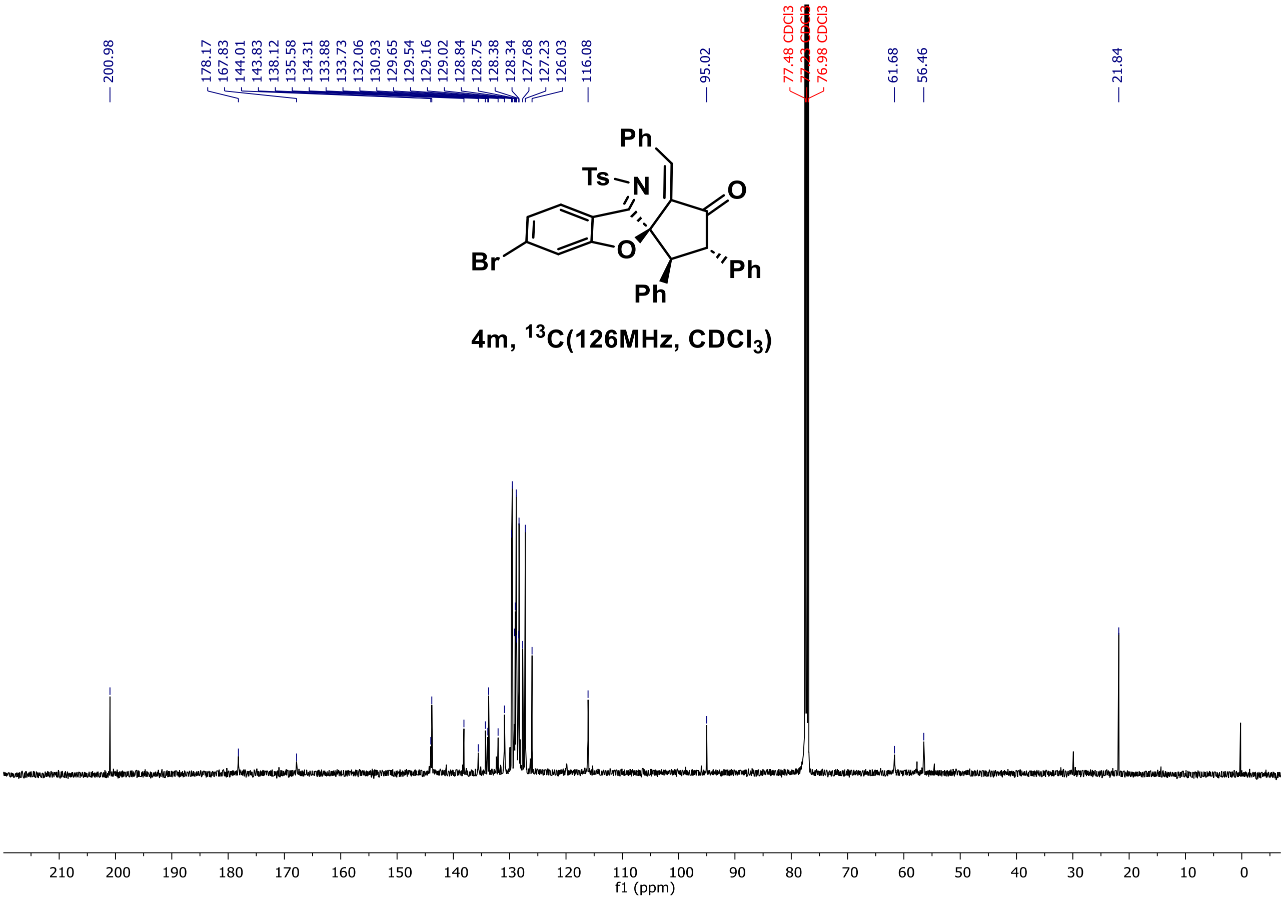
4m, 13C(126MHz, CDCl3)
f1 (ppm)

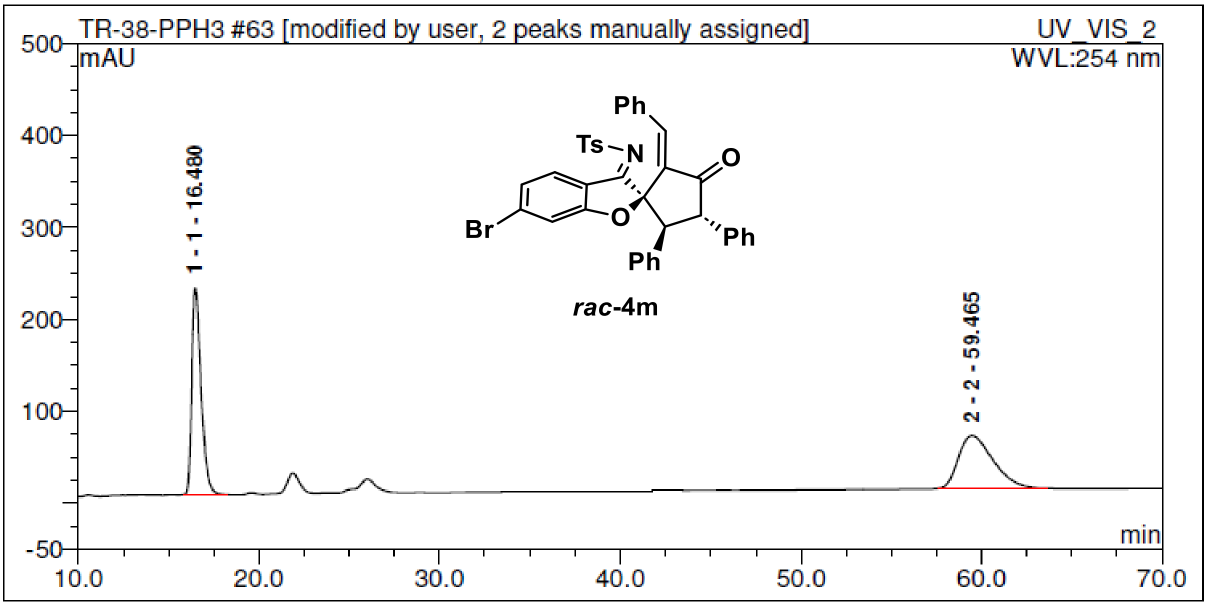

Peak Name		Ret.Time (detected) min	Area mAU*min	Rel.Area(ident.) %	Height mAU	Amount
1	1	16.48	134.8425	51.57120796	224.5716	n.a.
2	2	59.47	126.626	48.42879204	57.231	n.a.

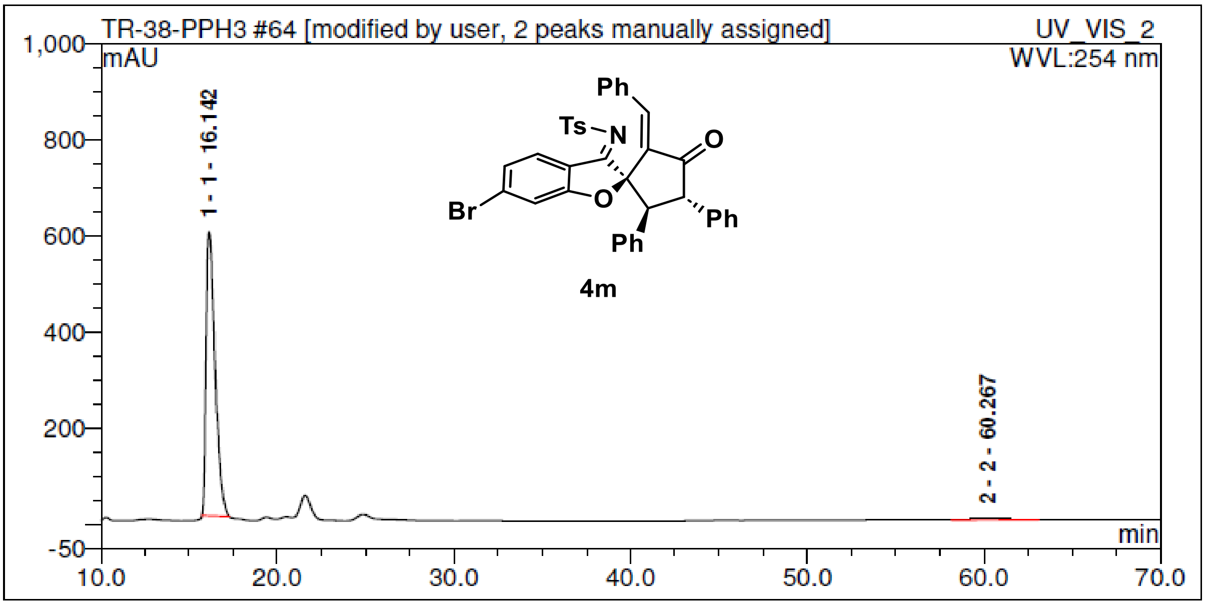

Peak Name		Ret.Time (detected) min	Area mAU*min	Rel.Area(ident.) %	Height mAU	Amount
1	1	16.14	345.9093	96.8343323	590.0226	n.a.
2	2	60.27	11.308	3.165667704	5.413	n.a.

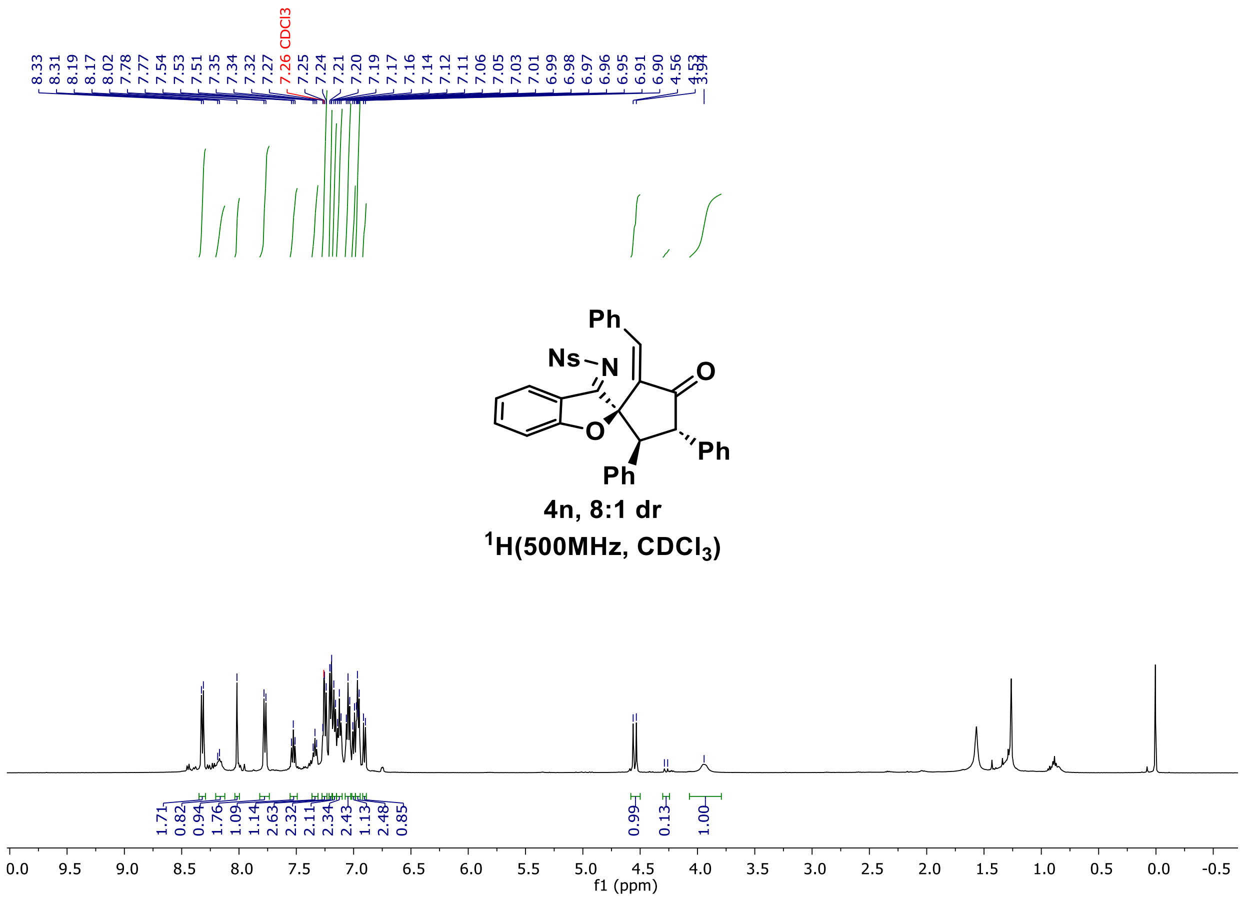
Ph
Ns
N
O
O
Ph
Ph
4n, 8:1 dr
^{1}H(500MHz, CDCl$_3$)
f1 (ppm)

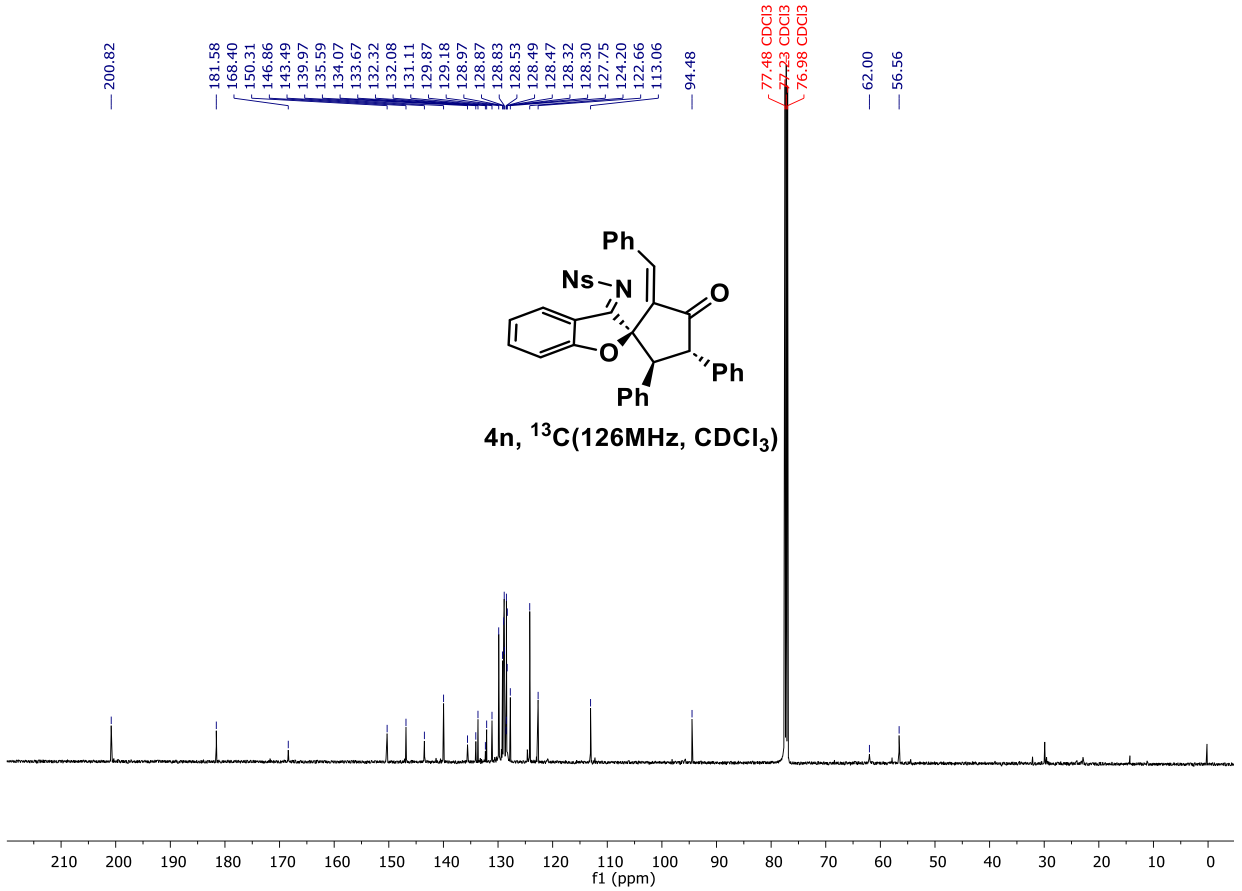
Ph
Ns
N
O
O
Ph
Ph
4n, ^{13}C(126MHz, CDCl$_3$)
f1 (ppm)

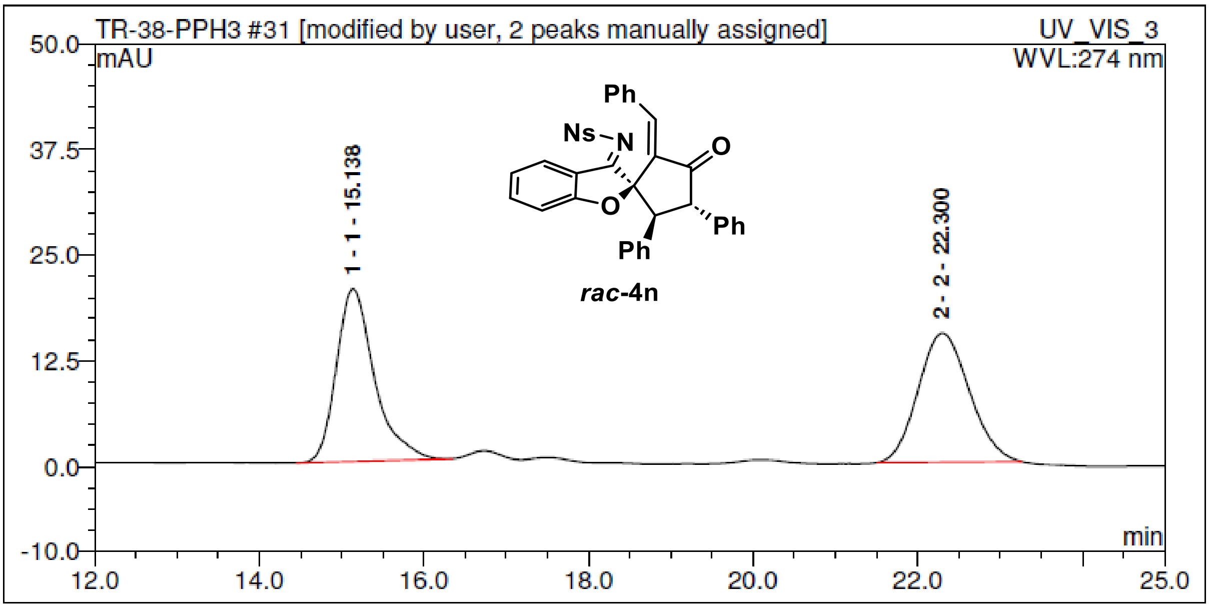

Peak Name		Ret.Time (detected) min	Area mAU*min	Rel.Area(ident.) %	Height mAU	Amount
1	1	15.14	10.93143	49.91928757	20.40543	n.a.
2	2	22.30	10.967	50.08071243	15.247	n.a.

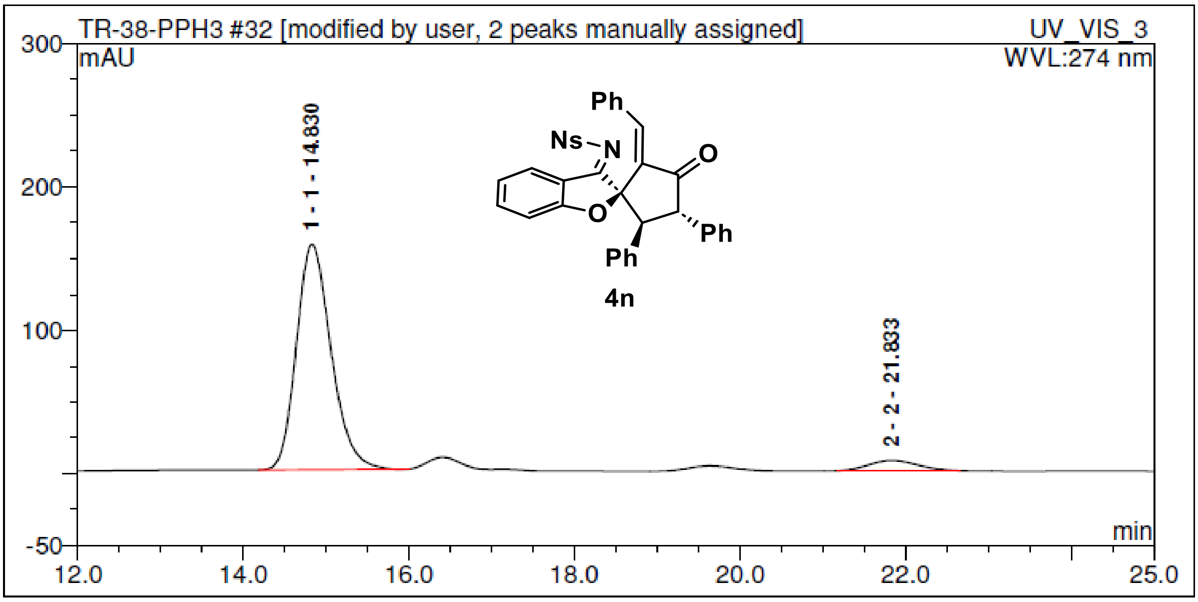

Peak Name		Ret.Time (detected) min	Area mAU*min	Rel.Area(ident.) %	Height mAU	Amount
1	1	14.83	76.1934	93.96735604	156.8146	n.a.
2	2	21.83	4.892	6.03264396	7.175	n.a.

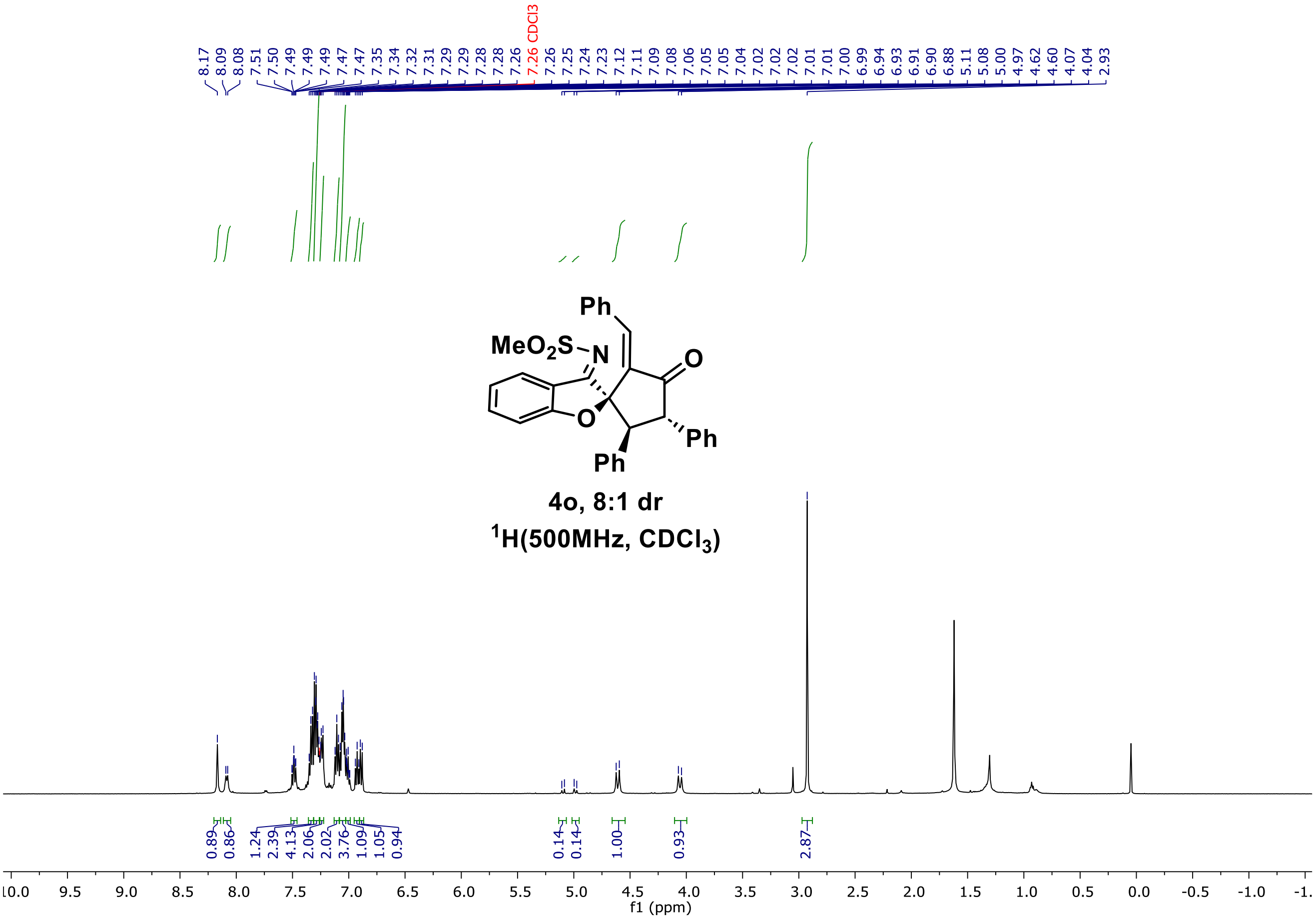
4o, 8:1 dr
^{1}H(500MHz, CDCl$_3$)
f1 (ppm)

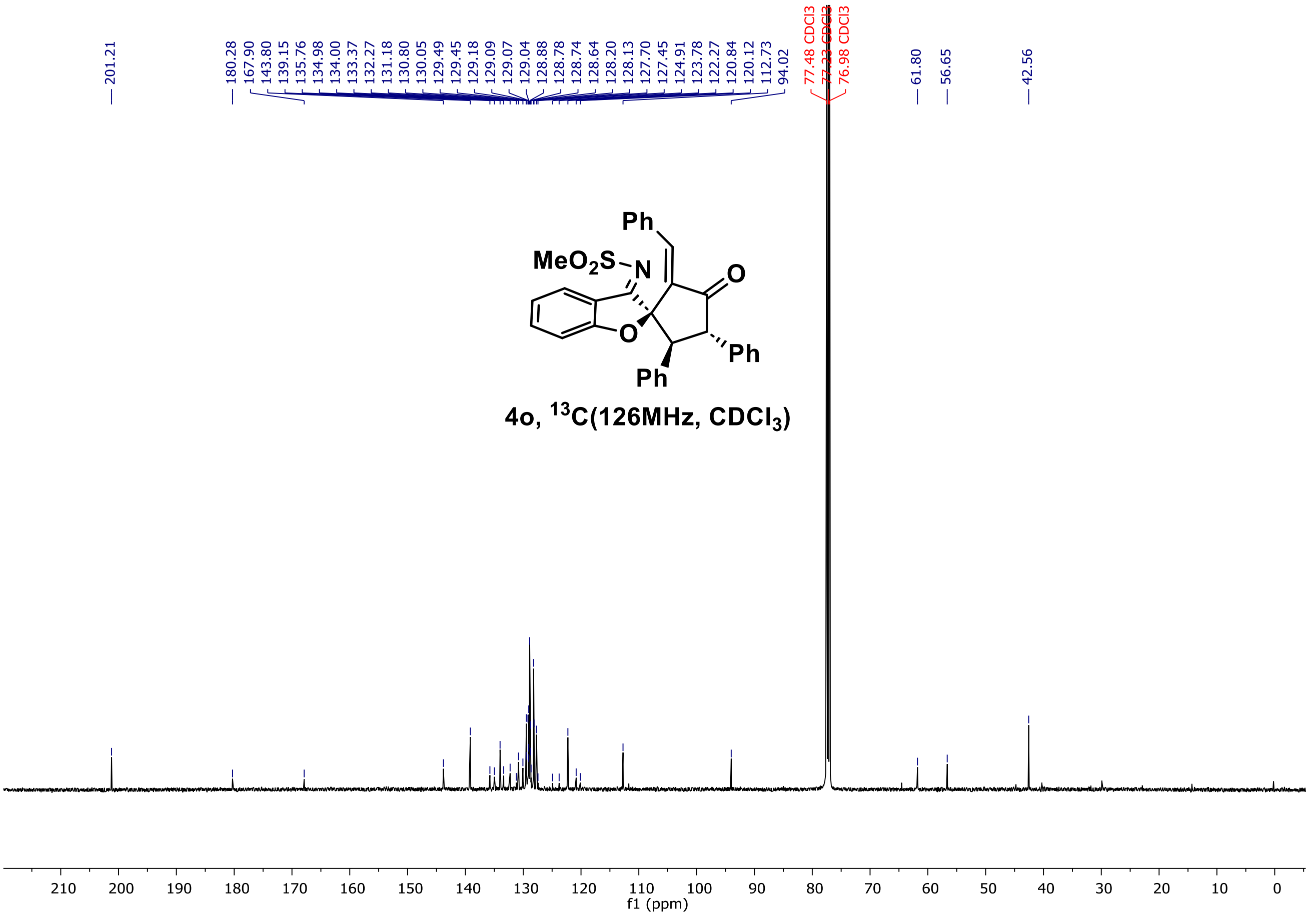
4o, ^{13}C(126MHz, CDCl$_3$)
f1 (ppm)

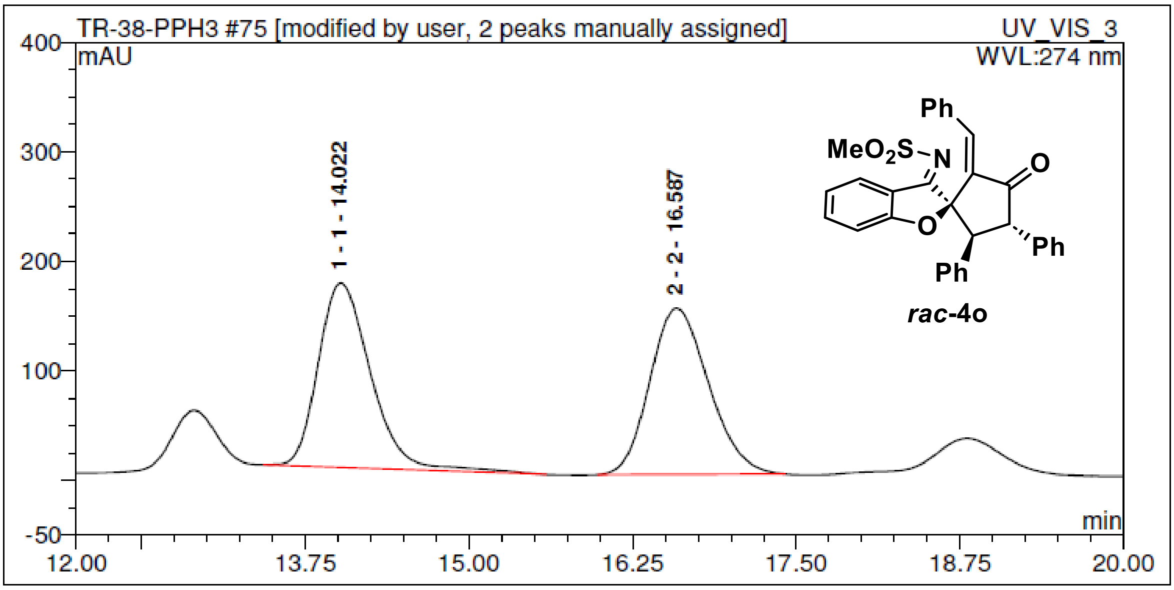

Peak	Name	Ret.Time (detected) min	Area mAU*min	Rel.Area(ident.) %	Height mAU	Amount
1	1	14.02	78.53934	49.83800986	168.4198	n.a.
2	2	16.59	79.050	50.16199014	151.701	n.a.

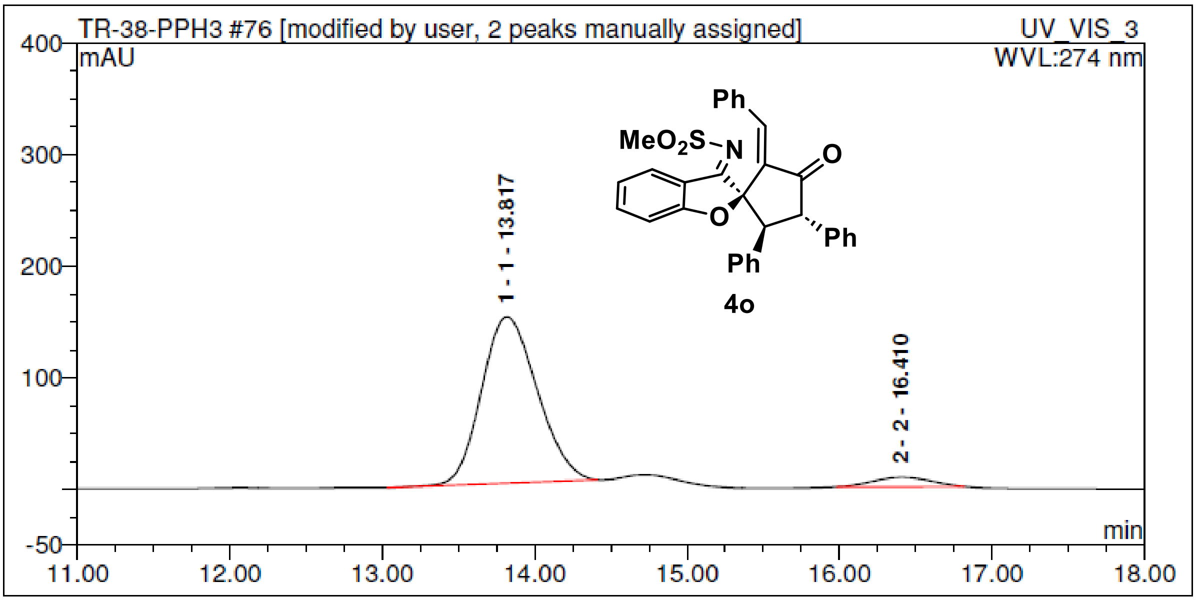

Peak	Name	Ret.Time (detected) min	Area mAU*min	Rel.Area(ident.) %	Height mAU	Amount
1	1	13.82	63.27539	94.51817587	149.2236	n.a.
2	2	16.41	3.670	5.481824126	8.462	n.a.

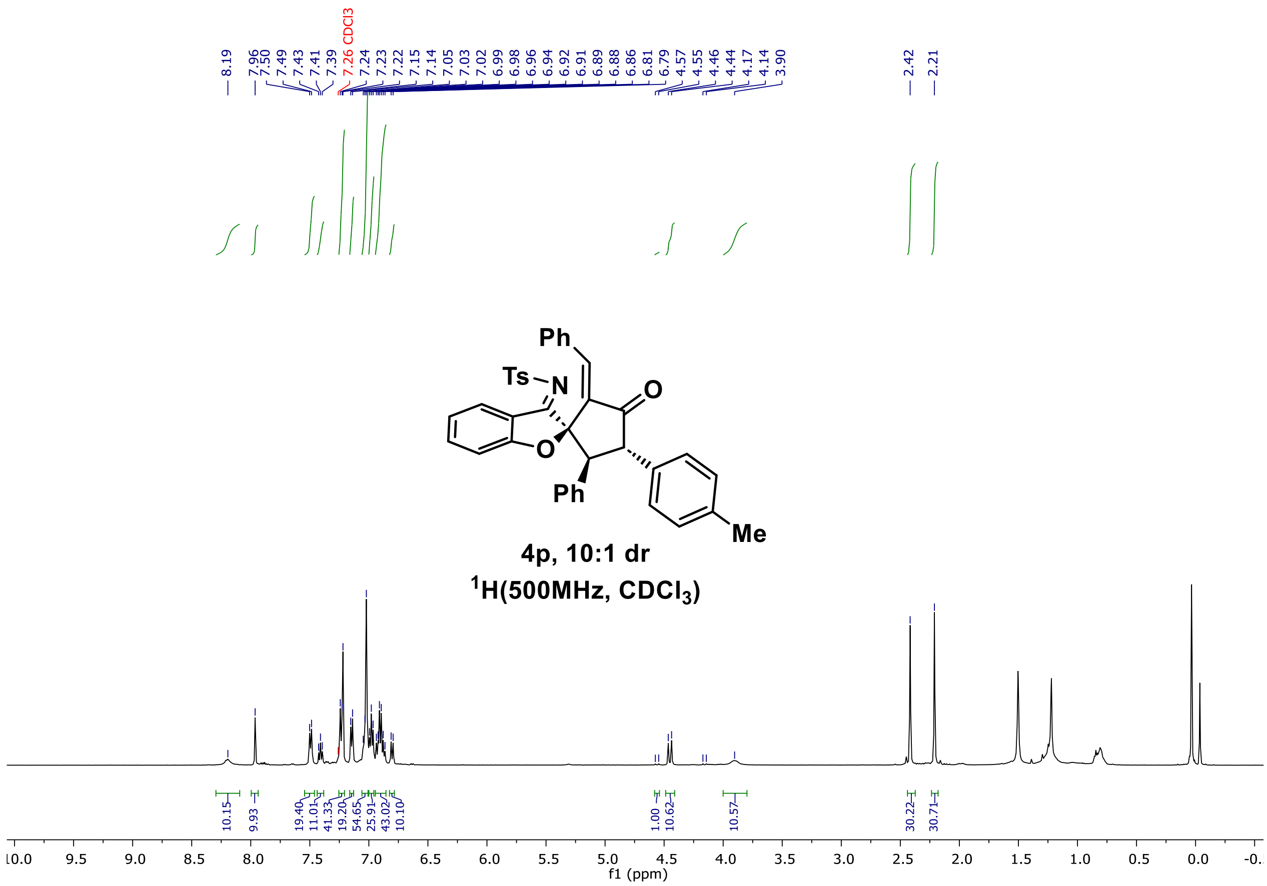
8.19
7.96
7.50
7.49
7.43
7.41
7.39
7.26 CDCl3
7.24
7.23
7.22
7.15
7.14
7.05
7.03
7.02
6.99
6.98
6.96
6.94
6.92
6.91
6.89
6.88
6.86
6.81
6.79
4.57
4.55
4.46
4.44
4.17
4.14
3.90
2.42
2.21
Ph
Ts
N
O
O
Ph
Me
4p, 10:1 dr
^{1}H(500MHz, CDCl$_3$)
10.15
9.93
19.40
11.01
41.33
19.20
54.65
25.91
43.02
10.10
1.00
10.62
10.57
30.22
30.71
f1 (ppm)

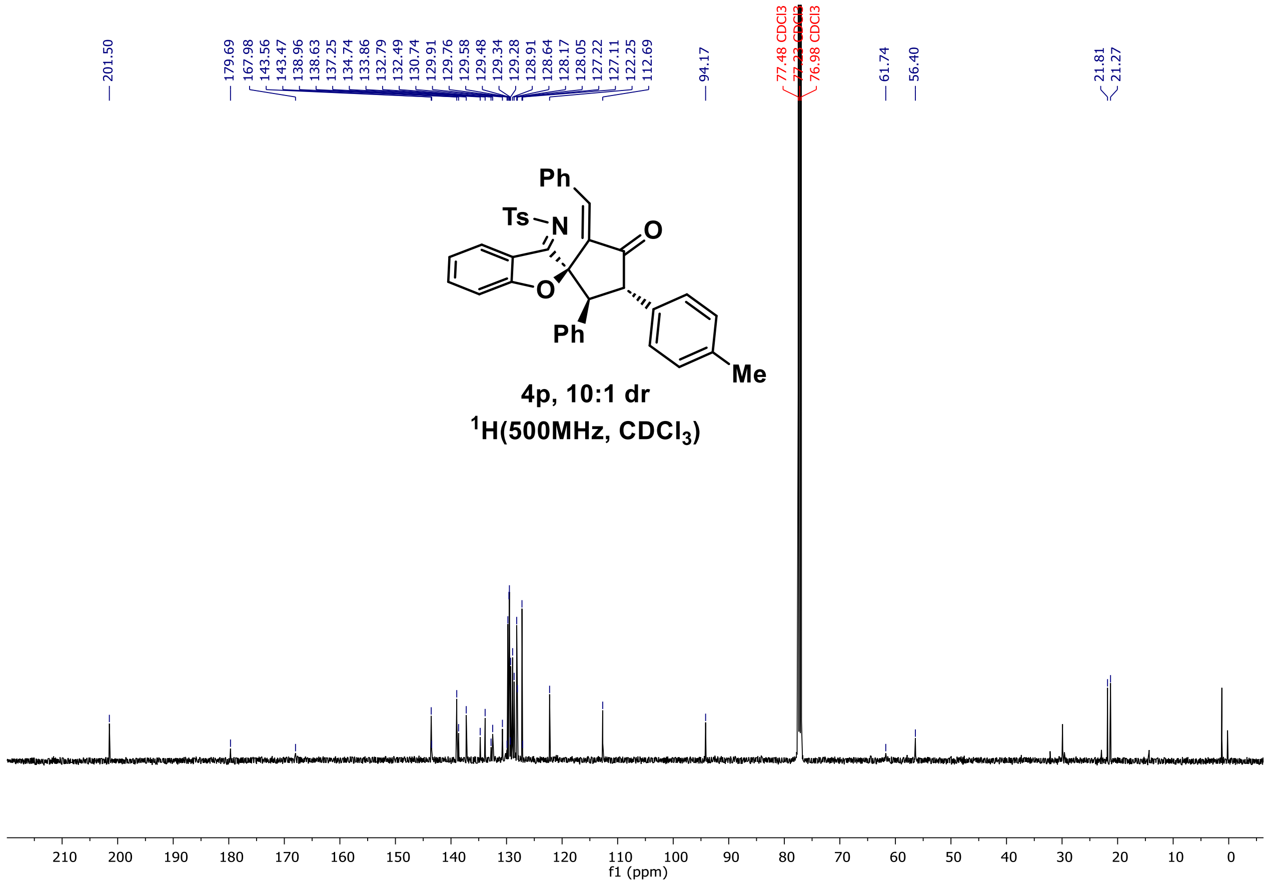
201.50
179.69
167.98
143.56
143.47
138.96
138.63
137.25
134.74
133.86
132.79
132.49
130.74
129.91
129.76
129.58
129.48
129.34
129.28
128.91
128.64
128.17
128.05
127.22
127.11
122.25
112.69
94.17
77.48 CDCl3
76.98 CDCl3
61.74
56.40
21.81
21.27
Ph
Ts
N
O
O
Ph
Me
4p, 10:1 dr
^{1}H(500MHz, CDCl$_3$)
f1 (ppm)

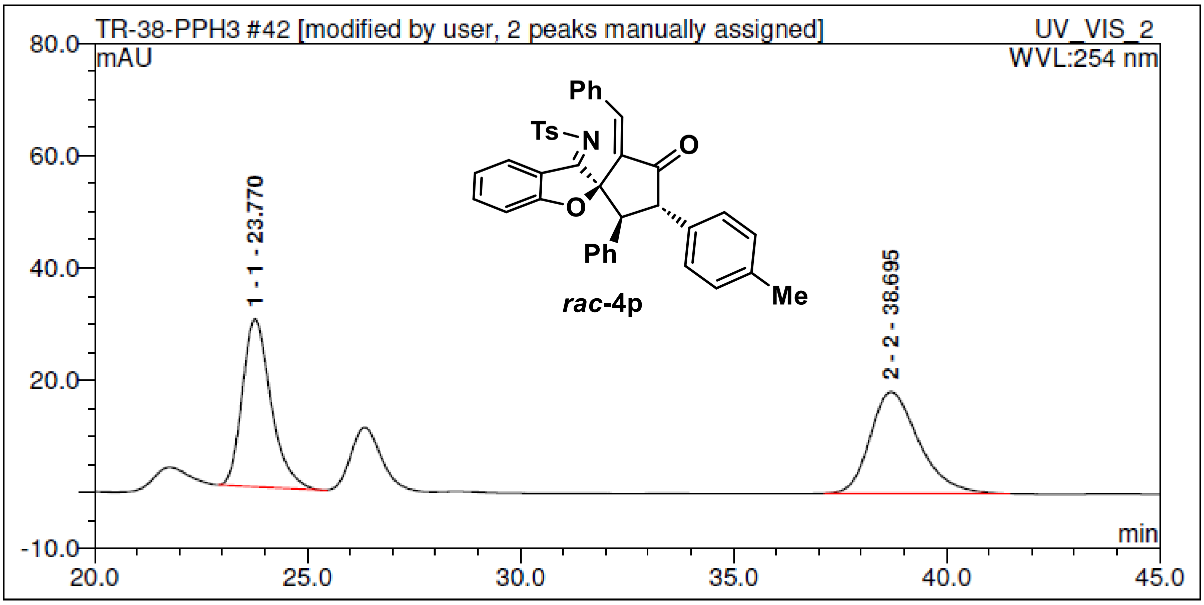

Peak	Name	Ret.Time (detected) min	Area mAU*min	Rel.Area(ident.) %	Height mAU	Amount
1	1	23.77	23.32942	49.2986353	29.89354	n.a.
2	2	38.70	23.993	50.7013647	18.093	n.a.

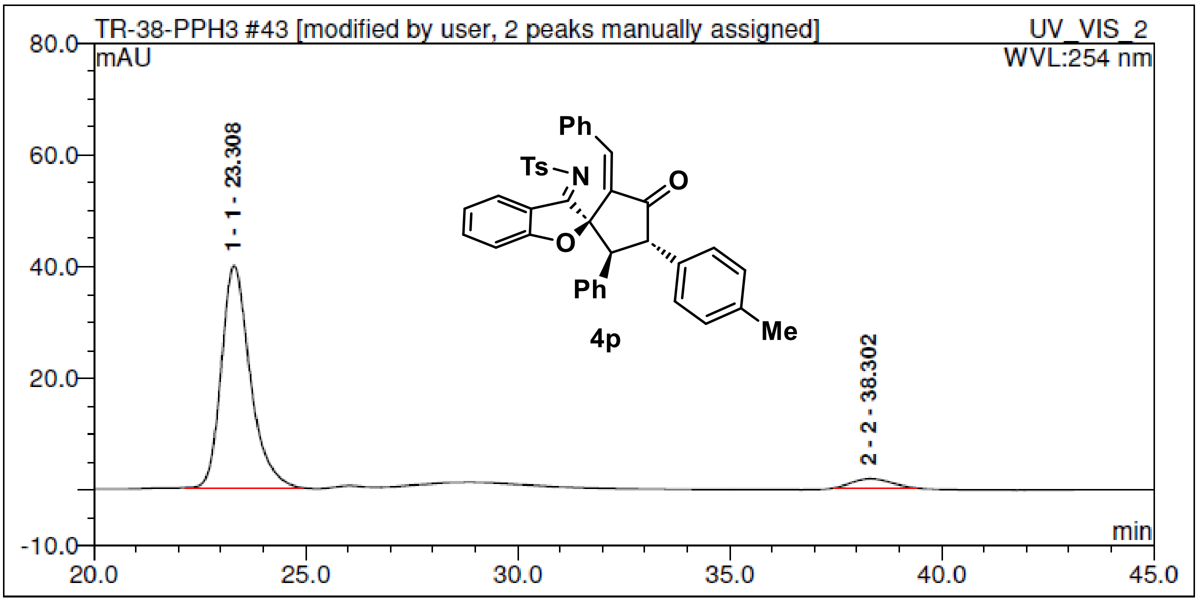

Peak	Name	Ret.Time (detected) min	Area mAU*min	Rel.Area(ident.) %	Height mAU	Amount
1	1	23.31	31.24895	94.80346206	39.75179	n.a.
2	2	38.30	1.713	5.196537937	1.662	n.a.

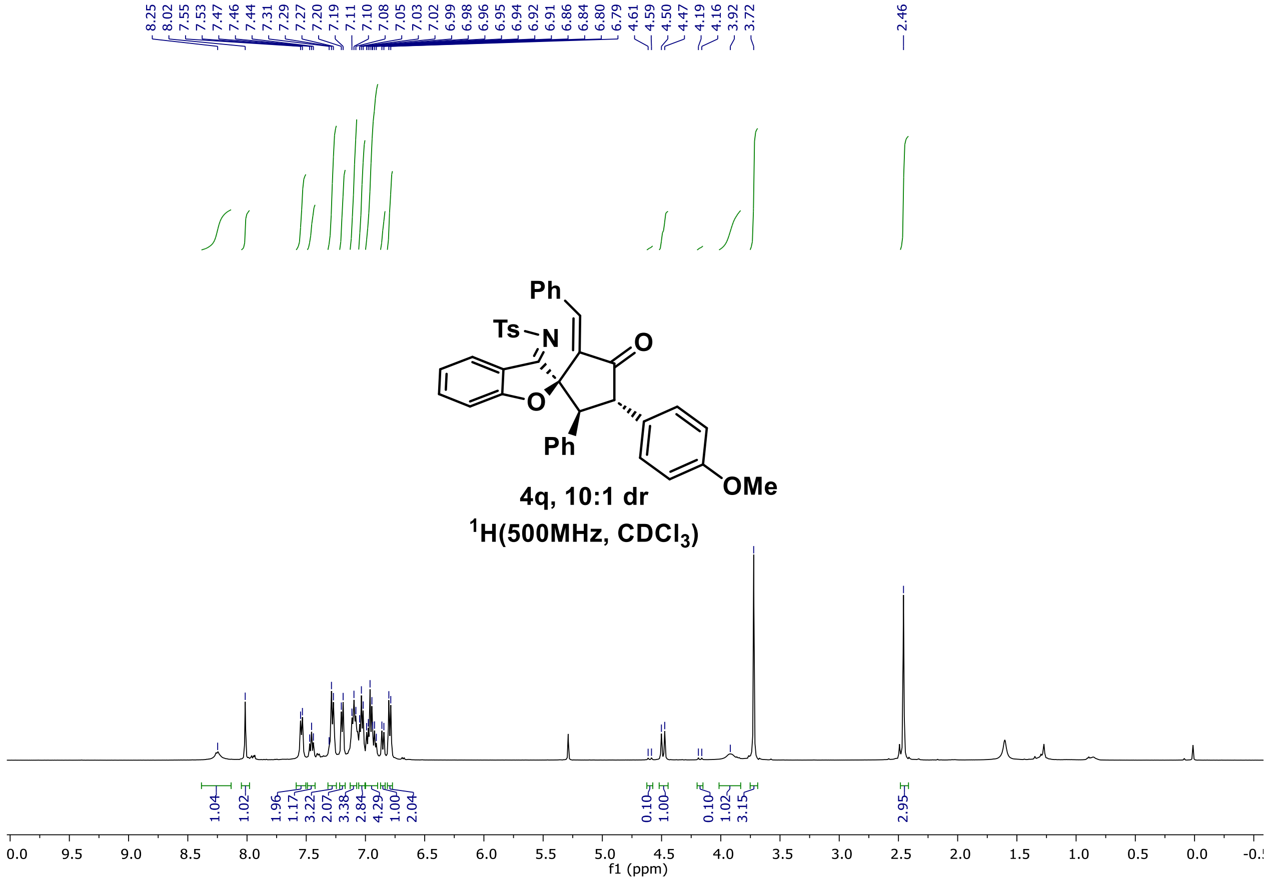
Ph
Ts
N
O
O
Ph
OMe
4q, 10:1 dr
^1H(500MHz, $CDCl_3$)
f1 (ppm)

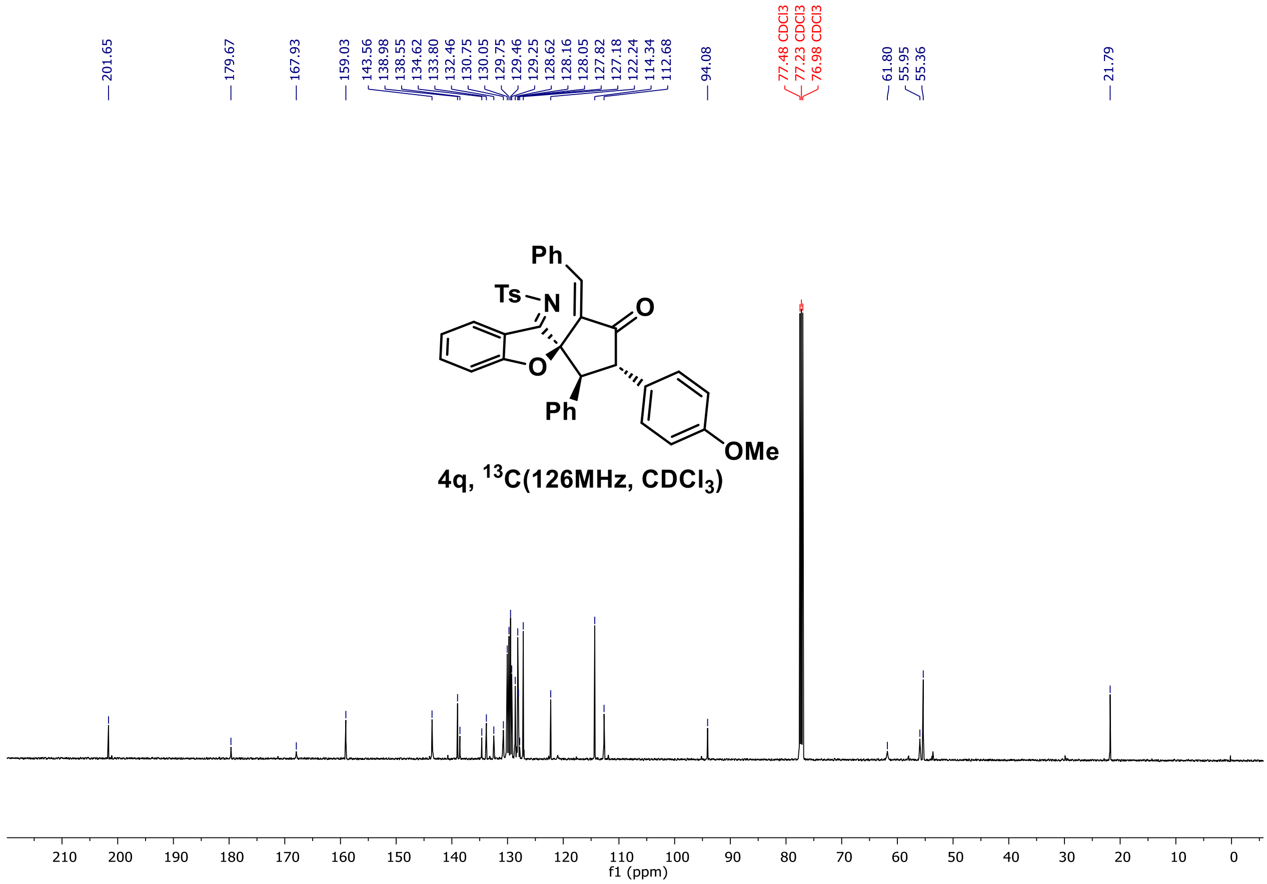
77.48 CDCl3
77.23 CDCl3
76.98 CDCl3
Ph
Ts
N
O
O
Ph
OMe
4q, ^{13}C(126MHz, $CDCl_3$)
f1 (ppm)

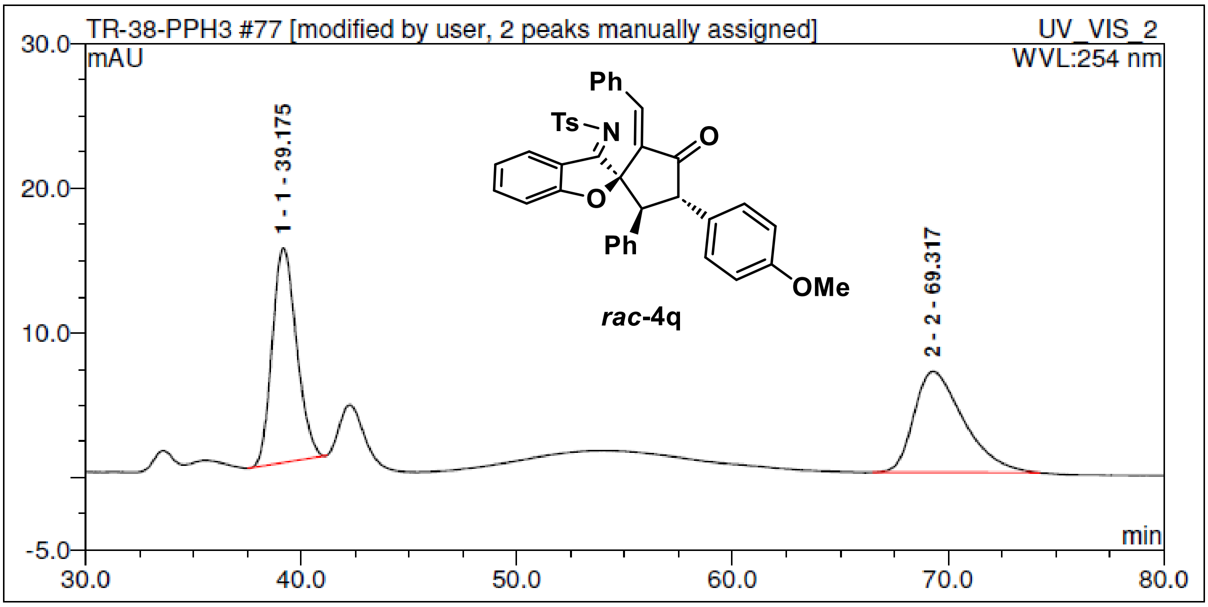

Peak	Name	Ret.Time (detected) min	Area mAU*min	Rel.Area(ident.) %	Height mAU	Amount
1	1	39.18	19.40968	50.96546048	14.83563	n.a.
2	2	69.32	18.674	49.03453952	6.975	n.a.

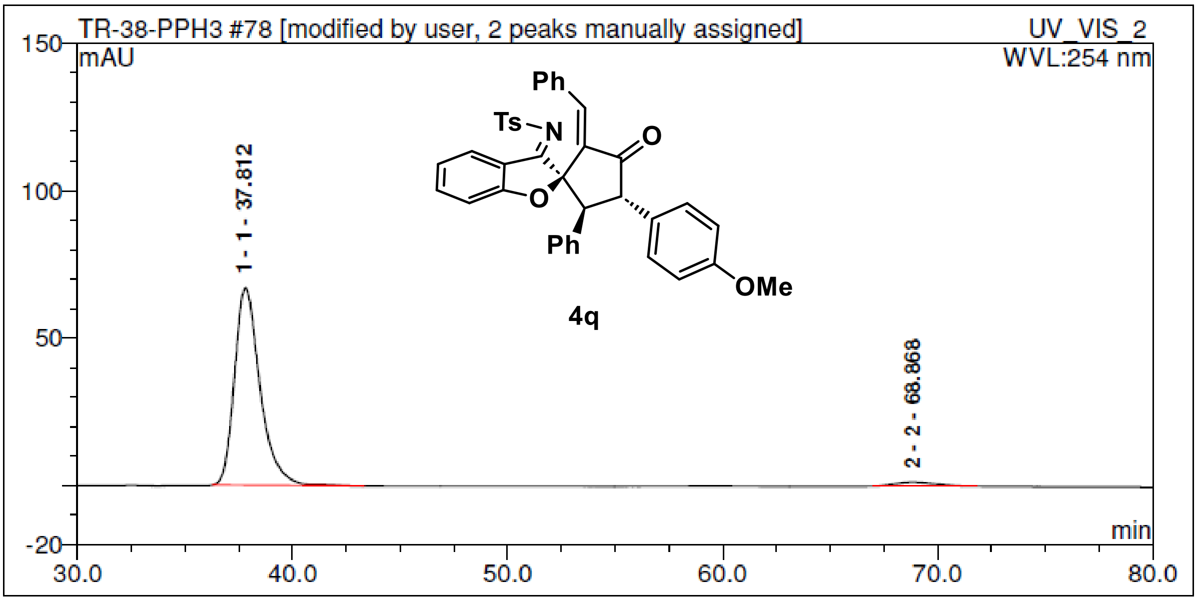

Peak	Name	Ret.Time (detected) min	Area mAU*min	Rel.Area(ident.) %	Height mAU	Amount
1	1	37.81	92.52033	96.77391916	66.83202	n.a.
2	2	68.87	3.084	3.226080837	1.342	n.a.

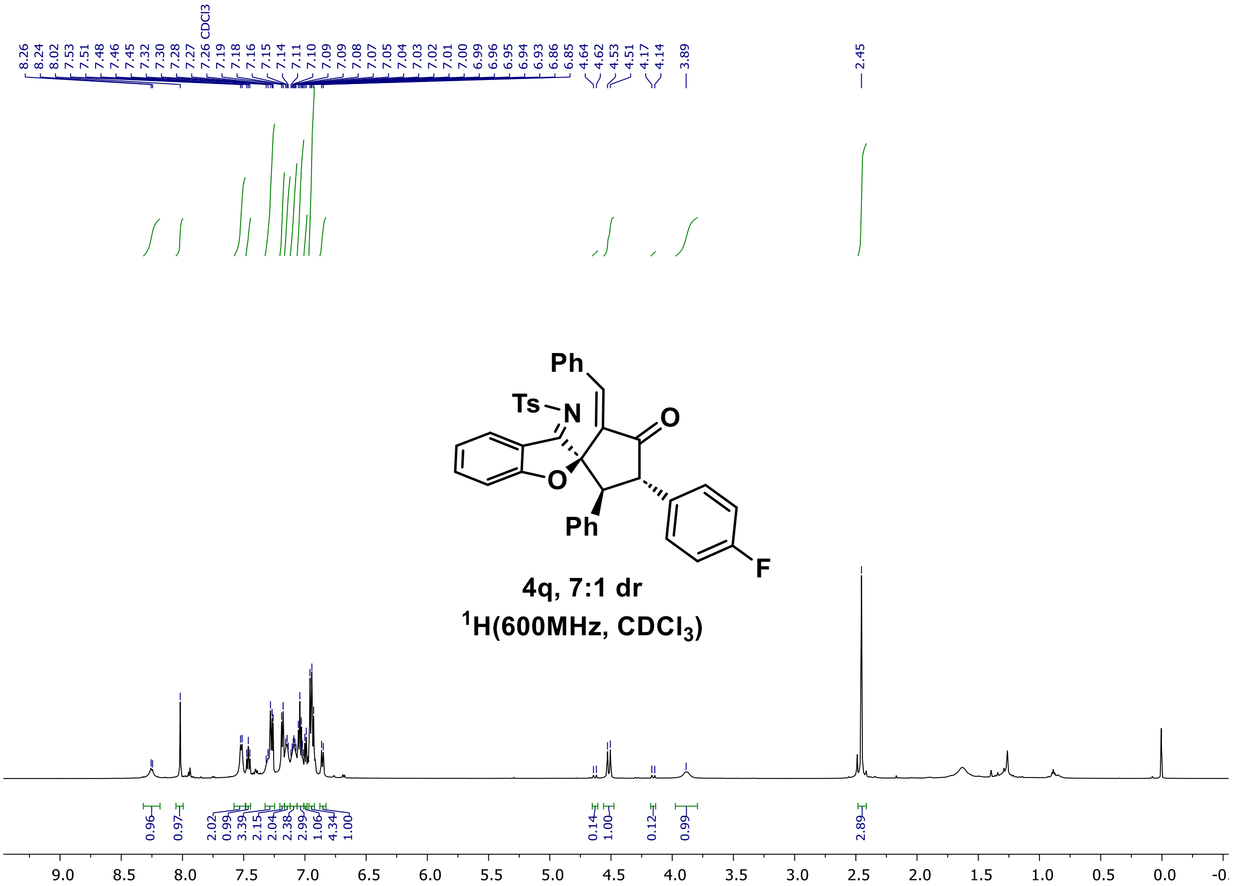
8.26 8.24 8.02 7.53 7.51 7.48 7.46 7.45 7.32 7.30 7.28 7.27 7.26 CDCl3 7.19 7.18 7.16 7.15 7.14 7.11 7.10 7.09 7.08 7.07 7.05 7.04 7.03 7.02 7.01 7.00 6.99 6.96 6.95 6.94 6.93 6.86 6.85 4.64 4.62 4.53 4.51 4.17 4.14 3.89
2.45
Ph
Ts
N
O
O
Ph
F
4q, 7:1 dr
^{1}H(600MHz, CDCl$_3$)
0.96 0.97 2.02 0.99 3.39 2.15 2.04 2.38 2.99 1.06 4.34 1.00
0.14 1.00 0.12 0.99 2.89
9.0 8.5 8.0 7.5 7.0 6.5 6.0 5.5 5.0 4.5 4.0 3.5 3.0 2.5 2.0 1.5 1.0 0.5 0.0

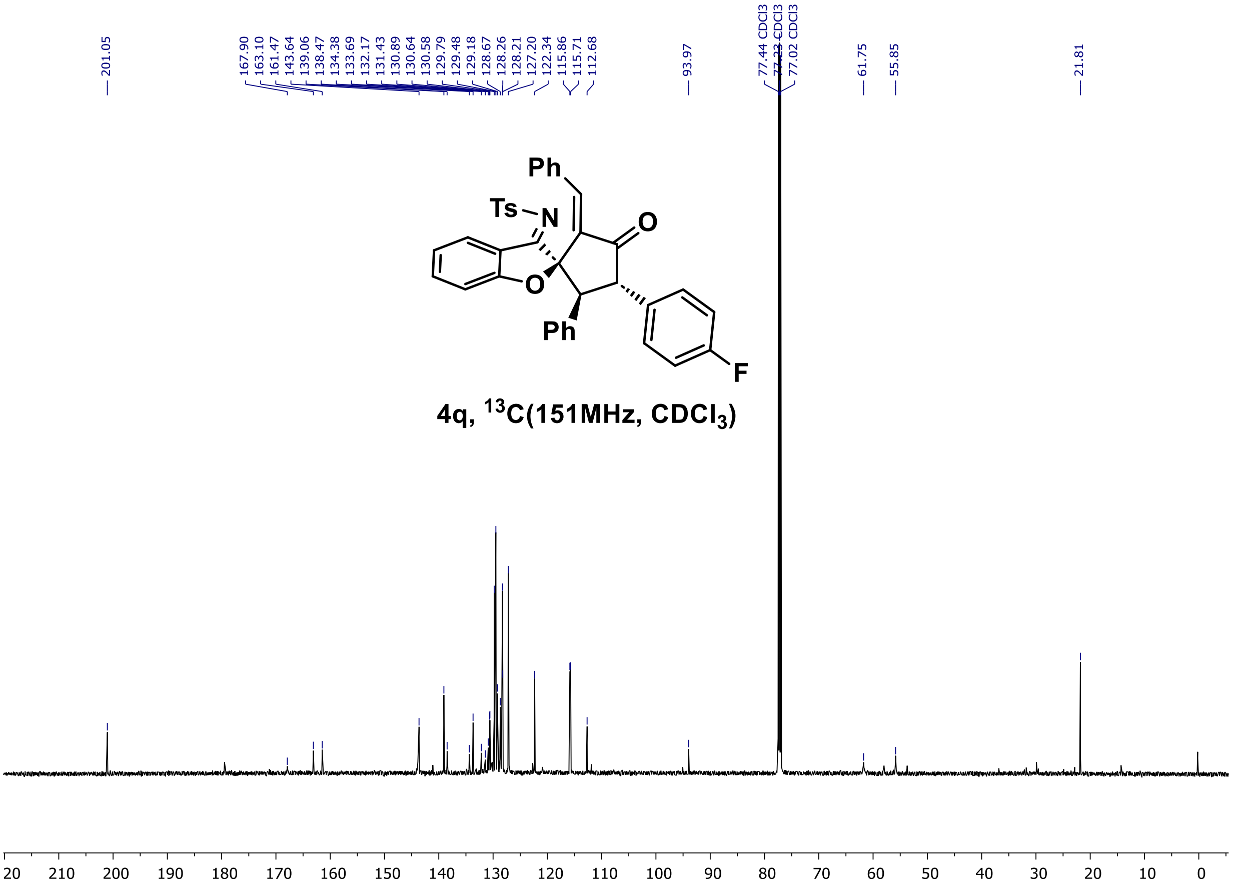
201.05
167.90 163.10 161.47 143.64 139.06 138.47 134.38 133.69 132.17 131.43 130.89 130.64 130.58 129.79 129.48 129.18 128.67 128.26 128.21 127.20 122.34 115.86 115.71 112.68
93.97
77.44 CDCl3 77.23 CDCl3 77.02 CDCl3
61.75
55.85
21.81
Ph
Ts
N
O
O
Ph
F
4q, ^{13}C(151MHz, CDCl$_3$)
210 200 190 180 170 160 150 140 130 120 110 100 90 80 70 60 50 40 30 20 10 0

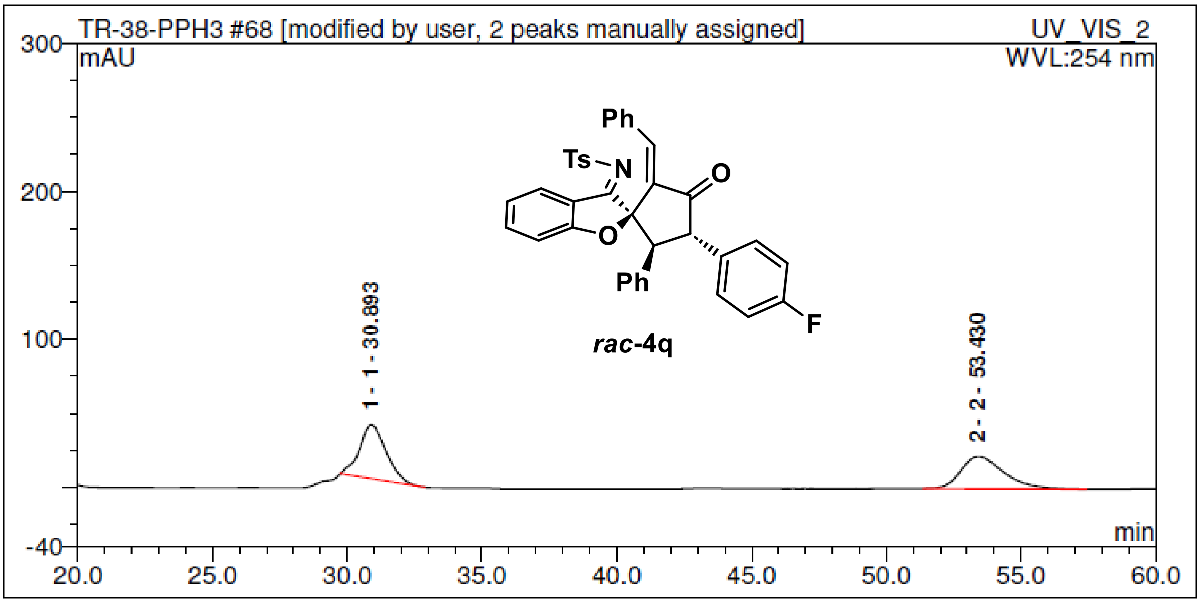

	Peak Name	Ret.Time (detected) min	Area mAU*min	Rel.Area(ident.) %	Height mAU	Amount
1	1	30.89	41.67435	51.09812449	36.24735	n.a.
2	2	53.43	39.883	48.90187551	21.696	n.a.

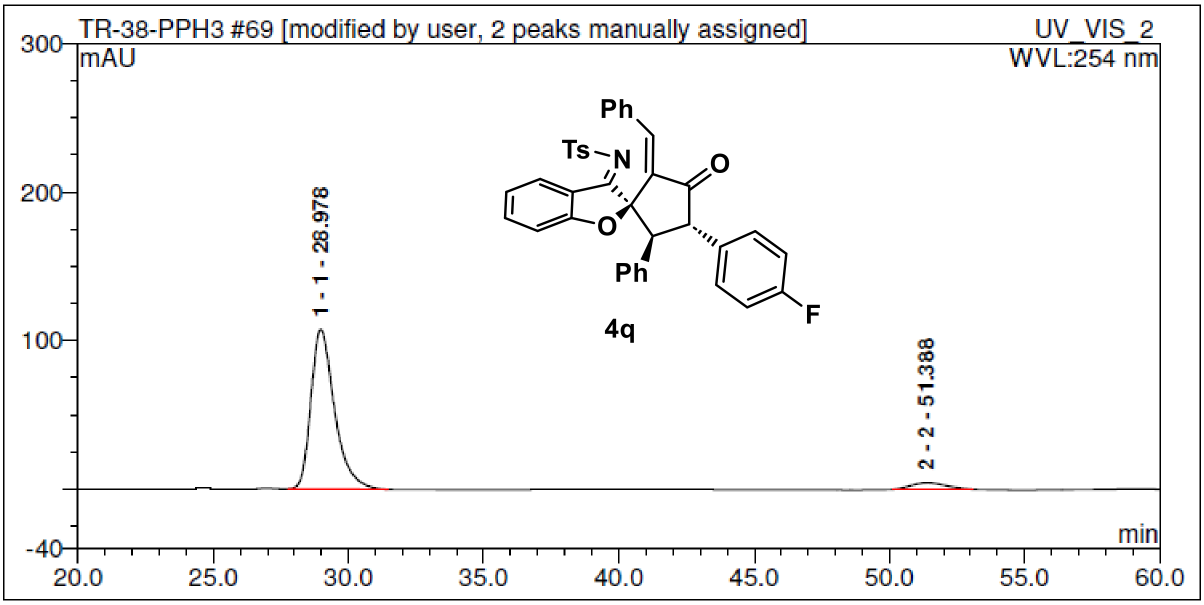

	Peak Name	Ret.Time (detected) min	Area mAU*min	Rel.Area(ident.) %	Height mAU	Amount
1	1	28.98	108.8931	94.4147708	107.2221	n.a.
2	2	51.39	6.442	5.585229195	4.291	n.a.

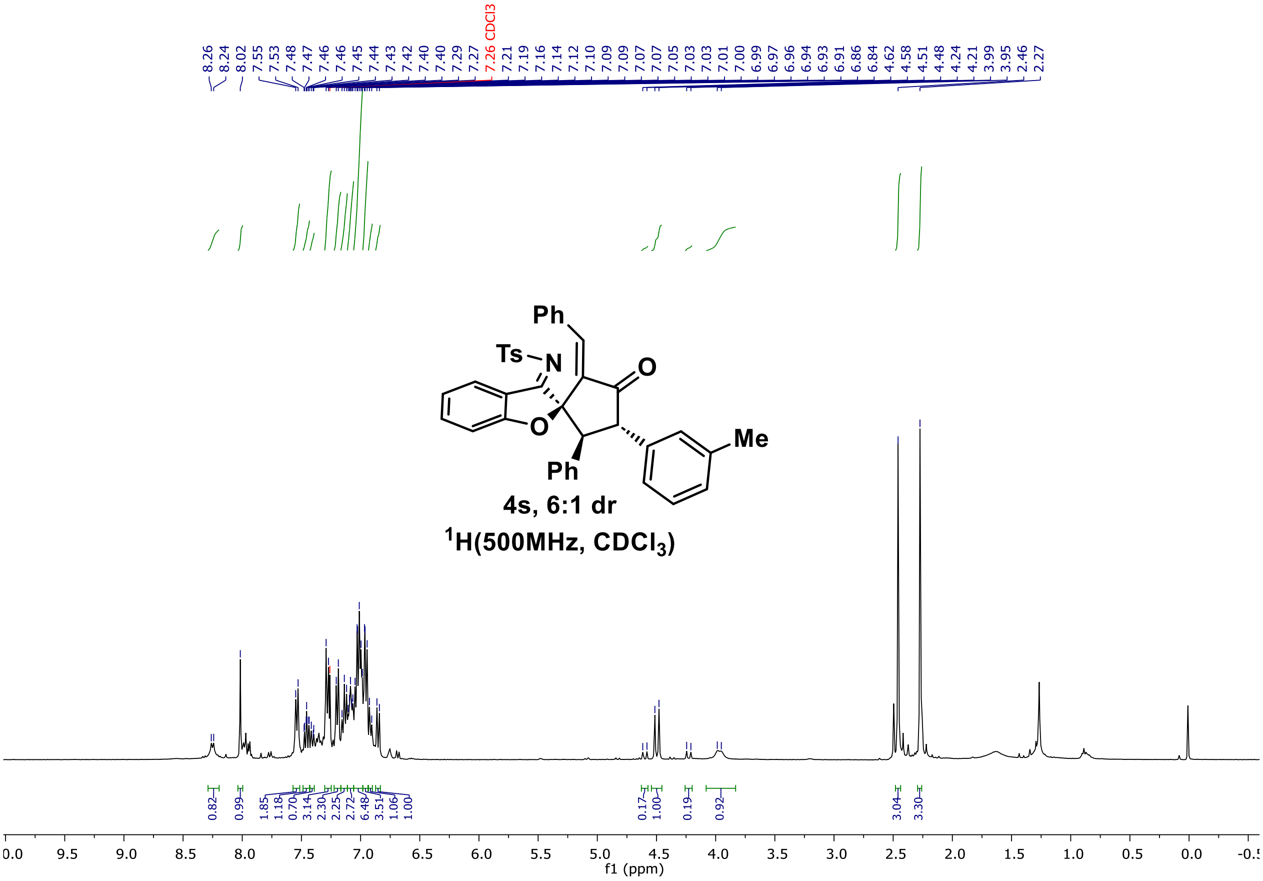
Ph
Ts
N
O
O
Ph
Me
4s, 6:1 dr
^{1}H(500MHz, CDCl$_3$)
f1 (ppm)

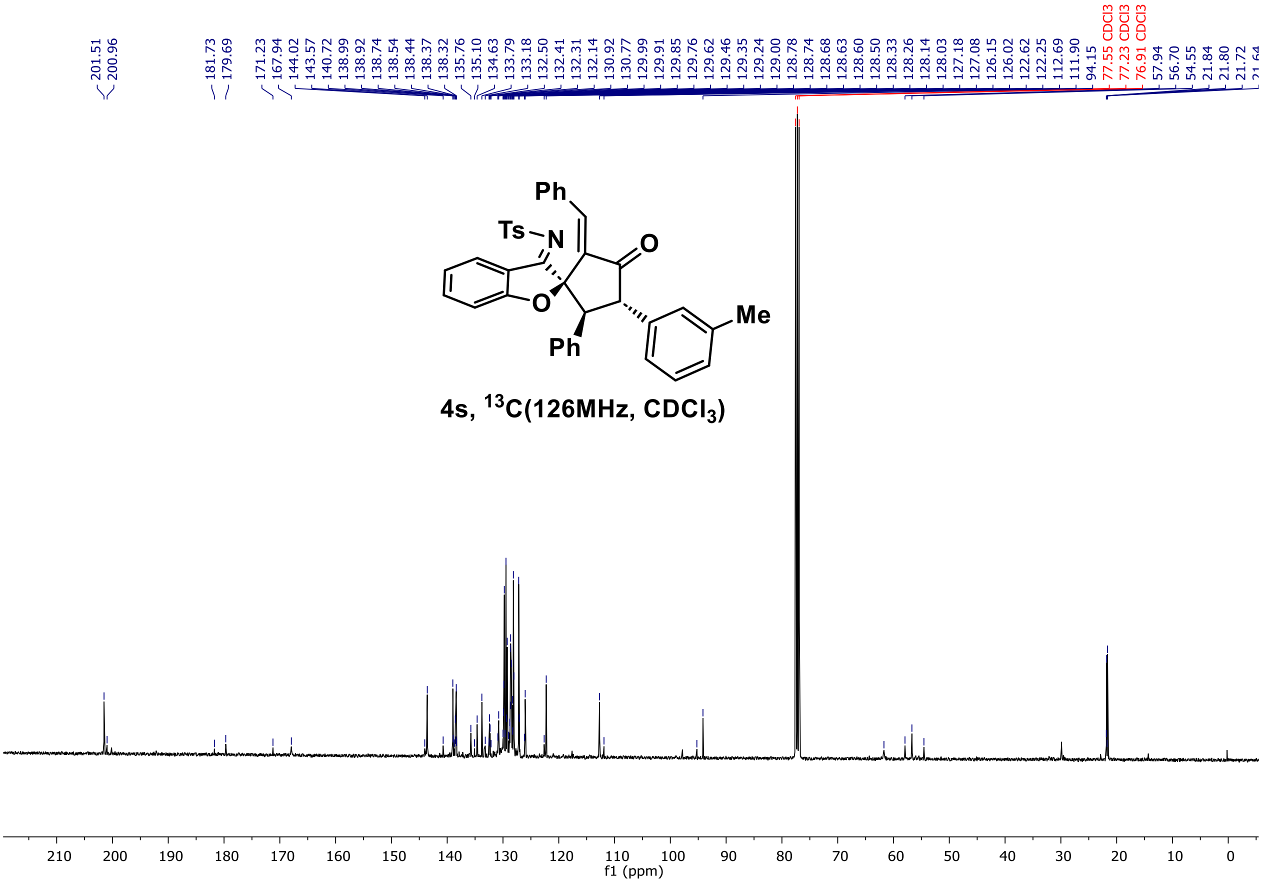
Ph
Ts
N
O
O
Ph
Me
4s, ^{13}C(126MHz, CDCl$_3$)
f1 (ppm)

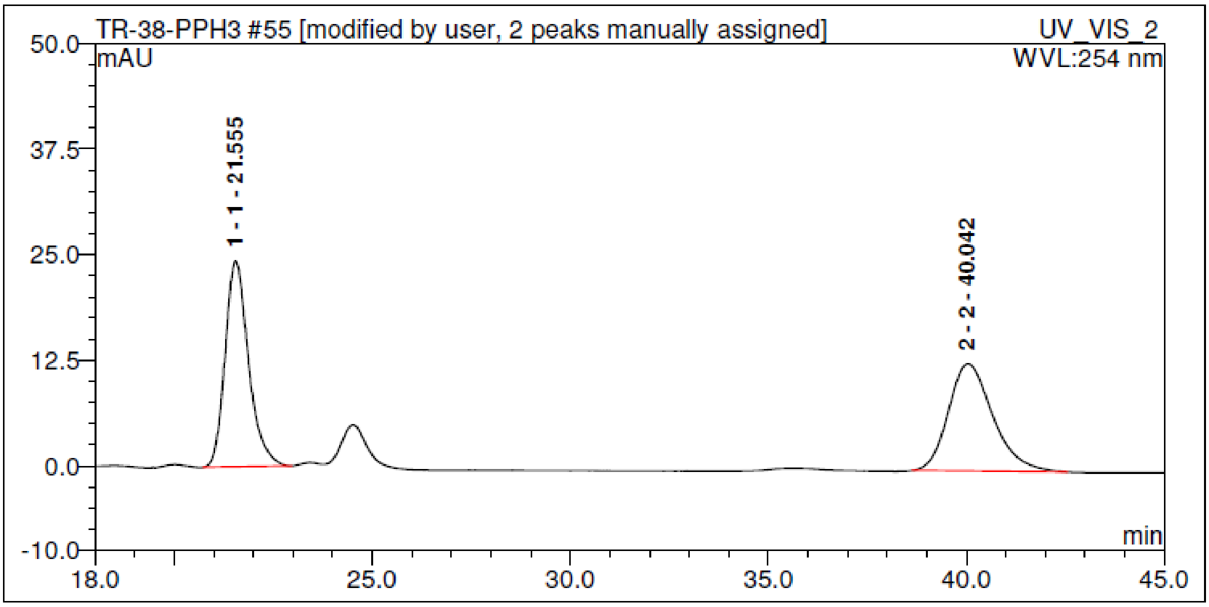

Peak	Name	Ret.Time (detected) min	Area mAU*min	Rel.Area(ident.) %	Height mAU	Amount
1	1	21.56	16.75679	50.38114535	24.38641	n.a.
2	2	40.04	16.503	49.61885465	12.693	n.a.

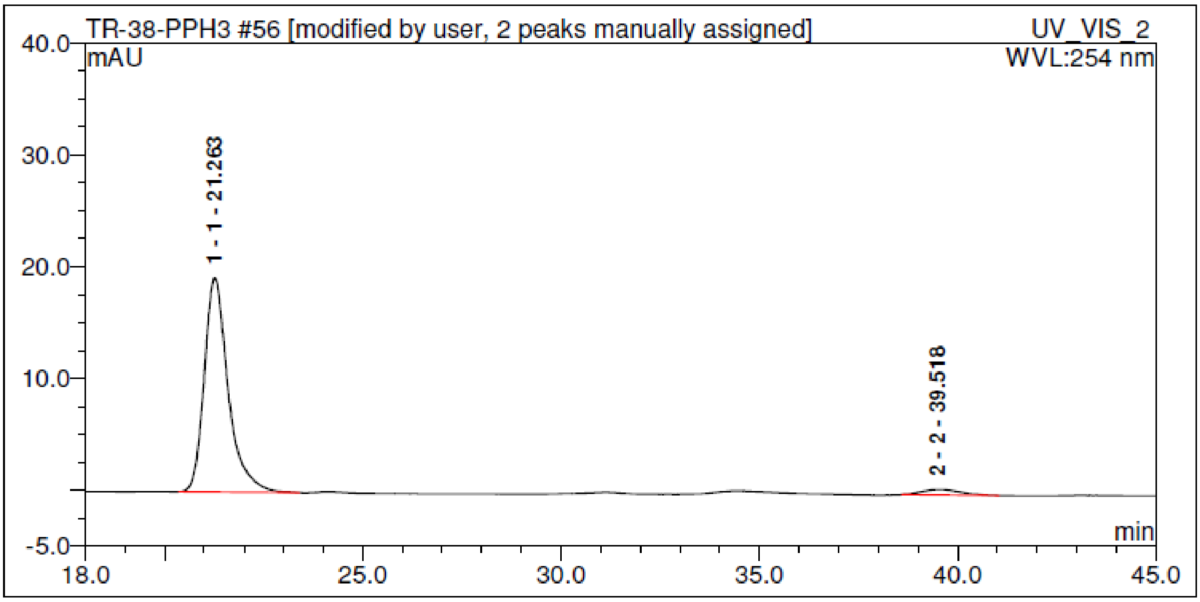

Peak	Name	Ret.Time (detected) min	Area mAU*min	Rel.Area(ident.) %	Height mAU	Amount
1	1	21.26	13.44979	96.05848386	19.15092	n.a.
2	2	39.52	0.552	3.941516142	0.489	n.a.

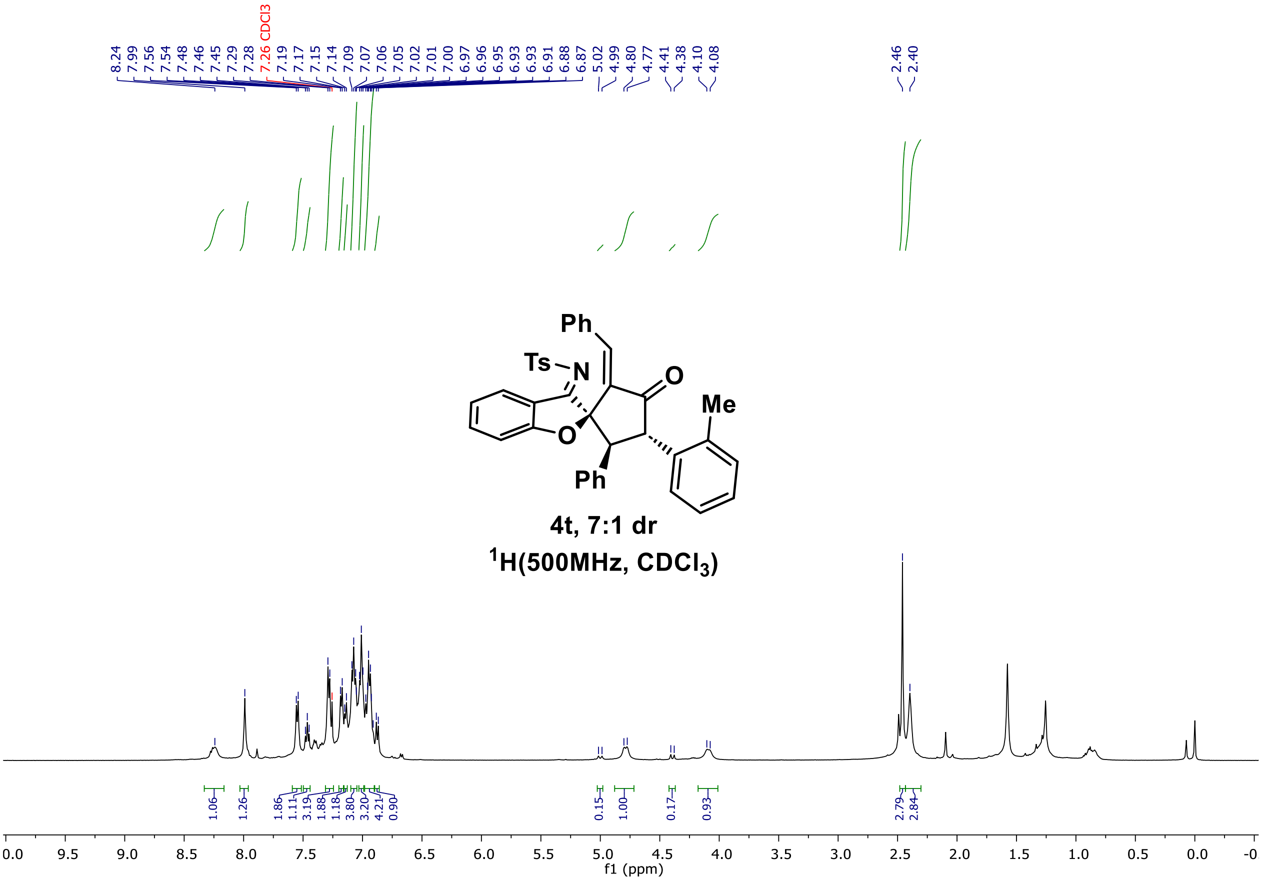

4t, 7:1 dr

^{1}H(500MHz, $CDCl_3$)

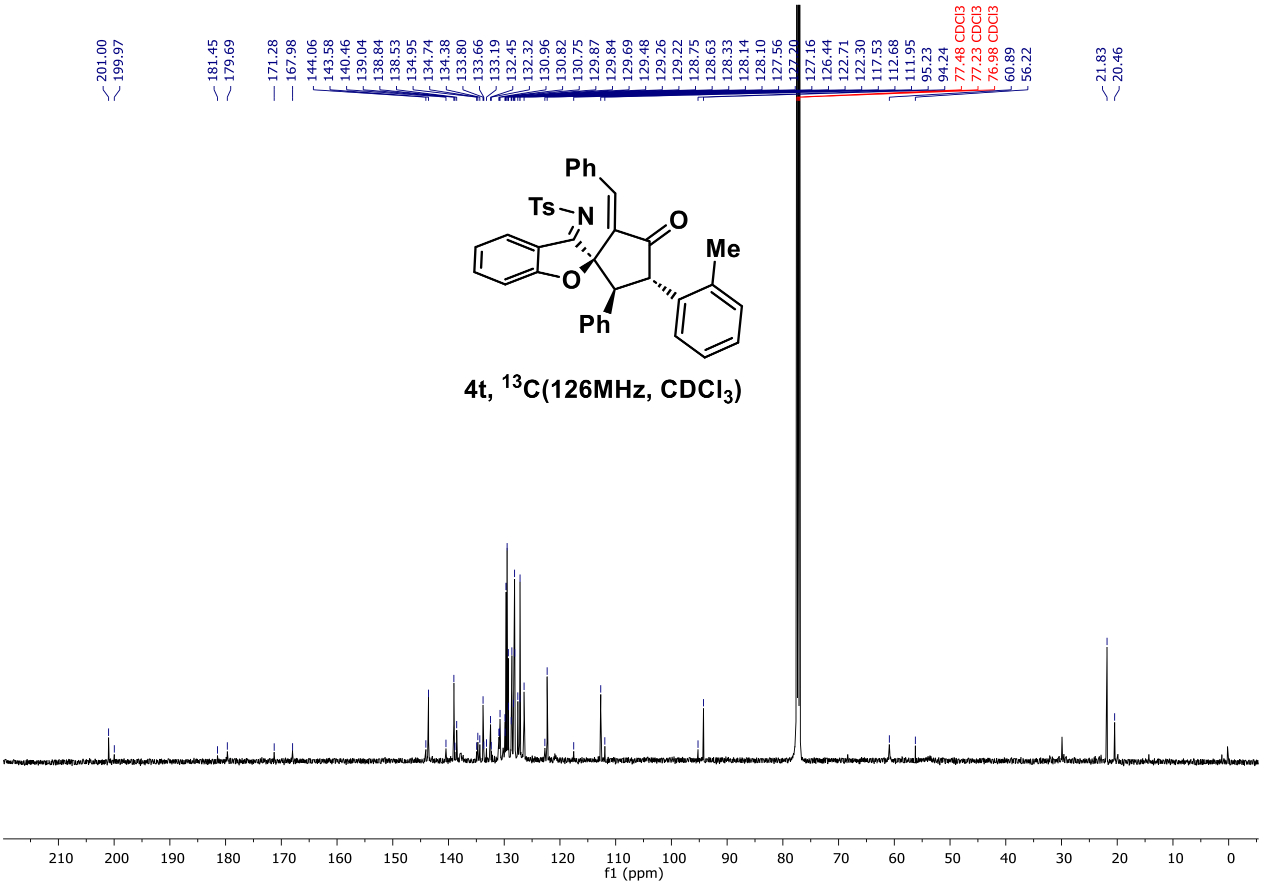

4t, ^{13}C(126MHz, $CDCl_3$)

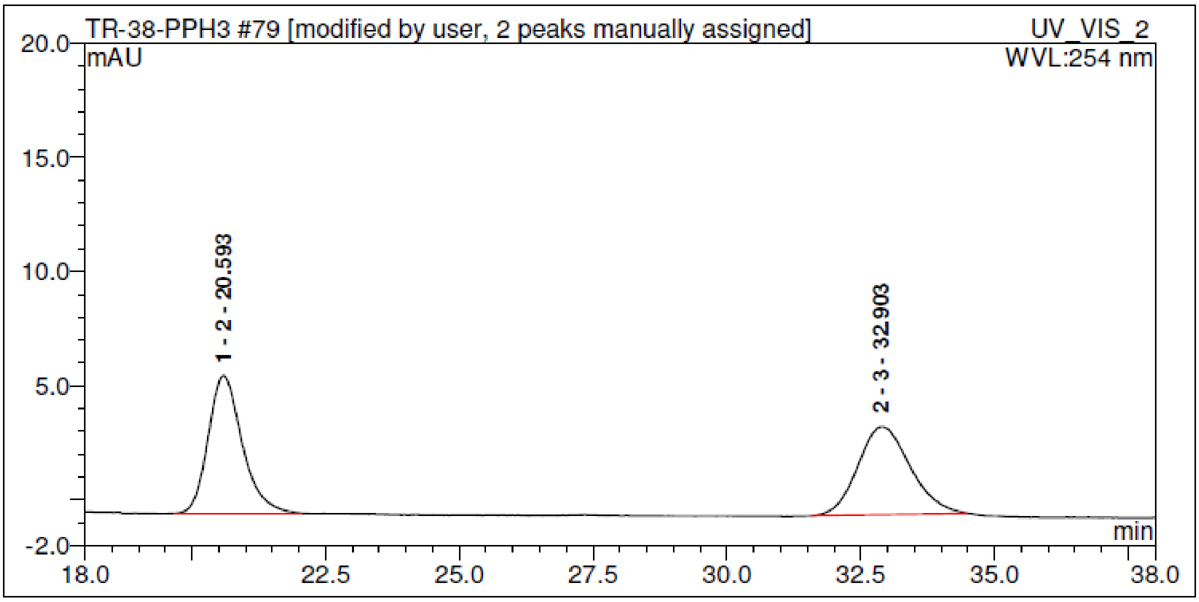

Peak	Name	Ret.Time (detected) min	Area mAU*min	Rel.Area(ident.) %	Height mAU	Amount
1	2	20.59	4.445955	50.46266276	6.0217	n.a.
2	3	32.90	4.364	49.53733724	3.851	n.a.

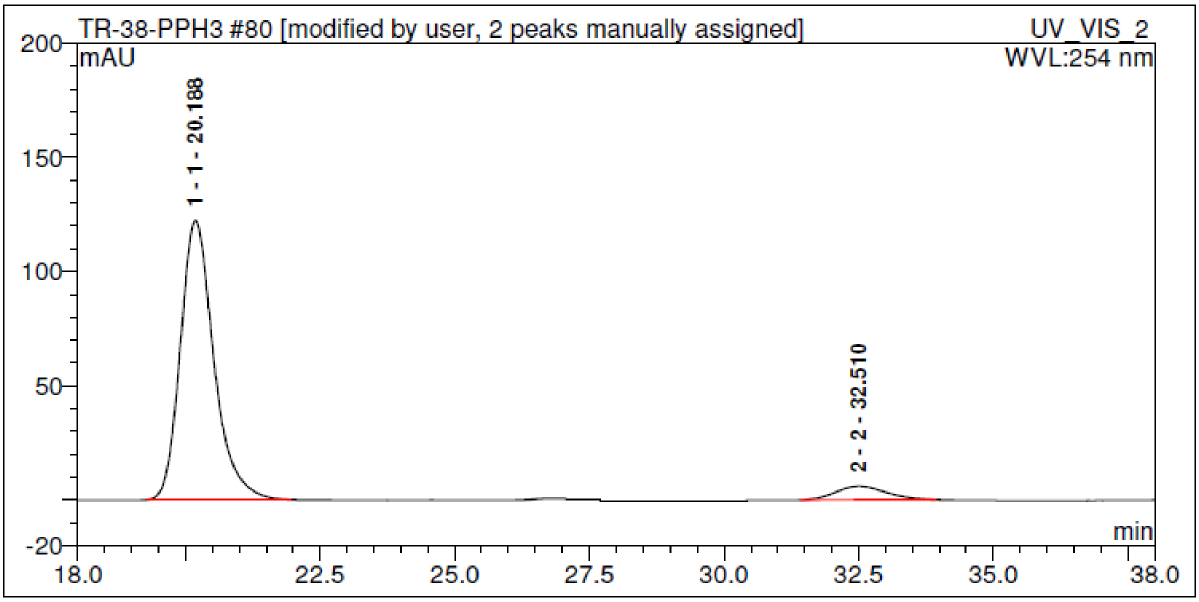

Peak	Name	Ret.Time (detected) min	Area mAU*min	Rel.Area(ident.) %	Height mAU	Amount
1	1	20.19	88.05592	93.18466099	122.0815	n.a.
2	2	32.51	6.440	6.815339012	5.973	n.a.

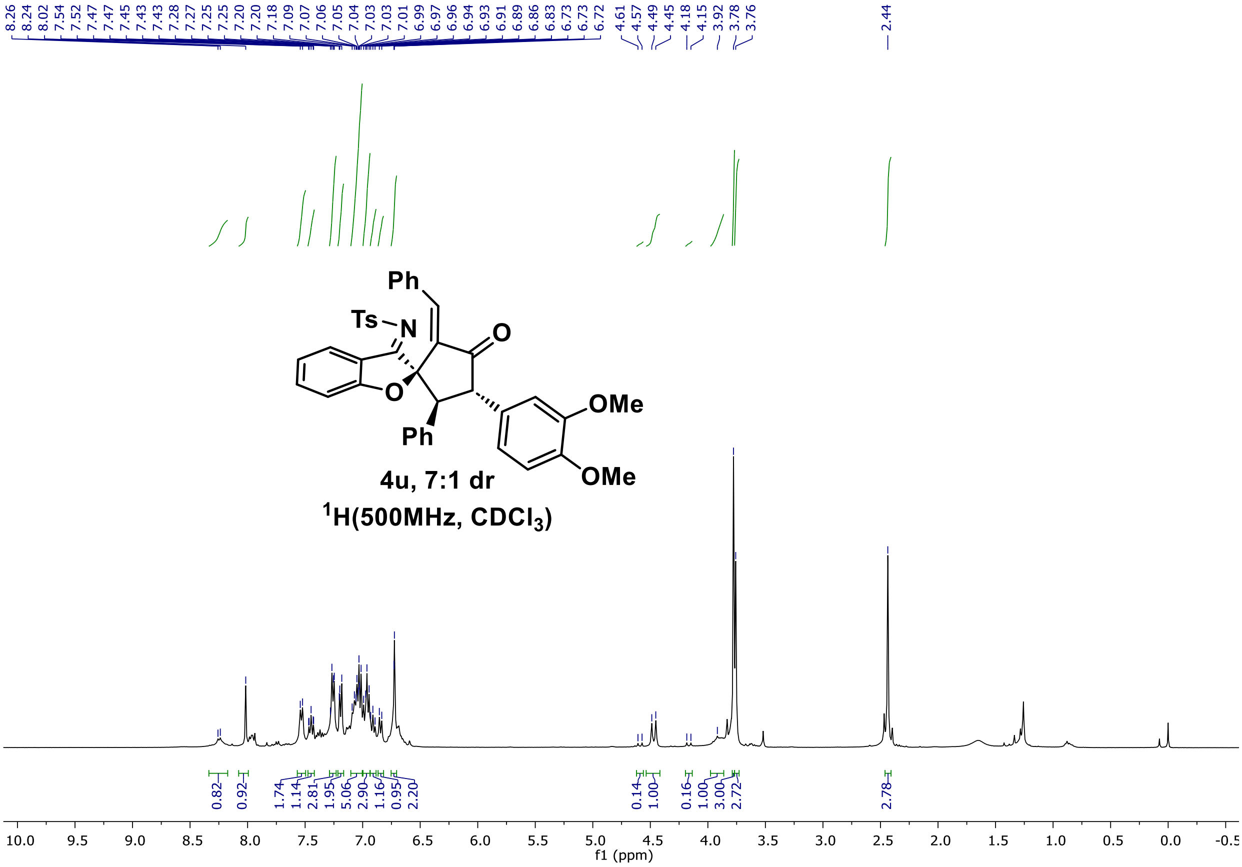
Ph
Ts
N
O
O
Ph
OMe
OMe
4u, 7:1 dr
1H(500MHz, CDCl3)
f1 (ppm)

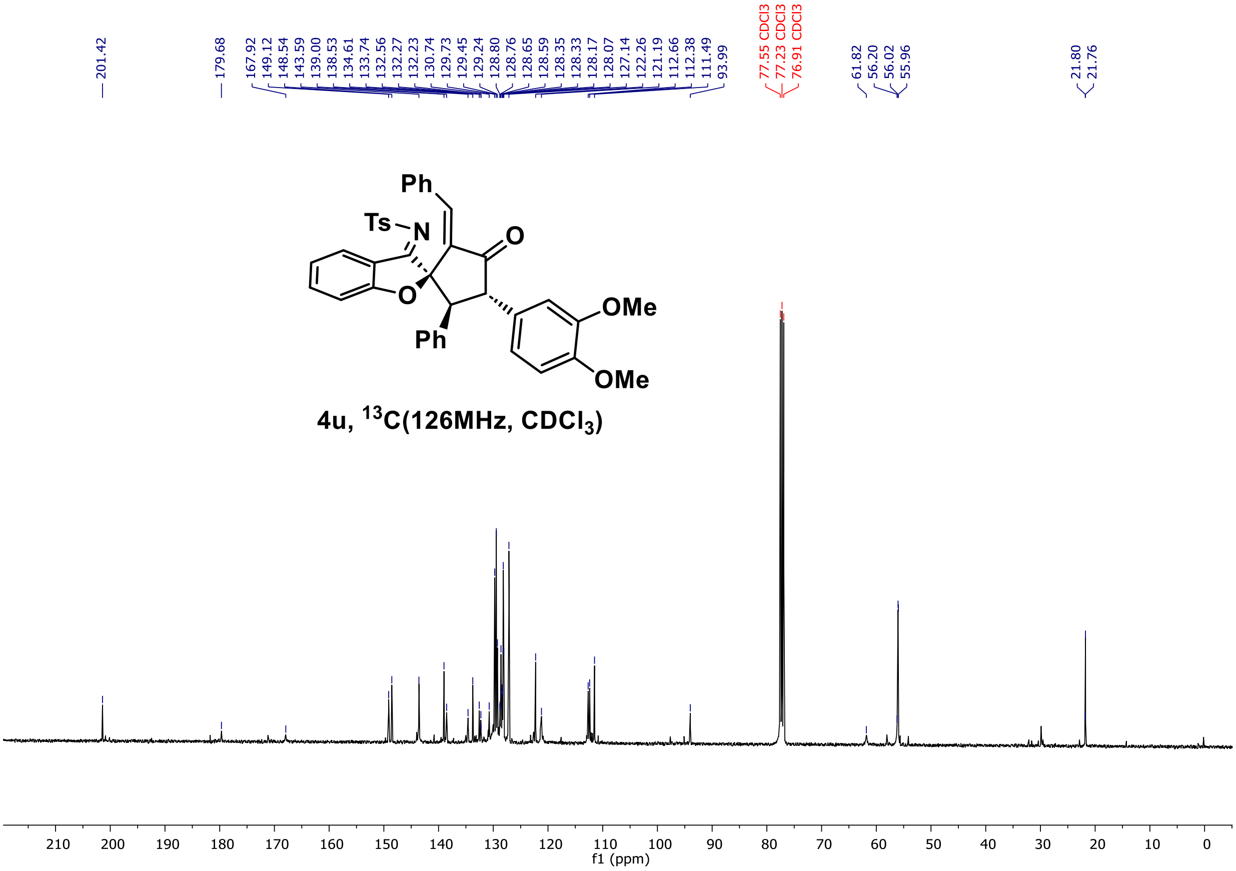
Ph
Ts
N
O
O
Ph
OMe
OMe
4u, 13C(126MHz, CDCl3)
f1 (ppm)

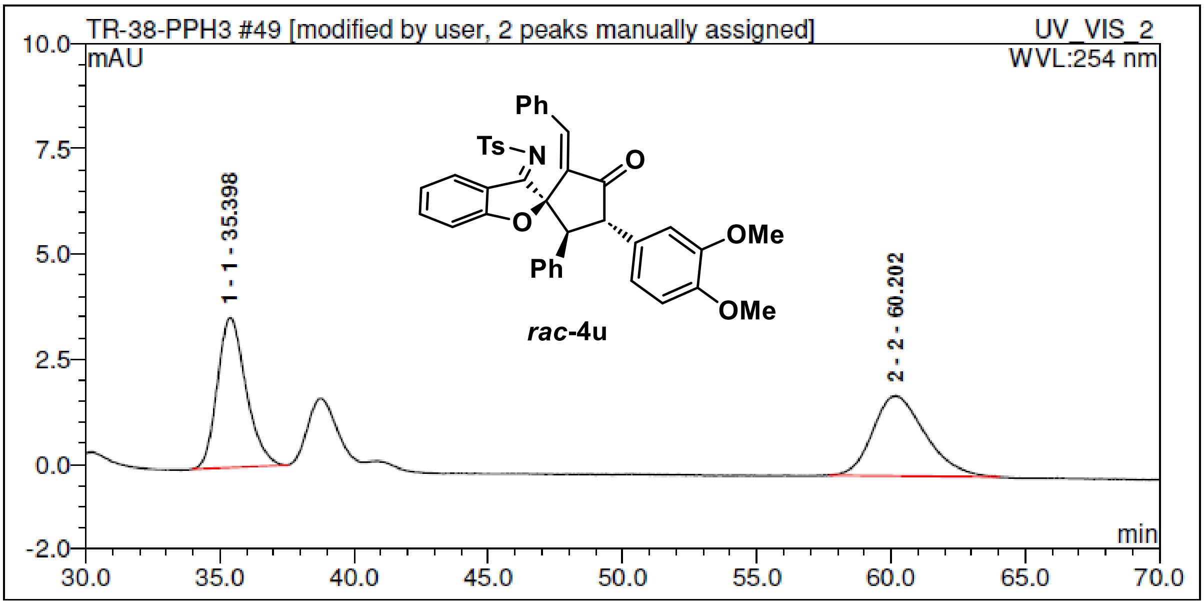

	Peak Name	Ret.Time (detected) min	Area mAU*min	Rel.Area(ident.) %	Height mAU	Amount
1	1	35.40	4.412084	50.58122251	3.56209	n.a.
2	2	60.20	4.311	49.41877749	1.902	n.a.

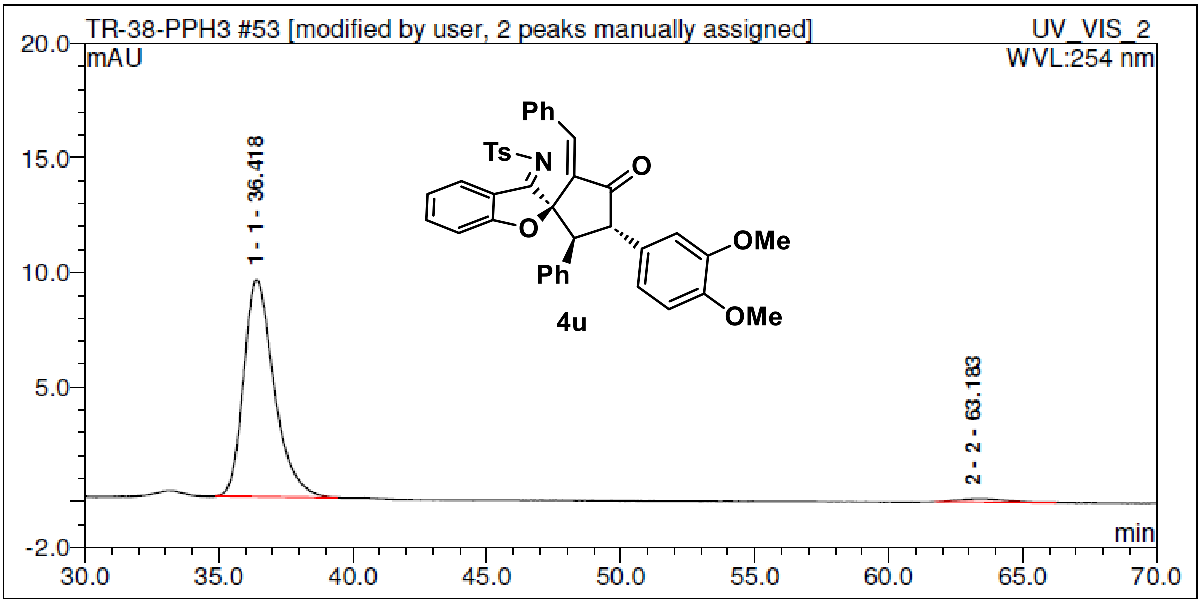

	Peak Name	Ret.Time (detected) min	Area mAU*min	Rel.Area(ident.) %	Height mAU	Amount
1	1	36.42	12.50898	97.48672608	9.47894	n.a.
2	2	63.18	0.322	2.513273924	0.151	n.a.

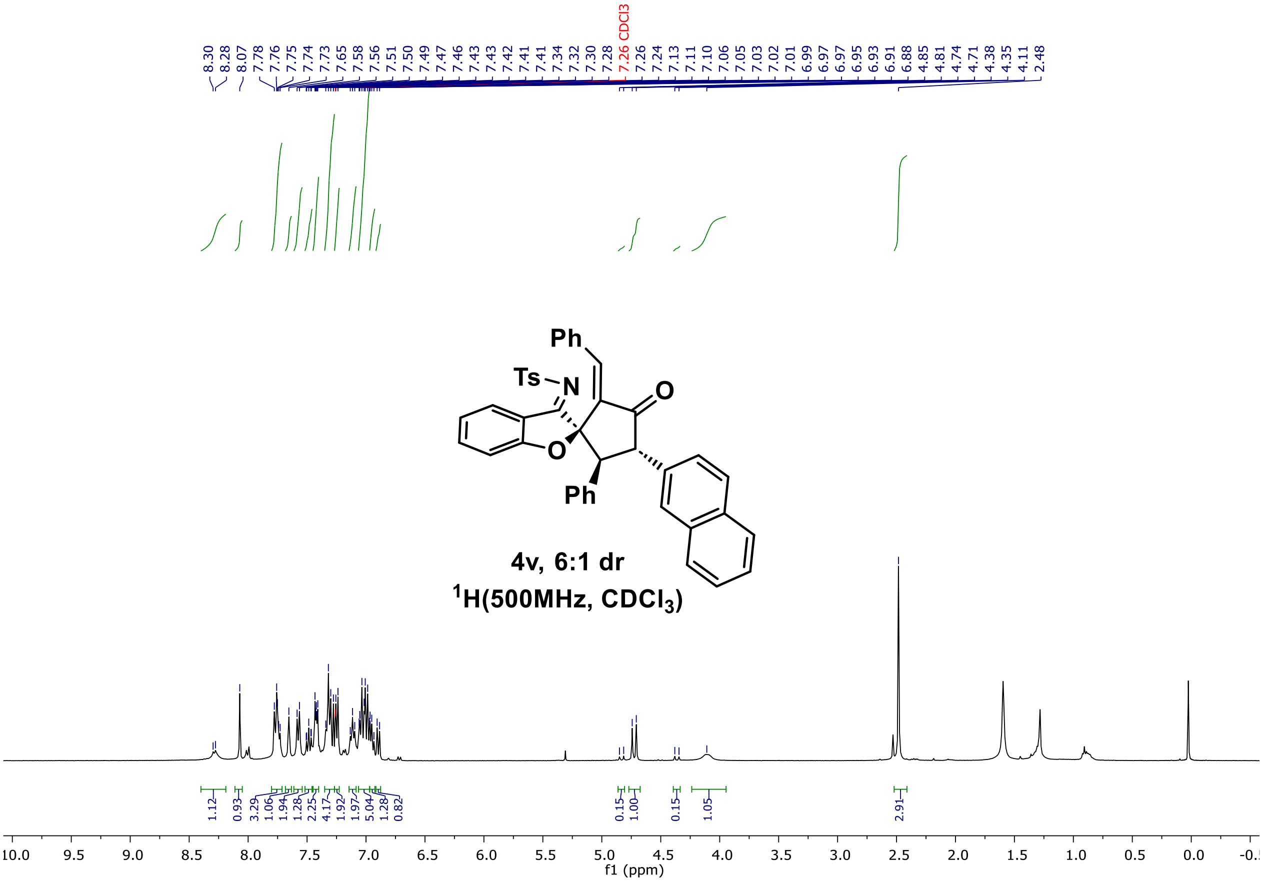
8.30 8.28 8.07 7.78 7.76 7.75 7.74 7.73 7.65 7.58 7.56 7.51 7.50 7.49 7.47 7.46 7.43 7.43 7.42 7.41 7.41 7.34 7.32 7.30 7.28 7.26 CDCl3 7.26 7.24 7.13 7.11 7.10 7.06 7.05 7.03 7.02 7.01 6.99 6.97 6.97 6.95 6.93 6.91 6.88 4.85 4.81 4.74 4.71 4.38 4.35 4.11 2.48
Ph
Ts
N
O
O
Ph
4v, 6:1 dr
1H(500MHz, CDCl3)
1.12 0.93 3.29 1.06 1.94 1.28 2.25 4.17 1.92 1.97 5.04 1.28 0.82 0.15 1.00 0.15 1.05 2.91
f1 (ppm)

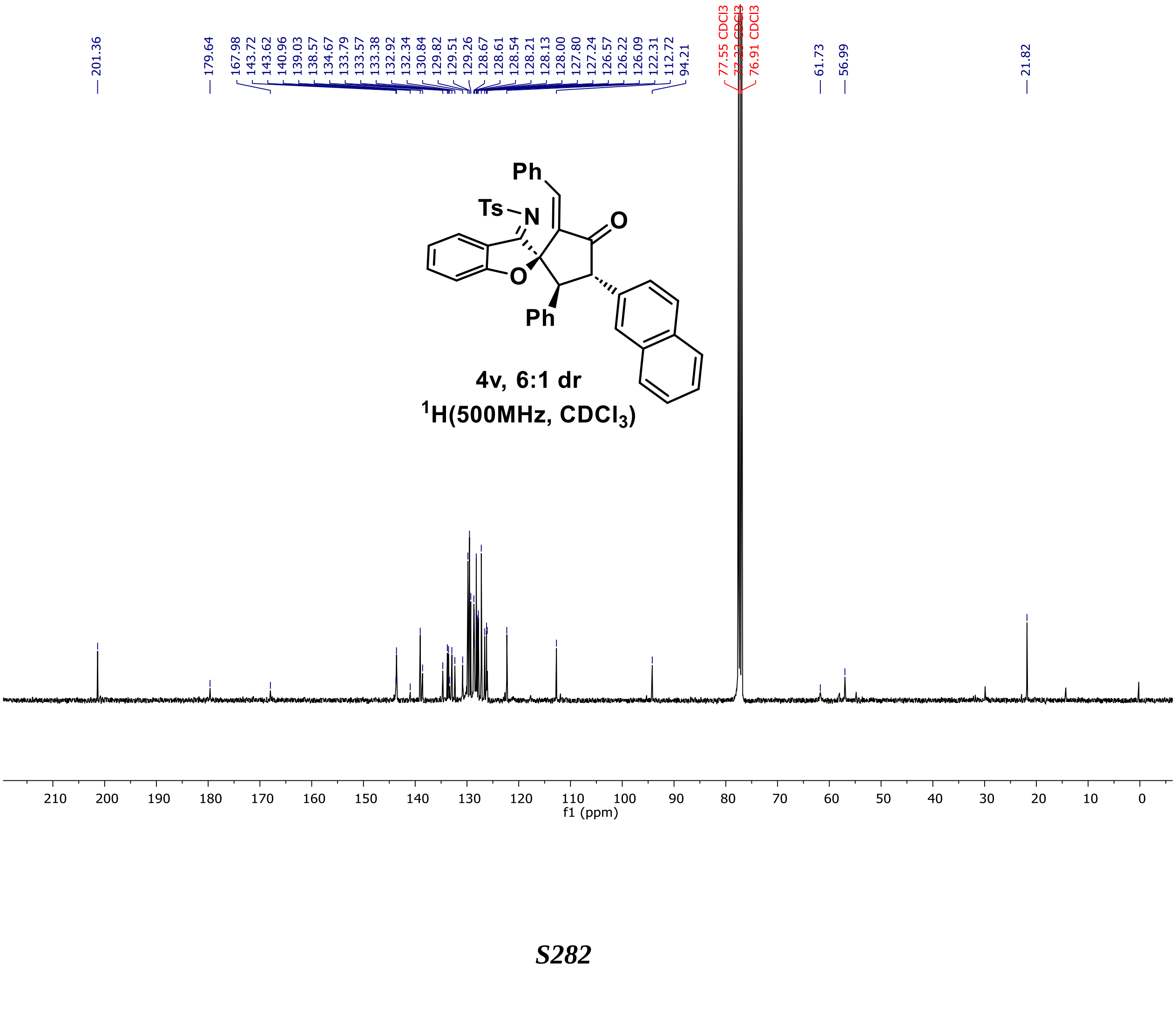
201.36 179.64 167.98 143.72 143.62 140.96 139.03 138.57 134.67 133.79 133.57 133.38 132.92 132.34 130.84 129.82 129.51 129.26 128.67 128.61 128.54 128.21 128.13 128.00 127.80 127.24 126.57 126.22 126.09 122.31 112.72 94.21 77.55 CDCl3 77.23 CDCl3 76.91 CDCl3 61.73 56.99 21.82
Ph
Ts
N
O
O
Ph
4v, 6:1 dr
1H(500MHz, CDCl3)
f1 (ppm)

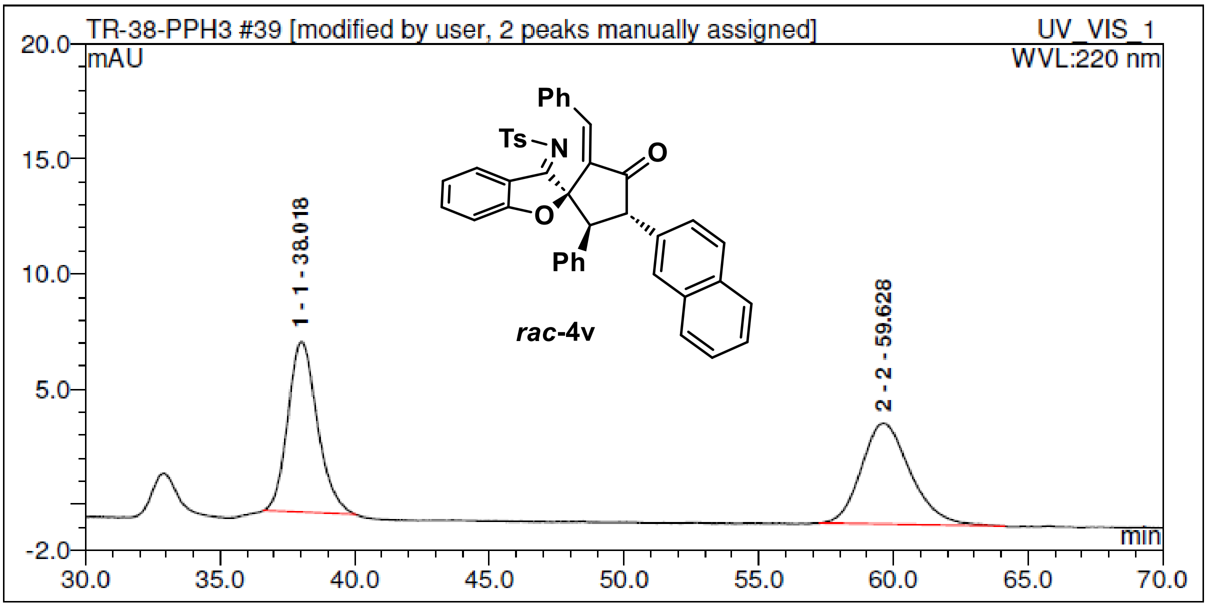

Peak Name	Ret.Time (detected) min	Area mAU*min	Rel.Area(ident.) %	Height mAU	Amount
1 1	38.02	9.427003	50.51143062	7.39474	n.a.
2 2	59.63	9.236	49.48856938	4.366	n.a.

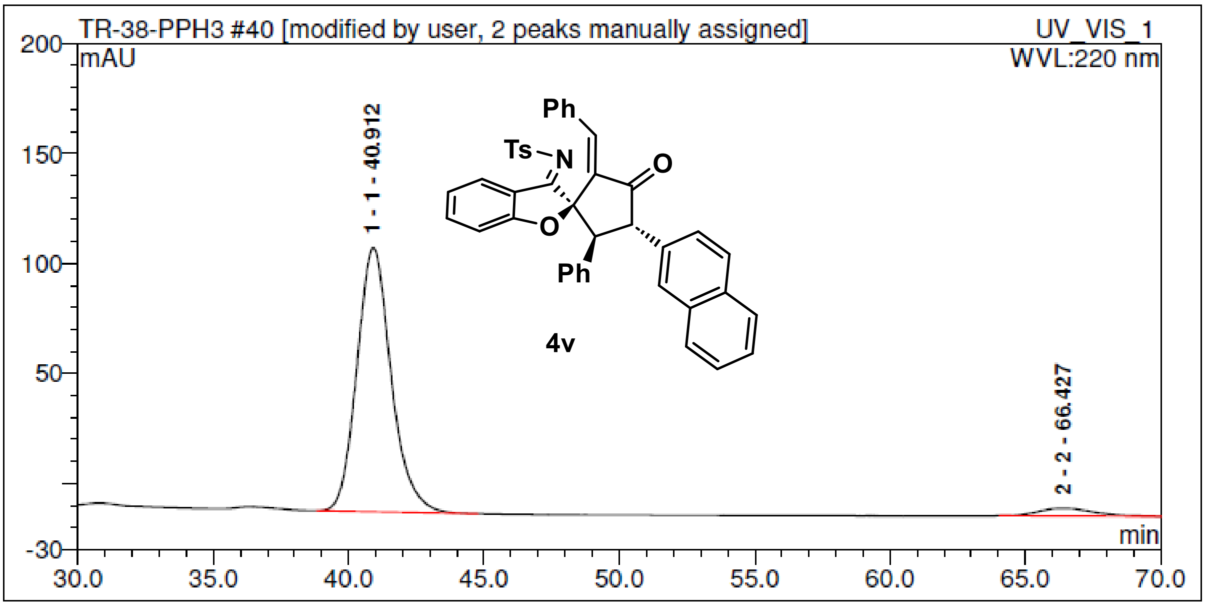

Peak Name	Ret.Time (detected) min	Area mAU*min	Rel.Area(ident.) %	Height mAU	Amount
1 1	40.91	176.305	95.36219668	119.9234	n.a.
2 2	66.43	8.574	4.637803321	3.639	n.a.

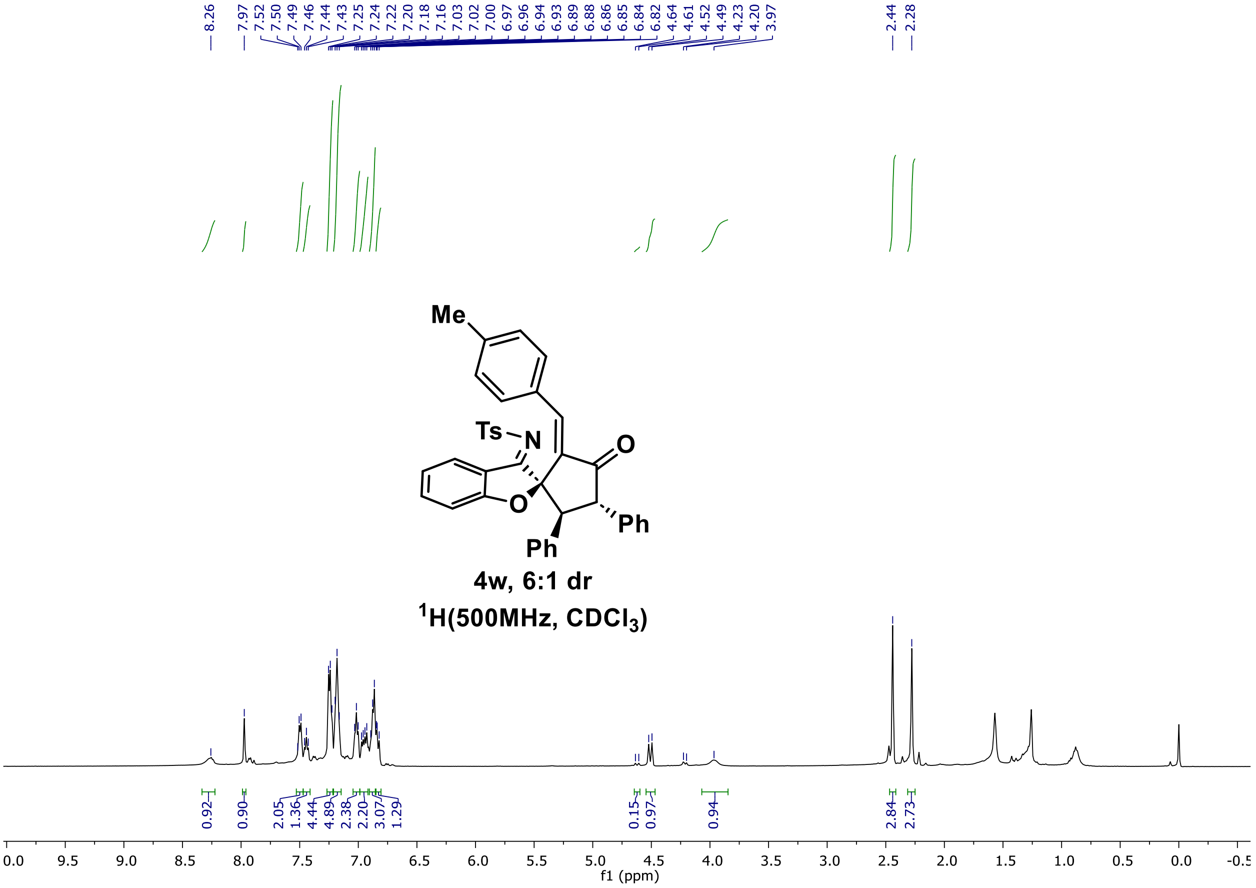
8.26
7.97
7.52
7.50
7.49
7.46
7.44
7.43
7.25
7.24
7.22
7.20
7.18
7.16
7.03
7.02
7.00
6.97
6.96
6.94
6.93
6.89
6.88
6.86
6.85
6.84
6.82
4.64
4.61
4.52
4.49
4.23
4.20
3.97
2.44
2.28
Me
Ts
N
O
O
Ph
Ph
4w, 6:1 dr
1H(500MHz, CDCl3)
0.92
0.90
2.05
1.36
4.44
4.89
2.38
2.20
3.07
1.29
0.15
0.97
0.94
2.84
2.73
f1 (ppm)

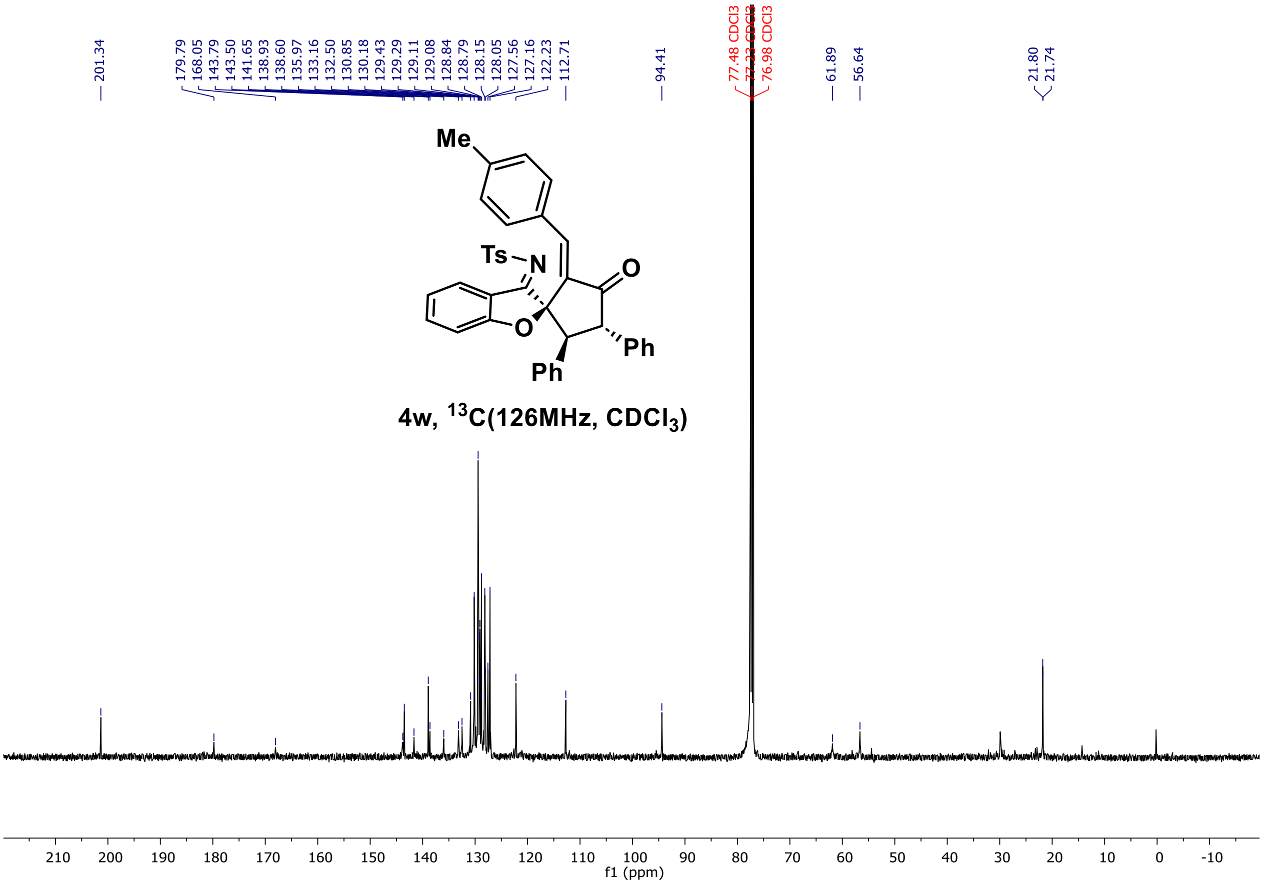
201.34
179.79
168.05
143.79
143.50
141.65
138.93
138.60
135.97
133.16
132.50
130.85
130.18
129.43
129.29
129.11
129.08
128.84
128.79
128.15
128.05
127.56
127.16
122.23
112.71
94.41
77.48 CDCl3
77.23 CDCl3
76.98 CDCl3
61.89
56.64
21.80
21.74
Me
Ts
N
O
O
Ph
Ph
4w, 13C(126MHz, CDCl3)
f1 (ppm)

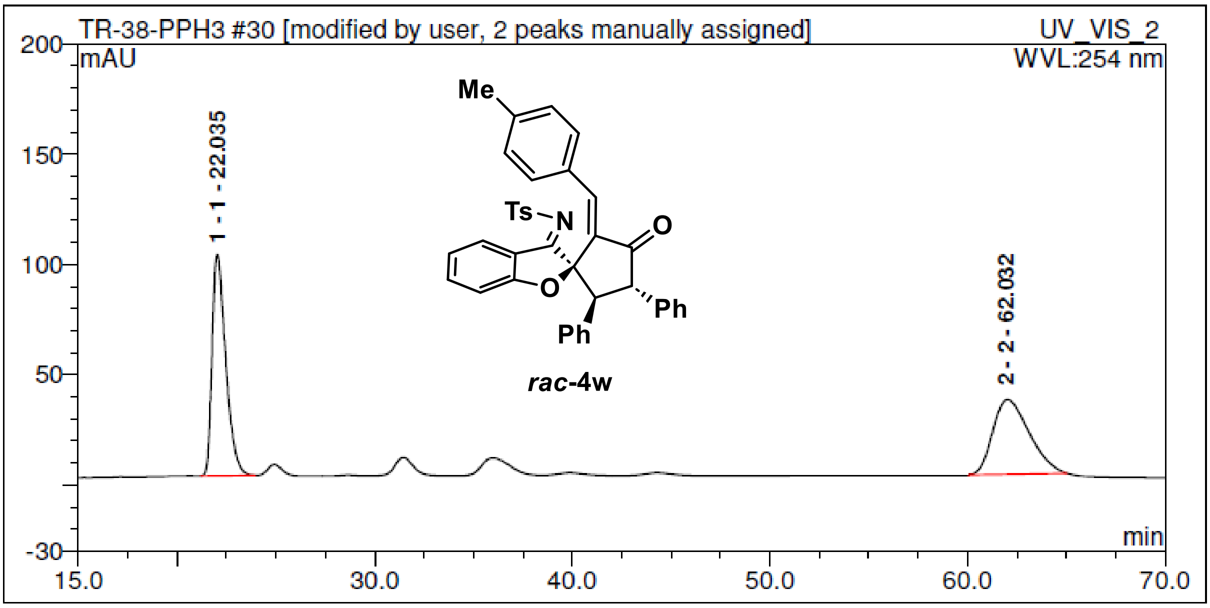

Peak Name	Ret.Time (detected) min	Area mAU*min	Rel.Area(ident.) %	Height mAU	Amount
1 1	22.04	80.29198	52.71673226	100.1274	n.a.
2 2	62.03	72.016	47.28326774	33.734	n.a.

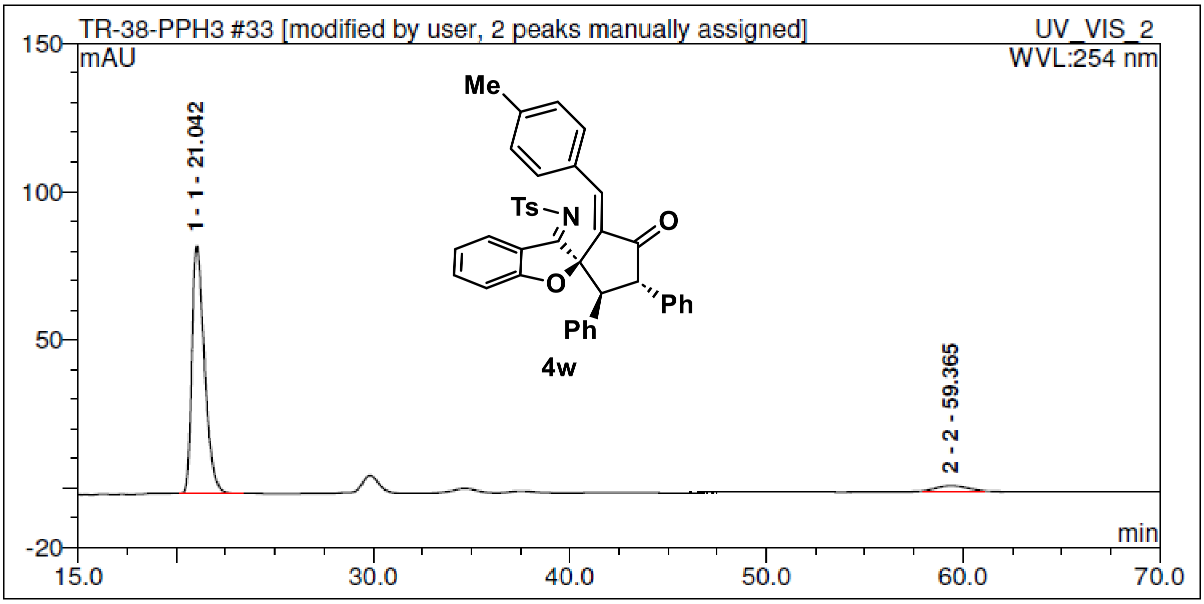

Peak Name	Ret.Time (detected) min	Area mAU*min	Rel.Area(ident.) %	Height mAU	Amount
1 1	21.04	61.54839	95.54672139	83.30613	n.a.
2 2	59.37	2.869	4.453278609	1.737	n.a.

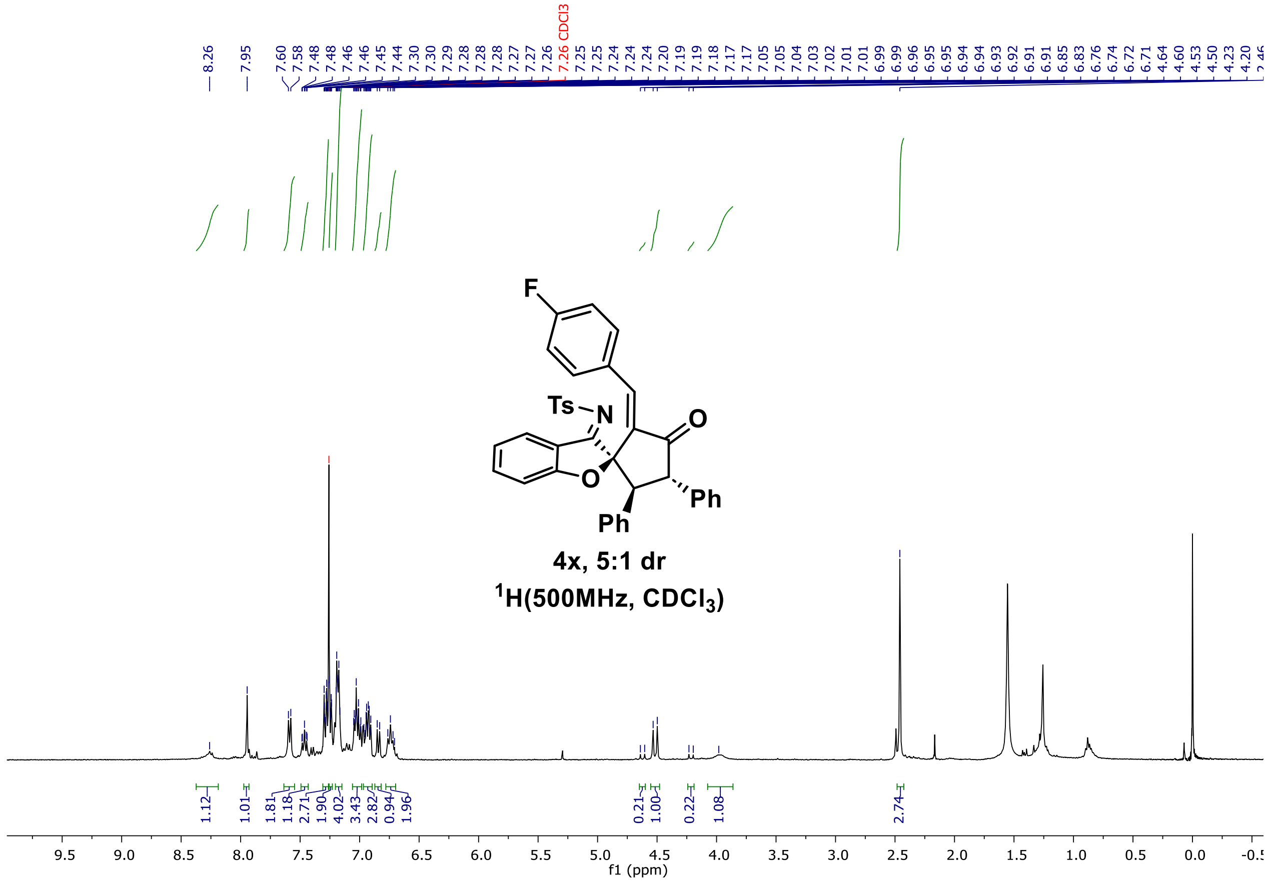
4x, 5:1 dr
1H(500MHz, CDCl3)
f1 (ppm)

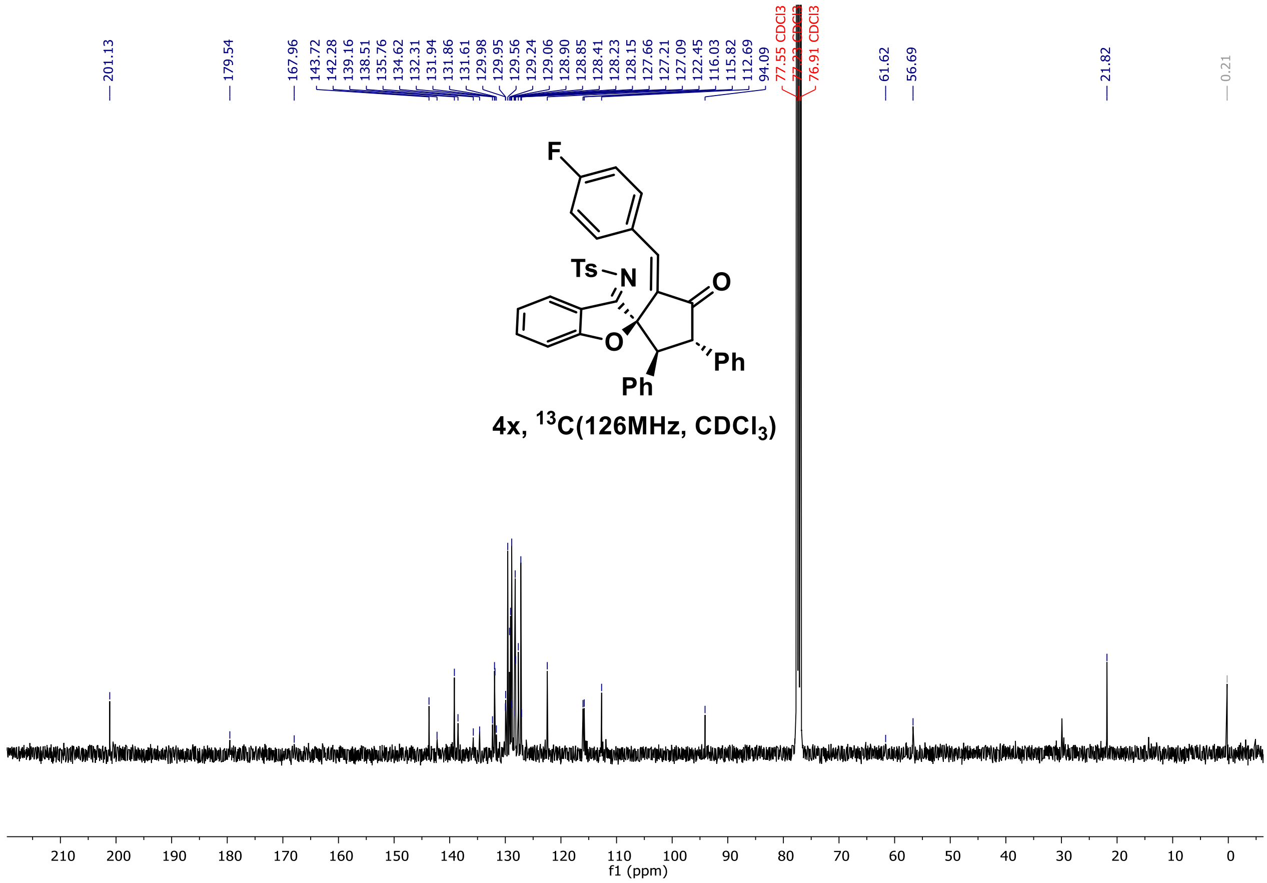
4x, 13C(126MHz, CDCl3)
f1 (ppm)

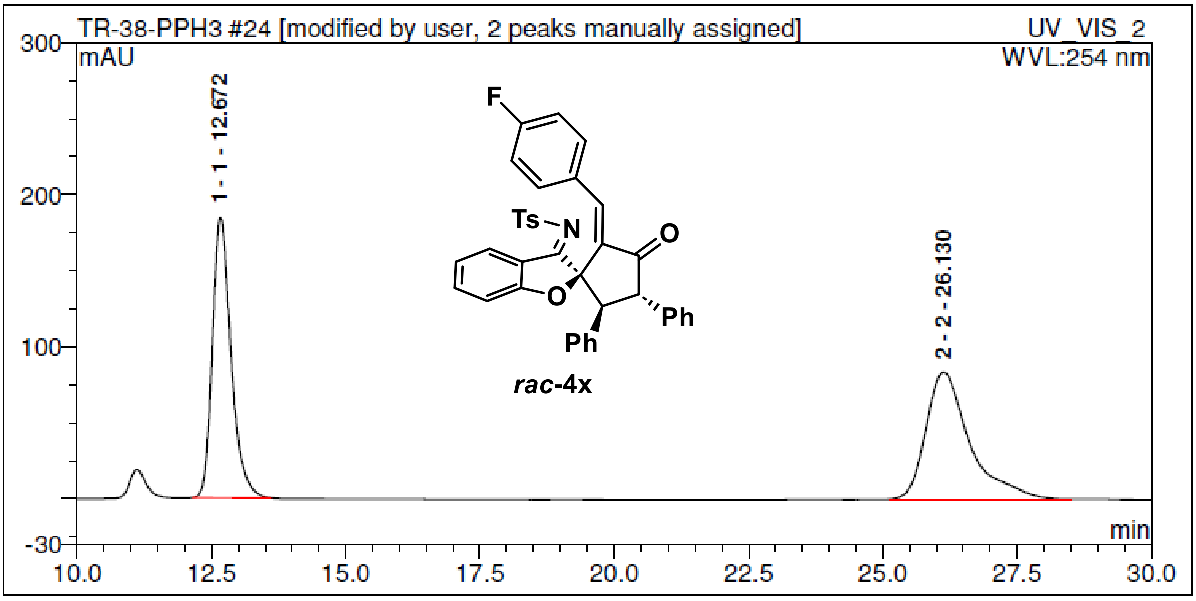

Peak	Name	Ret.Time (detected) min	Area mAU*min	Rel.Area(ident.) %	Height mAU	Amount
1	1	12.67	74.47225	48.68944756	184.1355	n.a.
2	2	26.13	78.481	51.31055244	83.495	n.a.

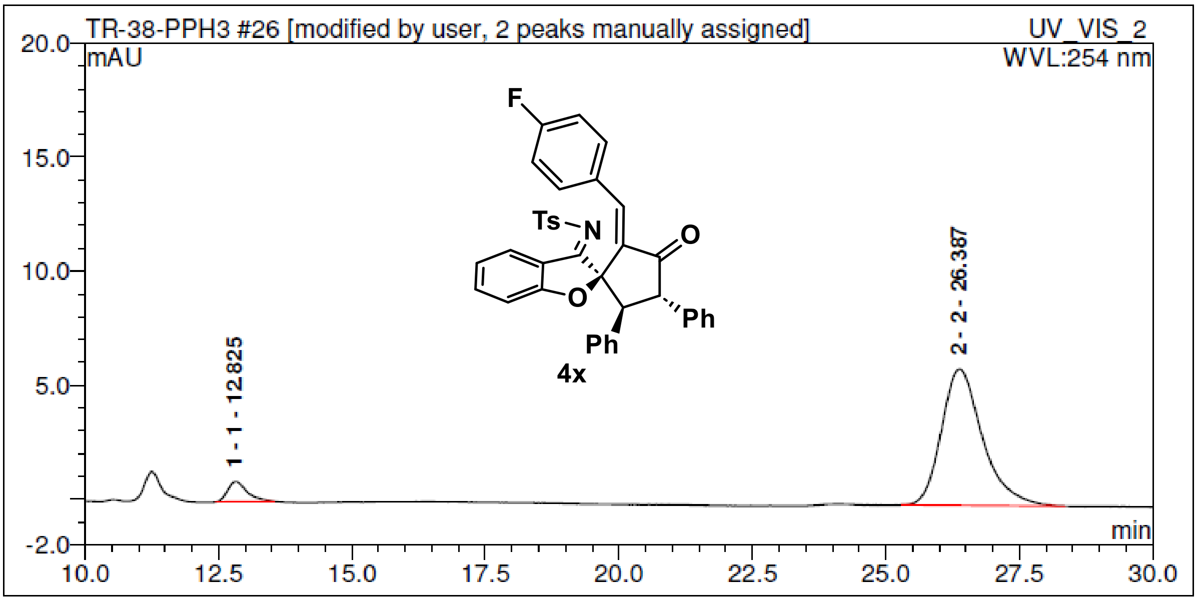

Peak	Name	Ret.Time (detected) min	Area mAU*min	Rel.Area(ident.) %	Height mAU	Amount
1	1	12.83	0.369501	6.467962436	0.88433	n.a.
2	2	26.39	5.343	93.53203756	5.977	n.a.

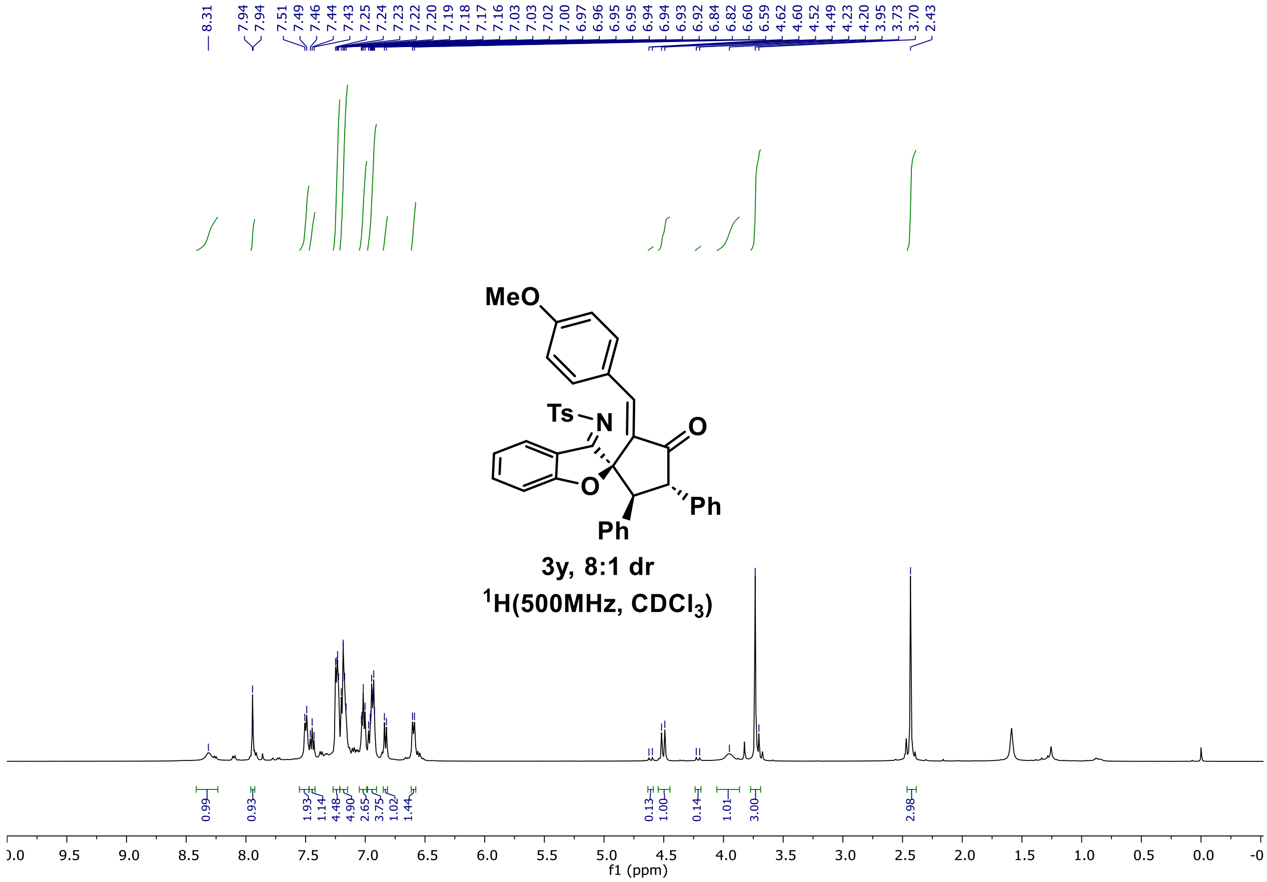
8.31
7.94
7.94
7.51
7.49
7.46
7.44
7.43
7.25
7.24
7.23
7.22
7.20
7.19
7.18
7.17
7.16
7.03
7.03
7.02
7.00
6.97
6.96
6.95
6.95
6.94
6.94
6.93
6.92
6.84
6.82
6.60
6.59
4.62
4.60
4.52
4.49
4.23
4.20
3.95
3.73
3.70
2.43
MeO
Ts
N
O
O
Ph
Ph
3y, 8:1 dr
1H(500MHz, CDCl3)
0.99
0.93
1.93
1.14
4.48
4.90
2.65
3.75
1.02
1.44
0.13
1.00
0.14
1.01
3.00
2.98
f1 (ppm)

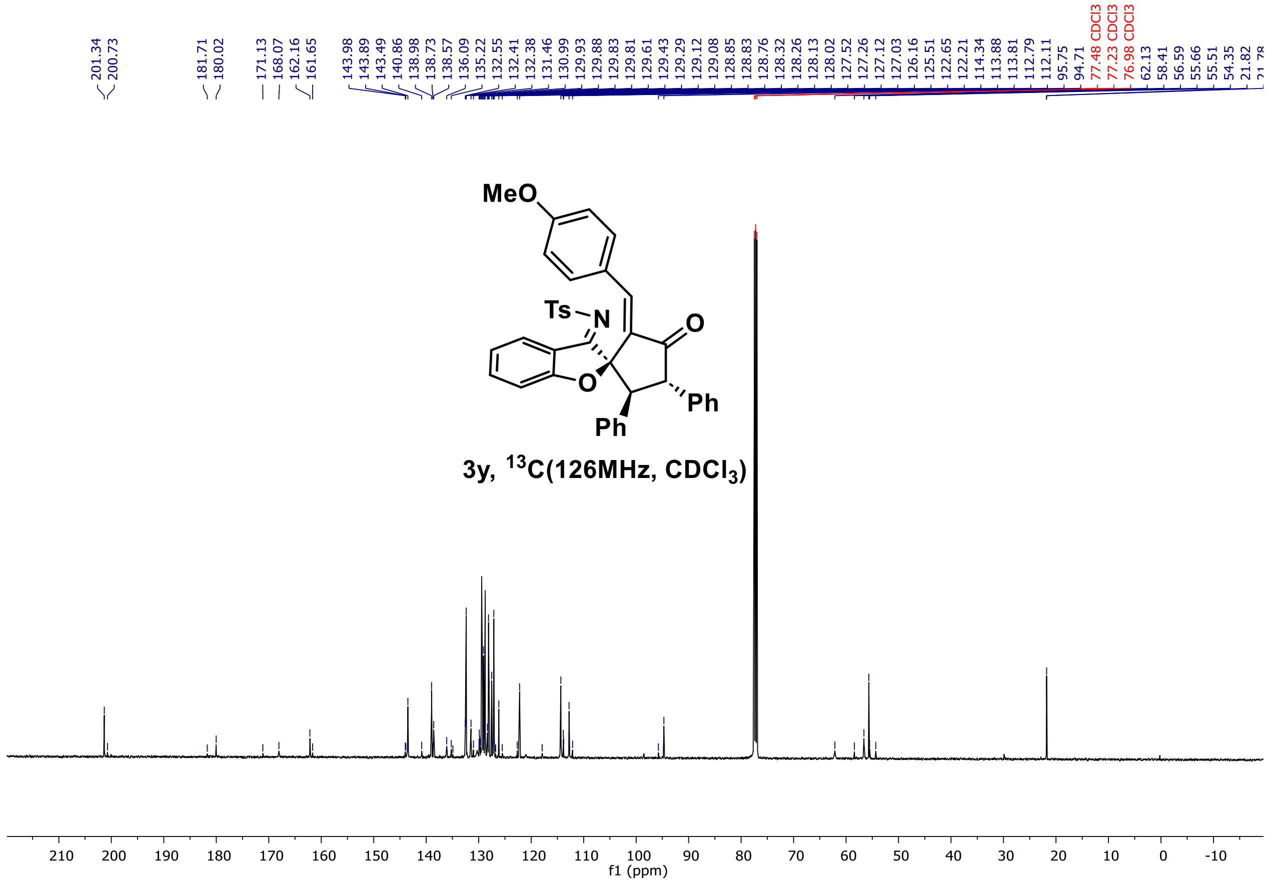
201.34
200.73
181.71
180.02
171.13
168.07
162.16
161.65
143.98
143.89
143.49
140.86
138.98
138.73
138.57
136.09
135.22
132.55
132.41
132.38
131.46
130.99
129.93
129.88
129.83
129.81
129.61
129.43
129.29
129.12
129.08
128.85
128.83
128.76
128.32
128.26
128.13
128.02
127.52
127.26
127.12
127.03
126.16
125.51
122.65
122.21
114.34
113.88
113.81
112.79
112.11
95.75
94.71
77.48 CDCl3
77.23 CDCl3
76.98 CDCl3
62.13
58.41
56.59
55.66
55.51
54.35
21.82
MeO
Ts
N
O
O
Ph
Ph
3y, 13C(126MHz, CDCl3)
f1 (ppm)

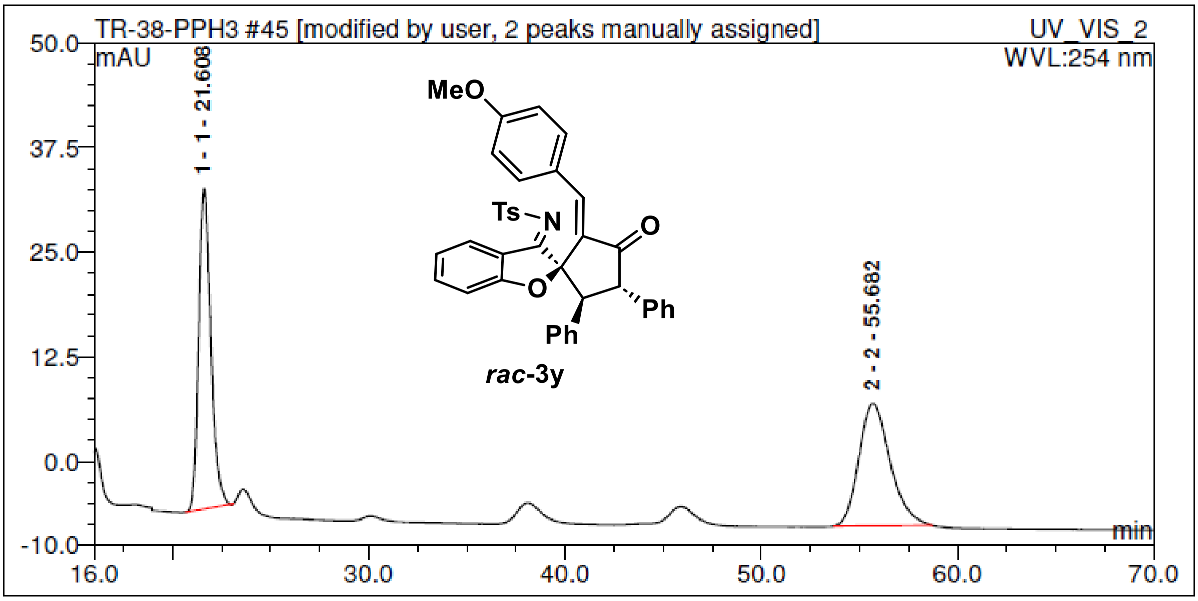

	Peak Name	Ret.Time (detected) min	Area mAU*min	Rel.Area(ident.) %	Height mAU	Amount
1	1	21.61	27.38952	50.40200943	38.26391	n.a.
2	2	55.68	26.953	49.59799057	14.574	n.a.

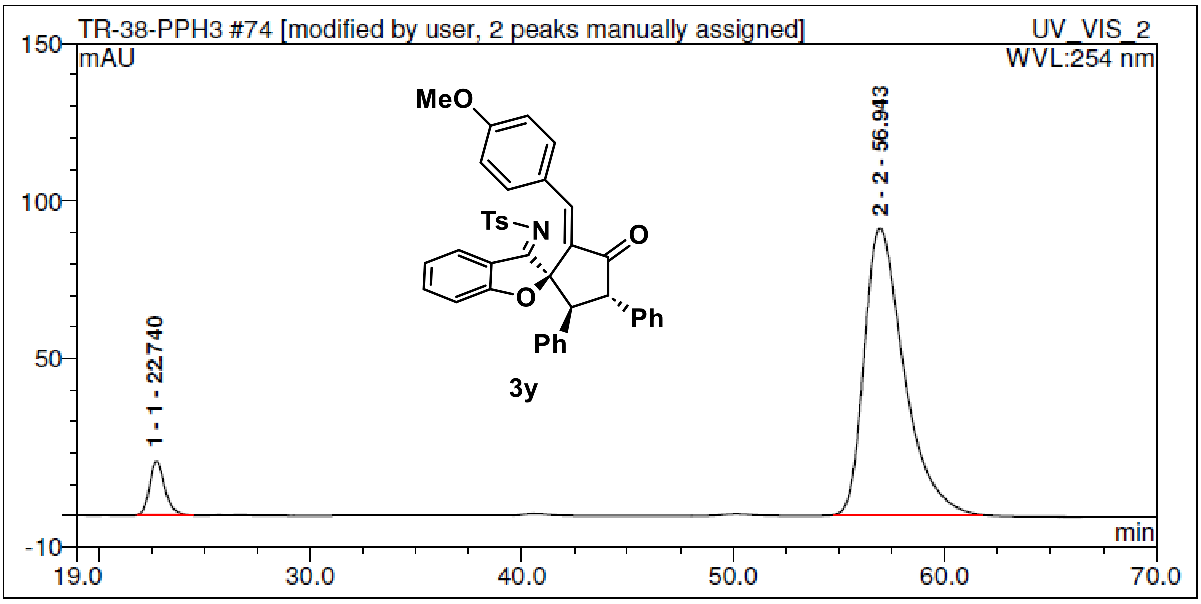

	Peak Name	Ret.Time (detected) min	Area mAU*min	Rel.Area(ident.) %	Height mAU	Amount
1	1	22.74	14.3231	6.731066067	17.10321	n.a.
2	2	56.94	198.468	93.26893393	91.010	n.a.

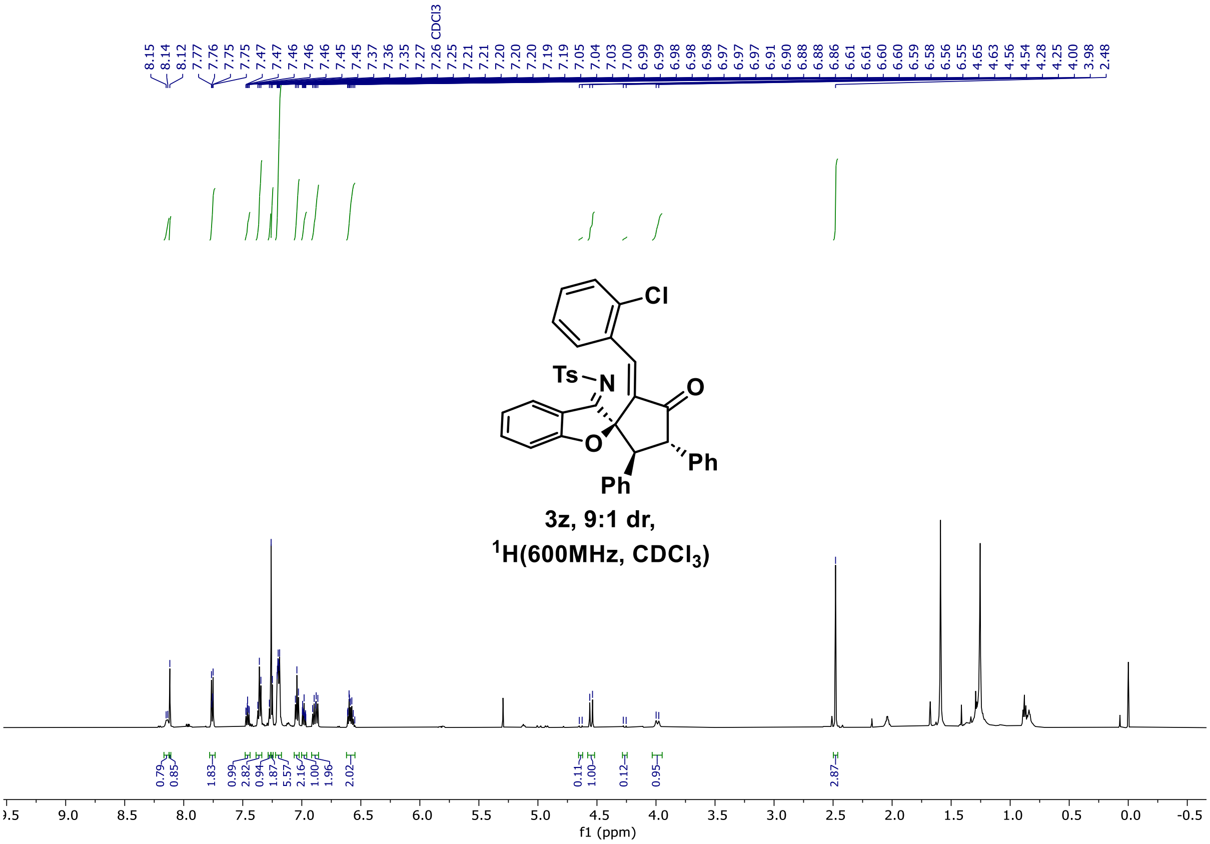
3z, 9:1 dr,
^{1}H(600MHz, CDCl$_3$)
f1 (ppm)

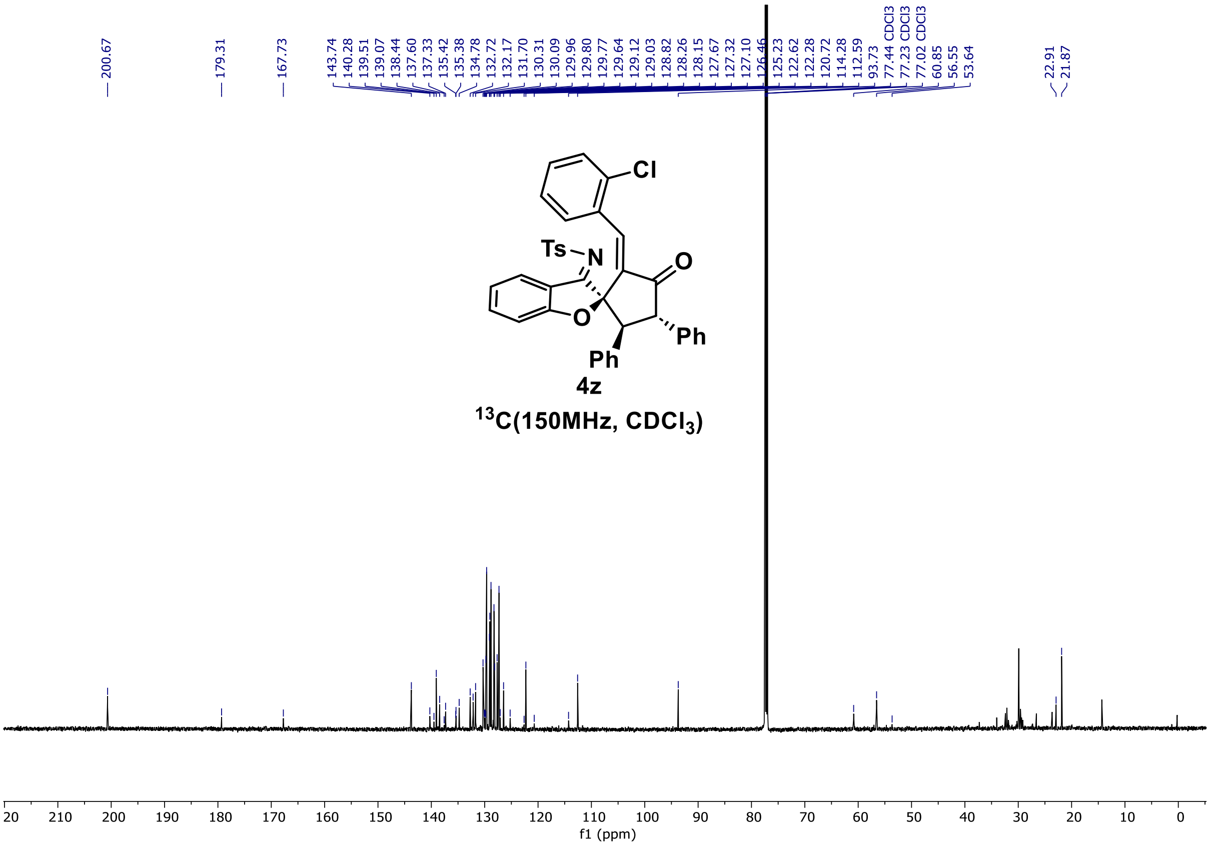
4z
^{13}C(150MHz, CDCl$_3$)
f1 (ppm)

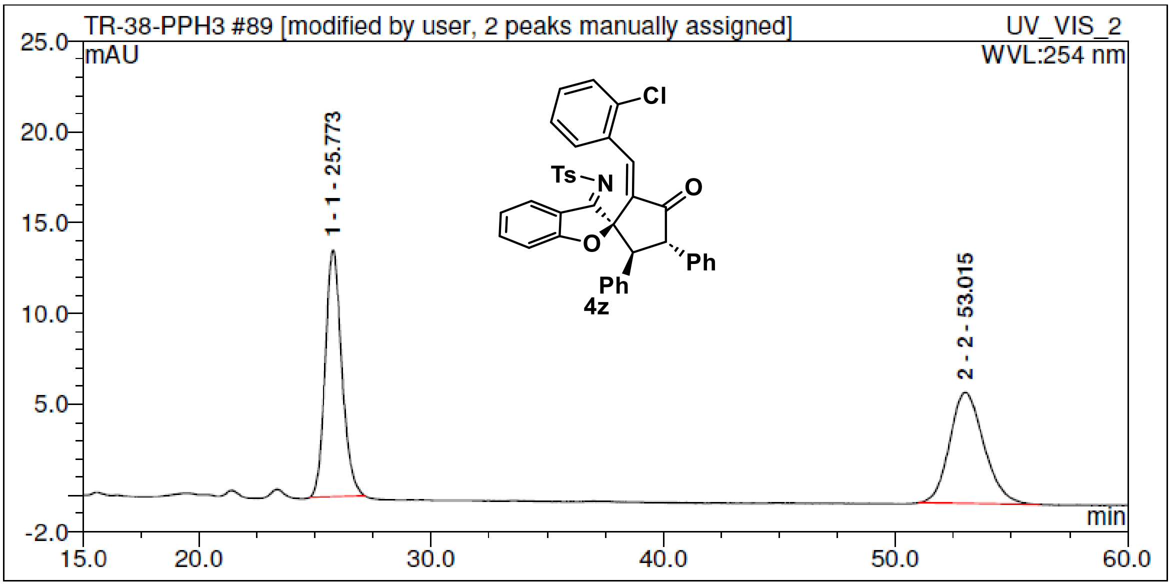

	Peak Name	Ret.Time (detected) min	Area mAU*min	Rel.Area(ident.) %	Height mAU	Amount
1	1	25.77	11.22304	51.82261625	13.56417	n.a.
2	2	53.02	10.434	48.17738375	6.100	n.a.

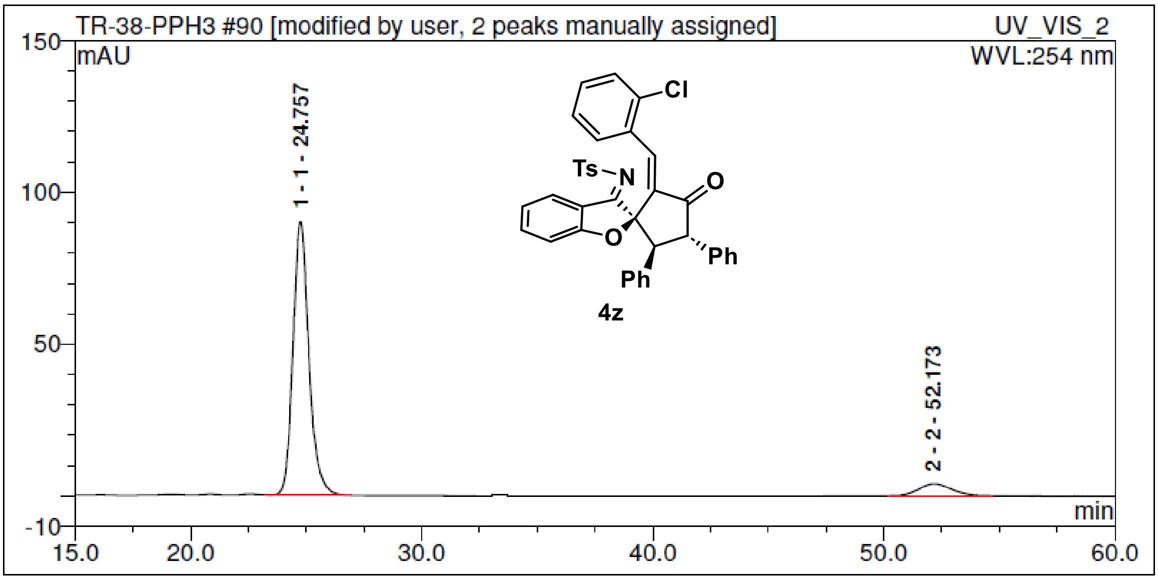

	Peak Name	Ret.Time (detected) min	Area mAU*min	Rel.Area(ident.) %	Height mAU	Amount
1	1	24.76	71.8033	91.66000281	90.03193	n.a.
2	2	52.17	6.533	8.339997188	3.898	n.a.

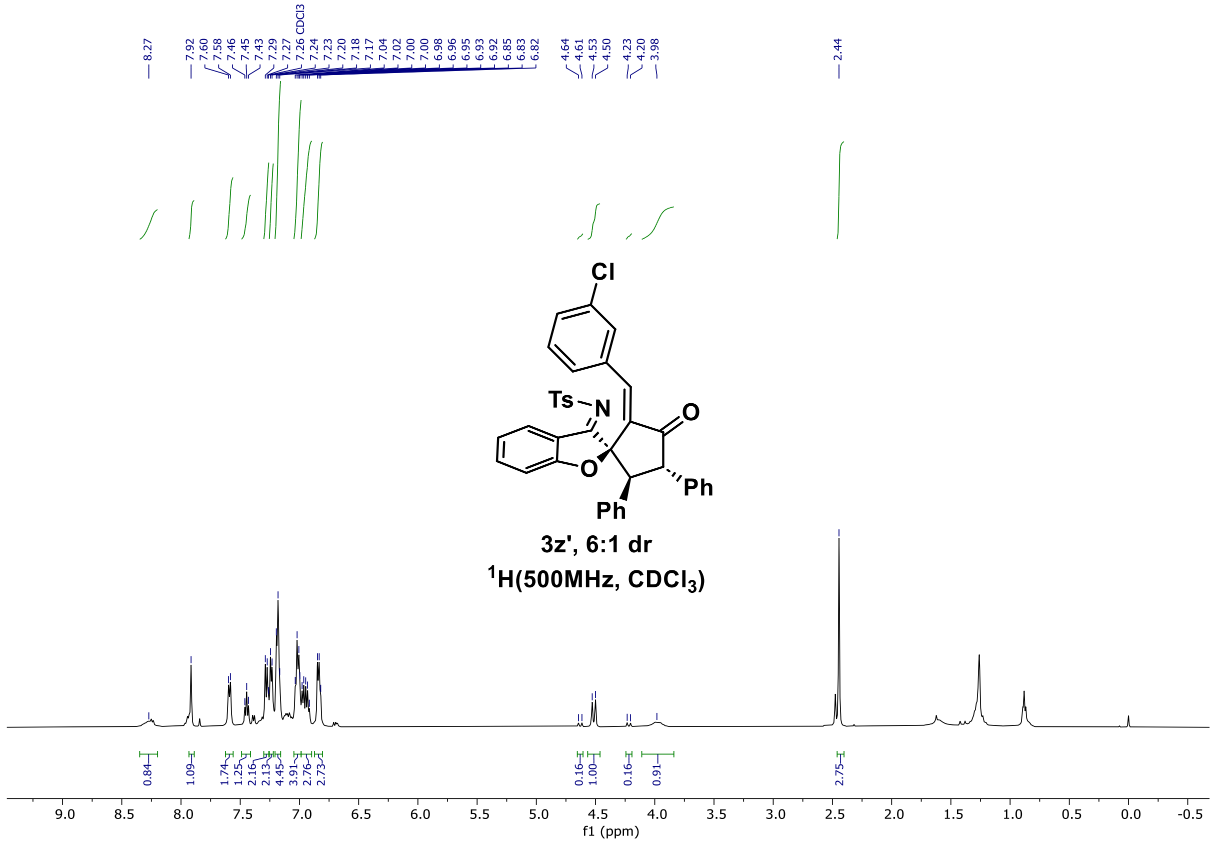
8.27
7.92
7.60
7.58
7.46
7.45
7.43
7.29
7.27
7.26 CDCl3
7.24
7.23
7.20
7.18
7.17
7.04
7.02
7.00
7.00
6.98
6.96
6.95
6.93
6.92
6.85
6.83
6.82
4.64
4.61
4.53
4.50
4.23
4.20
3.98
2.44
Cl
Ts
N
O
Ph
Ph
3z', 6:1 dr
1H(500MHz, CDCl3)
0.84
1.09
1.74
1.25
2.16
2.13
4.45
3.91
2.76
2.73
0.16
1.00
0.16
0.91
2.75
f1 (ppm)

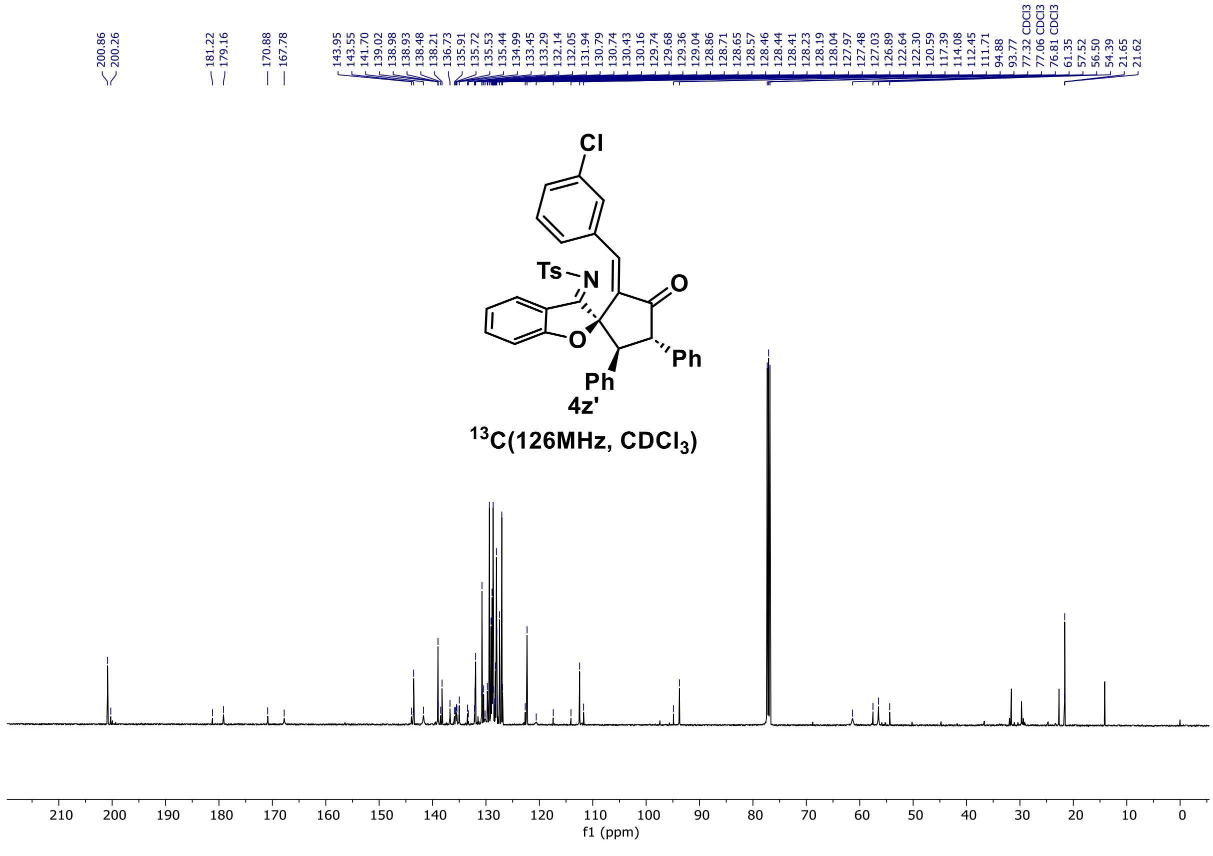
200.86
200.26
181.22
179.16
170.88
167.78
143.95
143.55
141.70
139.02
138.98
138.93
138.48
138.21
136.73
135.91
135.72
135.53
135.44
134.99
133.45
133.29
132.14
132.05
131.94
130.79
130.74
130.43
130.16
129.74
129.68
129.36
129.04
128.86
128.71
128.65
128.57
128.46
128.44
128.41
128.23
128.19
128.04
127.97
127.48
127.03
126.89
122.64
122.30
120.59
117.39
114.08
112.45
111.71
94.88
93.77
77.32 CDCl3
77.06 CDCl3
76.81 CDCl3
61.35
57.52
56.50
54.39
21.65
21.62
Cl
Ts
N
O
Ph
Ph
4z'
13C(126MHz, CDCl3)
f1 (ppm)

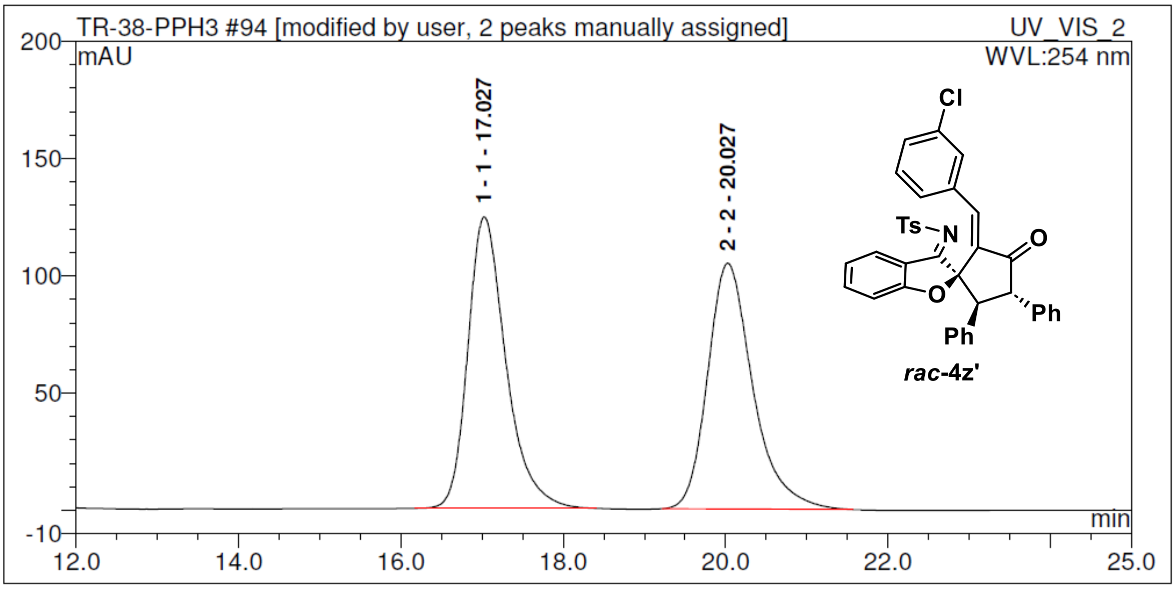

	Peak Name	Ret.Time (detected) min	Area mAU*min	Rel.Area(ident.) %	Height mAU	Amount
1	1	17.03	67.65067	49.99423939	124.281	n.a.
2	2	20.03	67.666	50.00576061	104.794	n.a.

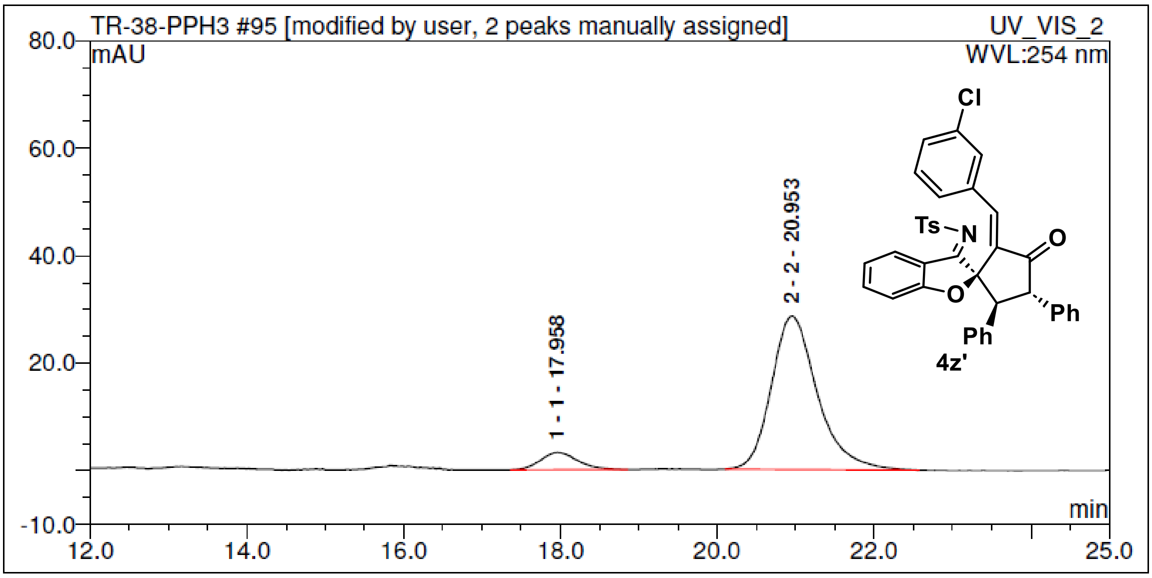

	Peak Name	Ret.Time (detected) min	Area mAU*min	Rel.Area(ident.) %	Height mAU	Amount
1	1	17.96	1.698089	8.073408595	3.14163	n.a.
2	2	20.95	19.335	91.92659141	28.491	n.a.

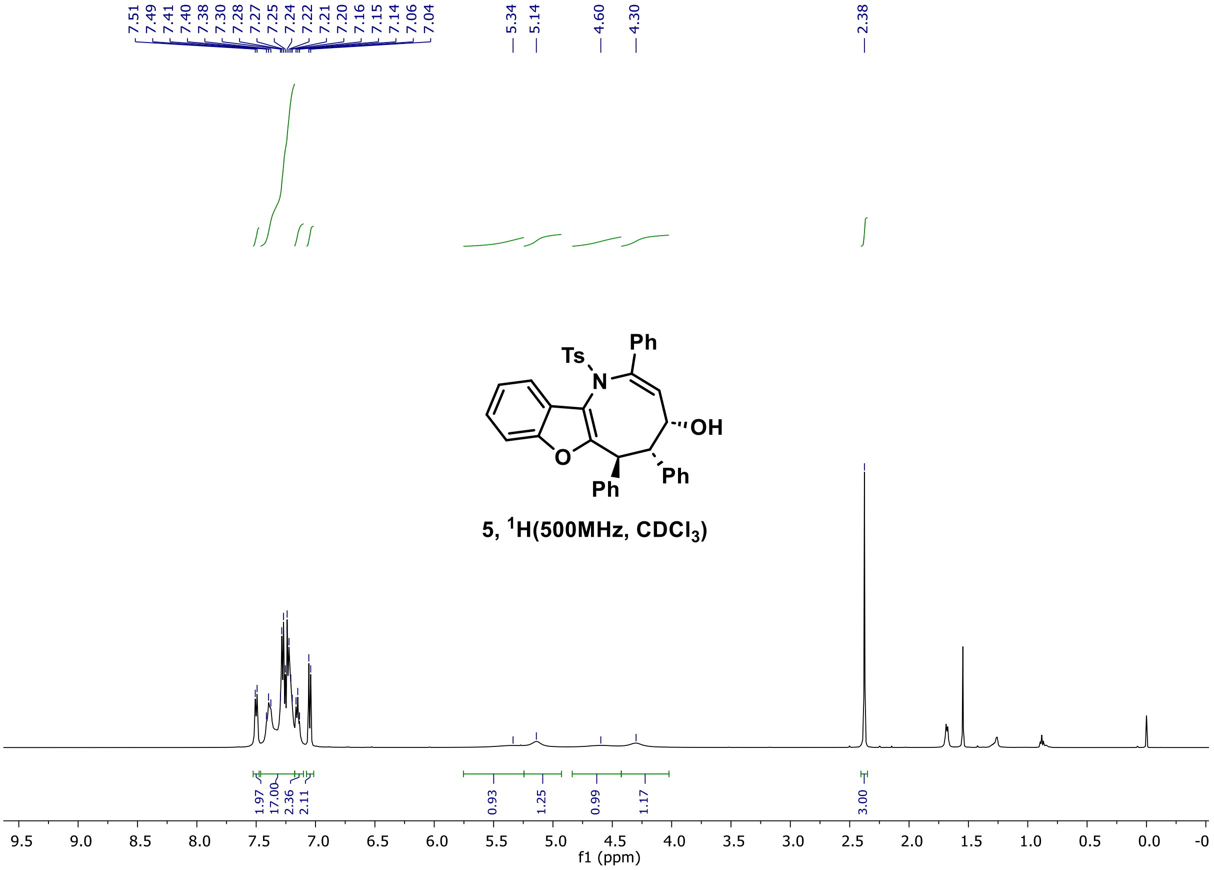
7.51 7.49 7.41 7.40 7.38 7.30 7.28 7.27 7.25 7.24 7.22 7.21 7.20 7.16 7.15 7.14 7.06 7.04
5.34 5.14 4.60 4.30 2.38
Ts
N
Ph
OH
O
Ph
Ph
5, ^{1}H(500MHz, CDCl$_3$)
1.97 17.00 2.36 2.11 0.93 1.25 0.99 1.17 3.00
9.5 9.0 8.5 8.0 7.5 7.0 6.5 6.0 5.5 5.0 4.5 4.0 3.5 3.0 2.5 2.0 1.5 1.0 0.5 0.0 -0
f1 (ppm)

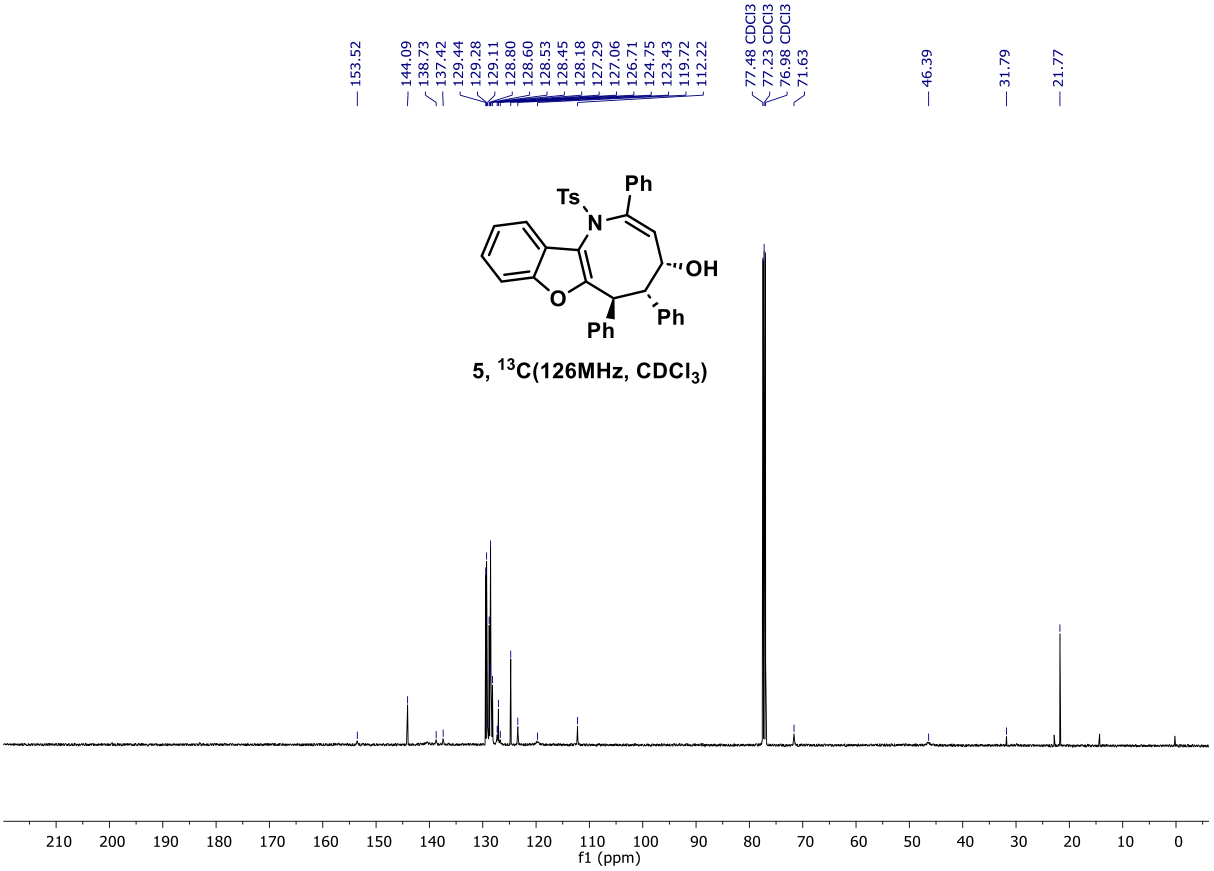
153.52 144.09 138.73 137.42 129.44 129.28 129.11 128.80 128.60 128.53 128.45 128.18 127.29 127.06 126.71 124.75 123.43 119.72 112.22
77.48 CDCl3 77.23 CDCl3 76.98 CDCl3 71.63
46.39 31.79 21.77
Ts
N
Ph
OH
O
Ph
Ph
5, ^{13}C(126MHz, CDCl$_3$)
210 200 190 180 170 160 150 140 130 120 110 100 90 80 70 60 50 40 30 20 10 0
f1 (ppm)

VT Experiment of 5:

As we observed no splitting between H_a, H_b and H_c proton in ^{1}H NMR spectra at 25 °C. To understand the stereochemistry, we perform variable temperature NMR experiments at 0 °C, -10 °C, -20 °C. A staked ^{1}H NMR at various temperature is given below.

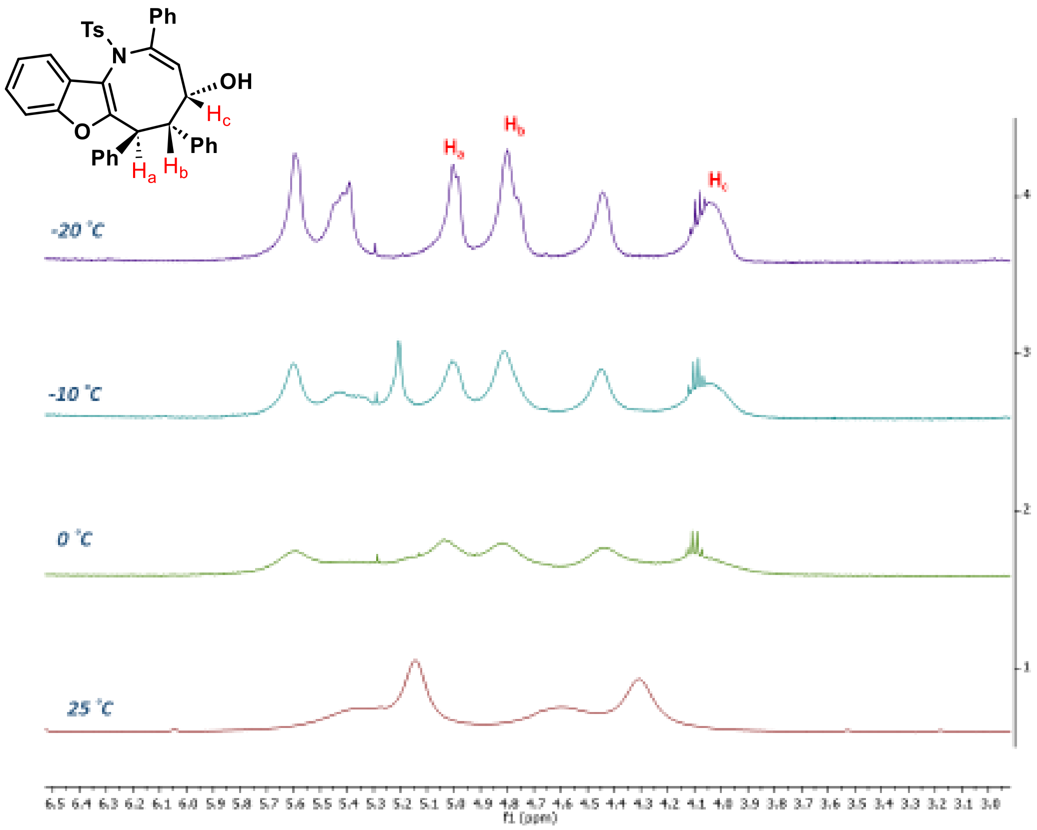

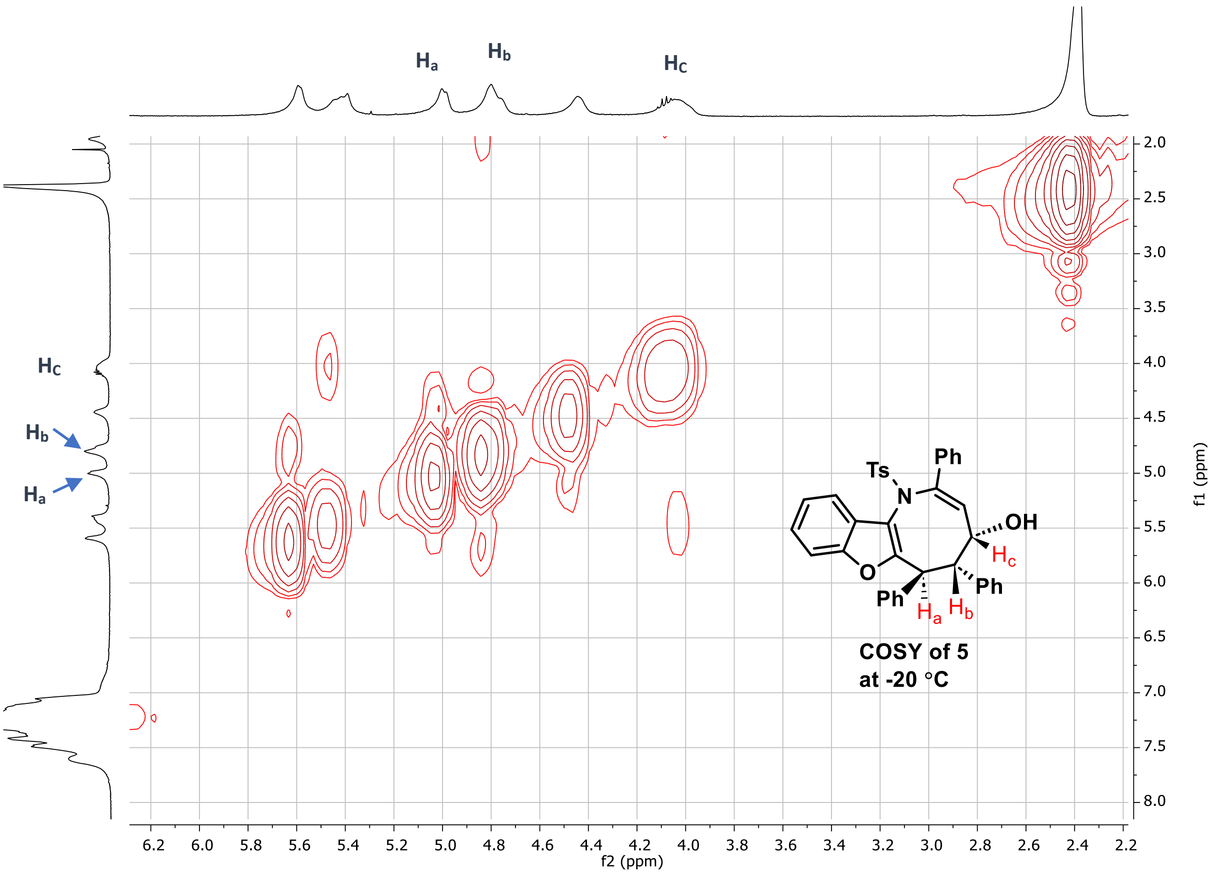
Ha
Hb
Hc
Ts
N
Ph
OH
O
COSY of 5
at -20 °C
f2 (ppm)
f1 (ppm)

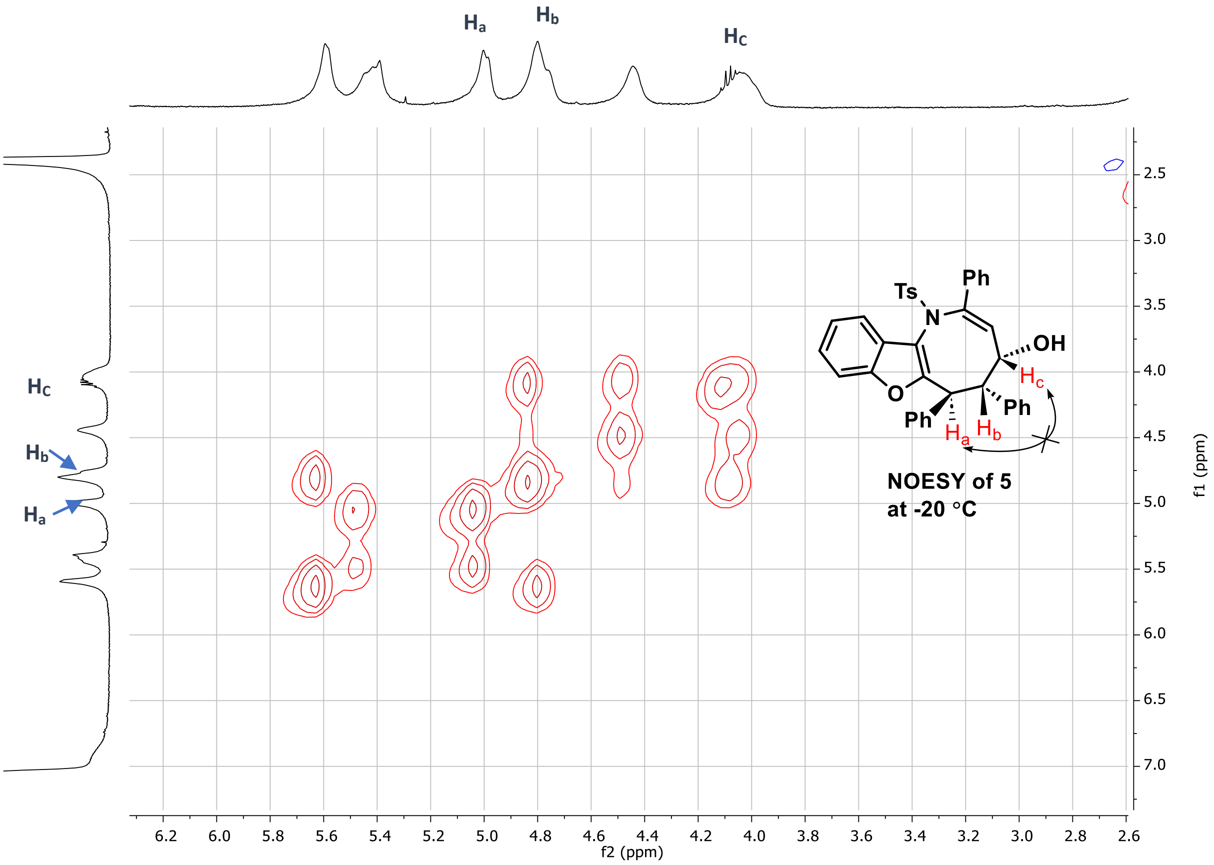
Ha
Hb
Hc
Ts
N
Ph
OH
O
NOESY of 5
at -20 °C
f2 (ppm)
f1 (ppm)

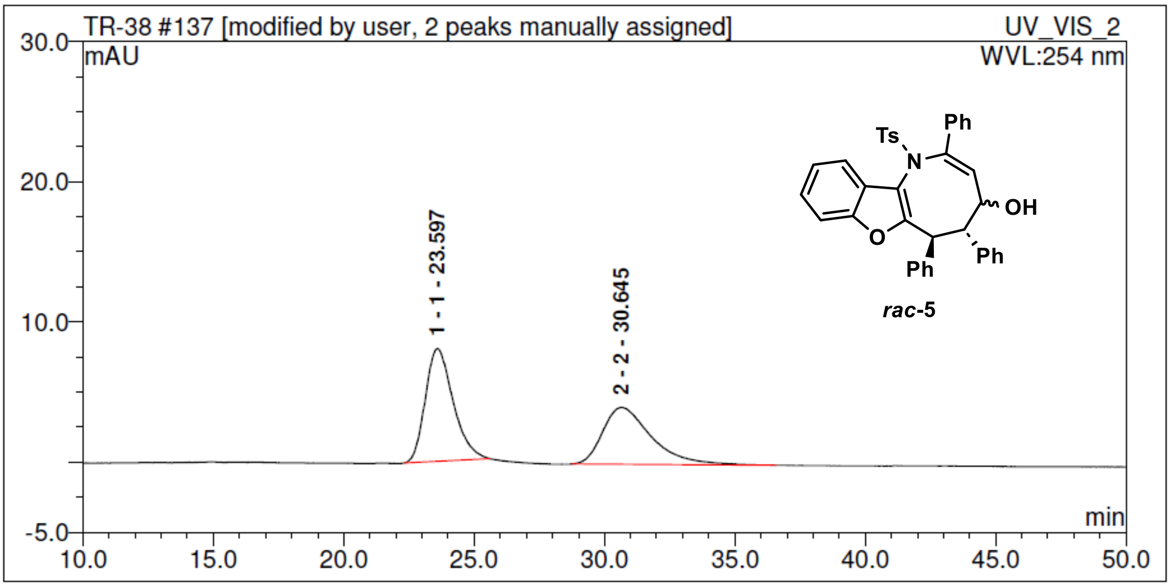

Peak Name	Ret.Time (detected) min	Area mAU*min	Rel.Area(ident.) %	Height mAU	Amount
1 1	23.60	9.915024	53.11033727	8.01853	n.a.
2 2	30.65	8.754	46.88966273	4.054	n.a.

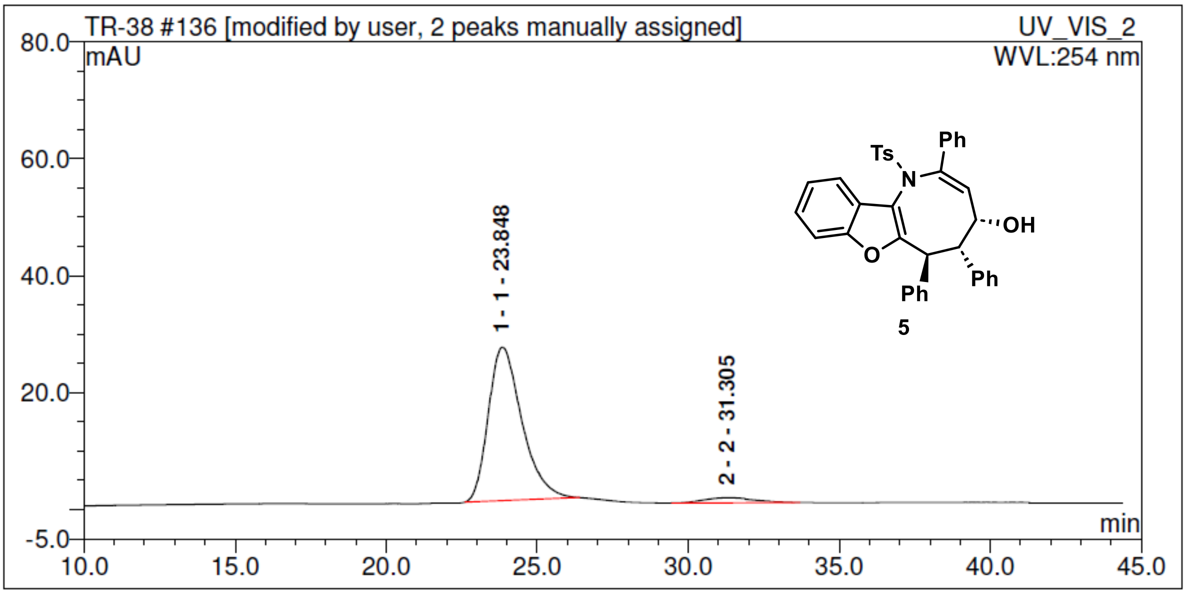

Peak Name	Ret.Time (detected) min	Area mAU*min	Rel.Area(ident.) %	Height mAU	Amount
1 1	23.85	33.87526	95.4346562	26.25407	n.a.
2 2	31.31	1.621	4.565343802	0.865	n.a.

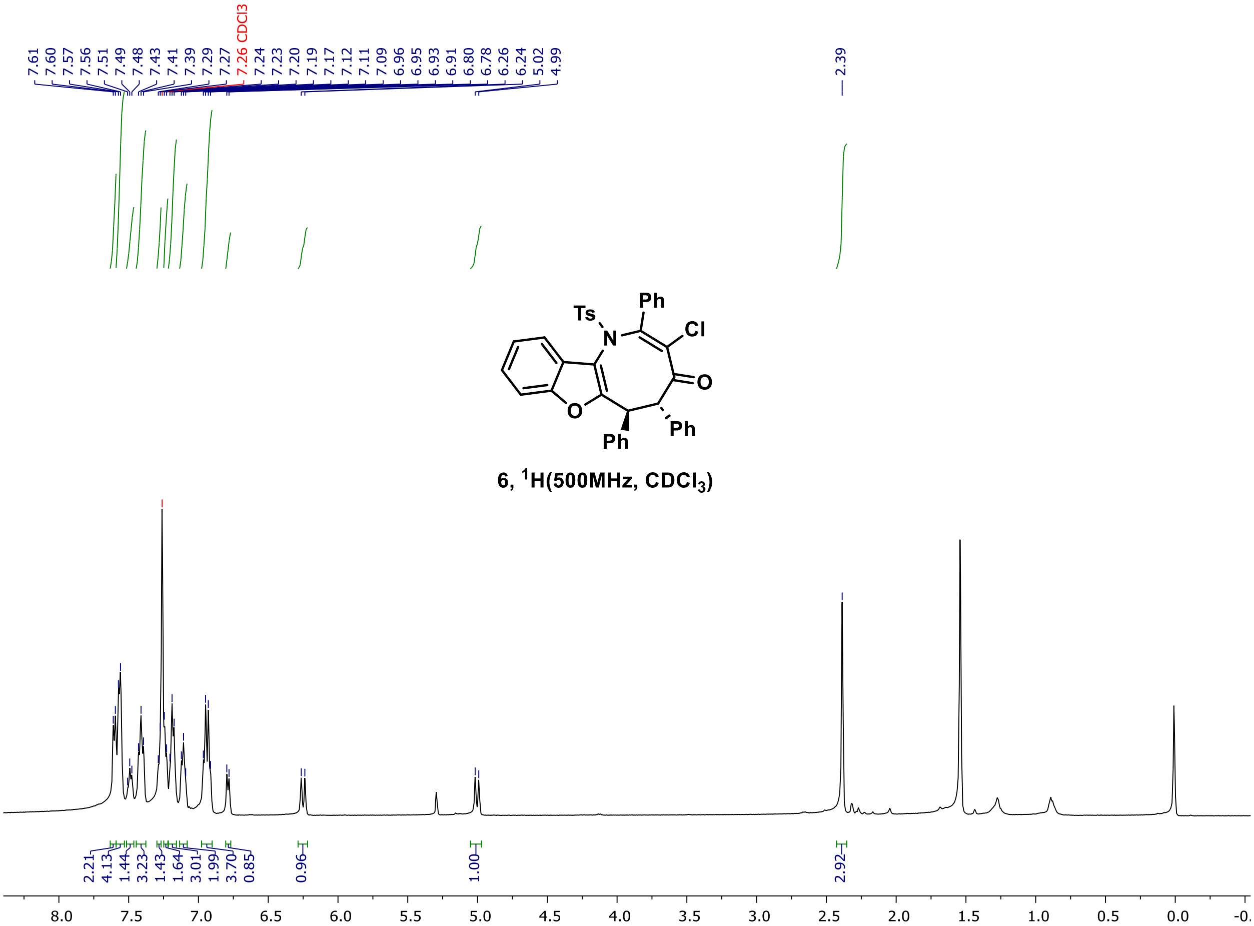
7.61 7.60 7.57 7.56 7.51 7.49 7.48 7.43 7.41 7.39 7.29 7.27 7.26 CDCl3 7.24 7.23 7.20 7.19 7.17 7.12 7.11 7.09 6.96 6.95 6.93 6.91 6.80 6.78 6.26 6.24 5.02 4.99
2.39
Ts
N
Ph
Cl
O
O
Ph
Ph
6, ^{1}H(500MHz, $CDCl_3$)
2.21 4.13 1.44 3.23 1.43 1.64 3.01 1.99 3.70 0.85
0.96
1.00
2.92
8.0 7.5 7.0 6.5 6.0 5.5 5.0 4.5 4.0 3.5 3.0 2.5 2.0 1.5 1.0 0.5 0.0

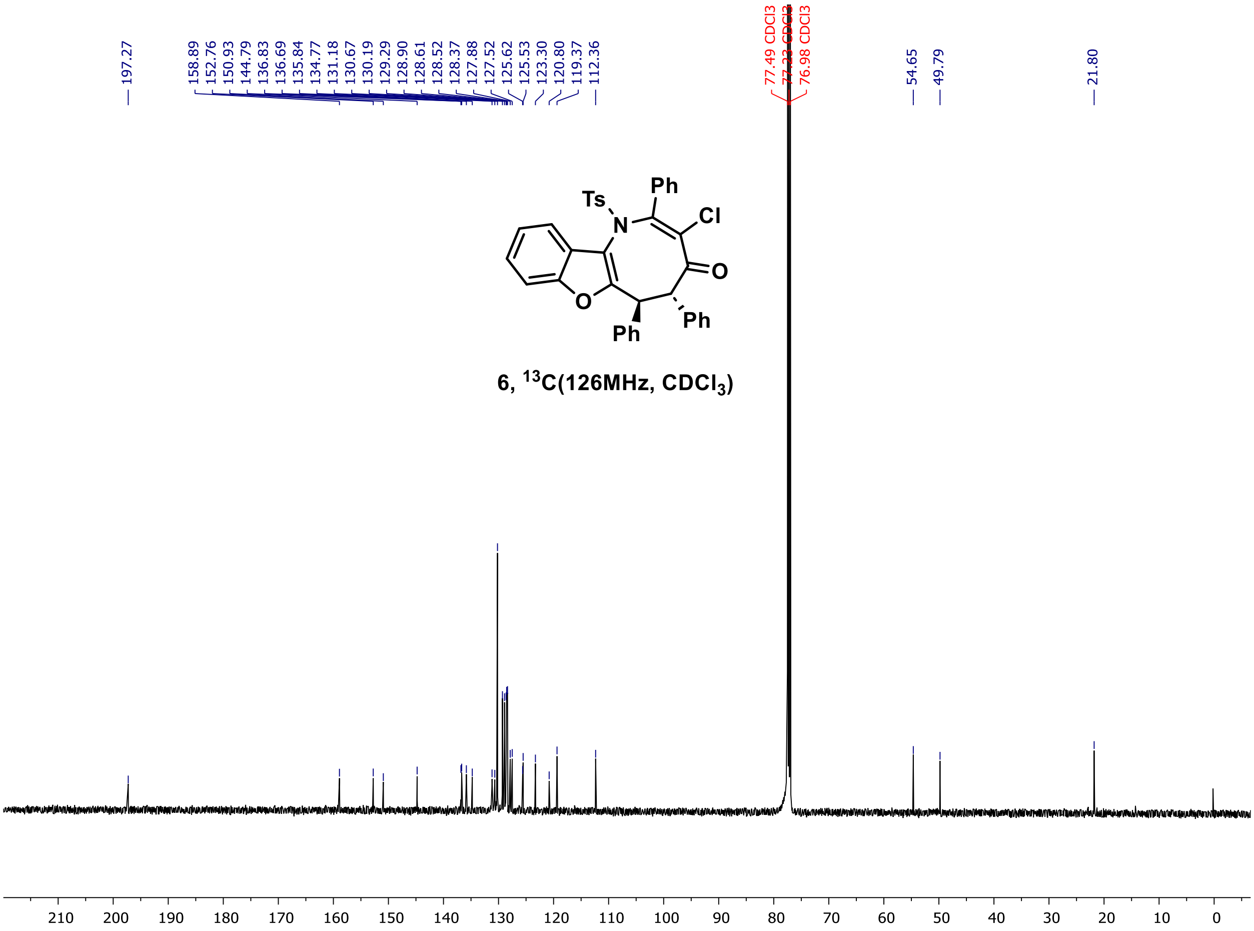
197.27
158.89 152.76 150.93 144.79 136.83 136.69 135.84 134.77 131.18 130.67 130.19 129.29 128.90 128.61 128.52 128.37 127.88 127.52 125.62 125.53 123.30 120.80 119.37 112.36
77.49 CDCl3 77.23 CDCl3 76.98 CDCl3
54.65
49.79
21.80
Ts
N
Ph
Cl
O
O
Ph
Ph
6, ^{13}C(126MHz, $CDCl_3$)
210 200 190 180 170 160 150 140 130 120 110 100 90 80 70 60 50 40 30 20 10 0

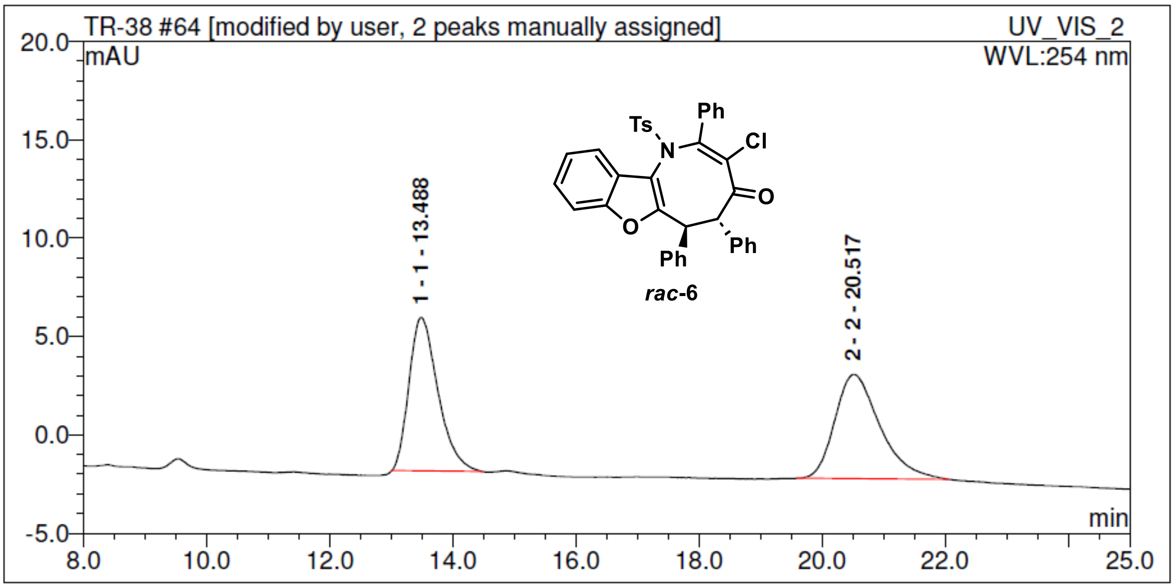

Peak Name		Ret.Time (detected) min	Area mAU*min	Rel.Area(ident.) %	Height mAU	Amount
1	1	13.49	4.259287	48.69296571	7.80147	n.a.
2	2	20.52	4.488	51.30703429	5.293	n.a.

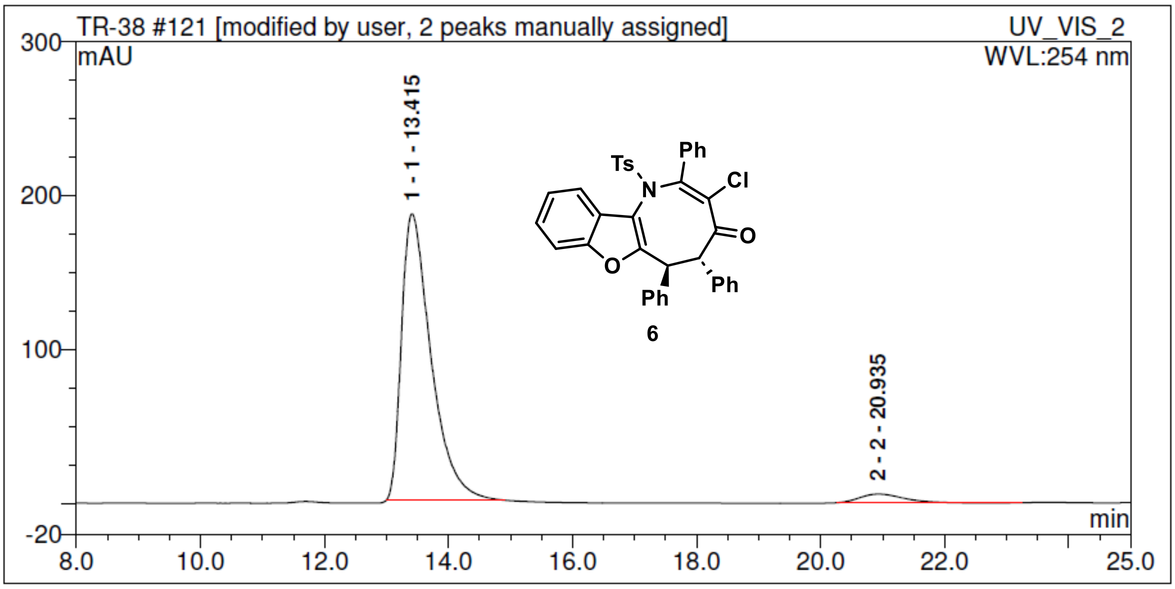

Peak Name		Ret.Time (detected) min	Area mAU*min	Rel.Area(ident.) %	Height mAU	Amount
1	1	13.42	102.5229	95.96243862	185.8448	n.a.
2	2	20.94	4.314	4.037561376	5.571	n.a.

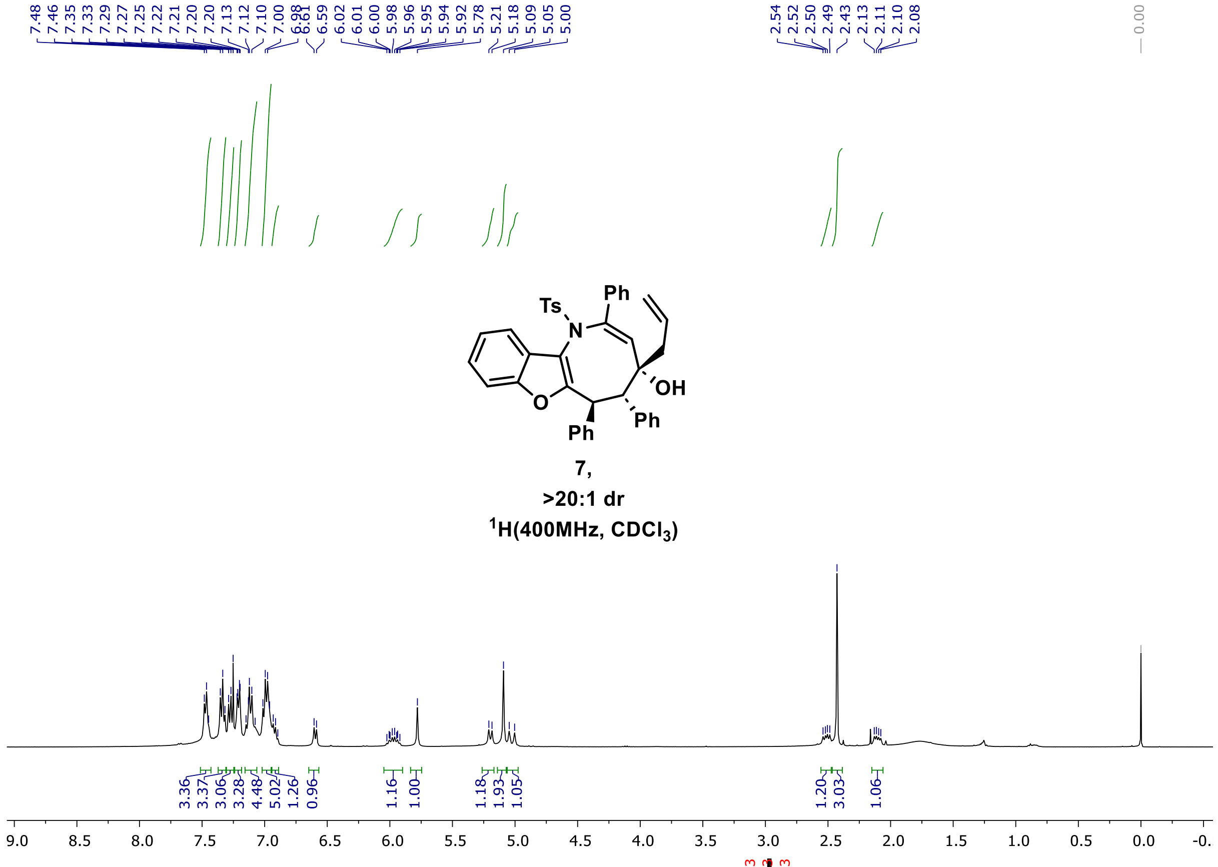
Ts
N
Ph
O
OH
Ph
Ph
7,
>20:1 dr
1H(400MHz, CDCl3)

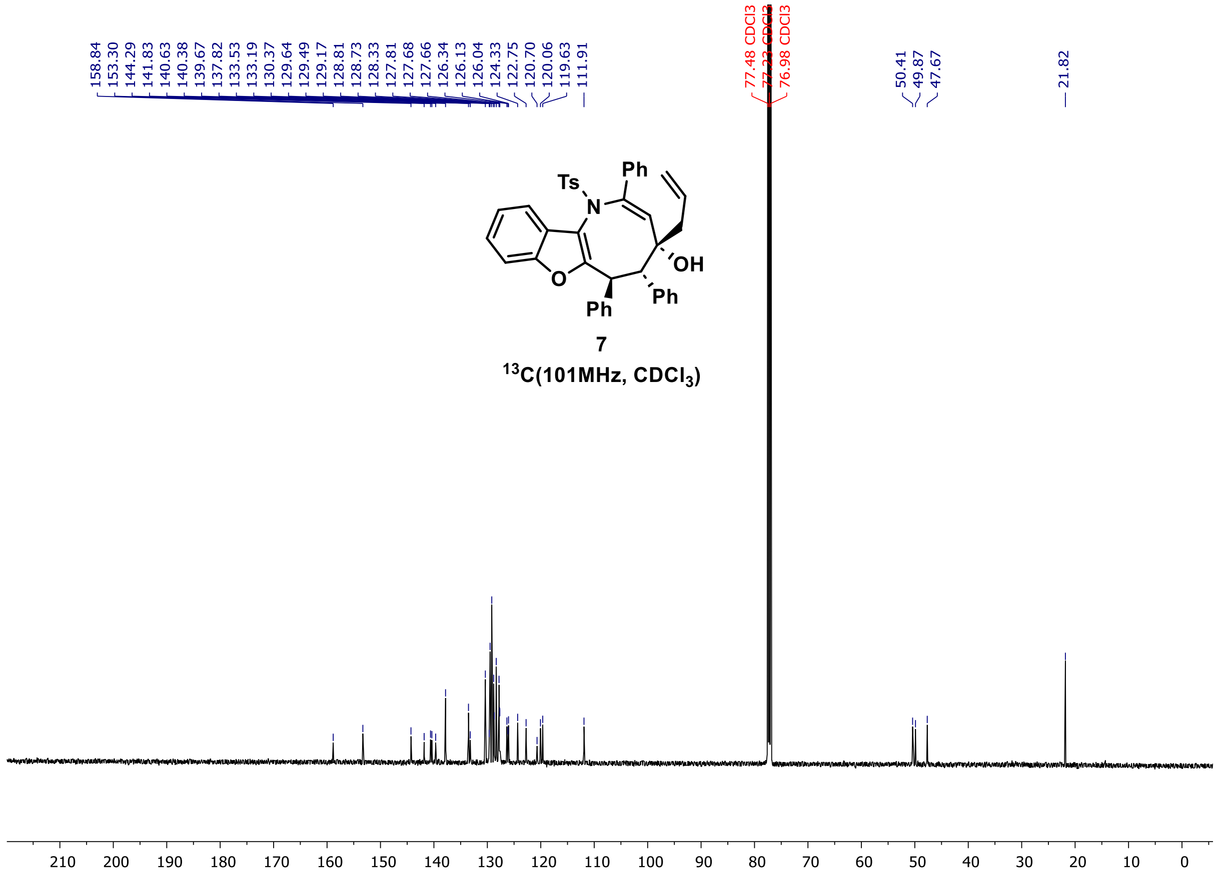
77.48 CDCl3
76.98 CDCl3
Ts
N
Ph
O
OH
Ph
Ph
7
13C(101MHz, CDCl3)

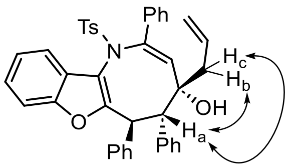
Ts
N
Ph
Hc
Hb
OH
Ha
O
Ph
Ph

NOESY of **7**

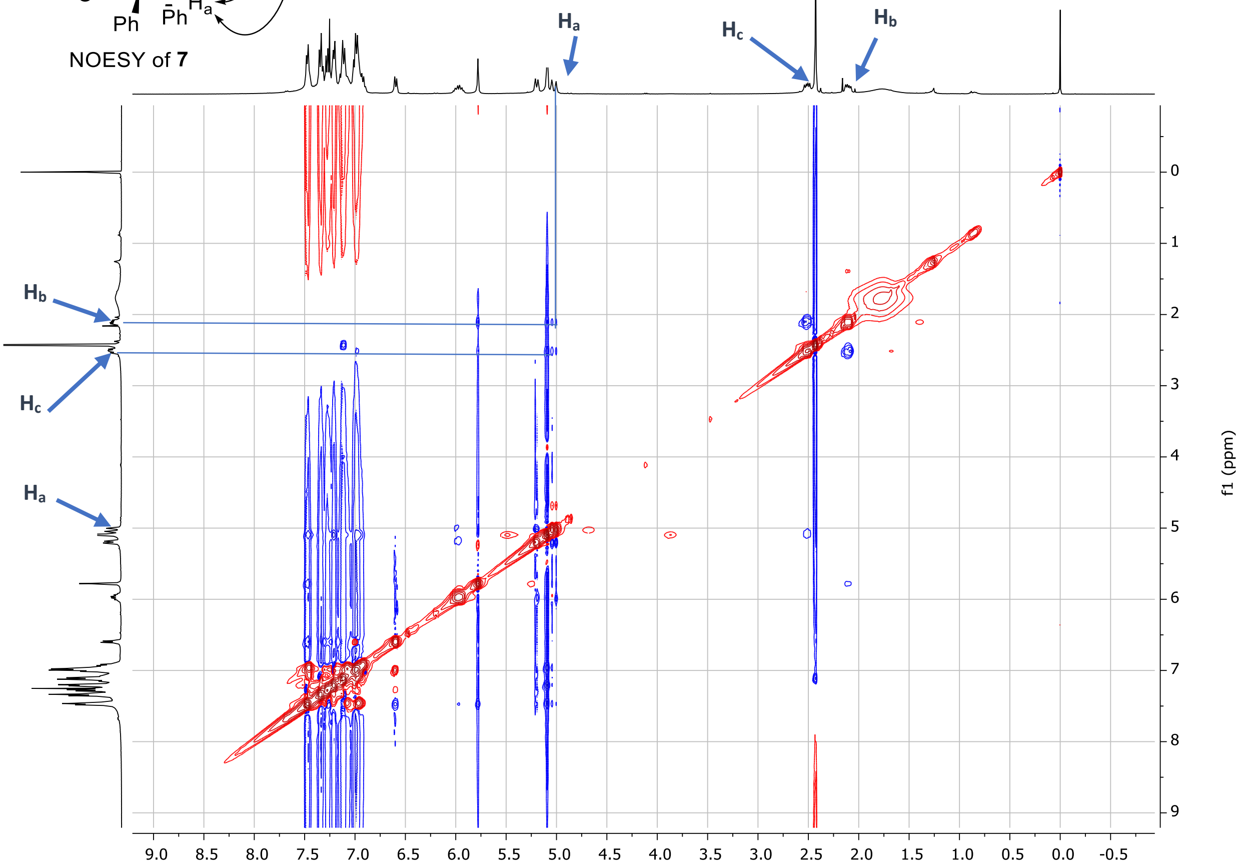
Ha
Hc
Hb
Hb
Hc
Ha
f1 (ppm)
9.0 8.5 8.0 7.5 7.0 6.5 6.0 5.5 5.0 4.5 4.0 3.5 3.0 2.5 2.0 1.5 1.0 0.5 0.0 -0.5
0 1 2 3 4 5 6 7 8 9

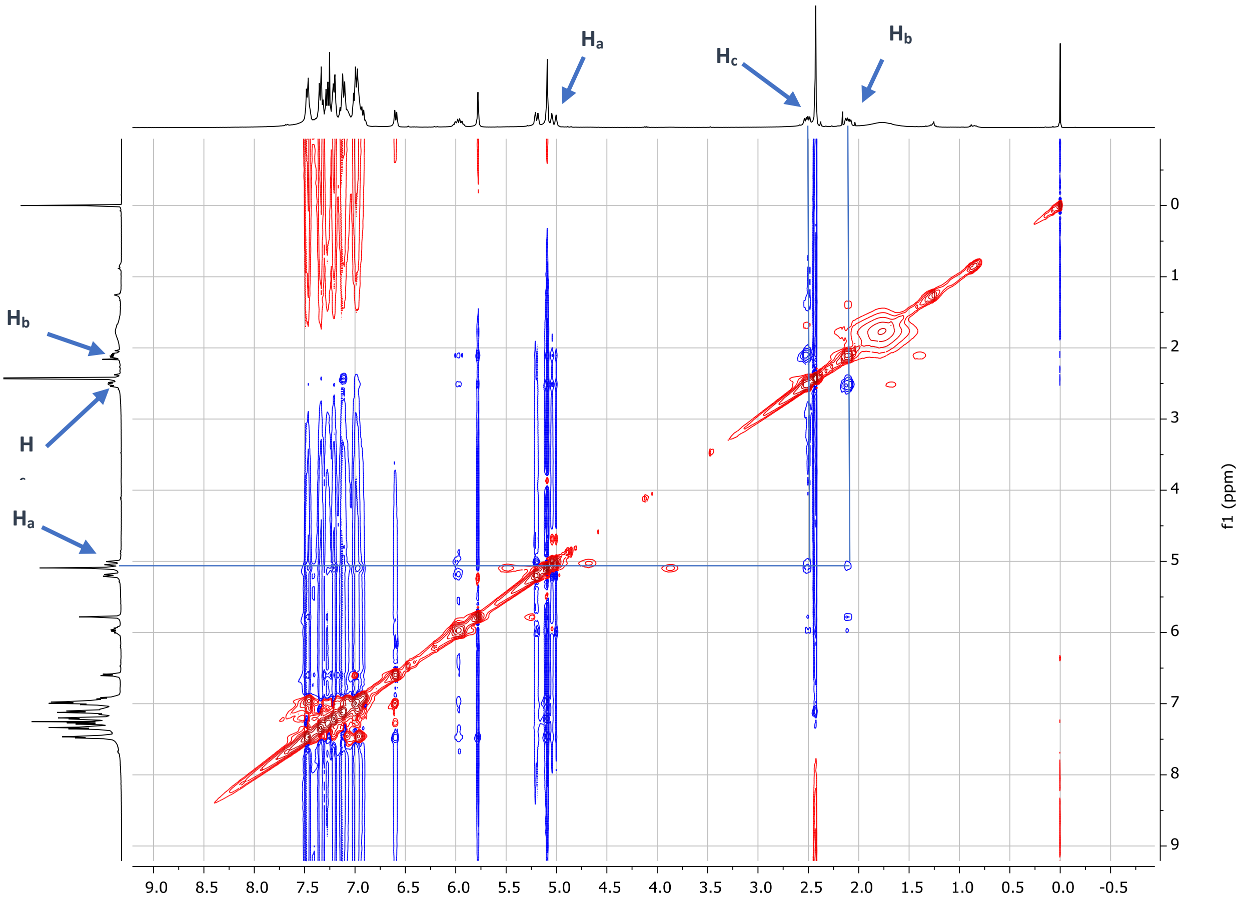
Ha
Hc
Hb
Hb
H
Ha
f1 (ppm)
9.0 8.5 8.0 7.5 7.0 6.5 6.0 5.5 5.0 4.5 4.0 3.5 3.0 2.5 2.0 1.5 1.0 0.5 0.0 -0.5
0 1 2 3 4 5 6 7 8 9

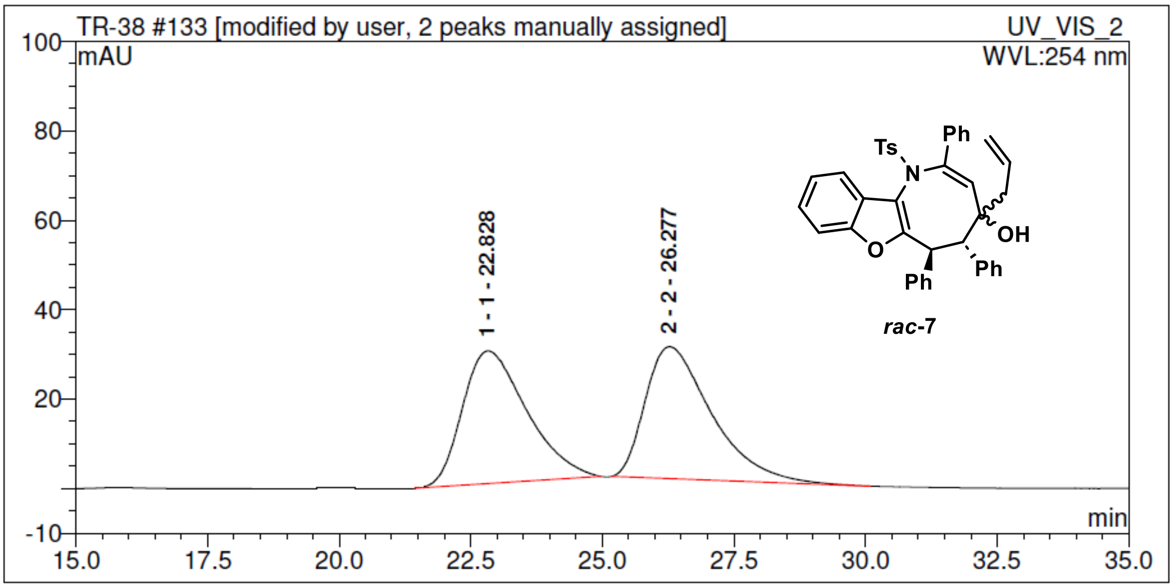

Peak Name	Ret.Time (detected)	Area	Rel.Area(ident.)	Height	Amount
	min	mAU*min	%	mAU	
1 1	22.83	41.96408	49.29883068	29.65025	n.a.
2 2	26.28	43.158	50.70116932	29.517	n.a.

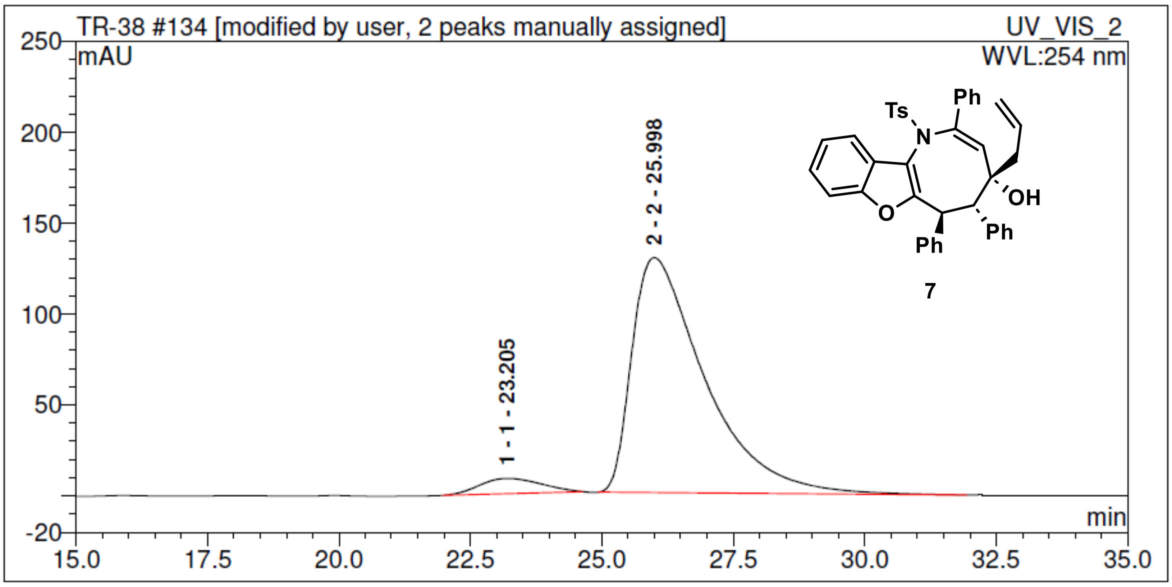

Peak Name	Ret.Time (detected)	Area	Rel.Area(ident.)	Height	Amount
	min	mAU*min	%	mAU	
1 1	23.21	11.29338	5.13931689	8.38958	n.a.
2 2	26.00	208.451	94.86068311	129.256	n.a.

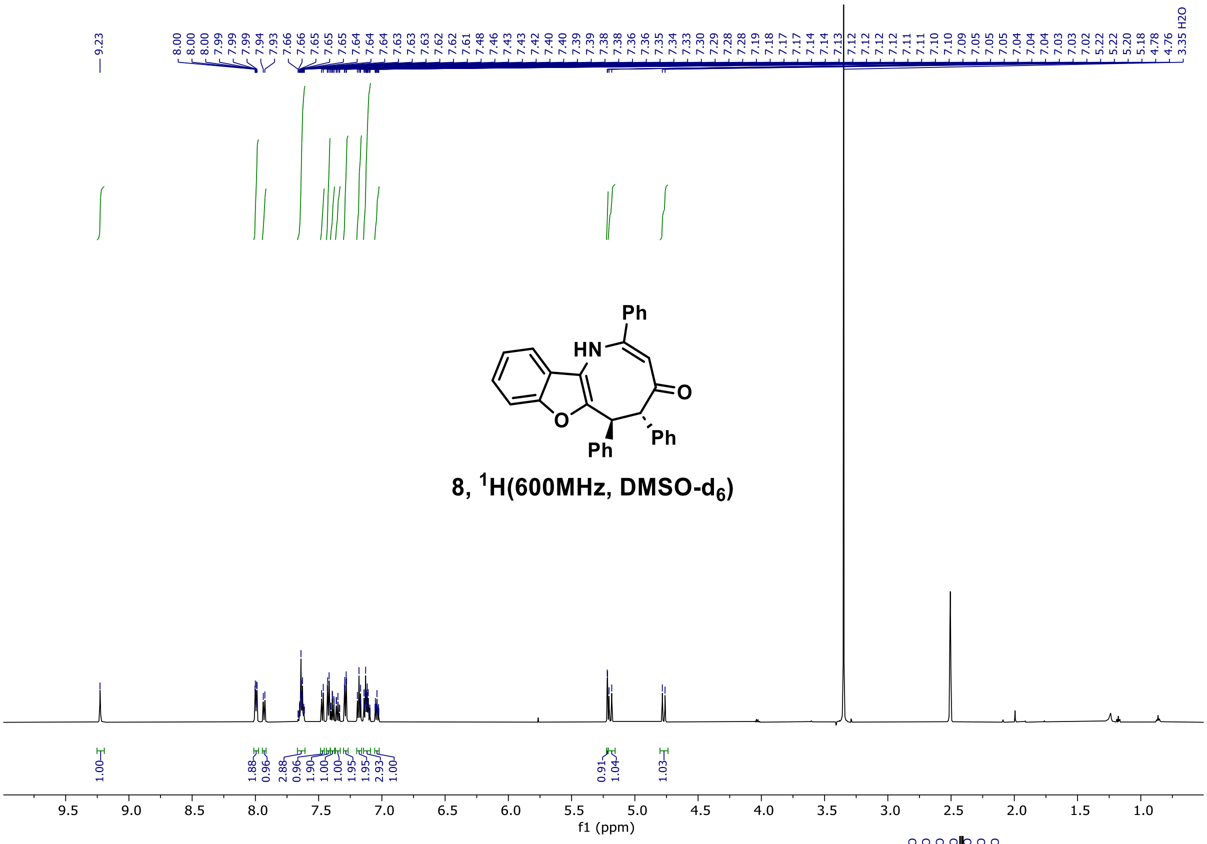

8, ^{1}H(600MHz, DMSO-d_6)

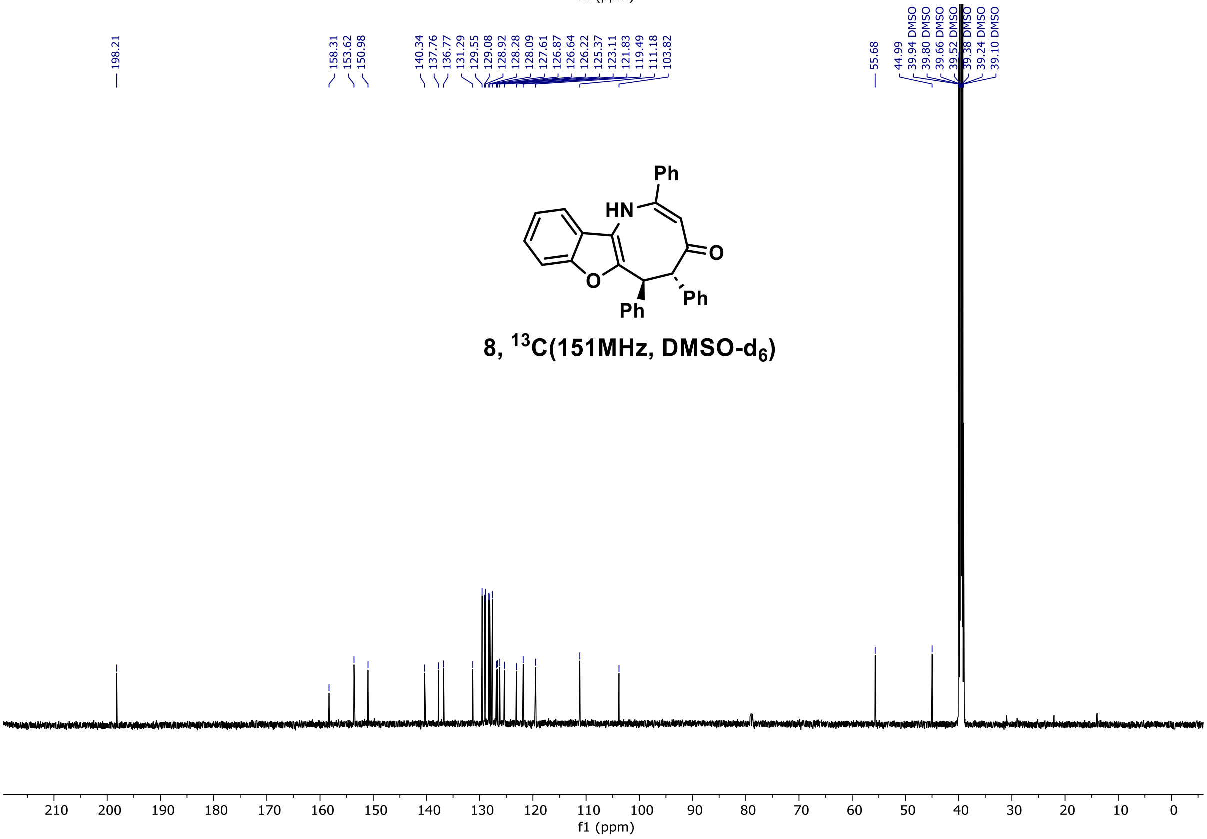

8, ^{13}C(151MHz, DMSO-d_6)

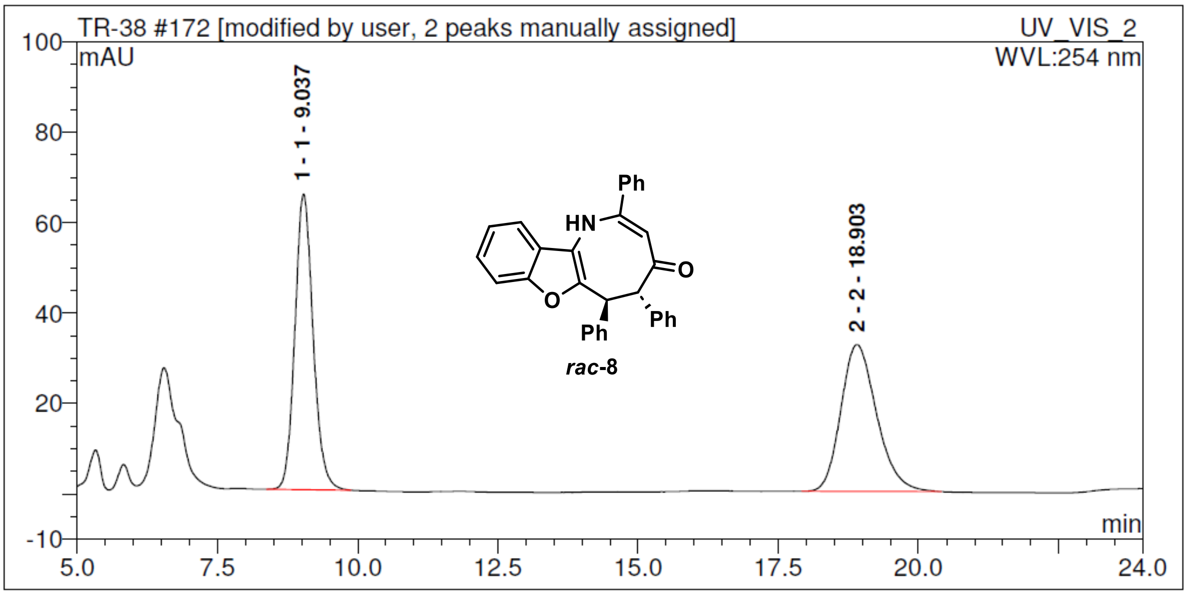

Peak	Name	Ret.Time (detected) min	Area mAU*min	Rel.Area(ident.) %	Height mAU	Amount
1	1	9.04	24.60834	50.57557064	65.36341	n.a.
2	2	18.90	24.048	49.42442936	32.472	n.a.

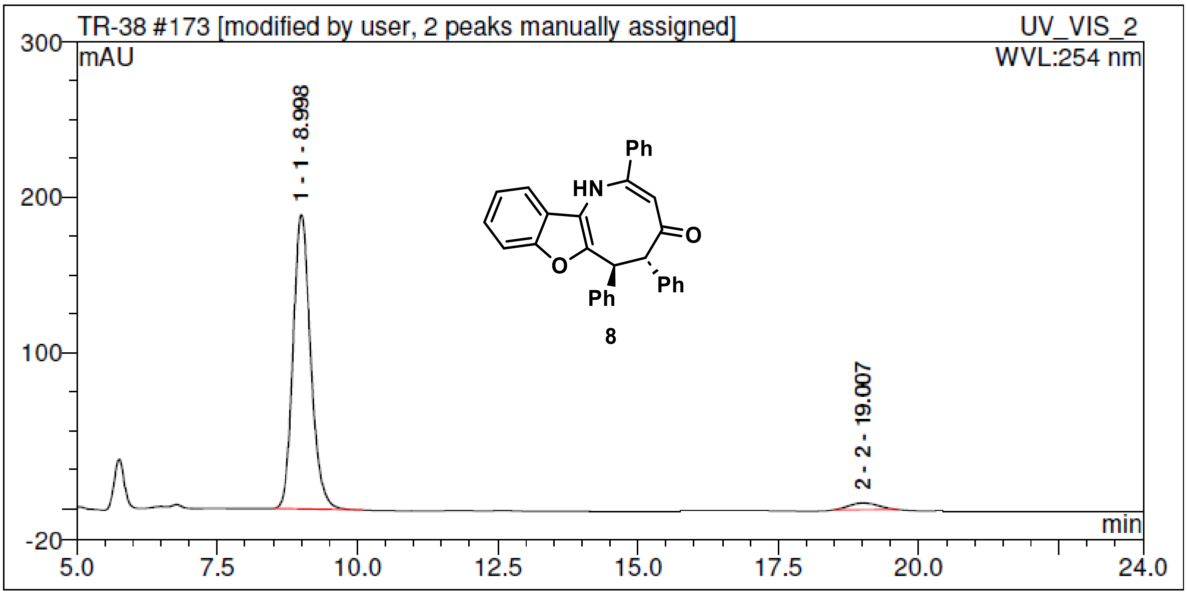

Peak	Name	Ret.Time (detected) min	Area mAU*min	Rel.Area(ident.) %	Height mAU	Amount
1	1	9.00	68.65999	96.23084124	188.926	n.a.
2	2	19.01	2.689	3.769158756	4.386	n.a.
